# Quantum computing formulation of some classical Hadamard matrix searching methods and its implementation on a quantum computer

**DOI:** 10.1038/s41598-021-03586-0

**Published:** 2022-01-07

**Authors:** Andriyan Bayu Suksmono, Yuichiro Minato

**Affiliations:** 1 The School of Electrical Engineering and Informatics, Institut Teknologi Bandung, Bandung, Indonesia; 2 Blueqat Inc., Tokyo, Japan

**Keywords:** Quantum information, Information theory and computation

## Abstract

Finding a Hadamard matrix (H-matrix) among all possible binary matrices of corresponding order is a hard problem that can be solved by a quantum computer. Due to the limitation on the number of qubits and connections in current quantum processors, only low order H-matrix search of orders 2 and 4 were implementable by previous method. In this paper, we show that by adopting classical searching techniques of the H-matrices, we can formulate new quantum computing methods for finding higher order ones. We present some results of finding H-matrices of order up to more than one hundred and a prototypical experiment of the classical-quantum resource balancing method that yields a 92-order H-matrix previously found by Jet Propulsion Laboratory researchers in 1961 using a mainframe computer. Since the exactness of the solutions can be verified by an orthogonality test performed in polynomial time; which is untypical for optimization of hard problems, the proposed method can potentially be used for demonstrating practical quantum supremacy in the near future.

## Introduction

### Background

A Hadamard matrix (H-matrix) is a binary orthogonal matrix with $\{-1, +1\}$ elements whose any distinct pair of its columns (or rows) are orthogonal to each other. Such a matrix only exists when it is square and the length of its column (row) is equal to 1, 2 or a multiple of four; i.e., for an $M \times M$ dimension H-matrix, then $M=1,2$ or $M=4k$ for a positive integer *k*. The reversed statement that for any positive integer *k* there is a H-matrix is also believed to be true, although neither a mathematical proof nor disproof yet exists. This is a long standing problem of the *Hadamard Matrix Conjecture*.

The H-matrix has been a subject of scientific and practical interests. First discovered and described by Sylverster in 1867^1^, it is further studied by Hadamard concerning its relationship with the determinant problem^2^. The orthogonal property and binaryness of its elements make it widely used in information processing and digital communications. The CDMA (Code Division Multiple Access) system employs Hadamard-Walsh code to reduce interference among their users, so that the capacity of the communication system is not badly deteriorated by the increasing number of its users^3,4^. The H-matrix was also used by Mariner 9 space-craft as its ECC (Error Correcting Code) for sending images of Mars to a receiving station located on Earth, thanks to its capability for long error correction^4,5^.

Some particular kinds of H-matrices can be found (constructed) easily, while others need huge computational resource to do. An H-matrix of size $M\times M$ is also called an *M*-order H-matrix. When *M* follows a particular pattern of $M=2^n$, where *n* is a positive integer, the matrix can be easily constructed by the Sylvester’s method of tensor product. Hadamard^2^ constructed the H-matrices of order 12 and 20, whose orders do not follow the $2^n$ pattern. It indicates that other orders than prescribed by the Sylvester’s method do exist. Paley showed the construction of H-matrix of order $M=4k$ where $k \equiv 1 \mod 4$ and $k \equiv 3 \mod 4$, which are known as the Paley Type I and Type II H-matrices, respectively^6^. In the formulation, he employed the method of quadratic residues in a Galois field *GF*(*q*), where *q* is a power of an odd prime number.

Various kinds of construction methods have also been proposed. A cocyclic technique, which is based on a group development over a finite group *G* modified by the action of a cocycle defined on $G \times G$, has been introduced by De Launey and Horadam^7,8^. The Hadamard matrices can be generated by this scheme when it is applied to binary matrices. A general introduction on the cocyclic methods are described by Horadam^9^ and recent progress are presented; among others by Alvarez et al.^10,11^. In developing a quantum computing based H-matrix searching method, we found that a simple and straight forward method will be a good starting point. Our methods described in this paper have been based on earlier techniques proposed by Williamson^12^, Baumert–Hall^13^, and Turyn^14^, which is suitable for this purpose. These three methods involve searching of particular binary sequences as an essential stage. In this paper, we will refer these methods to as classical H-matrix searching methods.

Although at a glance it looks simple, finding a H-matrix is actually a challenging task. To find a H-matrix of order 92, in 1961 three JPL (Jet Propulsion Laboratory) researchers employed a state-of -the-art computer at that time, i.e. the IBM/7090 Mainframe^15^. For matrix order under 1000, the most recent unknown H-matrix successfully found is the one with order 428, which was discovered in 2005 by using computer search of particular binary sequences^16^. The method described in the paper is of particular interest because the next unknown H-matrices, such as the one with order 668, possibly can be found by using the same method. The main reason they have not been found at this time is because of the huge computational resource needed to find such matrices, which grows exponentially by the order of the matrix.

Finding a H-matrix of order *M* among all of $O(2^{M^2})$ binary matrices, which we refer to as *H-SEARCH*, is a hard problem. We have proposed to find such a matrix by using a quantum computer considering its capability in solving hard problems^17^. Theoretically, a quantum computer will need $O(M^2)$ qubits in superposition to solve such a problem. However, in the existing quantum annealing processor, we need $O(M^3)$ due to extra ancillary qubits required to translate *k*-body terms into 2-body Ising Hamiltonian model. In this paper, we show that by adopting the classical searching methods, we can reduce the required computing resource, which for a quantum annealing processor implementing the Ising model, will become $O(M^2)$. We describe how to formulate the corresponding Hamiltonians related to the classical methods and show some results of order up to more than one hundred. We also describe how to further develop this technique to find higher order matrices, by managing the classical and quantum computing resources. In such a classical-quantum hybridized algorithm, the complexity of the classical part still grows exponentially, but the quantum part grows polynomially. We shows that this algorithm extends the capability of a pure quantum method with limited number of qubits, so that a few higher order of H-matrices can be found, compared to the pure quantum method that cannot be implemented on present days quantum computer.

Usually, solving an optimization problem by annealing or heuristic methods yields only an approximate solution, i.e., we can not sure that it is actually the optimal point, unless all of possible solutions are enumerated. However, enumeration of all possible solutions of a hard problem is an extremely laborious task. In contrast, the correctness of a solution in H-SEARCH can be verified easily in polynomial time; i.e., by evaluating the orthogonality of the found matrix (solution). If we consider the solution as a certificate, H-SEARCH behaves like an NP-complete problem because finding the solution is hard, but checking its correctness is easy. In this particular point of view, H-SEARCH is an interesting hard problem worth to consider in addressing practical quantum supremacy.

### A brief on quantum computing and finding H-matrices using quantum computers

Quantum computers are expected to have computational capability beyond their classical counterparts; a feature which is well known as *quantum speedup*^18^ or even *quantum supremacy*^19^. An important progress regarding this issue is the achievement of the Google researchers in 2019, who claimed that their Sycamore quantum processor needs only about 200 s to do a particular computational task; which is sampling random quantum circuits in this case, where a classical supercomputer would take about 10,000 years to perform^20^. In the next step, a capability of solving a real-life problems, where classical computers cannot do in a reasonable time, is desired. Creative thinking of building algorithms that can demonstrate such practical supremacy are needed.

**Figure 1:**
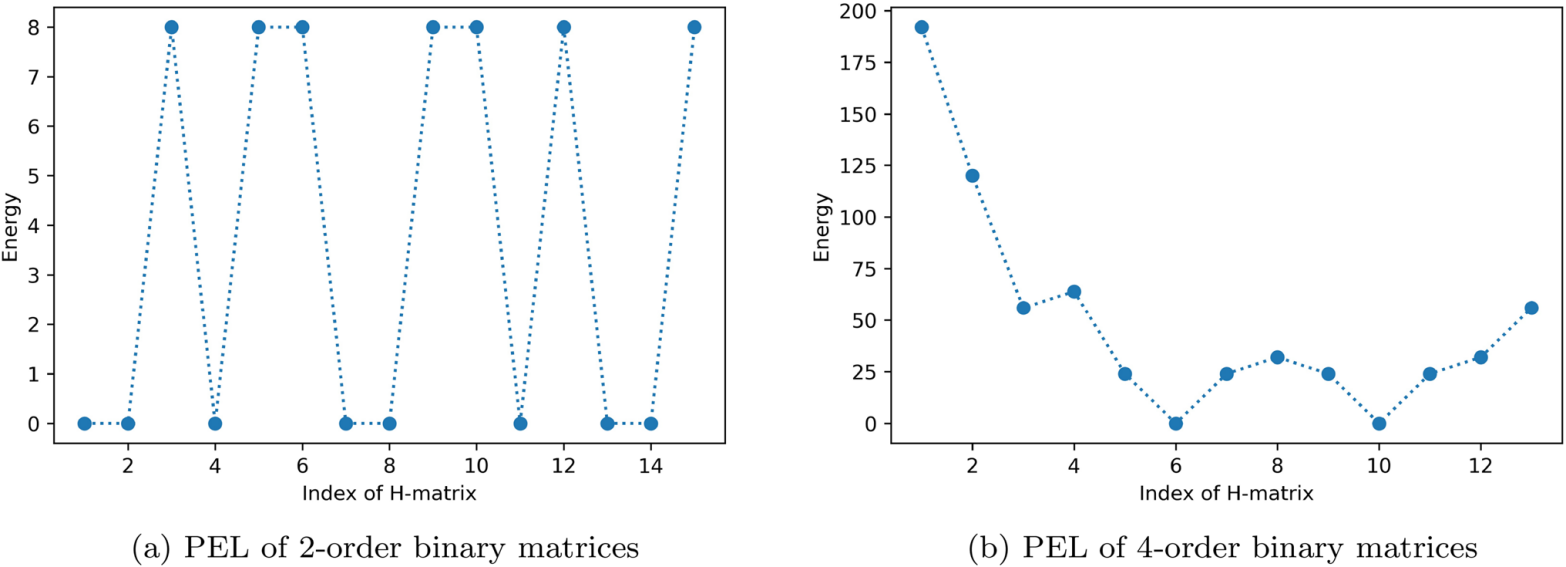
Potential Energy Landscape (PEL) of 2-order and 4-order binary matrices. The energy of the binary matrix *B* is calculated from sum of the squared off-diagonal of the D matrix, where $D=B^TB$, similar to Eq. (8) in our previous paper^21^. In (**a**), the 2-order binary matrices are generated and their energies are plotted against their indices. The indices are converted to the matrices after a binary to spin variable transform. As an example, for the second matrix with index 1, then the process is as follows: $$1 \rightarrow 0001 \rightarrow [1,1,1,-1] \rightarrow \left( {\begin{matrix} 1 &{} 1\\ 1 &{} -1\end{matrix}} \right)$$ . Zero energies matrices, such as ones with indices $1,2, 4, \ldots$ indicates orthogonal/Hadamard matrices. In (**b**) a few number of 4-order binary matrices neighboring (in term of Hamming distance) to an orthogonal matrix are plotted. Matrices with index 6 and 10 are Hadamard and therefore they are in global minima, while other matrices are not. An example of local minimum is given in the matrix with index 3. We can observe degeneration of energies in both of these 2 and 4 binary matrices.

The working principle of QAM computers are based on quantum annealing (QA)^22,23^, which is a quantum analog to the classical (thermal) annealing (CA). Whereas the CA works by gradually decreasing temperature with sometimes allowing the system to jump over higher energy, the QA seeks for the solution by quantum tunneling through the energy barrier. Energy landscape of the H-SEARCH problem’s Hamiltonian are degenerates; i.e., there are many equivalent binary matrices that have identical energy. Illustration of the potential energy landscapes (PEL) for 2-order and 4-order binary matrices are given in Fig. 1. Considering the PELs, quantum annealing approach is suitable to find the solution. We expect that the speed-up comes from the process of finding the minimum energy by quantum tunneling. Further analysis on how quantum computing can speed up a search algorithm is described by Farhi and Gutmann^24^. A comprehensive review on quantum annealing and analog quantum computation has been given by Das and Chakrabarti^25^.

**Figure 2:**
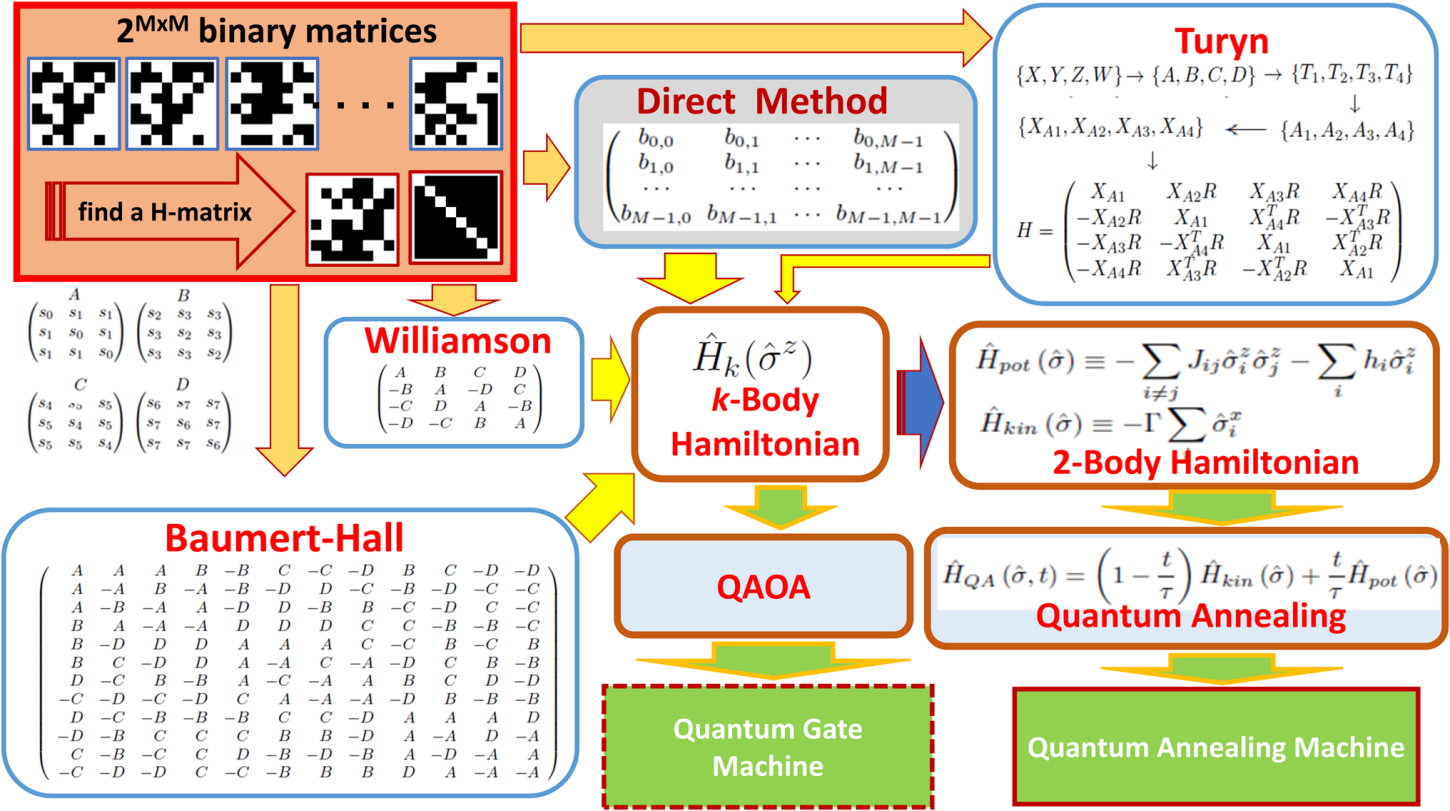
Quantum computing methods for solving the problem of finding H-matrix developed from classical methods. In the previous *direct method*^17^, we represent an *M*-order H-matrix to be found by an $M\times M$ binary variables, which becomes $O(M^2)$ logical qubits and $O(M^3)$ physical qubits including the ancillas, to be implemented on a quantum processor with Ising model. In this paper, we adopt three classical methods; i.e., the Williamson, Baumert–Hall, and Turyn, into quantum computing algorithms by formulating their corresponding quantum Hamiltonians. Each of the corresponding quantum computing methods developed in this paper needs *O*(*M*) logical qubits, which translates into  $O(M^2)$ physical qubits when they are implemented on a QAM (Quantum Annealing Machine) type processor. In a QGM (Quantum Gate Machine) processor, the number of required qubits will be proportional to the number of logical qubits in the Hamiltonians, i.e. *O*(*M*). We can employ QAOA (Quantum Approximate Optimization Algorithm) to implement the proposed methods in the QGM processor.

In general, existing quantum computers can be categorized into the universal quantum gate (QGM-Quantum Gate Machine) and quantum annealer (QAM-Quantum Annealing Machine). Regardless some issues related to noise and other non-ideal conditions, both of these types of quantum processors have been built and are accessible by public users through the Internet. The implementation scheme of the proposed methods for both of these kinds of quantum computers are illustrated in Fig. 2. The *direct method*; which works for QAM that has been described in our previous paper^17^, will be used as a reference. Three main proposed quantum computing methods are derived from non-quantum computing/classical H-matrix construction/searching methods, which we will referred to as the Williamson, Baumert–Hall, and Turyn methods.

The QAM processor, such as the D-Wave, only accepts problems in the form of a 2-body Hamiltonian that generally can be expressed by

1 $$\begin{aligned} {\hat{H}}_{pot} \left( {\hat{\sigma }} \right) \equiv -\sum _{i\ne j} J_{ij} {\hat{\sigma }}_i^z {\hat{\sigma }}_j^z -\sum _i h_i {\hat{\sigma }}_i^z \end{aligned}$$

which is a Hamiltonian of an Ising system, where $J_{ij}$ is a coupling constant or interaction strength between a spin at site *i* with a spin at site *j*, $h_j$ is magnetic strength at site *j*, and $\{{\hat{\sigma }}_i^{\alpha }\}$ are Pauli’s matrices of directions $\alpha =\{x,y,z\}$ at site-*i*. The processor performs quantum annealing by introducing a transverse field given by

2 $$\begin{aligned} {\hat{H}}_{kin}\left( {\hat{\sigma }} \right) \equiv -\Gamma \sum _i {\hat{\sigma }}_i^x \end{aligned}$$

which is evolved over time according to the following equation

3 $$\begin{aligned} {\hat{H}}_{QA}\left( {\hat{\sigma }}, t \right) =\left( 1-\frac{t}{\tau } \right) {\hat{H}}_{kin} \left( {\hat{\sigma }} \right) + \frac{t}{\tau }{\hat{H}}_{pot}\left( {\hat{\sigma }} \right) \end{aligned}$$

where $t \in [0,\tau ]$ denotes time^22,26^. The problem to solve should be encoded in ${\hat{H}}_{pot}$, which is represented by the Ising’s coefficient $J_{ij}$ and $h_i$ for each of the problem. Some optimization problems have been solved by the quantum annealing methods; among others are: graph isomorphism^27^, wireless network optimization^28^, nurse scheduling problem^29^, hand written digit recognition^30^, computational biology^31^, and hydrologic inverse analysis^32^.

In a QAM, the formulation of the H-SEARCH is started by calculation of its energy function *E*(*s*) as a function of binary variables $s \in \{-1,+1\}$. For conciseness, we will represent the value of *s* by its signs $\{-, +\}$. In general, *E*(*s*) might contain high order *k*-body interaction terms so that we will denote it by $E_k(s)$, whereas the Ising model allows only up to 2-body terms in $E_2(s)$. To obtain the 2-body expression, and eventually a 2-body quantum Hamiltonian ${\hat{H}}_2\left( {\hat{\sigma }}\right)$, a sequence of transforms given by the following *construction diagram* should be conducted^17^,

4 $$\begin{aligned} E_k(s) \rightarrow E_k(q) \rightarrow E_2(q) \rightarrow E_2(s)\rightarrow {\hat{H}}_2\left( {\hat{\sigma }}\right) \end{aligned}$$

where $q\in \{0,1\}$ is a Boolean variable. Actually both of *s* and *q* are binary variables, but with different values. For now on, we will refer $s \in \{-1,+1\}$ as spin variable and $q \in \{0,1\}$ as Boolean variable.

In the previous paper^17^, implementation of an *M*-order H-matrix on a QAM needs $M^2$ number of logical (binary) variables and additional $M^2\times (M-1)/2$ ancillary variables (*ancillas*) so that the overall complexity is $O(M^3)$. In this paper, by adopting classical H-matrix construction/searching methods, we can reduce the required number of variables significantly into $O(M^2)$ which enables the search of higher order H-matrices than before. In the followings, we will address three quantum H-SEARCH methods, which are derived from the classical methods of Williamson, Baumert–Hall, and Turyn. For each of these methods, we derive their corresponding Hamiltonians based on some criteria that are specifics for each of the cases. Low order cases can be calculated by hand, while higher order ones should be calculated by a computer through symbolic computing due to the large number of terms and variables which are involved. The complete lists and expressions of the Hamiltonians are provided in the Supplementary Information section.

In the QGM quantum computing, we can employ QAOA (Quantum Approximate Optimization Algorithm)^33^, which is well-suited for solving an optimization problem on NISQ (Noisy Intermediate-Scale Quantum) processors. In principle, the general *k*-body Hamiltonian can directly be implemented on a QGM. Therefore, the required number of physical qubits will be about the same as the number of logical qubits. However, since the implementation needs direct connection to the actual machine, which is not available for us at this time, we will not address it in the current paper.

## Results

### Williamson based quantum computing method

The Williamson’s method builds a matrix *W* of size $4k \times 4k$ from four sub-matrices *A*, *B*, *C*, *D* each of size $k \times k$^4,12,34^. Any pair of these sub-matrices are commutative. The orthogonality property of *W* will be satisfied when

5 $$\begin{aligned} V \equiv A^TA + B^TB + C^TC + D^TD = 4k I_k \end{aligned}$$

where $V=I_k$ is a $k \times k$ identity matrix. Then, the problem becomes choosing the elements of $s_i \in \{-1,+1\}$ in the sub-matrices that makes the orthogonality condition in Eq. (5) is satisfied. Further simplification and efficiency of the number of variables can be achieved when we choose the sub-matrices which are symmetric and circular.

**Figure 3:**
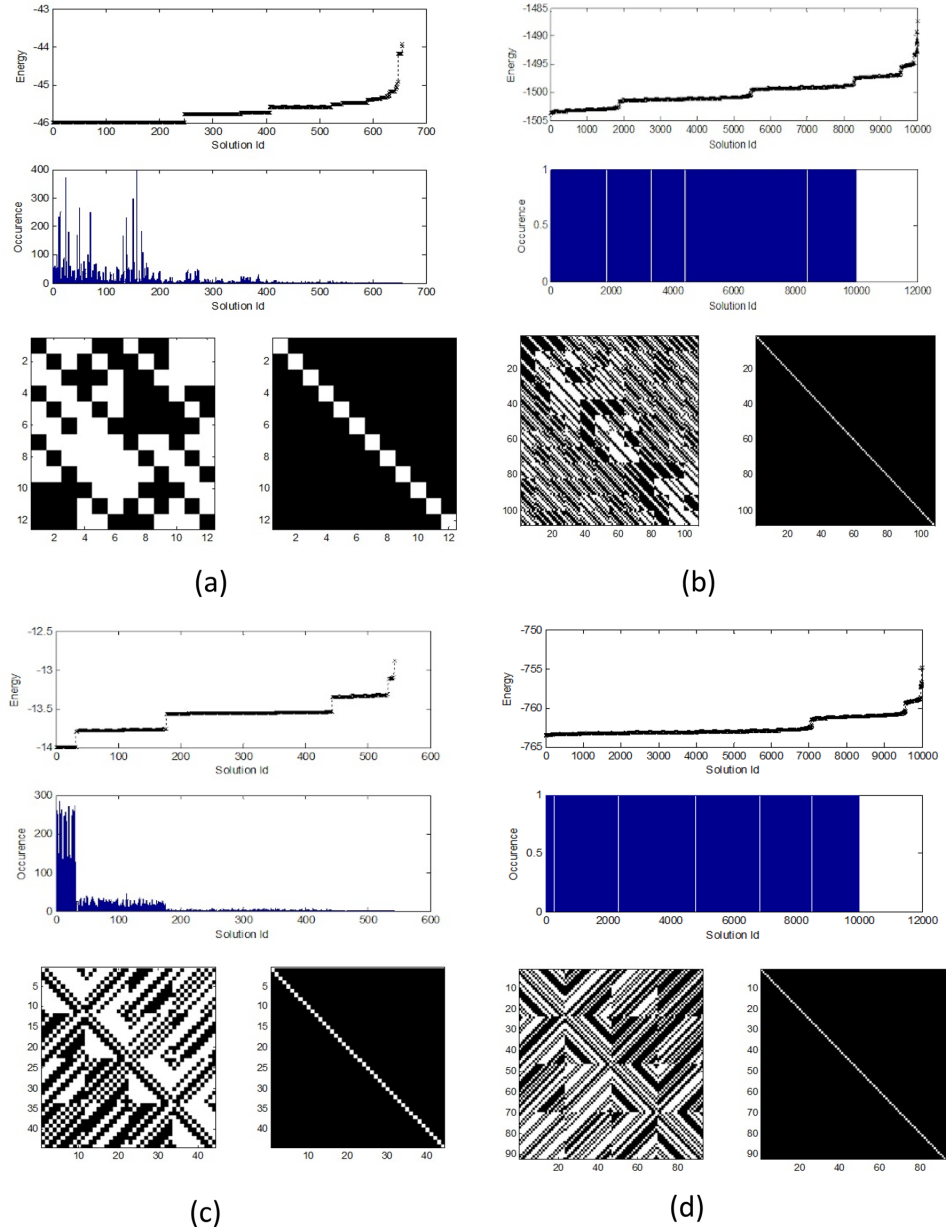
Experiment results of finding H-matrices using a quantum computer (D-Wave): (**a**) Williamson based method yields a 12 order H-matrix, (**b**) Baumert–Hall based method gives 108 order H-matrix, (**c**) Turyn based method resulting a 44 order H-matrix, and (**d**) extension of the Turyn based method that yields a 92 order H-matrix. Top parts show running statistics of the quantum processor. Bottom parts show the obtained H-matrices at the left hand side and corresponding indicator matrix at the right hand side. White pixels in the matrix indicates $+1$, whereas the black one is $-1$. White pixels in the indicator matrix correspond to *M*; which is the order of the matrix, and the black ones are zeroes. Running statistics shows that the solutions tend to cluster around low energy for low order case of 12 and 44, whereas they tend to uniformly distributed for high order cases of 108 and 92.

By imposing the orthogonality conditions, the commutativity among the sub-matrices, and the non-negativity of the energy, we arrive to the following *s*-dependent energy function

6 $$\begin{aligned} E_k(s)=\sum _{i=0}^{k-1} \sum _{j=0}^{k-1} \left( v_{i,j}(s)-4k\delta _{i,j}\right) ^2 \end{aligned}$$

where $v_{i,j}$ denotes the element at row *i* and column *j* of the matrix *V* that consists of products of spin/binary variables $s_i$ given by Eq. (5) and $\delta _{i,j}$ is the Kronecker delta function. The orthogonality requirement of *W* will be satisfied when $E_k(s)=0$, which is the lowest value of the energy function of Eq. (6). For $k=3$ and a particular value of Boolean reduction factor $\delta$ (note that it was written as $\delta _{ij}$ in^17^), by expanding this equation and then following the construction diagram in Eq. (4), we will arrive to the following 2-body Hamiltonian

7 $$\begin{aligned}{\hat{H}}_2({\hat{\sigma }}^z)&= 13,728{\hat{\sigma }}_0^z + 13,728{\hat{\sigma }}_1^z\\ & \quad+ \cdots + 13,488{\hat{\sigma }}_0^z{\hat{\sigma }}_1^z + \cdots +192{\hat{\sigma }}_{10}^z{\hat{\sigma }}_{11}^z + 162,720 \end{aligned}$$

which can be encoded into a quantum annealing processor.

In the experiment, we extract the Ising coefficients $\{J_{ij}, h_i\}$ then submit them to the D-Wave. We observe that the magnitude of the coefficients in the Hamiltonian’s terms are quite large, however they will be normalized by the D-Wave system. Additionally, the constant term, such as 162, 720 in ${\hat{H}}_2({\hat{\sigma }}^z)$ of Eq. (7), will also be removed. Consequently, instead of zero, the minimum of the energy will be a negative value. We have set the number of reads to 10,000 and obtain some solutions at minimum energy values. For $k=3$, which corresponds to H-matrix of order 12, the required number of logical qubits was 8 which translates into 36 physical qubits. We obtained the minimum energy at $-45.988$. The experimental results are displayed in Fig. 3a, where the bottom part shows the found H-matrix *H* on the left side and its indicator matrix $D\equiv H^TH$ on the right side, whereas the top parts show energy distribution of the solutions. Higher orders matrices, up to order 36 that needs 49 physical qubits to implement, have also been found successfully using the D-Wave. They are listed in the Supplementary Information section.

### Baumert–Hall based quantum computing method

The Baumert–Hall method works in a similar manner as the Williamson’s by first finding the *A*, *B*, *C*, *D* block matrices, except that the construction of the H-matrix is given by a $12\times 12$ structure of block matrix^13,34^, which yields a $12k\times 12k$ matrix for particular values of positive integers *k*.

Experiments on finding Baumert–Hall matrices using D-Wave quantum processor indicates that the capability of the method is limited by the available number of qubits, the number of couplers, and the capability of the embedding tool^35^. We have successfully found a few of Hadamard matrices up to order 108 using this method. For the 108-order case; which corresponds to $k=9$, by following the construction diagram with particular value of the Boolean reduction factor $\delta$, we will obtain a 2-body Hamiltonian given by,

8 $$\begin{aligned} {\hat{H}}_2({\hat{\sigma }}^z)&= 10,555,200{\hat{\sigma }}^z_{0} + \cdots +2,636,352{\hat{\sigma }}^z_{0}{\hat{\sigma }}^z_{1} \nonumber \\&\quad +\cdots + 1,728{\hat{\sigma }}^z_{54}{\hat{\sigma }}^z_{59} + 316,483,200 \end{aligned}$$

After extracting the Ising parameters and submitting to the D-Wave, we obtain the solutions containing correct values of $s_i$ for building the H-matrices. Figure 3b shows a 108 order H-matrix, which was found by the Baumert–Hall based method and its corresponding energy statistics as output of the quantum computer. Other Baumert–Hall matrices found by this method, i.e. 36, 60 and 84, are listed in the Supplementary Information section.

### The Turyn based quantum computing Method

In this method, first we have to find a set of 4-sequences $\{X,Y,Z,W\}$ that has particular properties, then use them to construct a H-matrix based on Goethals-Seidel method^14,16^. We derive the energy function from the requirement of a valid TT-sequences given by,

9 $$\begin{aligned} N_X(r) +N_Y(r) +2N_Z(r) +2N_W(r)=0; r\ge 1 \end{aligned}$$

where $N_X(r),N_Y(r),N_Z(r),N_W(r)$ are non-periodic auto-correlation functions of the sequences $\{X,Y,Z,W\}$ calculated at lag-*r*, respectively. Since the value given by the left-hand side of Eq. (9) can be negative, whereas the annealing is performed to achieve a minimum value, we modify it into a non-negative energy function which are squared sum of the auto-correlation function at each lag $r\ge 1$ as follows,

10 $$\begin{aligned} E_k\equiv \sum _{r\ge 1} \left( N_X(r)+N_Y(r)+2N_Z(r)+2N_W(r)\right) ^2 \end{aligned}$$

We have inserted a *k* subscript to indicate that the energy may includes *k*-body interaction terms. The searching problem becomes finding a TT-sequence that satisfy this condition. We will represent the elements of $\{X,Y,Z,W\}$ as spin variables $s_i$ as before. As an example we will calculate the Hamiltonian for $k=4$. By considering normalized sequence for efficiently use variables^16^, we obtain the following expressions for *TT*(4)

11 $$\begin{aligned} X&= \left( 1,1,1,-1\right) ^T \nonumber \\ Y&= \left( 1,s_0,-s_0,-1\right) ^T \nonumber \\ Z&= \left( 1,s_1,s_2,1\right) ^T \nonumber \\ W&= \left( 1,s_3,s_4\right) ^T \end{aligned}$$

Then, the energy in Eq. (10) which after following construction diagram given by Eq. (4) with a particular value of Boolean reduction factor $\delta$, yields the following 2-body Hamiltonian

12 $$\begin{aligned} \begin{aligned} {\hat{H}}_2({\hat{\sigma }}^z) = 912{\hat{\sigma }}^z_{0} + 1376{\hat{\sigma }}^z_{1} + \cdots + 8{\hat{\sigma }}^z_{7}{\hat{\sigma }}^z_{10} + 8,448 \end{aligned} \end{aligned}$$

In the experiment, we encode the Hamiltonian to the D-Wave system. We have successfully found the lowest order H-matrix by the Turyn-based method shown in Fig. 3c. By setting $k=6$, we also found H-matrix of order 68 listed in the Supplementary Information section.

### Balancing the quantum and classical resources: extension of the Turyn based quantum computing method

Finding H-matrix by the Turyn’s method can be achieved by checking all possible binary vector that satisfy the TT-sequences $\{X,Y,Z,W\}$ requirements. Exhaustive enumeration of all $(n, n, n, n-1)$ TT-sequence needs $2^{4n-1}$ steps, which is an exponentially increasing task. For finding higher order H-matrices, we can explore the properties of the TT-sequence to reduce the number of binary sequence to enumerate^16,36^. In this method, instead of finding all $\{X,Y,Z,W\}$ at once, it will be more computationally realistic to start with filling some part of them, then subsequently imposing conditions and properties of the TT-sequence to limit the number of the sequences to check.

**Figure 4:**
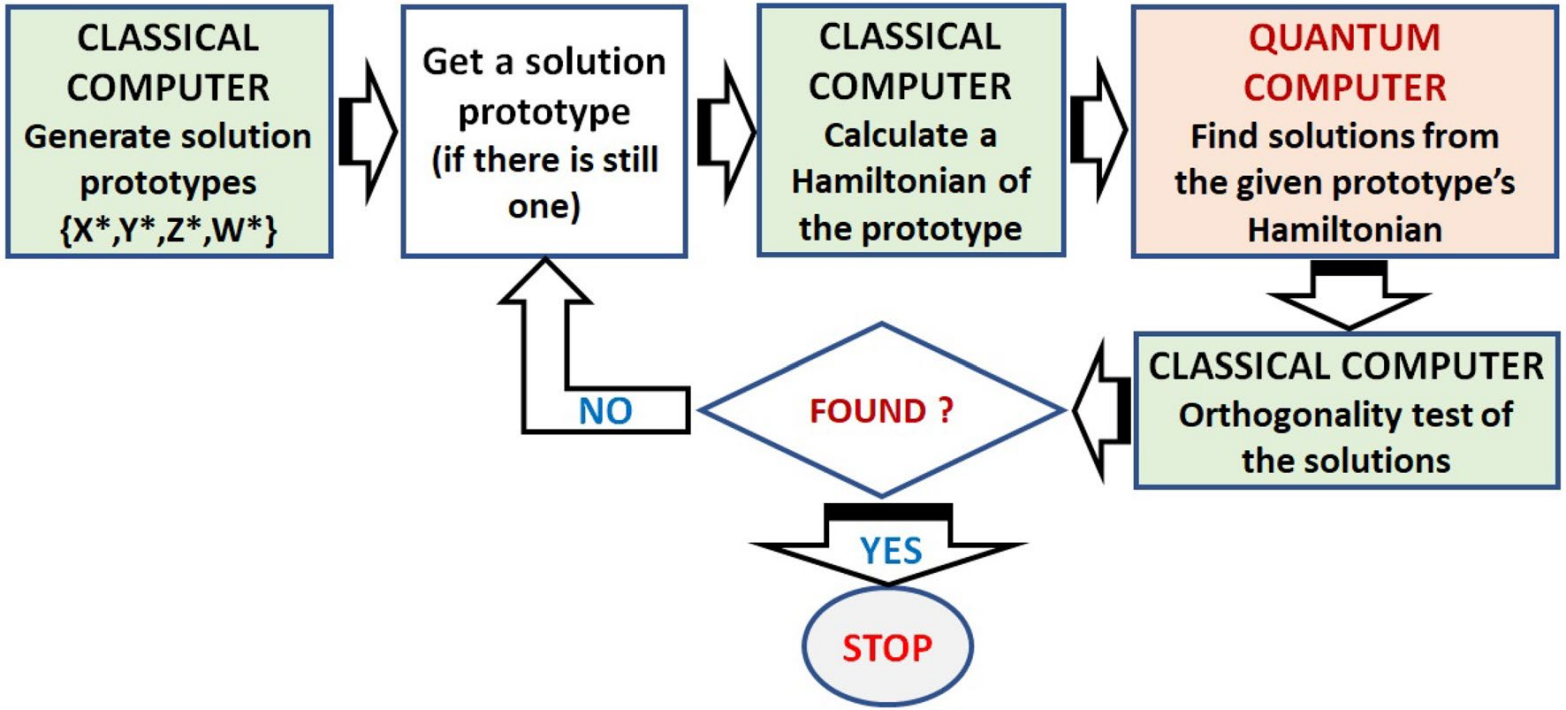
Block diagram of classical and quantum processing in extended Turyn-based quantum computing method. The classical processing includes generation of the $\{X^*,Y^*,Z^*,W^*\}$ solution prototypes, construction of the Hamiltonian, and orthogonality test of the solutions. The quantum computing is solely employed to find the solution (ground state) of the problem defined in the Hamiltonian. In this scheme, the classical and quantum processing will be terminated either after a valid solution is found or we run out of the solution prototypes.

Partially filled sequences $\{X^*, Y^*, Z^*, W^*\}$ with *m*-elements on the left part and another *m*-elements on the right one, are given as follows

$$\begin{aligned} X^*&= (x_0, x_1, ..., x_{m-1},*,*,\ldots ,*,*, x_{n-m},\ldots ,x_{n-1})^T \nonumber \\ Y^*&= (y_0, y_1, ..., y_{m-1},*,*,\ldots ,*,*, y_{n-m},\ldots , y_{n-1})^T \nonumber \\ Z^*&= (z_0, z_1, ..., z_{m-1},*,*,\ldots ,*,*, z_{n-m},\ldots , z_{n-1})^T \nonumber \\ W^*&= (w_0, w_1, ..., w_{m-1},*,*,\ldots ,*,*, w_{n-m},\ldots , w_{n-2})^T \end{aligned}$$

The requirement of non-periodic auto-correlation sum for these sequences is now become

13 $$\begin{aligned} N_{X^*}(r) + N_{Y^*}(r) + 2N_{Z^*}(r) + 2N_{W^*}(r)=0; \ r\ge (n-m) \end{aligned}$$

We will refer all $\{X^*, Y^*, Z^*, W^*\}$ sequences satisfying condition given by Eq. (13) as *solution prototypes*. Then the energy function becomes

14 $$\begin{aligned} E_k\equiv \sum _{r\ge 1} \left( N_{X^*}(r)+N_{Y^*}(r)+2N_{Z^*}(r)+2N_{W^*}(r)\right) ^2 \end{aligned}$$

Figure 4 shows a block diagram of extended Turyn-based quantum computing method, involving both of classical and quantum computing parts. The generation of $\{X^*,Y^*,Z^*,W^*\}$ solution prototypes and their corresponding Hamiltonians are conducted in a classical computer. They are fetch one-by-one and processed by a quantum computer which deliver solutions. In the next step, the classical computer checks the orthogonality of the matrices. Notes that the simplest way to check the orthogonality of an $M \times M$ matrix *H* is by multiplication $H^TH$ which consisting of *M* times multiplications for each of all $M^2$ entries in the product followed by checking them whether the off diagonal are zeros and the diagonal entries are equal to $M^2$. Therefore, the orthogonality test can be done in $O(M^3)$.

Although increasing the value of *m* in Eq. (13) will reduce the number of sequence to check in the following steps, it also increases the number of the solution prototypes itself. There are about 2 millions prototypes for $2m=12$, which will increase into about 23 millions for $2m=14$^16,36,37^. It has been reported that a few TT-sequence of up to 40 can be found using classical computers, whereas higher order ones need more powerful computers which is impossible to be implemented at the moment. This is one of the main reasons that H-matrix of order 668 has not been found nor declared non-exists yet, assuming that such a matrix can be constructed by the Turyn’s method.

On the other hand, we can use the solution prototypes to reduce the number of required qubits when a quantum computer is involved in the searching process. For clarity, in the followings we illustrate this method by a simple case which is implementable on a current quantum processor. We will consider a (4, 4, 4, 3) solution prototype to find a (8, 8, 8, 7) TT-sequences by using quantum computing; therefore, it is a kind of finding higher order sequence by extending the lower one. The extended TT-sequences can be expressed by

15 $$\begin{aligned} X&= (x_0, x_1, s_0, s_1,s_2, s_3, x_2,x_3)^T \nonumber \\ Y&= (y_0, y_1, s_4, s_5,s_6, s_7, y_2,y_3)^T \nonumber \\ Z&= (z_0, z_1, s_8, s_9,s_{10}, s_{11}, z_2,z_3)^T \nonumber \\ W&= (w_0, w_1, s_{12}, s_{13},s_{14}, s_{15}, w_2)^T \end{aligned}$$

with known $x_0,\ldots ,x_3, y_0,\ldots ,y_3,\ldots ,\ldots ,w_0, w_2$ and unknown $s_0,s_1,\ldots ,s_{15}$.

To find the unknown values represented by $s_i$, we calculate the energy of the Turyn’s based method as before. Among all possible $\{X^*,Y^*,Z^*,W^*\}$ prototypes and the replacement of the unknowns with binary variables, we choose the following solution prototype as an example

16 $$\begin{aligned} X&= (1, 1, s_0, s_1, s_2, s_3, 1, -1)^T\nonumber \\ Y&= (1, -1, s_4, s_5, s_6, s_7, 1, -1 )^T \nonumber \\ Z&= (1, -1, s_8, s_9, s_{10}, s_{11}, -1, 1)^T\nonumber \\ W&= (1, -1, s_{12}, s_{13}, s_{14}, s_{15}, 1)^T \end{aligned}$$

Note that in the real case, we might have to check all of the solution prototypes.

Further calculation by applying the construction diagram with a particular value of Boolean reduction factor $\delta$ yields the following 2-body Hamiltonian,

17 $$\begin{aligned} {\hat{H}}_2({\hat{\sigma }}^z)&=197,860{\hat{\sigma }}^z_0 + \cdots + 16,484{\hat{\sigma }}^z_0{\hat{\sigma }}^z_1+ \cdots \nonumber \\&\quad +64{\hat{\sigma }}^z_{102}{\hat{\sigma }}^z_{107} + 4,551,232 \end{aligned}$$

We then encode the Hamiltonian into the D-Wave. Calculation shows that we need 108 physical qubits to implement, but embedding into the Chimera graph with the D-Wave provided embedding tools indicates that more qubits are required, which in this case is 860. After quantum annealing, we get among others, the following solution

18 $$\begin{aligned} X&= (1, 1,-1, 1,-1, 1, 1,-1)^T \nonumber \\ Y&= (1,-1, 1, 1, 1, 1, 1,-1^T \nonumber \\ Z&= (1,-1,-1, 1, 1, 1,-1,1^T \nonumber \\ W&= (1,-1,-1,-1,-1,-1,1)^T \end{aligned}$$

Among 10,000 of obtained results, we identified two correct solutions. One of the solution that has been constructed to a H-matrix; its corresponding indicator matrix, and solution statistics are displayed in Fig. 3d. This TT(8)-sequences yields a 92-order Hadamard matrix, which in 1961 was also found by JPL researchers in a search using IBM/7090 mainframe computer^15^.

**Table 1:** Resource required in the proposed quantum computing methods.

Method	No.	*k*	Order	Number of qubits			E/P ratio	Number of correct solution in 10,000
				Logical	Physical	Embedded		
Williamson/Baumert–Hall	1	3	12 / 36*	8	36	51	1.4	322
	2	5	20/60*	12	78	207	2.7	493
	3	7	28/84*	16	316	688	5.1	282
	4	9	36/108*	20	210	1492	7.1	18
	5	11	44/132*	24	300	NA	NA	NA
Turyn	1	4	44	5	11	25	2.3	17
	2	6	68	13	36	397	11.0	35
	3	8	92	31	199	NA	NA	NA
Extended Turyn	1	4 $\rightarrow$ 8	68 $\rightarrow$ 92	16	108	860	8	2

The Willamson and Baumert–Hall based method: to find an *M* order H-matrix, the required logical qubits grows by *O*(*M*) and the physical qubits by $O(M^2)$. Embedding the connections implied by the Hamiltonian on existing Chimera graph further increases the required qubits, which ultimately limit the capability of the method. Decreasing percentage of correct solutions, knowing that only 10,000 reads in a single run is allowed, indicates that repeated experiments will be needed to find higher order matrices. The Turyn-based quantum computing method: although the number of required physical qubits also grows by $O(M^2)$, the jump among the order is high so that the next one after order 68, i.e. 92 and beyond, cannot successfully be embedded in *DW-2000Q*. We also cannot conclude the success rate for given 10,000 number of reads due to lack of data, although we may suspect that it will also decrease as in the Williamsons and Baumert–Hall adopted methods. Extended Turyn based method: the number of logical qubits is determined by the number of additional *k* in the extension $\Delta k$, not by the final qubits. We show the result of extending order $k=4$ into $k=8$ and only one of successful solution prototypes in the table.

*Order for Williamson / Baumert–Hall method.

## Discussions

Difficulties in finding a H-matrix by classical computing methods, due to the exponential grows of the complexity, can be overcome by quantum-computing based search such as by directly represents each elements of the matrix into binary variables, which is then translated into qubits^17^. However, the availability of quantum computing resource limits the implementation to only finding low order H-matrices. We have shown in the previous section that classical H-matrix searching methods can be adopted to efficiently use available quantum computing resource to solve larger problems, i.e., finding higher order H-matrices than previously achieved by the direct method^17^.

The data displayed in the top part of Table 1 shows required resource and results in the Williamson and Baumert–Hall based methods for each order of the H-matrix. Since both of them share the same *A*, *B*, *C*, *D* block matrices, we put them side-by-side on the table. We observe in the table that the number of required *logical qubits* grows linearly by *O*(*M*) with respect to the order of the searched matrix, whereas the number of *physical qubits* grows quadratically as $O(M^2)$, which is caused by the ancillary qubits required to translate *k*-body into 2-body Hamiltonians. In the implementation, the physical qubits and their connections should be mapped to the topology of qubits’s connections in the quantum annealing processor; which is the Chimera graph in the *DW-2000Q*. We have used (default) embedding tool provided by D-Wave^35^ and the number of embedding qubits displayed in the table are taken from the output of the software. This mapping process, which is also called *minor embedding*, further increases the number of required qubits. In the following discussions, the number of required qubits after the embedding process will be labeled as the *embedding qubits*.

**Figure 5:**
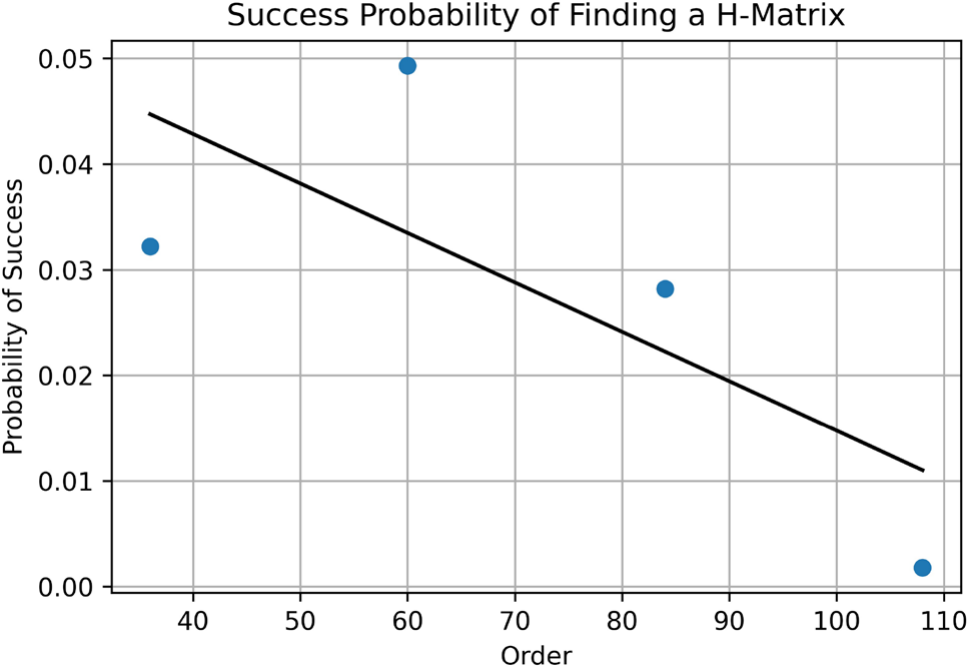
The plot of success probability against the matrix order in finding Hadamard matrices by using the proposed Baumert–Hall based quantum algorithm.

The Williamson and Baumert–Hall adopted methods can be implemented to all of matrix order as long as the embedding process is successful, which is up to 36 for the Williamson and up to 108 for the Baumert–Hall. We observe from the output of embedding tool that the highest order needs 1, 492 qubits to implement, which is more than 6 times of the required physical qubits. Observing that the trends of the embedding-to-physical qubits ratio; denoted by E/P ratio in the table, increases with the H-matrix order, by taking a moderate estimate of 7 times, the 300 physical qubits for the order of 132 matrix (in the Baumert–Hall based method) requires 2, 100 qubits to be implemented; which is more than currently available qubits in the DW-2000Q. We also observe from the experiment results, especially those displayed in the last column of Table 1, that the number of correct solutions among 10,000 number of reads tends to decrease with the increasing order of the matrix; i.e., it is about $4\%$ at the beginning then decreased to about $0.2 \%$ at order 108 for the Baumert–Hall. A possible explanation to this phenomena is that when the order of the matrix is increased, the magnitude of the coefficients in the Hamiltonian are also increased so that the difference between the largest to the smallest value becomes very large. Since they are normalized when fed into the D-Wave, with limited resolution to 1/256, the D-wave will set lower coefficients to zero. Accordingly, some of the terms will be discarded and the solutions become degenerate. It makes the percentage of the correct true solutions are reduced, as shown in the last column of the table. Since the number of reads in one run is limited by the D-Wave system to 10,000, several repeated runs on the quantum processor should be done to find higher order H-matrices. Figure 5 plots the probability of success against the order of Baumert–Hall matrices; it shows that in general higher order matrices are more difficult than the lower ones to find by the method. This also means that, for finding higher order H-matrices; assuming that the processor has a number of sufficient qubits to implement, what we have to do is by repeating the experiments many times.

**Figure 6:**
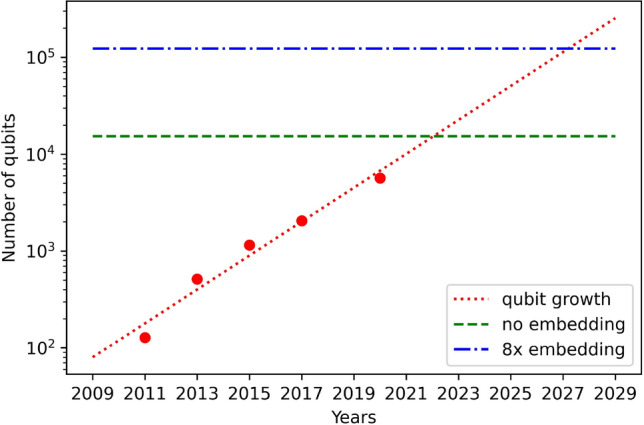
Progress of required and available qubits for solving a H-SEARCH problem. The red-dotted curve is a regression line based on actual number of qubits (shown as red circles) produced by D-Wave^38–42^. The linear curve in semi-logarithmic plot indicates that the number of qubits is doubled every two years. The blue (dash-dot) line at the top shows required number of qubits in finding 668 order H-matrix for each of a given $\{X^*,Y^*,Z^*,W^*\}$ by assuming an embedding factor of 8, whereas the green (dashed) curve is the required number of qubits with no embedding factor, meaning an ideal complete graph connections among the qubits are available.

Middle part of Table 1 shows required number of qubits and performance of the Turyn based quantum computing method. An H-matrix of order 44 and 68 have successfully been found, but higher order matrices have not. We observe that the E/P ratio grows faster than the similar case in the Williamson and Baumert–Hall based methods; i.e, it is now about 11 times at the order of 68. Assuming this factor stay the same, higher order matrices of 92, which needs 199 physical qubits, might require about 2, 189 embedding qubits. This is more than the currently available number of qubits in the DW-2000Q quantum processor, and therefore the search of order 92 H-matrix did not successful. We have proposed a solution for the limitation of quantum computer resource by the extended Turyn based method described previously.

Bottom part of Table 1 shows the required resource and performance of extended Turyn based method. For extending $k=4$ into $k=8$, we need 108 physical qubits; which is then increased into 860 embedding qubits. An important feature of this method is that, as long as the number of additional/extension $\Delta k=4$ is kept the same, the required qubits to solve extended problem will also stay the same, regardless the targeted order. However, this advantage should be paid by increasing number of solution prototypes, implying that more classical computing resources is needed and the frequency usage of the quantum processor will be increased. We expect to have an optimal point where the combination of the classical and quantum resources delivers the best solution and achieves highest order of the searched H-matrix.

At present, some of H-matrices of order under 1000; such as 668, 716, and 892, have not been found by any methods yet due to huge computational resource required to perform the computation by existing classical methods. When using the Turyn-based quantum computing method, even after extension, H-SEARCH for such orders still cannot be implemented. As an illustration, with a 12 pre-filled $\{X^*, Y^*, Z^*, W^*\}$, the required logical qubits for the 668 case will be $4\cdot (56-12)= 176$ which becomes $176\cdot 175/2 = 15,400$ physical qubits. Assuming the similar embedding performance as before at a factor of 8, the required number of qubits is 123, 200 which is beyond the capability of current quantum annealing processors.

Figure 6 shows the progress of available qubits in D-Wave quantum annealers [? ] and the decrease number of required qubits to implement H-matrix search of order 668 by solving the $\{X^*, Y^*, Z^*, W^*\}$ problem. The points in the graph shows actual number of qubits achieved in every year since 2011. We can see that the number of qubits doubled every two years; therefore, by using regression we get a linear line in a semi-logarithmic plot as shown by a dotted red curve. The middle dashed green horizontal line indicates the number of required qubits when no additional embedding qubits are required, which means that an ideal complete graph connection among the qubits is available. The top blue dashed dotted line indicates the number of required qubits with embedding factor of 8. Assuming that the connections among qubits are also improved substantially every year, we can expect the H-SEARCH of order 668 can be implemented between the year 2022 to 2029. Additionally, recent achievement of 64 qubits volume^43^ and the 1000 qubits milestone^44^ of QGM processor, the H-SEARCH implementation through QAOA is also very promising to explore.

## Methods

### Derivation of the Williamson based method

The Sylvester construction method builds a larger H-matrix $H_{2^n}$ from smaller ones $H_{2^{n-1}}$ by iteratively applying the following tensor product,

$$\begin{aligned} H_{2^n}=H_2 \otimes H_{2^{n-1}} = \begin{pmatrix} H_{2^{n-1}} &{} H_{2^{n-1}} \\ H_{2^{n-1}} &{} -H_{2^{n-1}} \end{pmatrix} \end{aligned}$$

where $$H_2=\begin{pmatrix} + &{} + \\ + &{} - \end{pmatrix}$$, i.e., it is a kind of plugging-in smaller H-matrices into a particular structure to obtain a larger H-matrix. Similarly, the Williamson method also builds a higher-order matrix from smaller ones, except that the smaller matrices are not necessarily orthogonal. In general, we can express the Williamson type H-matrices *W* by^4,12,34^

19 $$\begin{aligned} W= \begin{pmatrix} A &{} B &{} C &{} D\\ -B &{} A &{} -D &{} C\\ -C &{} D &{} A &{} -B\\ -D &{} -C &{} B &{} A \end{pmatrix} \end{aligned}$$

where *A*, *B*, *C*, *D* are block matrices, whose any pair of them are commutative, i.e., $[A,B]=[A,C]=[A,D]=[B,C]=[B,D]=[C,D]=0$, with $[A,B]=A^TB-B^TA, \ldots ,$ etc expressed the commutativity of a pair of matrices $A,B, \ldots$ etc. The orthogonality property of *W* needs the following requirement to be satisfied,

20 $$\begin{aligned} A^TA + B^TB + C^TC + D^TD = 4k I_k \end{aligned}$$

where $I_k$ is a $k\times k$ identity matrix. We will use the properties of the Williamson matrix; especially the one given by Eq. (20), to formulate the Hamiltonian of Williamson-based quantum computing method. To further reduce the number of variables, we choose *A*, *B*, *C*, *D* sub-matrices which are symmetric and circular.

For an illustration, consider $k=3$ which yields a $4k=12$-order H-matrix. The matrices can be expressed in terms of binary variables $s_i \in \{-1,+1\}$ by

21 $$\begin{aligned} A= & {} \begin{pmatrix} s_0 &{} s_1 &{} s_1\\ s_1 &{} s_0 &{} s_1\\ s_1 &{} s_1 &{} s_0 \end{pmatrix} , B= \begin{pmatrix} s_2 &{} s_3 &{} s_3\\ s_3 &{} s_2 &{} s_3\\ s_3 &{} s_3 &{} s_2 \end{pmatrix}, \nonumber \\ C= & {} \begin{pmatrix} s_4 &{} s_5 &{} s_5\\ s_5 &{} s_4 &{} s_5\\ s_5 &{} s_5 &{} s_4 \end{pmatrix}, D= \begin{pmatrix} s_6 &{} s_7 &{} s_7\\ s_7 &{} s_6 &{} s_7\\ s_7 &{} s_7 &{} s_6 \end{pmatrix}. \end{aligned}$$

Then, the requirement for Williamson matrix given by Eq. (20) for $k=3$ becomes

22 $$\begin{aligned} A^TA + B^TB + C^TC + D^TD= \begin{pmatrix} 12 &{} v &{} v \\ v &{} 12 &{} v \\ v &{} v &{} 12 \end{pmatrix} =12I_3 \end{aligned}$$

where $v=4+2\left( s_0s_1+s_2s_3+s_4s_5+s_6s_7\right)$. Suppose that $V \equiv [v_{i,j}]=A^TA+B^TB+C^TC+D^TD$. Naturally, we can define an *s*-dependent *k*-body energy function by

23 $$\begin{aligned} E_k(s)=\sum _{i=0}^2 \sum _{j=0}^2 \left( v_{i,j}(s)-12\delta _{i,j}\right) ^2 \end{aligned}$$

where $\delta _{i,j}$ is the Kronecker delta function. The orthogonality requirement of *W* will be satisfied when $E_k(s)=0$, which is the lowest value of the energy function in Eq. (23). In the $k=3$ case, the energy function $E_k(s)$ can be expanded into

24 $$\begin{aligned} E_k(s)=6\left( 4+2(s_0s_1+s_2s_3+s_4s_5+s_6s_7) \right) ^2 \end{aligned}$$

For implementing an energy function to a QAM processor; such as in the D-Wave, the *k*-body energy function $E_k(s)$ should be transformed into a 2-body energy function $E_2(s)$ using the steps given by the construction diagram in Eq. (4). In the process, we should choose a Boolean reduction factor $\delta$ to transform the *k*-body into 2-body function, that should be larger than the maximum value $E_{max}$ of the energy function^45^. By taking $E_{max}=26,976$, which is the maximum value of $E_k(s)$ by assuming all of $s_i=+1$, then setting $\delta =2E_{max}$, we obtain the following result

25 $$\begin{aligned} E_2(s)&= 13,728s_0 + 13,728s_1 + \cdots + 13,488s_0s_1 \nonumber \\&\quad + \cdots + 192s_{10}s_{11} + 162,720 \end{aligned}$$

This 2-body energy function gives the potential Hamiltonian ${\hat{H}}_{pot}({\hat{\sigma }}) \equiv {\hat{H}}_2({\hat{\sigma }}^z)$ as follows,

26 $$\begin{aligned} {\hat{H}}_2({\hat{\sigma }}^z)&= 13,728{\hat{\sigma }}_0^z + 13,728{\hat{\sigma }}_1^z \nonumber \\&\quad + \cdots+13,488{\hat{\sigma }}_0^z{\hat{\sigma }}_1^z + \cdots +192{\hat{\sigma }}_{10}^z{\hat{\sigma }}_{11}^z + 162,720 \end{aligned}$$

Complete expressions for the equations can be found in Supplementary Information section.

### Derivation of the Baumert–Hall based method

In principle, the Baumert–Hall quantum computing method works in a similar manner as the Williamson’s by first finding the *A*, *B*, *C*, *D* block matrices, except that the construction of the H-matrix is given by the following $12\times 12$ structure of block matrix^13,34^:

27 $$\begin{aligned} H= \left( \begin{array}{rrrr rrrr rrrr} A &{} A &{} A &{} B &{} -B &{} C &{} -C &{} -D &{} B &{} C &{} -D &{} -D\\ A &{} -A &{} B &{} -A &{} -B &{} -D &{} D &{} -C &{} -B &{} -D &{} -C &{} -C\\ A &{} -B &{} -A &{} A &{} -D &{} D &{} -B &{} B &{} -C &{} -D &{} C &{} -C\\ B &{} A &{} -A &{} -A &{} D &{} D &{} D &{} C &{} C &{} -B &{} -B &{} -C\\ B &{} -D &{} D &{} D &{} A &{} A &{} A &{} C &{} -C &{} B &{} -C &{} B\\ B &{} C &{} -D &{} D &{} A &{} -A &{} C &{} -A &{} -D &{} C &{} B &{} -B\\ D &{} -C &{} B &{} -B &{} A &{} -C &{} -A &{} A &{} B &{} C &{} D &{} -D\\ -C &{} -D &{} -C &{} -D &{} C &{} A &{} -A &{} -A &{} -D &{} B &{} -B &{} -B\\ D &{} -C &{} -B &{} -B &{} -B &{} C &{} C &{} -D &{} A &{} A &{} A &{} D\\ -D &{} -B &{} C &{} C &{} C &{} B &{} B &{} -D &{} A &{} -A &{} D &{} -A\\ C &{} -B &{} -C &{} C &{} D &{} -B &{} -D &{} -B &{} A &{} -D &{} -A &{} A\\ -C &{} -D &{} -D &{} C &{} -C &{} -B &{} B &{} B &{} D &{} A &{} -A &{} -A \end{array}\right) \end{aligned}$$

Considering the usage efficiency of the variables, *A*, *B*, *C*, *D* are also chosen to be symmetric circulant block matrices identical to the Williamsons’s method described in the previous section. For a $k\times k$ size of the block matrices, Eq. (27) yields a $12k \times 12k$ dimension of the H-matrix. The formulation of the energy function also follows the Williamsons method described previously.

Experiments on finding Baumert–Hall matrices using D-Wave quantum processor indicates that the capability of the method is limited by the available number of qubits and the capability of the embedding tool^35^. We have successfully find the Hadamard matrix up to order 108 using this method. For the 108-order case, initial energy function $E_k(s)$ to find this matrix is given by the following

28 $$\begin{aligned} E_k(s)&= 432s_0s_2 + \cdots + 720s_{18}s_{19} \nonumber \\&\quad +\cdots+ 432s_{16}s_{17}s_{18}s_{19} + 5760 \end{aligned}$$

whose corresponding *k*-body Hamiltonian is given by

29 $$\begin{aligned} {\hat{H}}_k({\hat{\sigma }}^z)&= 432{\hat{\sigma }}^z_0{\hat{\sigma }}^z_2 +\cdots + 720{\hat{\sigma }}^z_{18}{\hat{\sigma }}^z_{19} \nonumber \\&\quad + \cdots+ 432{\hat{\sigma }}^z_{16}{\hat{\sigma }}^z_{17}{\hat{\sigma }}^z_{18}{\hat{\sigma }}^z_{19} + 5760 \end{aligned}$$

Then the 2-body Hamiltonian realized on the quantum annealing processor will be given by,

30 $$\begin{aligned} {\hat{H}}_2({\hat{\sigma }}^z)&= 10,555,200{\hat{\sigma }}^z_{0} + \cdots +2,636,352{\hat{\sigma }}^z_{0}{\hat{\sigma }}^z_{1} \nonumber \\&\quad +\cdots + 1,728{\hat{\sigma }}^z_{54}{\hat{\sigma }}^z_{59} + 316,483,200 \end{aligned}$$

Complete expressions for the equations can be found in Supplementary Information section.

### Derivation of the Turyn based method

In this method, first we find a set of 4-sequences $\{X,Y,Z,W\}$ that has particular properties, then use them to construct a H-matrix based on Goethals-Seidel method^14,16^. We translate the requirements into energy functions which then programmed into a quantum processor. In essence, the workflows of the Turyn based method are as follows

1. Find an $(n, n, n, n-1)$ Turyn-Type (TT) sequence $\{X,Y,Z,W\}$.
2. Construct base sequences $\{A,B,C,D\}$
3. Construct T-sequences $$\{ T_1,T_2,T_3,T_4\}$$
4. Construct seed sequences $\{A_1,A_2,A_3,A_4\}$
5. Construct block symmetric circular matrices $\{X_{A1},X_{A2},X_{A3},X_{A4}\}$
6. Construct Hadamard matrix, which is given by
  31 $$\begin{aligned} H= \begin{pmatrix} X_{A1} &{} X_{A2}R &{} X_{A3}R &{} X_{A4}R \\ -X_{A2}R &{} X_{A1} &{} X_{A4}^TR &{} -X_{A3}^TR\\ -X_{A3}R &{} -X_{A4}^TR &{} X_{A1} &{} X_{A2}^TR\\ -X_{A4}R &{} X_{A3}^TR &{} -X_{A2}^TR &{} X_{A1} \end{pmatrix} \end{aligned}$$
  where *R* is a back-diagonal identity matrix of size $k \times k$ as follows
  32 $$\begin{aligned} R= \begin{pmatrix} 0 &{} 0 &{} \cdots &{} 0 &{} 1 \\ 0 &{} 0 &{} \cdots &{} 1 &{} 0 \\ \cdots &{} \cdots &{}\cdots &{} \cdots &{} \cdots \\ 0 &{} 1 &{} \cdots &{} 0 &{} 0 \\ 1 &{} 0 &{} \cdots &{} 0 &{} 0 \\ \end{pmatrix} \end{aligned}$$

We derive the energy function from the requirement of a valid TT-sequences given by,

33 $$\begin{aligned} N_X(r) +N_Y(r) +2N_Z(r) +2N_W(r)=0; r\ge 1 \end{aligned}$$

where $N_X(r),N_Y(r),N_Z(r),N_W(r)$ are non-periodic auto-correlation functions of the sequences $\{X,Y,Z,W\}$ calculated at lag-*r*, respectively. The non-periodic auto-correlation function of a sequence $V=[v_0,v_1, \ldots , v_{n-1}]^T$ is given by,

34 $$\begin{aligned} N_V(r)=\sum _{t=0}^{n-1-r} v_i v_{i+r} \end{aligned}$$

for $r = 0, 1,\ldots , n-1$ and $N_V(r)=0$ for $r\ge n$. Since the value given by the left-hand side of Eq. (33) can be negative, whereas the annealing is performed to achieve a minimum value, we adopt a non-negative energy function which are sum of squared value of the auto-correlation function at each lag $r\ge 1$ as follows,

35 $$\begin{aligned} E\equiv \sum _{r\ge 1} \left( N_X(r)+N_Y(r)+2N_Z(r)+2N_W(r)\right) ^2 \end{aligned}$$

To efficiently use available qubits in the quantum processor, it is important to reduce the number of variables encoded to the qubits as few as possible. We can achieve this by further employing the property of the TT-sequences. In this case, we can normalize the TT-sequences^16^ to obtain $X^T=(x_0, x_1, \ldots x_{n-1})$, $Y^T=(y_0, y_1, \ldots y_{n-1})^T$, $Z^T=(z_0, z_1, \ldots z_{n-1})$, and $W^T=(w_0, w_1, \ldots w_{n-1})$, which have the following properties

- $$x_0=y_0=z_0=w_0=1$$
- $$x_{n-1}=y_{n-1}=-1, z_{n-1}=1$$
- $$x_1=x_{n-2}=1, y_1y_{n-2}=-1$$

For clarity, in the followings we present an example of the Hamiltonian formulation for the lowest order of $k=4$ case. The first step as described previously is to find a *TT*(4) -sequences $\{X,Y,Z,W\}$. By representing the elements of the sequences as binary (spin) variables $s_i\in \{-1,+1\}$, and applying the properties of a normalized sequence explained previously, a *TT*(4) will be as follows,

36 $$\begin{aligned} X&= \left( 1,1,1,-1\right) ^T \nonumber \\ Y&= \left( 1,s_0,-s_0,-1\right) ^T \nonumber \\ Z&= \left( 1,s_1,s_2,1\right) ^T \nonumber \\ W&= \left( 1,s_3,s_4\right) ^T \end{aligned}$$

To determine the energy function, we have to calculate non-periodic auto-correlation functions $N_X,N_Y,N_Z,N_W$ given by Eq. (34). Since $s_i^2=1$, we get the following results after simplifications

37 $$\begin{aligned} N_X&= ( 4, 1, 0, -1)^T \nonumber \\ N_Y&= ( 4, 2s_0 - 1, -2s_0, -1)^T \nonumber \\ N_Z&= \left( 4, s_1 + s_2 + s_1s_2, s_1 + s_2, 1\right) ^T \nonumber \\ N_W&= \left( 3, s_3 + s_3s_4, s_4\right) ^T \end{aligned}$$

Therefore, the energy $E \equiv E_k(s)$ in Eq. (35), whose terms may contain products of *k* variables, is now given by

38 $$\begin{aligned} E_k(s)&= 2s_1 + 2s_2 + 2s_4 + 4s_0s_3 + 4s_1s_2 \nonumber \\&\quad - 4s_0s_4 + 2s_1s_3 + 2s_1s_4 + 2s_2s_3 + 2s_2s_4 \nonumber \\&\quad + 4s_0s_1s_2+ 2s_1s_2s_3 + 4s_0s_3s_4 + 2s_1s_3s_4 \nonumber \\&\quad + 2s_2s_3s_4 + 2s_1s_2s_3s_4 + 242 \end{aligned}$$

In the following steps, as described by the construction diagram in Eq. (4), the energy function should be transformed into a 2-body interacting Ising Hamiltonian. Therefore, we have to change the *s*-dependent energy function into *q*-dependent energy function $E_k(q)$. After simplification, this transform yields the following

39 $$\begin{aligned} E_k(q)&= - 16q_0 - 40q_1 - 40q_2 - 40q_3 - 24q_4 \nonumber \\&\quad + 16q_0q_1 + 16q_0q_2 + 32q_0q_3 + 48q_1q_2+ 32q_1q_3 \nonumber \\&\quad + 24q_1q_4 + 32q_2q_3 + 24q_2q_4 + 40q_3q_4- 32q_0q_1q_2 \nonumber \\&\quad - 32q_1q_2q_3 - 32q_0q_3q_4 - 16q_1q_2q_4 - 32q_1q_3q_4 \nonumber \\&\quad - 32q_2q_3q_4 + 32q_1q_2q_3q_4 + 276 \end{aligned}$$

The conversion into 2-body energy function requires a Boolean reduction factor $\delta$ set to be larger than the maximum value of the energy function $E_{max}(k)$. Assuming it is at least an absolute sum of the $E_k(q)$ coefficients as before, we have $E_{max} = 908$. By taking twice of this maximum value, we obtain $\delta =1,816$, which transforms Eq. (39) into

40 $$\begin{aligned} E_2(q)&= - 16q_0 - 40q_1 - 40q_2 - 40q_3 - 24q_4 \nonumber \\&\quad + 5,464q_5+ 5,480q_6 + 5,496q_7 + 5,480q_8 \nonumber \\&\quad + 5,480q_9 + 5,488q_{10} + 1,816q_0q_1 + 16q_0q_2 \nonumber \\&\quad + 1,816q_0q_3 + 1,816q_1q_2+ 1,816q_1q_3-3,632q_0q_5 \nonumber \\&\quad + 24q_1q_4 + 1,816q_2q_3- 3,632q_0q_6 - 3,632q_1q_5 \nonumber \\&\quad + 24q_2q_4 - 32q_2q_5 + 1,816q_3q_4 - 3,632q_1q_7 \nonumber \\&\quad - 3,632q_1q_8 - 3,632q_2q_7- 3,632q_3q_6 - 32q_3q_7\nonumber \\&\quad - 32q_4q_6 - 3,632q_2q_9- 3,632q_3q_8 - 16q_4q_7 \nonumber \\&\quad - 3632q_3q_9 - 32q_4q_8 - 3,632q_3q_{10} - 32q_4q_9 \nonumber \\&\quad - 3,632q_4q_{10} + 32q_7q_{10} + 276 \end{aligned}$$

Transforming back Eq. (40) to the *s*-domain yields the following expression,

41 $$\begin{aligned} E_2(s)&= 912s_{0} + 1376s_{1} + 926s_{2} + 1844s_{3} + 482s_{4} \nonumber \\&\quad - 908s_{5} - 916s_{6} - 928s_{7}- 916s_{8} - 916s_{9} \nonumber \\&\quad - 936s_{10} + 454s_{0}s_{1} + 4s_{0}s_{2} + 454s_{0}s_{3} + 454s_{1}s_{2} \nonumber \\&\quad + 454s_{1}s_{3} - 908s_{0}s_{5} + 6s_{1}s_{4} + 454s_{2}s_{3} - 908s_{0}s_{6} \nonumber \\&\quad - 908s_{1}s_{5} + 6s_{2}s_{4} - 8s_{2}s_{5} + 454s_{3}s_{4} - 908s_{1}s_{7} \nonumber \\&\quad - 908s_{1}s_{8} - 908s_{2}s_{7}- 908s_{3}s_{6} - 8s_{3}s_{7} - 8s_{4}s_{6} \nonumber \\&\quad - 908s_{2}s_{9} - 908s_{3}s_{8} - 4s_{4}s_{7} - 908s_{3}s_{9} - 8s_{4}s_{8} \nonumber \\&\quad - 908s_{3}s_{10} - 8s_{4}s_{9} - 908s_{4}s_{10} + 8s_{7}s_{10} + 8,448 \end{aligned}$$

which corresponds to the following 2-body Hamiltonian,

42 $$\begin{aligned} {\hat{H}}_2({\hat{\sigma }}^z)&= 912{\hat{\sigma }}^z_{0} + 1376{\hat{\sigma }}^z_{1} + 926{\hat{\sigma }}^z_{2} + 1844{\hat{\sigma }}^z_{3} \nonumber \\&\quad + 482{\hat{\sigma }}^z_{4} - 908{\hat{\sigma }}^z_{5}- 916{\hat{\sigma }}^z_{6} - 928{\hat{\sigma }}^z_{7} -916{\hat{\sigma }}^z_{8} \nonumber \\&\quad - 916{\hat{\sigma }}^z_{9} - 936{\hat{\sigma }}^z_{10} + 454{\hat{\sigma }}^z_{0}{\hat{\sigma }}^z_{1} + 4{\hat{\sigma }}^z_{0}{\hat{\sigma }}^z_{2} \nonumber \\&\quad + 454{\hat{\sigma }}^z_{0}{\hat{\sigma }}^z_{3} + 454{\hat{\sigma }}^z_{1}{\hat{\sigma }}^z_{2} + 454{\hat{\sigma }}^z_{1}{\hat{\sigma }}^z_{3}- 908{\hat{\sigma }}^z_{0}{\hat{\sigma }}^z_{5} \nonumber \\&\quad + 6{\hat{\sigma }}^z_{1}{\hat{\sigma }}^z_{4} + 454{\hat{\sigma }}^z_{2}{\hat{\sigma }}^z_{3} - 908{\hat{\sigma }}^z_{0}{\hat{\sigma }}^z_{6} - 908{\hat{\sigma }}^z_{1}{\hat{\sigma }}^z_{5} \nonumber \\&\quad + 6{\hat{\sigma }}^z_{2}{\hat{\sigma }}^z_{4} - 8{\hat{\sigma }}^z_{2}{\hat{\sigma }}^z_{5} + 454{\hat{\sigma }}^z_{3}{\hat{\sigma }}^z_{4} - 908{\hat{\sigma }}^z_{1}{\hat{\sigma }}^z_{7} \nonumber \\&\quad - 908{\hat{\sigma }}^z_{1}{\hat{\sigma }}^z_{8} - 908{\hat{\sigma }}^z_{2}{\hat{\sigma }}^z_{7} - 908{\hat{\sigma }}^z_{3}{\hat{\sigma }}^z_{6} - 8{\hat{\sigma }}^z_{3}{\hat{\sigma }}^z_{7} \nonumber \\&\quad - 8{\hat{\sigma }}^z_{4}{\hat{\sigma }}^z_{6} - 908{\hat{\sigma }}^z_{2}{\hat{\sigma }}^z_{9} - 908{\hat{\sigma }}^z_{3}{\hat{\sigma }}^z_{8} - 4{\hat{\sigma }}^z_{4}{\hat{\sigma }}^z_{7} \nonumber \\&\quad - 908{\hat{\sigma }}^z_{3}{\hat{\sigma }}^z_{9} - 8{\hat{\sigma }}^z_{4}{\hat{\sigma }}^z_{8} - 908{\hat{\sigma }}^z_{3}{\hat{\sigma }}^z_{10} - 8{\hat{\sigma }}^z_{4}{\hat{\sigma }}^z_{9} \nonumber \\&\quad - 908{\hat{\sigma }}^z_{4}{\hat{\sigma }}^z_{10} + 8{\hat{\sigma }}^z_{7}{\hat{\sigma }}^z_{10} + 8,448 \end{aligned}$$

### Complexity analysis

This subsection describes complexity analysis on the number of required qubits, especially the reduction from $O(M^3)$ in the direct method of^17^ to $O(M^2)$ proposed in this paper. In worst case condition, a brute force method of finding an $M \times M$ H-matrix should check all possible combinations of “-1” and “+1” in the $M^2$ entries of the matrix, i.e., we should perform orthogonality test to all of $2^{M\times M}$ matrices. In the SI (Supplementary Information) of^17^, we have showed that we need $M^2$ logical qubits if the machine capable to implement *k*-body interactions for any non negative integer *k*; which in this case is up to 4-body Hamiltonian terms (Eq. (S5)). When the machine is only capable of implementing 2-body Hamiltonian terms, additional ancillary qubits are required. In the sub section *High-Order Case: The Needs of Symbolic Computing* of the SI, we have showed that it will further increase the number of required qubits into $M^2+M\times M(M-1)/2$; i.e., an increase from $O(M^2)$ to $O(M^3)$.

Further reduction of the needed qubits is achieved through the usage of proposed methods described in this paper, such as the Turyn based method. As explained in the section *Methods*, subsection *C. Derivation of the Turyn based Method*, the (Turyn) Hadamard matrix can be constructed from an $\left( n, n, n, n-1\right)$ Turyn Type/TT-sequence. For a given $\left( n, n, n, n-1\right)$ TT-sequence, we can construct a $4(4n-1)$ order Hadamard matrix; i.e, to find an $4(4n-1)$ order H matrix, we only need to find a $(4n-1)$ length sequence. Therefore, in the Turyn-based method, the required logical qubits to find the $M\times M$ Hadamard matrix is *O*(*M*). The quadratic energy function given by Eq. (35) implies that there will be up to 4-body terms in the Hamiltonian. Again, when using D-Wave that can only accommodate up-to 2-body terms, additional ancillary qubits are needed. Accordingly, the final number of the required logical qubits will be $O(M^2)$.

## Supplementary Information

Supplementary Information.

## Data Availability

All of codes and data will be provided upon direct request to the authors. Some parts of the codes can be found in https://github.com/suksmono.

## References

[1] Sylvester JJLX (1867). Thoughts on inverse orthogonal matrices, simultaneous sign successions, and tessellated pavements in two or more colours, with applications to Newton’s Rule, ornamental tile-work, and the theory of numbers. Philos. Mag..

[2] Hadamard J (1893). Resolution d’une question relative aux determinants. Bull. Des Sci. Math..

[3] Garg, V. *Wireless Communications and Networking* (Morgan-Kaufman, 2007).

[4] Horadam, K. J. *Hadamard Matrices and Their Applications* (Princeton University Press, 2007).

[5] Pless, V. & Huffman, W. C. *Handbook of Coding Theory* (North Holland, 1998).

[6] Paley REAC (1933). On orthogonal matrices. J. Math. Phys..

[7] Horadam KJ (1993). Cocyclic development of designs. J. Algebraic Comb..

[8] Horadam KJ, de Launey W (1995). Generation of cocyclic Hadamard matrices. Math. Appl..

[9] Horadam KJ (2000). An introduction to cocyclic generalised hadamard matrices. Discret. Appl. Math..

[10] Álvarez V (2020). On cocyclic hadamard matrices over goethals-seidel loops. Mathematics.

[11] Álvarez, V. *et al.* Hadamard matrices with cocyclic core. *Mathematics* **9**, 857 (2021).

[12] Williamson J (1944). Hadamard’s determinant theorem and the sum of four squares. Duke Math. J..

[13] Baumert L, Hall M (1965). A new construction for Hadamard matrices. Bull. Am. Math. Soc..

[14] Turyn RJ (1974). Hadamard matrices, baumert-hall units, four-symbol sequences, pulse compression, and surface wave encodings. J. Comb. Theory Ser. A..

[15] Baumert L, Golomb SW, Hall M (1962). Discovery of an Hadamard matrix of order 92. Bull. Am. Math. Soc..

[16] Kharaghani H, Tayfeh-Rezaie B (2005). A Hadamard matrix of order 428. J. Comb. Designs..

[17] Suksmono AB, Minato Y (2019). Finding hadamard matrices by a quantum annealing machine. Sci. Rep..

[18] Papageorgiou A, Traub JF (2013). Measures of quantum computing speedup. Phys. Rev. A..

[19] Harrow AW, Montanaro A (2017). Quantum computational supremacy. Nature.

[20] Arute F (2019). Quantum supremacy using a programmable superconducting processor. Nature.

[21] Suksmono AB (2018). Finding a hadamard matrix by simulated quantum annealing. Entropy.

[22] Kadowaki T, Nishimori H (1988). Quantum annealing in the transverse Ising model. Phys. Rev. E..

[23] Ray P, Chakrabarti BK, Chakrabarti A (1989). Sherrington–Kirkpatrick model in a transverse field: Absence of replica symmetry breaking due to quantum fluctuations. Phys. Rev. B..

[24] Farhi E, Gutmann S (1998). Quantum computation and decision tree. Phys. Rev. A..

[25] Das A, Chakrabarti BK (2008). Colloquium: Quantum annealing and analog quantum computation. RMP..

[26] Boixo S (2014). Evidence for quantum annealing with more than one hundred qubits. Nat. Phys..

[27] Zick KM, Shehab O, French M (2015). Experimental quantum annealing: Case study involving the graph isomorphism problem. Sci. Rep..

[28] Wang C, Chen H, Jonckheere E (2016). Quantum versus simulated annealing in wireless interference network optimization. Sci. Rep..

[29] Ikeda K, Nakamura Y, Humble TS (2019). Application of quantum annealing to nurse scheduling problem. Sci. Rep..

[30] Benedetti M, Realpe-Gómez J, Biswas R, Perdomo-Ortiz A (2017). Quantum-assisted learning of hardware-embedded probabilistic graphical models. Phys. Rev. X..

[31] Li R, Felice R, Rohs R, Lidar D (2018). Quantum annealing versus classical machine learning applied to a simplified computational biology problem. NPJ Quant. Inf..

[32] O’Malley D (2018). An approach to quantum-computational hydrologic inverse analysis. Sci. Rep..

[33] Farhi, E., Goldstone, J. & Gutmann, S. A quantum approximate optimization algorithm. Preprint arXiv:1411.4028 (2014).

[34] Hedayat A, Wallis WD (1978). Hadamard matrices and their applications. Ann. Stat..

[35] D-Wave System, Inc., *Developer Guide for MATLAB: User Manual.* D-Wave System Inc. (2018).

[36] Best D, Dokovic DZ, Kharaghani H, Ramp H (2012). Turyn-type sequences: Classification, enumeration, and construction. J. Comb. Des..

[37] London, S. Constructing New Turyn Type Sequences, T-Sequences and Hadamard Matrices. *PhD. Thesis, Grad. College.* University of Illinois at Chicago (2013).

[38] Johnson MW (2011). Quantum annealing with manufactured spins. Nature.

[39] Choi, C. Google and NASA Launch Quantum Computing AI Lab. *MIT Technology Review.* (2013).

[40] D-Wave Systems. D-Wave Systems Announces Multi-Year Agreement To Provide Its Technology To Google. *NASA And USRA’s Quantum Artificial Intelligence Lab.* https://www.dwavesys.com/company/newsroom/. (2015).

[41] Finley, K. Quantum Computing Is Real, and D-Wave Just Open-Sourced It. *Wired*. (2017).

[42] Timmer, J. D-Wave announces new hardware, compiler, and plans for quantum computing. *Ars Technica*. (2021).

[43] Jurcevic, P. et al. Demonstration of quantum volume 64 on a superconducting quantum computing system. Preprint arXiv:2008.08571 (2020).

[44] Cho, A. IBM promises 1000-qubit quantum computer-a milestone-by 2023. *Science.* Sept. **15**, (2020).

[45] Perdomo A, Truncik C, Tubert-Brohman I, Rose G, Aspuru-Guzik A (2008). Construction of model hamiltonians for adiabatic quantum computation and its application to finding low-energy conformations of lattice protein models. Phys. Rev. A..

