# Supplementary material for: Quantum computing formulation of some classical Hadamard matrix searching methods and its implementation on a quantum computer
Source: Sci Rep. 2022 Jan 7;12:197. doi: 10.1038/s41598-021-03586-0 (PMC8741795; doi:10.1038/s41598-021-03586-0)
Supplement: Supplementary file 1 — Supplementary Information. [file 41598_2021_3586_MOESM1_ESM.pdf]

Supplementary Information
Quantum computing formulation of some classical Hadamard matrix searching methods and its implementation on a quantum computer

ANDRIYAN BAYU SUKSMONO, Institut Teknologi Bandung, Indonesia
YUICHIRO MINATO, Bluegat Inc., Japan

Hadamard Matrices Found by Williamson Based Quantum Computing Method.

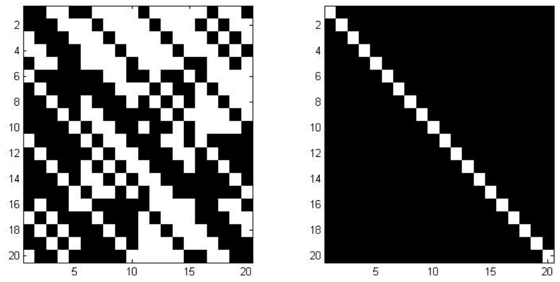

Fig. S.1. H-matrix of order 20: left part is the H-matrix (white=+1, black=−1), right part is indicator matrix (black=0, white=20).

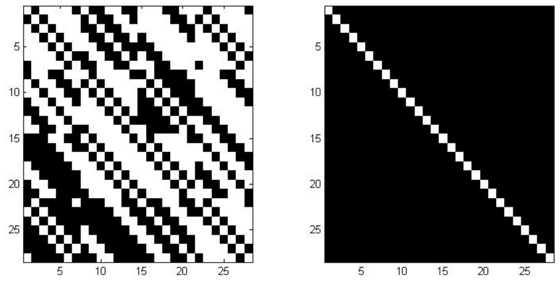

Fig. S.2. H-matrix of order 28: left part is the H-matrix (white= +1, black=-1), right part is indicator matrix (black=0, white=28).

Authors' addresses: Andriyan Bayu Suksmono, suksmono@stei.itb.ac.id, Institut Teknologi Bandung, Jl. Ganesha No.10, Bandung, Indonesia; Yuichiro Minato, Blueqat Inc., Hongo 2-40-14-3F, Bunkyo-ku, Tokyo, Japan.

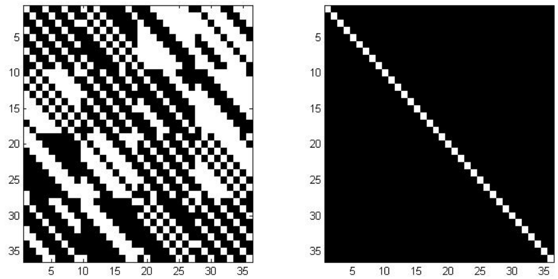

Fig. S.3. H-matrix of order 36: left part is the H-matrix (white=+1, black=-1), right part is indicator matrix (black=0, white=36).

Hadamard Matrices Found by Baumert-Hall Based Quantum Computing Method.

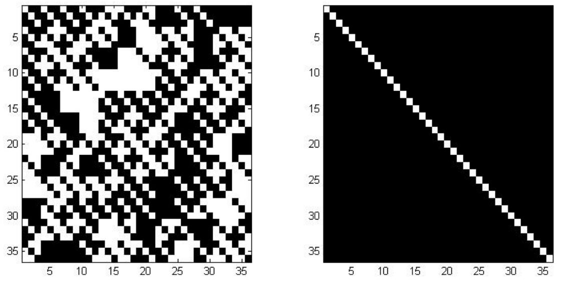

Fig. S.4. H-matrix of order 36: left part is the H-matrix (white=+1, black=−1), right part is indicator matrix (black=0, white=36).

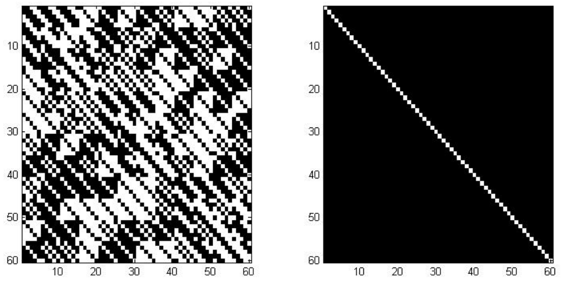

Fig. S.5. H-matrix of order 60: left part is the H-matrix (white=+1, black=−1), right part is indicator matrix (black=0, white=60).

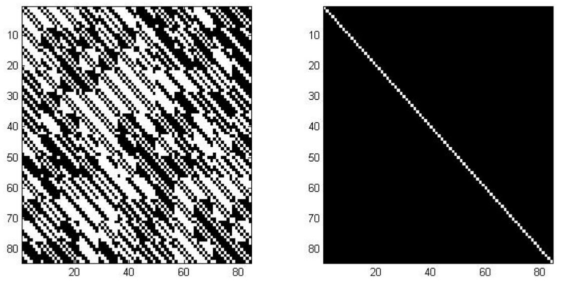

Fig. S.6. H-matrix of order 84: left part is the H-matrix (white=+1, black=−1), right part is indicator matrix (black=0, white=84).

Hadamard Matrices Found by Turyn's Based Quantum Computing Method.

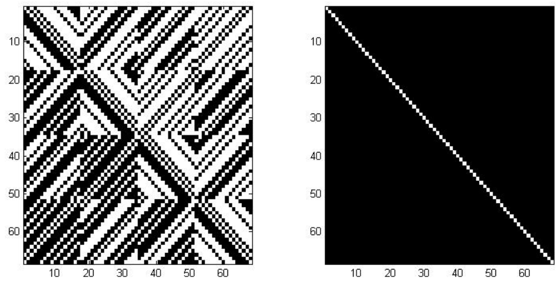

Fig. S.7. H-matrix of order 68: left part is the H-matrix (white=+1, black=−1), right part is indicator matrix (black=0, white=68).

Complete Expressions in The Williamson (and Baumert-Hall)'s Based Method

A complete expression of $E_k(s)$ for 12-order Williamson based method or 36-order Baumert-Hall based method

$E_k(s) = 96s_0s_1 + 96s_2s_3 + 96s_4s_5 + 96s_6s_7 + 48s_0s_1s_2s_3 + 48s_0s_1s_4s_5 + 48s_0s_1s_6s_7 + 48s_2s_3s_4s_5 + 48s_2s_3s_6s_7 + 48s_4s_5s_6s_7 + 192$

A complete expression of $E_k(q)$ for 12-order Williamson based method or 36-order Baumert-Hall based method

$E_k(q) = 960q_0q_1 - 480q_1 - 480q_2 - 480q_3 - 480q_4 - 480q_5 - 480q_6 - 480q_7 - 480q_0 + 192q_0q_2 + 192q_0q_3 + 192q_1q_2 + 192q_0q_4 + 192q_1q_3 + 192q_0q_5 + 192q_1q_4 + 960q_2q_3 + 192q_0q_6 + 192q_1q_5 + 192q_2q_4 + 192q_0q_7 + 192q_1q_6 + 192q_2q_5 + 192q_3q_4 + 192q_1q_7 + 192q_2q_6 + 192q_3q_5 + 192q_2q_7 + 192q_3q_6 + 960q_4q_5 + 192q_3q_7 + 192q_4q_6 + 192q_4q_7 + 192q_5q_6 + 192q_5q_7 + 960q_6q_7 - 384q_0q_1q_2 - 384q_0q_1q_3 - 384q_0q_1q_4 - 384q_0q_2q_3 - 384q_0q_1q_5 - 384q_1q_2q_3 - 384q_0q_1q_6 - 384q_0q_1q_7 - 384q_0q_4q_5 - 384q_2q_3q_4 - 384q_1q_4q_5 - 384q_2q_3q_5 - 384q_2q_3q_6 - 384q_2q_4q_5 - 384q_2q_3q_7 - 384q_3q_4q_5 - 384q_0q_6q_7 - 384q_1q_6q_7 - 384q_2q_6q_7 - 384q_4q_5q_6 - 384q_3q_6q_7 - 384q_4q_5q_7 - 384q_4q_6q_7 - 384q_5q_6q_7 + 768q_0q_1q_2q_3 + 768q_0q_1q_4q_5 + 768q_0q_1q_6q_7 + 768q_2q_3q_4q_5 + 768q_2q_3q_6q_7 + 768q_4q_5q_6q_7 + 864$

A complete expression of $E_2(q)$ for 12-order Williamson based method or 36-order Baumert-Hall based method by taking $\delta = 53,952$

$E_2(q) = 162816q_8 - 480q_1 - 480q_2 - 480q_3 - 480q_4 - 480q_5 - 480q_6 - 480q_7 - 480q_0 + 162816q_9 + 162816q_{10} + 162816q_{11} + 53952q_0q_1 + 192q_0q_2 + 192q_0q_3 + 192q_1q_2 + 192q_0q_4 + 192q_1q_3 + 192q_0q_5 + 192q_1q_4 + 53952q_2q_3 + 192q_0q_6 + 192q_1q_5 + 192q_2q_4 + 192q_0q_7 + 192q_1q_6 + 192q_2q_5 + 192q_3q_4 - 107904q_0q_8 + 192q_1q_7 + 192q_2q_6 + 192q_3q_5 - 384q_0q_9 - 107904q_1q_8 + 192q_2q_7 + 192q_3q_6 + 53952q_4q_5 - 384q_0q_{10} - 384q_1q_9 - 384q_2q_8 + 192q_3q_7 + 192q_4q_6 - 384q_0q_{11} - 384q_1q_{10} - 107904q_2q_9 - 384q_3q_8 + 192q_4q_7 + 192q_5q_6 - 384q_1q_{11} - 384q_2q_{10} - 107904q_3q_9 - 384q_4q_8 + 192q_5q_7 - 384q_2q_{11} - 384q_3q_{10} - 384q_4q_9 - 384q_5q_8 + 53952q_6q_7 - 384q_3q_{11} - 107904q_4q_{10} - 384q_5q_9 - 384q_6q_8 - 384q_4q_{11} - 107904q_5q_{10} - 384q_6q_9 - 384q_7q_8 - 384q_5q_{11} - 384q_6q_{10} - 384q_7q_9 - 107904q_6q_{11} - 384q_7q_{10} + 768q_8q_9 - 107904q_7q_{11} + 768q_8q_{10} + 768q_8q_{11} + 768q_9q_{10} + 768q_9q_{11} + 768q_{10}q_{11} + 864$

A complete expression of $E_2(s)$ for 12-order Williamson based method or 36-order Baumert-Hall based method

$E_2(s) = 13728s_0 + 13728s_1 + 13728s_2 + 13728s_3 + 13728s_4 + 13728s_5 + 13728s_6 + 13728s_7 - 27456s_8 - 27456s_9 - 27456s_{10} - 27456s_{11} + 13488s_0s_1 + 48s_0s_2 + 48s_0s_3 + 48s_1s_2 + 48s_0s_4 + 48s_1s_3 + 48s_0s_5 + 48s_1s_4 + 13488s_2s_3 + 48s_0s_6 + 48s_1s_5 + 48s_2s_4 + 48s_0s_7 + 48s_1s_6 + 48s_2s_5 + 48s_3s_4 - 26976s_0s_8 + 48s_1s_7 + 48s_2s_6 + 48s_3s_5 - 96s_0s_9 - 26976s_1s_8 + 48s_2s_7 + 48s_3s_6 + 13488s_4s_5 - 96s_0s_{10} - 96s_1s_9 - 96s_2s_8 + 48s_3s_7 + 48s_4s_6 - 96s_0s_{11} - 96s_1s_{10} - 26976s_2s_9 - 96s_3s_8 + 48s_4s_7 + 48s_5s_6 - 96s_1s_{11} - 96s_2s_{10} - 26976s_3s_9 - 96s_4s_8 + 48s_5s_7 - 96s_2s_{11} - 96s_3s_{10} - 96s_4s_9 - 96s_5s_8 + 13488s_6s_7 - 96s_3s_{11} - 26976s_4s_{10} - 96s_5s_9 - 96s_6s_8 - 96s_4s_{11} - 26976s_5s_{10} - 96s_6s_9 - 96s_7s_8 - 96s_5s_{11} - 96s_6s_{10} - 96s_7s_9 - 26976s_6s_{11} - 96s_7s_{10} + 192s_8s_9 - 26976s_7s_{11} + 192s_8s_{10} + 192s_8s_{11} + 192s_9s_{10} + 192s_9s_{11} + 192s_{10}s_{11} + 162720$

A complete expression of $\hat{H}_2(\hat{\sigma}^z)$ for 12-order Williamson based method or 36-order Baumert-Hall based method

$\hat{H}_2(\hat{\sigma}^z) = 13728\hat{\sigma}^z_0 + 13728\hat{\sigma}^z_1 + 13728\hat{\sigma}^z_2 + 13728\hat{\sigma}^z_3 + 13728\hat{\sigma}^z_4 + 13728\hat{\sigma}^z_5 + 13728\hat{\sigma}^z_6 + 13728\hat{\sigma}^z_7 - 27456\hat{\sigma}^z_8 - 27456\hat{\sigma}^z_9 - 27456\hat{\sigma}^z_{10} - 27456\hat{\sigma}^z_{11} + 13488\hat{\sigma}^z_0\hat{\sigma}^z_1 + 48\hat{\sigma}^z_0\hat{\sigma}^z_2 + 48\hat{\sigma}^z_0\hat{\sigma}^z_3 + 48\hat{\sigma}^z_1\hat{\sigma}^z_2 + 48\hat{\sigma}^z_0\hat{\sigma}^z_4 + 48\hat{\sigma}^z_1\hat{\sigma}^z_3 + 48\hat{\sigma}^z_0\hat{\sigma}^z_5 + 48\hat{\sigma}^z_1\hat{\sigma}^z_4 + 13488\hat{\sigma}^z_2\hat{\sigma}^z_3 + 48\hat{\sigma}^z_0\hat{\sigma}^z_6 + 48\hat{\sigma}^z_1\hat{\sigma}^z_5 + 48\hat{\sigma}^z_2\hat{\sigma}^z_4 + 48\hat{\sigma}^z_0\hat{\sigma}^z_7 + 48\hat{\sigma}^z_1\hat{\sigma}^z_6 + 48\hat{\sigma}^z_2\hat{\sigma}^z_5 + 48\hat{\sigma}^z_3\hat{\sigma}^z_4 - 26976\hat{\sigma}^z_0\hat{\sigma}^z_8 + 48\hat{\sigma}^z_1\hat{\sigma}^z_7 + 48\hat{\sigma}^z_2\hat{\sigma}^z_6 + 48\hat{\sigma}^z_3\hat{\sigma}^z_5 - 96\hat{\sigma}^z_0\hat{\sigma}^z_9 - 26976\hat{\sigma}^z_1\hat{\sigma}^z_8 + 48\hat{\sigma}^z_2\hat{\sigma}^z_7 + 48\hat{\sigma}^z_3\hat{\sigma}^z_6 + 13488\hat{\sigma}^z_4\hat{\sigma}^z_5 -$

$96\hat{\sigma}_0^z\hat{\sigma}_{10}^z - 96\hat{\sigma}_1^z\hat{\sigma}_9^z - 96\hat{\sigma}_2^z\hat{\sigma}_8^z + 48\hat{\sigma}_3^z\hat{\sigma}_7^z + 48\hat{\sigma}_4^z\hat{\sigma}_6^z - 96\hat{\sigma}_0^z\hat{\sigma}_{11}^z - 96\hat{\sigma}_1^z\hat{\sigma}_{10}^z - 26976\hat{\sigma}_2^z\hat{\sigma}_9^z - 96\hat{\sigma}_3^z\hat{\sigma}_8^z + 48\hat{\sigma}_4^z\hat{\sigma}_7^z + 48\hat{\sigma}_5^z\hat{\sigma}_6^z - 96\hat{\sigma}_1^z\hat{\sigma}_{11}^z - 96\hat{\sigma}_2^z\hat{\sigma}_{10}^z - 26976\hat{\sigma}_3^z\hat{\sigma}_9^z - 96\hat{\sigma}_4^z\hat{\sigma}_8^z + 48\hat{\sigma}_5^z\hat{\sigma}_7^z - 96\hat{\sigma}_2^z\hat{\sigma}_{11}^z - 96\hat{\sigma}_3^z\hat{\sigma}_{10}^z - 96\hat{\sigma}_4^z\hat{\sigma}_9^z - 96\hat{\sigma}_5^z\hat{\sigma}_8^z + 13488\hat{\sigma}_6^z\hat{\sigma}_7^z - 96\hat{\sigma}_3^z\hat{\sigma}_{11}^z - 26976\hat{\sigma}_4^z\hat{\sigma}_{10}^z - 96\hat{\sigma}_5^z\hat{\sigma}_9^z - 96\hat{\sigma}_6^z\hat{\sigma}_8^z - 96\hat{\sigma}_4^z\hat{\sigma}_{11}^z - 26976\hat{\sigma}_5^z\hat{\sigma}_{10}^z - 96\hat{\sigma}_6^z\hat{\sigma}_9^z - 96\hat{\sigma}_7^z\hat{\sigma}_8^z - 96\hat{\sigma}_5^z\hat{\sigma}_{11}^z - 96\hat{\sigma}_6^z\hat{\sigma}_{10}^z - 96\hat{\sigma}_7^z\hat{\sigma}_9^z - 26976\hat{\sigma}_6^z\hat{\sigma}_{11}^z - 96\hat{\sigma}_7^z\hat{\sigma}_{10}^z + 192\hat{\sigma}_8^z\hat{\sigma}_9^z - 26976\hat{\sigma}_7^z\hat{\sigma}_{11}^z + 192\hat{\sigma}_8^z\hat{\sigma}_{10}^z + 192\hat{\sigma}_8^z\hat{\sigma}_{11}^z + 192\hat{\sigma}_9^z\hat{\sigma}_{10}^z + 192\hat{\sigma}_9^z\hat{\sigma}_{11}^z + 192\hat{\sigma}_{10}^z\hat{\sigma}_{11}^z + 162720$

A complete expression of $E_k(s)$ for 20-order Williamson based method or 60-order Baumert-Hall based method

$E_k(s) = 240s_0s_1 + 240s_0s_2 + 320s_1s_2 + 240s_3s_4 + 240s_3s_5 + 320s_4s_5 + 240s_6s_7 + 240s_6s_8 + 320s_7s_8 + 240s_9s_{10} + 240s_9s_{11} + 320s_{10}s_{11} + 80s_0s_1s_3s_4 + 80s_0s_1s_4s_5 + 80s_0s_2s_3s_5 + 80s_1s_2s_3s_4 + 80s_0s_2s_4s_5 + 80s_1s_2s_3s_5 + 160s_1s_2s_4s_5 + 80s_0s_1s_6s_7 + 80s_0s_1s_7s_8 + 80s_0s_2s_6s_8 + 80s_1s_2s_6s_7 + 80s_0s_2s_7s_8 + 80s_1s_2s_6s_8 + 160s_1s_2s_7s_8 + 80s_0s_1s_9s_{10} + 80s_3s_4s_6s_7 + 80s_0s_1s_{10}s_{11} + 80s_0s_2s_9s_{11} + 80s_1s_2s_9s_{10} + 80s_3s_4s_7s_8 + 80s_3s_5s_6s_8 + 80s_4s_5s_6s_7 + 80s_0s_2s_{10}s_{11} + 80s_1s_2s_9s_{11} + 80s_3s_5s_7s_8 + 80s_4s_5s_6s_8 + 160s_1s_2s_{10}s_{11} + 160s_4s_5s_7s_8 + 80s_3s_4s_9s_{10} + 80s_3s_4s_{10}s_{11} + 80s_3s_5s_9s_{11} + 80s_4s_5s_9s_{10} + 80s_3s_5s_{10}s_{11} + 80s_4s_5s_9s_{11} + 160s_4s_5s_{10}s_{11} + 80s_6s_7s_9s_{10} + 80s_6s_7s_{10}s_{11} + 80s_6s_8s_9s_{11} + 80s_7s_8s_9s_{10} + 80s_6s_8s_{10}s_{11} + 80s_7s_8s_9s_{11} + 160s_7s_8s_{10}s_{11} + 960$

A complete expression of $E_k(q)$ for 20-order Williamson based method or 60-order Baumert-Hall based method by taking $\delta = 681,600$

$E_k(q) = 2880q_0q_1 - 4000q_1 - 4000q_2 - 2880q_3 - 4000q_4 - 4000q_5 - 2880q_6 - 4000q_7 - 4000q_8 - 2880q_9 - 4000q_{10} - 4000q_{11} - 2880q_0 + 2880q_0q_2 + 640q_0q_3 + 5120q_1q_2 + 960q_0q_4 + 960q_1q_3 + 960q_0q_5 + 1600q_1q_4 + 960q_2q_3 + 640q_0q_6 + 1280q_1q_5 + 1280q_2q_4 + 960q_0q_7 + 960q_1q_6 + 1600q_2q_5 + 2880q_3q_4 + 960q_0q_8 + 1600q_1q_7 + 960q_2q_6 + 2880q_3q_5 + 640q_0q_9 + 1280q_1q_8 + 1280q_2q_7 + 640q_3q_6 + 5120q_4q_5 + 960q_0q_{10} + 960q_1q_9 + 1600q_2q_8 + 960q_3q_7 + 960q_4q_6 + 960q_0q_{11} + 1600q_1q_{10} + 960q_2q_9 + 960q_3q_8 + 1600q_4q_7 + 960q_5q_6 + 1280q_1q_{11} + 1280q_2q_{10} + 640q_3q_9 + 1280q_4q_8 + 1280q_5q_7 + 1600q_2q_{11} + 960q_3q_{10} + 960q_4q_9 + 1600q_5q_8 + 2880q_6q_7 + 960q_3q_{11} + 1600q_4q_{10} + 960q_5q_9 + 2880q_6q_8 + 1280q_4q_{11} + 1280q_5q_{10} + 640q_6q_9 + 5120q_7q_8 + 1600q_5q_{11} + 960q_6q_{10} + 960q_7q_9 + 960q_6q_{11} + 1600q_7q_{10} + 960q_8q_9 + 1280q_7q_{11} + 1280q_8q_{10} + 1600q_8q_{11} + 2880q_9q_{10} + 2880q_9q_{11} + 5120q_{10}q_{11} - 640q_0q_1q_3 - 1280q_0q_1q_4 - 640q_0q_2q_3 - 640q_0q_1q_5 - 640q_0q_2q_4 - 1280q_1q_2q_3 - 640q_0q_1q_6 - 1280q_0q_2q_5 - 640q_0q_3q_4 - 1920q_1q_2q_4 - 1280q_0q_1q_7 - 640q_0q_2q_6 - 640q_0q_3q_5 - 1920q_1q_2q_5 - 1280q_1q_3q_4 - 640q_0q_1q_8 - 640q_0q_2q_7 - 1280q_0q_4q_5 - 1280q_1q_2q_6 - 640q_1q_3q_5 - 640q_2q_3q_4 - 640q_0q_1q_9 - 1280q_0q_2q_8 - 1920q_1q_2q_7 - 1920q_1q_4q_5 - 1280q_2q_3q_5 - 1280q_0q_1q_{10} - 640q_0q_2q_9 - 1920q_1q_2q_8 - 1920q_2q_4q_5 - 640q_0q_1q_{11} - 640q_0q_2q_{10} - 1280q_1q_2q_9 - 1280q_0q_2q_{11} - 640q_0q_6q_7 - 1920q_1q_2q_{10} - 640q_3q_4q_6 - 640q_0q_6q_8 - 1920q_1q_2q_{11} - 1280q_1q_6q_7 - 1280q_3q_4q_7 - 640q_3q_5q_6 - 1280q_0q_7q_8 - 640q_1q_6q_8 - 640q_2q_6q_7 - 640q_3q_4q_8 - 640q_3q_5q_7 - 1280q_4q_5q_6 - 1920q_1q_7q_8 - 1280q_2q_6q_8 - 640q_3q_4q_9 - 1280q_3q_5q_8 - 640q_3q_6q_7 - 1920q_4q_5q_7 - 1920q_2q_7q_8 - 1280q_3q_4q_{10} - 640q_3q_5q_9 - 640q_3q_6q_8 - 1920q_4q_5q_8 - 1280q_4q_6q_7 - 640q_3q_4q_{11} - 640q_3q_5q_{10} - 1280q_3q_7q_8 - 1280q_4q_5q_9 - 640q_4q_6q_8 - 640q_5q_6q_7 - 640q_0q_9q_{10} - 1280q_3q_5q_{11} - 1920q_4q_5q_{10} - 1920q_4q_7q_8 - 1280q_5q_6q_8 - 640q_0q_9q_{11} - 1280q_1q_9q_{10} - 1920q_4q_5q_{11} - 1920q_5q_7q_8 - 1280q_0q_{10}q_{11} - 640q_1q_9q_{11} - 640q_2q_9q_{10} - 1920q_1q_{10}q_{11} - 1280q_2q_9q_{11} - 640q_3q_9q_{10} - 640q_6q_7q_9 - 1920q_2q_{10}q_{11} - 640q_3q_9q_{11} - 1280q_4q_9q_{10} - 1280q_6q_7q_{10} - 640q_6q_8q_9 - 1280q_3q_{10}q_{11} - 640q_4q_9q_{11} - 640q_5q_9q_{10} - 640q_6q_7q_{11} - 640q_6q_8q_{10} - 1280q_7q_8q_9 - 1920q_4q_{10}q_{11} - 1280q_5q_9q_{11} - 1280q_6q_8q_{11} - 640q_6q_9q_{10} - 1920q_7q_8q_{10} - 1920q_5q_{10}q_{11} - 640q_6q_9q_{11} - 1920q_7q_8q_{11} - 1280q_7q_9q_{10} - 1280q_6q_{10}q_{11} - 640q_7q_9q_{11} - 640q_8q_9q_{10} - 1920q_7q_{10}q_{11} - 1280q_8q_9q_{11} - 1920q_8q_{10}q_{11} + 1280q_0q_1q_3q_4 + 1280q_0q_1q_4q_5 + 1280q_0q_2q_3q_5 + 1280q_1q_2q_3q_4 + 1280q_0q_2q_4q_5 + 1280q_1q_2q_3q_5 + 2560q_1q_2q_4q_5 + 1280q_0q_1q_6q_7 + 1280q_0q_1q_7q_8 + 1280q_0q_2q_6q_8 + 1280q_1q_2q_6q_7 + 1280q_0q_2q_7q_8 + 1280q_1q_2q_6q_8 + 2560q_1q_2q_7q_8 + 1280q_0q_1q_9q_{10} + 1280q_3q_4q_6q_7 + 1280q_0q_1q_{10}q_{11} + 1280q_0q_2q_9q_{11} + 1280q_1q_2q_9q_{10} + 1280q_3q_4q_7q_8 + 1280q_3q_5q_6q_8 + 1280q_4q_5q_6q_7 + 1280q_0q_2q_{10}q_{11} + 1280q_1q_2q_9q_{11} + 1280q_3q_5q_7q_8 + 1280q_4q_5q_6q_8 + 2560q_1q_2q_{10}q_{11} + 2560q_4q_5q_7q_8 + 1280q_3q_4q_9q_{10} + 1280q_3q_4q_{10}q_{11} + 1280q_3q_5q_9q_{11} + 1280q_4q_5q_9q_{10} + 1280q_3q_5q_{10}q_{11} + 1280q_4q_5q_9q_{11} + 2560q_4q_5q_{10}q_{11} + 1280q_6q_7q_9q_{10} + 1280q_6q_7q_{10}q_{11} + 1280q_6q_8q_9q_{11} + 1280q_7q_8q_9q_{10} + 1280q_6q_8q_{10}q_{11} + 1280q_7q_8q_9q_{11} + 2560q_7q_8q_{10}q_{11} + 8000$

A complete expression of $E_2(q)$ for 20-order Williamson based method or 60-order Baumert-Hall based method

$E_2(q) = 2047680q_{12} - 4000q_1 - 4000q_2 - 2880q_3 - 4000q_4 - 4000q_5 - 2880q_6 - 4000q_7 - 4000q_8 - 2880q_9 - 4000q_{10} - 4000q_{11} - 2880q_0 + 2047680q_{13} + 2049920q_{14} + 2047680q_{15} + 2047680q_{16} + 2049920q_{17} +$

$2047680q_{18} + 2047680q_{19} + 2049920q_{20} + 2047680q_{21} + 2047680q_{22} + 2049920q_{23} + 681600q_0q_1 +$
$681600q_0q_2 + 640q_0q_3 + 681600q_1q_2 + 960q_0q_4 + 960q_1q_3 + 960q_0q_5 + 1600q_1q_4 + 960q_2q_3 + 640q_0q_6 +$
$1280q_1q_5 + 1280q_2q_4 + 960q_0q_7 + 960q_1q_6 + 1600q_2q_5 + 681600q_3q_4 + 960q_0q_8 + 1600q_1q_7 + 960q_2q_6 +$
$681600q_3q_5 + 640q_0q_9 + 1280q_1q_8 + 1280q_2q_7 + 640q_3q_6 + 681600q_4q_5 + 960q_0q_{10} + 960q_1q_9 + 1600q_2q_8 +$
$960q_3q_7 + 960q_4q_6 + 960q_0q_{11} + 1600q_1q_{10} + 960q_2q_9 + 960q_3q_8 + 1600q_4q_7 + 960q_5q_6 - 1363200q_0q_{12} +$
$1280q_1q_{11} + 1280q_2q_{10} + 640q_3q_9 + 1280q_4q_8 + 1280q_5q_7 - 1363200q_0q_{13} - 1363200q_1q_{12} + 1600q_2q_{11} +$
$960q_3q_{10} + 960q_4q_9 + 1600q_5q_8 + 681600q_6q_7 + 960q_3q_{11} + 1600q_4q_{10} + 960q_5q_9 + 681600q_6q_8 - 640q_0q_{15} -$
$1363200q_1q_{14} - 1363200q_2q_{13} - 640q_3q_{12} + 1280q_4q_{11} + 1280q_5q_{10} + 640q_6q_9 + 681600q_7q_8 - 640q_0q_{16} -$
$1280q_1q_{15} - 1363200q_2q_{14} - 640q_3q_{13} - 1280q_4q_{12} + 1600q_5q_{11} + 960q_6q_{10} + 960q_7q_9 - 1280q_0q_{17} -$
$640q_1q_{16} - 640q_2q_{15} - 1280q_3q_{14} - 640q_4q_{13} - 640q_5q_{12} + 960q_6q_{11} + 1600q_7q_{10} + 960q_8q_9 - 640q_0q_{18} -$
$1920q_1q_{17} - 1280q_2q_{16} - 1363200q_3q_{15} - 1920q_4q_{14} - 1280q_5q_{13} - 640q_6q_{12} + 1280q_7q_{11} + 1280q_8q_{10} -$
$640q_0q_{19} - 1280q_1q_{18} - 1920q_2q_{17} - 1363200q_3q_{16} - 1363200q_4q_{15} - 1920q_5q_{14} - 640q_6q_{13} - 1280q_7q_{12} +$
$1600q_8q_{11} + 681600q_9q_{10} - 1280q_0q_{20} - 640q_1q_{19} - 640q_2q_{18} - 1280q_6q_{14} - 640q_7q_{13} - 640q_8q_{12} +$
$681600q_9q_{11} - 640q_0q_{21} - 1920q_1q_{20} - 1280q_2q_{19} - 640q_3q_{18} - 1363200q_4q_{17} - 1363200q_5q_{16} - 640q_6q_{15} -$
$1920q_7q_{14} - 1280q_8q_{13} - 640q_9q_{12} + 681600q_{10}q_{11} - 640q_0q_{22} - 1280q_1q_{21} - 1920q_2q_{20} - 640q_3q_{19} -$
$1280q_4q_{18} - 1363200q_5q_{17} - 640q_6q_{16} - 1280q_7q_{15} - 1920q_8q_{14} - 640q_9q_{13} - 1280q_{10}q_{12} - 1280q_0q_{23} -$
$640q_1q_{22} - 640q_2q_{21} - 1280q_3q_{20} - 640q_4q_{19} - 640q_5q_{18} - 1280q_6q_{17} - 640q_7q_{16} - 640q_8q_{15} - 1280q_9q_{14} -$
$640q_{10}q_{13} - 640q_{11}q_{12} - 1920q_1q_{23} - 1280q_2q_{22} - 640q_3q_{21} - 1920q_4q_{20} - 1280q_5q_{19} - 1363200q_6q_{18} -$
$1920q_7q_{17} - 1280q_8q_{16} - 640q_9q_{15} - 1920q_{10}q_{14} - 1280q_{11}q_{13} - 1920q_2q_{23} - 640q_3q_{22} - 1280q_4q_{21} -$
$1920q_5q_{20} - 1363200q_6q_{19} - 1363200q_7q_{18} - 1920q_8q_{17} - 640q_9q_{16} - 1280q_{10}q_{15} - 1920q_{11}q_{14} -$
$1280q_3q_{23} - 640q_4q_{22} - 640q_5q_{21} - 1280q_9q_{17} - 640q_{10}q_{16} - 640q_{11}q_{15} - 1920q_4q_{23} - 1280q_5q_{22} -$
$640q_6q_{21} - 1363200q_7q_{20} - 1363200q_8q_{19} - 640q_9q_{18} - 1920q_{10}q_{17} - 1280q_{11}q_{16} + 1280q_{12}q_{15} - 1920q_5q_{23} -$
$640q_6q_{22} - 1280q_7q_{21} - 1363200q_8q_{20} - 640q_9q_{19} - 1280q_{10}q_{18} - 1920q_{11}q_{17} - 1280q_6q_{23} - 640q_7q_{22} -$
$640q_8q_{21} - 1280q_9q_{20} - 640q_{10}q_{19} - 640q_{11}q_{18} + 1280q_{12}q_{17} + 1280q_{13}q_{16} + 1280q_{14}q_{15} - 1920q_7q_{23} -$
$1280q_8q_{22} - 1363200q_9q_{21} - 1920q_{10}q_{20} - 1280q_{11}q_{19} + 1280q_{12}q_{18} + 1280q_{13}q_{17} + 1280q_{14}q_{16} - 1920q_8q_{23} -$
$1363200q_9q_{22} - 1363200q_{10}q_{21} - 1920q_{11}q_{20} + 2560q_{14}q_{17} + 1280q_{12}q_{20} + 1280q_{13}q_{19} + 1280q_{14}q_{18} -$
$1363200q_{10}q_{23} - 1363200q_{11}q_{22} + 1280q_{12}q_{21} + 1280q_{13}q_{20} + 1280q_{14}q_{19} + 1280q_{15}q_{18} - 1363200q_{11}q_{23} +$
$2560q_{14}q_{20} + 1280q_{12}q_{23} + 1280q_{13}q_{22} + 1280q_{14}q_{21} + 1280q_{15}q_{20} + 1280q_{16}q_{19} + 1280q_{17}q_{18} + 1280q_{13}q_{23} +$
$1280q_{14}q_{22} + 1280q_{15}q_{21} + 1280q_{16}q_{20} + 1280q_{17}q_{19} + 2560q_{14}q_{23} + 2560q_{17}q_{20} + 1280q_{15}q_{23} + 1280q_{16}q_{22} +$
$1280q_{17}q_{21} + 1280q_{16}q_{23} + 1280q_{17}q_{22} + 1280q_{18}q_{21} + 2560q_{17}q_{23} + 1280q_{18}q_{23} + 1280q_{19}q_{22} + 1280q_{20}q_{21} +$
$1280q_{19}q_{23} + 1280q_{20}q_{22} + 2560q_{20}q_{23} + 8000$

A complete expression of $E_2(s)$ for 20-order Williamson based method or 60-order Baumert-Hall based method

$E_2(s) = 342240s_0 + 342800s_1 + 342800s_2 + 342240s_3 + 342800s_4 + 342800s_5 + 342240s_6 + 342800s_7 +$
$342800s_8 + 342240s_9 + 342800s_{10} + 342800s_{11} - 342240s_{12} - 342240s_{13} - 343360s_{14} - 342240s_{15} - 342240s_{16} -$
$343360s_{17} - 342240s_{18} - 342240s_{19} - 343360s_{20} - 342240s_{21} - 342240s_{22} - 343360s_{23} + 170400s_0s_1 +$
$170400s_0s_2 + 160s_0s_3 + 170400s_1s_2 + 240s_0s_4 + 240s_1s_3 + 240s_0s_5 + 400s_1s_4 + 240s_2s_3 + 160s_0s_6 + 320s_1s_5 +$
$320s_2s_4 + 240s_0s_7 + 240s_1s_6 + 400s_2s_5 + 170400s_3s_4 + 240s_0s_8 + 400s_1s_7 + 240s_2s_6 + 170400s_3s_5 + 160s_0s_9 +$
$320s_1s_8 + 320s_2s_7 + 160s_3s_6 + 170400s_4s_5 + 240s_0s_{10} + 240s_1s_9 + 400s_2s_8 + 240s_3s_7 + 240s_4s_6 + 240s_0s_{11} +$
$400s_1s_{10} + 240s_2s_9 + 240s_3s_8 + 400s_4s_7 + 240s_5s_6 - 340800s_0s_{12} + 320s_1s_{11} + 320s_2s_{10} + 160s_3s_9 + 320s_4s_8 +$
$320s_5s_7 - 340800s_0s_{13} - 340800s_1s_{12} + 400s_2s_{11} + 240s_3s_{10} + 240s_4s_9 + 400s_5s_8 + 170400s_6s_7 + 240s_3s_{11} +$
$400s_4s_{10} + 240s_5s_9 + 170400s_6s_8 - 160s_0s_{15} - 340800s_1s_{14} - 340800s_2s_{13} - 160s_3s_{12} + 320s_4s_{11} + 320s_5s_{10} +$
$160s_6s_9 + 170400s_7s_8 - 160s_0s_{16} - 320s_1s_{15} - 340800s_2s_{14} - 160s_3s_{13} - 320s_4s_{12} + 400s_5s_{11} + 240s_6s_{10} +$
$240s_7s_9 - 320s_0s_{17} - 160s_1s_{16} - 160s_2s_{15} - 320s_3s_{14} - 160s_4s_{13} - 160s_5s_{12} + 240s_6s_{11} + 400s_7s_{10} + 240s_8s_9 -$
$160s_0s_{18} - 480s_1s_{17} - 320s_2s_{16} - 340800s_3s_{15} - 480s_4s_{14} - 320s_5s_{13} - 160s_6s_{12} + 320s_7s_{11} + 320s_8s_{10} -$
$160s_0s_{19} - 320s_1s_{18} - 480s_2s_{17} - 340800s_3s_{16} - 340800s_4s_{15} - 480s_5s_{14} - 160s_6s_{13} - 320s_7s_{12} + 400s_8s_{11} +$
$170400s_9s_{10} - 320s_0s_{20} - 160s_1s_{19} - 160s_2s_{18} - 320s_6s_{14} - 160s_7s_{13} - 160s_8s_{12} + 170400s_9s_{11} - 160s_0s_{21} -$

$480s_1s_{20} - 320s_2s_{19} - 160s_3s_{18} - 340800s_4s_{17} - 340800s_5s_{16} - 160s_6s_{15} - 480s_7s_{14} - 320s_8s_{13} - 160s_9s_{12} +$
$170400s_{10}s_{11} - 160s_0s_{22} - 320s_1s_{21} - 480s_2s_{20} - 160s_3s_{19} - 320s_4s_{18} - 340800s_5s_{17} - 160s_6s_{16} - 320s_7s_{15} -$
$480s_8s_{14} - 160s_9s_{13} - 320s_{10}s_{12} - 320s_0s_{23} - 160s_1s_{22} - 160s_2s_{21} - 320s_3s_{20} - 160s_4s_{19} - 160s_5s_{18} - 320s_6s_{17} -$
$160s_7s_{16} - 160s_8s_{15} - 320s_9s_{14} - 160s_{10}s_{13} - 160s_{11}s_{12} - 480s_1s_{23} - 320s_2s_{22} - 160s_3s_{21} - 480s_4s_{20} - 320s_5s_{19} -$
$340800s_6s_{18} - 480s_7s_{17} - 320s_8s_{16} - 160s_9s_{15} - 480s_{10}s_{14} - 320s_{11}s_{13} - 480s_2s_{23} - 160s_3s_{22} - 320s_4s_{21} -$
$480s_5s_{20} - 340800s_6s_{19} - 340800s_7s_{18} - 480s_8s_{17} - 160s_9s_{16} - 320s_{10}s_{15} - 480s_{11}s_{14} - 320s_3s_{23} - 160s_4s_{22} -$
$160s_5s_{21} - 320s_9s_{17} - 160s_{10}s_{16} - 160s_{11}s_{15} - 480s_4s_{23} - 320s_5s_{22} - 160s_6s_{21} - 340800s_7s_{20} - 340800s_8s_{19} -$
$160s_9s_{18} - 480s_{10}s_{17} - 320s_{11}s_{16} + 320s_{12}s_{15} - 480s_5s_{23} - 160s_6s_{22} - 320s_7s_{21} - 340800s_8s_{20} - 160s_9s_{19} -$
$320s_{10}s_{18} - 480s_{11}s_{17} - 320s_6s_{23} - 160s_7s_{22} - 160s_8s_{21} - 320s_9s_{20} - 160s_{10}s_{19} - 160s_{11}s_{18} + 320s_{12}s_{17} +$
$320s_{13}s_{16} + 320s_{14}s_{15} - 480s_7s_{23} - 320s_8s_{22} - 340800s_9s_{21} - 480s_{10}s_{20} - 320s_{11}s_{19} + 320s_{12}s_{18} + 320s_{13}s_{17} +$
$320s_{14}s_{16} - 480s_8s_{23} - 340800s_9s_{22} - 340800s_{10}s_{21} - 480s_{11}s_{20} + 640s_{14}s_{17} + 320s_{12}s_{20} + 320s_{13}s_{19} + 320s_{14}s_{18} -$
$340800s_{10}s_{23} - 340800s_{11}s_{22} + 320s_{12}s_{21} + 320s_{13}s_{20} + 320s_{14}s_{19} + 320s_{15}s_{18} - 340800s_{11}s_{23} + 640s_{14}s_{20} +$
$320s_{12}s_{23} + 320s_{13}s_{22} + 320s_{14}s_{21} + 320s_{15}s_{20} + 320s_{16}s_{19} + 320s_{17}s_{18} + 320s_{13}s_{23} + 320s_{14}s_{22} + 320s_{15}s_{21} +$
$320s_{16}s_{20} + 320s_{17}s_{19} + 640s_{14}s_{23} + 640s_{17}s_{20} + 320s_{15}s_{23} + 320s_{16}s_{22} + 320s_{17}s_{21} + 320s_{16}s_{23} + 320s_{17}s_{22} +$
$320s_{18}s_{21} + 640s_{17}s_{23} + 320s_{18}s_{23} + 320s_{19}s_{22} + 320s_{20}s_{21} + 320s_{19}s_{23} + 320s_{20}s_{22} + 640s_{20}s_{23} + 6142400$

A complete expression of $\hat{H}_2\left(\hat{\sigma}^z\right)$ for 20-order Williamson based method or 60-order Baumert-Hall based method

$\hat{H}_2\left(\hat{\sigma}^z\right) = 342240\hat{\sigma}^z_0 + 342800\hat{\sigma}^z_1 + 342800\hat{\sigma}^z_2 + 342240\hat{\sigma}^z_3 + 342800\hat{\sigma}^z_4 + 342800\hat{\sigma}^z_5 + 342240\hat{\sigma}^z_6 + 342800\hat{\sigma}^z_7 +$
$342800\hat{\sigma}^z_8 + 342240\hat{\sigma}^z_9 + 342800\hat{\sigma}^z_{10} + 342800\hat{\sigma}^z_{11} - 342240\hat{\sigma}^z_{12} - 342240\hat{\sigma}^z_{13} - 343360\hat{\sigma}^z_{14} - 342240\hat{\sigma}^z_{15} -$
$342240\hat{\sigma}^z_{16} - 343360\hat{\sigma}^z_{17} - 342240\hat{\sigma}^z_{18} - 342240\hat{\sigma}^z_{19} - 343360\hat{\sigma}^z_{20} - 342240\hat{\sigma}^z_{21} - 342240\hat{\sigma}^z_{22} - 343360\hat{\sigma}^z_{23} +$
$170400\hat{\sigma}^z_0\hat{\sigma}^z_1 + 170400\hat{\sigma}^z_0\hat{\sigma}^z_2 + 160\hat{\sigma}^z_0\hat{\sigma}^z_3 + 170400\hat{\sigma}^z_1\hat{\sigma}^z_2 + 240\hat{\sigma}^z_0\hat{\sigma}^z_4 + 240\hat{\sigma}^z_1\hat{\sigma}^z_3 + 240\hat{\sigma}^z_0\hat{\sigma}^z_5 + 400\hat{\sigma}^z_1\hat{\sigma}^z_4 + 240\hat{\sigma}^z_2\hat{\sigma}^z_3 +$
$160\hat{\sigma}^z_0\hat{\sigma}^z_6 + 320\hat{\sigma}^z_1\hat{\sigma}^z_5 + 320\hat{\sigma}^z_2\hat{\sigma}^z_4 + 240\hat{\sigma}^z_0\hat{\sigma}^z_7 + 240\hat{\sigma}^z_1\hat{\sigma}^z_6 + 400\hat{\sigma}^z_2\hat{\sigma}^z_5 + 170400\hat{\sigma}^z_3\hat{\sigma}^z_4 + 240\hat{\sigma}^z_0\hat{\sigma}^z_8 + 400\hat{\sigma}^z_1\hat{\sigma}^z_7 +$
$240\hat{\sigma}^z_2\hat{\sigma}^z_6 + 170400\hat{\sigma}^z_3\hat{\sigma}^z_5 + 160\hat{\sigma}^z_0\hat{\sigma}^z_9 + 320\hat{\sigma}^z_1\hat{\sigma}^z_8 + 320\hat{\sigma}^z_2\hat{\sigma}^z_7 + 160\hat{\sigma}^z_3\hat{\sigma}^z_6 + 170400\hat{\sigma}^z_4\hat{\sigma}^z_5 + 240\hat{\sigma}^z_0\hat{\sigma}^z_{10} + 240\hat{\sigma}^z_1\hat{\sigma}^z_9 +$
$400\hat{\sigma}^z_2\hat{\sigma}^z_8 + 240\hat{\sigma}^z_3\hat{\sigma}^z_7 + 240\hat{\sigma}^z_4\hat{\sigma}^z_6 + 240\hat{\sigma}^z_0\hat{\sigma}^z_{11} + 400\hat{\sigma}^z_1\hat{\sigma}^z_{10} + 240\hat{\sigma}^z_2\hat{\sigma}^z_9 + 240\hat{\sigma}^z_3\hat{\sigma}^z_8 + 400\hat{\sigma}^z_4\hat{\sigma}^z_7 + 240\hat{\sigma}^z_5\hat{\sigma}^z_6 -$
$340800\hat{\sigma}^z_0\hat{\sigma}^z_{12} + 320\hat{\sigma}^z_1\hat{\sigma}^z_{11} + 320\hat{\sigma}^z_2\hat{\sigma}^z_{10} + 160\hat{\sigma}^z_3\hat{\sigma}^z_9 + 320\hat{\sigma}^z_4\hat{\sigma}^z_8 + 320\hat{\sigma}^z_5\hat{\sigma}^z_7 - 340800\hat{\sigma}^z_0\hat{\sigma}^z_{13} - 340800\hat{\sigma}^z_1\hat{\sigma}^z_{12} +$
$400\hat{\sigma}^z_2\hat{\sigma}^z_{11} + 240\hat{\sigma}^z_3\hat{\sigma}^z_{10} + 240\hat{\sigma}^z_4\hat{\sigma}^z_9 + 400\hat{\sigma}^z_5\hat{\sigma}^z_8 + 170400\hat{\sigma}^z_6\hat{\sigma}^z_7 + 240\hat{\sigma}^z_3\hat{\sigma}^z_{11} + 400\hat{\sigma}^z_4\hat{\sigma}^z_{10} + 240\hat{\sigma}^z_5\hat{\sigma}^z_9 + 170400\hat{\sigma}^z_6\hat{\sigma}^z_8 -$
$160\hat{\sigma}^z_0\hat{\sigma}^z_{15} - 340800\hat{\sigma}^z_1\hat{\sigma}^z_{14} - 340800\hat{\sigma}^z_2\hat{\sigma}^z_{13} - 160\hat{\sigma}^z_3\hat{\sigma}^z_{12} + 320\hat{\sigma}^z_4\hat{\sigma}^z_{11} + 320\hat{\sigma}^z_5\hat{\sigma}^z_{10} + 160\hat{\sigma}^z_6\hat{\sigma}^z_9 + 170400\hat{\sigma}^z_7\hat{\sigma}^z_8 -$
$160\hat{\sigma}^z_0\hat{\sigma}^z_{16} - 320\hat{\sigma}^z_1\hat{\sigma}^z_{15} - 340800\hat{\sigma}^z_2\hat{\sigma}^z_{14} - 160\hat{\sigma}^z_3\hat{\sigma}^z_{13} - 320\hat{\sigma}^z_4\hat{\sigma}^z_{12} + 400\hat{\sigma}^z_5\hat{\sigma}^z_{11} + 240\hat{\sigma}^z_6\hat{\sigma}^z_{10} + 240\hat{\sigma}^z_7\hat{\sigma}^z_9 - 320\hat{\sigma}^z_0\hat{\sigma}^z_{17} -$
$160\hat{\sigma}^z_1\hat{\sigma}^z_{16} - 160\hat{\sigma}^z_2\hat{\sigma}^z_{15} - 320\hat{\sigma}^z_3\hat{\sigma}^z_{14} - 160\hat{\sigma}^z_4\hat{\sigma}^z_{13} - 160\hat{\sigma}^z_5\hat{\sigma}^z_{12} + 240\hat{\sigma}^z_6\hat{\sigma}^z_{11} + 400\hat{\sigma}^z_7\hat{\sigma}^z_{10} + 240\hat{\sigma}^z_8\hat{\sigma}^z_9 - 160\hat{\sigma}^z_0\hat{\sigma}^z_{18} -$
$480\hat{\sigma}^z_1\hat{\sigma}^z_{17} - 320\hat{\sigma}^z_2\hat{\sigma}^z_{16} - 340800\hat{\sigma}^z_3\hat{\sigma}^z_{15} - 480\hat{\sigma}^z_4\hat{\sigma}^z_{14} - 320\hat{\sigma}^z_5\hat{\sigma}^z_{13} - 160\hat{\sigma}^z_6\hat{\sigma}^z_{12} + 320\hat{\sigma}^z_7\hat{\sigma}^z_{11} + 320\hat{\sigma}^z_8\hat{\sigma}^z_{10} -$
$160\hat{\sigma}^z_0\hat{\sigma}^z_{19} - 320\hat{\sigma}^z_1\hat{\sigma}^z_{18} - 480\hat{\sigma}^z_2\hat{\sigma}^z_{17} - 340800\hat{\sigma}^z_3\hat{\sigma}^z_{16} - 340800\hat{\sigma}^z_4\hat{\sigma}^z_{15} - 480\hat{\sigma}^z_5\hat{\sigma}^z_{14} - 160\hat{\sigma}^z_6\hat{\sigma}^z_{13} - 320\hat{\sigma}^z_7\hat{\sigma}^z_{12} +$
$400\hat{\sigma}^z_8\hat{\sigma}^z_{11} + 170400\hat{\sigma}^z_9\hat{\sigma}^z_{10} - 320\hat{\sigma}^z_0\hat{\sigma}^z_{20} - 160\hat{\sigma}^z_1\hat{\sigma}^z_{19} - 160\hat{\sigma}^z_2\hat{\sigma}^z_{18} - 320\hat{\sigma}^z_6\hat{\sigma}^z_{14} - 160\hat{\sigma}^z_7\hat{\sigma}^z_{13} - 160\hat{\sigma}^z_8\hat{\sigma}^z_{12} +$
$170400\hat{\sigma}^z_9\hat{\sigma}^z_{11} - 160\hat{\sigma}^z_0\hat{\sigma}^z_{21} - 480\hat{\sigma}^z_1\hat{\sigma}^z_{20} - 320\hat{\sigma}^z_2\hat{\sigma}^z_{19} - 160\hat{\sigma}^z_3\hat{\sigma}^z_{18} - 340800\hat{\sigma}^z_4\hat{\sigma}^z_{17} - 340800\hat{\sigma}^z_5\hat{\sigma}^z_{16} - 160\hat{\sigma}^z_6\hat{\sigma}^z_{15} -$
$480\hat{\sigma}^z_7\hat{\sigma}^z_{14} - 320\hat{\sigma}^z_8\hat{\sigma}^z_{13} - 160\hat{\sigma}^z_9\hat{\sigma}^z_{12} + 170400\hat{\sigma}^z_{10}\hat{\sigma}^z_{11} - 160\hat{\sigma}^z_0\hat{\sigma}^z_{22} - 320\hat{\sigma}^z_1\hat{\sigma}^z_{21} - 480\hat{\sigma}^z_2\hat{\sigma}^z_{20} - 160\hat{\sigma}^z_3\hat{\sigma}^z_{19} -$
$320\hat{\sigma}^z_4\hat{\sigma}^z_{18} - 340800\hat{\sigma}^z_5\hat{\sigma}^z_{17} - 160\hat{\sigma}^z_6\hat{\sigma}^z_{16} - 320\hat{\sigma}^z_7\hat{\sigma}^z_{15} - 480\hat{\sigma}^z_8\hat{\sigma}^z_{14} - 160\hat{\sigma}^z_9\hat{\sigma}^z_{13} - 320\hat{\sigma}^z_{10}\hat{\sigma}^z_{12} - 320\hat{\sigma}^z_0\hat{\sigma}^z_{23} -$
$160\hat{\sigma}^z_1\hat{\sigma}^z_{22} - 160\hat{\sigma}^z_2\hat{\sigma}^z_{21} - 320\hat{\sigma}^z_3\hat{\sigma}^z_{20} - 160\hat{\sigma}^z_4\hat{\sigma}^z_{19} - 160\hat{\sigma}^z_5\hat{\sigma}^z_{18} - 320\hat{\sigma}^z_6\hat{\sigma}^z_{17} - 160\hat{\sigma}^z_7\hat{\sigma}^z_{16} - 160\hat{\sigma}^z_8\hat{\sigma}^z_{15} - 320\hat{\sigma}^z_9\hat{\sigma}^z_{14} -$
$160\hat{\sigma}^z_{10}\hat{\sigma}^z_{13} - 160\hat{\sigma}^z_{11}\hat{\sigma}^z_{12} - 480\hat{\sigma}^z_1\hat{\sigma}^z_{23} - 320\hat{\sigma}^z_2\hat{\sigma}^z_{22} - 160\hat{\sigma}^z_3\hat{\sigma}^z_{21} - 480\hat{\sigma}^z_4\hat{\sigma}^z_{20} - 320\hat{\sigma}^z_5\hat{\sigma}^z_{19} - 340800\hat{\sigma}^z_6\hat{\sigma}^z_{18} -$
$480\hat{\sigma}^z_7\hat{\sigma}^z_{17} - 320\hat{\sigma}^z_8\hat{\sigma}^z_{16} - 160\hat{\sigma}^z_9\hat{\sigma}^z_{15} - 480\hat{\sigma}^z_{10}\hat{\sigma}^z_{14} - 320\hat{\sigma}^z_{11}\hat{\sigma}^z_{13} - 480\hat{\sigma}^z_2\hat{\sigma}^z_{23} - 160\hat{\sigma}^z_3\hat{\sigma}^z_{22} - 320\hat{\sigma}^z_4\hat{\sigma}^z_{21} - 480\hat{\sigma}^z_5\hat{\sigma}^z_{20} -$
$340800\hat{\sigma}^z_6\hat{\sigma}^z_{19} - 340800\hat{\sigma}^z_7\hat{\sigma}^z_{18} - 480\hat{\sigma}^z_8\hat{\sigma}^z_{17} - 160\hat{\sigma}^z_9\hat{\sigma}^z_{16} - 320\hat{\sigma}^z_{10}\hat{\sigma}^z_{15} - 480\hat{\sigma}^z_{11}\hat{\sigma}^z_{14} - 320\hat{\sigma}^z_3\hat{\sigma}^z_{23} - 160\hat{\sigma}^z_4\hat{\sigma}^z_{22} -$
$160\hat{\sigma}^z_5\hat{\sigma}^z_{21} - 320\hat{\sigma}^z_9\hat{\sigma}^z_{17} - 160\hat{\sigma}^z_{10}\hat{\sigma}^z_{16} - 160\hat{\sigma}^z_{11}\hat{\sigma}^z_{15} - 480\hat{\sigma}^z_4\hat{\sigma}^z_{23} - 320\hat{\sigma}^z_5\hat{\sigma}^z_{22} - 160\hat{\sigma}^z_6\hat{\sigma}^z_{21} - 340800\hat{\sigma}^z_7\hat{\sigma}^z_{20} -$
$340800\hat{\sigma}^z_8\hat{\sigma}^z_{19} - 160\hat{\sigma}^z_9\hat{\sigma}^z_{18} - 480\hat{\sigma}^z_{10}\hat{\sigma}^z_{17} - 320\hat{\sigma}^z_{11}\hat{\sigma}^z_{16} + 320\hat{\sigma}^z_{12}\hat{\sigma}^z_{15} - 480\hat{\sigma}^z_5\hat{\sigma}^z_{23} - 160\hat{\sigma}^z_6\hat{\sigma}^z_{22} - 320\hat{\sigma}^z_7\hat{\sigma}^z_{21} -$
$340800\hat{\sigma}^z_8\hat{\sigma}^z_{20} - 160\hat{\sigma}^z_9\hat{\sigma}^z_{19} - 320\hat{\sigma}^z_{10}\hat{\sigma}^z_{18} - 480\hat{\sigma}^z_{11}\hat{\sigma}^z_{17} - 320\hat{\sigma}^z_6\hat{\sigma}^z_{23} - 160\hat{\sigma}^z_7\hat{\sigma}^z_{22} - 160\hat{\sigma}^z_8\hat{\sigma}^z_{21} - 320\hat{\sigma}^z_9\hat{\sigma}^z_{20} -$
$160\hat{\sigma}^z_{10}\hat{\sigma}^z_{19} - 160\hat{\sigma}^z_{11}\hat{\sigma}^z_{18} + 320\hat{\sigma}^z_{12}\hat{\sigma}^z_{17} + 320\hat{\sigma}^z_{13}\hat{\sigma}^z_{16} + 320\hat{\sigma}^z_{14}\hat{\sigma}^z_{15} - 480\hat{\sigma}^z_7\hat{\sigma}^z_{23} - 320\hat{\sigma}^z_8\hat{\sigma}^z_{22} - 340800\hat{\sigma}^z_9\hat{\sigma}^z_{21} -$
$480\hat{\sigma}^z_{10}\hat{\sigma}^z_{20} - 320\hat{\sigma}^z_{11}\hat{\sigma}^z_{19} + 320\hat{\sigma}^z_{12}\hat{\sigma}^z_{18} + 320\hat{\sigma}^z_{13}\hat{\sigma}^z_{17} + 320\hat{\sigma}^z_{14}\hat{\sigma}^z_{16} - 480\hat{\sigma}^z_8\hat{\sigma}^z_{23} - 340800\hat{\sigma}^z_9\hat{\sigma}^z_{22} - 340800\hat{\sigma}^z_{10}\hat{\sigma}^z_{21} -$
$480\hat{\sigma}^z_{11}\hat{\sigma}^z_{20} + 640\hat{\sigma}^z_{14}\hat{\sigma}^z_{17} + 320\hat{\sigma}^z_{12}\hat{\sigma}^z_{20} + 320\hat{\sigma}^z_{13}\hat{\sigma}^z_{19} + 320\hat{\sigma}^z_{14}\hat{\sigma}^z_{18} - 340800\hat{\sigma}^z_{10}\hat{\sigma}^z_{23} - 340800\hat{\sigma}^z_{11}\hat{\sigma}^z_{22} + 320\hat{\sigma}^z_{12}\hat{\sigma}^z_{21} +$
$320\hat{\sigma}^z_{13}\hat{\sigma}^z_{20} + 320\hat{\sigma}^z_{14}\hat{\sigma}^z_{19} + 320\hat{\sigma}^z_{15}\hat{\sigma}^z_{18} - 340800\hat{\sigma}^z_{11}\hat{\sigma}^z_{23} + 640\hat{\sigma}^z_{14}\hat{\sigma}^z_{20} + 320\hat{\sigma}^z_{12}\hat{\sigma}^z_{23} + 320\hat{\sigma}^z_{13}\hat{\sigma}^z_{22} + 320\hat{\sigma}^z_{14}\hat{\sigma}^z_{21} +$
$320\hat{\sigma}^z_{15}\hat{\sigma}^z_{20} + 320\hat{\sigma}^z_{16}\hat{\sigma}^z_{19} + 320\hat{\sigma}^z_{17}\hat{\sigma}^z_{18} + 320\hat{\sigma}^z_{13}\hat{\sigma}^z_{23} + 320\hat{\sigma}^z_{14}\hat{\sigma}^z_{22} + 320\hat{\sigma}^z_{15}\hat{\sigma}^z_{21} + 320\hat{\sigma}^z_{16}\hat{\sigma}^z_{20} + 320\hat{\sigma}^z_{17}\hat{\sigma}^z_{19} +$

$640\hat{\sigma}^z_{14}\hat{\sigma}^z_{23} + 640\hat{\sigma}^z_{17}\hat{\sigma}^z_{20} + 320\hat{\sigma}^z_{15}\hat{\sigma}^z_{23} + 320\hat{\sigma}^z_{16}\hat{\sigma}^z_{22} + 320\hat{\sigma}^z_{17}\hat{\sigma}^z_{21} + 320\hat{\sigma}^z_{16}\hat{\sigma}^z_{23} + 320\hat{\sigma}^z_{17}\hat{\sigma}^z_{22} + 320\hat{\sigma}^z_{18}\hat{\sigma}^z_{21} + 640\hat{\sigma}^z_{17}\hat{\sigma}^z_{23} + 320\hat{\sigma}^z_{18}\hat{\sigma}^z_{23} + 320\hat{\sigma}^z_{19}\hat{\sigma}^z_{22} + 320\hat{\sigma}^z_{20}\hat{\sigma}^z_{21} + 320\hat{\sigma}^z_{19}\hat{\sigma}^z_{23} + 320\hat{\sigma}^z_{20}\hat{\sigma}^z_{22} + 640\hat{\sigma}^z_{20}\hat{\sigma}^z_{23} + 6142400$

A complete expression of $E_k(s)$ for 28-order Williamson based method or 84-order Baumert-Hall based method

$E_k(s) = 336s_0s_1 + 336s_0s_2 + 336s_0s_3 + 560s_1s_2 + 560s_1s_3 + 560s_2s_3 + 336s_4s_5 + 336s_4s_6 + 336s_4s_7 +$
$560s_5s_6 + 560s_5s_7 + 560s_6s_7 + 336s_8s_9 + 336s_8s_{10} + 336s_8s_{11} + 560s_9s_{10} + 560s_9s_{11} + 560s_{10}s_{11} + 336s_{12}s_{13} +$
$336s_{12}s_{14} + 336s_{12}s_{15} + 560s_{13}s_{14} + 560s_{13}s_{15} + 560s_{14}s_{15} + 336s_0s_1s_2s_3 + 112s_0s_1s_4s_5 + 112s_0s_1s_5s_6 +$
$112s_0s_2s_4s_6 + 112s_1s_2s_4s_5 + 112s_0s_1s_6s_7 + 112s_0s_2s_5s_7 + 112s_0s_3s_4s_7 + 112s_0s_3s_5s_6 + 112s_1s_2s_4s_7 + 224s_1s_2s_5s_6 +$
$112s_1s_3s_4s_6 + 112s_2s_3s_4s_5 + 112s_0s_2s_6s_7 + 112s_0s_3s_5s_7 + 112s_1s_2s_5s_7 + 112s_1s_3s_4s_7 + 112s_1s_3s_5s_6 + 112s_2s_3s_4s_6 +$
$112s_1s_2s_6s_7 + 224s_1s_3s_5s_7 + 112s_2s_3s_5s_6 + 112s_1s_3s_6s_7 + 112s_2s_3s_5s_7 + 112s_0s_1s_8s_9 + 224s_2s_3s_6s_7 + 112s_0s_1s_9s_{10} +$
$112s_0s_2s_8s_{10} + 112s_1s_2s_8s_9 + 112s_0s_1s_{10}s_{11} + 112s_0s_2s_9s_{11} + 112s_0s_3s_8s_{11} + 112s_0s_3s_9s_{10} + 112s_1s_2s_8s_{11} +$
$224s_1s_2s_9s_{10} + 112s_1s_3s_8s_{10} + 112s_2s_3s_8s_9 + 336s_4s_5s_6s_7 + 112s_0s_2s_{10}s_{11} + 112s_0s_3s_9s_{11} + 112s_1s_2s_9s_{11} +$
$112s_1s_3s_8s_{11} + 112s_1s_3s_9s_{10} + 112s_2s_3s_8s_{10} + 112s_1s_2s_{10}s_{11} + 224s_1s_3s_9s_{11} + 112s_2s_3s_9s_{10} + 112s_1s_3s_{10}s_{11} +$
$112s_2s_3s_9s_{11} + 112s_0s_1s_{12}s_{13} + 224s_2s_3s_{10}s_{11} + 112s_4s_5s_8s_9 + 112s_0s_1s_{13}s_{14} + 112s_0s_2s_{12}s_{14} + 112s_1s_2s_{12}s_{13} +$
$112s_4s_5s_9s_{10} + 112s_4s_6s_8s_{10} + 112s_5s_6s_8s_9 + 112s_0s_1s_{14}s_{15} + 112s_0s_2s_{13}s_{15} + 112s_0s_3s_{12}s_{15} + 112s_0s_3s_{13}s_{14} +$
$112s_1s_2s_{12}s_{15} + 224s_1s_2s_{13}s_{14} + 112s_1s_3s_{12}s_{14} + 112s_2s_3s_{12}s_{13} + 112s_4s_5s_{10}s_{11} + 112s_4s_6s_9s_{11} + 112s_4s_7s_8s_{11} +$
$112s_4s_7s_9s_{10} + 112s_5s_6s_8s_{11} + 224s_5s_6s_9s_{10} + 112s_5s_7s_8s_{10} + 112s_6s_7s_8s_9 + 112s_0s_2s_{14}s_{15} + 112s_0s_3s_{13}s_{15} +$
$112s_1s_2s_{13}s_{15} + 112s_1s_3s_{12}s_{15} + 112s_1s_3s_{13}s_{14} + 112s_2s_3s_{12}s_{14} + 112s_4s_6s_{10}s_{11} + 112s_4s_7s_9s_{11} + 112s_5s_6s_9s_{11} +$
$112s_5s_7s_8s_{11} + 112s_5s_7s_9s_{10} + 112s_6s_7s_8s_{10} + 112s_1s_2s_{14}s_{15} + 224s_1s_3s_{13}s_{15} + 112s_2s_3s_{13}s_{14} + 112s_5s_6s_{10}s_{11} +$
$224s_5s_7s_9s_{11} + 112s_6s_7s_9s_{10} + 112s_1s_3s_{14}s_{15} + 112s_2s_3s_{13}s_{15} + 112s_5s_7s_{10}s_{11} + 112s_6s_7s_9s_{11} + 224s_2s_3s_{14}s_{15} +$
$112s_4s_5s_{12}s_{13} + 224s_6s_7s_{10}s_{11} + 112s_4s_5s_{13}s_{14} + 112s_4s_6s_{12}s_{14} + 112s_5s_6s_{12}s_{13} + 112s_4s_5s_{14}s_{15} + 112s_4s_6s_{13}s_{15} +$
$112s_4s_7s_{12}s_{15} + 112s_4s_7s_{13}s_{14} + 112s_5s_6s_{12}s_{15} + 224s_5s_6s_{13}s_{14} + 112s_5s_7s_{12}s_{14} + 112s_6s_7s_{12}s_{13} + 336s_8s_9s_{10}s_{11} +$
$112s_4s_6s_{14}s_{15} + 112s_4s_7s_{13}s_{15} + 112s_5s_6s_{13}s_{15} + 112s_5s_7s_{12}s_{15} + 112s_5s_7s_{13}s_{14} + 112s_6s_7s_{12}s_{14} + 112s_5s_6s_{14}s_{15} +$
$224s_5s_7s_{13}s_{15} + 112s_6s_7s_{13}s_{14} + 112s_5s_7s_{14}s_{15} + 112s_6s_7s_{13}s_{15} + 224s_6s_7s_{14}s_{15} + 112s_8s_9s_{12}s_{13} + 112s_8s_9s_{13}s_{14} +$
$112s_8s_{10}s_{12}s_{14} + 112s_9s_{10}s_{12}s_{13} + 112s_8s_9s_{14}s_{15} + 112s_8s_{10}s_{13}s_{15} + 112s_8s_{11}s_{12}s_{15} + 112s_8s_{11}s_{13}s_{14} + 112s_9s_{10}s_{12}s_{15} +$
$224s_9s_{10}s_{13}s_{14} + 112s_9s_{11}s_{12}s_{14} + 112s_{10}s_{11}s_{12}s_{13} + 112s_8s_{10}s_{14}s_{15} + 112s_8s_{11}s_{13}s_{15} + 112s_9s_{10}s_{13}s_{15} +$
$112s_9s_{11}s_{12}s_{15} + 112s_9s_{11}s_{13}s_{14} + 112s_{10}s_{11}s_{12}s_{14} + 112s_9s_{10}s_{14}s_{15} + 224s_9s_{11}s_{13}s_{15} + 112s_{10}s_{11}s_{13}s_{14} +$
$112s_9s_{11}s_{14}s_{15} + 112s_{10}s_{11}s_{13}s_{15} + 224s_{10}s_{11}s_{14}s_{15} + 336s_{12}s_{13}s_{14}s_{15} + 2688$

A complete expression of $E_k(q)$ for 28-order Williamson based method or 84-order Baumert-Hall based method

$E_k(q) = 6720q_0q_1 - 13664q_1 - 13664q_2 - 13664q_3 - 8736q_4 - 13664q_5 - 13664q_6 - 13664q_7 -$
$8736q_8 - 13664q_9 - 13664q_{10} - 13664q_{11} - 8736q_{12} - 13664q_{13} - 13664q_{14} - 13664q_{15} - 8736q_0 +$
$6720q_0q_2 + 6720q_0q_3 + 11648q_1q_2 + 1344q_0q_4 + 11648q_1q_3 + 2240q_0q_5 + 2240q_1q_4 + 11648q_2q_3 +$
$2240q_0q_6 + 4032q_1q_5 + 2240q_2q_4 + 2240q_0q_7 + 3584q_1q_6 + 3584q_2q_5 + 2240q_3q_4 + 1344q_0q_8 + 3584q_1q_7 +$
$4032q_2q_6 + 3584q_3q_5 + 2240q_0q_9 + 2240q_1q_8 + 3584q_2q_7 + 3584q_3q_6 + 6720q_4q_5 + 2240q_0q_{10} + 4032q_1q_9 +$
$2240q_2q_8 + 4032q_3q_7 + 6720q_4q_6 + 2240q_0q_{11} + 3584q_1q_{10} + 3584q_2q_9 + 2240q_3q_8 + 6720q_4q_7 + 11648q_5q_6 +$
$1344q_0q_{12} + 3584q_1q_{11} + 4032q_2q_{10} + 3584q_3q_9 + 1344q_4q_8 + 11648q_5q_7 + 2240q_0q_{13} + 2240q_1q_{12} +$
$3584q_2q_{11} + 3584q_3q_{10} + 2240q_4q_9 + 2240q_5q_8 + 11648q_6q_7 + 2240q_0q_{14} + 4032q_1q_{13} + 2240q_2q_{12} +$
$4032q_3q_{11} + 2240q_4q_{10} + 4032q_5q_9 + 2240q_6q_8 + 2240q_0q_{15} + 3584q_1q_{14} + 3584q_2q_{13} + 2240q_3q_{12} +$
$2240q_4q_{11} + 3584q_5q_{10} + 3584q_6q_9 + 2240q_7q_8 + 3584q_1q_{15} + 4032q_2q_{14} + 3584q_3q_{13} + 1344q_4q_{12} +$
$3584q_5q_{11} + 4032q_6q_{10} + 3584q_7q_9 + 3584q_2q_{15} + 3584q_3q_{14} + 2240q_4q_{13} + 2240q_5q_{12} + 3584q_6q_{11} +$
$3584q_7q_{10} + 6720q_8q_9 + 4032q_3q_{15} + 2240q_4q_{14} + 4032q_5q_{13} + 2240q_6q_{12} + 4032q_7q_{11} + 6720q_8q_{10} +$
$2240q_4q_{15} + 3584q_5q_{14} + 3584q_6q_{13} + 2240q_7q_{12} + 6720q_8q_{11} + 11648q_9q_{10} + 3584q_5q_{15} + 4032q_6q_{14} +$
$3584q_7q_{13} + 1344q_8q_{12} + 11648q_9q_{11} + 3584q_6q_{15} + 3584q_7q_{14} + 2240q_8q_{13} + 2240q_9q_{12} + 11648q_{10}q_{11} +$
$4032q_7q_{15} + 2240q_8q_{14} + 4032q_9q_{13} + 2240q_{10}q_{12} + 2240q_8q_{15} + 3584q_9q_{14} + 3584q_{10}q_{13} + 2240q_{11}q_{12} +$
$3584q_9q_{15} + 4032q_{10}q_{14} + 3584q_{11}q_{13} + 3584q_{10}q_{15} + 3584q_{11}q_{14} + 6720q_{12}q_{13} + 4032q_{11}q_{15} + 6720q_{12}q_{14} +$
$6720q_{12}q_{15} + 11648q_{13}q_{14} + 11648q_{13}q_{15} + 11648q_{14}q_{15} - 2688q_0q_1q_2 - 2688q_0q_1q_3 - 896q_0q_1q_4 -$
$2688q_0q_2q_3 - 1792q_0q_1q_5 - 896q_0q_2q_4 - 2688q_1q_2q_3 - 1792q_0q_1q_6 - 896q_0q_2q_5 - 896q_0q_3q_4 - 1792q_1q_2q_4 -$

$896q_0q_1q_7 - 1792q_0q_2q_6 - 1792q_0q_3q_5 - 3584q_1q_2q_5 - 1792q_1q_3q_4 - 896q_0q_1q_8 - 1792q_0q_2q_7 - 896q_0q_3q_6 -$
$896q_0q_4q_5 - 2688q_1q_2q_6 - 2688q_1q_3q_5 - 1792q_2q_3q_4 - 1792q_0q_1q_9 - 896q_0q_2q_8 - 1792q_0q_3q_7 - 896q_0q_4q_6 -$
$2688q_1q_2q_7 - 2688q_1q_3q_6 - 1792q_1q_4q_5 - 2688q_2q_3q_5 - 1792q_0q_1q_{10} - 896q_0q_2q_9 - 896q_0q_3q_8 - 896q_0q_4q_7 -$
$1792q_0q_5q_6 - 1792q_1q_2q_8 - 3584q_1q_3q_7 - 896q_1q_4q_6 - 3584q_2q_3q_6 - 1792q_2q_4q_5 - 896q_0q_1q_{11} -$
$1792q_0q_2q_{10} - 1792q_0q_3q_9 - 1792q_0q_5q_7 - 3584q_1q_2q_9 - 1792q_1q_3q_8 - 1792q_1q_4q_7 - 3584q_1q_5q_6 -$
$2688q_2q_3q_7 - 1792q_2q_4q_6 - 896q_3q_4q_5 - 896q_0q_1q_{12} - 1792q_0q_2q_{11} - 896q_0q_3q_{10} - 1792q_0q_6q_7 -$
$2688q_1q_2q_{10} - 2688q_1q_3q_9 - 2688q_1q_5q_7 - 1792q_2q_3q_8 - 896q_2q_4q_7 - 2688q_2q_5q_6 - 1792q_3q_4q_6 -$
$1792q_0q_1q_{13} - 896q_0q_2q_{12} - 1792q_0q_3q_{11} - 2688q_1q_2q_{11} - 2688q_1q_3q_{10} - 2688q_1q_6q_7 - 2688q_2q_3q_9 -$
$2688q_2q_5q_7 - 1792q_3q_4q_7 - 2688q_3q_5q_6 - 1792q_0q_1q_{14} - 896q_0q_2q_{13} - 896q_0q_3q_{12} - 1792q_1q_2q_{12} -$
$3584q_1q_3q_{11} - 3584q_2q_3q_{10} - 3584q_2q_6q_7 - 3584q_3q_5q_7 - 2688q_4q_5q_6 - 896q_0q_1q_{15} - 1792q_0q_2q_{14} -$
$1792q_0q_3q_{13} - 3584q_1q_2q_{13} - 1792q_1q_3q_{12} - 2688q_2q_3q_{11} - 2688q_3q_6q_7 - 2688q_4q_5q_7 - 1792q_0q_2q_{15} -$
$896q_0q_3q_{14} - 896q_0q_8q_9 - 2688q_1q_2q_{14} - 2688q_1q_3q_{13} - 1792q_2q_3q_{12} - 896q_4q_5q_8 - 2688q_4q_6q_7 -$
$1792q_0q_3q_{15} - 896q_0q_8q_{10} - 2688q_1q_2q_{15} - 2688q_1q_3q_{14} - 1792q_1q_8q_9 - 2688q_2q_3q_{13} - 1792q_4q_5q_9 -$
$896q_4q_6q_8 - 2688q_5q_6q_7 - 896q_0q_8q_{11} - 1792q_0q_9q_{10} - 3584q_1q_3q_{15} - 896q_1q_8q_{10} - 3584q_2q_3q_{14} -$
$1792q_2q_8q_9 - 1792q_4q_5q_{10} - 896q_4q_6q_9 - 896q_4q_7q_8 - 1792q_5q_6q_8 - 1792q_0q_9q_{11} - 1792q_1q_8q_{11} -$
$3584q_1q_9q_{10} - 2688q_2q_3q_{15} - 1792q_2q_8q_{10} - 896q_3q_8q_9 - 896q_4q_5q_{11} - 1792q_4q_6q_{10} - 1792q_4q_7q_9 -$
$3584q_5q_6q_9 - 1792q_5q_7q_8 - 1792q_0q_{10}q_{11} - 2688q_1q_9q_{11} - 896q_2q_8q_{11} - 2688q_2q_9q_{10} - 1792q_3q_8q_{10} -$
$896q_4q_5q_{12} - 1792q_4q_6q_{11} - 896q_4q_7q_{10} - 896q_4q_8q_9 - 2688q_5q_6q_{10} - 2688q_5q_7q_9 - 1792q_6q_7q_8 -$
$2688q_1q_{10}q_{11} - 2688q_2q_9q_{11} - 1792q_3q_8q_{11} - 2688q_3q_9q_{10} - 1792q_4q_5q_{13} - 896q_4q_6q_{12} - 1792q_4q_7q_{11} -$
$896q_4q_8q_{10} - 2688q_5q_6q_{11} - 2688q_5q_7q_{10} - 1792q_5q_8q_9 - 2688q_6q_7q_9 - 3584q_2q_{10}q_{11} - 3584q_3q_9q_{11} -$
$1792q_4q_5q_{14} - 896q_4q_6q_{13} - 896q_4q_7q_{12} - 896q_4q_8q_{11} - 1792q_4q_9q_{10} - 1792q_5q_6q_{12} - 3584q_5q_7q_{11} -$
$896q_5q_8q_{10} - 3584q_6q_7q_{10} - 1792q_6q_8q_9 - 2688q_3q_{10}q_{11} - 896q_4q_5q_{15} - 1792q_4q_6q_{14} - 1792q_4q_7q_{13} -$
$1792q_4q_9q_{11} - 3584q_5q_6q_{13} - 1792q_5q_7q_{12} - 1792q_5q_8q_{11} - 3584q_5q_9q_{10} - 2688q_6q_7q_{11} - 1792q_6q_8q_{10} -$
$896q_7q_8q_9 - 896q_0q_{12}q_{13} - 1792q_4q_6q_{15} - 896q_4q_7q_{14} - 1792q_4q_{10}q_{11} - 2688q_5q_6q_{14} - 2688q_5q_7q_{13} -$
$2688q_5q_9q_{11} - 1792q_6q_7q_{12} - 896q_6q_8q_{11} - 2688q_6q_9q_{10} - 1792q_7q_8q_{10} - 896q_0q_{12}q_{14} - 1792q_1q_{12}q_{13} -$
$1792q_4q_7q_{15} - 2688q_5q_6q_{15} - 2688q_5q_7q_{14} - 2688q_5q_{10}q_{11} - 2688q_6q_7q_{13} - 2688q_6q_9q_{11} - 1792q_7q_8q_{11} -$
$2688q_7q_9q_{10} - 896q_0q_{12}q_{15} - 1792q_0q_{13}q_{14} - 896q_1q_{12}q_{14} - 1792q_2q_{12}q_{13} - 3584q_5q_7q_{15} - 3584q_6q_7q_{14} -$
$3584q_6q_{10}q_{11} - 3584q_7q_9q_{11} - 2688q_8q_9q_{10} - 1792q_0q_{13}q_{15} - 1792q_1q_{12}q_{15} - 3584q_1q_{13}q_{14} - 1792q_2q_{12}q_{14} -$
$896q_3q_{12}q_{13} - 2688q_6q_7q_{15} - 2688q_7q_{10}q_{11} - 2688q_8q_9q_{11} - 1792q_0q_{14}q_{15} - 2688q_1q_{13}q_{15} - 896q_2q_{12}q_{15} -$
$2688q_2q_{13}q_{14} - 1792q_3q_{12}q_{14} - 896q_4q_{12}q_{13} - 896q_8q_9q_{12} - 2688q_8q_{10}q_{11} - 2688q_1q_{14}q_{15} - 2688q_2q_{13}q_{15} -$
$1792q_3q_{12}q_{15} - 2688q_3q_{13}q_{14} - 896q_4q_{12}q_{14} - 1792q_5q_{12}q_{13} - 1792q_8q_9q_{13} - 896q_8q_{10}q_{12} - 2688q_9q_{10}q_{11} -$
$3584q_2q_{14}q_{15} - 3584q_3q_{13}q_{15} - 896q_4q_{12}q_{15} - 1792q_4q_{13}q_{14} - 896q_5q_{12}q_{14} - 1792q_6q_{12}q_{13} - 1792q_8q_9q_{14} -$
$896q_8q_{10}q_{13} - 896q_8q_{11}q_{12} - 1792q_9q_{10}q_{12} - 2688q_3q_{14}q_{15} - 1792q_4q_{13}q_{15} - 1792q_5q_{12}q_{15} - 3584q_5q_{13}q_{14} -$
$1792q_6q_{12}q_{14} - 896q_7q_{12}q_{13} - 896q_8q_9q_{15} - 1792q_8q_{10}q_{14} - 1792q_8q_{11}q_{13} - 3584q_9q_{10}q_{13} - 1792q_9q_{11}q_{12} -$
$1792q_4q_{14}q_{15} - 2688q_5q_{13}q_{15} - 896q_6q_{12}q_{15} - 2688q_6q_{13}q_{14} - 1792q_7q_{12}q_{14} - 1792q_8q_{10}q_{15} - 896q_8q_{11}q_{14} -$
$896q_8q_{12}q_{13} - 2688q_9q_{10}q_{14} - 2688q_9q_{11}q_{13} - 1792q_{10}q_{11}q_{12} - 2688q_5q_{14}q_{15} - 2688q_6q_{13}q_{15} - 1792q_7q_{12}q_{15} -$
$2688q_7q_{13}q_{14} - 1792q_8q_{11}q_{15} - 896q_8q_{12}q_{14} - 2688q_9q_{10}q_{15} - 2688q_9q_{11}q_{14} - 1792q_9q_{12}q_{13} - 2688q_{10}q_{11}q_{13} -$
$3584q_6q_{14}q_{15} - 3584q_7q_{13}q_{15} - 896q_8q_{12}q_{15} - 1792q_8q_{13}q_{14} - 3584q_9q_{11}q_{15} - 896q_9q_{12}q_{14} - 3584q_{10}q_{11}q_{14} -$
$1792q_{10}q_{12}q_{13} - 2688q_7q_{14}q_{15} - 1792q_8q_{13}q_{15} - 1792q_9q_{12}q_{15} - 3584q_9q_{13}q_{14} - 2688q_{10}q_{11}q_{15} -$
$1792q_{10}q_{12}q_{14} - 896q_{11}q_{12}q_{13} - 1792q_8q_{14}q_{15} - 2688q_9q_{13}q_{15} - 896q_{10}q_{12}q_{15} - 2688q_{10}q_{13}q_{14} - 1792q_{11}q_{12}q_{14} -$
$2688q_9q_{14}q_{15} - 2688q_{10}q_{13}q_{15} - 1792q_{11}q_{12}q_{15} - 2688q_{11}q_{13}q_{14} - 3584q_{10}q_{14}q_{15} - 3584q_{11}q_{13}q_{15} -$
$2688q_{12}q_{13}q_{14} - 2688q_{11}q_{14}q_{15} - 2688q_{12}q_{13}q_{15} - 2688q_{12}q_{14}q_{15} - 2688q_{13}q_{14}q_{15} + 5376q_0q_1q_2q_3 +$
$1792q_0q_1q_4q_5 + 1792q_0q_1q_5q_6 + 1792q_0q_2q_4q_6 + 1792q_1q_2q_4q_5 + 1792q_0q_1q_6q_7 + 1792q_0q_2q_5q_7 +$
$1792q_0q_3q_4q_7 + 1792q_0q_3q_5q_6 + 1792q_1q_2q_4q_7 + 3584q_1q_2q_5q_6 + 1792q_1q_3q_4q_6 + 1792q_2q_3q_4q_5 +$
$1792q_0q_2q_6q_7 + 1792q_0q_3q_5q_7 + 1792q_1q_2q_5q_7 + 1792q_1q_3q_4q_7 + 1792q_1q_3q_5q_6 + 1792q_2q_3q_4q_6 +$
$1792q_1q_2q_6q_7 + 3584q_1q_3q_5q_7 + 1792q_2q_3q_5q_6 + 1792q_1q_3q_6q_7 + 1792q_2q_3q_5q_7 + 1792q_0q_1q_8q_9 +$
$3584q_2q_3q_6q_7 + 1792q_0q_1q_9q_{10} + 1792q_0q_2q_8q_{10} + 1792q_1q_2q_8q_9 + 1792q_0q_1q_{10}q_{11} + 1792q_0q_2q_9q_{11} +$
$1792q_0q_3q_8q_{11} + 1792q_0q_3q_9q_{10} + 1792q_1q_2q_8q_{11} + 3584q_1q_2q_9q_{10} + 1792q_1q_3q_8q_{10} + 1792q_2q_3q_8q_9 +$

$5376q_4q_5q_6q_7 + 1792q_0q_2q_{10}q_{11} + 1792q_0q_3q_9q_{11} + 1792q_1q_2q_9q_{11} + 1792q_1q_3q_8q_{11} + 1792q_1q_3q_9q_{10} +$
$1792q_2q_3q_8q_{10} + 1792q_1q_2q_{10}q_{11} + 3584q_1q_3q_9q_{11} + 1792q_2q_3q_9q_{10} + 1792q_1q_3q_{10}q_{11} + 1792q_2q_3q_9q_{11} +$
$1792q_0q_1q_{12}q_{13} + 3584q_2q_3q_{10}q_{11} + 1792q_4q_5q_8q_9 + 1792q_0q_1q_{13}q_{14} + 1792q_0q_2q_{12}q_{14} + 1792q_1q_2q_{12}q_{13} +$
$1792q_4q_5q_9q_{10} + 1792q_4q_6q_8q_{10} + 1792q_5q_6q_8q_9 + 1792q_0q_1q_{14}q_{15} + 1792q_0q_2q_{13}q_{15} + 1792q_0q_3q_{12}q_{15} +$
$1792q_0q_3q_{13}q_{14} + 1792q_1q_2q_{12}q_{15} + 3584q_1q_2q_{13}q_{14} + 1792q_1q_3q_{12}q_{14} + 1792q_2q_3q_{12}q_{13} + 1792q_4q_5q_{10}q_{11} +$
$1792q_4q_6q_9q_{11} + 1792q_4q_7q_8q_{11} + 1792q_4q_7q_9q_{10} + 1792q_5q_6q_8q_{11} + 3584q_5q_6q_9q_{10} + 1792q_5q_7q_8q_{10} +$
$1792q_6q_7q_8q_9 + 1792q_0q_2q_{14}q_{15} + 1792q_0q_3q_{13}q_{15} + 1792q_1q_2q_{13}q_{15} + 1792q_1q_3q_{12}q_{15} + 1792q_1q_3q_{13}q_{14} +$
$1792q_2q_3q_{12}q_{14} + 1792q_4q_6q_{10}q_{11} + 1792q_4q_7q_9q_{11} + 1792q_5q_6q_9q_{11} + 1792q_5q_7q_8q_{11} + 1792q_5q_7q_9q_{10} +$
$1792q_6q_7q_8q_{10} + 1792q_1q_2q_{14}q_{15} + 3584q_1q_3q_{13}q_{15} + 1792q_2q_3q_{13}q_{14} + 1792q_5q_6q_{10}q_{11} + 3584q_5q_7q_9q_{11} +$
$1792q_6q_7q_9q_{10} + 1792q_1q_3q_{14}q_{15} + 1792q_2q_3q_{13}q_{15} + 1792q_5q_7q_{10}q_{11} + 1792q_6q_7q_9q_{11} + 3584q_2q_3q_{14}q_{15} +$
$1792q_4q_5q_{12}q_{13} + 3584q_6q_7q_{10}q_{11} + 1792q_4q_5q_{13}q_{14} + 1792q_4q_6q_{12}q_{14} + 1792q_5q_6q_{12}q_{13} + 1792q_4q_5q_{14}q_{15} +$
$1792q_4q_6q_{13}q_{15} + 1792q_4q_7q_{12}q_{15} + 1792q_4q_7q_{13}q_{14} + 1792q_5q_6q_{12}q_{15} + 3584q_5q_6q_{13}q_{14} + 1792q_5q_7q_{12}q_{14} +$
$1792q_6q_7q_{12}q_{13} + 5376q_8q_9q_{10}q_{11} + 1792q_4q_6q_{14}q_{15} + 1792q_4q_7q_{13}q_{15} + 1792q_5q_6q_{13}q_{15} + 1792q_5q_7q_{12}q_{15} +$
$1792q_5q_7q_{13}q_{14} + 1792q_6q_7q_{12}q_{14} + 1792q_5q_6q_{14}q_{15} + 3584q_5q_7q_{13}q_{15} + 1792q_6q_7q_{13}q_{14} + 1792q_5q_7q_{14}q_{15} +$
$1792q_6q_7q_{13}q_{15} + 3584q_6q_7q_{14}q_{15} + 1792q_8q_9q_{12}q_{13} + 1792q_8q_9q_{13}q_{14} + 1792q_8q_{10}q_{12}q_{14} + 1792q_9q_{10}q_{12}q_{13} +$
$1792q_8q_9q_{14}q_{15} + 1792q_8q_{10}q_{13}q_{15} + 1792q_8q_{11}q_{12}q_{15} + 1792q_8q_{11}q_{13}q_{14} + 1792q_9q_{10}q_{12}q_{15} + 3584q_9q_{10}q_{13}q_{14} +$
$1792q_9q_{11}q_{12}q_{14} + 1792q_{10}q_{11}q_{12}q_{13} + 1792q_8q_{10}q_{14}q_{15} + 1792q_8q_{11}q_{13}q_{15} + 1792q_9q_{10}q_{13}q_{15} + 1792q_9q_{11}q_{12}q_{15} +$
$1792q_9q_{11}q_{13}q_{14} + 1792q_{10}q_{11}q_{12}q_{14} + 1792q_9q_{10}q_{14}q_{15} + 3584q_9q_{11}q_{13}q_{15} + 1792q_{10}q_{11}q_{13}q_{14} + 1792q_9q_{11}q_{14}q_{15} +$
$1792q_{10}q_{11}q_{13}q_{15} + 3584q_{10}q_{11}q_{14}q_{15} + 5376q_{12}q_{13}q_{14}q_{15} + 32928$

A complete expression of $E_2(q)$ for 28-order Williamson based method or 84-order Baumert-Hall based method by taking $\delta = 3,355,968$

$E_2(q) = 10074624q_{16} - 13664q_1 - 13664q_2 - 13664q_3 - 8736q_4 - 13664q_5 - 13664q_6 - 13664q_7 -$
$8736q_8 - 13664q_9 - 13664q_{10} - 13664q_{11} - 8736q_{12} - 13664q_{13} - 13664q_{14} - 13664q_{15} - 8736q_0 +$
$10074624q_{17} + 10074624q_{18} + 10079552q_{19} + 10079552q_{20} + 10079552q_{21} + 10074624q_{22} + 10074624q_{23} +$
$10074624q_{24} + 10079552q_{25} + 10079552q_{26} + 10079552q_{27} + 10074624q_{28} + 10074624q_{29} + 10074624q_{30} +$
$10079552q_{31} + 10079552q_{32} + 10079552q_{33} + 10074624q_{34} + 10074624q_{35} + 10074624q_{36} + 10079552q_{37} +$
$10079552q_{38} + 10079552q_{39} + 3355968q_0q_1 + 3355968q_0q_2 + 3355968q_0q_3 + 3355968q_1q_2 + 1344q_0q_4 +$
$3355968q_1q_3 + 2240q_0q_5 + 2240q_1q_4 + 3355968q_2q_3 + 2240q_0q_6 + 4032q_1q_5 + 2240q_2q_4 + 2240q_0q_7 +$
$3584q_1q_6 + 3584q_2q_5 + 2240q_3q_4 + 1344q_0q_8 + 3584q_1q_7 + 4032q_2q_6 + 3584q_3q_5 + 2240q_0q_9 + 2240q_1q_8 +$
$3584q_2q_7 + 3584q_3q_6 + 3355968q_4q_5 + 2240q_0q_{10} + 4032q_1q_9 + 2240q_2q_8 + 4032q_3q_7 + 3355968q_4q_6 +$
$2240q_0q_{11} + 3584q_1q_{10} + 3584q_2q_9 + 2240q_3q_8 + 3355968q_4q_7 + 3355968q_5q_6 + 1344q_0q_{12} + 3584q_1q_{11} +$
$4032q_2q_{10} + 3584q_3q_9 + 1344q_4q_8 + 3355968q_5q_7 + 2240q_0q_{13} + 2240q_1q_{12} + 3584q_2q_{11} + 3584q_3q_{10} +$
$2240q_4q_9 + 2240q_5q_8 + 3355968q_6q_7 + 2240q_0q_{14} + 4032q_1q_{13} + 2240q_2q_{12} + 4032q_3q_{11} + 2240q_4q_{10} +$
$4032q_5q_9 + 2240q_6q_8 + 2240q_0q_{15} + 3584q_1q_{14} + 3584q_2q_{13} + 2240q_3q_{12} + 2240q_4q_{11} + 3584q_5q_{10} +$
$3584q_6q_9 + 2240q_7q_8 - 6711936q_0q_{16} + 3584q_1q_{15} + 4032q_2q_{14} + 3584q_3q_{13} + 1344q_4q_{12} + 3584q_5q_{11} +$
$4032q_6q_{10} + 3584q_7q_9 - 6711936q_0q_{17} - 6711936q_1q_{16} + 3584q_2q_{15} + 3584q_3q_{14} + 2240q_4q_{13} + 2240q_5q_{12} +$
$3584q_6q_{11} + 3584q_7q_{10} + 3355968q_8q_9 - 6711936q_0q_{18} - 2688q_2q_{16} + 4032q_3q_{15} + 2240q_4q_{14} + 4032q_5q_{13} +$
$2240q_6q_{12} + 4032q_7q_{11} + 3355968q_8q_{10} - 6711936q_2q_{17} - 2688q_3q_{16} + 2240q_4q_{15} + 3584q_5q_{14} + 3584q_6q_{13} +$
$2240q_7q_{12} + 3355968q_8q_{11} + 3355968q_9q_{10} - 6711936q_1q_{19} - 2688q_3q_{17} - 896q_4q_{16} + 3584q_5q_{15} +$
$4032q_6q_{14} + 3584q_7q_{13} + 1344q_8q_{12} + 3355968q_9q_{11} - 6711936q_1q_{20} - 6711936q_2q_{19} - 6711936q_3q_{18} -$
$896q_4q_{17} - 1792q_5q_{16} + 3584q_6q_{15} + 3584q_7q_{14} + 2240q_8q_{13} + 2240q_9q_{12} + 3355968q_{10}q_{11} - 896q_0q_{22} -$
$2688q_3q_{19} - 896q_4q_{18} - 896q_5q_{17} - 1792q_6q_{16} + 4032q_7q_{15} + 2240q_8q_{14} + 4032q_9q_{13} + 2240q_{10}q_{12} -$
$896q_0q_{23} - 1792q_1q_{22} - 6711936q_2q_{21} - 6711936q_3q_{20} - 1792q_4q_{19} - 1792q_5q_{18} - 1792q_6q_{17} - 896q_7q_{16} +$
$2240q_8q_{15} + 3584q_9q_{14} + 3584q_{10}q_{13} + 2240q_{11}q_{12} - 896q_0q_{24} - 896q_1q_{23} - 1792q_2q_{22} - 6711936q_3q_{21} -$
$1792q_4q_{20} - 3584q_5q_{19} - 896q_6q_{18} - 1792q_7q_{17} - 896q_8q_{16} + 3584q_9q_{15} + 4032q_{10}q_{14} + 3584q_{11}q_{13} -$
$1792q_0q_{25} - 1792q_1q_{24} - 1792q_2q_{23} - 896q_3q_{22} - 1792q_4q_{21} - 2688q_5q_{20} - 2688q_6q_{19} - 1792q_7q_{18} -$
$896q_8q_{17} - 1792q_9q_{16} + 3584q_{10}q_{15} + 3584q_{11}q_{14} + 3355968q_{12}q_{13} - 1792q_0q_{26} - 3584q_1q_{25} - 896q_2q_{24} -$

$1792q_3q_{23} - 6711936q_4q_{22} - 2688q_5q_{21} - 2688q_6q_{20} - 2688q_7q_{19} - 896q_8q_{18} - 896q_9q_{17} - 1792q_{10}q_{16} +$
$4032q_{11}q_{15} + 3355968q_{12}q_{14} - 1792q_0q_{27} - 2688q_1q_{26} - 2688q_2q_{25} - 1792q_3q_{24} - 6711936q_4q_{23} -$
$6711936q_5q_{22} - 3584q_6q_{21} - 3584q_7q_{20} - 1792q_8q_{19} - 1792q_9q_{18} - 1792q_{10}q_{17} - 896q_{11}q_{16} + 3355968q_{12}q_{15} +$
$3355968q_{13}q_{14} - 896q_0q_{28} - 2688q_1q_{27} - 2688q_2q_{26} - 2688q_3q_{25} - 6711936q_4q_{24} - 2688q_6q_{22} - 2688q_7q_{21} -$
$1792q_8q_{20} - 3584q_9q_{19} - 896q_{10}q_{18} - 1792q_{11}q_{17} - 896q_{12}q_{16} + 3355968q_{13}q_{15} - 896q_0q_{29} - 1792q_1q_{28} -$
$3584q_2q_{27} - 3584q_3q_{26} - 6711936q_6q_{23} - 2688q_7q_{22} - 1792q_8q_{21} - 2688q_9q_{20} - 2688q_{10}q_{19} - 1792q_{11}q_{18} -$
$896q_{12}q_{17} - 1792q_{13}q_{16} + 3355968q_{14}q_{15} - 896q_0q_{30} - 896q_1q_{29} - 1792q_2q_{28} - 2688q_3q_{27} - 6711936q_5q_{25} -$
$2688q_7q_{23} - 896q_8q_{22} - 2688q_9q_{21} - 2688q_{10}q_{20} - 2688q_{11}q_{19} - 896q_{12}q_{18} - 896q_{13}q_{17} - 1792q_{14}q_{16} -$
$1792q_0q_{31} - 1792q_1q_{30} - 1792q_2q_{29} - 896q_3q_{28} - 6711936q_5q_{26} - 6711936q_6q_{25} - 6711936q_7q_{24} -$
$896q_8q_{23} - 1792q_9q_{22} - 3584q_{10}q_{21} - 3584q_{11}q_{20} - 1792q_{12}q_{19} - 1792q_{13}q_{18} - 1792q_{14}q_{17} - 896q_{15}q_{16} -$
$1792q_0q_{32} - 3584q_1q_{31} - 896q_2q_{30} - 1792q_3q_{29} - 896q_4q_{28} - 2688q_7q_{25} - 896q_8q_{24} - 896q_9q_{23} -$
$1792q_{10}q_{22} - 2688q_{11}q_{21} - 1792q_{12}q_{20} - 3584q_{13}q_{19} - 896q_{14}q_{18} - 1792q_{15}q_{17} - 1792q_0q_{33} - 2688q_1q_{32} -$
$2688q_2q_{31} - 1792q_3q_{30} - 896q_4q_{29} - 1792q_5q_{28} - 6711936q_6q_{27} - 6711936q_7q_{26} - 1792q_8q_{25} - 1792q_9q_{24} -$
$1792q_{10}q_{23} - 896q_{11}q_{22} - 1792q_{12}q_{21} - 2688q_{13}q_{20} - 2688q_{14}q_{19} - 1792q_{15}q_{18} - 896q_0q_{34} - 2688q_1q_{33} -$
$2688q_2q_{32} - 2688q_3q_{31} - 896q_4q_{30} - 896q_5q_{29} - 1792q_6q_{28} - 6711936q_7q_{27} - 1792q_8q_{26} - 3584q_9q_{25} -$
$896q_{10}q_{24} - 1792q_{11}q_{23} - 896q_{12}q_{22} - 2688q_{13}q_{21} - 2688q_{14}q_{20} - 2688q_{15}q_{19} - 896q_0q_{35} - 1792q_1q_{34} -$
$3584q_2q_{33} - 3584q_3q_{32} - 1792q_4q_{31} - 1792q_5q_{30} - 1792q_6q_{29} - 896q_7q_{28} - 1792q_8q_{27} - 2688q_9q_{26} -$
$2688q_{10}q_{25} - 1792q_{11}q_{24} - 896q_{12}q_{23} - 1792q_{13}q_{22} - 3584q_{14}q_{21} - 3584q_{15}q_{20} - 896q_0q_{36} - 896q_1q_{35} -$
$1792q_2q_{34} - 2688q_3q_{33} - 1792q_4q_{32} - 3584q_5q_{31} - 896q_6q_{30} - 1792q_7q_{29} - 6711936q_8q_{28} - 2688q_9q_{27} -$
$2688q_{10}q_{26} - 2688q_{11}q_{25} - 896q_{12}q_{24} - 896q_{13}q_{23} - 1792q_{14}q_{22} - 2688q_{15}q_{21} - 1792q_0q_{37} - 1792q_1q_{36} -$
$1792q_2q_{35} - 896q_3q_{34} - 1792q_4q_{33} - 2688q_5q_{32} - 2688q_6q_{31} - 1792q_7q_{30} - 6711936q_8q_{29} - 6711936q_9q_{28} -$
$3584q_{10}q_{27} - 3584q_{11}q_{26} - 1792q_{12}q_{25} - 1792q_{13}q_{24} - 1792q_{14}q_{23} - 896q_{15}q_{22} + 5376q_{16}q_{21} - 1792q_0q_{38} -$
$3584q_1q_{37} - 896q_2q_{36} - 1792q_3q_{35} - 896q_4q_{34} - 2688q_5q_{33} - 2688q_6q_{32} - 2688q_7q_{31} - 6711936q_8q_{30} -$
$2688q_{10}q_{28} - 2688q_{11}q_{27} - 1792q_{12}q_{26} - 3584q_{13}q_{25} - 896q_{14}q_{24} - 1792q_{15}q_{23} + 1792q_{16}q_{22} - 1792q_0q_{39} -$
$2688q_1q_{38} - 2688q_2q_{37} - 1792q_3q_{36} - 896q_4q_{35} - 1792q_5q_{34} - 3584q_6q_{33} - 3584q_7q_{32} - 6711936q_{10}q_{29} -$
$2688q_{11}q_{28} - 1792q_{12}q_{27} - 2688q_{13}q_{26} - 2688q_{14}q_{25} - 1792q_{15}q_{24} - 2688q_1q_{39} - 2688q_2q_{38} - 2688q_3q_{37} -$
$896q_4q_{36} - 896q_5q_{35} - 1792q_6q_{34} - 2688q_7q_{33} - 6711936q_9q_{31} - 2688q_{11}q_{29} - 896q_{12}q_{28} - 2688q_{13}q_{27} -$
$2688q_{14}q_{26} - 2688q_{15}q_{25} + 1792q_{17}q_{23} - 3584q_2q_{39} - 3584q_3q_{38} - 1792q_4q_{37} - 1792q_5q_{36} - 1792q_6q_{35} -$
$896q_7q_{34} - 6711936q_9q_{32} - 6711936q_{10}q_{31} - 6711936q_{11}q_{30} - 896q_{12}q_{29} - 1792q_{13}q_{28} - 3584q_{14}q_{27} -$
$3584q_{15}q_{26} + 1792q_{16}q_{25} + 1792q_{19}q_{22} - 2688q_3q_{39} - 1792q_4q_{38} - 3584q_5q_{37} - 896q_6q_{36} - 1792q_7q_{35} -$
$896q_8q_{34} - 2688q_{11}q_{31} - 896q_{12}q_{30} - 896q_{13}q_{29} - 1792q_{14}q_{28} - 2688q_{15}q_{27} + 1792q_{18}q_{24} - 1792q_4q_{39} -$
$2688q_5q_{38} - 2688q_6q_{37} - 1792q_7q_{36} - 896q_8q_{35} - 1792q_9q_{34} - 6711936q_{10}q_{33} - 6711936q_{11}q_{32} -$
$1792q_{12}q_{31} - 1792q_{13}q_{30} - 1792q_{14}q_{29} - 896q_{15}q_{28} + 1792q_{16}q_{27} + 1792q_{17}q_{26} + 1792q_{18}q_{25} + 1792q_{19}q_{24} +$
$1792q_{20}q_{23} + 1792q_{21}q_{22} - 2688q_5q_{39} - 2688q_6q_{38} - 2688q_7q_{37} - 896q_8q_{36} - 896q_9q_{35} - 1792q_{10}q_{34} -$
$6711936q_{11}q_{33} - 1792q_{12}q_{32} - 3584q_{13}q_{31} - 896q_{14}q_{30} - 1792q_{15}q_{29} + 1792q_{16}q_{28} + 1792q_{17}q_{27} +$
$1792q_{18}q_{26} + 3584q_{19}q_{25} + 1792q_{20}q_{24} + 1792q_{21}q_{23} - 3584q_6q_{39} - 3584q_7q_{38} - 1792q_8q_{37} - 1792q_9q_{36} -$
$1792q_{10}q_{35} - 896q_{11}q_{34} - 1792q_{12}q_{33} - 2688q_{13}q_{32} - 2688q_{14}q_{31} - 1792q_{15}q_{30} + 1792q_{19}q_{26} + 1792q_{20}q_{25} -$
$2688q_7q_{39} - 1792q_8q_{38} - 3584q_9q_{37} - 896q_{10}q_{36} - 1792q_{11}q_{35} - 6711936q_{12}q_{34} - 2688q_{13}q_{33} - 2688q_{14}q_{32} -$
$2688q_{15}q_{31} + 1792q_{17}q_{29} + 1792q_{19}q_{27} + 3584q_{20}q_{26} + 1792q_{21}q_{25} - 1792q_8q_{39} - 2688q_9q_{38} - 2688q_{10}q_{37} -$
$1792q_{11}q_{36} - 6711936q_{12}q_{35} - 6711936q_{13}q_{34} - 3584q_{14}q_{33} - 3584q_{15}q_{32} + 1792q_{16}q_{31} + 1792q_{19}q_{28} +$
$1792q_{20}q_{27} + 1792q_{21}q_{26} - 2688q_9q_{39} - 2688q_{10}q_{38} - 2688q_{11}q_{37} - 6711936q_{12}q_{36} - 2688q_{14}q_{34} -$
$2688q_{15}q_{33} + 1792q_{18}q_{30} + 3584q_{21}q_{27} - 3584q_{10}q_{39} - 3584q_{11}q_{38} - 6711936q_{14}q_{35} - 2688q_{15}q_{34} +$
$1792q_{16}q_{33} + 1792q_{17}q_{32} + 1792q_{18}q_{31} + 1792q_{19}q_{30} + 1792q_{20}q_{29} + 1792q_{21}q_{28} + 5376q_{22}q_{27} - 2688q_{11}q_{39} -$
$6711936q_{13}q_{37} - 2688q_{15}q_{35} + 1792q_{16}q_{34} + 1792q_{17}q_{33} + 1792q_{18}q_{32} + 3584q_{19}q_{31} + 1792q_{20}q_{30} +$
$1792q_{21}q_{29} + 1792q_{22}q_{28} - 6711936q_{13}q_{38} - 6711936q_{14}q_{37} - 6711936q_{15}q_{36} + 1792q_{19}q_{32} + 1792q_{20}q_{31} -$
$2688q_{15}q_{37} + 1792q_{17}q_{35} + 1792q_{19}q_{33} + 3584q_{20}q_{32} + 1792q_{21}q_{31} + 1792q_{23}q_{29} - 6711936q_{14}q_{39} -$
$6711936q_{15}q_{38} + 1792q_{16}q_{37} + 1792q_{19}q_{34} + 1792q_{20}q_{33} + 1792q_{21}q_{32} + 1792q_{22}q_{31} + 1792q_{25}q_{28} -$
$6711936q_{15}q_{39} + 1792q_{18}q_{36} + 3584q_{21}q_{33} + 1792q_{24}q_{30} + 1792q_{16}q_{39} + 1792q_{17}q_{38} + 1792q_{18}q_{37} +$

$1792q_{19}q_{36}+1792q_{20}q_{35}+1792q_{21}q_{34}+1792q_{22}q_{33}+1792q_{23}q_{32}+1792q_{24}q_{31}+1792q_{25}q_{30}+1792q_{26}q_{29}+$
$1792q_{27}q_{28}+1792q_{17}q_{39}+1792q_{18}q_{38}+3584q_{19}q_{37}+1792q_{20}q_{36}+1792q_{21}q_{35}+1792q_{22}q_{34}+1792q_{23}q_{33}+$
$1792q_{24}q_{32}+3584q_{25}q_{31}+1792q_{26}q_{30}+1792q_{27}q_{29}+1792q_{19}q_{38}+1792q_{20}q_{37}+1792q_{25}q_{32}+1792q_{26}q_{31}+$
$1792q_{19}q_{39}+3584q_{20}q_{38}+1792q_{21}q_{37}+1792q_{23}q_{35}+1792q_{25}q_{33}+3584q_{26}q_{32}+1792q_{27}q_{31}+1792q_{20}q_{39}+$
$1792q_{21}q_{38}+1792q_{22}q_{37}+1792q_{25}q_{34}+1792q_{26}q_{33}+1792q_{27}q_{32}+3584q_{21}q_{39}+1792q_{24}q_{36}+3584q_{27}q_{33}+$
$1792q_{22}q_{39}+1792q_{23}q_{38}+1792q_{24}q_{37}+1792q_{25}q_{36}+1792q_{26}q_{35}+1792q_{27}q_{34}+5376q_{28}q_{33}+1792q_{23}q_{39}+$
$1792q_{24}q_{38}+3584q_{25}q_{37}+1792q_{26}q_{36}+1792q_{27}q_{35}+1792q_{28}q_{34}+1792q_{25}q_{38}+1792q_{26}q_{37}+1792q_{25}q_{39}+$
$3584q_{26}q_{38}+1792q_{27}q_{37}+1792q_{29}q_{35}+1792q_{26}q_{39}+1792q_{27}q_{38}+1792q_{28}q_{37}+1792q_{31}q_{34}+3584q_{27}q_{39}+$
$1792q_{30}q_{36}+1792q_{28}q_{39}+1792q_{29}q_{38}+1792q_{30}q_{37}+1792q_{31}q_{36}+1792q_{32}q_{35}+1792q_{33}q_{34}+1792q_{29}q_{39}+$
$1792q_{30}q_{38}+3584q_{31}q_{37}+1792q_{32}q_{36}+1792q_{33}q_{35}+1792q_{31}q_{38}+1792q_{32}q_{37}+1792q_{31}q_{39}+3584q_{32}q_{38}+$
$1792q_{33}q_{37}+1792q_{32}q_{39}+1792q_{33}q_{38}+3584q_{33}q_{39}+5376q_{34}q_{39}+32928$

A complete expression of $E_2(s)$ for 28-order Williamson based method or 84-order Baumert-Hall based method by taking $\delta = 3,355,968$.

$E_2(s) = 2521344s_0+2523808s_1+2524480s_2+2525824s_3+2521344s_4+2523808s_5+2524480s_6+2525824s_7+$
$2521344s_8+2523808s_9+2524480s_{10}+2525824s_{11}+2521344s_{12}+2523808s_{13}+2524480s_{14}+2525824s_{15}-$
$1681344s_{16}-1680672s_{17}-1681344s_{18}-1683136s_{19}-1683808s_{20}-1685152s_{21}-1681344s_{22}-1680672s_{23}-$
$1681344s_{24}-1683136s_{25}-1683808s_{26}-1685152s_{27}-1681344s_{28}-1680672s_{29}-1681344s_{30}-1683136s_{31}-$
$1683808s_{32}-1685152s_{33}-1681344s_{34}-1680672s_{35}-1681344s_{36}-1683136s_{37}-1683808s_{38}-1685152s_{39}+$
$838992s_0s_1+838992s_0s_2+838992s_0s_3+838992s_1s_2+336s_0s_4+838992s_1s_3+560s_0s_5+560s_1s_4+838992s_2s_3+$
$560s_0s_6+1008s_1s_5+560s_2s_4+560s_0s_7+896s_1s_6+896s_2s_5+560s_3s_4+336s_0s_8+896s_1s_7+1008s_2s_6+896s_3s_5+$
$560s_0s_9+560s_1s_8+896s_2s_7+896s_3s_6+838992s_4s_5+560s_0s_{10}+1008s_1s_9+560s_2s_8+1008s_3s_7+838992s_4s_6+$
$560s_0s_{11}+896s_1s_{10}+896s_2s_9+560s_3s_8+838992s_4s_7+838992s_5s_6+336s_0s_{12}+896s_1s_{11}+1008s_2s_{10}+896s_3s_9+$
$336s_4s_8+838992s_5s_7+560s_0s_{13}+560s_1s_{12}+896s_2s_{11}+896s_3s_{10}+560s_4s_9+560s_5s_8+838992s_6s_7+560s_0s_{14}+$
$1008s_1s_{13}+560s_2s_{12}+1008s_3s_{11}+560s_4s_{10}+1008s_5s_9+560s_6s_8+560s_0s_{15}+896s_1s_{14}+896s_2s_{13}+560s_3s_{12}+$
$560s_4s_{11}+896s_5s_{10}+896s_6s_9+560s_7s_8-1677984s_0s_{16}+896s_1s_{15}+1008s_2s_{14}+896s_3s_{13}+336s_4s_{12}+$
$896s_5s_{11}+1008s_6s_{10}+896s_7s_9-1677984s_0s_{17}-1677984s_1s_{16}+896s_2s_{15}+896s_3s_{14}+560s_4s_{13}+560s_5s_{12}+$
$896s_6s_{11}+896s_7s_{10}+838992s_8s_9-1677984s_0s_{18}-672s_2s_{16}+1008s_3s_{15}+560s_4s_{14}+1008s_5s_{13}+560s_6s_{12}+$
$1008s_7s_{11}+838992s_8s_{10}-1677984s_2s_{17}-672s_3s_{16}+560s_4s_{15}+896s_5s_{14}+896s_6s_{13}+560s_7s_{12}+838992s_8s_{11}+$
$838992s_9s_{10}-1677984s_1s_{19}-672s_3s_{17}-224s_4s_{16}+896s_5s_{15}+1008s_6s_{14}+896s_7s_{13}+336s_8s_{12}+838992s_9s_{11}-$
$1677984s_1s_{20}-1677984s_2s_{19}-1677984s_3s_{18}-224s_4s_{17}-448s_5s_{16}+896s_6s_{15}+896s_7s_{14}+560s_8s_{13}+$
$560s_9s_{12}+838992s_{10}s_{11}-224s_0s_{22}-672s_3s_{19}-224s_4s_{18}-224s_5s_{17}-448s_6s_{16}+1008s_7s_{15}+560s_8s_{14}+$
$1008s_9s_{13}+560s_{10}s_{12}-224s_0s_{23}-448s_1s_{22}-1677984s_2s_{21}-1677984s_3s_{20}-448s_4s_{19}-448s_5s_{18}-448s_6s_{17}-$
$224s_7s_{16}+560s_8s_{15}+896s_9s_{14}+896s_{10}s_{13}+560s_{11}s_{12}-224s_0s_{24}-224s_1s_{23}-448s_2s_{22}-1677984s_3s_{21}-$
$448s_4s_{20}-896s_5s_{19}-224s_6s_{18}-448s_7s_{17}-224s_8s_{16}+896s_9s_{15}+1008s_{10}s_{14}+896s_{11}s_{13}-448s_0s_{25}-$
$448s_1s_{24}-448s_2s_{23}-224s_3s_{22}-448s_4s_{21}-672s_5s_{20}-672s_6s_{19}-448s_7s_{18}-224s_8s_{17}-448s_9s_{16}+896s_{10}s_{15}+$
$896s_{11}s_{14}+838992s_{12}s_{13}-448s_0s_{26}-896s_1s_{25}-224s_2s_{24}-448s_3s_{23}-1677984s_4s_{22}-672s_5s_{21}-672s_6s_{20}-$
$672s_7s_{19}-224s_8s_{18}-224s_9s_{17}-448s_{10}s_{16}+1008s_{11}s_{15}+838992s_{12}s_{14}-448s_0s_{27}-672s_1s_{26}-672s_2s_{25}-$
$448s_3s_{24}-1677984s_4s_{23}-1677984s_5s_{22}-896s_6s_{21}-896s_7s_{20}-448s_8s_{19}-448s_9s_{18}-448s_{10}s_{17}-224s_{11}s_{16}+$
$838992s_{12}s_{15}+838992s_{13}s_{14}-224s_0s_{28}-672s_1s_{27}-672s_2s_{26}-672s_3s_{25}-1677984s_4s_{24}-672s_6s_{22}-672s_7s_{21}-$
$448s_8s_{20}-896s_9s_{19}-224s_{10}s_{18}-448s_{11}s_{17}-224s_{12}s_{16}+838992s_{13}s_{15}-224s_0s_{29}-448s_1s_{28}-896s_2s_{27}-$
$896s_3s_{26}-1677984s_6s_{23}-672s_7s_{22}-448s_8s_{21}-672s_9s_{20}-672s_{10}s_{19}-448s_{11}s_{18}-224s_{12}s_{17}-448s_{13}s_{16}+$
$838992s_{14}s_{15}-224s_0s_{30}-224s_1s_{29}-448s_2s_{28}-672s_3s_{27}-1677984s_5s_{25}-672s_7s_{23}-224s_8s_{22}-672s_9s_{21}-$
$672s_{10}s_{20}-672s_{11}s_{19}-224s_{12}s_{18}-224s_{13}s_{17}-448s_{14}s_{16}-448s_0s_{31}-448s_1s_{30}-448s_2s_{29}-224s_3s_{28}-$
$1677984s_5s_{26}-1677984s_6s_{25}-1677984s_7s_{24}-224s_8s_{23}-448s_9s_{22}-896s_{10}s_{21}-896s_{11}s_{20}-448s_{12}s_{19}-$
$448s_{13}s_{18}-448s_{14}s_{17}-224s_{15}s_{16}-448s_0s_{32}-896s_1s_{31}-224s_2s_{30}-448s_3s_{29}-224s_4s_{28}-672s_7s_{25}-$
$224s_8s_{24}-224s_9s_{23}-448s_{10}s_{22}-672s_{11}s_{21}-448s_{12}s_{20}-896s_{13}s_{19}-224s_{14}s_{18}-448s_{15}s_{17}-448s_0s_{33}-$
$672s_1s_{32}-672s_2s_{31}-448s_3s_{30}-224s_4s_{29}-448s_5s_{28}-1677984s_6s_{27}-1677984s_7s_{26}-448s_8s_{25}-448s_9s_{24}-$

$448s_{10}s_{23} - 224s_{11}s_{22} - 448s_{12}s_{21} - 672s_{13}s_{20} - 672s_{14}s_{19} - 448s_{15}s_{18} - 224s_{0}s_{34} - 672s_{1}s_{33} - 672s_{2}s_{32} -$
$672s_{3}s_{31} - 224s_{4}s_{30} - 224s_{5}s_{29} - 448s_{6}s_{28} - 1677984s_{7}s_{27} - 448s_{8}s_{26} - 896s_{9}s_{25} - 224s_{10}s_{24} - 448s_{11}s_{23} -$
$224s_{12}s_{22} - 672s_{13}s_{21} - 672s_{14}s_{20} - 672s_{15}s_{19} - 224s_{0}s_{35} - 448s_{1}s_{34} - 896s_{2}s_{33} - 896s_{3}s_{32} - 448s_{4}s_{31} -$
$448s_{5}s_{30} - 448s_{6}s_{29} - 224s_{7}s_{28} - 448s_{8}s_{27} - 672s_{9}s_{26} - 672s_{10}s_{25} - 448s_{11}s_{24} - 224s_{12}s_{23} - 448s_{13}s_{22} -$
$896s_{14}s_{21} - 896s_{15}s_{20} - 224s_{0}s_{36} - 224s_{1}s_{35} - 448s_{2}s_{34} - 672s_{3}s_{33} - 448s_{4}s_{32} - 896s_{5}s_{31} - 224s_{6}s_{30} - 448s_{7}s_{29} -$
$1677984s_{8}s_{28} - 672s_{9}s_{27} - 672s_{10}s_{26} - 672s_{11}s_{25} - 224s_{12}s_{24} - 224s_{13}s_{23} - 448s_{14}s_{22} - 672s_{15}s_{21} - 448s_{0}s_{37} -$
$448s_{1}s_{36} - 448s_{2}s_{35} - 224s_{3}s_{34} - 448s_{4}s_{33} - 672s_{5}s_{32} - 672s_{6}s_{31} - 448s_{7}s_{30} - 1677984s_{8}s_{29} - 1677984s_{9}s_{28} -$
$896s_{10}s_{27} - 896s_{11}s_{26} - 448s_{12}s_{25} - 448s_{13}s_{24} - 448s_{14}s_{23} - 224s_{15}s_{22} + 1344s_{16}s_{21} - 448s_{0}s_{38} - 896s_{1}s_{37} -$
$224s_{2}s_{36} - 448s_{3}s_{35} - 224s_{4}s_{34} - 672s_{5}s_{33} - 672s_{6}s_{32} - 672s_{7}s_{31} - 1677984s_{8}s_{30} - 672s_{10}s_{28} - 672s_{11}s_{27} -$
$448s_{12}s_{26} - 896s_{13}s_{25} - 224s_{14}s_{24} - 448s_{15}s_{23} + 448s_{16}s_{22} - 448s_{0}s_{39} - 672s_{1}s_{38} - 672s_{2}s_{37} - 448s_{3}s_{36} -$
$224s_{4}s_{35} - 448s_{5}s_{34} - 896s_{6}s_{33} - 896s_{7}s_{32} - 1677984s_{10}s_{29} - 672s_{11}s_{28} - 448s_{12}s_{27} - 672s_{13}s_{26} - 672s_{14}s_{25} -$
$448s_{15}s_{24} - 672s_{1}s_{39} - 672s_{2}s_{38} - 672s_{3}s_{37} - 224s_{4}s_{36} - 224s_{5}s_{35} - 448s_{6}s_{34} - 672s_{7}s_{33} - 1677984s_{9}s_{31} -$
$672s_{11}s_{29} - 224s_{12}s_{28} - 672s_{13}s_{27} - 672s_{14}s_{26} - 672s_{15}s_{25} + 448s_{17}s_{23} - 896s_{2}s_{39} - 896s_{3}s_{38} - 448s_{4}s_{37} -$
$448s_{5}s_{36} - 448s_{6}s_{35} - 224s_{7}s_{34} - 1677984s_{9}s_{32} - 1677984s_{10}s_{31} - 1677984s_{11}s_{30} - 224s_{12}s_{29} - 448s_{13}s_{28} -$
$896s_{14}s_{27} - 896s_{15}s_{26} + 448s_{16}s_{25} + 448s_{19}s_{22} - 672s_{3}s_{39} - 448s_{4}s_{38} - 896s_{5}s_{37} - 224s_{6}s_{36} - 448s_{7}s_{35} -$
$224s_{8}s_{34} - 672s_{11}s_{31} - 224s_{12}s_{30} - 224s_{13}s_{29} - 448s_{14}s_{28} - 672s_{15}s_{27} + 448s_{18}s_{24} - 448s_{4}s_{39} - 672s_{5}s_{38} -$
$672s_{6}s_{37} - 448s_{7}s_{36} - 224s_{8}s_{35} - 448s_{9}s_{34} - 1677984s_{10}s_{33} - 1677984s_{11}s_{32} - 448s_{12}s_{31} - 448s_{13}s_{30} -$
$448s_{14}s_{29} - 224s_{15}s_{28} + 448s_{16}s_{27} + 448s_{17}s_{26} + 448s_{18}s_{25} + 448s_{19}s_{24} + 448s_{20}s_{23} + 448s_{21}s_{22} - 672s_{5}s_{39} -$
$672s_{6}s_{38} - 672s_{7}s_{37} - 224s_{8}s_{36} - 224s_{9}s_{35} - 448s_{10}s_{34} - 1677984s_{11}s_{33} - 448s_{12}s_{32} - 896s_{13}s_{31} - 224s_{14}s_{30} -$
$448s_{15}s_{29} + 448s_{16}s_{28} + 448s_{17}s_{27} + 448s_{18}s_{26} + 896s_{19}s_{25} + 448s_{20}s_{24} + 448s_{21}s_{23} - 896s_{6}s_{39} - 896s_{7}s_{38} -$
$448s_{8}s_{37} - 448s_{9}s_{36} - 448s_{10}s_{35} - 224s_{11}s_{34} - 448s_{12}s_{33} - 672s_{13}s_{32} - 672s_{14}s_{31} - 448s_{15}s_{30} + 448s_{19}s_{26} +$
$448s_{20}s_{25} - 672s_{7}s_{39} - 448s_{8}s_{38} - 896s_{9}s_{37} - 224s_{10}s_{36} - 448s_{11}s_{35} - 1677984s_{12}s_{34} - 672s_{13}s_{33} - 672s_{14}s_{32} -$
$672s_{15}s_{31} + 448s_{17}s_{29} + 448s_{19}s_{27} + 896s_{20}s_{26} + 448s_{21}s_{25} - 448s_{8}s_{39} - 672s_{9}s_{38} - 672s_{10}s_{37} - 448s_{11}s_{36} -$
$1677984s_{12}s_{35} - 1677984s_{13}s_{34} - 896s_{14}s_{33} - 896s_{15}s_{32} + 448s_{16}s_{31} + 448s_{19}s_{28} + 448s_{20}s_{27} + 448s_{21}s_{26} -$
$672s_{9}s_{39} - 672s_{10}s_{38} - 672s_{11}s_{37} - 1677984s_{12}s_{36} - 672s_{14}s_{34} - 672s_{15}s_{33} + 448s_{18}s_{30} + 896s_{21}s_{27} - 896s_{10}s_{39} -$
$896s_{11}s_{38} - 1677984s_{14}s_{35} - 672s_{15}s_{34} + 448s_{16}s_{33} + 448s_{17}s_{32} + 448s_{18}s_{31} + 448s_{19}s_{30} + 448s_{20}s_{29} + 448s_{21}s_{28} +$
$1344s_{22}s_{27} - 672s_{11}s_{39} - 1677984s_{13}s_{37} - 672s_{15}s_{35} + 448s_{16}s_{34} + 448s_{17}s_{33} + 448s_{18}s_{32} + 896s_{19}s_{31} +$
$448s_{20}s_{30} + 448s_{21}s_{29} + 448s_{22}s_{28} - 1677984s_{13}s_{38} - 1677984s_{14}s_{37} - 1677984s_{15}s_{36} + 448s_{19}s_{32} + 448s_{20}s_{31} -$
$672s_{15}s_{37} + 448s_{17}s_{35} + 448s_{19}s_{33} + 896s_{20}s_{32} + 448s_{21}s_{31} + 448s_{23}s_{29} - 1677984s_{14}s_{39} - 1677984s_{15}s_{38} +$
$448s_{16}s_{37} + 448s_{19}s_{34} + 448s_{20}s_{33} + 448s_{21}s_{32} + 448s_{22}s_{31} + 448s_{25}s_{28} - 1677984s_{15}s_{39} + 448s_{18}s_{36} + 896s_{21}s_{33} +$
$448s_{24}s_{30} + 448s_{16}s_{39} + 448s_{17}s_{38} + 448s_{18}s_{37} + 448s_{19}s_{36} + 448s_{20}s_{35} + 448s_{21}s_{34} + 448s_{22}s_{33} + 448s_{23}s_{32} +$
$448s_{24}s_{31} + 448s_{25}s_{30} + 448s_{26}s_{29} + 448s_{27}s_{28} + 448s_{17}s_{39} + 448s_{18}s_{38} + 896s_{19}s_{37} + 448s_{20}s_{36} + 448s_{21}s_{35} +$
$448s_{22}s_{34} + 448s_{23}s_{33} + 448s_{24}s_{32} + 896s_{25}s_{31} + 448s_{26}s_{30} + 448s_{27}s_{29} + 448s_{19}s_{38} + 448s_{20}s_{37} + 448s_{25}s_{32} +$
$448s_{26}s_{31} + 448s_{19}s_{39} + 896s_{20}s_{38} + 448s_{21}s_{37} + 448s_{23}s_{35} + 448s_{25}s_{33} + 896s_{26}s_{32} + 448s_{27}s_{31} + 448s_{20}s_{39} +$
$448s_{21}s_{38} + 448s_{22}s_{37} + 448s_{25}s_{34} + 448s_{26}s_{33} + 448s_{27}s_{32} + 896s_{21}s_{39} + 448s_{24}s_{36} + 896s_{27}s_{33} + 448s_{22}s_{39} +$
$448s_{23}s_{38} + 448s_{24}s_{37} + 448s_{25}s_{36} + 448s_{26}s_{35} + 448s_{27}s_{34} + 1344s_{28}s_{33} + 448s_{23}s_{39} + 448s_{24}s_{38} + 896s_{25}s_{37} +$
$448s_{26}s_{36} + 448s_{27}s_{35} + 448s_{28}s_{34} + 448s_{25}s_{38} + 448s_{26}s_{37} + 448s_{25}s_{39} + 896s_{26}s_{38} + 448s_{27}s_{37} + 448s_{29}s_{35} +$
$448s_{26}s_{39} + 448s_{27}s_{38} + 448s_{28}s_{37} + 448s_{31}s_{34} + 896s_{27}s_{39} + 448s_{30}s_{36} + 448s_{28}s_{39} + 448s_{29}s_{38} + 448s_{30}s_{37} +$
$448s_{31}s_{36} + 448s_{32}s_{35} + 448s_{33}s_{34} + 448s_{29}s_{39} + 448s_{30}s_{38} + 896s_{31}s_{37} + 448s_{32}s_{36} + 448s_{33}s_{35} + 448s_{31}s_{38} +$
$448s_{32}s_{37} + 448s_{31}s_{39} + 896s_{32}s_{38} + 448s_{33}s_{37} + 448s_{32}s_{39} + 448s_{33}s_{38} + 896s_{33}s_{39} + 1344s_{34}s_{39} + 60445728$

A complete expression of $\hat{H}_2\left(\hat{\sigma}^z\right)$ for 28-order Williamson based method or 84-order Baumert-Hall based method

$\hat{H}_2\left(\hat{\sigma}^z\right) = 2521344\hat{\sigma}^z_0 + 2523808\hat{\sigma}^z_1 + 2524480\hat{\sigma}^z_2 + 2525824\hat{\sigma}^z_3 + 2521344\hat{\sigma}^z_4 + 2523808\hat{\sigma}^z_5 + 2524480\hat{\sigma}^z_6 +$
$2525824\hat{\sigma}^z_7 + 2521344\hat{\sigma}^z_8 + 2523808\hat{\sigma}^z_9 + 2524480\hat{\sigma}^z_{10} + 2525824\hat{\sigma}^z_{11} + 2521344\hat{\sigma}^z_{12} + 2523808\hat{\sigma}^z_{13} + 2524480\hat{\sigma}^z_{14} +$
$2525824\hat{\sigma}^z_{15} - 1681344\hat{\sigma}^z_{16} - 1680672\hat{\sigma}^z_{17} - 1681344\hat{\sigma}^z_{18} - 1683136\hat{\sigma}^z_{19} - 1683808\hat{\sigma}^z_{20} - 1685152\hat{\sigma}^z_{21} -$
$1681344\hat{\sigma}^z_{22} - 1680672\hat{\sigma}^z_{23} - 1681344\hat{\sigma}^z_{24} - 1683136\hat{\sigma}^z_{25} - 1683808\hat{\sigma}^z_{26} - 1685152\hat{\sigma}^z_{27} - 1681344\hat{\sigma}^z_{28} -$
$1680672\hat{\sigma}^z_{29} - 1681344\hat{\sigma}^z_{30} - 1683136\hat{\sigma}^z_{31} - 1683808\hat{\sigma}^z_{32} - 1685152\hat{\sigma}^z_{33} - 1681344\hat{\sigma}^z_{34} - 1680672\hat{\sigma}^z_{35} -$

$1681344\hat{\sigma}^z_{36} - 1683136\hat{\sigma}^z_{37} - 1683808\hat{\sigma}^z_{38} - 1685152\hat{\sigma}^z_{39} + 838992\hat{\sigma}^z_{0}\hat{\sigma}^z_{1} + 838992\hat{\sigma}^z_{0}\hat{\sigma}^z_{2} + 838992\hat{\sigma}^z_{0}\hat{\sigma}^z_{3} +$
$838992\hat{\sigma}^z_{1}\hat{\sigma}^z_{2} + 336\hat{\sigma}^z_{0}\hat{\sigma}^z_{4} + 838992\hat{\sigma}^z_{1}\hat{\sigma}^z_{3} + 560\hat{\sigma}^z_{0}\hat{\sigma}^z_{5} + 560\hat{\sigma}^z_{1}\hat{\sigma}^z_{4} + 838992\hat{\sigma}^z_{2}\hat{\sigma}^z_{3} + 560\hat{\sigma}^z_{0}\hat{\sigma}^z_{6} + 1008\hat{\sigma}^z_{1}\hat{\sigma}^z_{5} +$
$560\hat{\sigma}^z_{2}\hat{\sigma}^z_{4} + 560\hat{\sigma}^z_{0}\hat{\sigma}^z_{7} + 896\hat{\sigma}^z_{1}\hat{\sigma}^z_{6} + 896\hat{\sigma}^z_{2}\hat{\sigma}^z_{5} + 560\hat{\sigma}^z_{3}\hat{\sigma}^z_{4} + 336\hat{\sigma}^z_{0}\hat{\sigma}^z_{8} + 896\hat{\sigma}^z_{1}\hat{\sigma}^z_{7} + 1008\hat{\sigma}^z_{2}\hat{\sigma}^z_{6} + 896\hat{\sigma}^z_{3}\hat{\sigma}^z_{5} + 560\hat{\sigma}^z_{0}\hat{\sigma}^z_{9} +$
$560\hat{\sigma}^z_{1}\hat{\sigma}^z_{8} + 896\hat{\sigma}^z_{2}\hat{\sigma}^z_{7} + 896\hat{\sigma}^z_{3}\hat{\sigma}^z_{6} + 838992\hat{\sigma}^z_{4}\hat{\sigma}^z_{5} + 560\hat{\sigma}^z_{0}\hat{\sigma}^z_{10} + 1008\hat{\sigma}^z_{1}\hat{\sigma}^z_{9} + 560\hat{\sigma}^z_{2}\hat{\sigma}^z_{8} + 1008\hat{\sigma}^z_{3}\hat{\sigma}^z_{7} + 838992\hat{\sigma}^z_{4}\hat{\sigma}^z_{6} +$
$560\hat{\sigma}^z_{0}\hat{\sigma}^z_{11} + 896\hat{\sigma}^z_{1}\hat{\sigma}^z_{10} + 896\hat{\sigma}^z_{2}\hat{\sigma}^z_{9} + 560\hat{\sigma}^z_{3}\hat{\sigma}^z_{8} + 838992\hat{\sigma}^z_{4}\hat{\sigma}^z_{7} + 838992\hat{\sigma}^z_{5}\hat{\sigma}^z_{6} + 336\hat{\sigma}^z_{0}\hat{\sigma}^z_{12} + 896\hat{\sigma}^z_{1}\hat{\sigma}^z_{11} +$
$1008\hat{\sigma}^z_{2}\hat{\sigma}^z_{10} + 896\hat{\sigma}^z_{3}\hat{\sigma}^z_{9} + 336\hat{\sigma}^z_{4}\hat{\sigma}^z_{8} + 838992\hat{\sigma}^z_{5}\hat{\sigma}^z_{7} + 560\hat{\sigma}^z_{0}\hat{\sigma}^z_{13} + 560\hat{\sigma}^z_{1}\hat{\sigma}^z_{12} + 896\hat{\sigma}^z_{2}\hat{\sigma}^z_{11} + 896\hat{\sigma}^z_{3}\hat{\sigma}^z_{10} + 560\hat{\sigma}^z_{4}\hat{\sigma}^z_{9} +$
$560\hat{\sigma}^z_{5}\hat{\sigma}^z_{8} + 838992\hat{\sigma}^z_{6}\hat{\sigma}^z_{7} + 560\hat{\sigma}^z_{0}\hat{\sigma}^z_{14} + 1008\hat{\sigma}^z_{1}\hat{\sigma}^z_{13} + 560\hat{\sigma}^z_{2}\hat{\sigma}^z_{12} + 1008\hat{\sigma}^z_{3}\hat{\sigma}^z_{11} + 560\hat{\sigma}^z_{4}\hat{\sigma}^z_{10} + 1008\hat{\sigma}^z_{5}\hat{\sigma}^z_{9} + 560\hat{\sigma}^z_{6}\hat{\sigma}^z_{8} +$
$560\hat{\sigma}^z_{0}\hat{\sigma}^z_{15} + 896\hat{\sigma}^z_{1}\hat{\sigma}^z_{14} + 896\hat{\sigma}^z_{2}\hat{\sigma}^z_{13} + 560\hat{\sigma}^z_{3}\hat{\sigma}^z_{12} + 560\hat{\sigma}^z_{4}\hat{\sigma}^z_{11} + 896\hat{\sigma}^z_{5}\hat{\sigma}^z_{10} + 896\hat{\sigma}^z_{6}\hat{\sigma}^z_{9} + 560\hat{\sigma}^z_{7}\hat{\sigma}^z_{8} - 1677984\hat{\sigma}^z_{0}\hat{\sigma}^z_{16} +$
$896\hat{\sigma}^z_{1}\hat{\sigma}^z_{15} + 1008\hat{\sigma}^z_{2}\hat{\sigma}^z_{14} + 896\hat{\sigma}^z_{3}\hat{\sigma}^z_{13} + 336\hat{\sigma}^z_{4}\hat{\sigma}^z_{12} + 896\hat{\sigma}^z_{5}\hat{\sigma}^z_{11} + 1008\hat{\sigma}^z_{6}\hat{\sigma}^z_{10} + 896\hat{\sigma}^z_{7}\hat{\sigma}^z_{9} - 1677984\hat{\sigma}^z_{0}\hat{\sigma}^z_{17} -$
$1677984\hat{\sigma}^z_{1}\hat{\sigma}^z_{16} + 896\hat{\sigma}^z_{2}\hat{\sigma}^z_{15} + 896\hat{\sigma}^z_{3}\hat{\sigma}^z_{14} + 560\hat{\sigma}^z_{4}\hat{\sigma}^z_{13} + 560\hat{\sigma}^z_{5}\hat{\sigma}^z_{12} + 896\hat{\sigma}^z_{6}\hat{\sigma}^z_{11} + 896\hat{\sigma}^z_{7}\hat{\sigma}^z_{10} + 838992\hat{\sigma}^z_{8}\hat{\sigma}^z_{9} -$
$1677984\hat{\sigma}^z_{0}\hat{\sigma}^z_{18} - 672\hat{\sigma}^z_{2}\hat{\sigma}^z_{16} + 1008\hat{\sigma}^z_{3}\hat{\sigma}^z_{15} + 560\hat{\sigma}^z_{4}\hat{\sigma}^z_{14} + 1008\hat{\sigma}^z_{5}\hat{\sigma}^z_{13} + 560\hat{\sigma}^z_{6}\hat{\sigma}^z_{12} + 1008\hat{\sigma}^z_{7}\hat{\sigma}^z_{11} + 838992\hat{\sigma}^z_{8}\hat{\sigma}^z_{10} -$
$1677984\hat{\sigma}^z_{2}\hat{\sigma}^z_{17} - 672\hat{\sigma}^z_{3}\hat{\sigma}^z_{16} + 560\hat{\sigma}^z_{4}\hat{\sigma}^z_{15} + 896\hat{\sigma}^z_{5}\hat{\sigma}^z_{14} + 896\hat{\sigma}^z_{6}\hat{\sigma}^z_{13} + 560\hat{\sigma}^z_{7}\hat{\sigma}^z_{12} + 838992\hat{\sigma}^z_{8}\hat{\sigma}^z_{11} + 838992\hat{\sigma}^z_{9}\hat{\sigma}^z_{10} -$
$1677984\hat{\sigma}^z_{1}\hat{\sigma}^z_{19} - 672\hat{\sigma}^z_{3}\hat{\sigma}^z_{17} - 224\hat{\sigma}^z_{4}\hat{\sigma}^z_{16} + 896\hat{\sigma}^z_{5}\hat{\sigma}^z_{15} + 1008\hat{\sigma}^z_{6}\hat{\sigma}^z_{14} + 896\hat{\sigma}^z_{7}\hat{\sigma}^z_{13} + 336\hat{\sigma}^z_{8}\hat{\sigma}^z_{12} + 838992\hat{\sigma}^z_{9}\hat{\sigma}^z_{11} -$
$1677984\hat{\sigma}^z_{1}\hat{\sigma}^z_{20} - 1677984\hat{\sigma}^z_{2}\hat{\sigma}^z_{19} - 1677984\hat{\sigma}^z_{3}\hat{\sigma}^z_{18} - 224\hat{\sigma}^z_{4}\hat{\sigma}^z_{17} - 448\hat{\sigma}^z_{5}\hat{\sigma}^z_{16} + 896\hat{\sigma}^z_{6}\hat{\sigma}^z_{15} + 896\hat{\sigma}^z_{7}\hat{\sigma}^z_{14} + 560\hat{\sigma}^z_{8}\hat{\sigma}^z_{13} +$
$560\hat{\sigma}^z_{9}\hat{\sigma}^z_{12} + 838992\hat{\sigma}^z_{10}\hat{\sigma}^z_{11} - 224\hat{\sigma}^z_{0}\hat{\sigma}^z_{22} - 672\hat{\sigma}^z_{3}\hat{\sigma}^z_{19} - 224\hat{\sigma}^z_{4}\hat{\sigma}^z_{18} - 224\hat{\sigma}^z_{5}\hat{\sigma}^z_{17} - 448\hat{\sigma}^z_{6}\hat{\sigma}^z_{16} + 1008\hat{\sigma}^z_{7}\hat{\sigma}^z_{15} +$
$560\hat{\sigma}^z_{8}\hat{\sigma}^z_{14} + 1008\hat{\sigma}^z_{9}\hat{\sigma}^z_{13} + 560\hat{\sigma}^z_{10}\hat{\sigma}^z_{12} - 224\hat{\sigma}^z_{0}\hat{\sigma}^z_{23} - 448\hat{\sigma}^z_{1}\hat{\sigma}^z_{22} - 1677984\hat{\sigma}^z_{2}\hat{\sigma}^z_{21} - 1677984\hat{\sigma}^z_{3}\hat{\sigma}^z_{20} - 448\hat{\sigma}^z_{4}\hat{\sigma}^z_{19} -$
$448\hat{\sigma}^z_{5}\hat{\sigma}^z_{18} - 448\hat{\sigma}^z_{6}\hat{\sigma}^z_{17} - 224\hat{\sigma}^z_{7}\hat{\sigma}^z_{16} + 560\hat{\sigma}^z_{8}\hat{\sigma}^z_{15} + 896\hat{\sigma}^z_{9}\hat{\sigma}^z_{14} + 896\hat{\sigma}^z_{10}\hat{\sigma}^z_{13} + 560\hat{\sigma}^z_{11}\hat{\sigma}^z_{12} - 224\hat{\sigma}^z_{0}\hat{\sigma}^z_{24} - 224\hat{\sigma}^z_{1}\hat{\sigma}^z_{23} -$
$448\hat{\sigma}^z_{2}\hat{\sigma}^z_{22} - 1677984\hat{\sigma}^z_{3}\hat{\sigma}^z_{21} - 448\hat{\sigma}^z_{4}\hat{\sigma}^z_{20} - 896\hat{\sigma}^z_{5}\hat{\sigma}^z_{19} - 224\hat{\sigma}^z_{6}\hat{\sigma}^z_{18} - 448\hat{\sigma}^z_{7}\hat{\sigma}^z_{17} - 224\hat{\sigma}^z_{8}\hat{\sigma}^z_{16} + 896\hat{\sigma}^z_{9}\hat{\sigma}^z_{15} +$
$1008\hat{\sigma}^z_{10}\hat{\sigma}^z_{14} + 896\hat{\sigma}^z_{11}\hat{\sigma}^z_{13} - 448\hat{\sigma}^z_{0}\hat{\sigma}^z_{25} - 448\hat{\sigma}^z_{1}\hat{\sigma}^z_{24} - 448\hat{\sigma}^z_{2}\hat{\sigma}^z_{23} - 224\hat{\sigma}^z_{3}\hat{\sigma}^z_{22} - 448\hat{\sigma}^z_{4}\hat{\sigma}^z_{21} - 672\hat{\sigma}^z_{5}\hat{\sigma}^z_{20} - 672\hat{\sigma}^z_{6}\hat{\sigma}^z_{19} -$
$448\hat{\sigma}^z_{7}\hat{\sigma}^z_{18} - 224\hat{\sigma}^z_{8}\hat{\sigma}^z_{17} - 448\hat{\sigma}^z_{9}\hat{\sigma}^z_{16} + 896\hat{\sigma}^z_{10}\hat{\sigma}^z_{15} + 896\hat{\sigma}^z_{11}\hat{\sigma}^z_{14} + 838992\hat{\sigma}^z_{12}\hat{\sigma}^z_{13} - 448\hat{\sigma}^z_{0}\hat{\sigma}^z_{26} - 896\hat{\sigma}^z_{1}\hat{\sigma}^z_{25} -$
$224\hat{\sigma}^z_{2}\hat{\sigma}^z_{24} - 448\hat{\sigma}^z_{3}\hat{\sigma}^z_{23} - 1677984\hat{\sigma}^z_{4}\hat{\sigma}^z_{22} - 672\hat{\sigma}^z_{5}\hat{\sigma}^z_{21} - 672\hat{\sigma}^z_{6}\hat{\sigma}^z_{20} - 672\hat{\sigma}^z_{7}\hat{\sigma}^z_{19} - 224\hat{\sigma}^z_{8}\hat{\sigma}^z_{18} - 224\hat{\sigma}^z_{9}\hat{\sigma}^z_{17} -$
$448\hat{\sigma}^z_{10}\hat{\sigma}^z_{16} + 1008\hat{\sigma}^z_{11}\hat{\sigma}^z_{15} + 838992\hat{\sigma}^z_{12}\hat{\sigma}^z_{14} - 448\hat{\sigma}^z_{0}\hat{\sigma}^z_{27} - 672\hat{\sigma}^z_{1}\hat{\sigma}^z_{26} - 672\hat{\sigma}^z_{2}\hat{\sigma}^z_{25} - 448\hat{\sigma}^z_{3}\hat{\sigma}^z_{24} - 1677984\hat{\sigma}^z_{4}\hat{\sigma}^z_{23} -$
$1677984\hat{\sigma}^z_{5}\hat{\sigma}^z_{22} - 896\hat{\sigma}^z_{6}\hat{\sigma}^z_{21} - 896\hat{\sigma}^z_{7}\hat{\sigma}^z_{20} - 448\hat{\sigma}^z_{8}\hat{\sigma}^z_{19} - 448\hat{\sigma}^z_{9}\hat{\sigma}^z_{18} - 448\hat{\sigma}^z_{10}\hat{\sigma}^z_{17} - 224\hat{\sigma}^z_{11}\hat{\sigma}^z_{16} + 838992\hat{\sigma}^z_{12}\hat{\sigma}^z_{15} +$
$838992\hat{\sigma}^z_{13}\hat{\sigma}^z_{14} - 224\hat{\sigma}^z_{0}\hat{\sigma}^z_{28} - 672\hat{\sigma}^z_{1}\hat{\sigma}^z_{27} - 672\hat{\sigma}^z_{2}\hat{\sigma}^z_{26} - 672\hat{\sigma}^z_{3}\hat{\sigma}^z_{25} - 1677984\hat{\sigma}^z_{4}\hat{\sigma}^z_{24} - 672\hat{\sigma}^z_{6}\hat{\sigma}^z_{22} - 672\hat{\sigma}^z_{7}\hat{\sigma}^z_{21} -$
$448\hat{\sigma}^z_{8}\hat{\sigma}^z_{20} - 896\hat{\sigma}^z_{9}\hat{\sigma}^z_{19} - 224\hat{\sigma}^z_{10}\hat{\sigma}^z_{18} - 448\hat{\sigma}^z_{11}\hat{\sigma}^z_{17} - 224\hat{\sigma}^z_{12}\hat{\sigma}^z_{16} + 838992\hat{\sigma}^z_{13}\hat{\sigma}^z_{15} - 224\hat{\sigma}^z_{0}\hat{\sigma}^z_{29} - 448\hat{\sigma}^z_{1}\hat{\sigma}^z_{28} -$
$896\hat{\sigma}^z_{2}\hat{\sigma}^z_{27} - 896\hat{\sigma}^z_{3}\hat{\sigma}^z_{26} - 1677984\hat{\sigma}^z_{6}\hat{\sigma}^z_{23} - 672\hat{\sigma}^z_{7}\hat{\sigma}^z_{22} - 448\hat{\sigma}^z_{8}\hat{\sigma}^z_{21} - 672\hat{\sigma}^z_{9}\hat{\sigma}^z_{20} - 672\hat{\sigma}^z_{10}\hat{\sigma}^z_{19} - 448\hat{\sigma}^z_{11}\hat{\sigma}^z_{18} -$
$224\hat{\sigma}^z_{12}\hat{\sigma}^z_{17} - 448\hat{\sigma}^z_{13}\hat{\sigma}^z_{16} + 838992\hat{\sigma}^z_{14}\hat{\sigma}^z_{15} - 224\hat{\sigma}^z_{0}\hat{\sigma}^z_{30} - 224\hat{\sigma}^z_{1}\hat{\sigma}^z_{29} - 448\hat{\sigma}^z_{2}\hat{\sigma}^z_{28} - 672\hat{\sigma}^z_{3}\hat{\sigma}^z_{27} - 1677984\hat{\sigma}^z_{5}\hat{\sigma}^z_{25} -$
$672\hat{\sigma}^z_{7}\hat{\sigma}^z_{23} - 224\hat{\sigma}^z_{8}\hat{\sigma}^z_{22} - 672\hat{\sigma}^z_{9}\hat{\sigma}^z_{21} - 672\hat{\sigma}^z_{10}\hat{\sigma}^z_{20} - 672\hat{\sigma}^z_{11}\hat{\sigma}^z_{19} - 224\hat{\sigma}^z_{12}\hat{\sigma}^z_{18} - 224\hat{\sigma}^z_{13}\hat{\sigma}^z_{17} - 448\hat{\sigma}^z_{14}\hat{\sigma}^z_{16} -$
$448\hat{\sigma}^z_{0}\hat{\sigma}^z_{31} - 448\hat{\sigma}^z_{1}\hat{\sigma}^z_{30} - 448\hat{\sigma}^z_{2}\hat{\sigma}^z_{29} - 224\hat{\sigma}^z_{3}\hat{\sigma}^z_{28} - 1677984\hat{\sigma}^z_{5}\hat{\sigma}^z_{26} - 1677984\hat{\sigma}^z_{6}\hat{\sigma}^z_{25} - 1677984\hat{\sigma}^z_{7}\hat{\sigma}^z_{24} -$
$224\hat{\sigma}^z_{8}\hat{\sigma}^z_{23} - 448\hat{\sigma}^z_{9}\hat{\sigma}^z_{22} - 896\hat{\sigma}^z_{10}\hat{\sigma}^z_{21} - 896\hat{\sigma}^z_{11}\hat{\sigma}^z_{20} - 448\hat{\sigma}^z_{12}\hat{\sigma}^z_{19} - 448\hat{\sigma}^z_{13}\hat{\sigma}^z_{18} - 448\hat{\sigma}^z_{14}\hat{\sigma}^z_{17} - 224\hat{\sigma}^z_{15}\hat{\sigma}^z_{16} -$
$448\hat{\sigma}^z_{0}\hat{\sigma}^z_{32} - 896\hat{\sigma}^z_{1}\hat{\sigma}^z_{31} - 224\hat{\sigma}^z_{2}\hat{\sigma}^z_{30} - 448\hat{\sigma}^z_{3}\hat{\sigma}^z_{29} - 224\hat{\sigma}^z_{4}\hat{\sigma}^z_{28} - 672\hat{\sigma}^z_{7}\hat{\sigma}^z_{25} - 224\hat{\sigma}^z_{8}\hat{\sigma}^z_{24} - 224\hat{\sigma}^z_{9}\hat{\sigma}^z_{23} - 448\hat{\sigma}^z_{10}\hat{\sigma}^z_{22} -$
$672\hat{\sigma}^z_{11}\hat{\sigma}^z_{21} - 448\hat{\sigma}^z_{12}\hat{\sigma}^z_{20} - 896\hat{\sigma}^z_{13}\hat{\sigma}^z_{19} - 224\hat{\sigma}^z_{14}\hat{\sigma}^z_{18} - 448\hat{\sigma}^z_{15}\hat{\sigma}^z_{17} - 448\hat{\sigma}^z_{0}\hat{\sigma}^z_{33} - 672\hat{\sigma}^z_{1}\hat{\sigma}^z_{32} - 672\hat{\sigma}^z_{2}\hat{\sigma}^z_{31} -$
$448\hat{\sigma}^z_{3}\hat{\sigma}^z_{30} - 224\hat{\sigma}^z_{4}\hat{\sigma}^z_{29} - 448\hat{\sigma}^z_{5}\hat{\sigma}^z_{28} - 1677984\hat{\sigma}^z_{6}\hat{\sigma}^z_{27} - 1677984\hat{\sigma}^z_{7}\hat{\sigma}^z_{26} - 448\hat{\sigma}^z_{8}\hat{\sigma}^z_{25} - 448\hat{\sigma}^z_{9}\hat{\sigma}^z_{24} - 448\hat{\sigma}^z_{10}\hat{\sigma}^z_{23} -$
$224\hat{\sigma}^z_{11}\hat{\sigma}^z_{22} - 448\hat{\sigma}^z_{12}\hat{\sigma}^z_{21} - 672\hat{\sigma}^z_{13}\hat{\sigma}^z_{20} - 672\hat{\sigma}^z_{14}\hat{\sigma}^z_{19} - 448\hat{\sigma}^z_{15}\hat{\sigma}^z_{18} - 224\hat{\sigma}^z_{0}\hat{\sigma}^z_{34} - 672\hat{\sigma}^z_{1}\hat{\sigma}^z_{33} - 672\hat{\sigma}^z_{2}\hat{\sigma}^z_{32} -$
$672\hat{\sigma}^z_{3}\hat{\sigma}^z_{31} - 224\hat{\sigma}^z_{4}\hat{\sigma}^z_{30} - 224\hat{\sigma}^z_{5}\hat{\sigma}^z_{29} - 448\hat{\sigma}^z_{6}\hat{\sigma}^z_{28} - 1677984\hat{\sigma}^z_{7}\hat{\sigma}^z_{27} - 448\hat{\sigma}^z_{8}\hat{\sigma}^z_{26} - 896\hat{\sigma}^z_{9}\hat{\sigma}^z_{25} - 224\hat{\sigma}^z_{10}\hat{\sigma}^z_{24} -$
$448\hat{\sigma}^z_{11}\hat{\sigma}^z_{23} - 224\hat{\sigma}^z_{12}\hat{\sigma}^z_{22} - 672\hat{\sigma}^z_{13}\hat{\sigma}^z_{21} - 672\hat{\sigma}^z_{14}\hat{\sigma}^z_{20} - 672\hat{\sigma}^z_{15}\hat{\sigma}^z_{19} - 224\hat{\sigma}^z_{0}\hat{\sigma}^z_{35} - 448\hat{\sigma}^z_{1}\hat{\sigma}^z_{34} - 896\hat{\sigma}^z_{2}\hat{\sigma}^z_{33} - 896\hat{\sigma}^z_{3}\hat{\sigma}^z_{32} -$
$448\hat{\sigma}^z_{4}\hat{\sigma}^z_{31} - 448\hat{\sigma}^z_{5}\hat{\sigma}^z_{30} - 448\hat{\sigma}^z_{6}\hat{\sigma}^z_{29} - 224\hat{\sigma}^z_{7}\hat{\sigma}^z_{28} - 448\hat{\sigma}^z_{8}\hat{\sigma}^z_{27} - 672\hat{\sigma}^z_{9}\hat{\sigma}^z_{26} - 672\hat{\sigma}^z_{10}\hat{\sigma}^z_{25} - 448\hat{\sigma}^z_{11}\hat{\sigma}^z_{24} - 224\hat{\sigma}^z_{12}\hat{\sigma}^z_{23} -$
$448\hat{\sigma}^z_{13}\hat{\sigma}^z_{22} - 896\hat{\sigma}^z_{14}\hat{\sigma}^z_{21} - 896\hat{\sigma}^z_{15}\hat{\sigma}^z_{20} - 224\hat{\sigma}^z_{0}\hat{\sigma}^z_{36} - 224\hat{\sigma}^z_{1}\hat{\sigma}^z_{35} - 448\hat{\sigma}^z_{2}\hat{\sigma}^z_{34} - 672\hat{\sigma}^z_{3}\hat{\sigma}^z_{33} - 448\hat{\sigma}^z_{4}\hat{\sigma}^z_{32} - 896\hat{\sigma}^z_{5}\hat{\sigma}^z_{31} -$
$224\hat{\sigma}^z_{6}\hat{\sigma}^z_{30} - 448\hat{\sigma}^z_{7}\hat{\sigma}^z_{29} - 1677984\hat{\sigma}^z_{8}\hat{\sigma}^z_{28} - 672\hat{\sigma}^z_{9}\hat{\sigma}^z_{27} - 672\hat{\sigma}^z_{10}\hat{\sigma}^z_{26} - 672\hat{\sigma}^z_{11}\hat{\sigma}^z_{25} - 224\hat{\sigma}^z_{12}\hat{\sigma}^z_{24} - 224\hat{\sigma}^z_{13}\hat{\sigma}^z_{23} -$
$448\hat{\sigma}^z_{14}\hat{\sigma}^z_{22} - 672\hat{\sigma}^z_{15}\hat{\sigma}^z_{21} - 448\hat{\sigma}^z_{0}\hat{\sigma}^z_{37} - 448\hat{\sigma}^z_{1}\hat{\sigma}^z_{36} - 448\hat{\sigma}^z_{2}\hat{\sigma}^z_{35} - 224\hat{\sigma}^z_{3}\hat{\sigma}^z_{34} - 448\hat{\sigma}^z_{4}\hat{\sigma}^z_{33} - 672\hat{\sigma}^z_{5}\hat{\sigma}^z_{32} - 672\hat{\sigma}^z_{6}\hat{\sigma}^z_{31} -$
$448\hat{\sigma}^z_{7}\hat{\sigma}^z_{30} - 1677984\hat{\sigma}^z_{8}\hat{\sigma}^z_{29} - 1677984\hat{\sigma}^z_{9}\hat{\sigma}^z_{28} - 896\hat{\sigma}^z_{10}\hat{\sigma}^z_{27} - 896\hat{\sigma}^z_{11}\hat{\sigma}^z_{26} - 448\hat{\sigma}^z_{12}\hat{\sigma}^z_{25} - 448\hat{\sigma}^z_{13}\hat{\sigma}^z_{24} - 448\hat{\sigma}^z_{14}\hat{\sigma}^z_{23} -$
$224\hat{\sigma}^z_{15}\hat{\sigma}^z_{22} + 1344\hat{\sigma}^z_{16}\hat{\sigma}^z_{21} - 448\hat{\sigma}^z_{0}\hat{\sigma}^z_{38} - 896\hat{\sigma}^z_{1}\hat{\sigma}^z_{37} - 224\hat{\sigma}^z_{2}\hat{\sigma}^z_{36} - 448\hat{\sigma}^z_{3}\hat{\sigma}^z_{35} - 224\hat{\sigma}^z_{4}\hat{\sigma}^z_{34} - 672\hat{\sigma}^z_{5}\hat{\sigma}^z_{33} - 672\hat{\sigma}^z_{6}\hat{\sigma}^z_{32} -$
$672\hat{\sigma}^z_{7}\hat{\sigma}^z_{31} - 1677984\hat{\sigma}^z_{8}\hat{\sigma}^z_{30} - 672\hat{\sigma}^z_{10}\hat{\sigma}^z_{28} - 672\hat{\sigma}^z_{11}\hat{\sigma}^z_{27} - 448\hat{\sigma}^z_{12}\hat{\sigma}^z_{26} - 896\hat{\sigma}^z_{13}\hat{\sigma}^z_{25} - 224\hat{\sigma}^z_{14}\hat{\sigma}^z_{24} - 448\hat{\sigma}^z_{15}\hat{\sigma}^z_{23} +$
$448\hat{\sigma}^z_{16}\hat{\sigma}^z_{22} - 448\hat{\sigma}^z_{0}\hat{\sigma}^z_{39} - 672\hat{\sigma}^z_{1}\hat{\sigma}^z_{38} - 672\hat{\sigma}^z_{2}\hat{\sigma}^z_{37} - 448\hat{\sigma}^z_{3}\hat{\sigma}^z_{36} - 224\hat{\sigma}^z_{4}\hat{\sigma}^z_{35} - 448\hat{\sigma}^z_{5}\hat{\sigma}^z_{34} - 896\hat{\sigma}^z_{6}\hat{\sigma}^z_{33} - 896\hat{\sigma}^z_{7}\hat{\sigma}^z_{32} -$
$1677984\hat{\sigma}^z_{10}\hat{\sigma}^z_{29} - 672\hat{\sigma}^z_{11}\hat{\sigma}^z_{28} - 448\hat{\sigma}^z_{12}\hat{\sigma}^z_{27} - 672\hat{\sigma}^z_{13}\hat{\sigma}^z_{26} - 672\hat{\sigma}^z_{14}\hat{\sigma}^z_{25} - 448\hat{\sigma}^z_{15}\hat{\sigma}^z_{24} - 672\hat{\sigma}^z_{1}\hat{\sigma}^z_{39} - 672\hat{\sigma}^z_{2}\hat{\sigma}^z_{38} -$
$672\hat{\sigma}^z_{3}\hat{\sigma}^z_{37} - 224\hat{\sigma}^z_{4}\hat{\sigma}^z_{36} - 224\hat{\sigma}^z_{5}\hat{\sigma}^z_{35} - 448\hat{\sigma}^z_{6}\hat{\sigma}^z_{34} - 672\hat{\sigma}^z_{7}\hat{\sigma}^z_{33} - 1677984\hat{\sigma}^z_{9}\hat{\sigma}^z_{31} - 672\hat{\sigma}^z_{11}\hat{\sigma}^z_{29} - 224\hat{\sigma}^z_{12}\hat{\sigma}^z_{28} -$
$672\hat{\sigma}^z_{13}\hat{\sigma}^z_{27} - 672\hat{\sigma}^z_{14}\hat{\sigma}^z_{26} - 672\hat{\sigma}^z_{15}\hat{\sigma}^z_{25} + 448\hat{\sigma}^z_{17}\hat{\sigma}^z_{23} - 896\hat{\sigma}^z_{2}\hat{\sigma}^z_{39} - 896\hat{\sigma}^z_{3}\hat{\sigma}^z_{38} - 448\hat{\sigma}^z_{4}\hat{\sigma}^z_{37} - 448\hat{\sigma}^z_{5}\hat{\sigma}^z_{36} - 448\hat{\sigma}^z_{6}\hat{\sigma}^z_{35} -$
$224\hat{\sigma}^z_{7}\hat{\sigma}^z_{34} - 1677984\hat{\sigma}^z_{9}\hat{\sigma}^z_{32} - 1677984\hat{\sigma}^z_{10}\hat{\sigma}^z_{31} - 1677984\hat{\sigma}^z_{11}\hat{\sigma}^z_{30} - 224\hat{\sigma}^z_{12}\hat{\sigma}^z_{29} - 448\hat{\sigma}^z_{13}\hat{\sigma}^z_{28} - 896\hat{\sigma}^z_{14}\hat{\sigma}^z_{27} -$

$$896\hat{\sigma}^z_{15}\hat{\sigma}^z_{26}+448\hat{\sigma}^z_{16}\hat{\sigma}^z_{25}+448\hat{\sigma}^z_{19}\hat{\sigma}^z_{22}-672\hat{\sigma}^z_{3}\hat{\sigma}^z_{39}-448\hat{\sigma}^z_{4}\hat{\sigma}^z_{38}-896\hat{\sigma}^z_{5}\hat{\sigma}^z_{37}-224\hat{\sigma}^z_{6}\hat{\sigma}^z_{36}-448\hat{\sigma}^z_{7}\hat{\sigma}^z_{35}-224\hat{\sigma}^z_{8}\hat{\sigma}^z_{34}-$$
$$672\hat{\sigma}^z_{11}\hat{\sigma}^z_{31}-224\hat{\sigma}^z_{12}\hat{\sigma}^z_{30}-224\hat{\sigma}^z_{13}\hat{\sigma}^z_{29}-448\hat{\sigma}^z_{14}\hat{\sigma}^z_{28}-672\hat{\sigma}^z_{15}\hat{\sigma}^z_{27}+448\hat{\sigma}^z_{18}\hat{\sigma}^z_{24}-448\hat{\sigma}^z_{4}\hat{\sigma}^z_{39}-672\hat{\sigma}^z_{5}\hat{\sigma}^z_{38}-672\hat{\sigma}^z_{6}\hat{\sigma}^z_{37}-$$
$$448\hat{\sigma}^z_{7}\hat{\sigma}^z_{36}-224\hat{\sigma}^z_{8}\hat{\sigma}^z_{35}-448\hat{\sigma}^z_{9}\hat{\sigma}^z_{34}-1677984\hat{\sigma}^z_{10}\hat{\sigma}^z_{33}-1677984\hat{\sigma}^z_{11}\hat{\sigma}^z_{32}-448\hat{\sigma}^z_{12}\hat{\sigma}^z_{31}-448\hat{\sigma}^z_{13}\hat{\sigma}^z_{30}-448\hat{\sigma}^z_{14}\hat{\sigma}^z_{29}-$$
$$224\hat{\sigma}^z_{15}\hat{\sigma}^z_{28}+448\hat{\sigma}^z_{16}\hat{\sigma}^z_{27}+448\hat{\sigma}^z_{17}\hat{\sigma}^z_{26}+448\hat{\sigma}^z_{18}\hat{\sigma}^z_{25}+448\hat{\sigma}^z_{19}\hat{\sigma}^z_{24}+448\hat{\sigma}^z_{20}\hat{\sigma}^z_{23}+448\hat{\sigma}^z_{21}\hat{\sigma}^z_{22}-672\hat{\sigma}^z_{5}\hat{\sigma}^z_{39}-672\hat{\sigma}^z_{6}\hat{\sigma}^z_{38}-$$
$$672\hat{\sigma}^z_{7}\hat{\sigma}^z_{37}-224\hat{\sigma}^z_{8}\hat{\sigma}^z_{36}-224\hat{\sigma}^z_{9}\hat{\sigma}^z_{35}-448\hat{\sigma}^z_{10}\hat{\sigma}^z_{34}-1677984\hat{\sigma}^z_{11}\hat{\sigma}^z_{33}-448\hat{\sigma}^z_{12}\hat{\sigma}^z_{32}-896\hat{\sigma}^z_{13}\hat{\sigma}^z_{31}-224\hat{\sigma}^z_{14}\hat{\sigma}^z_{30}-$$
$$448\hat{\sigma}^z_{15}\hat{\sigma}^z_{29}+448\hat{\sigma}^z_{16}\hat{\sigma}^z_{28}+448\hat{\sigma}^z_{17}\hat{\sigma}^z_{27}+448\hat{\sigma}^z_{18}\hat{\sigma}^z_{26}+896\hat{\sigma}^z_{19}\hat{\sigma}^z_{25}+448\hat{\sigma}^z_{20}\hat{\sigma}^z_{24}+448\hat{\sigma}^z_{21}\hat{\sigma}^z_{23}-896\hat{\sigma}^z_{6}\hat{\sigma}^z_{39}-896\hat{\sigma}^z_{7}\hat{\sigma}^z_{38}-$$
$$448\hat{\sigma}^z_{8}\hat{\sigma}^z_{37}-448\hat{\sigma}^z_{9}\hat{\sigma}^z_{36}-448\hat{\sigma}^z_{10}\hat{\sigma}^z_{35}-224\hat{\sigma}^z_{11}\hat{\sigma}^z_{34}-448\hat{\sigma}^z_{12}\hat{\sigma}^z_{33}-672\hat{\sigma}^z_{13}\hat{\sigma}^z_{32}-672\hat{\sigma}^z_{14}\hat{\sigma}^z_{31}-448\hat{\sigma}^z_{15}\hat{\sigma}^z_{30}+$$
$$448\hat{\sigma}^z_{19}\hat{\sigma}^z_{26}+448\hat{\sigma}^z_{20}\hat{\sigma}^z_{25}-672\hat{\sigma}^z_{7}\hat{\sigma}^z_{39}-448\hat{\sigma}^z_{8}\hat{\sigma}^z_{38}-896\hat{\sigma}^z_{9}\hat{\sigma}^z_{37}-224\hat{\sigma}^z_{10}\hat{\sigma}^z_{36}-448\hat{\sigma}^z_{11}\hat{\sigma}^z_{35}-1677984\hat{\sigma}^z_{12}\hat{\sigma}^z_{34}-$$
$$672\hat{\sigma}^z_{13}\hat{\sigma}^z_{33}-672\hat{\sigma}^z_{14}\hat{\sigma}^z_{32}-672\hat{\sigma}^z_{15}\hat{\sigma}^z_{31}+448\hat{\sigma}^z_{17}\hat{\sigma}^z_{29}+448\hat{\sigma}^z_{19}\hat{\sigma}^z_{27}+896\hat{\sigma}^z_{20}\hat{\sigma}^z_{26}+448\hat{\sigma}^z_{21}\hat{\sigma}^z_{25}-448\hat{\sigma}^z_{8}\hat{\sigma}^z_{39}-$$
$$672\hat{\sigma}^z_{9}\hat{\sigma}^z_{38}-672\hat{\sigma}^z_{10}\hat{\sigma}^z_{37}-448\hat{\sigma}^z_{11}\hat{\sigma}^z_{36}-1677984\hat{\sigma}^z_{12}\hat{\sigma}^z_{35}-1677984\hat{\sigma}^z_{13}\hat{\sigma}^z_{34}-896\hat{\sigma}^z_{14}\hat{\sigma}^z_{33}-896\hat{\sigma}^z_{15}\hat{\sigma}^z_{32}+448\hat{\sigma}^z_{16}\hat{\sigma}^z_{31}+$$
$$448\hat{\sigma}^z_{19}\hat{\sigma}^z_{28}+448\hat{\sigma}^z_{20}\hat{\sigma}^z_{27}+448\hat{\sigma}^z_{21}\hat{\sigma}^z_{26}-672\hat{\sigma}^z_{9}\hat{\sigma}^z_{39}-672\hat{\sigma}^z_{10}\hat{\sigma}^z_{38}-672\hat{\sigma}^z_{11}\hat{\sigma}^z_{37}-1677984\hat{\sigma}^z_{12}\hat{\sigma}^z_{36}-672\hat{\sigma}^z_{14}\hat{\sigma}^z_{34}-$$
$$672\hat{\sigma}^z_{15}\hat{\sigma}^z_{33}+448\hat{\sigma}^z_{18}\hat{\sigma}^z_{30}+896\hat{\sigma}^z_{21}\hat{\sigma}^z_{27}-896\hat{\sigma}^z_{10}\hat{\sigma}^z_{39}-896\hat{\sigma}^z_{11}\hat{\sigma}^z_{38}-1677984\hat{\sigma}^z_{14}\hat{\sigma}^z_{35}-672\hat{\sigma}^z_{15}\hat{\sigma}^z_{34}+448\hat{\sigma}^z_{16}\hat{\sigma}^z_{33}+$$
$$448\hat{\sigma}^z_{17}\hat{\sigma}^z_{32}+448\hat{\sigma}^z_{18}\hat{\sigma}^z_{31}+448\hat{\sigma}^z_{19}\hat{\sigma}^z_{30}+448\hat{\sigma}^z_{20}\hat{\sigma}^z_{29}+448\hat{\sigma}^z_{21}\hat{\sigma}^z_{28}+1344\hat{\sigma}^z_{22}\hat{\sigma}^z_{27}-672\hat{\sigma}^z_{11}\hat{\sigma}^z_{39}-1677984\hat{\sigma}^z_{13}\hat{\sigma}^z_{37}-$$
$$672\hat{\sigma}^z_{15}\hat{\sigma}^z_{35}+448\hat{\sigma}^z_{16}\hat{\sigma}^z_{34}+448\hat{\sigma}^z_{17}\hat{\sigma}^z_{33}+448\hat{\sigma}^z_{18}\hat{\sigma}^z_{32}+896\hat{\sigma}^z_{19}\hat{\sigma}^z_{31}+448\hat{\sigma}^z_{20}\hat{\sigma}^z_{30}+448\hat{\sigma}^z_{21}\hat{\sigma}^z_{29}+448\hat{\sigma}^z_{22}\hat{\sigma}^z_{28}-$$
$$1677984\hat{\sigma}^z_{13}\hat{\sigma}^z_{38}-1677984\hat{\sigma}^z_{14}\hat{\sigma}^z_{37}-1677984\hat{\sigma}^z_{15}\hat{\sigma}^z_{36}+448\hat{\sigma}^z_{19}\hat{\sigma}^z_{32}+448\hat{\sigma}^z_{20}\hat{\sigma}^z_{31}-672\hat{\sigma}^z_{15}\hat{\sigma}^z_{37}+448\hat{\sigma}^z_{17}\hat{\sigma}^z_{35}+$$
$$448\hat{\sigma}^z_{19}\hat{\sigma}^z_{33}+896\hat{\sigma}^z_{20}\hat{\sigma}^z_{32}+448\hat{\sigma}^z_{21}\hat{\sigma}^z_{31}+448\hat{\sigma}^z_{23}\hat{\sigma}^z_{29}-1677984\hat{\sigma}^z_{14}\hat{\sigma}^z_{39}-1677984\hat{\sigma}^z_{15}\hat{\sigma}^z_{38}+448\hat{\sigma}^z_{16}\hat{\sigma}^z_{37}+448\hat{\sigma}^z_{19}\hat{\sigma}^z_{34}+$$
$$448\hat{\sigma}^z_{20}\hat{\sigma}^z_{33}+448\hat{\sigma}^z_{21}\hat{\sigma}^z_{32}+448\hat{\sigma}^z_{22}\hat{\sigma}^z_{31}+448\hat{\sigma}^z_{25}\hat{\sigma}^z_{28}-1677984\hat{\sigma}^z_{15}\hat{\sigma}^z_{39}+448\hat{\sigma}^z_{18}\hat{\sigma}^z_{36}+896\hat{\sigma}^z_{21}\hat{\sigma}^z_{33}+448\hat{\sigma}^z_{24}\hat{\sigma}^z_{30}+$$
$$448\hat{\sigma}^z_{16}\hat{\sigma}^z_{39}+448\hat{\sigma}^z_{17}\hat{\sigma}^z_{38}+448\hat{\sigma}^z_{18}\hat{\sigma}^z_{37}+448\hat{\sigma}^z_{19}\hat{\sigma}^z_{36}+448\hat{\sigma}^z_{20}\hat{\sigma}^z_{35}+448\hat{\sigma}^z_{21}\hat{\sigma}^z_{34}+448\hat{\sigma}^z_{22}\hat{\sigma}^z_{33}+448\hat{\sigma}^z_{23}\hat{\sigma}^z_{32}+$$
$$448\hat{\sigma}^z_{24}\hat{\sigma}^z_{31}+448\hat{\sigma}^z_{25}\hat{\sigma}^z_{30}+448\hat{\sigma}^z_{26}\hat{\sigma}^z_{29}+448\hat{\sigma}^z_{27}\hat{\sigma}^z_{28}+448\hat{\sigma}^z_{17}\hat{\sigma}^z_{39}+448\hat{\sigma}^z_{18}\hat{\sigma}^z_{38}+896\hat{\sigma}^z_{19}\hat{\sigma}^z_{37}+448\hat{\sigma}^z_{20}\hat{\sigma}^z_{36}+$$
$$448\hat{\sigma}^z_{21}\hat{\sigma}^z_{35}+448\hat{\sigma}^z_{22}\hat{\sigma}^z_{34}+448\hat{\sigma}^z_{23}\hat{\sigma}^z_{33}+448\hat{\sigma}^z_{24}\hat{\sigma}^z_{32}+896\hat{\sigma}^z_{25}\hat{\sigma}^z_{31}+448\hat{\sigma}^z_{26}\hat{\sigma}^z_{30}+448\hat{\sigma}^z_{27}\hat{\sigma}^z_{29}+448\hat{\sigma}^z_{19}\hat{\sigma}^z_{38}+$$
$$448\hat{\sigma}^z_{20}\hat{\sigma}^z_{37}+448\hat{\sigma}^z_{25}\hat{\sigma}^z_{32}+448\hat{\sigma}^z_{26}\hat{\sigma}^z_{31}+448\hat{\sigma}^z_{19}\hat{\sigma}^z_{39}+896\hat{\sigma}^z_{20}\hat{\sigma}^z_{38}+448\hat{\sigma}^z_{21}\hat{\sigma}^z_{37}+448\hat{\sigma}^z_{23}\hat{\sigma}^z_{35}+448\hat{\sigma}^z_{25}\hat{\sigma}^z_{33}+$$
$$896\hat{\sigma}^z_{26}\hat{\sigma}^z_{32}+448\hat{\sigma}^z_{27}\hat{\sigma}^z_{31}+448\hat{\sigma}^z_{20}\hat{\sigma}^z_{39}+448\hat{\sigma}^z_{21}\hat{\sigma}^z_{38}+448\hat{\sigma}^z_{22}\hat{\sigma}^z_{37}+448\hat{\sigma}^z_{25}\hat{\sigma}^z_{34}+448\hat{\sigma}^z_{26}\hat{\sigma}^z_{33}+448\hat{\sigma}^z_{27}\hat{\sigma}^z_{32}+$$
$$896\hat{\sigma}^z_{21}\hat{\sigma}^z_{39}+448\hat{\sigma}^z_{24}\hat{\sigma}^z_{36}+896\hat{\sigma}^z_{27}\hat{\sigma}^z_{33}+448\hat{\sigma}^z_{22}\hat{\sigma}^z_{39}+448\hat{\sigma}^z_{23}\hat{\sigma}^z_{38}+448\hat{\sigma}^z_{24}\hat{\sigma}^z_{37}+448\hat{\sigma}^z_{25}\hat{\sigma}^z_{36}+448\hat{\sigma}^z_{26}\hat{\sigma}^z_{35}+$$
$$448\hat{\sigma}^z_{27}\hat{\sigma}^z_{34}+1344\hat{\sigma}^z_{28}\hat{\sigma}^z_{33}+448\hat{\sigma}^z_{23}\hat{\sigma}^z_{39}+448\hat{\sigma}^z_{24}\hat{\sigma}^z_{38}+896\hat{\sigma}^z_{25}\hat{\sigma}^z_{37}+448\hat{\sigma}^z_{26}\hat{\sigma}^z_{36}+448\hat{\sigma}^z_{27}\hat{\sigma}^z_{35}+448\hat{\sigma}^z_{28}\hat{\sigma}^z_{34}+$$
$$448\hat{\sigma}^z_{25}\hat{\sigma}^z_{38}+448\hat{\sigma}^z_{26}\hat{\sigma}^z_{37}+448\hat{\sigma}^z_{25}\hat{\sigma}^z_{39}+896\hat{\sigma}^z_{26}\hat{\sigma}^z_{38}+448\hat{\sigma}^z_{27}\hat{\sigma}^z_{37}+448\hat{\sigma}^z_{29}\hat{\sigma}^z_{35}+448\hat{\sigma}^z_{26}\hat{\sigma}^z_{39}+448\hat{\sigma}^z_{27}\hat{\sigma}^z_{38}+$$
$$448\hat{\sigma}^z_{28}\hat{\sigma}^z_{37}+448\hat{\sigma}^z_{31}\hat{\sigma}^z_{34}+896\hat{\sigma}^z_{27}\hat{\sigma}^z_{39}+448\hat{\sigma}^z_{30}\hat{\sigma}^z_{36}+448\hat{\sigma}^z_{28}\hat{\sigma}^z_{39}+448\hat{\sigma}^z_{29}\hat{\sigma}^z_{38}+448\hat{\sigma}^z_{30}\hat{\sigma}^z_{37}+448\hat{\sigma}^z_{31}\hat{\sigma}^z_{36}+$$
$$448\hat{\sigma}^z_{32}\hat{\sigma}^z_{35}+448\hat{\sigma}^z_{33}\hat{\sigma}^z_{34}+448\hat{\sigma}^z_{29}\hat{\sigma}^z_{39}+448\hat{\sigma}^z_{30}\hat{\sigma}^z_{38}+896\hat{\sigma}^z_{31}\hat{\sigma}^z_{37}+448\hat{\sigma}^z_{32}\hat{\sigma}^z_{36}+448\hat{\sigma}^z_{33}\hat{\sigma}^z_{35}+448\hat{\sigma}^z_{31}\hat{\sigma}^z_{38}+$$
$$448\hat{\sigma}^z_{32}\hat{\sigma}^z_{37}+448\hat{\sigma}^z_{31}\hat{\sigma}^z_{39}+896\hat{\sigma}^z_{32}\hat{\sigma}^z_{38}+448\hat{\sigma}^z_{33}\hat{\sigma}^z_{37}+448\hat{\sigma}^z_{32}\hat{\sigma}^z_{39}+448\hat{\sigma}^z_{33}\hat{\sigma}^z_{38}+896\hat{\sigma}^z_{33}\hat{\sigma}^z_{39}+1344\hat{\sigma}^z_{34}\hat{\sigma}^z_{39}+60445728$$

A complete expression of $E_k(s)$ for 36-order Williamson based method or 108-order Baumert-Hall based method

$E_k(s) = 432s_0s_1+432s_0s_2+288s_0s_3+864s_1s_2+432s_0s_4+720s_1s_3+864s_1s_4+720s_2s_3+864s_2s_4+720s_3s_4+$
$432s_5s_6+432s_5s_7+288s_5s_8+864s_6s_7+432s_5s_9+720s_6s_8+864s_6s_9+720s_7s_8+864s_7s_9+720s_8s_9+432s_{10}s_{11}+$
$432s_{10}s_{12}+288s_{10}s_{13}+864s_{11}s_{12}+432s_{10}s_{14}+720s_{11}s_{13}+864s_{11}s_{14}+720s_{12}s_{13}+864s_{12}s_{14}+720s_{13}s_{14}+$
$432s_{15}s_{16}+432s_{15}s_{17}+288s_{15}s_{18}+864s_{16}s_{17}+432s_{15}s_{19}+720s_{16}s_{18}+864s_{16}s_{19}+720s_{17}s_{18}+864s_{17}s_{19}+$
$720s_{18}s_{19}+432s_0s_1s_2s_3+432s_0s_1s_3s_4+432s_0s_2s_3s_4+432s_1s_2s_3s_4+144s_0s_1s_5s_6+144s_0s_1s_6s_7+144s_0s_2s_5s_7+$
$144s_1s_2s_5s_6+144s_0s_1s_7s_8+144s_0s_2s_6s_8+144s_0s_3s_5s_8+144s_0s_3s_6s_7+144s_1s_2s_5s_8+288s_1s_2s_6s_7+144s_1s_3s_5s_7+$
$144s_2s_3s_5s_6+144s_0s_1s_8s_9+144s_0s_2s_7s_9+144s_0s_3s_6s_9+144s_0s_4s_5s_9+144s_0s_4s_6s_8+144s_1s_2s_6s_9+144s_1s_2s_7s_8+$
$144s_1s_3s_5s_9+288s_1s_3s_6s_8+144s_1s_4s_5s_8+144s_1s_4s_6s_7+144s_2s_3s_6s_7+144s_2s_4s_5s_7+144s_3s_4s_5s_6+144s_0s_2s_8s_9+$
$144s_0s_3s_7s_9+144s_0s_4s_6s_9+144s_0s_4s_7s_8+144s_1s_2s_7s_9+144s_1s_3s_6s_9+144s_1s_3s_7s_8+144s_1s_4s_5s_9+144s_1s_4s_6s_8+$
$144s_2s_3s_5s_9+144s_2s_3s_6s_8+144s_2s_4s_5s_8+144s_2s_4s_6s_7+144s_3s_4s_5s_7+144s_1s_2s_8s_9+144s_1s_3s_7s_9+288s_1s_4s_6s_9+$
$144s_1s_4s_7s_8+144s_2s_3s_6s_9+288s_2s_3s_7s_8+144s_2s_4s_6s_8+144s_3s_4s_6s_7+144s_1s_3s_8s_9+144s_1s_4s_7s_9+144s_2s_4s_6s_9+$
$144s_3s_4s_6s_8+144s_0s_1s_{10}s_{11}+144s_2s_3s_8s_9+288s_2s_4s_7s_9+144s_3s_4s_7s_8+144s_2s_4s_8s_9+144s_3s_4s_7s_9+$
$144s_0s_1s_{11}s_{12}+144s_0s_2s_{10}s_{12}+144s_1s_2s_{10}s_{11}+288s_3s_4s_8s_9+144s_0s_1s_{12}s_{13}+144s_0s_2s_{11}s_{13}+144s_0s_3s_{10}s_{13}+$
$144s_0s_3s_{11}s_{12}+144s_1s_2s_{10}s_{13}+288s_1s_2s_{11}s_{12}+144s_1s_3s_{10}s_{12}+144s_2s_3s_{10}s_{11}+432s_5s_6s_7s_8+144s_0s_1s_{13}s_{14}+$
$144s_0s_2s_{12}s_{14}+144s_0s_3s_{11}s_{14}+144s_0s_4s_{10}s_{14}+144s_0s_4s_{11}s_{13}+144s_1s_2s_{11}s_{14}+144s_1s_2s_{12}s_{13}+144s_1s_3s_{10}s_{14}+$
$288s_1s_3s_{11}s_{13}+144s_1s_4s_{10}s_{13}+144s_1s_4s_{11}s_{12}+144s_2s_3s_{11}s_{12}+144s_2s_4s_{10}s_{12}+144s_3s_4s_{10}s_{11}+432s_5s_6s_8s_9+$
$144s_0s_2s_{13}s_{14}+144s_0s_3s_{12}s_{14}+144s_0s_4s_{11}s_{14}+144s_0s_4s_{12}s_{13}+144s_1s_2s_{12}s_{14}+144s_1s_3s_{11}s_{14}+144s_1s_3s_{12}s_{13}+$
$144s_1s_4s_{10}s_{14}+144s_1s_4s_{11}s_{13}+144s_2s_3s_{10}s_{14}+144s_2s_3s_{11}s_{13}+144s_2s_4s_{10}s_{13}+144s_2s_4s_{11}s_{12}+144s_3s_4s_{10}s_{12}+$
$432s_5s_7s_8s_9+144s_1s_2s_{13}s_{14}+144s_1s_3s_{12}s_{14}+288s_1s_4s_{11}s_{14}+144s_1s_4s_{12}s_{13}+144s_2s_3s_{11}s_{14}+288s_2s_3s_{12}s_{13}+$
$144s_2s_4s_{11}s_{13}+144s_3s_4s_{11}s_{12}+432s_6s_7s_8s_9+144s_1s_3s_{13}s_{14}+144s_1s_4s_{12}s_{14}+144s_2s_4s_{11}s_{14}+144s_3s_4s_{11}s_{13}+$
$144s_0s_1s_{15}s_{16}+144s_2s_3s_{13}s_{14}+288s_2s_4s_{12}s_{14}+144s_3s_4s_{12}s_{13}+144s_5s_6s_{10}s_{11}+144s_2s_4s_{13}s_{14}+144s_3s_4s_{12}s_{14}+$
$144s_0s_1s_{16}s_{17}+144s_0s_2s_{15}s_{17}+144s_1s_2s_{15}s_{16}+288s_3s_4s_{13}s_{14}+144s_5s_6s_{11}s_{12}+144s_5s_7s_{10}s_{12}+144s_6s_7s_{10}s_{11}+$
$144s_0s_1s_{17}s_{18}+144s_0s_2s_{16}s_{18}+144s_0s_3s_{15}s_{18}+144s_0s_3s_{16}s_{17}+144s_1s_2s_{15}s_{18}+288s_1s_2s_{16}s_{17}+144s_1s_3s_{15}s_{17}+$
$144s_2s_3s_{15}s_{16}+144s_5s_6s_{12}s_{13}+144s_5s_7s_{11}s_{13}+144s_5s_8s_{10}s_{13}+144s_5s_8s_{11}s_{12}+144s_6s_7s_{10}s_{13}+288s_6s_7s_{11}s_{12}+$
$144s_6s_8s_{10}s_{12}+144s_7s_8s_{10}s_{11}+144s_0s_1s_{18}s_{19}+144s_0s_2s_{17}s_{19}+144s_0s_3s_{16}s_{19}+144s_0s_4s_{15}s_{19}+144s_0s_4s_{16}s_{18}+$
$144s_1s_2s_{16}s_{19}+144s_1s_2s_{17}s_{18}+144s_1s_3s_{15}s_{19}+288s_1s_3s_{16}s_{18}+144s_1s_4s_{15}s_{18}+144s_1s_4s_{16}s_{17}+144s_2s_3s_{16}s_{17}+$
$144s_2s_4s_{15}s_{17}+144s_3s_4s_{15}s_{16}+144s_5s_6s_{13}s_{14}+144s_5s_7s_{12}s_{14}+144s_5s_8s_{11}s_{14}+144s_5s_9s_{10}s_{14}+144s_5s_9s_{11}s_{13}+$
$144s_6s_7s_{11}s_{14}+144s_6s_7s_{12}s_{13}+144s_6s_8s_{10}s_{14}+288s_6s_8s_{11}s_{13}+144s_6s_9s_{10}s_{13}+144s_6s_9s_{11}s_{12}+144s_7s_8s_{11}s_{12}+$
$144s_7s_9s_{10}s_{12}+144s_8s_9s_{10}s_{11}+144s_0s_2s_{18}s_{19}+144s_0s_3s_{17}s_{19}+144s_0s_4s_{16}s_{19}+144s_0s_4s_{17}s_{18}+144s_1s_2s_{17}s_{19}+$
$144s_1s_3s_{16}s_{19}+144s_1s_3s_{17}s_{18}+144s_1s_4s_{15}s_{19}+144s_1s_4s_{16}s_{18}+144s_2s_3s_{15}s_{19}+144s_2s_3s_{16}s_{18}+144s_2s_4s_{15}s_{18}+$
$144s_2s_4s_{16}s_{17}+144s_3s_4s_{15}s_{17}+144s_5s_7s_{13}s_{14}+144s_5s_8s_{12}s_{14}+144s_5s_9s_{11}s_{14}+144s_5s_9s_{12}s_{13}+144s_6s_7s_{12}s_{14}+$
$144s_6s_8s_{11}s_{14}+144s_6s_8s_{12}s_{13}+144s_6s_9s_{10}s_{14}+144s_6s_9s_{11}s_{13}+144s_7s_8s_{10}s_{14}+144s_7s_8s_{11}s_{13}+144s_7s_9s_{10}s_{13}+$
$144s_7s_9s_{11}s_{12}+144s_8s_9s_{10}s_{12}+144s_1s_2s_{18}s_{19}+144s_1s_3s_{17}s_{19}+288s_1s_4s_{16}s_{19}+144s_1s_4s_{17}s_{18}+144s_2s_3s_{16}s_{19}+$
$288s_2s_3s_{17}s_{18}+144s_2s_4s_{16}s_{18}+144s_3s_4s_{16}s_{17}+144s_6s_7s_{13}s_{14}+144s_6s_8s_{12}s_{14}+288s_6s_9s_{11}s_{14}+144s_6s_9s_{12}s_{13}+$
$144s_7s_8s_{11}s_{14}+288s_7s_8s_{12}s_{13}+144s_7s_9s_{11}s_{13}+144s_8s_9s_{11}s_{12}+144s_1s_3s_{18}s_{19}+144s_1s_4s_{17}s_{19}+144s_2s_4s_{16}s_{19}+$
$144s_3s_4s_{16}s_{18}+144s_6s_8s_{13}s_{14}+144s_6s_9s_{12}s_{14}+144s_7s_9s_{11}s_{14}+144s_8s_9s_{11}s_{13}+144s_2s_3s_{18}s_{19}+288s_2s_4s_{17}s_{19}+$
$144s_3s_4s_{17}s_{18}+144s_5s_6s_{15}s_{16}+144s_7s_8s_{13}s_{14}+288s_7s_9s_{12}s_{14}+144s_8s_9s_{12}s_{13}+144s_2s_4s_{18}s_{19}+144s_3s_4s_{17}s_{19}+$
$144s_7s_9s_{13}s_{14}+144s_8s_9s_{12}s_{14}+288s_3s_4s_{18}s_{19}+144s_5s_6s_{16}s_{17}+144s_5s_7s_{15}s_{17}+144s_6s_7s_{15}s_{16}+288s_8s_9s_{13}s_{14}+$
$144s_5s_6s_{17}s_{18}+144s_5s_7s_{16}s_{18}+144s_5s_8s_{15}s_{18}+144s_5s_8s_{16}s_{17}+144s_6s_7s_{15}s_{18}+288s_6s_7s_{16}s_{17}+144s_6s_8s_{15}s_{17}+$
$144s_7s_8s_{15}s_{16}+432s_{10}s_{11}s_{12}s_{13}+144s_5s_6s_{18}s_{19}+144s_5s_7s_{17}s_{19}+144s_5s_8s_{16}s_{19}+144s_5s_9s_{15}s_{19}+144s_5s_9s_{16}s_{18}+$
$144s_6s_7s_{16}s_{19}+144s_6s_7s_{17}s_{18}+144s_6s_8s_{15}s_{19}+288s_6s_8s_{16}s_{18}+144s_6s_9s_{15}s_{18}+144s_6s_9s_{16}s_{17}+144s_7s_8s_{16}s_{17}+$
$144s_7s_9s_{15}s_{17}+144s_8s_9s_{15}s_{16}+432s_{10}s_{11}s_{13}s_{14}+144s_5s_7s_{18}s_{19}+144s_5s_8s_{17}s_{19}+144s_5s_9s_{16}s_{19}+144s_5s_9s_{17}s_{18}+$
$144s_6s_7s_{17}s_{19}+144s_6s_8s_{16}s_{19}+144s_6s_8s_{17}s_{18}+144s_6s_9s_{15}s_{19}+144s_6s_9s_{16}s_{18}+144s_7s_8s_{15}s_{19}+144s_7s_8s_{16}s_{18}+$
$144s_7s_9s_{15}s_{18}+144s_7s_9s_{16}s_{17}+144s_8s_9s_{15}s_{17}+432s_{10}s_{12}s_{13}s_{14}+144s_6s_7s_{18}s_{19}+144s_6s_8s_{17}s_{19}+288s_6s_9s_{16}s_{19}+$
$144s_6s_9s_{17}s_{18}+144s_7s_8s_{16}s_{19}+288s_7s_8s_{17}s_{18}+144s_7s_9s_{16}s_{18}+144s_8s_9s_{16}s_{17}+432s_{11}s_{12}s_{13}s_{14}+144s_6s_8s_{18}s_{19}+$

$144s_6s_9s_{17}s_{19}+144s_7s_9s_{16}s_{19}+144s_8s_9s_{16}s_{18}+144s_7s_8s_{18}s_{19}+288s_7s_9s_{17}s_{19}+144s_8s_9s_{17}s_{18}+144s_{10}s_{11}s_{15}s_{16}+144s_7s_9s_{18}s_{19}+144s_8s_9s_{17}s_{19}+288s_8s_9s_{18}s_{19}+144s_{10}s_{11}s_{16}s_{17}+144s_{10}s_{12}s_{15}s_{17}+144s_{11}s_{12}s_{15}s_{16}+144s_{10}s_{11}s_{17}s_{18}+144s_{10}s_{12}s_{16}s_{18}+144s_{10}s_{13}s_{15}s_{18}+144s_{10}s_{13}s_{16}s_{17}+144s_{11}s_{12}s_{15}s_{18}+288s_{11}s_{12}s_{16}s_{17}+144s_{11}s_{13}s_{15}s_{17}+144s_{12}s_{13}s_{15}s_{16}+144s_{10}s_{11}s_{18}s_{19}+144s_{10}s_{12}s_{17}s_{19}+144s_{10}s_{13}s_{16}s_{19}+144s_{10}s_{14}s_{15}s_{19}+144s_{10}s_{14}s_{16}s_{18}+144s_{11}s_{12}s_{16}s_{19}+144s_{11}s_{12}s_{17}s_{18}+144s_{11}s_{13}s_{15}s_{19}+288s_{11}s_{13}s_{16}s_{18}+144s_{11}s_{14}s_{15}s_{18}+144s_{11}s_{14}s_{16}s_{17}+144s_{12}s_{13}s_{16}s_{17}+144s_{12}s_{14}s_{15}s_{17}+144s_{13}s_{14}s_{15}s_{16}+144s_{10}s_{12}s_{18}s_{19}+144s_{10}s_{13}s_{17}s_{19}+144s_{10}s_{14}s_{16}s_{19}+144s_{10}s_{14}s_{17}s_{18}+144s_{11}s_{12}s_{17}s_{19}+144s_{11}s_{13}s_{16}s_{19}+144s_{11}s_{13}s_{17}s_{18}+144s_{11}s_{14}s_{15}s_{19}+144s_{11}s_{14}s_{16}s_{18}+144s_{12}s_{13}s_{15}s_{19}+144s_{12}s_{13}s_{16}s_{18}+144s_{12}s_{14}s_{15}s_{18}+144s_{12}s_{14}s_{16}s_{17}+144s_{13}s_{14}s_{15}s_{17}+144s_{11}s_{12}s_{18}s_{19}+144s_{11}s_{13}s_{17}s_{19}+288s_{11}s_{14}s_{16}s_{19}+144s_{11}s_{14}s_{17}s_{18}+144s_{12}s_{13}s_{16}s_{19}+288s_{12}s_{13}s_{17}s_{18}+144s_{12}s_{14}s_{16}s_{18}+144s_{13}s_{14}s_{16}s_{17}+144s_{11}s_{13}s_{18}s_{19}+144s_{11}s_{14}s_{17}s_{19}+144s_{12}s_{14}s_{16}s_{19}+144s_{13}s_{14}s_{16}s_{18}+144s_{12}s_{13}s_{18}s_{19}+288s_{12}s_{14}s_{17}s_{19}+144s_{13}s_{14}s_{17}s_{18}+144s_{12}s_{14}s_{18}s_{19}+144s_{13}s_{14}s_{17}s_{19}+288s_{13}s_{14}s_{18}s_{19}+432s_{15}s_{16}s_{17}s_{18}+432s_{15}s_{16}s_{18}s_{19}+432s_{15}s_{17}s_{18}s_{19}+432s_{16}s_{17}s_{18}s_{19}+5760$

A complete expression of $E_k(q)$ for 36-order Williamson based method or 108-order Baumert-Hall based method

$E_k(q)=12096q_0q_1-32544q_1-32544q_2-32544q_3-32544q_4-19584q_5-32544q_6-32544q_7-32544q_8-32544q_9-19584q_{10}-32544q_{11}-32544q_{12}-32544q_{13}-32544q_{14}-19584q_{15}-32544q_{16}-32544q_{17}-32544q_{18}-32544q_{19}-19584q_0+12096q_0q_2+13248q_0q_3+20736q_1q_2+12096q_0q_4+21888q_1q_3+2304q_0q_5+20736q_1q_4+21888q_2q_3+4032q_0q_6+4032q_1q_5+20736q_2q_4+4032q_0q_7+7488q_1q_6+4032q_2q_5+21888q_3q_4+4032q_0q_8+6912q_1q_7+6912q_2q_6+4032q_3q_5+4032q_0q_9+6912q_1q_8+7488q_2q_7+6912q_3q_6+4032q_4q_5+2304q_0q_{10}+6912q_1q_9+6912q_2q_8+6912q_3q_7+6912q_4q_6+4032q_0q_{11}+4032q_1q_{10}+6912q_2q_9+7488q_3q_8+6912q_4q_7+12096q_5q_6+4032q_0q_{12}+7488q_1q_{11}+4032q_2q_{10}+6912q_3q_9+6912q_4q_8+12096q_5q_7+4032q_0q_{13}+6912q_1q_{12}+6912q_2q_{11}+4032q_3q_{10}+7488q_4q_9+13248q_5q_8+20736q_6q_7+4032q_0q_{14}+6912q_1q_{13}+7488q_2q_{12}+6912q_3q_{11}+4032q_4q_{10}+12096q_5q_9+21888q_6q_8+2304q_0q_{15}+6912q_1q_{14}+6912q_2q_{13}+6912q_3q_{12}+6912q_4q_{11}+2304q_5q_{10}+20736q_6q_9+21888q_7q_8+4032q_0q_{16}+4032q_1q_{15}+6912q_2q_{14}+7488q_3q_{13}+6912q_4q_{12}+4032q_5q_{11}+4032q_6q_{10}+20736q_7q_9+4032q_0q_{17}+7488q_1q_{16}+4032q_2q_{15}+6912q_3q_{14}+6912q_4q_{13}+4032q_5q_{12}+7488q_6q_{11}+4032q_7q_{10}+21888q_8q_9+4032q_0q_{18}+6912q_1q_{17}+6912q_2q_{16}+4032q_3q_{15}+7488q_4q_{14}+4032q_5q_{13}+6912q_6q_{12}+6912q_7q_{11}+4032q_8q_{10}+4032q_0q_{19}+6912q_1q_{18}+7488q_2q_{17}+6912q_3q_{16}+4032q_4q_{15}+4032q_5q_{14}+6912q_6q_{13}+7488q_7q_{12}+6912q_8q_{11}+4032q_9q_{10}+6912q_1q_{19}+6912q_2q_{18}+6912q_3q_{17}+6912q_4q_{16}+2304q_5q_{15}+6912q_6q_{14}+6912q_7q_{13}+6912q_8q_{12}+6912q_9q_{11}+6912q_2q_{19}+7488q_3q_{18}+6912q_4q_{17}+4032q_5q_{16}+4032q_6q_{15}+6912q_7q_{14}+7488q_8q_{13}+6912q_9q_{12}+12096q_{10}q_{11}+6912q_3q_{19}+6912q_4q_{18}+4032q_5q_{17}+7488q_6q_{16}+4032q_7q_{15}+6912q_8q_{14}+6912q_9q_{13}+12096q_{10}q_{12}+7488q_4q_{19}+4032q_5q_{18}+6912q_6q_{17}+6912q_7q_{16}+4032q_8q_{15}+7488q_9q_{14}+13248q_{10}q_{13}+20736q_{11}q_{12}+4032q_5q_{19}+6912q_6q_{18}+7488q_7q_{17}+6912q_8q_{16}+4032q_9q_{15}+12096q_{10}q_{14}+21888q_{11}q_{13}+6912q_6q_{19}+6912q_7q_{18}+6912q_8q_{17}+6912q_9q_{16}+2304q_{10}q_{15}+20736q_{11}q_{14}+21888q_{12}q_{13}+6912q_7q_{19}+7488q_8q_{18}+6912q_9q_{17}+4032q_{10}q_{16}+4032q_{11}q_{15}+20736q_{12}q_{14}+6912q_8q_{19}+6912q_9q_{18}+4032q_{10}q_{17}+7488q_{11}q_{16}+4032q_{12}q_{15}+21888q_{13}q_{14}+7488q_9q_{19}+4032q_{10}q_{18}+6912q_{11}q_{17}+6912q_{12}q_{16}+4032q_{13}q_{15}+4032q_{10}q_{19}+6912q_{11}q_{18}+7488q_{12}q_{17}+6912q_{13}q_{16}+4032q_{14}q_{15}+6912q_{11}q_{19}+6912q_{12}q_{18}+6912q_{13}q_{17}+6912q_{14}q_{16}+6912q_{12}q_{19}+7488q_{13}q_{18}+6912q_{14}q_{17}+12096q_{15}q_{16}+6912q_{13}q_{19}+6912q_{14}q_{18}+12096q_{15}q_{17}+7488q_{14}q_{19}+13248q_{15}q_{18}+20736q_{16}q_{17}+12096q_{15}q_{19}+21888q_{16}q_{18}+20736q_{16}q_{19}+21888q_{17}q_{18}+20736q_{17}q_{19}+21888q_{18}q_{19}-3456q_0q_1q_2-6912q_0q_1q_3-3456q_0q_1q_4-6912q_0q_2q_3-1152q_0q_1q_5-3456q_0q_2q_4-6912q_1q_2q_3-2304q_0q_1q_6-1152q_0q_2q_5-6912q_0q_3q_4-3456q_1q_2q_4-2304q_0q_1q_7-1152q_0q_2q_6-1152q_0q_3q_5-2304q_1q_2q_5-6912q_1q_3q_4-2304q_0q_1q_8-2304q_0q_2q_7-2304q_0q_3q_6-1152q_0q_4q_5-4608q_1q_2q_6-2304q_1q_3q_5-6912q_2q_3q_4-1152q_0q_1q_9-2304q_0q_2q_8-2304q_0q_3q_7-2304q_0q_4q_6-4608q_1q_2q_7-3456q_1q_3q_6-2304q_1q_4q_5-2304q_2q_3q_5-1152q_0q_1q_{10}-2304q_0q_2q_9-1152q_0q_3q_8-1152q_0q_4q_7-1152q_0q_5q_6-3456q_1q_2q_8-3456q_1q_3q_7-4608q_1q_4q_6-4608q_2q_3q_6-2304q_2q_4q_5-2304q_0q_1q_{11}-1152q_0q_2q_{10}-2304q_0q_3q_9-2304q_0q_4q_8-1152q_0q_5q_7-3456q_1q_2q_9-4608q_1q_3q_8-3456q_1q_4q_7-2304q_1q_5q_6-$

$$3456q_2q_3q_7 - 3456q_2q_4q_6 - 2304q_3q_4q_5 - 2304q_0q_1q_{12} - 1152q_0q_2q_{11} - 1152q_0q_3q_{10} - 2304q_0q_4q_9 -$$
$$1152q_0q_5q_8 - 2304q_0q_6q_7 - 2304q_1q_2q_{10} - 4608q_1q_3q_9 - 3456q_1q_4q_8 - 1152q_1q_5q_7 - 4608q_2q_3q_8 -$$
$$4608q_2q_4q_7 - 2304q_2q_5q_6 - 3456q_3q_4q_6 - 2304q_0q_1q_{13} - 2304q_0q_2q_{12} - 2304q_0q_3q_{11} - 1152q_0q_4q_{10} -$$
$$1152q_0q_5q_9 - 2304q_0q_6q_8 - 4608q_1q_2q_{11} - 2304q_1q_3q_{10} - 4608q_1q_4q_9 - 2304q_1q_5q_8 - 4608q_1q_6q_7 -$$
$$3456q_2q_3q_9 - 3456q_2q_4q_8 - 2304q_2q_5q_7 - 4608q_3q_4q_7 - 2304q_3q_5q_6 - 1152q_0q_1q_{14} - 2304q_0q_2q_{13} -$$
$$2304q_0q_3q_{12} - 2304q_0q_4q_{11} - 2304q_0q_6q_9 - 2304q_0q_7q_8 - 4608q_1q_2q_{12} - 3456q_1q_3q_{11} - 2304q_1q_4q_{10} -$$
$$2304q_1q_5q_9 - 3456q_1q_6q_8 - 2304q_2q_3q_{10} - 4608q_2q_4q_9 - 2304q_2q_5q_8 - 4608q_2q_6q_7 - 4608q_3q_4q_8 -$$
$$2304q_3q_5q_7 - 1152q_4q_5q_6 - 1152q_0q_1q_{15} - 2304q_0q_2q_{14} - 1152q_0q_3q_{13} - 1152q_0q_4q_{12} - 2304q_0q_7q_9 -$$
$$3456q_1q_2q_{13} - 3456q_1q_3q_{12} - 4608q_1q_4q_{11} - 4608q_1q_6q_9 - 4608q_1q_7q_8 - 4608q_2q_3q_{11} - 2304q_2q_4q_{10} -$$
$$1152q_2q_5q_9 - 3456q_2q_6q_8 - 3456q_3q_4q_9 - 1152q_3q_5q_8 - 3456q_3q_6q_7 - 2304q_4q_5q_7 - 2304q_0q_1q_{16} -$$
$$1152q_0q_2q_{15} - 2304q_0q_3q_{14} - 2304q_0q_4q_{13} - 2304q_0q_8q_9 - 3456q_1q_2q_{14} - 4608q_1q_3q_{13} - 3456q_1q_4q_{12} -$$
$$3456q_1q_7q_9 - 3456q_2q_3q_{12} - 3456q_2q_4q_{11} - 3456q_2q_6q_9 - 3456q_2q_7q_8 - 2304q_3q_4q_{10} - 2304q_3q_5q_9 -$$
$$4608q_3q_6q_8 - 2304q_4q_5q_8 - 3456q_4q_6q_7 - 2304q_0q_1q_{17} - 1152q_0q_2q_{16} - 1152q_0q_3q_{15} - 2304q_0q_4q_{14} -$$
$$2304q_1q_2q_{15} - 4608q_1q_3q_{14} - 3456q_1q_4q_{13} - 3456q_1q_8q_9 - 4608q_2q_3q_{13} - 4608q_2q_4q_{12} - 4608q_2q_7q_9 -$$
$$3456q_3q_4q_{11} - 3456q_3q_6q_9 - 4608q_3q_7q_8 - 2304q_4q_5q_9 - 4608q_4q_6q_8 - 3456q_5q_6q_7 - 2304q_0q_1q_{18} -$$
$$2304q_0q_2q_{17} - 2304q_0q_3q_{16} - 1152q_0q_4q_{15} - 4608q_1q_2q_{16} - 2304q_1q_3q_{15} - 4608q_1q_4q_{14} - 3456q_2q_3q_{14} -$$
$$3456q_2q_4q_{13} - 4608q_2q_8q_9 - 4608q_3q_4q_{12} - 3456q_3q_7q_9 - 4608q_4q_6q_9 - 3456q_4q_7q_8 - 6912q_5q_6q_8 -$$
$$1152q_0q_1q_{19} - 2304q_0q_2q_{18} - 2304q_0q_3q_{17} - 2304q_0q_4q_{16} - 4608q_1q_2q_{17} - 3456q_1q_3q_{16} - 2304q_1q_4q_{15} -$$
$$2304q_2q_3q_{15} - 4608q_2q_4q_{14} - 4608q_3q_4q_{13} - 4608q_3q_8q_9 - 4608q_4q_7q_9 - 3456q_5q_6q_9 - 6912q_5q_7q_8 -$$
$$2304q_0q_2q_{19} - 1152q_0q_3q_{18} - 1152q_0q_4q_{17} - 1152q_0q_{10}q_{11} - 3456q_1q_2q_{18} - 3456q_1q_3q_{17} - 4608q_1q_4q_{16} -$$
$$4608q_2q_3q_{16} - 2304q_2q_4q_{15} - 3456q_3q_4q_{14} - 3456q_4q_8q_9 - 1152q_5q_6q_{10} - 3456q_5q_7q_9 - 6912q_6q_7q_8 -$$
$$2304q_0q_3q_{19} - 2304q_0q_4q_{18} - 1152q_0q_{10}q_{12} - 3456q_1q_2q_{19} - 4608q_1q_3q_{18} - 3456q_1q_4q_{17} - 2304q_1q_{10}q_{11} -$$
$$3456q_2q_3q_{17} - 3456q_2q_4q_{16} - 2304q_3q_4q_{15} - 2304q_5q_6q_{11} - 1152q_5q_7q_{10} - 6912q_5q_8q_9 - 3456q_6q_7q_9 -$$
$$2304q_0q_4q_{19} - 1152q_0q_{10}q_{13} - 2304q_0q_{11}q_{12} - 4608q_1q_3q_{19} - 3456q_1q_4q_{18} - 1152q_1q_{10}q_{12} - 4608q_2q_3q_{18} -$$
$$4608q_2q_4q_{17} - 2304q_2q_{10}q_{11} - 3456q_3q_4q_{16} - 2304q_5q_6q_{12} - 1152q_5q_7q_{11} - 1152q_5q_8q_{10} - 2304q_6q_7q_{10} -$$
$$6912q_6q_8q_9 - 1152q_0q_{10}q_{14} - 2304q_0q_{11}q_{13} - 4608q_1q_4q_{19} - 2304q_1q_{10}q_{13} - 4608q_1q_{11}q_{12} - 3456q_2q_3q_{19} -$$
$$3456q_2q_4q_{18} - 2304q_2q_{10}q_{12} - 4608q_3q_4q_{17} - 2304q_3q_{10}q_{11} - 2304q_5q_6q_{13} - 2304q_5q_7q_{12} - 2304q_5q_8q_{11} -$$
$$1152q_5q_9q_{10} - 4608q_6q_7q_{11} - 2304q_6q_8q_{10} - 6912q_7q_8q_9 - 2304q_0q_{11}q_{14} - 2304q_0q_{12}q_{13} - 2304q_1q_{10}q_{14} -$$
$$3456q_1q_{11}q_{13} - 4608q_2q_4q_{19} - 2304q_2q_{10}q_{13} - 4608q_2q_{11}q_{12} - 4608q_3q_4q_{18} - 2304q_3q_{10}q_{12} - 1152q_4q_{10}q_{11} -$$
$$1152q_5q_6q_{14} - 2304q_5q_7q_{13} - 2304q_5q_8q_{12} - 2304q_5q_9q_{11} - 4608q_6q_7q_{12} - 3456q_6q_8q_{11} - 2304q_6q_9q_{10} -$$
$$2304q_7q_8q_{10} - 2304q_0q_{12}q_{14} - 4608q_1q_{11}q_{14} - 4608q_1q_{12}q_{13} - 1152q_2q_{10}q_{14} - 3456q_2q_{11}q_{13} - 3456q_3q_4q_{19} -$$
$$1152q_3q_{10}q_{13} - 3456q_3q_{11}q_{12} - 2304q_4q_{10}q_{12} - 1152q_5q_6q_{15} - 2304q_5q_7q_{14} - 1152q_5q_8q_{13} - 1152q_5q_9q_{12} -$$
$$1152q_5q_{10}q_{11} - 3456q_6q_7q_{13} - 3456q_6q_8q_{12} - 4608q_6q_9q_{11} - 4608q_7q_8q_{11} - 2304q_7q_9q_{10} - 2304q_0q_{13}q_{14} -$$
$$3456q_1q_{12}q_{14} - 3456q_2q_{11}q_{14} - 3456q_2q_{12}q_{13} - 2304q_3q_{10}q_{14} - 4608q_3q_{11}q_{13} - 2304q_4q_{10}q_{13} - 3456q_4q_{11}q_{12} -$$
$$2304q_5q_6q_{16} - 1152q_5q_7q_{15} - 2304q_5q_8q_{14} - 2304q_5q_9q_{13} - 1152q_5q_{10}q_{12} - 3456q_6q_7q_{14} - 4608q_6q_8q_{13} -$$
$$3456q_6q_9q_{12} - 2304q_6q_{10}q_{11} - 3456q_7q_8q_{12} - 3456q_7q_9q_{11} - 2304q_8q_9q_{10} - 3456q_1q_{13}q_{14} - 4608q_2q_{12}q_{14} -$$
$$3456q_3q_{11}q_{14} - 4608q_3q_{12}q_{13} - 2304q_4q_{10}q_{14} - 4608q_4q_{11}q_{13} - 2304q_5q_6q_{17} - 1152q_5q_7q_{16} - 1152q_5q_8q_{15} -$$
$$2304q_5q_9q_{14} - 1152q_5q_{10}q_{13} - 2304q_5q_{11}q_{12} - 2304q_6q_7q_{15} - 4608q_6q_8q_{14} - 3456q_6q_9q_{13} - 1152q_6q_{10}q_{12} -$$
$$4608q_7q_8q_{13} - 4608q_7q_9q_{12} - 2304q_7q_{10}q_{11} - 3456q_8q_9q_{11} - 4608q_2q_{13}q_{14} - 3456q_3q_{12}q_{14} - 4608q_4q_{11}q_{14} -$$
$$3456q_4q_{12}q_{13} - 2304q_5q_6q_{18} - 2304q_5q_7q_{17} - 2304q_5q_8q_{16} - 1152q_5q_9q_{15} - 1152q_5q_{10}q_{14} - 2304q_5q_{11}q_{13} -$$
$$4608q_6q_7q_{16} - 2304q_6q_8q_{15} - 4608q_6q_9q_{14} - 2304q_6q_{10}q_{13} - 4608q_6q_{11}q_{12} - 3456q_7q_8q_{14} - 3456q_7q_9q_{13} -$$
$$2304q_7q_{10}q_{12} - 4608q_8q_9q_{12} - 2304q_8q_{10}q_{11} - 4608q_3q_{13}q_{14} - 4608q_4q_{12}q_{14} - 1152q_5q_6q_{19} - 2304q_5q_7q_{18} -$$
$$2304q_5q_8q_{17} - 2304q_5q_9q_{16} - 2304q_5q_{11}q_{14} - 2304q_5q_{12}q_{13} - 4608q_6q_7q_{17} - 3456q_6q_8q_{16} - 2304q_6q_9q_{15} -$$
$$2304q_6q_{10}q_{14} - 3456q_6q_{11}q_{13} - 2304q_7q_8q_{15} - 4608q_7q_9q_{14} - 2304q_7q_{10}q_{13} - 4608q_7q_{11}q_{12} - 4608q_8q_9q_{13} -$$
$$2304q_8q_{10}q_{12} - 1152q_9q_{10}q_{11} - 1152q_0q_{15}q_{16} - 3456q_4q_{13}q_{14} - 2304q_5q_7q_{19} - 1152q_5q_8q_{18} - 1152q_5q_9q_{17} -$$
$$2304q_5q_{12}q_{14} - 3456q_6q_7q_{18} - 3456q_6q_8q_{17} - 4608q_6q_9q_{16} - 4608q_6q_{11}q_{14} - 4608q_6q_{12}q_{13} - 4608q_7q_8q_{16} -$$
$$2304q_7q_9q_{15} - 1152q_7q_{10}q_{14} - 3456q_7q_{11}q_{13} - 3456q_8q_9q_{14} - 1152q_8q_{10}q_{13} - 3456q_8q_{11}q_{12} - 2304q_9q_{10}q_{12} -$$
$$1152q_0q_{15}q_{17} - 2304q_1q_{15}q_{16} - 2304q_5q_8q_{19} - 2304q_5q_9q_{18} - 2304q_5q_{13}q_{14} - 3456q_6q_7q_{19} - 4608q_6q_8q_{18} -$$

$3456q_6q_9q_{17} - 3456q_6q_{12}q_{14} - 3456q_7q_8q_{17} - 3456q_7q_9q_{16} - 3456q_7q_{11}q_{14} - 3456q_7q_{12}q_{13} - 2304q_8q_9q_{15} -$
$2304q_8q_{10}q_{14} - 4608q_8q_{11}q_{13} - 2304q_9q_{10}q_{13} - 3456q_9q_{11}q_{12} - 1152q_0q_{15}q_{18} - 2304q_0q_{16}q_{17} - 1152q_1q_{15}q_{17} -$
$2304q_2q_{15}q_{16} - 2304q_5q_9q_{19} - 4608q_6q_8q_{19} - 3456q_6q_9q_{18} - 3456q_6q_{13}q_{14} - 4608q_7q_8q_{18} - 4608q_7q_9q_{17} -$
$4608q_7q_{12}q_{14} - 3456q_8q_9q_{16} - 3456q_8q_{11}q_{14} - 4608q_8q_{12}q_{13} - 2304q_9q_{10}q_{14} - 4608q_9q_{11}q_{13} - 3456q_{10}q_{11}q_{12} -$
$1152q_0q_{15}q_{19} - 2304q_0q_{16}q_{18} - 2304q_1q_{15}q_{18} - 4608q_1q_{16}q_{17} - 2304q_2q_{15}q_{17} - 2304q_3q_{15}q_{16} - 4608q_6q_9q_{19} -$
$3456q_7q_8q_{19} - 3456q_7q_9q_{18} - 4608q_7q_{13}q_{14} - 4608q_8q_9q_{17} - 3456q_8q_{12}q_{14} - 4608q_9q_{11}q_{14} - 3456q_9q_{12}q_{13} -$
$6912q_{10}q_{11}q_{13} - 2304q_0q_{16}q_{19} - 2304q_0q_{17}q_{18} - 2304q_1q_{15}q_{19} - 3456q_1q_{16}q_{18} - 2304q_2q_{15}q_{18} - 4608q_2q_{16}q_{17} -$
$2304q_3q_{15}q_{17} - 1152q_4q_{15}q_{16} - 4608q_7q_9q_{19} - 4608q_8q_9q_{18} - 4608q_8q_{13}q_{14} - 4608q_9q_{12}q_{14} - 3456q_{10}q_{11}q_{14} -$
$6912q_{10}q_{12}q_{13} - 2304q_0q_{17}q_{19} - 4608q_1q_{16}q_{19} - 4608q_1q_{17}q_{18} - 1152q_2q_{15}q_{19} - 3456q_2q_{16}q_{18} - 1152q_3q_{15}q_{18} -$
$3456q_3q_{16}q_{17} - 2304q_4q_{15}q_{17} - 1152q_5q_{15}q_{16} - 3456q_8q_9q_{19} - 3456q_9q_{13}q_{14} - 1152q_{10}q_{11}q_{15} - 3456q_{10}q_{12}q_{14} -$
$6912q_{11}q_{12}q_{13} - 2304q_0q_{18}q_{19} - 3456q_1q_{17}q_{19} - 3456q_2q_{16}q_{19} - 3456q_2q_{17}q_{18} - 2304q_3q_{15}q_{19} - 4608q_3q_{16}q_{18} -$
$2304q_4q_{15}q_{18} - 3456q_4q_{16}q_{17} - 1152q_5q_{15}q_{17} - 2304q_6q_{15}q_{16} - 2304q_{10}q_{11}q_{16} - 1152q_{10}q_{12}q_{15} -$
$6912q_{10}q_{13}q_{14} - 3456q_{11}q_{12}q_{14} - 3456q_1q_{18}q_{19} - 4608q_2q_{17}q_{19} - 3456q_3q_{16}q_{19} - 4608q_3q_{17}q_{18} -$
$2304q_4q_{15}q_{19} - 4608q_4q_{16}q_{18} - 1152q_5q_{15}q_{18} - 2304q_5q_{16}q_{17} - 1152q_6q_{15}q_{17} - 2304q_7q_{15}q_{16} - 2304q_{10}q_{11}q_{17} -$
$1152q_{10}q_{12}q_{16} - 1152q_{10}q_{13}q_{15} - 2304q_{11}q_{12}q_{15} - 6912q_{11}q_{13}q_{14} - 4608q_2q_{18}q_{19} - 3456q_3q_{17}q_{19} -$
$4608q_4q_{16}q_{19} - 3456q_4q_{17}q_{18} - 1152q_5q_{15}q_{19} - 2304q_5q_{16}q_{18} - 2304q_6q_{15}q_{18} - 4608q_6q_{16}q_{17} - 2304q_7q_{15}q_{17} -$
$2304q_8q_{15}q_{16} - 2304q_{10}q_{11}q_{18} - 2304q_{10}q_{12}q_{17} - 2304q_{10}q_{13}q_{16} - 1152q_{10}q_{14}q_{15} - 4608q_{11}q_{12}q_{16} -$
$2304q_{11}q_{13}q_{15} - 6912q_{12}q_{13}q_{14} - 4608q_3q_{18}q_{19} - 4608q_4q_{17}q_{19} - 2304q_5q_{16}q_{19} - 2304q_5q_{17}q_{18} -$
$2304q_6q_{15}q_{19} - 3456q_6q_{16}q_{18} - 2304q_7q_{15}q_{18} - 4608q_7q_{16}q_{17} - 2304q_8q_{15}q_{17} - 1152q_9q_{15}q_{16} - 1152q_{10}q_{11}q_{19} -$
$2304q_{10}q_{12}q_{18} - 2304q_{10}q_{13}q_{17} - 2304q_{10}q_{14}q_{16} - 4608q_{11}q_{12}q_{17} - 3456q_{11}q_{13}q_{16} - 2304q_{11}q_{14}q_{15} -$
$2304q_{12}q_{13}q_{15} - 3456q_4q_{18}q_{19} - 2304q_5q_{17}q_{19} - 4608q_6q_{16}q_{19} - 4608q_6q_{17}q_{18} - 1152q_7q_{15}q_{19} - 3456q_7q_{16}q_{18} -$
$1152q_8q_{15}q_{18} - 3456q_8q_{16}q_{17} - 2304q_9q_{15}q_{17} - 2304q_{10}q_{12}q_{19} - 1152q_{10}q_{13}q_{18} - 1152q_{10}q_{14}q_{17} -$
$1152q_{10}q_{15}q_{16} - 3456q_{11}q_{12}q_{18} - 3456q_{11}q_{13}q_{17} - 4608q_{11}q_{14}q_{16} - 4608q_{12}q_{13}q_{16} - 2304q_{12}q_{14}q_{15} -$
$2304q_5q_{18}q_{19} - 3456q_6q_{17}q_{19} - 3456q_7q_{16}q_{19} - 3456q_7q_{17}q_{18} - 2304q_8q_{15}q_{19} - 4608q_8q_{16}q_{18} - 2304q_9q_{15}q_{18} -$
$3456q_9q_{16}q_{17} - 2304q_{10}q_{13}q_{19} - 2304q_{10}q_{14}q_{18} - 1152q_{10}q_{15}q_{17} - 3456q_{11}q_{12}q_{19} - 4608q_{11}q_{13}q_{18} -$
$3456q_{11}q_{14}q_{17} - 2304q_{11}q_{15}q_{16} - 3456q_{12}q_{13}q_{17} - 3456q_{12}q_{14}q_{16} - 2304q_{13}q_{14}q_{15} - 3456q_6q_{18}q_{19} -$
$4608q_7q_{17}q_{19} - 3456q_8q_{16}q_{19} - 4608q_8q_{17}q_{18} - 2304q_9q_{15}q_{19} - 4608q_9q_{16}q_{18} - 2304q_{10}q_{14}q_{19} - 1152q_{10}q_{15}q_{18} -$
$2304q_{10}q_{16}q_{17} - 4608q_{11}q_{13}q_{19} - 3456q_{11}q_{14}q_{18} - 1152q_{11}q_{15}q_{17} - 4608q_{12}q_{13}q_{18} - 4608q_{12}q_{14}q_{17} -$
$2304q_{12}q_{15}q_{16} - 3456q_{13}q_{14}q_{16} - 4608q_7q_{18}q_{19} - 3456q_8q_{17}q_{19} - 4608q_9q_{16}q_{19} - 3456q_9q_{17}q_{18} -$
$1152q_{10}q_{15}q_{19} - 2304q_{10}q_{16}q_{18} - 4608q_{11}q_{14}q_{19} - 2304q_{11}q_{15}q_{18} - 4608q_{11}q_{16}q_{17} - 3456q_{12}q_{13}q_{19} -$
$3456q_{12}q_{14}q_{18} - 2304q_{12}q_{15}q_{17} - 4608q_{13}q_{14}q_{17} - 2304q_{13}q_{15}q_{16} - 4608q_8q_{18}q_{19} - 4608q_9q_{17}q_{19} -$
$2304q_{10}q_{16}q_{19} - 2304q_{10}q_{17}q_{18} - 2304q_{11}q_{15}q_{19} - 3456q_{11}q_{16}q_{18} - 4608q_{12}q_{14}q_{19} - 2304q_{12}q_{15}q_{18} -$
$4608q_{12}q_{16}q_{17} - 4608q_{13}q_{14}q_{18} - 2304q_{13}q_{15}q_{17} - 1152q_{14}q_{15}q_{16} - 3456q_9q_{18}q_{19} - 2304q_{10}q_{17}q_{19} -$
$4608q_{11}q_{16}q_{19} - 4608q_{11}q_{17}q_{18} - 1152q_{12}q_{15}q_{19} - 3456q_{12}q_{16}q_{18} - 3456q_{13}q_{14}q_{19} - 1152q_{13}q_{15}q_{18} -$
$3456q_{13}q_{16}q_{17} - 2304q_{14}q_{15}q_{17} - 2304q_{10}q_{18}q_{19} - 3456q_{11}q_{17}q_{19} - 3456q_{12}q_{16}q_{19} - 3456q_{12}q_{17}q_{18} -$
$2304q_{13}q_{15}q_{19} - 4608q_{13}q_{16}q_{18} - 2304q_{14}q_{15}q_{18} - 3456q_{14}q_{16}q_{17} - 3456q_{11}q_{18}q_{19} - 4608q_{12}q_{17}q_{19} -$
$3456q_{13}q_{16}q_{19} - 4608q_{13}q_{17}q_{18} - 2304q_{14}q_{15}q_{19} - 4608q_{14}q_{16}q_{18} - 3456q_{15}q_{16}q_{17} - 4608q_{12}q_{18}q_{19} -$
$3456q_{13}q_{17}q_{19} - 4608q_{14}q_{16}q_{19} - 3456q_{14}q_{17}q_{18} - 6912q_{15}q_{16}q_{18} - 4608q_{13}q_{18}q_{19} - 4608q_{14}q_{17}q_{19} -$
$3456q_{15}q_{16}q_{19} - 6912q_{15}q_{17}q_{18} - 3456q_{14}q_{18}q_{19} - 3456q_{15}q_{17}q_{19} - 6912q_{16}q_{17}q_{18} - 6912q_{15}q_{18}q_{19} -$
$3456q_{16}q_{17}q_{19} - 6912q_{16}q_{18}q_{19} - 6912q_{17}q_{18}q_{19} + 6912q_0q_1q_2q_3 + 6912q_0q_1q_3q_4 + 6912q_0q_2q_3q_4 +$
$6912q_1q_2q_3q_4 + 2304q_0q_1q_5q_6 + 2304q_0q_1q_6q_7 + 2304q_0q_2q_5q_7 + 2304q_1q_2q_5q_6 + 2304q_0q_1q_7q_8 +$
$2304q_0q_2q_6q_8 + 2304q_0q_3q_5q_8 + 2304q_0q_3q_6q_7 + 2304q_1q_2q_5q_8 + 4608q_1q_2q_6q_7 + 2304q_1q_3q_5q_7 +$
$2304q_2q_3q_5q_6 + 2304q_0q_1q_8q_9 + 2304q_0q_2q_7q_9 + 2304q_0q_3q_6q_9 + 2304q_0q_4q_5q_9 + 2304q_0q_4q_6q_8 +$
$2304q_1q_2q_6q_9 + 2304q_1q_2q_7q_8 + 2304q_1q_3q_5q_9 + 4608q_1q_3q_6q_8 + 2304q_1q_4q_5q_8 + 2304q_1q_4q_6q_7 +$
$2304q_2q_3q_6q_7 + 2304q_2q_4q_5q_7 + 2304q_3q_4q_5q_6 + 2304q_0q_2q_8q_9 + 2304q_0q_3q_7q_9 + 2304q_0q_4q_6q_9 +$
$2304q_0q_4q_7q_8 + 2304q_1q_2q_7q_9 + 2304q_1q_3q_6q_9 + 2304q_1q_3q_7q_8 + 2304q_1q_4q_5q_9 + 2304q_1q_4q_6q_8 +$
$2304q_2q_3q_5q_9 + 2304q_2q_3q_6q_8 + 2304q_2q_4q_5q_8 + 2304q_2q_4q_6q_7 + 2304q_3q_4q_5q_7 + 2304q_1q_2q_8q_9 +$
$2304q_1q_3q_7q_9 + 4608q_1q_4q_6q_9 + 2304q_1q_4q_7q_8 + 2304q_2q_3q_6q_9 + 4608q_2q_3q_7q_8 + 2304q_2q_4q_6q_8 +$

$2304q_3q_4q_6q_7 + 2304q_1q_3q_8q_9 + 2304q_1q_4q_7q_9 + 2304q_2q_4q_6q_9 + 2304q_3q_4q_6q_8 + 2304q_0q_1q_{10}q_{11} +$
$2304q_2q_3q_8q_9 + 4608q_2q_4q_7q_9 + 2304q_3q_4q_7q_8 + 2304q_2q_4q_8q_9 + 2304q_3q_4q_7q_9 + 2304q_0q_1q_{11}q_{12} +$
$2304q_0q_2q_{10}q_{12} + 2304q_1q_2q_{10}q_{11} + 4608q_3q_4q_8q_9 + 2304q_0q_1q_{12}q_{13} + 2304q_0q_2q_{11}q_{13} + 2304q_0q_3q_{10}q_{13} +$
$2304q_0q_3q_{11}q_{12} + 2304q_1q_2q_{10}q_{13} + 4608q_1q_2q_{11}q_{12} + 2304q_1q_3q_{10}q_{12} + 2304q_2q_3q_{10}q_{11} + 6912q_5q_6q_7q_8 +$
$2304q_0q_1q_{13}q_{14} + 2304q_0q_2q_{12}q_{14} + 2304q_0q_3q_{11}q_{14} + 2304q_0q_4q_{10}q_{14} + 2304q_0q_4q_{11}q_{13} + 2304q_1q_2q_{11}q_{14} +$
$2304q_1q_2q_{12}q_{13} + 2304q_1q_3q_{10}q_{14} + 4608q_1q_3q_{11}q_{13} + 2304q_1q_4q_{10}q_{13} + 2304q_1q_4q_{11}q_{12} + 2304q_2q_3q_{11}q_{12} +$
$2304q_2q_4q_{10}q_{12} + 2304q_3q_4q_{10}q_{11} + 6912q_5q_6q_8q_9 + 2304q_0q_2q_{13}q_{14} + 2304q_0q_3q_{12}q_{14} + 2304q_0q_4q_{11}q_{14} +$
$2304q_0q_4q_{12}q_{13} + 2304q_1q_2q_{12}q_{14} + 2304q_1q_3q_{11}q_{14} + 2304q_1q_3q_{12}q_{13} + 2304q_1q_4q_{10}q_{14} + 2304q_1q_4q_{11}q_{13} +$
$2304q_2q_3q_{10}q_{14} + 2304q_2q_3q_{11}q_{13} + 2304q_2q_4q_{10}q_{13} + 2304q_2q_4q_{11}q_{12} + 2304q_3q_4q_{10}q_{12} + 6912q_5q_7q_8q_9 +$
$2304q_1q_2q_{13}q_{14} + 2304q_1q_3q_{12}q_{14} + 4608q_1q_4q_{11}q_{14} + 2304q_1q_4q_{12}q_{13} + 2304q_2q_3q_{11}q_{14} + 4608q_2q_3q_{12}q_{13} +$
$2304q_2q_4q_{11}q_{13} + 2304q_3q_4q_{11}q_{12} + 6912q_6q_7q_8q_9 + 2304q_1q_3q_{13}q_{14} + 2304q_1q_4q_{12}q_{14} + 2304q_2q_4q_{11}q_{14} +$
$2304q_3q_4q_{11}q_{13} + 2304q_0q_1q_{15}q_{16} + 2304q_2q_3q_{13}q_{14} + 4608q_2q_4q_{12}q_{14} + 2304q_3q_4q_{12}q_{13} + 2304q_5q_6q_{10}q_{11} +$
$2304q_2q_4q_{13}q_{14} + 2304q_3q_4q_{12}q_{14} + 2304q_0q_1q_{16}q_{17} + 2304q_0q_2q_{15}q_{17} + 2304q_1q_2q_{15}q_{16} + 4608q_3q_4q_{13}q_{14} +$
$2304q_5q_6q_{11}q_{12} + 2304q_5q_7q_{10}q_{12} + 2304q_6q_7q_{10}q_{11} + 2304q_0q_1q_{17}q_{18} + 2304q_0q_2q_{16}q_{18} + 2304q_0q_3q_{15}q_{18} +$
$2304q_0q_3q_{16}q_{17} + 2304q_1q_2q_{15}q_{18} + 4608q_1q_2q_{16}q_{17} + 2304q_1q_3q_{15}q_{17} + 2304q_2q_3q_{15}q_{16} + 2304q_5q_6q_{12}q_{13} +$
$2304q_5q_7q_{11}q_{13} + 2304q_5q_8q_{10}q_{13} + 2304q_5q_8q_{11}q_{12} + 2304q_6q_7q_{10}q_{13} + 4608q_6q_7q_{11}q_{12} + 2304q_6q_8q_{10}q_{12} +$
$2304q_7q_8q_{10}q_{11} + 2304q_0q_1q_{18}q_{19} + 2304q_0q_2q_{17}q_{19} + 2304q_0q_3q_{16}q_{19} + 2304q_0q_4q_{15}q_{19} + 2304q_0q_4q_{16}q_{18} +$
$2304q_1q_2q_{16}q_{19} + 2304q_1q_2q_{17}q_{18} + 2304q_1q_3q_{15}q_{19} + 4608q_1q_3q_{16}q_{18} + 2304q_1q_4q_{15}q_{18} + 2304q_1q_4q_{16}q_{17} +$
$2304q_2q_3q_{16}q_{17} + 2304q_2q_4q_{15}q_{17} + 2304q_3q_4q_{15}q_{16} + 2304q_5q_6q_{13}q_{14} + 2304q_5q_7q_{12}q_{14} + 2304q_5q_8q_{11}q_{14} +$
$2304q_5q_9q_{10}q_{14} + 2304q_5q_9q_{11}q_{13} + 2304q_6q_7q_{11}q_{14} + 2304q_6q_7q_{12}q_{13} + 2304q_6q_8q_{10}q_{14} + 4608q_6q_8q_{11}q_{13} +$
$2304q_6q_9q_{10}q_{13} + 2304q_6q_9q_{11}q_{12} + 2304q_7q_8q_{11}q_{12} + 2304q_7q_9q_{10}q_{12} + 2304q_8q_9q_{10}q_{11} + 2304q_0q_2q_{18}q_{19} +$
$2304q_0q_3q_{17}q_{19} + 2304q_0q_4q_{16}q_{19} + 2304q_0q_4q_{17}q_{18} + 2304q_1q_2q_{17}q_{19} + 2304q_1q_3q_{16}q_{19} + 2304q_1q_3q_{17}q_{18} +$
$2304q_1q_4q_{15}q_{19} + 2304q_1q_4q_{16}q_{18} + 2304q_2q_3q_{15}q_{19} + 2304q_2q_3q_{16}q_{18} + 2304q_2q_4q_{15}q_{18} + 2304q_2q_4q_{16}q_{17} +$
$2304q_3q_4q_{15}q_{17} + 2304q_5q_7q_{13}q_{14} + 2304q_5q_8q_{12}q_{14} + 2304q_5q_9q_{11}q_{14} + 2304q_5q_9q_{12}q_{13} + 2304q_6q_7q_{12}q_{14} +$
$2304q_6q_8q_{11}q_{14} + 2304q_6q_8q_{12}q_{13} + 2304q_6q_9q_{10}q_{14} + 2304q_6q_9q_{11}q_{13} + 2304q_7q_8q_{10}q_{14} + 2304q_7q_8q_{11}q_{13} +$
$2304q_7q_9q_{10}q_{13} + 2304q_7q_9q_{11}q_{12} + 2304q_8q_9q_{10}q_{12} + 2304q_1q_2q_{18}q_{19} + 2304q_1q_3q_{17}q_{19} + 4608q_1q_4q_{16}q_{19} +$
$2304q_1q_4q_{17}q_{18} + 2304q_2q_3q_{16}q_{19} + 4608q_2q_3q_{17}q_{18} + 2304q_2q_4q_{16}q_{18} + 2304q_3q_4q_{16}q_{17} + 2304q_6q_7q_{13}q_{14} +$
$2304q_6q_8q_{12}q_{14} + 4608q_6q_9q_{11}q_{14} + 2304q_6q_9q_{12}q_{13} + 2304q_7q_8q_{11}q_{14} + 4608q_7q_8q_{12}q_{13} + 2304q_7q_9q_{11}q_{13} +$
$2304q_8q_9q_{11}q_{12} + 2304q_1q_3q_{18}q_{19} + 2304q_1q_4q_{17}q_{19} + 2304q_2q_4q_{16}q_{19} + 2304q_3q_4q_{16}q_{18} + 2304q_6q_8q_{13}q_{14} +$
$2304q_6q_9q_{12}q_{14} + 2304q_7q_9q_{11}q_{14} + 2304q_8q_9q_{11}q_{13} + 2304q_2q_3q_{18}q_{19} + 4608q_2q_4q_{17}q_{19} + 2304q_3q_4q_{17}q_{18} +$
$2304q_5q_6q_{15}q_{16} + 2304q_7q_8q_{13}q_{14} + 4608q_7q_9q_{12}q_{14} + 2304q_8q_9q_{12}q_{13} + 2304q_2q_4q_{18}q_{19} + 2304q_3q_4q_{17}q_{19} +$
$2304q_7q_9q_{13}q_{14} + 2304q_8q_9q_{12}q_{14} + 4608q_3q_4q_{18}q_{19} + 2304q_5q_6q_{16}q_{17} + 2304q_5q_7q_{15}q_{17} + 2304q_6q_7q_{15}q_{16} +$
$4608q_8q_9q_{13}q_{14} + 2304q_5q_6q_{17}q_{18} + 2304q_5q_7q_{16}q_{18} + 2304q_5q_8q_{15}q_{18} + 2304q_5q_8q_{16}q_{17} + 2304q_6q_7q_{15}q_{18} +$
$4608q_6q_7q_{16}q_{17} + 2304q_6q_8q_{15}q_{17} + 2304q_7q_8q_{15}q_{16} + 6912q_{10}q_{11}q_{12}q_{13} + 2304q_5q_6q_{18}q_{19} + 2304q_5q_7q_{17}q_{19} +$
$2304q_5q_8q_{16}q_{19} + 2304q_5q_9q_{15}q_{19} + 2304q_5q_9q_{16}q_{18} + 2304q_6q_7q_{16}q_{19} + 2304q_6q_7q_{17}q_{18} + 2304q_6q_8q_{15}q_{19} +$
$4608q_6q_8q_{16}q_{18} + 2304q_6q_9q_{15}q_{18} + 2304q_6q_9q_{16}q_{17} + 2304q_7q_8q_{16}q_{17} + 2304q_7q_9q_{15}q_{17} + 2304q_8q_9q_{15}q_{16} +$
$6912q_{10}q_{11}q_{13}q_{14} + 2304q_5q_7q_{18}q_{19} + 2304q_5q_8q_{17}q_{19} + 2304q_5q_9q_{16}q_{19} + 2304q_5q_9q_{17}q_{18} + 2304q_6q_7q_{17}q_{19} +$
$2304q_6q_8q_{16}q_{19} + 2304q_6q_8q_{17}q_{18} + 2304q_6q_9q_{15}q_{19} + 2304q_6q_9q_{16}q_{18} + 2304q_7q_8q_{15}q_{19} + 2304q_7q_8q_{16}q_{18} +$
$2304q_7q_9q_{15}q_{18} + 2304q_7q_9q_{16}q_{17} + 2304q_8q_9q_{15}q_{17} + 6912q_{10}q_{12}q_{13}q_{14} + 2304q_6q_7q_{18}q_{19} + 2304q_6q_8q_{17}q_{19} +$
$4608q_6q_9q_{16}q_{19} + 2304q_6q_9q_{17}q_{18} + 2304q_7q_8q_{16}q_{19} + 4608q_7q_8q_{17}q_{18} + 2304q_7q_9q_{16}q_{18} + 2304q_8q_9q_{16}q_{17} +$
$6912q_{11}q_{12}q_{13}q_{14} + 2304q_6q_8q_{18}q_{19} + 2304q_6q_9q_{17}q_{19} + 2304q_7q_9q_{16}q_{19} + 2304q_8q_9q_{16}q_{18} + 2304q_7q_8q_{18}q_{19} +$
$4608q_7q_9q_{17}q_{19} + 2304q_8q_9q_{17}q_{18} + 2304q_{10}q_{11}q_{15}q_{16} + 2304q_7q_9q_{18}q_{19} + 2304q_8q_9q_{17}q_{19} + 4608q_8q_9q_{18}q_{19} +$
$2304q_{10}q_{11}q_{16}q_{17} + 2304q_{10}q_{12}q_{15}q_{17} + 2304q_{11}q_{12}q_{15}q_{16} + 2304q_{10}q_{11}q_{17}q_{18} + 2304q_{10}q_{12}q_{16}q_{18} +$
$2304q_{10}q_{13}q_{15}q_{18} + 2304q_{10}q_{13}q_{16}q_{17} + 2304q_{11}q_{12}q_{15}q_{18} + 4608q_{11}q_{12}q_{16}q_{17} + 2304q_{11}q_{13}q_{15}q_{17} +$
$2304q_{12}q_{13}q_{15}q_{16} + 2304q_{10}q_{11}q_{18}q_{19} + 2304q_{10}q_{12}q_{17}q_{19} + 2304q_{10}q_{13}q_{16}q_{19} + 2304q_{10}q_{14}q_{15}q_{19} +$
$2304q_{10}q_{14}q_{16}q_{18} + 2304q_{11}q_{12}q_{16}q_{19} + 2304q_{11}q_{12}q_{17}q_{18} + 2304q_{11}q_{13}q_{15}q_{19} + 4608q_{11}q_{13}q_{16}q_{18} +$
$2304q_{11}q_{14}q_{15}q_{18} + 2304q_{11}q_{14}q_{16}q_{17} + 2304q_{12}q_{13}q_{16}q_{17} + 2304q_{12}q_{14}q_{15}q_{17} + 2304q_{13}q_{14}q_{15}q_{16} +$
$2304q_{10}q_{12}q_{18}q_{19} + 2304q_{10}q_{13}q_{17}q_{19} + 2304q_{10}q_{14}q_{16}q_{19} + 2304q_{10}q_{14}q_{17}q_{18} + 2304q_{11}q_{12}q_{17}q_{19} +$

$2304q_{11}q_{13}q_{16}q_{19} + 2304q_{11}q_{13}q_{17}q_{18} + 2304q_{11}q_{14}q_{15}q_{19} + 2304q_{11}q_{14}q_{16}q_{18} + 2304q_{12}q_{13}q_{15}q_{19} + 2304q_{12}q_{13}q_{16}q_{18} + 2304q_{12}q_{14}q_{15}q_{18} + 2304q_{12}q_{14}q_{16}q_{17} + 2304q_{13}q_{14}q_{15}q_{17} + 2304q_{11}q_{12}q_{18}q_{19} + 2304q_{11}q_{13}q_{17}q_{19} + 4608q_{11}q_{14}q_{16}q_{19} + 2304q_{11}q_{14}q_{17}q_{18} + 2304q_{12}q_{13}q_{16}q_{19} + 4608q_{12}q_{13}q_{17}q_{18} + 2304q_{12}q_{14}q_{16}q_{18} + 2304q_{13}q_{14}q_{16}q_{17} + 2304q_{11}q_{13}q_{18}q_{19} + 2304q_{11}q_{14}q_{17}q_{19} + 2304q_{12}q_{14}q_{16}q_{19} + 2304q_{13}q_{14}q_{16}q_{18} + 2304q_{12}q_{13}q_{18}q_{19} + 4608q_{12}q_{14}q_{17}q_{19} + 2304q_{13}q_{14}q_{17}q_{18} + 2304q_{12}q_{14}q_{18}q_{19} + 2304q_{13}q_{14}q_{17}q_{19} + 4608q_{13}q_{14}q_{18}q_{19} + 6912q_{15}q_{16}q_{17}q_{18} + 6912q_{15}q_{16}q_{18}q_{19} + 6912q_{15}q_{17}q_{18}q_{19} + 6912q_{16}q_{17}q_{18}q_{19} + 93312$

A complete expression of $E_2(q)$ for 36-order Williamson based method or 108-order Baumert-Hall based method by taking $\delta = 10,545,408$

$E_2(q) = 31648320q_{20} - 32544q_1 - 32544q_2 - 32544q_3 - 32544q_4 - 19584q_5 - 32544q_6 - 32544q_7 - 32544q_8 - 32544q_9 - 19584q_{10} - 32544q_{11} - 32544q_{12} - 32544q_{13} - 32544q_{14} - 19584q_{15} - 32544q_{16} - 32544q_{17} - 32544q_{18} - 32544q_{19} - 19584q_0 + 31648320q_{21} + 31649472q_{22} + 31648320q_{23} + 31656960q_{24} + 31658112q_{25} + 31656960q_{26} + 31658112q_{27} + 31656960q_{28} + 31658112q_{29} + 31648320q_{30} + 31648320q_{31} + 31649472q_{32} + 31648320q_{33} + 31656960q_{34} + 31658112q_{35} + 31656960q_{36} + 31658112q_{37} + 31656960q_{38} + 31658112q_{39} + 31648320q_{40} + 31648320q_{41} + 31649472q_{42} + 31648320q_{43} + 31656960q_{44} + 31658112q_{45} + 31656960q_{46} + 31658112q_{47} + 31656960q_{48} + 31658112q_{49} + 31648320q_{50} + 31648320q_{51} + 31649472q_{52} + 31648320q_{53} + 31656960q_{54} + 31658112q_{55} + 31656960q_{56} + 31658112q_{57} + 31656960q_{58} + 31658112q_{59} + 10545408q_0q_1 + 10545408q_0q_2 + 10545408q_0q_3 + 10545408q_1q_2 + 10545408q_0q_4 + 10545408q_1q_3 + 2304q_0q_5 + 10545408q_1q_4 + 10545408q_2q_3 + 4032q_0q_6 + 4032q_1q_5 + 10545408q_2q_4 + 4032q_0q_7 + 7488q_1q_6 + 4032q_2q_5 + 10545408q_3q_4 + 4032q_0q_8 + 6912q_1q_7 + 6912q_2q_6 + 4032q_3q_5 + 4032q_0q_9 + 6912q_1q_8 + 7488q_2q_7 + 6912q_3q_6 + 4032q_4q_5 + 2304q_0q_{10} + 6912q_1q_9 + 6912q_2q_8 + 6912q_3q_7 + 6912q_4q_6 + 4032q_0q_{11} + 4032q_1q_{10} + 6912q_2q_9 + 7488q_3q_8 + 6912q_4q_7 + 10545408q_5q_6 + 4032q_0q_{12} + 7488q_1q_{11} + 4032q_2q_{10} + 6912q_3q_9 + 6912q_4q_8 + 10545408q_5q_7 + 4032q_0q_{13} + 6912q_1q_{12} + 6912q_2q_{11} + 4032q_3q_{10} + 7488q_4q_9 + 10545408q_5q_8 + 10545408q_6q_7 + 4032q_0q_{14} + 6912q_1q_{13} + 7488q_2q_{12} + 6912q_3q_{11} + 4032q_4q_{10} + 10545408q_5q_9 + 10545408q_6q_8 + 2304q_0q_{15} + 6912q_1q_{14} + 6912q_2q_{13} + 6912q_3q_{12} + 6912q_4q_{11} + 2304q_5q_{10} + 10545408q_6q_9 + 10545408q_7q_8 + 4032q_0q_{16} + 4032q_1q_{15} + 6912q_2q_{14} + 7488q_3q_{13} + 6912q_4q_{12} + 4032q_5q_{11} + 4032q_6q_{10} + 10545408q_7q_9 + 4032q_0q_{17} + 7488q_1q_{16} + 4032q_2q_{15} + 6912q_3q_{14} + 6912q_4q_{13} + 4032q_5q_{12} + 7488q_6q_{11} + 4032q_7q_{10} + 10545408q_8q_9 + 4032q_0q_{18} + 6912q_1q_{17} + 6912q_2q_{16} + 4032q_3q_{15} + 7488q_4q_{14} + 4032q_5q_{13} + 6912q_6q_{12} + 6912q_7q_{11} + 4032q_8q_{10} + 4032q_0q_{19} + 6912q_1q_{18} + 7488q_2q_{17} + 6912q_3q_{16} + 4032q_4q_{15} + 4032q_5q_{14} + 6912q_6q_{13} + 7488q_7q_{12} + 6912q_8q_{11} + 4032q_9q_{10} - 21090816q_0q_{20} + 6912q_1q_{19} + 6912q_2q_{18} + 6912q_3q_{17} + 6912q_4q_{16} + 2304q_5q_{15} + 6912q_6q_{14} + 6912q_7q_{13} + 6912q_8q_{12} + 6912q_9q_{11} - 21090816q_0q_{21} - 21090816q_1q_{20} + 6912q_2q_{19} + 7488q_3q_{18} + 6912q_4q_{17} + 4032q_5q_{16} + 4032q_6q_{15} + 6912q_7q_{14} + 7488q_8q_{13} + 6912q_9q_{12} + 10545408q_{10}q_{11} - 21090816q_0q_{22} - 3456q_2q_{20} + 6912q_3q_{19} + 6912q_4q_{18} + 4032q_5q_{17} + 7488q_6q_{16} + 4032q_7q_{15} + 6912q_8q_{14} + 6912q_9q_{13} + 10545408q_{10}q_{12} - 21090816q_0q_{23} - 21090816q_2q_{21} - 6912q_3q_{20} + 7488q_4q_{19} + 4032q_5q_{18} + 6912q_6q_{17} + 6912q_7q_{16} + 4032q_8q_{15} + 7488q_9q_{14} + 10545408q_{10}q_{13} + 10545408q_{11}q_{12} - 6912q_3q_{21} - 3456q_4q_{20} + 4032q_5q_{19} + 6912q_6q_{18} + 7488q_7q_{17} + 6912q_8q_{16} + 4032q_9q_{15} + 10545408q_{10}q_{14} + 10545408q_{11}q_{13} - 21090816q_1q_{24} - 21090816q_3q_{22} - 3456q_4q_{21} - 1152q_5q_{20} + 6912q_6q_{19} + 6912q_7q_{18} + 6912q_8q_{17} + 6912q_9q_{16} + 2304q_{10}q_{15} + 10545408q_{11}q_{14} + 10545408q_{12}q_{13} - 21090816q_1q_{25} - 21090816q_2q_{24} - 6912q_4q_{22} - 1152q_5q_{21} - 2304q_6q_{20} + 6912q_7q_{19} + 7488q_8q_{18} + 6912q_9q_{17} + 4032q_{10}q_{16} + 4032q_{11}q_{15} + 10545408q_{12}q_{14} - 21090816q_1q_{26} - 6912q_3q_{24} - 21090816q_4q_{23} - 1152q_5q_{22} - 1152q_6q_{21} - 2304q_7q_{20} + 6912q_8q_{19} + 6912q_9q_{18} + 4032q_{10}q_{17} + 7488q_{11}q_{16} + 4032q_{12}q_{15} + 10545408q_{13}q_{14} - 21090816q_3q_{25} - 3456q_4q_{24} - 1152q_5q_{23} - 2304q_6q_{22} - 2304q_7q_{21} - 2304q_8q_{20} + 7488q_9q_{19} + 4032q_{10}q_{18} + 6912q_{11}q_{17} + 6912q_{12}q_{16} + 4032q_{13}q_{15} - 21090816q_2q_{27} - 6912q_4q_{25} - 2304q_5q_{24} - 2304q_6q_{23} - 2304q_7q_{22} - 2304q_8q_{21} - 1152q_9q_{20} + 4032q_{10}q_{19} + 6912q_{11}q_{18} + 7488q_{12}q_{17} + 6912q_{13}q_{16} + 4032q_{14}q_{15} - 1152q_0q_{30} - 21090816q_2q_{28} - 21090816q_3q_{27} - 21090816q_4q_{26} - 2304q_5q_{25} - 4608q_6q_{24} - 1152q_7q_{23} - 1152q_8q_{22} - 2304q_9q_{21} - 1152q_{10}q_{20} + 6912q_{11}q_{19} + 6912q_{12}q_{18} + 6912q_{13}q_{17} + 6912q_{14}q_{16} - 1152q_0q_{31} - 2304q_1q_{30} - 6912q_4q_{27} - 2304q_5q_{26} - 3456q_6q_{25} - 4608q_7q_{24} - 2304q_8q_{23} - 2304q_9q_{22} - 1152q_{10}q_{21} - 2304q_{11}q_{20} +$

$6912q_{12}q_{19} + 7488q_{13}q_{18} + 6912q_{14}q_{17} + 10545408q_{15}q_{16} - 1152q_{0}q_{32} - 1152q_{1}q_{31} - 2304q_{2}q_{30} -$
$21090816q_{3}q_{29} - 21090816q_{4}q_{28} - 2304q_{5}q_{27} - 4608q_{6}q_{26} - 3456q_{7}q_{25} - 3456q_{8}q_{24} - 2304q_{9}q_{23} -$
$1152q_{10}q_{22} - 1152q_{11}q_{21} - 2304q_{12}q_{20} + 6912q_{13}q_{19} + 6912q_{14}q_{18} + 10545408q_{15}q_{17} - 1152q_{0}q_{33} -$
$2304q_{1}q_{32} - 2304q_{2}q_{31} - 2304q_{3}q_{30} - 21090816q_{4}q_{29} - 2304q_{5}q_{28} - 4608q_{6}q_{27} - 3456q_{7}q_{26} - 4608q_{8}q_{25} -$
$3456q_{9}q_{24} - 1152q_{10}q_{23} - 2304q_{11}q_{22} - 2304q_{12}q_{21} - 2304q_{13}q_{20} + 7488q_{14}q_{19} + 10545408q_{15}q_{18} +$
$10545408q_{16}q_{17} - 2304q_{0}q_{34} - 2304q_{1}q_{33} - 2304q_{2}q_{32} - 2304q_{3}q_{31} - 1152q_{4}q_{30} - 2304q_{5}q_{29} - 3456q_{6}q_{28} -$
$3456q_{7}q_{27} - 3456q_{8}q_{26} - 4608q_{9}q_{25} - 2304q_{10}q_{24} - 2304q_{11}q_{23} - 2304q_{12}q_{22} - 2304q_{13}q_{21} - 1152q_{14}q_{20} +$
$10545408q_{15}q_{19} + 10545408q_{16}q_{18} - 2304q_{0}q_{35} - 4608q_{1}q_{34} - 1152q_{2}q_{33} - 1152q_{3}q_{32} - 2304q_{4}q_{31} -$
$21090816q_{5}q_{30} - 3456q_{6}q_{29} - 4608q_{7}q_{28} - 4608q_{8}q_{27} - 4608q_{9}q_{26} - 2304q_{10}q_{25} - 4608q_{11}q_{24} - 1152q_{12}q_{23} -$
$1152q_{13}q_{22} - 2304q_{14}q_{21} - 1152q_{15}q_{20} + 10545408q_{16}q_{19} + 10545408q_{17}q_{18} - 2304q_{0}q_{36} - 3456q_{1}q_{35} -$
$4608q_{2}q_{34} - 2304q_{3}q_{33} - 2304q_{4}q_{32} - 21090816q_{5}q_{31} - 21090816q_{6}q_{30} - 4608q_{7}q_{29} - 3456q_{8}q_{28} -$
$3456q_{9}q_{27} - 2304q_{10}q_{26} - 3456q_{11}q_{25} - 4608q_{12}q_{24} - 2304q_{13}q_{23} - 2304q_{14}q_{22} - 1152q_{15}q_{21} - 2304q_{16}q_{20} +$
$10545408q_{17}q_{19} - 2304q_{0}q_{37} - 4608q_{1}q_{36} - 3456q_{2}q_{35} - 3456q_{3}q_{34} - 2304q_{4}q_{33} - 21090816q_{5}q_{32} -$
$3456q_{7}q_{30} - 4608q_{8}q_{29} - 4608q_{9}q_{28} - 2304q_{10}q_{27} - 4608q_{11}q_{26} - 3456q_{12}q_{25} - 3456q_{13}q_{24} - 2304q_{14}q_{23} -$
$1152q_{15}q_{22} - 1152q_{16}q_{21} - 2304q_{17}q_{20} + 10545408q_{18}q_{19} - 2304q_{0}q_{38} - 4608q_{1}q_{37} - 3456q_{2}q_{36} - 4608q_{3}q_{35} -$
$3456q_{4}q_{34} - 21090816q_{5}q_{33} - 21090816q_{7}q_{31} - 6912q_{8}q_{30} - 3456q_{9}q_{29} - 2304q_{10}q_{28} - 4608q_{11}q_{27} -$
$3456q_{12}q_{26} - 4608q_{13}q_{25} - 3456q_{14}q_{24} - 1152q_{15}q_{23} - 2304q_{16}q_{22} - 2304q_{17}q_{21} - 2304q_{18}q_{20} - 2304q_{0}q_{39} -$
$3456q_{1}q_{38} - 3456q_{2}q_{37} - 3456q_{3}q_{36} - 4608q_{4}q_{35} - 6912q_{8}q_{31} - 3456q_{9}q_{30} - 2304q_{10}q_{29} - 3456q_{11}q_{28} -$
$3456q_{12}q_{27} - 3456q_{13}q_{26} - 4608q_{14}q_{25} - 2304q_{15}q_{24} - 2304q_{16}q_{23} - 2304q_{17}q_{22} - 2304q_{18}q_{21} - 1152q_{19}q_{20} -$
$1152q_{0}q_{40} - 3456q_{1}q_{39} - 4608q_{2}q_{38} - 4608q_{3}q_{37} - 4608q_{4}q_{36} - 21090816q_{6}q_{34} - 21090816q_{8}q_{32} -$
$3456q_{9}q_{31} - 1152q_{10}q_{30} - 3456q_{11}q_{29} - 4608q_{12}q_{28} - 4608q_{13}q_{27} - 4608q_{14}q_{26} - 2304q_{15}q_{25} - 4608q_{16}q_{24} -$
$1152q_{17}q_{23} - 1152q_{18}q_{22} - 2304q_{19}q_{21} - 1152q_{0}q_{41} - 2304q_{1}q_{40} - 4608q_{2}q_{39} - 3456q_{3}q_{38} - 3456q_{4}q_{37} -$
$21090816q_{6}q_{35} - 21090816q_{7}q_{34} - 6912q_{9}q_{32} - 1152q_{10}q_{31} - 2304q_{11}q_{30} - 4608q_{12}q_{29} - 3456q_{13}q_{28} -$
$3456q_{14}q_{27} - 2304q_{15}q_{26} - 3456q_{16}q_{25} - 4608q_{17}q_{24} - 2304q_{18}q_{23} - 2304q_{19}q_{22} - 1152q_{0}q_{42} - 1152q_{1}q_{41} -$
$2304q_{2}q_{40} - 4608q_{3}q_{39} - 4608q_{4}q_{38} - 21090816q_{6}q_{36} - 6912q_{8}q_{34} - 21090816q_{9}q_{33} - 1152q_{10}q_{32} -$
$1152q_{11}q_{31} - 2304q_{12}q_{30} - 4608q_{13}q_{29} - 4608q_{14}q_{28} - 2304q_{15}q_{27} - 4608q_{16}q_{26} - 3456q_{17}q_{25} - 3456q_{18}q_{24} -$
$2304q_{19}q_{23} - 1152q_{0}q_{43} - 2304q_{1}q_{42} - 2304q_{2}q_{41} - 2304q_{3}q_{40} - 3456q_{4}q_{39} - 21090816q_{8}q_{35} - 3456q_{9}q_{34} -$
$1152q_{10}q_{33} - 2304q_{11}q_{32} - 2304q_{12}q_{31} - 2304q_{13}q_{30} - 3456q_{14}q_{29} - 2304q_{15}q_{28} - 4608q_{16}q_{27} - 3456q_{17}q_{26} -$
$4608q_{18}q_{25} - 3456q_{19}q_{24} - 2304q_{0}q_{44} - 2304q_{1}q_{43} - 2304q_{2}q_{42} - 2304q_{3}q_{41} - 1152q_{4}q_{40} - 21090816q_{7}q_{37} -$
$6912q_{9}q_{35} - 2304q_{10}q_{34} - 2304q_{11}q_{33} - 2304q_{12}q_{32} - 2304q_{13}q_{31} - 1152q_{14}q_{30} - 2304q_{15}q_{29} - 3456q_{16}q_{28} -$
$3456q_{17}q_{27} - 3456q_{18}q_{26} - 4608q_{19}q_{25} - 2304q_{0}q_{45} - 4608q_{1}q_{44} - 1152q_{2}q_{43} - 1152q_{3}q_{42} - 2304q_{4}q_{41} -$
$1152q_{5}q_{40} - 21090816q_{7}q_{38} - 21090816q_{8}q_{37} - 21090816q_{9}q_{36} - 2304q_{10}q_{35} - 4608q_{11}q_{34} - 1152q_{12}q_{33} -$
$1152q_{13}q_{32} - 2304q_{14}q_{31} - 1152q_{15}q_{30} - 3456q_{16}q_{29} - 4608q_{17}q_{28} - 4608q_{18}q_{27} - 4608q_{19}q_{26} - 2304q_{0}q_{46} -$
$3456q_{1}q_{45} - 4608q_{2}q_{44} - 2304q_{3}q_{43} - 2304q_{4}q_{42} - 1152q_{5}q_{41} - 2304q_{6}q_{40} - 6912q_{9}q_{37} - 2304q_{10}q_{36} -$
$3456q_{11}q_{35} - 4608q_{12}q_{34} - 2304q_{13}q_{33} - 2304q_{14}q_{32} - 1152q_{15}q_{31} - 2304q_{16}q_{30} - 4608q_{17}q_{29} - 3456q_{18}q_{28} -$
$3456q_{19}q_{27} - 2304q_{0}q_{47} - 4608q_{1}q_{46} - 3456q_{2}q_{45} - 3456q_{3}q_{44} - 2304q_{4}q_{43} - 1152q_{5}q_{42} - 1152q_{6}q_{41} -$
$2304q_{7}q_{40} - 21090816q_{8}q_{39} - 21090816q_{9}q_{38} - 2304q_{10}q_{37} - 4608q_{11}q_{36} - 3456q_{12}q_{35} - 3456q_{13}q_{34} -$
$2304q_{14}q_{33} - 1152q_{15}q_{32} - 1152q_{16}q_{31} - 2304q_{17}q_{30} - 4608q_{18}q_{29} - 4608q_{19}q_{28} + 6912q_{20}q_{27} - 2304q_{0}q_{48} -$
$4608q_{1}q_{47} - 3456q_{2}q_{46} - 4608q_{3}q_{45} - 3456q_{4}q_{44} - 1152q_{5}q_{43} - 2304q_{6}q_{42} - 2304q_{7}q_{41} - 2304q_{8}q_{40} -$
$21090816q_{9}q_{39} - 2304q_{10}q_{38} - 4608q_{11}q_{37} - 3456q_{12}q_{36} - 4608q_{13}q_{35} - 3456q_{14}q_{34} - 1152q_{15}q_{33} -$
$2304q_{16}q_{32} - 2304q_{17}q_{31} - 2304q_{18}q_{30} - 3456q_{19}q_{29} - 2304q_{0}q_{49} - 3456q_{1}q_{48} - 3456q_{2}q_{47} - 3456q_{3}q_{46} -$
$4608q_{4}q_{45} - 2304q_{5}q_{44} - 2304q_{6}q_{43} - 2304q_{7}q_{42} - 2304q_{8}q_{41} - 1152q_{9}q_{40} - 2304q_{10}q_{39} - 3456q_{11}q_{38} -$
$3456q_{12}q_{37} - 3456q_{13}q_{36} - 4608q_{14}q_{35} - 2304q_{15}q_{34} - 2304q_{16}q_{33} - 2304q_{17}q_{32} - 2304q_{18}q_{31} - 1152q_{19}q_{30} +$
$6912q_{20}q_{29} - 1152q_{0}q_{50} - 3456q_{1}q_{49} - 4608q_{2}q_{48} - 4608q_{3}q_{47} - 4608q_{4}q_{46} - 2304q_{5}q_{45} - 4608q_{6}q_{44} -$
$1152q_{7}q_{43} - 1152q_{8}q_{42} - 2304q_{9}q_{41} - 21090816q_{10}q_{40} - 3456q_{11}q_{39} - 4608q_{12}q_{38} - 4608q_{13}q_{37} -$
$4608q_{14}q_{36} - 2304q_{15}q_{35} - 4608q_{16}q_{34} - 1152q_{17}q_{33} - 1152q_{18}q_{32} - 2304q_{19}q_{31} + 2304q_{20}q_{30} + 6912q_{21}q_{29} -$
$1152q_{0}q_{51} - 2304q_{1}q_{50} - 4608q_{2}q_{49} - 3456q_{3}q_{48} - 3456q_{4}q_{47} - 2304q_{5}q_{46} - 3456q_{6}q_{45} - 4608q_{7}q_{44} -$
$2304q_{8}q_{43} - 2304q_{9}q_{42} - 21090816q_{10}q_{41} - 21090816q_{11}q_{40} - 4608q_{12}q_{39} - 3456q_{13}q_{38} - 3456q_{14}q_{37} -$

$2304q_{15}q_{36} - 3456q_{16}q_{35} - 4608q_{17}q_{34} - 2304q_{18}q_{33} - 2304q_{19}q_{32} - 1152q_{0}q_{52} - 1152q_{1}q_{51} - 2304q_{2}q_{50} -$
$4608q_{3}q_{49} - 4608q_{4}q_{48} - 2304q_{5}q_{47} - 4608q_{6}q_{46} - 3456q_{7}q_{45} - 3456q_{8}q_{44} - 2304q_{9}q_{43} - 21090816q_{10}q_{42} -$
$3456q_{12}q_{40} - 4608q_{13}q_{39} - 4608q_{14}q_{38} - 2304q_{15}q_{37} - 4608q_{16}q_{36} - 3456q_{17}q_{35} - 3456q_{18}q_{34} - 2304q_{19}q_{33} +$
$2304q_{21}q_{31} - 1152q_{0}q_{53} - 2304q_{1}q_{52} - 2304q_{2}q_{51} - 2304q_{3}q_{50} - 3456q_{4}q_{49} - 2304q_{5}q_{48} - 4608q_{6}q_{47} -$
$3456q_{7}q_{46} - 4608q_{8}q_{45} - 3456q_{9}q_{44} - 21090816q_{10}q_{43} - 21090816q_{12}q_{41} - 6912q_{13}q_{40} - 3456q_{14}q_{39} -$
$2304q_{15}q_{38} - 4608q_{16}q_{37} - 3456q_{17}q_{36} - 4608q_{18}q_{35} - 3456q_{19}q_{34} + 6912q_{24}q_{29} - 2304q_{0}q_{54} - 2304q_{1}q_{53} -$
$2304q_{2}q_{52} - 2304q_{3}q_{51} - 1152q_{4}q_{50} - 2304q_{5}q_{49} - 3456q_{6}q_{48} - 3456q_{7}q_{47} - 3456q_{8}q_{46} - 4608q_{9}q_{45} -$
$6912q_{13}q_{41} - 3456q_{14}q_{40} - 2304q_{15}q_{39} - 3456q_{16}q_{38} - 3456q_{17}q_{37} - 3456q_{18}q_{36} - 4608q_{19}q_{35} + 2304q_{20}q_{34} +$
$2304q_{22}q_{32} + 2304q_{24}q_{30} - 2304q_{0}q_{55} - 4608q_{1}q_{54} - 1152q_{2}q_{53} - 1152q_{3}q_{52} - 2304q_{4}q_{51} - 1152q_{5}q_{50} -$
$3456q_{6}q_{49} - 4608q_{7}q_{48} - 4608q_{8}q_{47} - 4608q_{9}q_{46} - 21090816q_{11}q_{44} - 21090816q_{13}q_{42} - 3456q_{14}q_{41} -$
$1152q_{15}q_{40} - 3456q_{16}q_{39} - 4608q_{17}q_{38} - 4608q_{18}q_{37} - 4608q_{19}q_{36} - 2304q_{0}q_{56} - 3456q_{1}q_{55} - 4608q_{2}q_{54} -$
$2304q_{3}q_{53} - 2304q_{4}q_{52} - 1152q_{5}q_{51} - 2304q_{6}q_{50} - 4608q_{7}q_{49} - 3456q_{8}q_{48} - 3456q_{9}q_{47} - 21090816q_{11}q_{45} -$
$21090816q_{12}q_{44} - 6912q_{14}q_{42} - 1152q_{15}q_{41} - 2304q_{16}q_{40} - 4608q_{17}q_{39} - 3456q_{18}q_{38} - 3456q_{19}q_{37} +$
$2304q_{21}q_{35} + 2304q_{22}q_{34} + 2304q_{23}q_{33} + 2304q_{24}q_{32} + 2304q_{25}q_{31} - 2304q_{0}q_{57} - 4608q_{1}q_{56} - 3456q_{2}q_{55} -$
$3456q_{3}q_{54} - 2304q_{4}q_{53} - 1152q_{5}q_{52} - 1152q_{6}q_{51} - 2304q_{7}q_{50} - 4608q_{8}q_{49} - 4608q_{9}q_{48} - 21090816q_{11}q_{46} -$
$6912q_{13}q_{44} - 21090816q_{14}q_{43} - 1152q_{15}q_{42} - 1152q_{16}q_{41} - 2304q_{17}q_{40} - 4608q_{18}q_{39} - 4608q_{19}q_{38} +$
$2304q_{20}q_{37} + 2304q_{27}q_{30} - 2304q_{0}q_{58} - 4608q_{1}q_{57} - 3456q_{2}q_{56} - 4608q_{3}q_{55} - 3456q_{4}q_{54} - 1152q_{5}q_{53} -$
$2304q_{6}q_{52} - 2304q_{7}q_{51} - 2304q_{8}q_{50} - 3456q_{9}q_{49} - 21090816q_{13}q_{45} - 3456q_{14}q_{44} - 1152q_{15}q_{43} - 2304q_{16}q_{42} -$
$2304q_{17}q_{41} - 2304q_{18}q_{40} - 3456q_{19}q_{39} + 2304q_{22}q_{36} + 2304q_{23}q_{35} + 4608q_{24}q_{34} + 2304q_{25}q_{33} + 2304q_{26}q_{32} -$
$2304q_{0}q_{59} - 3456q_{1}q_{58} - 3456q_{2}q_{57} - 3456q_{3}q_{56} - 4608q_{4}q_{55} - 2304q_{5}q_{54} - 2304q_{6}q_{53} - 2304q_{7}q_{52} -$
$2304q_{8}q_{51} - 1152q_{9}q_{50} - 21090816q_{12}q_{47} - 6912q_{14}q_{45} - 2304q_{15}q_{44} - 2304q_{16}q_{43} - 2304q_{17}q_{42} -$
$2304q_{18}q_{41} - 1152q_{19}q_{40} + 2304q_{20}q_{39} + 2304q_{21}q_{38} + 2304q_{23}q_{36} + 2304q_{26}q_{33} + 2304q_{28}q_{31} + 2304q_{29}q_{30} -$
$3456q_{1}q_{59} - 4608q_{2}q_{58} - 4608q_{3}q_{57} - 4608q_{4}q_{56} - 2304q_{5}q_{55} - 4608q_{6}q_{54} - 1152q_{7}q_{53} - 1152q_{8}q_{52} -$
$2304q_{9}q_{51} - 1152q_{10}q_{50} - 21090816q_{12}q_{48} - 21090816q_{13}q_{47} - 21090816q_{14}q_{46} - 2304q_{15}q_{45} - 4608q_{16}q_{44} -$
$1152q_{17}q_{43} - 1152q_{18}q_{42} - 2304q_{19}q_{41} + 2304q_{20}q_{40} + 2304q_{21}q_{39} + 2304q_{22}q_{38} + 2304q_{23}q_{37} + 2304q_{24}q_{36} +$
$4608q_{25}q_{35} + 2304q_{26}q_{34} + 2304q_{27}q_{33} + 2304q_{28}q_{32} + 2304q_{29}q_{31} - 4608q_{2}q_{59} - 3456q_{3}q_{58} - 3456q_{4}q_{57} -$
$2304q_{5}q_{56} - 3456q_{6}q_{55} - 4608q_{7}q_{54} - 2304q_{8}q_{53} - 2304q_{9}q_{52} - 1152q_{10}q_{51} - 2304q_{11}q_{50} - 6912q_{14}q_{47} -$
$2304q_{15}q_{46} - 3456q_{16}q_{45} - 4608q_{17}q_{44} - 2304q_{18}q_{43} - 2304q_{19}q_{42} + 2304q_{24}q_{37} + 2304q_{25}q_{36} + 2304q_{26}q_{35} +$
$2304q_{27}q_{34} - 4608q_{3}q_{59} - 4608q_{4}q_{58} - 2304q_{5}q_{57} - 4608q_{6}q_{56} - 3456q_{7}q_{55} - 3456q_{8}q_{54} - 2304q_{9}q_{53} -$
$1152q_{10}q_{52} - 1152q_{11}q_{51} - 2304q_{12}q_{50} - 21090816q_{13}q_{49} - 21090816q_{14}q_{48} - 2304q_{15}q_{47} - 4608q_{16}q_{46} -$
$3456q_{17}q_{45} - 3456q_{18}q_{44} - 2304q_{19}q_{43} + 2304q_{21}q_{41} + 2304q_{24}q_{38} + 2304q_{25}q_{37} + 4608q_{26}q_{36} + 2304q_{27}q_{35} +$
$2304q_{28}q_{34} - 3456q_{4}q_{59} - 2304q_{5}q_{58} - 4608q_{6}q_{57} - 3456q_{7}q_{56} - 4608q_{8}q_{55} - 3456q_{9}q_{54} - 1152q_{10}q_{53} -$
$2304q_{11}q_{52} - 2304q_{12}q_{51} - 2304q_{13}q_{50} - 21090816q_{14}q_{49} - 2304q_{15}q_{48} - 4608q_{16}q_{47} - 3456q_{17}q_{46} -$
$4608q_{18}q_{45} - 3456q_{19}q_{44} + 2304q_{24}q_{39} + 2304q_{25}q_{38} + 2304q_{26}q_{37} + 2304q_{27}q_{36} + 2304q_{28}q_{35} + 2304q_{29}q_{34} -$
$2304q_{5}q_{59} - 3456q_{6}q_{58} - 3456q_{7}q_{57} - 3456q_{8}q_{56} - 4608q_{9}q_{55} - 2304q_{10}q_{54} - 2304q_{11}q_{53} - 2304q_{12}q_{52} -$
$2304q_{13}q_{51} - 1152q_{14}q_{50} - 2304q_{15}q_{49} - 3456q_{16}q_{48} - 3456q_{17}q_{47} - 3456q_{18}q_{46} - 4608q_{19}q_{45} + 2304q_{20}q_{44} +$
$2304q_{22}q_{42} + 2304q_{24}q_{40} + 2304q_{25}q_{39} + 2304q_{26}q_{38} + 4608q_{27}q_{37} + 2304q_{28}q_{36} + 2304q_{29}q_{35} - 3456q_{6}q_{59} -$
$4608q_{7}q_{58} - 4608q_{8}q_{57} - 4608q_{9}q_{56} - 2304q_{10}q_{55} - 4608q_{11}q_{54} - 1152q_{12}q_{53} - 1152q_{13}q_{52} - 2304q_{14}q_{51} -$
$21090816q_{15}q_{50} - 3456q_{16}q_{49} - 4608q_{17}q_{48} - 4608q_{18}q_{47} - 4608q_{19}q_{46} - 4608q_{7}q_{59} - 3456q_{8}q_{58} -$
$3456q_{9}q_{57} - 2304q_{10}q_{56} - 3456q_{11}q_{55} - 4608q_{12}q_{54} - 2304q_{13}q_{53} - 2304q_{14}q_{52} - 21090816q_{15}q_{51} -$
$21090816q_{16}q_{50} - 4608q_{17}q_{49} - 3456q_{18}q_{48} - 3456q_{19}q_{47} + 2304q_{21}q_{45} + 2304q_{22}q_{44} + 2304q_{23}q_{43} +$
$2304q_{24}q_{42} + 2304q_{25}q_{41} + 2304q_{27}q_{39} + 4608q_{28}q_{38} + 2304q_{29}q_{37} - 4608q_{8}q_{59} - 4608q_{9}q_{58} - 2304q_{10}q_{57} -$
$4608q_{11}q_{56} - 3456q_{12}q_{55} - 3456q_{13}q_{54} - 2304q_{14}q_{53} - 21090816q_{15}q_{52} - 3456q_{17}q_{50} - 4608q_{18}q_{49} -$
$4608q_{19}q_{48} + 2304q_{20}q_{47} + 2304q_{27}q_{40} + 2304q_{28}q_{39} + 2304q_{29}q_{38} + 6912q_{30}q_{37} - 3456q_{9}q_{59} - 2304q_{10}q_{58} -$
$4608q_{11}q_{57} - 3456q_{12}q_{56} - 4608q_{13}q_{55} - 3456q_{14}q_{54} - 21090816q_{15}q_{53} - 21090816q_{17}q_{51} - 6912q_{18}q_{50} -$
$3456q_{19}q_{49} + 2304q_{22}q_{46} + 2304q_{23}q_{45} + 4608q_{24}q_{44} + 2304q_{25}q_{43} + 2304q_{26}q_{42} + 4608q_{29}q_{39} - 2304q_{10}q_{59} -$
$3456q_{11}q_{58} - 3456q_{12}q_{57} - 3456q_{13}q_{56} - 4608q_{14}q_{55} - 6912q_{18}q_{51} - 3456q_{19}q_{50} + 2304q_{20}q_{49} + 2304q_{21}q_{48} +$
$2304q_{23}q_{46} + 2304q_{26}q_{43} + 2304q_{28}q_{41} + 2304q_{29}q_{40} + 6912q_{30}q_{39} - 3456q_{11}q_{59} - 4608q_{12}q_{58} - 4608q_{13}q_{57} -$

$4608q_{14}q_{56} - 21090816q_{16}q_{54} - 21090816q_{18}q_{52} - 3456q_{19}q_{51} + 2304q_{20}q_{50} + 2304q_{21}q_{49} + 2304q_{22}q_{48} +$
$2304q_{23}q_{47} + 2304q_{24}q_{46} + 4608q_{25}q_{45} + 2304q_{26}q_{44} + 2304q_{27}q_{43} + 2304q_{28}q_{42} + 2304q_{29}q_{41} + 2304q_{30}q_{40} +$
$6912q_{31}q_{39} - 4608q_{12}q_{59} - 3456q_{13}q_{58} - 3456q_{14}q_{57} - 21090816q_{16}q_{55} - 21090816q_{17}q_{54} - 6912q_{19}q_{52} +$
$2304q_{24}q_{47} + 2304q_{25}q_{46} + 2304q_{26}q_{45} + 2304q_{27}q_{44} - 4608q_{13}q_{59} - 4608q_{14}q_{58} - 21090816q_{16}q_{56} -$
$6912q_{18}q_{54} - 21090816q_{19}q_{53} + 2304q_{21}q_{51} + 2304q_{24}q_{48} + 2304q_{25}q_{47} + 4608q_{26}q_{46} + 2304q_{27}q_{45} +$
$2304q_{28}q_{44} + 2304q_{31}q_{41} - 3456q_{14}q_{59} - 21090816q_{18}q_{55} - 3456q_{19}q_{54} + 2304q_{24}q_{49} + 2304q_{25}q_{48} +$
$2304q_{26}q_{47} + 2304q_{27}q_{46} + 2304q_{28}q_{45} + 2304q_{29}q_{44} + 6912q_{34}q_{39} - 21090816q_{17}q_{57} - 6912q_{19}q_{55} +$
$2304q_{20}q_{54} + 2304q_{22}q_{52} + 2304q_{24}q_{50} + 2304q_{25}q_{49} + 2304q_{26}q_{48} + 4608q_{27}q_{47} + 2304q_{28}q_{46} + 2304q_{29}q_{45} +$
$2304q_{30}q_{44} + 2304q_{32}q_{42} + 2304q_{34}q_{40} - 21090816q_{17}q_{58} - 21090816q_{18}q_{57} - 21090816q_{19}q_{56} - 6912q_{19}q_{57} +$
$2304q_{21}q_{55} + 2304q_{22}q_{54} + 2304q_{23}q_{53} + 2304q_{24}q_{52} + 2304q_{25}q_{51} + 2304q_{27}q_{49} + 4608q_{28}q_{48} + 2304q_{29}q_{47} +$
$2304q_{31}q_{45} + 2304q_{32}q_{44} + 2304q_{33}q_{43} + 2304q_{34}q_{42} + 2304q_{35}q_{41} - 21090816q_{18}q_{59} - 21090816q_{19}q_{58} +$
$2304q_{20}q_{57} + 2304q_{27}q_{50} + 2304q_{28}q_{49} + 2304q_{29}q_{48} + 2304q_{30}q_{47} + 2304q_{37}q_{40} - 21090816q_{19}q_{59} +$
$2304q_{22}q_{56} + 2304q_{23}q_{55} + 4608q_{24}q_{54} + 2304q_{25}q_{53} + 2304q_{26}q_{52} + 4608q_{29}q_{49} + 2304q_{32}q_{46} + 2304q_{33}q_{45} +$
$4608q_{34}q_{44} + 2304q_{35}q_{43} + 2304q_{36}q_{42} + 2304q_{20}q_{59} + 2304q_{21}q_{58} + 2304q_{23}q_{56} + 2304q_{26}q_{53} + 2304q_{28}q_{51} +$
$2304q_{29}q_{50} + 2304q_{30}q_{49} + 2304q_{31}q_{48} + 2304q_{33}q_{46} + 2304q_{36}q_{43} + 2304q_{38}q_{41} + 2304q_{39}q_{40} + 2304q_{21}q_{59} +$
$2304q_{22}q_{58} + 2304q_{23}q_{57} + 2304q_{24}q_{56} + 4608q_{25}q_{55} + 2304q_{26}q_{54} + 2304q_{27}q_{53} + 2304q_{28}q_{52} + 2304q_{29}q_{51} +$
$2304q_{30}q_{50} + 2304q_{31}q_{49} + 2304q_{32}q_{48} + 2304q_{33}q_{47} + 2304q_{34}q_{46} + 4608q_{35}q_{45} + 2304q_{36}q_{44} + 2304q_{37}q_{43} +$
$2304q_{38}q_{42} + 2304q_{39}q_{41} + 2304q_{24}q_{57} + 2304q_{25}q_{56} + 2304q_{26}q_{55} + 2304q_{27}q_{54} + 2304q_{34}q_{47} + 2304q_{35}q_{46} +$
$2304q_{36}q_{45} + 2304q_{37}q_{44} + 2304q_{24}q_{58} + 2304q_{25}q_{57} + 4608q_{26}q_{56} + 2304q_{27}q_{55} + 2304q_{28}q_{54} + 2304q_{31}q_{51} +$
$2304q_{34}q_{48} + 2304q_{35}q_{47} + 4608q_{36}q_{46} + 2304q_{37}q_{45} + 2304q_{38}q_{44} + 2304q_{24}q_{59} + 2304q_{25}q_{58} + 2304q_{26}q_{57} +$
$2304q_{27}q_{56} + 2304q_{28}q_{55} + 2304q_{29}q_{54} + 2304q_{34}q_{49} + 2304q_{35}q_{48} + 2304q_{36}q_{47} + 2304q_{37}q_{46} + 2304q_{38}q_{45} +$
$2304q_{39}q_{44} + 2304q_{25}q_{59} + 2304q_{26}q_{58} + 4608q_{27}q_{57} + 2304q_{28}q_{56} + 2304q_{29}q_{55} + 2304q_{30}q_{54} + 2304q_{32}q_{52} +$
$2304q_{34}q_{50} + 2304q_{35}q_{49} + 2304q_{36}q_{48} + 4608q_{37}q_{47} + 2304q_{38}q_{46} + 2304q_{39}q_{45} + 2304q_{27}q_{59} + 4608q_{28}q_{58} +$
$2304q_{29}q_{57} + 2304q_{31}q_{55} + 2304q_{32}q_{54} + 2304q_{33}q_{53} + 2304q_{34}q_{52} + 2304q_{35}q_{51} + 2304q_{37}q_{49} + 4608q_{38}q_{48} +$
$2304q_{39}q_{47} + 2304q_{28}q_{59} + 2304q_{29}q_{58} + 2304q_{30}q_{57} + 2304q_{37}q_{50} + 2304q_{38}q_{49} + 2304q_{39}q_{48} + 6912q_{40}q_{47} +$
$4608q_{29}q_{59} + 2304q_{32}q_{56} + 2304q_{33}q_{55} + 4608q_{34}q_{54} + 2304q_{35}q_{53} + 2304q_{36}q_{52} + 4608q_{39}q_{49} + 2304q_{30}q_{59} +$
$2304q_{31}q_{58} + 2304q_{33}q_{56} + 2304q_{36}q_{53} + 2304q_{38}q_{51} + 2304q_{39}q_{50} + 6912q_{40}q_{49} + 2304q_{31}q_{59} + 2304q_{32}q_{58} +$
$2304q_{33}q_{57} + 2304q_{34}q_{56} + 4608q_{35}q_{55} + 2304q_{36}q_{54} + 2304q_{37}q_{53} + 2304q_{38}q_{52} + 2304q_{39}q_{51} + 2304q_{40}q_{50} +$
$6912q_{41}q_{49} + 2304q_{34}q_{57} + 2304q_{35}q_{56} + 2304q_{36}q_{55} + 2304q_{37}q_{54} + 2304q_{34}q_{58} + 2304q_{35}q_{57} + 4608q_{36}q_{56} +$
$2304q_{37}q_{55} + 2304q_{38}q_{54} + 2304q_{41}q_{51} + 2304q_{34}q_{59} + 2304q_{35}q_{58} + 2304q_{36}q_{57} + 2304q_{37}q_{56} + 2304q_{38}q_{55} +$
$2304q_{39}q_{54} + 6912q_{44}q_{49} + 2304q_{35}q_{59} + 2304q_{36}q_{58} + 4608q_{37}q_{57} + 2304q_{38}q_{56} + 2304q_{39}q_{55} + 2304q_{40}q_{54} +$
$2304q_{42}q_{52} + 2304q_{44}q_{50} + 2304q_{37}q_{59} + 4608q_{38}q_{58} + 2304q_{39}q_{57} + 2304q_{41}q_{55} + 2304q_{42}q_{54} + 2304q_{43}q_{53} +$
$2304q_{44}q_{52} + 2304q_{45}q_{51} + 2304q_{38}q_{59} + 2304q_{39}q_{58} + 2304q_{40}q_{57} + 2304q_{47}q_{50} + 4608q_{39}q_{59} + 2304q_{42}q_{56} +$
$2304q_{43}q_{55} + 4608q_{44}q_{54} + 2304q_{45}q_{53} + 2304q_{46}q_{52} + 2304q_{40}q_{59} + 2304q_{41}q_{58} + 2304q_{43}q_{56} + 2304q_{46}q_{53} +$
$2304q_{48}q_{51} + 2304q_{49}q_{50} + 2304q_{41}q_{59} + 2304q_{42}q_{58} + 2304q_{43}q_{57} + 2304q_{44}q_{56} + 4608q_{45}q_{55} + 2304q_{46}q_{54} +$
$2304q_{47}q_{53} + 2304q_{48}q_{52} + 2304q_{49}q_{51} + 2304q_{44}q_{57} + 2304q_{45}q_{56} + 2304q_{46}q_{55} + 2304q_{47}q_{54} + 2304q_{44}q_{58} +$
$2304q_{45}q_{57} + 4608q_{46}q_{56} + 2304q_{47}q_{55} + 2304q_{48}q_{54} + 2304q_{44}q_{59} + 2304q_{45}q_{58} + 2304q_{46}q_{57} + 2304q_{47}q_{56} +$
$2304q_{48}q_{55} + 2304q_{49}q_{54} + 2304q_{45}q_{59} + 2304q_{46}q_{58} + 4608q_{47}q_{57} + 2304q_{48}q_{56} + 2304q_{49}q_{55} + 2304q_{47}q_{59} +$
$4608q_{48}q_{58} + 2304q_{49}q_{57} + 2304q_{48}q_{59} + 2304q_{49}q_{58} + 6912q_{50}q_{57} + 4608q_{49}q_{59} + 6912q_{50}q_{59} + 6912q_{51}q_{59} +$
$6912q_{54}q_{59} + 93312$

A complete expression of $E_2(s)$ for 36-order Williamson based method or 108-order Baumert-Hall based method

$E_2(s) = 10555200s_0 + 10561680s_1 + 10562544s_2 + 10566864s_3 + 10569456s_4 + 10555200s_5 + 10561680s_6 +$
$10562544s_7 + 10566864s_8 + 10569456s_9 + 10555200s_{10} + 10561680s_{11} + 10562544s_{12} + 10566864s_{13} +$
$10569456s_{14} + 10555200s_{15} + 10561680s_{16} + 10562544s_{17} + 10566864s_{18} + 10569456s_{19} - 5278752s_{20} -$
$5277888s_{21} - 5277600s_{22} - 5278752s_{23} - 5282208s_{24} - 5281920s_{25} - 5283072s_{26} - 5283648s_{27} - 5283072s_{28} -$
$5288832s_{29} - 5278752s_{30} - 5277888s_{31} - 5277600s_{32} - 5278752s_{33} - 5282208s_{34} - 5281920s_{35} - 5283072s_{36} -$

$5283648s_{37} - 5283072s_{38} - 5288832s_{39} - 5278752s_{40} - 5277888s_{41} - 5277600s_{42} - 5278752s_{43} - 5282208s_{44} -$
$5281920s_{45} - 5283072s_{46} - 5283648s_{47} - 5283072s_{48} - 5288832s_{49} - 5278752s_{50} - 5277888s_{51} - 5277600s_{52} -$
$5278752s_{53} - 5282208s_{54} - 5281920s_{55} - 5283072s_{56} - 5283648s_{57} - 5283072s_{58} - 5288832s_{59} + 2636352s_0s_1 +$
$2636352s_0s_2 + 2636352s_0s_3 + 2636352s_1s_2 + 2636352s_0s_4 + 2636352s_1s_3 + 576s_0s_5 + 2636352s_1s_4 +$
$2636352s_2s_3 + 1008s_0s_6 + 1008s_1s_5 + 2636352s_2s_4 + 1008s_0s_7 + 1872s_1s_6 + 1008s_2s_5 + 2636352s_3s_4 + 1008s_0s_8 +$
$1728s_1s_7 + 1728s_2s_6 + 1008s_3s_5 + 1008s_0s_9 + 1728s_1s_8 + 1872s_2s_7 + 1728s_3s_6 + 1008s_4s_5 + 576s_0s_{10} + 1728s_1s_9 +$
$1728s_2s_8 + 1728s_3s_7 + 1728s_4s_6 + 1008s_0s_{11} + 1008s_1s_{10} + 1728s_2s_9 + 1872s_3s_8 + 1728s_4s_7 + 2636352s_5s_6 +$
$1008s_0s_{12} + 1872s_1s_{11} + 1008s_2s_{10} + 1728s_3s_9 + 1728s_4s_8 + 2636352s_5s_7 + 1008s_0s_{13} + 1728s_1s_{12} + 1728s_2s_{11} +$
$1008s_3s_{10} + 1872s_4s_9 + 2636352s_5s_8 + 2636352s_6s_7 + 1008s_0s_{14} + 1728s_1s_{13} + 1872s_2s_{12} + 1728s_3s_{11} +$
$1008s_4s_{10} + 2636352s_5s_9 + 2636352s_6s_8 + 576s_0s_{15} + 1728s_1s_{14} + 1728s_2s_{13} + 1728s_3s_{12} + 1728s_4s_{11} + 576s_5s_{10} +$
$2636352s_6s_9 + 2636352s_7s_8 + 1008s_0s_{16} + 1008s_1s_{15} + 1728s_2s_{14} + 1872s_3s_{13} + 1728s_4s_{12} + 1008s_5s_{11} +$
$1008s_6s_{10} + 2636352s_7s_9 + 1008s_0s_{17} + 1872s_1s_{16} + 1008s_2s_{15} + 1728s_3s_{14} + 1728s_4s_{13} + 1008s_5s_{12} + 1872s_6s_{11} +$
$1008s_7s_{10} + 2636352s_8s_9 + 1008s_0s_{18} + 1728s_1s_{17} + 1728s_2s_{16} + 1008s_3s_{15} + 1872s_4s_{14} + 1008s_5s_{13} + 1728s_6s_{12} +$
$1728s_7s_{11} + 1008s_8s_{10} + 1008s_0s_{19} + 1728s_1s_{18} + 1872s_2s_{17} + 1728s_3s_{16} + 1008s_4s_{15} + 1008s_5s_{14} + 1728s_6s_{13} +$
$1872s_7s_{12} + 1728s_8s_{11} + 1008s_9s_{10} - 5272704s_0s_{20} + 1728s_1s_{19} + 1728s_2s_{18} + 1728s_3s_{17} + 1728s_4s_{16} + 576s_5s_{15} +$
$1728s_6s_{14} + 1728s_7s_{13} + 1728s_8s_{12} + 1728s_9s_{11} - 5272704s_0s_{21} - 5272704s_1s_{20} + 1728s_2s_{19} + 1872s_3s_{18} +$
$1728s_4s_{17} + 1008s_5s_{16} + 1008s_6s_{15} + 1728s_7s_{14} + 1872s_8s_{13} + 1728s_9s_{12} + 2636352s_{10}s_{11} - 5272704s_0s_{22} -$
$864s_2s_{20} + 1728s_3s_{19} + 1728s_4s_{18} + 1008s_5s_{17} + 1872s_6s_{16} + 1008s_7s_{15} + 1728s_8s_{14} + 1728s_9s_{13} + 2636352s_{10}s_{12} -$
$5272704s_0s_{23} - 5272704s_2s_{21} - 1728s_3s_{20} + 1872s_4s_{19} + 1008s_5s_{18} + 1728s_6s_{17} + 1728s_7s_{16} + 1008s_8s_{15} +$
$1872s_9s_{14} + 2636352s_{10}s_{13} + 2636352s_{11}s_{12} - 1728s_3s_{21} - 864s_4s_{20} + 1008s_5s_{19} + 1728s_6s_{18} + 1872s_7s_{17} +$
$1728s_8s_{16} + 1008s_9s_{15} + 2636352s_{10}s_{14} + 2636352s_{11}s_{13} - 5272704s_1s_{24} - 5272704s_3s_{22} - 864s_4s_{21} - 288s_5s_{20} +$
$1728s_6s_{19} + 1728s_7s_{18} + 1728s_8s_{17} + 1728s_9s_{16} + 576s_{10}s_{15} + 2636352s_{11}s_{14} + 2636352s_{12}s_{13} - 5272704s_1s_{25} -$
$5272704s_2s_{24} - 1728s_4s_{22} - 288s_5s_{21} - 576s_6s_{20} + 1728s_7s_{19} + 1872s_8s_{18} + 1728s_9s_{17} + 1008s_{10}s_{16} + 1008s_{11}s_{15} +$
$2636352s_{12}s_{14} - 5272704s_1s_{26} - 1728s_3s_{24} - 5272704s_4s_{23} - 288s_5s_{22} - 288s_6s_{21} - 576s_7s_{20} + 1728s_8s_{19} +$
$1728s_9s_{18} + 1008s_{10}s_{17} + 1872s_{11}s_{16} + 1008s_{12}s_{15} + 2636352s_{13}s_{14} - 5272704s_3s_{25} - 864s_4s_{24} - 288s_5s_{23} -$
$576s_6s_{22} - 576s_7s_{21} - 576s_8s_{20} + 1872s_9s_{19} + 1008s_{10}s_{18} + 1728s_{11}s_{17} + 1728s_{12}s_{16} + 1008s_{13}s_{15} - 5272704s_2s_{27} -$
$1728s_4s_{25} - 576s_5s_{24} - 576s_6s_{23} - 576s_7s_{22} - 576s_8s_{21} - 288s_9s_{20} + 1008s_{10}s_{19} + 1728s_{11}s_{18} + 1872s_{12}s_{17} +$
$1728s_{13}s_{16} + 1008s_{14}s_{15} - 288s_0s_{30} - 5272704s_2s_{28} - 5272704s_3s_{27} - 5272704s_4s_{26} - 576s_5s_{25} - 1152s_6s_{24} -$
$288s_7s_{23} - 288s_8s_{22} - 576s_9s_{21} - 288s_{10}s_{20} + 1728s_{11}s_{19} + 1728s_{12}s_{18} + 1728s_{13}s_{17} + 1728s_{14}s_{16} - 288s_0s_{31} -$
$576s_1s_{30} - 1728s_4s_{27} - 576s_5s_{26} - 864s_6s_{25} - 1152s_7s_{24} - 576s_8s_{23} - 576s_9s_{22} - 288s_{10}s_{21} - 576s_{11}s_{20} +$
$1728s_{12}s_{19} + 1872s_{13}s_{18} + 1728s_{14}s_{17} + 2636352s_{15}s_{16} - 288s_0s_{32} - 288s_1s_{31} - 576s_2s_{30} - 5272704s_3s_{29} -$
$5272704s_4s_{28} - 576s_5s_{27} - 1152s_6s_{26} - 864s_7s_{25} - 864s_8s_{24} - 576s_9s_{23} - 288s_{10}s_{22} - 288s_{11}s_{21} - 576s_{12}s_{20} +$
$1728s_{13}s_{19} + 1728s_{14}s_{18} + 2636352s_{15}s_{17} - 288s_0s_{33} - 576s_1s_{32} - 576s_2s_{31} - 576s_3s_{30} - 5272704s_4s_{29} -$
$576s_5s_{28} - 1152s_6s_{27} - 864s_7s_{26} - 1152s_8s_{25} - 864s_9s_{24} - 288s_{10}s_{23} - 576s_{11}s_{22} - 576s_{12}s_{21} - 576s_{13}s_{20} +$
$1872s_{14}s_{19} + 2636352s_{15}s_{18} + 2636352s_{16}s_{17} - 576s_0s_{34} - 576s_1s_{33} - 576s_2s_{32} - 576s_3s_{31} - 288s_4s_{30} - 576s_5s_{29} -$
$864s_6s_{28} - 864s_7s_{27} - 864s_8s_{26} - 1152s_9s_{25} - 576s_{10}s_{24} - 576s_{11}s_{23} - 576s_{12}s_{22} - 576s_{13}s_{21} - 288s_{14}s_{20} +$
$2636352s_{15}s_{19} + 2636352s_{16}s_{18} - 576s_0s_{35} - 1152s_1s_{34} - 288s_2s_{33} - 288s_3s_{32} - 576s_4s_{31} - 5272704s_5s_{30} -$
$864s_6s_{29} - 1152s_7s_{28} - 1152s_8s_{27} - 1152s_9s_{26} - 576s_{10}s_{25} - 1152s_{11}s_{24} - 288s_{12}s_{23} - 288s_{13}s_{22} - 576s_{14}s_{21} -$
$288s_{15}s_{20} + 2636352s_{16}s_{19} + 2636352s_{17}s_{18} - 576s_0s_{36} - 864s_1s_{35} - 1152s_2s_{34} - 576s_3s_{33} - 576s_4s_{32} -$
$5272704s_5s_{31} - 5272704s_6s_{30} - 1152s_7s_{29} - 864s_8s_{28} - 864s_9s_{27} - 576s_{10}s_{26} - 864s_{11}s_{25} - 1152s_{12}s_{24} -$
$576s_{13}s_{23} - 576s_{14}s_{22} - 288s_{15}s_{21} - 576s_{16}s_{20} + 2636352s_{17}s_{19} - 576s_0s_{37} - 1152s_1s_{36} - 864s_2s_{35} - 864s_3s_{34} -$
$576s_4s_{33} - 5272704s_5s_{32} - 864s_7s_{30} - 1152s_8s_{29} - 1152s_9s_{28} - 576s_{10}s_{27} - 1152s_{11}s_{26} - 864s_{12}s_{25} - 864s_{13}s_{24} -$
$576s_{14}s_{23} - 288s_{15}s_{22} - 288s_{16}s_{21} - 576s_{17}s_{20} + 2636352s_{18}s_{19} - 576s_0s_{38} - 1152s_1s_{37} - 864s_2s_{36} - 1152s_3s_{35} -$
$864s_4s_{34} - 5272704s_5s_{33} - 5272704s_7s_{31} - 1728s_8s_{30} - 864s_9s_{29} - 576s_{10}s_{28} - 1152s_{11}s_{27} - 864s_{12}s_{26} -$
$1152s_{13}s_{25} - 864s_{14}s_{24} - 288s_{15}s_{23} - 576s_{16}s_{22} - 576s_{17}s_{21} - 576s_{18}s_{20} - 576s_0s_{39} - 864s_1s_{38} - 864s_2s_{37} -$
$864s_3s_{36} - 1152s_4s_{35} - 1728s_8s_{31} - 864s_9s_{30} - 576s_{10}s_{29} - 864s_{11}s_{28} - 864s_{12}s_{27} - 864s_{13}s_{26} - 1152s_{14}s_{25} -$
$576s_{15}s_{24} - 576s_{16}s_{23} - 576s_{17}s_{22} - 576s_{18}s_{21} - 288s_{19}s_{20} - 288s_0s_{40} - 864s_1s_{39} - 1152s_2s_{38} - 1152s_3s_{37} -$
$1152s_4s_{36} - 5272704s_6s_{34} - 5272704s_8s_{32} - 864s_9s_{31} - 288s_{10}s_{30} - 864s_{11}s_{29} - 1152s_{12}s_{28} - 1152s_{13}s_{27} -$

$1152s_{14}s_{26} - 576s_{15}s_{25} - 1152s_{16}s_{24} - 288s_{17}s_{23} - 288s_{18}s_{22} - 576s_{19}s_{21} - 288s_{0}s_{41} - 576s_{1}s_{40} - 1152s_{2}s_{39} -$
$864s_{3}s_{38} - 864s_{4}s_{37} - 5272704s_{6}s_{35} - 5272704s_{7}s_{34} - 1728s_{9}s_{32} - 288s_{10}s_{31} - 576s_{11}s_{30} - 1152s_{12}s_{29} -$
$864s_{13}s_{28} - 864s_{14}s_{27} - 576s_{15}s_{26} - 864s_{16}s_{25} - 1152s_{17}s_{24} - 576s_{18}s_{23} - 576s_{19}s_{22} - 288s_{0}s_{42} - 288s_{1}s_{41} -$
$576s_{2}s_{40} - 1152s_{3}s_{39} - 1152s_{4}s_{38} - 5272704s_{6}s_{36} - 1728s_{8}s_{34} - 5272704s_{9}s_{33} - 288s_{10}s_{32} - 288s_{11}s_{31} -$
$576s_{12}s_{30} - 1152s_{13}s_{29} - 1152s_{14}s_{28} - 576s_{15}s_{27} - 1152s_{16}s_{26} - 864s_{17}s_{25} - 864s_{18}s_{24} - 576s_{19}s_{23} - 288s_{0}s_{43} -$
$576s_{1}s_{42} - 576s_{2}s_{41} - 576s_{3}s_{40} - 864s_{4}s_{39} - 5272704s_{8}s_{35} - 864s_{9}s_{34} - 288s_{10}s_{33} - 576s_{11}s_{32} - 576s_{12}s_{31} -$
$576s_{13}s_{30} - 864s_{14}s_{29} - 576s_{15}s_{28} - 1152s_{16}s_{27} - 864s_{17}s_{26} - 1152s_{18}s_{25} - 864s_{19}s_{24} - 576s_{0}s_{44} - 576s_{1}s_{43} -$
$576s_{2}s_{42} - 576s_{3}s_{41} - 288s_{4}s_{40} - 5272704s_{7}s_{37} - 1728s_{9}s_{35} - 576s_{10}s_{34} - 576s_{11}s_{33} - 576s_{12}s_{32} - 576s_{13}s_{31} -$
$288s_{14}s_{30} - 576s_{15}s_{29} - 864s_{16}s_{28} - 864s_{17}s_{27} - 864s_{18}s_{26} - 1152s_{19}s_{25} - 576s_{0}s_{45} - 1152s_{1}s_{44} - 288s_{2}s_{43} -$
$288s_{3}s_{42} - 576s_{4}s_{41} - 288s_{5}s_{40} - 5272704s_{7}s_{38} - 5272704s_{8}s_{37} - 5272704s_{9}s_{36} - 576s_{10}s_{35} - 1152s_{11}s_{34} -$
$288s_{12}s_{33} - 288s_{13}s_{32} - 576s_{14}s_{31} - 288s_{15}s_{30} - 864s_{16}s_{29} - 1152s_{17}s_{28} - 1152s_{18}s_{27} - 1152s_{19}s_{26} - 576s_{0}s_{46} -$
$864s_{1}s_{45} - 1152s_{2}s_{44} - 576s_{3}s_{43} - 576s_{4}s_{42} - 288s_{5}s_{41} - 576s_{6}s_{40} - 1728s_{9}s_{37} - 576s_{10}s_{36} - 864s_{11}s_{35} -$
$1152s_{12}s_{34} - 576s_{13}s_{33} - 576s_{14}s_{32} - 288s_{15}s_{31} - 576s_{16}s_{30} - 1152s_{17}s_{29} - 864s_{18}s_{28} - 864s_{19}s_{27} - 576s_{0}s_{47} -$
$1152s_{1}s_{46} - 864s_{2}s_{45} - 864s_{3}s_{44} - 576s_{4}s_{43} - 288s_{5}s_{42} - 288s_{6}s_{41} - 576s_{7}s_{40} - 5272704s_{8}s_{39} - 5272704s_{9}s_{38} -$
$576s_{10}s_{37} - 1152s_{11}s_{36} - 864s_{12}s_{35} - 864s_{13}s_{34} - 576s_{14}s_{33} - 288s_{15}s_{32} - 288s_{16}s_{31} - 576s_{17}s_{30} - 1152s_{18}s_{29} -$
$1152s_{19}s_{28} + 1728s_{20}s_{27} - 576s_{0}s_{48} - 1152s_{1}s_{47} - 864s_{2}s_{46} - 1152s_{3}s_{45} - 864s_{4}s_{44} - 288s_{5}s_{43} - 576s_{6}s_{42} -$
$576s_{7}s_{41} - 576s_{8}s_{40} - 5272704s_{9}s_{39} - 576s_{10}s_{38} - 1152s_{11}s_{37} - 864s_{12}s_{36} - 1152s_{13}s_{35} - 864s_{14}s_{34} - 288s_{15}s_{33} -$
$576s_{16}s_{32} - 576s_{17}s_{31} - 576s_{18}s_{30} - 864s_{19}s_{29} - 576s_{0}s_{49} - 864s_{1}s_{48} - 864s_{2}s_{47} - 864s_{3}s_{46} - 1152s_{4}s_{45} -$
$576s_{5}s_{44} - 576s_{6}s_{43} - 576s_{7}s_{42} - 576s_{8}s_{41} - 288s_{9}s_{40} - 576s_{10}s_{39} - 864s_{11}s_{38} - 864s_{12}s_{37} - 864s_{13}s_{36} -$
$1152s_{14}s_{35} - 576s_{15}s_{34} - 576s_{16}s_{33} - 576s_{17}s_{32} - 576s_{18}s_{31} - 288s_{19}s_{30} + 1728s_{20}s_{29} - 288s_{0}s_{50} - 864s_{1}s_{49} -$
$1152s_{2}s_{48} - 1152s_{3}s_{47} - 1152s_{4}s_{46} - 576s_{5}s_{45} - 1152s_{6}s_{44} - 288s_{7}s_{43} - 288s_{8}s_{42} - 576s_{9}s_{41} - 5272704s_{10}s_{40} -$
$864s_{11}s_{39} - 1152s_{12}s_{38} - 1152s_{13}s_{37} - 1152s_{14}s_{36} - 576s_{15}s_{35} - 1152s_{16}s_{34} - 288s_{17}s_{33} - 288s_{18}s_{32} - 576s_{19}s_{31} +$
$576s_{20}s_{30} + 1728s_{21}s_{29} - 288s_{0}s_{51} - 576s_{1}s_{50} - 1152s_{2}s_{49} - 864s_{3}s_{48} - 864s_{4}s_{47} - 576s_{5}s_{46} - 864s_{6}s_{45} -$
$1152s_{7}s_{44} - 576s_{8}s_{43} - 576s_{9}s_{42} - 5272704s_{10}s_{41} - 5272704s_{11}s_{40} - 1152s_{12}s_{39} - 864s_{13}s_{38} - 864s_{14}s_{37} -$
$576s_{15}s_{36} - 864s_{16}s_{35} - 1152s_{17}s_{34} - 576s_{18}s_{33} - 576s_{19}s_{32} - 288s_{0}s_{52} - 288s_{1}s_{51} - 576s_{2}s_{50} - 1152s_{3}s_{49} -$
$1152s_{4}s_{48} - 576s_{5}s_{47} - 1152s_{6}s_{46} - 864s_{7}s_{45} - 864s_{8}s_{44} - 576s_{9}s_{43} - 5272704s_{10}s_{42} - 864s_{12}s_{40} - 1152s_{13}s_{39} -$
$1152s_{14}s_{38} - 576s_{15}s_{37} - 1152s_{16}s_{36} - 864s_{17}s_{35} - 864s_{18}s_{34} - 576s_{19}s_{33} + 576s_{21}s_{31} - 288s_{0}s_{53} - 576s_{1}s_{52} -$
$576s_{2}s_{51} - 576s_{3}s_{50} - 864s_{4}s_{49} - 576s_{5}s_{48} - 1152s_{6}s_{47} - 864s_{7}s_{46} - 1152s_{8}s_{45} - 864s_{9}s_{44} - 5272704s_{10}s_{43} -$
$5272704s_{12}s_{41} - 1728s_{13}s_{40} - 864s_{14}s_{39} - 576s_{15}s_{38} - 1152s_{16}s_{37} - 864s_{17}s_{36} - 1152s_{18}s_{35} - 864s_{19}s_{34} +$
$1728s_{24}s_{29} - 576s_{0}s_{54} - 576s_{1}s_{53} - 576s_{2}s_{52} - 576s_{3}s_{51} - 288s_{4}s_{50} - 576s_{5}s_{49} - 864s_{6}s_{48} - 864s_{7}s_{47} - 864s_{8}s_{46} -$
$1152s_{9}s_{45} - 1728s_{13}s_{41} - 864s_{14}s_{40} - 576s_{15}s_{39} - 864s_{16}s_{38} - 864s_{17}s_{37} - 864s_{18}s_{36} - 1152s_{19}s_{35} + 576s_{20}s_{34} +$
$576s_{22}s_{32} + 576s_{24}s_{30} - 576s_{0}s_{55} - 1152s_{1}s_{54} - 288s_{2}s_{53} - 288s_{3}s_{52} - 576s_{4}s_{51} - 288s_{5}s_{50} - 864s_{6}s_{49} -$
$1152s_{7}s_{48} - 1152s_{8}s_{47} - 1152s_{9}s_{46} - 5272704s_{11}s_{44} - 5272704s_{13}s_{42} - 864s_{14}s_{41} - 288s_{15}s_{40} - 864s_{16}s_{39} -$
$1152s_{17}s_{38} - 1152s_{18}s_{37} - 1152s_{19}s_{36} - 576s_{0}s_{56} - 864s_{1}s_{55} - 1152s_{2}s_{54} - 576s_{3}s_{53} - 576s_{4}s_{52} - 288s_{5}s_{51} -$
$576s_{6}s_{50} - 1152s_{7}s_{49} - 864s_{8}s_{48} - 864s_{9}s_{47} - 5272704s_{11}s_{45} - 5272704s_{12}s_{44} - 1728s_{14}s_{42} - 288s_{15}s_{41} -$
$576s_{16}s_{40} - 1152s_{17}s_{39} - 864s_{18}s_{38} - 864s_{19}s_{37} + 576s_{21}s_{35} + 576s_{22}s_{34} + 576s_{23}s_{33} + 576s_{24}s_{32} + 576s_{25}s_{31} -$
$576s_{0}s_{57} - 1152s_{1}s_{56} - 864s_{2}s_{55} - 864s_{3}s_{54} - 576s_{4}s_{53} - 288s_{5}s_{52} - 288s_{6}s_{51} - 576s_{7}s_{50} - 1152s_{8}s_{49} -$
$1152s_{9}s_{48} - 5272704s_{11}s_{46} - 1728s_{13}s_{44} - 5272704s_{14}s_{43} - 288s_{15}s_{42} - 288s_{16}s_{41} - 576s_{17}s_{40} - 1152s_{18}s_{39} -$
$1152s_{19}s_{38} + 576s_{20}s_{37} + 576s_{27}s_{30} - 576s_{0}s_{58} - 1152s_{1}s_{57} - 864s_{2}s_{56} - 1152s_{3}s_{55} - 864s_{4}s_{54} - 288s_{5}s_{53} -$
$576s_{6}s_{52} - 576s_{7}s_{51} - 576s_{8}s_{50} - 864s_{9}s_{49} - 5272704s_{13}s_{45} - 864s_{14}s_{44} - 288s_{15}s_{43} - 576s_{16}s_{42} - 576s_{17}s_{41} -$
$576s_{18}s_{40} - 864s_{19}s_{39} + 576s_{22}s_{36} + 576s_{23}s_{35} + 1152s_{24}s_{34} + 576s_{25}s_{33} + 576s_{26}s_{32} - 576s_{0}s_{59} - 864s_{1}s_{58} -$
$864s_{2}s_{57} - 864s_{3}s_{56} - 1152s_{4}s_{55} - 576s_{5}s_{54} - 576s_{6}s_{53} - 576s_{7}s_{52} - 576s_{8}s_{51} - 288s_{9}s_{50} - 5272704s_{12}s_{47} -$
$1728s_{14}s_{45} - 576s_{15}s_{44} - 576s_{16}s_{43} - 576s_{17}s_{42} - 576s_{18}s_{41} - 288s_{19}s_{40} + 576s_{20}s_{39} + 576s_{21}s_{38} + 576s_{23}s_{36} +$
$576s_{26}s_{33} + 576s_{28}s_{31} + 576s_{29}s_{30} - 864s_{1}s_{59} - 1152s_{2}s_{58} - 1152s_{3}s_{57} - 1152s_{4}s_{56} - 576s_{5}s_{55} - 1152s_{6}s_{54} -$
$288s_{7}s_{53} - 288s_{8}s_{52} - 576s_{9}s_{51} - 288s_{10}s_{50} - 5272704s_{12}s_{48} - 5272704s_{13}s_{47} - 5272704s_{14}s_{46} - 576s_{15}s_{45} -$
$1152s_{16}s_{44} - 288s_{17}s_{43} - 288s_{18}s_{42} - 576s_{19}s_{41} + 576s_{20}s_{40} + 576s_{21}s_{39} + 576s_{22}s_{38} + 576s_{23}s_{37} + 576s_{24}s_{36} +$
$1152s_{25}s_{35} + 576s_{26}s_{34} + 576s_{27}s_{33} + 576s_{28}s_{32} + 576s_{29}s_{31} - 1152s_{2}s_{59} - 864s_{3}s_{58} - 864s_{4}s_{57} - 576s_{5}s_{56} -$
$864s_{6}s_{55} - 1152s_{7}s_{54} - 576s_{8}s_{53} - 576s_{9}s_{52} - 288s_{10}s_{51} - 576s_{11}s_{50} - 1728s_{14}s_{47} - 576s_{15}s_{46} - 864s_{16}s_{45} -$

$1152s_{17}s_{44} - 576s_{18}s_{43} - 576s_{19}s_{42} + 576s_{24}s_{37} + 576s_{25}s_{36} + 576s_{26}s_{35} + 576s_{27}s_{34} - 1152s_{3}s_{59} - 1152s_{4}s_{58} -$
$576s_{5}s_{57} - 1152s_{6}s_{56} - 864s_{7}s_{55} - 864s_{8}s_{54} - 576s_{9}s_{53} - 288s_{10}s_{52} - 288s_{11}s_{51} - 576s_{12}s_{50} - 5272704s_{13}s_{49} -$
$5272704s_{14}s_{48} - 576s_{15}s_{47} - 1152s_{16}s_{46} - 864s_{17}s_{45} - 864s_{18}s_{44} - 576s_{19}s_{43} + 576s_{21}s_{41} + 576s_{24}s_{38} +$
$576s_{25}s_{37} + 1152s_{26}s_{36} + 576s_{27}s_{35} + 576s_{28}s_{34} - 864s_{4}s_{59} - 576s_{5}s_{58} - 1152s_{6}s_{57} - 864s_{7}s_{56} - 1152s_{8}s_{55} -$
$864s_{9}s_{54} - 288s_{10}s_{53} - 576s_{11}s_{52} - 576s_{12}s_{51} - 576s_{13}s_{50} - 5272704s_{14}s_{49} - 576s_{15}s_{48} - 1152s_{16}s_{47} -$
$864s_{17}s_{46} - 1152s_{18}s_{45} - 864s_{19}s_{44} + 576s_{24}s_{39} + 576s_{25}s_{38} + 576s_{26}s_{37} + 576s_{27}s_{36} + 576s_{28}s_{35} + 576s_{29}s_{34} -$
$576s_{5}s_{59} - 864s_{6}s_{58} - 864s_{7}s_{57} - 864s_{8}s_{56} - 1152s_{9}s_{55} - 576s_{10}s_{54} - 576s_{11}s_{53} - 576s_{12}s_{52} - 576s_{13}s_{51} -$
$288s_{14}s_{50} - 576s_{15}s_{49} - 864s_{16}s_{48} - 864s_{17}s_{47} - 864s_{18}s_{46} - 1152s_{19}s_{45} + 576s_{20}s_{44} + 576s_{22}s_{42} + 576s_{24}s_{40} +$
$576s_{25}s_{39} + 576s_{26}s_{38} + 1152s_{27}s_{37} + 576s_{28}s_{36} + 576s_{29}s_{35} - 864s_{6}s_{59} - 1152s_{7}s_{58} - 1152s_{8}s_{57} - 1152s_{9}s_{56} -$
$576s_{10}s_{55} - 1152s_{11}s_{54} - 288s_{12}s_{53} - 288s_{13}s_{52} - 576s_{14}s_{51} - 5272704s_{15}s_{50} - 864s_{16}s_{49} - 1152s_{17}s_{48} -$
$1152s_{18}s_{47} - 1152s_{19}s_{46} - 1152s_{7}s_{59} - 864s_{8}s_{58} - 864s_{9}s_{57} - 576s_{10}s_{56} - 864s_{11}s_{55} - 1152s_{12}s_{54} - 576s_{13}s_{53} -$
$576s_{14}s_{52} - 5272704s_{15}s_{51} - 5272704s_{16}s_{50} - 1152s_{17}s_{49} - 864s_{18}s_{48} - 864s_{19}s_{47} + 576s_{21}s_{45} + 576s_{22}s_{44} +$
$576s_{23}s_{43} + 576s_{24}s_{42} + 576s_{25}s_{41} + 576s_{27}s_{39} + 1152s_{28}s_{38} + 576s_{29}s_{37} - 1152s_{8}s_{59} - 1152s_{9}s_{58} - 576s_{10}s_{57} -$
$1152s_{11}s_{56} - 864s_{12}s_{55} - 864s_{13}s_{54} - 576s_{14}s_{53} - 5272704s_{15}s_{52} - 864s_{17}s_{50} - 1152s_{18}s_{49} - 1152s_{19}s_{48} +$
$576s_{20}s_{47} + 576s_{27}s_{40} + 576s_{28}s_{39} + 576s_{29}s_{38} + 1728s_{30}s_{37} - 864s_{9}s_{59} - 576s_{10}s_{58} - 1152s_{11}s_{57} - 864s_{12}s_{56} -$
$1152s_{13}s_{55} - 864s_{14}s_{54} - 5272704s_{15}s_{53} - 5272704s_{17}s_{51} - 1728s_{18}s_{50} - 864s_{19}s_{49} + 576s_{22}s_{46} + 576s_{23}s_{45} +$
$1152s_{24}s_{44} + 576s_{25}s_{43} + 576s_{26}s_{42} + 1152s_{29}s_{39} - 576s_{10}s_{59} - 864s_{11}s_{58} - 864s_{12}s_{57} - 864s_{13}s_{56} - 1152s_{14}s_{55} -$
$1728s_{18}s_{51} - 864s_{19}s_{50} + 576s_{20}s_{49} + 576s_{21}s_{48} + 576s_{23}s_{46} + 576s_{26}s_{43} + 576s_{28}s_{41} + 576s_{29}s_{40} + 1728s_{30}s_{39} -$
$864s_{11}s_{59} - 1152s_{12}s_{58} - 1152s_{13}s_{57} - 1152s_{14}s_{56} - 5272704s_{16}s_{54} - 5272704s_{18}s_{52} - 864s_{19}s_{51} + 576s_{20}s_{50} +$
$576s_{21}s_{49} + 576s_{22}s_{48} + 576s_{23}s_{47} + 576s_{24}s_{46} + 1152s_{25}s_{45} + 576s_{26}s_{44} + 576s_{27}s_{43} + 576s_{28}s_{42} + 576s_{29}s_{41} +$
$576s_{30}s_{40} + 1728s_{31}s_{39} - 1152s_{12}s_{59} - 864s_{13}s_{58} - 864s_{14}s_{57} - 5272704s_{16}s_{55} - 5272704s_{17}s_{54} - 1728s_{19}s_{52} +$
$576s_{24}s_{47} + 576s_{25}s_{46} + 576s_{26}s_{45} + 576s_{27}s_{44} - 1152s_{13}s_{59} - 1152s_{14}s_{58} - 5272704s_{16}s_{56} - 1728s_{18}s_{54} -$
$5272704s_{19}s_{53} + 576s_{21}s_{51} + 576s_{24}s_{48} + 576s_{25}s_{47} + 1152s_{26}s_{46} + 576s_{27}s_{45} + 576s_{28}s_{44} + 576s_{31}s_{41} - 864s_{14}s_{59} -$
$5272704s_{18}s_{55} - 864s_{19}s_{54} + 576s_{24}s_{49} + 576s_{25}s_{48} + 576s_{26}s_{47} + 576s_{27}s_{46} + 576s_{28}s_{45} + 576s_{29}s_{44} + 1728s_{34}s_{39} -$
$5272704s_{17}s_{57} - 1728s_{19}s_{55} + 576s_{20}s_{54} + 576s_{22}s_{52} + 576s_{24}s_{50} + 576s_{25}s_{49} + 576s_{26}s_{48} + 1152s_{27}s_{47} +$
$576s_{28}s_{46} + 576s_{29}s_{45} + 576s_{30}s_{44} + 576s_{32}s_{42} + 576s_{34}s_{40} - 5272704s_{17}s_{58} - 5272704s_{18}s_{57} - 5272704s_{19}s_{56} -$
$1728s_{19}s_{57} + 576s_{21}s_{55} + 576s_{22}s_{54} + 576s_{23}s_{53} + 576s_{24}s_{52} + 576s_{25}s_{51} + 576s_{27}s_{49} + 1152s_{28}s_{48} + 576s_{29}s_{47} +$
$576s_{31}s_{45} + 576s_{32}s_{44} + 576s_{33}s_{43} + 576s_{34}s_{42} + 576s_{35}s_{41} - 5272704s_{18}s_{59} - 5272704s_{19}s_{58} + 576s_{20}s_{57} +$
$576s_{27}s_{50} + 576s_{28}s_{49} + 576s_{29}s_{48} + 576s_{30}s_{47} + 576s_{37}s_{40} - 5272704s_{19}s_{59} + 576s_{22}s_{56} + 576s_{23}s_{55} + 1152s_{24}s_{54} +$
$576s_{25}s_{53} + 576s_{26}s_{52} + 1152s_{29}s_{49} + 576s_{32}s_{46} + 576s_{33}s_{45} + 1152s_{34}s_{44} + 576s_{35}s_{43} + 576s_{36}s_{42} + 576s_{20}s_{59} +$
$576s_{21}s_{58} + 576s_{23}s_{56} + 576s_{26}s_{53} + 576s_{28}s_{51} + 576s_{29}s_{50} + 576s_{30}s_{49} + 576s_{31}s_{48} + 576s_{33}s_{46} + 576s_{36}s_{43} +$
$576s_{38}s_{41} + 576s_{39}s_{40} + 576s_{21}s_{59} + 576s_{22}s_{58} + 576s_{23}s_{57} + 576s_{24}s_{56} + 1152s_{25}s_{55} + 576s_{26}s_{54} + 576s_{27}s_{53} +$
$576s_{28}s_{52} + 576s_{29}s_{51} + 576s_{30}s_{50} + 576s_{31}s_{49} + 576s_{32}s_{48} + 576s_{33}s_{47} + 576s_{34}s_{46} + 1152s_{35}s_{45} + 576s_{36}s_{44} +$
$576s_{37}s_{43} + 576s_{38}s_{42} + 576s_{39}s_{41} + 576s_{24}s_{57} + 576s_{25}s_{56} + 576s_{26}s_{55} + 576s_{27}s_{54} + 576s_{34}s_{47} + 576s_{35}s_{46} +$
$576s_{36}s_{45} + 576s_{37}s_{44} + 576s_{24}s_{58} + 576s_{25}s_{57} + 1152s_{26}s_{56} + 576s_{27}s_{55} + 576s_{28}s_{54} + 576s_{31}s_{51} + 576s_{34}s_{48} +$
$576s_{35}s_{47} + 1152s_{36}s_{46} + 576s_{37}s_{45} + 576s_{38}s_{44} + 576s_{24}s_{59} + 576s_{25}s_{58} + 576s_{26}s_{57} + 576s_{27}s_{56} + 576s_{28}s_{55} +$
$576s_{29}s_{54} + 576s_{34}s_{49} + 576s_{35}s_{48} + 576s_{36}s_{47} + 576s_{37}s_{46} + 576s_{38}s_{45} + 576s_{39}s_{44} + 576s_{25}s_{59} + 576s_{26}s_{58} +$
$1152s_{27}s_{57} + 576s_{28}s_{56} + 576s_{29}s_{55} + 576s_{30}s_{54} + 576s_{32}s_{52} + 576s_{34}s_{50} + 576s_{35}s_{49} + 576s_{36}s_{48} + 1152s_{37}s_{47} +$
$576s_{38}s_{46} + 576s_{39}s_{45} + 576s_{27}s_{59} + 1152s_{28}s_{58} + 576s_{29}s_{57} + 576s_{31}s_{55} + 576s_{32}s_{54} + 576s_{33}s_{53} + 576s_{34}s_{52} +$
$576s_{35}s_{51} + 576s_{37}s_{49} + 1152s_{38}s_{48} + 576s_{39}s_{47} + 576s_{28}s_{59} + 576s_{29}s_{58} + 576s_{30}s_{57} + 576s_{37}s_{50} + 576s_{38}s_{49} +$
$576s_{39}s_{48} + 1728s_{40}s_{47} + 1152s_{29}s_{59} + 576s_{32}s_{56} + 576s_{33}s_{55} + 1152s_{34}s_{54} + 576s_{35}s_{53} + 576s_{36}s_{52} + 1152s_{39}s_{49} +$
$576s_{30}s_{59} + 576s_{31}s_{58} + 576s_{33}s_{56} + 576s_{36}s_{53} + 576s_{38}s_{51} + 576s_{39}s_{50} + 1728s_{40}s_{49} + 576s_{31}s_{59} + 576s_{32}s_{58} +$
$576s_{33}s_{57} + 576s_{34}s_{56} + 1152s_{35}s_{55} + 576s_{36}s_{54} + 576s_{37}s_{53} + 576s_{38}s_{52} + 576s_{39}s_{51} + 576s_{40}s_{50} + 1728s_{41}s_{49} +$
$576s_{34}s_{57} + 576s_{35}s_{56} + 576s_{36}s_{55} + 576s_{37}s_{54} + 576s_{34}s_{58} + 576s_{35}s_{57} + 1152s_{36}s_{56} + 576s_{37}s_{55} + 576s_{38}s_{54} +$
$576s_{41}s_{51} + 576s_{34}s_{59} + 576s_{35}s_{58} + 576s_{36}s_{57} + 576s_{37}s_{56} + 576s_{38}s_{55} + 576s_{39}s_{54} + 1728s_{44}s_{49} + 576s_{35}s_{59} +$
$576s_{36}s_{58} + 1152s_{37}s_{57} + 576s_{38}s_{56} + 576s_{39}s_{55} + 576s_{40}s_{54} + 576s_{42}s_{52} + 576s_{44}s_{50} + 576s_{37}s_{59} + 1152s_{38}s_{58} +$
$576s_{39}s_{57} + 576s_{41}s_{55} + 576s_{42}s_{54} + 576s_{43}s_{53} + 576s_{44}s_{52} + 576s_{45}s_{51} + 576s_{38}s_{59} + 576s_{39}s_{58} + 576s_{40}s_{57} +$
$576s_{47}s_{50} + 1152s_{39}s_{59} + 576s_{42}s_{56} + 576s_{43}s_{55} + 1152s_{44}s_{54} + 576s_{45}s_{53} + 576s_{46}s_{52} + 576s_{40}s_{59} + 576s_{41}s_{58} +$

$576s_{43}s_{56} + 576s_{46}s_{53} + 576s_{48}s_{51} + 576s_{49}s_{50} + 576s_{41}s_{59} + 576s_{42}s_{58} + 576s_{43}s_{57} + 576s_{44}s_{56} + 1152s_{45}s_{55} +$
$576s_{46}s_{54} + 576s_{47}s_{53} + 576s_{48}s_{52} + 576s_{49}s_{51} + 576s_{44}s_{57} + 576s_{45}s_{56} + 576s_{46}s_{55} + 576s_{47}s_{54} + 576s_{44}s_{58} +$
$576s_{45}s_{57} + 1152s_{46}s_{56} + 576s_{47}s_{55} + 576s_{48}s_{54} + 576s_{44}s_{59} + 576s_{45}s_{58} + 576s_{46}s_{57} + 576s_{47}s_{56} + 576s_{48}s_{55} +$
$576s_{49}s_{54} + 576s_{45}s_{59} + 576s_{46}s_{58} + 1152s_{47}s_{57} + 576s_{48}s_{56} + 576s_{49}s_{55} + 576s_{47}s_{59} + 1152s_{48}s_{58} + 576s_{49}s_{57} +$
$576s_{48}s_{59} + 576s_{49}s_{58} + 1728s_{50}s_{57} + 1152s_{49}s_{59} + 1728s_{50}s_{59} + 1728s_{51}s_{59} + 1728s_{54}s_{59} + 316483200$

A complete expression of $\hat{H}_2\left(\hat{\sigma}^z\right)$ for 36-order Williamson based method or 108-order Baumert-Hall based method

$\hat{H}_2\left(\hat{\sigma}^z\right) = 10555200\hat{\sigma}^z_{0} + 10561680\hat{\sigma}^z_{1} + 10562544\hat{\sigma}^z_{2} + 10566864\hat{\sigma}^z_{3} + 10569456\hat{\sigma}^z_{4} + 10555200\hat{\sigma}^z_{5} +$
$10561680\hat{\sigma}^z_{6} + 10562544\hat{\sigma}^z_{7} + 10566864\hat{\sigma}^z_{8} + 10569456\hat{\sigma}^z_{9} + 10555200\hat{\sigma}^z_{10} + 10561680\hat{\sigma}^z_{11} + 10562544\hat{\sigma}^z_{12} +$
$10566864\hat{\sigma}^z_{13} + 10569456\hat{\sigma}^z_{14} + 10555200\hat{\sigma}^z_{15} + 10561680\hat{\sigma}^z_{16} + 10562544\hat{\sigma}^z_{17} + 10566864\hat{\sigma}^z_{18} + 10569456\hat{\sigma}^z_{19} -$
$5278752\hat{\sigma}^z_{20} - 5277888\hat{\sigma}^z_{21} - 5277600\hat{\sigma}^z_{22} - 5278752\hat{\sigma}^z_{23} - 5282208\hat{\sigma}^z_{24} - 5281920\hat{\sigma}^z_{25} - 5283072\hat{\sigma}^z_{26} -$
$5283648\hat{\sigma}^z_{27} - 5283072\hat{\sigma}^z_{28} - 5288832\hat{\sigma}^z_{29} - 5278752\hat{\sigma}^z_{30} - 5277888\hat{\sigma}^z_{31} - 5277600\hat{\sigma}^z_{32} - 5278752\hat{\sigma}^z_{33} -$
$5282208\hat{\sigma}^z_{34} - 5281920\hat{\sigma}^z_{35} - 5283072\hat{\sigma}^z_{36} - 5283648\hat{\sigma}^z_{37} - 5283072\hat{\sigma}^z_{38} - 5288832\hat{\sigma}^z_{39} - 5278752\hat{\sigma}^z_{40} -$
$5277888\hat{\sigma}^z_{41} - 5277600\hat{\sigma}^z_{42} - 5278752\hat{\sigma}^z_{43} - 5282208\hat{\sigma}^z_{44} - 5281920\hat{\sigma}^z_{45} - 5283072\hat{\sigma}^z_{46} - 5283648\hat{\sigma}^z_{47} -$
$5283072\hat{\sigma}^z_{48} - 5288832\hat{\sigma}^z_{49} - 5278752\hat{\sigma}^z_{50} - 5277888\hat{\sigma}^z_{51} - 5277600\hat{\sigma}^z_{52} - 5278752\hat{\sigma}^z_{53} - 5282208\hat{\sigma}^z_{54} -$
$5281920\hat{\sigma}^z_{55} - 5283072\hat{\sigma}^z_{56} - 5283648\hat{\sigma}^z_{57} - 5283072\hat{\sigma}^z_{58} - 5288832\hat{\sigma}^z_{59} + 2636352\hat{\sigma}^z_{0}\hat{\sigma}^z_{1} + 2636352\hat{\sigma}^z_{0}\hat{\sigma}^z_{2} +$
$2636352\hat{\sigma}^z_{0}\hat{\sigma}^z_{3} + 2636352\hat{\sigma}^z_{1}\hat{\sigma}^z_{2} + 2636352\hat{\sigma}^z_{0}\hat{\sigma}^z_{4} + 2636352\hat{\sigma}^z_{1}\hat{\sigma}^z_{3} + 576\hat{\sigma}^z_{0}\hat{\sigma}^z_{5} + 2636352\hat{\sigma}^z_{1}\hat{\sigma}^z_{4} + 2636352\hat{\sigma}^z_{2}\hat{\sigma}^z_{3} +$
$1008\hat{\sigma}^z_{0}\hat{\sigma}^z_{6} + 1008\hat{\sigma}^z_{1}\hat{\sigma}^z_{5} + 2636352\hat{\sigma}^z_{2}\hat{\sigma}^z_{4} + 1008\hat{\sigma}^z_{0}\hat{\sigma}^z_{7} + 1872\hat{\sigma}^z_{1}\hat{\sigma}^z_{6} + 1008\hat{\sigma}^z_{2}\hat{\sigma}^z_{5} + 2636352\hat{\sigma}^z_{3}\hat{\sigma}^z_{4} + 1008\hat{\sigma}^z_{0}\hat{\sigma}^z_{8} +$
$1728\hat{\sigma}^z_{1}\hat{\sigma}^z_{7} + 1728\hat{\sigma}^z_{2}\hat{\sigma}^z_{6} + 1008\hat{\sigma}^z_{3}\hat{\sigma}^z_{5} + 1008\hat{\sigma}^z_{0}\hat{\sigma}^z_{9} + 1728\hat{\sigma}^z_{1}\hat{\sigma}^z_{8} + 1872\hat{\sigma}^z_{2}\hat{\sigma}^z_{7} + 1728\hat{\sigma}^z_{3}\hat{\sigma}^z_{6} + 1008\hat{\sigma}^z_{4}\hat{\sigma}^z_{5} + 576\hat{\sigma}^z_{0}\hat{\sigma}^z_{10} +$
$1728\hat{\sigma}^z_{1}\hat{\sigma}^z_{9} + 1728\hat{\sigma}^z_{2}\hat{\sigma}^z_{8} + 1728\hat{\sigma}^z_{3}\hat{\sigma}^z_{7} + 1728\hat{\sigma}^z_{4}\hat{\sigma}^z_{6} + 1008\hat{\sigma}^z_{0}\hat{\sigma}^z_{11} + 1008\hat{\sigma}^z_{1}\hat{\sigma}^z_{10} + 1728\hat{\sigma}^z_{2}\hat{\sigma}^z_{9} + 1872\hat{\sigma}^z_{3}\hat{\sigma}^z_{8} +$
$1728\hat{\sigma}^z_{4}\hat{\sigma}^z_{7} + 2636352\hat{\sigma}^z_{5}\hat{\sigma}^z_{6} + 1008\hat{\sigma}^z_{0}\hat{\sigma}^z_{12} + 1872\hat{\sigma}^z_{1}\hat{\sigma}^z_{11} + 1008\hat{\sigma}^z_{2}\hat{\sigma}^z_{10} + 1728\hat{\sigma}^z_{3}\hat{\sigma}^z_{9} + 1728\hat{\sigma}^z_{4}\hat{\sigma}^z_{8} + 2636352\hat{\sigma}^z_{5}\hat{\sigma}^z_{7} +$
$1008\hat{\sigma}^z_{0}\hat{\sigma}^z_{13} + 1728\hat{\sigma}^z_{1}\hat{\sigma}^z_{12} + 1728\hat{\sigma}^z_{2}\hat{\sigma}^z_{11} + 1008\hat{\sigma}^z_{3}\hat{\sigma}^z_{10} + 1872\hat{\sigma}^z_{4}\hat{\sigma}^z_{9} + 2636352\hat{\sigma}^z_{5}\hat{\sigma}^z_{8} + 2636352\hat{\sigma}^z_{6}\hat{\sigma}^z_{7} + 1008\hat{\sigma}^z_{0}\hat{\sigma}^z_{14} +$
$1728\hat{\sigma}^z_{1}\hat{\sigma}^z_{13} + 1872\hat{\sigma}^z_{2}\hat{\sigma}^z_{12} + 1728\hat{\sigma}^z_{3}\hat{\sigma}^z_{11} + 1008\hat{\sigma}^z_{4}\hat{\sigma}^z_{10} + 2636352\hat{\sigma}^z_{5}\hat{\sigma}^z_{9} + 2636352\hat{\sigma}^z_{6}\hat{\sigma}^z_{8} + 576\hat{\sigma}^z_{0}\hat{\sigma}^z_{15} + 1728\hat{\sigma}^z_{1}\hat{\sigma}^z_{14} +$
$1728\hat{\sigma}^z_{2}\hat{\sigma}^z_{13} + 1728\hat{\sigma}^z_{3}\hat{\sigma}^z_{12} + 1728\hat{\sigma}^z_{4}\hat{\sigma}^z_{11} + 576\hat{\sigma}^z_{5}\hat{\sigma}^z_{10} + 2636352\hat{\sigma}^z_{6}\hat{\sigma}^z_{9} + 2636352\hat{\sigma}^z_{7}\hat{\sigma}^z_{8} + 1008\hat{\sigma}^z_{0}\hat{\sigma}^z_{16} + 1008\hat{\sigma}^z_{1}\hat{\sigma}^z_{15} +$
$1728\hat{\sigma}^z_{2}\hat{\sigma}^z_{14} + 1872\hat{\sigma}^z_{3}\hat{\sigma}^z_{13} + 1728\hat{\sigma}^z_{4}\hat{\sigma}^z_{12} + 1008\hat{\sigma}^z_{5}\hat{\sigma}^z_{11} + 1008\hat{\sigma}^z_{6}\hat{\sigma}^z_{10} + 2636352\hat{\sigma}^z_{7}\hat{\sigma}^z_{9} + 1008\hat{\sigma}^z_{0}\hat{\sigma}^z_{17} + 1872\hat{\sigma}^z_{1}\hat{\sigma}^z_{16} +$
$1008\hat{\sigma}^z_{2}\hat{\sigma}^z_{15} + 1728\hat{\sigma}^z_{3}\hat{\sigma}^z_{14} + 1728\hat{\sigma}^z_{4}\hat{\sigma}^z_{13} + 1008\hat{\sigma}^z_{5}\hat{\sigma}^z_{12} + 1872\hat{\sigma}^z_{6}\hat{\sigma}^z_{11} + 1008\hat{\sigma}^z_{7}\hat{\sigma}^z_{10} + 2636352\hat{\sigma}^z_{8}\hat{\sigma}^z_{9} + 1008\hat{\sigma}^z_{0}\hat{\sigma}^z_{18} +$
$1728\hat{\sigma}^z_{1}\hat{\sigma}^z_{17} + 1728\hat{\sigma}^z_{2}\hat{\sigma}^z_{16} + 1008\hat{\sigma}^z_{3}\hat{\sigma}^z_{15} + 1872\hat{\sigma}^z_{4}\hat{\sigma}^z_{14} + 1008\hat{\sigma}^z_{5}\hat{\sigma}^z_{13} + 1728\hat{\sigma}^z_{6}\hat{\sigma}^z_{12} + 1728\hat{\sigma}^z_{7}\hat{\sigma}^z_{11} + 1008\hat{\sigma}^z_{8}\hat{\sigma}^z_{10} +$
$1008\hat{\sigma}^z_{0}\hat{\sigma}^z_{19} + 1728\hat{\sigma}^z_{1}\hat{\sigma}^z_{18} + 1872\hat{\sigma}^z_{2}\hat{\sigma}^z_{17} + 1728\hat{\sigma}^z_{3}\hat{\sigma}^z_{16} + 1008\hat{\sigma}^z_{4}\hat{\sigma}^z_{15} + 1008\hat{\sigma}^z_{5}\hat{\sigma}^z_{14} + 1728\hat{\sigma}^z_{6}\hat{\sigma}^z_{13} + 1872\hat{\sigma}^z_{7}\hat{\sigma}^z_{12} +$
$1728\hat{\sigma}^z_{8}\hat{\sigma}^z_{11} + 1008\hat{\sigma}^z_{9}\hat{\sigma}^z_{10} - 5272704\hat{\sigma}^z_{0}\hat{\sigma}^z_{20} + 1728\hat{\sigma}^z_{1}\hat{\sigma}^z_{19} + 1728\hat{\sigma}^z_{2}\hat{\sigma}^z_{18} + 1728\hat{\sigma}^z_{3}\hat{\sigma}^z_{17} + 1728\hat{\sigma}^z_{4}\hat{\sigma}^z_{16} + 576\hat{\sigma}^z_{5}\hat{\sigma}^z_{15} +$
$1728\hat{\sigma}^z_{6}\hat{\sigma}^z_{14} + 1728\hat{\sigma}^z_{7}\hat{\sigma}^z_{13} + 1728\hat{\sigma}^z_{8}\hat{\sigma}^z_{12} + 1728\hat{\sigma}^z_{9}\hat{\sigma}^z_{11} - 5272704\hat{\sigma}^z_{0}\hat{\sigma}^z_{21} - 5272704\hat{\sigma}^z_{1}\hat{\sigma}^z_{20} + 1728\hat{\sigma}^z_{2}\hat{\sigma}^z_{19} +$
$1872\hat{\sigma}^z_{3}\hat{\sigma}^z_{18} + 1728\hat{\sigma}^z_{4}\hat{\sigma}^z_{17} + 1008\hat{\sigma}^z_{5}\hat{\sigma}^z_{16} + 1008\hat{\sigma}^z_{6}\hat{\sigma}^z_{15} + 1728\hat{\sigma}^z_{7}\hat{\sigma}^z_{14} + 1872\hat{\sigma}^z_{8}\hat{\sigma}^z_{13} + 1728\hat{\sigma}^z_{9}\hat{\sigma}^z_{12} + 2636352\hat{\sigma}^z_{10}\hat{\sigma}^z_{11} -$
$5272704\hat{\sigma}^z_{0}\hat{\sigma}^z_{22} - 864\hat{\sigma}^z_{2}\hat{\sigma}^z_{20} + 1728\hat{\sigma}^z_{3}\hat{\sigma}^z_{19} + 1728\hat{\sigma}^z_{4}\hat{\sigma}^z_{18} + 1008\hat{\sigma}^z_{5}\hat{\sigma}^z_{17} + 1872\hat{\sigma}^z_{6}\hat{\sigma}^z_{16} + 1008\hat{\sigma}^z_{7}\hat{\sigma}^z_{15} + 1728\hat{\sigma}^z_{8}\hat{\sigma}^z_{14} +$
$1728\hat{\sigma}^z_{9}\hat{\sigma}^z_{13} + 2636352\hat{\sigma}^z_{10}\hat{\sigma}^z_{12} - 5272704\hat{\sigma}^z_{0}\hat{\sigma}^z_{23} - 5272704\hat{\sigma}^z_{2}\hat{\sigma}^z_{21} - 1728\hat{\sigma}^z_{3}\hat{\sigma}^z_{20} + 1872\hat{\sigma}^z_{4}\hat{\sigma}^z_{19} + 1008\hat{\sigma}^z_{5}\hat{\sigma}^z_{18} +$
$1728\hat{\sigma}^z_{6}\hat{\sigma}^z_{17} + 1728\hat{\sigma}^z_{7}\hat{\sigma}^z_{16} + 1008\hat{\sigma}^z_{8}\hat{\sigma}^z_{15} + 1872\hat{\sigma}^z_{9}\hat{\sigma}^z_{14} + 2636352\hat{\sigma}^z_{10}\hat{\sigma}^z_{13} + 2636352\hat{\sigma}^z_{11}\hat{\sigma}^z_{12} - 1728\hat{\sigma}^z_{3}\hat{\sigma}^z_{21} -$
$864\hat{\sigma}^z_{4}\hat{\sigma}^z_{20} + 1008\hat{\sigma}^z_{5}\hat{\sigma}^z_{19} + 1728\hat{\sigma}^z_{6}\hat{\sigma}^z_{18} + 1872\hat{\sigma}^z_{7}\hat{\sigma}^z_{17} + 1728\hat{\sigma}^z_{8}\hat{\sigma}^z_{16} + 1008\hat{\sigma}^z_{9}\hat{\sigma}^z_{15} + 2636352\hat{\sigma}^z_{10}\hat{\sigma}^z_{14} + 2636352\hat{\sigma}^z_{11}\hat{\sigma}^z_{13} -$
$5272704\hat{\sigma}^z_{1}\hat{\sigma}^z_{24} - 5272704\hat{\sigma}^z_{3}\hat{\sigma}^z_{22} - 864\hat{\sigma}^z_{4}\hat{\sigma}^z_{21} - 288\hat{\sigma}^z_{5}\hat{\sigma}^z_{20} + 1728\hat{\sigma}^z_{6}\hat{\sigma}^z_{19} + 1728\hat{\sigma}^z_{7}\hat{\sigma}^z_{18} + 1728\hat{\sigma}^z_{8}\hat{\sigma}^z_{17} +$
$1728\hat{\sigma}^z_{9}\hat{\sigma}^z_{16} + 576\hat{\sigma}^z_{10}\hat{\sigma}^z_{15} + 2636352\hat{\sigma}^z_{11}\hat{\sigma}^z_{14} + 2636352\hat{\sigma}^z_{12}\hat{\sigma}^z_{13} - 5272704\hat{\sigma}^z_{1}\hat{\sigma}^z_{25} - 5272704\hat{\sigma}^z_{2}\hat{\sigma}^z_{24} - 1728\hat{\sigma}^z_{4}\hat{\sigma}^z_{22} -$
$288\hat{\sigma}^z_{5}\hat{\sigma}^z_{21} - 576\hat{\sigma}^z_{6}\hat{\sigma}^z_{20} + 1728\hat{\sigma}^z_{7}\hat{\sigma}^z_{19} + 1872\hat{\sigma}^z_{8}\hat{\sigma}^z_{18} + 1728\hat{\sigma}^z_{9}\hat{\sigma}^z_{17} + 1008\hat{\sigma}^z_{10}\hat{\sigma}^z_{16} + 1008\hat{\sigma}^z_{11}\hat{\sigma}^z_{15} + 2636352\hat{\sigma}^z_{12}\hat{\sigma}^z_{14} -$
$5272704\hat{\sigma}^z_{1}\hat{\sigma}^z_{26} - 1728\hat{\sigma}^z_{3}\hat{\sigma}^z_{24} - 5272704\hat{\sigma}^z_{4}\hat{\sigma}^z_{23} - 288\hat{\sigma}^z_{5}\hat{\sigma}^z_{22} - 288\hat{\sigma}^z_{6}\hat{\sigma}^z_{21} - 576\hat{\sigma}^z_{7}\hat{\sigma}^z_{20} + 1728\hat{\sigma}^z_{8}\hat{\sigma}^z_{19} + 1728\hat{\sigma}^z_{9}\hat{\sigma}^z_{18} +$
$1008\hat{\sigma}^z_{10}\hat{\sigma}^z_{17} + 1872\hat{\sigma}^z_{11}\hat{\sigma}^z_{16} + 1008\hat{\sigma}^z_{12}\hat{\sigma}^z_{15} + 2636352\hat{\sigma}^z_{13}\hat{\sigma}^z_{14} - 5272704\hat{\sigma}^z_{3}\hat{\sigma}^z_{25} - 864\hat{\sigma}^z_{4}\hat{\sigma}^z_{24} - 288\hat{\sigma}^z_{5}\hat{\sigma}^z_{23} -$
$576\hat{\sigma}^z_{6}\hat{\sigma}^z_{22} - 576\hat{\sigma}^z_{7}\hat{\sigma}^z_{21} - 576\hat{\sigma}^z_{8}\hat{\sigma}^z_{20} + 1872\hat{\sigma}^z_{9}\hat{\sigma}^z_{19} + 1008\hat{\sigma}^z_{10}\hat{\sigma}^z_{18} + 1728\hat{\sigma}^z_{11}\hat{\sigma}^z_{17} + 1728\hat{\sigma}^z_{12}\hat{\sigma}^z_{16} + 1008\hat{\sigma}^z_{13}\hat{\sigma}^z_{15} -$
$5272704\hat{\sigma}^z_{2}\hat{\sigma}^z_{27} - 1728\hat{\sigma}^z_{4}\hat{\sigma}^z_{25} - 576\hat{\sigma}^z_{5}\hat{\sigma}^z_{24} - 576\hat{\sigma}^z_{6}\hat{\sigma}^z_{23} - 576\hat{\sigma}^z_{7}\hat{\sigma}^z_{22} - 576\hat{\sigma}^z_{8}\hat{\sigma}^z_{21} - 288\hat{\sigma}^z_{9}\hat{\sigma}^z_{20} + 1008\hat{\sigma}^z_{10}\hat{\sigma}^z_{19} +$
$1728\hat{\sigma}^z_{11}\hat{\sigma}^z_{18} + 1872\hat{\sigma}^z_{12}\hat{\sigma}^z_{17} + 1728\hat{\sigma}^z_{13}\hat{\sigma}^z_{16} + 1008\hat{\sigma}^z_{14}\hat{\sigma}^z_{15} - 288\hat{\sigma}^z_{0}\hat{\sigma}^z_{30} - 5272704\hat{\sigma}^z_{2}\hat{\sigma}^z_{28} - 5272704\hat{\sigma}^z_{3}\hat{\sigma}^z_{27} -$
$5272704\hat{\sigma}^z_{4}\hat{\sigma}^z_{26} - 576\hat{\sigma}^z_{5}\hat{\sigma}^z_{25} - 1152\hat{\sigma}^z_{6}\hat{\sigma}^z_{24} - 288\hat{\sigma}^z_{7}\hat{\sigma}^z_{23} - 288\hat{\sigma}^z_{8}\hat{\sigma}^z_{22} - 576\hat{\sigma}^z_{9}\hat{\sigma}^z_{21} - 288\hat{\sigma}^z_{10}\hat{\sigma}^z_{20} + 1728\hat{\sigma}^z_{11}\hat{\sigma}^z_{19} +$
$1728\hat{\sigma}^z_{12}\hat{\sigma}^z_{18} + 1728\hat{\sigma}^z_{13}\hat{\sigma}^z_{17} + 1728\hat{\sigma}^z_{14}\hat{\sigma}^z_{16} - 288\hat{\sigma}^z_{0}\hat{\sigma}^z_{31} - 576\hat{\sigma}^z_{1}\hat{\sigma}^z_{30} - 1728\hat{\sigma}^z_{4}\hat{\sigma}^z_{27} - 576\hat{\sigma}^z_{5}\hat{\sigma}^z_{26} - 864\hat{\sigma}^z_{6}\hat{\sigma}^z_{25} -$
$1152\hat{\sigma}^z_{7}\hat{\sigma}^z_{24} - 576\hat{\sigma}^z_{8}\hat{\sigma}^z_{23} - 576\hat{\sigma}^z_{9}\hat{\sigma}^z_{22} - 288\hat{\sigma}^z_{10}\hat{\sigma}^z_{21} - 576\hat{\sigma}^z_{11}\hat{\sigma}^z_{20} + 1728\hat{\sigma}^z_{12}\hat{\sigma}^z_{19} + 1872\hat{\sigma}^z_{13}\hat{\sigma}^z_{18} + 1728\hat{\sigma}^z_{14}\hat{\sigma}^z_{17} +$
$2636352\hat{\sigma}^z_{15}\hat{\sigma}^z_{16} - 288\hat{\sigma}^z_{0}\hat{\sigma}^z_{32} - 288\hat{\sigma}^z_{1}\hat{\sigma}^z_{31} - 576\hat{\sigma}^z_{2}\hat{\sigma}^z_{30} - 5272704\hat{\sigma}^z_{3}\hat{\sigma}^z_{29} - 5272704\hat{\sigma}^z_{4}\hat{\sigma}^z_{28} - 576\hat{\sigma}^z_{5}\hat{\sigma}^z_{27} -$

$$
\begin{aligned}
&1152\hat{\sigma}^z_{6}\hat{\sigma}^z_{26} - 864\hat{\sigma}^z_{7}\hat{\sigma}^z_{25} - 864\hat{\sigma}^z_{8}\hat{\sigma}^z_{24} - 576\hat{\sigma}^z_{9}\hat{\sigma}^z_{23} - 288\hat{\sigma}^z_{10}\hat{\sigma}^z_{22} - 288\hat{\sigma}^z_{11}\hat{\sigma}^z_{21} - 576\hat{\sigma}^z_{12}\hat{\sigma}^z_{20} + 1728\hat{\sigma}^z_{13}\hat{\sigma}^z_{19} + \\
&1728\hat{\sigma}^z_{14}\hat{\sigma}^z_{18} + 2636352\hat{\sigma}^z_{15}\hat{\sigma}^z_{17} - 288\hat{\sigma}^z_{0}\hat{\sigma}^z_{33} - 576\hat{\sigma}^z_{1}\hat{\sigma}^z_{32} - 576\hat{\sigma}^z_{2}\hat{\sigma}^z_{31} - 576\hat{\sigma}^z_{3}\hat{\sigma}^z_{30} - 5272704\hat{\sigma}^z_{4}\hat{\sigma}^z_{29} - 576\hat{\sigma}^z_{5}\hat{\sigma}^z_{28} - \\
&1152\hat{\sigma}^z_{6}\hat{\sigma}^z_{27} - 864\hat{\sigma}^z_{7}\hat{\sigma}^z_{26} - 1152\hat{\sigma}^z_{8}\hat{\sigma}^z_{25} - 864\hat{\sigma}^z_{9}\hat{\sigma}^z_{24} - 288\hat{\sigma}^z_{10}\hat{\sigma}^z_{23} - 576\hat{\sigma}^z_{11}\hat{\sigma}^z_{22} - 576\hat{\sigma}^z_{12}\hat{\sigma}^z_{21} - 576\hat{\sigma}^z_{13}\hat{\sigma}^z_{20} + \\
&1872\hat{\sigma}^z_{14}\hat{\sigma}^z_{19} + 2636352\hat{\sigma}^z_{15}\hat{\sigma}^z_{18} + 2636352\hat{\sigma}^z_{16}\hat{\sigma}^z_{17} - 576\hat{\sigma}^z_{0}\hat{\sigma}^z_{34} - 576\hat{\sigma}^z_{1}\hat{\sigma}^z_{33} - 576\hat{\sigma}^z_{2}\hat{\sigma}^z_{32} - 576\hat{\sigma}^z_{3}\hat{\sigma}^z_{31} - 288\hat{\sigma}^z_{4}\hat{\sigma}^z_{30} - \\
&576\hat{\sigma}^z_{5}\hat{\sigma}^z_{29} - 864\hat{\sigma}^z_{6}\hat{\sigma}^z_{28} - 864\hat{\sigma}^z_{7}\hat{\sigma}^z_{27} - 864\hat{\sigma}^z_{8}\hat{\sigma}^z_{26} - 1152\hat{\sigma}^z_{9}\hat{\sigma}^z_{25} - 576\hat{\sigma}^z_{10}\hat{\sigma}^z_{24} - 576\hat{\sigma}^z_{11}\hat{\sigma}^z_{23} - 576\hat{\sigma}^z_{12}\hat{\sigma}^z_{22} - \\
&576\hat{\sigma}^z_{13}\hat{\sigma}^z_{21} - 288\hat{\sigma}^z_{14}\hat{\sigma}^z_{20} + 2636352\hat{\sigma}^z_{15}\hat{\sigma}^z_{19} + 2636352\hat{\sigma}^z_{16}\hat{\sigma}^z_{18} - 576\hat{\sigma}^z_{0}\hat{\sigma}^z_{35} - 1152\hat{\sigma}^z_{1}\hat{\sigma}^z_{34} - 288\hat{\sigma}^z_{2}\hat{\sigma}^z_{33} - 288\hat{\sigma}^z_{3}\hat{\sigma}^z_{32} - \\
&576\hat{\sigma}^z_{4}\hat{\sigma}^z_{31} - 5272704\hat{\sigma}^z_{5}\hat{\sigma}^z_{30} - 864\hat{\sigma}^z_{6}\hat{\sigma}^z_{29} - 1152\hat{\sigma}^z_{7}\hat{\sigma}^z_{28} - 1152\hat{\sigma}^z_{8}\hat{\sigma}^z_{27} - 1152\hat{\sigma}^z_{9}\hat{\sigma}^z_{26} - 576\hat{\sigma}^z_{10}\hat{\sigma}^z_{25} - 1152\hat{\sigma}^z_{11}\hat{\sigma}^z_{24} - \\
&288\hat{\sigma}^z_{12}\hat{\sigma}^z_{23} - 288\hat{\sigma}^z_{13}\hat{\sigma}^z_{22} - 576\hat{\sigma}^z_{14}\hat{\sigma}^z_{21} - 288\hat{\sigma}^z_{15}\hat{\sigma}^z_{20} + 2636352\hat{\sigma}^z_{16}\hat{\sigma}^z_{19} + 2636352\hat{\sigma}^z_{17}\hat{\sigma}^z_{18} - 576\hat{\sigma}^z_{0}\hat{\sigma}^z_{36} - 864\hat{\sigma}^z_{1}\hat{\sigma}^z_{35} - \\
&1152\hat{\sigma}^z_{2}\hat{\sigma}^z_{34} - 576\hat{\sigma}^z_{3}\hat{\sigma}^z_{33} - 576\hat{\sigma}^z_{4}\hat{\sigma}^z_{32} - 5272704\hat{\sigma}^z_{5}\hat{\sigma}^z_{31} - 5272704\hat{\sigma}^z_{6}\hat{\sigma}^z_{30} - 1152\hat{\sigma}^z_{7}\hat{\sigma}^z_{29} - 864\hat{\sigma}^z_{8}\hat{\sigma}^z_{28} - 864\hat{\sigma}^z_{9}\hat{\sigma}^z_{27} - \\
&576\hat{\sigma}^z_{10}\hat{\sigma}^z_{26} - 864\hat{\sigma}^z_{11}\hat{\sigma}^z_{25} - 1152\hat{\sigma}^z_{12}\hat{\sigma}^z_{24} - 576\hat{\sigma}^z_{13}\hat{\sigma}^z_{23} - 576\hat{\sigma}^z_{14}\hat{\sigma}^z_{22} - 288\hat{\sigma}^z_{15}\hat{\sigma}^z_{21} - 576\hat{\sigma}^z_{16}\hat{\sigma}^z_{20} + 2636352\hat{\sigma}^z_{17}\hat{\sigma}^z_{19} - \\
&576\hat{\sigma}^z_{0}\hat{\sigma}^z_{37} - 1152\hat{\sigma}^z_{1}\hat{\sigma}^z_{36} - 864\hat{\sigma}^z_{2}\hat{\sigma}^z_{35} - 864\hat{\sigma}^z_{3}\hat{\sigma}^z_{34} - 576\hat{\sigma}^z_{4}\hat{\sigma}^z_{33} - 5272704\hat{\sigma}^z_{5}\hat{\sigma}^z_{32} - 864\hat{\sigma}^z_{7}\hat{\sigma}^z_{30} - 1152\hat{\sigma}^z_{8}\hat{\sigma}^z_{29} - \\
&1152\hat{\sigma}^z_{9}\hat{\sigma}^z_{28} - 576\hat{\sigma}^z_{10}\hat{\sigma}^z_{27} - 1152\hat{\sigma}^z_{11}\hat{\sigma}^z_{26} - 864\hat{\sigma}^z_{12}\hat{\sigma}^z_{25} - 864\hat{\sigma}^z_{13}\hat{\sigma}^z_{24} - 576\hat{\sigma}^z_{14}\hat{\sigma}^z_{23} - 288\hat{\sigma}^z_{15}\hat{\sigma}^z_{22} - 288\hat{\sigma}^z_{16}\hat{\sigma}^z_{21} - \\
&576\hat{\sigma}^z_{17}\hat{\sigma}^z_{20} + 2636352\hat{\sigma}^z_{18}\hat{\sigma}^z_{19} - 576\hat{\sigma}^z_{0}\hat{\sigma}^z_{38} - 1152\hat{\sigma}^z_{1}\hat{\sigma}^z_{37} - 864\hat{\sigma}^z_{2}\hat{\sigma}^z_{36} - 1152\hat{\sigma}^z_{3}\hat{\sigma}^z_{35} - 864\hat{\sigma}^z_{4}\hat{\sigma}^z_{34} - 5272704\hat{\sigma}^z_{5}\hat{\sigma}^z_{33} - \\
&5272704\hat{\sigma}^z_{7}\hat{\sigma}^z_{31} - 1728\hat{\sigma}^z_{8}\hat{\sigma}^z_{30} - 864\hat{\sigma}^z_{9}\hat{\sigma}^z_{29} - 576\hat{\sigma}^z_{10}\hat{\sigma}^z_{28} - 1152\hat{\sigma}^z_{11}\hat{\sigma}^z_{27} - 864\hat{\sigma}^z_{12}\hat{\sigma}^z_{26} - 1152\hat{\sigma}^z_{13}\hat{\sigma}^z_{25} - 864\hat{\sigma}^z_{14}\hat{\sigma}^z_{24} - \\
&288\hat{\sigma}^z_{15}\hat{\sigma}^z_{23} - 576\hat{\sigma}^z_{16}\hat{\sigma}^z_{22} - 576\hat{\sigma}^z_{17}\hat{\sigma}^z_{21} - 576\hat{\sigma}^z_{18}\hat{\sigma}^z_{20} - 576\hat{\sigma}^z_{0}\hat{\sigma}^z_{39} - 864\hat{\sigma}^z_{1}\hat{\sigma}^z_{38} - 864\hat{\sigma}^z_{2}\hat{\sigma}^z_{37} - 864\hat{\sigma}^z_{3}\hat{\sigma}^z_{36} - 1152\hat{\sigma}^z_{4}\hat{\sigma}^z_{35} - \\
&1728\hat{\sigma}^z_{8}\hat{\sigma}^z_{31} - 864\hat{\sigma}^z_{9}\hat{\sigma}^z_{30} - 576\hat{\sigma}^z_{10}\hat{\sigma}^z_{29} - 864\hat{\sigma}^z_{11}\hat{\sigma}^z_{28} - 864\hat{\sigma}^z_{12}\hat{\sigma}^z_{27} - 864\hat{\sigma}^z_{13}\hat{\sigma}^z_{26} - 1152\hat{\sigma}^z_{14}\hat{\sigma}^z_{25} - 576\hat{\sigma}^z_{15}\hat{\sigma}^z_{24} - \\
&576\hat{\sigma}^z_{16}\hat{\sigma}^z_{23} - 576\hat{\sigma}^z_{17}\hat{\sigma}^z_{22} - 576\hat{\sigma}^z_{18}\hat{\sigma}^z_{21} - 288\hat{\sigma}^z_{19}\hat{\sigma}^z_{20} - 288\hat{\sigma}^z_{0}\hat{\sigma}^z_{40} - 864\hat{\sigma}^z_{1}\hat{\sigma}^z_{39} - 1152\hat{\sigma}^z_{2}\hat{\sigma}^z_{38} - 1152\hat{\sigma}^z_{3}\hat{\sigma}^z_{37} - \\
&1152\hat{\sigma}^z_{4}\hat{\sigma}^z_{36} - 5272704\hat{\sigma}^z_{6}\hat{\sigma}^z_{34} - 5272704\hat{\sigma}^z_{8}\hat{\sigma}^z_{32} - 864\hat{\sigma}^z_{9}\hat{\sigma}^z_{31} - 288\hat{\sigma}^z_{10}\hat{\sigma}^z_{30} - 864\hat{\sigma}^z_{11}\hat{\sigma}^z_{29} - 1152\hat{\sigma}^z_{12}\hat{\sigma}^z_{28} - \\
&1152\hat{\sigma}^z_{13}\hat{\sigma}^z_{27} - 1152\hat{\sigma}^z_{14}\hat{\sigma}^z_{26} - 576\hat{\sigma}^z_{15}\hat{\sigma}^z_{25} - 1152\hat{\sigma}^z_{16}\hat{\sigma}^z_{24} - 288\hat{\sigma}^z_{17}\hat{\sigma}^z_{23} - 288\hat{\sigma}^z_{18}\hat{\sigma}^z_{22} - 576\hat{\sigma}^z_{19}\hat{\sigma}^z_{21} - 288\hat{\sigma}^z_{0}\hat{\sigma}^z_{41} - \\
&576\hat{\sigma}^z_{1}\hat{\sigma}^z_{40} - 1152\hat{\sigma}^z_{2}\hat{\sigma}^z_{39} - 864\hat{\sigma}^z_{3}\hat{\sigma}^z_{38} - 864\hat{\sigma}^z_{4}\hat{\sigma}^z_{37} - 5272704\hat{\sigma}^z_{6}\hat{\sigma}^z_{35} - 5272704\hat{\sigma}^z_{7}\hat{\sigma}^z_{34} - 1728\hat{\sigma}^z_{9}\hat{\sigma}^z_{32} - 288\hat{\sigma}^z_{10}\hat{\sigma}^z_{31} - \\
&576\hat{\sigma}^z_{11}\hat{\sigma}^z_{30} - 1152\hat{\sigma}^z_{12}\hat{\sigma}^z_{29} - 864\hat{\sigma}^z_{13}\hat{\sigma}^z_{28} - 864\hat{\sigma}^z_{14}\hat{\sigma}^z_{27} - 576\hat{\sigma}^z_{15}\hat{\sigma}^z_{26} - 864\hat{\sigma}^z_{16}\hat{\sigma}^z_{25} - 1152\hat{\sigma}^z_{17}\hat{\sigma}^z_{24} - 576\hat{\sigma}^z_{18}\hat{\sigma}^z_{23} - \\
&576\hat{\sigma}^z_{19}\hat{\sigma}^z_{22} - 288\hat{\sigma}^z_{0}\hat{\sigma}^z_{42} - 288\hat{\sigma}^z_{1}\hat{\sigma}^z_{41} - 576\hat{\sigma}^z_{2}\hat{\sigma}^z_{40} - 1152\hat{\sigma}^z_{3}\hat{\sigma}^z_{39} - 1152\hat{\sigma}^z_{4}\hat{\sigma}^z_{38} - 5272704\hat{\sigma}^z_{6}\hat{\sigma}^z_{36} - 1728\hat{\sigma}^z_{8}\hat{\sigma}^z_{34} - \\
&5272704\hat{\sigma}^z_{9}\hat{\sigma}^z_{33} - 288\hat{\sigma}^z_{10}\hat{\sigma}^z_{32} - 288\hat{\sigma}^z_{11}\hat{\sigma}^z_{31} - 576\hat{\sigma}^z_{12}\hat{\sigma}^z_{30} - 1152\hat{\sigma}^z_{13}\hat{\sigma}^z_{29} - 1152\hat{\sigma}^z_{14}\hat{\sigma}^z_{28} - 576\hat{\sigma}^z_{15}\hat{\sigma}^z_{27} - 1152\hat{\sigma}^z_{16}\hat{\sigma}^z_{26} - \\
&864\hat{\sigma}^z_{17}\hat{\sigma}^z_{25} - 864\hat{\sigma}^z_{18}\hat{\sigma}^z_{24} - 576\hat{\sigma}^z_{19}\hat{\sigma}^z_{23} - 288\hat{\sigma}^z_{0}\hat{\sigma}^z_{43} - 576\hat{\sigma}^z_{1}\hat{\sigma}^z_{42} - 576\hat{\sigma}^z_{2}\hat{\sigma}^z_{41} - 576\hat{\sigma}^z_{3}\hat{\sigma}^z_{40} - 864\hat{\sigma}^z_{4}\hat{\sigma}^z_{39} - \\
&5272704\hat{\sigma}^z_{8}\hat{\sigma}^z_{35} - 864\hat{\sigma}^z_{9}\hat{\sigma}^z_{34} - 288\hat{\sigma}^z_{10}\hat{\sigma}^z_{33} - 576\hat{\sigma}^z_{11}\hat{\sigma}^z_{32} - 576\hat{\sigma}^z_{12}\hat{\sigma}^z_{31} - 576\hat{\sigma}^z_{13}\hat{\sigma}^z_{30} - 864\hat{\sigma}^z_{14}\hat{\sigma}^z_{29} - 576\hat{\sigma}^z_{15}\hat{\sigma}^z_{28} - \\
&1152\hat{\sigma}^z_{16}\hat{\sigma}^z_{27} - 864\hat{\sigma}^z_{17}\hat{\sigma}^z_{26} - 1152\hat{\sigma}^z_{18}\hat{\sigma}^z_{25} - 864\hat{\sigma}^z_{19}\hat{\sigma}^z_{24} - 576\hat{\sigma}^z_{0}\hat{\sigma}^z_{44} - 576\hat{\sigma}^z_{1}\hat{\sigma}^z_{43} - 576\hat{\sigma}^z_{2}\hat{\sigma}^z_{42} - 576\hat{\sigma}^z_{3}\hat{\sigma}^z_{41} - \\
&288\hat{\sigma}^z_{4}\hat{\sigma}^z_{40} - 5272704\hat{\sigma}^z_{7}\hat{\sigma}^z_{37} - 1728\hat{\sigma}^z_{9}\hat{\sigma}^z_{35} - 576\hat{\sigma}^z_{10}\hat{\sigma}^z_{34} - 576\hat{\sigma}^z_{11}\hat{\sigma}^z_{33} - 576\hat{\sigma}^z_{12}\hat{\sigma}^z_{32} - 576\hat{\sigma}^z_{13}\hat{\sigma}^z_{31} - 288\hat{\sigma}^z_{14}\hat{\sigma}^z_{30} - \\
&576\hat{\sigma}^z_{15}\hat{\sigma}^z_{29} - 864\hat{\sigma}^z_{16}\hat{\sigma}^z_{28} - 864\hat{\sigma}^z_{17}\hat{\sigma}^z_{27} - 864\hat{\sigma}^z_{18}\hat{\sigma}^z_{26} - 1152\hat{\sigma}^z_{19}\hat{\sigma}^z_{25} - 576\hat{\sigma}^z_{0}\hat{\sigma}^z_{45} - 1152\hat{\sigma}^z_{1}\hat{\sigma}^z_{44} - 288\hat{\sigma}^z_{2}\hat{\sigma}^z_{43} - \\
&288\hat{\sigma}^z_{3}\hat{\sigma}^z_{42} - 576\hat{\sigma}^z_{4}\hat{\sigma}^z_{41} - 288\hat{\sigma}^z_{5}\hat{\sigma}^z_{40} - 5272704\hat{\sigma}^z_{7}\hat{\sigma}^z_{38} - 5272704\hat{\sigma}^z_{8}\hat{\sigma}^z_{37} - 5272704\hat{\sigma}^z_{9}\hat{\sigma}^z_{36} - 576\hat{\sigma}^z_{10}\hat{\sigma}^z_{35} - \\
&1152\hat{\sigma}^z_{11}\hat{\sigma}^z_{34} - 288\hat{\sigma}^z_{12}\hat{\sigma}^z_{33} - 288\hat{\sigma}^z_{13}\hat{\sigma}^z_{32} - 576\hat{\sigma}^z_{14}\hat{\sigma}^z_{31} - 288\hat{\sigma}^z_{15}\hat{\sigma}^z_{30} - 864\hat{\sigma}^z_{16}\hat{\sigma}^z_{29} - 1152\hat{\sigma}^z_{17}\hat{\sigma}^z_{28} - 1152\hat{\sigma}^z_{18}\hat{\sigma}^z_{27} - \\
&1152\hat{\sigma}^z_{19}\hat{\sigma}^z_{26} - 576\hat{\sigma}^z_{0}\hat{\sigma}^z_{46} - 864\hat{\sigma}^z_{1}\hat{\sigma}^z_{45} - 1152\hat{\sigma}^z_{2}\hat{\sigma}^z_{44} - 576\hat{\sigma}^z_{3}\hat{\sigma}^z_{43} - 576\hat{\sigma}^z_{4}\hat{\sigma}^z_{42} - 288\hat{\sigma}^z_{5}\hat{\sigma}^z_{41} - 576\hat{\sigma}^z_{6}\hat{\sigma}^z_{40} - \\
&1728\hat{\sigma}^z_{9}\hat{\sigma}^z_{37} - 576\hat{\sigma}^z_{10}\hat{\sigma}^z_{36} - 864\hat{\sigma}^z_{11}\hat{\sigma}^z_{35} - 1152\hat{\sigma}^z_{12}\hat{\sigma}^z_{34} - 576\hat{\sigma}^z_{13}\hat{\sigma}^z_{33} - 576\hat{\sigma}^z_{14}\hat{\sigma}^z_{32} - 288\hat{\sigma}^z_{15}\hat{\sigma}^z_{31} - 576\hat{\sigma}^z_{16}\hat{\sigma}^z_{30} - \\
&1152\hat{\sigma}^z_{17}\hat{\sigma}^z_{29} - 864\hat{\sigma}^z_{18}\hat{\sigma}^z_{28} - 864\hat{\sigma}^z_{19}\hat{\sigma}^z_{27} - 576\hat{\sigma}^z_{0}\hat{\sigma}^z_{47} - 1152\hat{\sigma}^z_{1}\hat{\sigma}^z_{46} - 864\hat{\sigma}^z_{2}\hat{\sigma}^z_{45} - 864\hat{\sigma}^z_{3}\hat{\sigma}^z_{44} - 576\hat{\sigma}^z_{4}\hat{\sigma}^z_{43} - \\
&288\hat{\sigma}^z_{5}\hat{\sigma}^z_{42} - 288\hat{\sigma}^z_{6}\hat{\sigma}^z_{41} - 576\hat{\sigma}^z_{7}\hat{\sigma}^z_{40} - 5272704\hat{\sigma}^z_{8}\hat{\sigma}^z_{39} - 5272704\hat{\sigma}^z_{9}\hat{\sigma}^z_{38} - 576\hat{\sigma}^z_{10}\hat{\sigma}^z_{37} - 1152\hat{\sigma}^z_{11}\hat{\sigma}^z_{36} - 864\hat{\sigma}^z_{12}\hat{\sigma}^z_{35} - \\
&864\hat{\sigma}^z_{13}\hat{\sigma}^z_{34} - 576\hat{\sigma}^z_{14}\hat{\sigma}^z_{33} - 288\hat{\sigma}^z_{15}\hat{\sigma}^z_{32} - 288\hat{\sigma}^z_{16}\hat{\sigma}^z_{31} - 576\hat{\sigma}^z_{17}\hat{\sigma}^z_{30} - 1152\hat{\sigma}^z_{18}\hat{\sigma}^z_{29} - 1152\hat{\sigma}^z_{19}\hat{\sigma}^z_{28} + 1728\hat{\sigma}^z_{20}\hat{\sigma}^z_{27} - \\
&576\hat{\sigma}^z_{0}\hat{\sigma}^z_{48} - 1152\hat{\sigma}^z_{1}\hat{\sigma}^z_{47} - 864\hat{\sigma}^z_{2}\hat{\sigma}^z_{46} - 1152\hat{\sigma}^z_{3}\hat{\sigma}^z_{45} - 864\hat{\sigma}^z_{4}\hat{\sigma}^z_{44} - 288\hat{\sigma}^z_{5}\hat{\sigma}^z_{43} - 576\hat{\sigma}^z_{6}\hat{\sigma}^z_{42} - 576\hat{\sigma}^z_{7}\hat{\sigma}^z_{41} - 576\hat{\sigma}^z_{8}\hat{\sigma}^z_{40} - \\
&5272704\hat{\sigma}^z_{9}\hat{\sigma}^z_{39} - 576\hat{\sigma}^z_{10}\hat{\sigma}^z_{38} - 1152\hat{\sigma}^z_{11}\hat{\sigma}^z_{37} - 864\hat{\sigma}^z_{12}\hat{\sigma}^z_{36} - 1152\hat{\sigma}^z_{13}\hat{\sigma}^z_{35} - 864\hat{\sigma}^z_{14}\hat{\sigma}^z_{34} - 288\hat{\sigma}^z_{15}\hat{\sigma}^z_{33} - 576\hat{\sigma}^z_{16}\hat{\sigma}^z_{32} - \\
&576\hat{\sigma}^z_{17}\hat{\sigma}^z_{31} - 576\hat{\sigma}^z_{18}\hat{\sigma}^z_{30} - 864\hat{\sigma}^z_{19}\hat{\sigma}^z_{29} - 576\hat{\sigma}^z_{0}\hat{\sigma}^z_{49} - 864\hat{\sigma}^z_{1}\hat{\sigma}^z_{48} - 864\hat{\sigma}^z_{2}\hat{\sigma}^z_{47} - 864\hat{\sigma}^z_{3}\hat{\sigma}^z_{46} - 1152\hat{\sigma}^z_{4}\hat{\sigma}^z_{45} - \\
&576\hat{\sigma}^z_{5}\hat{\sigma}^z_{44} - 576\hat{\sigma}^z_{6}\hat{\sigma}^z_{43} - 576\hat{\sigma}^z_{7}\hat{\sigma}^z_{42} - 576\hat{\sigma}^z_{8}\hat{\sigma}^z_{41} - 288\hat{\sigma}^z_{9}\hat{\sigma}^z_{40} - 576\hat{\sigma}^z_{10}\hat{\sigma}^z_{39} - 864\hat{\sigma}^z_{11}\hat{\sigma}^z_{38} - 864\hat{\sigma}^z_{12}\hat{\sigma}^z_{37} - 864\hat{\sigma}^z_{13}\hat{\sigma}^z_{36} - \\
&1152\hat{\sigma}^z_{14}\hat{\sigma}^z_{35} - 576\hat{\sigma}^z_{15}\hat{\sigma}^z_{34} - 576\hat{\sigma}^z_{16}\hat{\sigma}^z_{33} - 576\hat{\sigma}^z_{17}\hat{\sigma}^z_{32} - 576\hat{\sigma}^z_{18}\hat{\sigma}^z_{31} - 288\hat{\sigma}^z_{19}\hat{\sigma}^z_{30} + 1728\hat{\sigma}^z_{20}\hat{\sigma}^z_{29} - 288\hat{\sigma}^z_{0}\hat{\sigma}^z_{50} - \\
&864\hat{\sigma}^z_{1}\hat{\sigma}^z_{49} - 1152\hat{\sigma}^z_{2}\hat{\sigma}^z_{48} - 1152\hat{\sigma}^z_{3}\hat{\sigma}^z_{47} - 1152\hat{\sigma}^z_{4}\hat{\sigma}^z_{46} - 576\hat{\sigma}^z_{5}\hat{\sigma}^z_{45} - 1152\hat{\sigma}^z_{6}\hat{\sigma}^z_{44} - 288\hat{\sigma}^z_{7}\hat{\sigma}^z_{43} - 288\hat{\sigma}^z_{8}\hat{\sigma}^z_{42} - \\
&576\hat{\sigma}^z_{9}\hat{\sigma}^z_{41} - 5272704\hat{\sigma}^z_{10}\hat{\sigma}^z_{40} - 864\hat{\sigma}^z_{11}\hat{\sigma}^z_{39} - 1152\hat{\sigma}^z_{12}\hat{\sigma}^z_{38} - 1152\hat{\sigma}^z_{13}\hat{\sigma}^z_{37} - 1152\hat{\sigma}^z_{14}\hat{\sigma}^z_{36} - 576\hat{\sigma}^z_{15}\hat{\sigma}^z_{35} - 1152\hat{\sigma}^z_{16}\hat{\sigma}^z_{34} - \\
&288\hat{\sigma}^z_{17}\hat{\sigma}^z_{33} - 288\hat{\sigma}^z_{18}\hat{\sigma}^z_{32} - 576\hat{\sigma}^z_{19}\hat{\sigma}^z_{31} + 576\hat{\sigma}^z_{20}\hat{\sigma}^z_{30} + 1728\hat{\sigma}^z_{21}\hat{\sigma}^z_{29} - 288\hat{\sigma}^z_{0}\hat{\sigma}^z_{51} - 576\hat{\sigma}^z_{1}\hat{\sigma}^z_{50} - 1152\hat{\sigma}^z_{2}\hat{\sigma}^z_{49} - \\
&864\hat{\sigma}^z_{3}\hat{\sigma}^z_{48} - 864\hat{\sigma}^z_{4}\hat{\sigma}^z_{47} - 576\hat{\sigma}^z_{5}\hat{\sigma}^z_{46} - 864\hat{\sigma}^z_{6}\hat{\sigma}^z_{45} - 1152\hat{\sigma}^z_{7}\hat{\sigma}^z_{44} - 576\hat{\sigma}^z_{8}\hat{\sigma}^z_{43} - 576\hat{\sigma}^z_{9}\hat{\sigma}^z_{42} - 5272704\hat{\sigma}^z_{10}\hat{\sigma}^z_{41} - \\
&5272704\hat{\sigma}^z_{11}\hat{\sigma}^z_{40} - 1152\hat{\sigma}^z_{12}\hat{\sigma}^z_{39} - 864\hat{\sigma}^z_{13}\hat{\sigma}^z_{38} - 864\hat{\sigma}^z_{14}\hat{\sigma}^z_{37} - 576\hat{\sigma}^z_{15}\hat{\sigma}^z_{36} - 864\hat{\sigma}^z_{16}\hat{\sigma}^z_{35} - 1152\hat{\sigma}^z_{17}\hat{\sigma}^z_{34} - 576\hat{\sigma}^z_{18}\hat{\sigma}^z_{33} - \\
&576\hat{\sigma}^z_{19}\hat{\sigma}^z_{32} - 288\hat{\sigma}^z_{0}\hat{\sigma}^z_{52} - 288\hat{\sigma}^z_{1}\hat{\sigma}^z_{51} - 576\hat{\sigma}^z_{2}\hat{\sigma}^z_{50} - 1152\hat{\sigma}^z_{3}\hat{\sigma}^z_{49} - 1152\hat{\sigma}^z_{4}\hat{\sigma}^z_{48} - 576\hat{\sigma}^z_{5}\hat{\sigma}^z_{47} - 1152\hat{\sigma}^z_{6}\hat{\sigma}^z_{46} - \\
&864\hat{\sigma}^z_{7}\hat{\sigma}^z_{45} - 864\hat{\sigma}^z_{8}\hat{\sigma}^z_{44} - 576\hat{\sigma}^z_{9}\hat{\sigma}^z_{43} - 5272704\hat{\sigma}^z_{10}\hat{\sigma}^z_{42} - 864\hat{\sigma}^z_{12}\hat{\sigma}^z_{40} - 1152\hat{\sigma}^z_{13}\hat{\sigma}^z_{39} - 1152\hat{\sigma}^z_{14}\hat{\sigma}^z_{38} - 576\hat{\sigma}^z_{15}\hat{\sigma}^z_{37} - \\
&1152\hat{\sigma}^z_{16}\hat{\sigma}^z_{36} - 864\hat{\sigma}^z_{17}\hat{\sigma}^z_{35} - 864\hat{\sigma}^z_{18}\hat{\sigma}^z_{34} - 576\hat{\sigma}^z_{19}\hat{\sigma}^z_{33} + 576\hat{\sigma}^z_{21}\hat{\sigma}^z_{31} - 288\hat{\sigma}^z_{0}\hat{\sigma}^z_{53} - 576\hat{\sigma}^z_{1}\hat{\sigma}^z_{52} - 576\hat{\sigma}^z_{2}\hat{\sigma}^z_{51} -
\end{aligned}
$$

$$576\hat{\sigma}^z_{3}\hat{\sigma}^z_{50} - 864\hat{\sigma}^z_{4}\hat{\sigma}^z_{49} - 576\hat{\sigma}^z_{5}\hat{\sigma}^z_{48} - 1152\hat{\sigma}^z_{6}\hat{\sigma}^z_{47} - 864\hat{\sigma}^z_{7}\hat{\sigma}^z_{46} - 1152\hat{\sigma}^z_{8}\hat{\sigma}^z_{45} - 864\hat{\sigma}^z_{9}\hat{\sigma}^z_{44} - 5272704\hat{\sigma}^z_{10}\hat{\sigma}^z_{43} -$$
$$5272704\hat{\sigma}^z_{12}\hat{\sigma}^z_{41} - 1728\hat{\sigma}^z_{13}\hat{\sigma}^z_{40} - 864\hat{\sigma}^z_{14}\hat{\sigma}^z_{39} - 576\hat{\sigma}^z_{15}\hat{\sigma}^z_{38} - 1152\hat{\sigma}^z_{16}\hat{\sigma}^z_{37} - 864\hat{\sigma}^z_{17}\hat{\sigma}^z_{36} - 1152\hat{\sigma}^z_{18}\hat{\sigma}^z_{35} - 864\hat{\sigma}^z_{19}\hat{\sigma}^z_{34} +$$
$$1728\hat{\sigma}^z_{24}\hat{\sigma}^z_{29} - 576\hat{\sigma}^z_{0}\hat{\sigma}^z_{54} - 576\hat{\sigma}^z_{1}\hat{\sigma}^z_{53} - 576\hat{\sigma}^z_{2}\hat{\sigma}^z_{52} - 576\hat{\sigma}^z_{3}\hat{\sigma}^z_{51} - 288\hat{\sigma}^z_{4}\hat{\sigma}^z_{50} - 576\hat{\sigma}^z_{5}\hat{\sigma}^z_{49} - 864\hat{\sigma}^z_{6}\hat{\sigma}^z_{48} - 864\hat{\sigma}^z_{7}\hat{\sigma}^z_{47} -$$
$$864\hat{\sigma}^z_{8}\hat{\sigma}^z_{46} - 1152\hat{\sigma}^z_{9}\hat{\sigma}^z_{45} - 1728\hat{\sigma}^z_{13}\hat{\sigma}^z_{41} - 864\hat{\sigma}^z_{14}\hat{\sigma}^z_{40} - 576\hat{\sigma}^z_{15}\hat{\sigma}^z_{39} - 864\hat{\sigma}^z_{16}\hat{\sigma}^z_{38} - 864\hat{\sigma}^z_{17}\hat{\sigma}^z_{37} - 864\hat{\sigma}^z_{18}\hat{\sigma}^z_{36} -$$
$$1152\hat{\sigma}^z_{19}\hat{\sigma}^z_{35} + 576\hat{\sigma}^z_{20}\hat{\sigma}^z_{34} + 576\hat{\sigma}^z_{22}\hat{\sigma}^z_{32} + 576\hat{\sigma}^z_{24}\hat{\sigma}^z_{30} - 576\hat{\sigma}^z_{0}\hat{\sigma}^z_{55} - 1152\hat{\sigma}^z_{1}\hat{\sigma}^z_{54} - 288\hat{\sigma}^z_{2}\hat{\sigma}^z_{53} - 288\hat{\sigma}^z_{3}\hat{\sigma}^z_{52} -$$
$$576\hat{\sigma}^z_{4}\hat{\sigma}^z_{51} - 288\hat{\sigma}^z_{5}\hat{\sigma}^z_{50} - 864\hat{\sigma}^z_{6}\hat{\sigma}^z_{49} - 1152\hat{\sigma}^z_{7}\hat{\sigma}^z_{48} - 1152\hat{\sigma}^z_{8}\hat{\sigma}^z_{47} - 1152\hat{\sigma}^z_{9}\hat{\sigma}^z_{46} - 5272704\hat{\sigma}^z_{11}\hat{\sigma}^z_{44} - 5272704\hat{\sigma}^z_{13}\hat{\sigma}^z_{42} -$$
$$864\hat{\sigma}^z_{14}\hat{\sigma}^z_{41} - 288\hat{\sigma}^z_{15}\hat{\sigma}^z_{40} - 864\hat{\sigma}^z_{16}\hat{\sigma}^z_{39} - 1152\hat{\sigma}^z_{17}\hat{\sigma}^z_{38} - 1152\hat{\sigma}^z_{18}\hat{\sigma}^z_{37} - 1152\hat{\sigma}^z_{19}\hat{\sigma}^z_{36} - 576\hat{\sigma}^z_{0}\hat{\sigma}^z_{56} - 864\hat{\sigma}^z_{1}\hat{\sigma}^z_{55} -$$
$$1152\hat{\sigma}^z_{2}\hat{\sigma}^z_{54} - 576\hat{\sigma}^z_{3}\hat{\sigma}^z_{53} - 576\hat{\sigma}^z_{4}\hat{\sigma}^z_{52} - 288\hat{\sigma}^z_{5}\hat{\sigma}^z_{51} - 576\hat{\sigma}^z_{6}\hat{\sigma}^z_{50} - 1152\hat{\sigma}^z_{7}\hat{\sigma}^z_{49} - 864\hat{\sigma}^z_{8}\hat{\sigma}^z_{48} - 864\hat{\sigma}^z_{9}\hat{\sigma}^z_{47} -$$
$$5272704\hat{\sigma}^z_{11}\hat{\sigma}^z_{45} - 5272704\hat{\sigma}^z_{12}\hat{\sigma}^z_{44} - 1728\hat{\sigma}^z_{14}\hat{\sigma}^z_{42} - 288\hat{\sigma}^z_{15}\hat{\sigma}^z_{41} - 576\hat{\sigma}^z_{16}\hat{\sigma}^z_{40} - 1152\hat{\sigma}^z_{17}\hat{\sigma}^z_{39} - 864\hat{\sigma}^z_{18}\hat{\sigma}^z_{38} -$$
$$864\hat{\sigma}^z_{19}\hat{\sigma}^z_{37} + 576\hat{\sigma}^z_{21}\hat{\sigma}^z_{35} + 576\hat{\sigma}^z_{22}\hat{\sigma}^z_{34} + 576\hat{\sigma}^z_{23}\hat{\sigma}^z_{33} + 576\hat{\sigma}^z_{24}\hat{\sigma}^z_{32} + 576\hat{\sigma}^z_{25}\hat{\sigma}^z_{31} - 576\hat{\sigma}^z_{0}\hat{\sigma}^z_{57} - 1152\hat{\sigma}^z_{1}\hat{\sigma}^z_{56} -$$
$$864\hat{\sigma}^z_{2}\hat{\sigma}^z_{55} - 864\hat{\sigma}^z_{3}\hat{\sigma}^z_{54} - 576\hat{\sigma}^z_{4}\hat{\sigma}^z_{53} - 288\hat{\sigma}^z_{5}\hat{\sigma}^z_{52} - 288\hat{\sigma}^z_{6}\hat{\sigma}^z_{51} - 576\hat{\sigma}^z_{7}\hat{\sigma}^z_{50} - 1152\hat{\sigma}^z_{8}\hat{\sigma}^z_{49} - 1152\hat{\sigma}^z_{9}\hat{\sigma}^z_{48} -$$
$$5272704\hat{\sigma}^z_{11}\hat{\sigma}^z_{46} - 1728\hat{\sigma}^z_{13}\hat{\sigma}^z_{44} - 5272704\hat{\sigma}^z_{14}\hat{\sigma}^z_{43} - 288\hat{\sigma}^z_{15}\hat{\sigma}^z_{42} - 288\hat{\sigma}^z_{16}\hat{\sigma}^z_{41} - 576\hat{\sigma}^z_{17}\hat{\sigma}^z_{40} - 1152\hat{\sigma}^z_{18}\hat{\sigma}^z_{39} -$$
$$1152\hat{\sigma}^z_{19}\hat{\sigma}^z_{38} + 576\hat{\sigma}^z_{20}\hat{\sigma}^z_{37} + 576\hat{\sigma}^z_{27}\hat{\sigma}^z_{30} - 576\hat{\sigma}^z_{0}\hat{\sigma}^z_{58} - 1152\hat{\sigma}^z_{1}\hat{\sigma}^z_{57} - 864\hat{\sigma}^z_{2}\hat{\sigma}^z_{56} - 1152\hat{\sigma}^z_{3}\hat{\sigma}^z_{55} - 864\hat{\sigma}^z_{4}\hat{\sigma}^z_{54} -$$
$$288\hat{\sigma}^z_{5}\hat{\sigma}^z_{53} - 576\hat{\sigma}^z_{6}\hat{\sigma}^z_{52} - 576\hat{\sigma}^z_{7}\hat{\sigma}^z_{51} - 576\hat{\sigma}^z_{8}\hat{\sigma}^z_{50} - 864\hat{\sigma}^z_{9}\hat{\sigma}^z_{49} - 5272704\hat{\sigma}^z_{13}\hat{\sigma}^z_{45} - 864\hat{\sigma}^z_{14}\hat{\sigma}^z_{44} - 288\hat{\sigma}^z_{15}\hat{\sigma}^z_{43} -$$
$$576\hat{\sigma}^z_{16}\hat{\sigma}^z_{42} - 576\hat{\sigma}^z_{17}\hat{\sigma}^z_{41} - 576\hat{\sigma}^z_{18}\hat{\sigma}^z_{40} - 864\hat{\sigma}^z_{19}\hat{\sigma}^z_{39} + 576\hat{\sigma}^z_{22}\hat{\sigma}^z_{36} + 576\hat{\sigma}^z_{23}\hat{\sigma}^z_{35} + 1152\hat{\sigma}^z_{24}\hat{\sigma}^z_{34} + 576\hat{\sigma}^z_{25}\hat{\sigma}^z_{33} +$$
$$576\hat{\sigma}^z_{26}\hat{\sigma}^z_{32} - 576\hat{\sigma}^z_{0}\hat{\sigma}^z_{59} - 864\hat{\sigma}^z_{1}\hat{\sigma}^z_{58} - 864\hat{\sigma}^z_{2}\hat{\sigma}^z_{57} - 864\hat{\sigma}^z_{3}\hat{\sigma}^z_{56} - 1152\hat{\sigma}^z_{4}\hat{\sigma}^z_{55} - 576\hat{\sigma}^z_{5}\hat{\sigma}^z_{54} - 576\hat{\sigma}^z_{6}\hat{\sigma}^z_{53} - 576\hat{\sigma}^z_{7}\hat{\sigma}^z_{52} -$$
$$576\hat{\sigma}^z_{8}\hat{\sigma}^z_{51} - 288\hat{\sigma}^z_{9}\hat{\sigma}^z_{50} - 5272704\hat{\sigma}^z_{12}\hat{\sigma}^z_{47} - 1728\hat{\sigma}^z_{14}\hat{\sigma}^z_{45} - 576\hat{\sigma}^z_{15}\hat{\sigma}^z_{44} - 576\hat{\sigma}^z_{16}\hat{\sigma}^z_{43} - 576\hat{\sigma}^z_{17}\hat{\sigma}^z_{42} - 576\hat{\sigma}^z_{18}\hat{\sigma}^z_{41} -$$
$$288\hat{\sigma}^z_{19}\hat{\sigma}^z_{40} + 576\hat{\sigma}^z_{20}\hat{\sigma}^z_{39} + 576\hat{\sigma}^z_{21}\hat{\sigma}^z_{38} + 576\hat{\sigma}^z_{23}\hat{\sigma}^z_{36} + 576\hat{\sigma}^z_{26}\hat{\sigma}^z_{33} + 576\hat{\sigma}^z_{28}\hat{\sigma}^z_{31} + 576\hat{\sigma}^z_{29}\hat{\sigma}^z_{30} - 864\hat{\sigma}^z_{1}\hat{\sigma}^z_{59} -$$
$$1152\hat{\sigma}^z_{2}\hat{\sigma}^z_{58} - 1152\hat{\sigma}^z_{3}\hat{\sigma}^z_{57} - 1152\hat{\sigma}^z_{4}\hat{\sigma}^z_{56} - 576\hat{\sigma}^z_{5}\hat{\sigma}^z_{55} - 1152\hat{\sigma}^z_{6}\hat{\sigma}^z_{54} - 288\hat{\sigma}^z_{7}\hat{\sigma}^z_{53} - 288\hat{\sigma}^z_{8}\hat{\sigma}^z_{52} - 576\hat{\sigma}^z_{9}\hat{\sigma}^z_{51} -$$
$$288\hat{\sigma}^z_{10}\hat{\sigma}^z_{50} - 5272704\hat{\sigma}^z_{12}\hat{\sigma}^z_{48} - 5272704\hat{\sigma}^z_{13}\hat{\sigma}^z_{47} - 5272704\hat{\sigma}^z_{14}\hat{\sigma}^z_{46} - 576\hat{\sigma}^z_{15}\hat{\sigma}^z_{45} - 1152\hat{\sigma}^z_{16}\hat{\sigma}^z_{44} - 288\hat{\sigma}^z_{17}\hat{\sigma}^z_{43} -$$
$$288\hat{\sigma}^z_{18}\hat{\sigma}^z_{42} - 576\hat{\sigma}^z_{19}\hat{\sigma}^z_{41} + 576\hat{\sigma}^z_{20}\hat{\sigma}^z_{40} + 576\hat{\sigma}^z_{21}\hat{\sigma}^z_{39} + 576\hat{\sigma}^z_{22}\hat{\sigma}^z_{38} + 576\hat{\sigma}^z_{23}\hat{\sigma}^z_{37} + 576\hat{\sigma}^z_{24}\hat{\sigma}^z_{36} + 1152\hat{\sigma}^z_{25}\hat{\sigma}^z_{35} +$$
$$576\hat{\sigma}^z_{26}\hat{\sigma}^z_{34} + 576\hat{\sigma}^z_{27}\hat{\sigma}^z_{33} + 576\hat{\sigma}^z_{28}\hat{\sigma}^z_{32} + 576\hat{\sigma}^z_{29}\hat{\sigma}^z_{31} - 1152\hat{\sigma}^z_{2}\hat{\sigma}^z_{59} - 864\hat{\sigma}^z_{3}\hat{\sigma}^z_{58} - 864\hat{\sigma}^z_{4}\hat{\sigma}^z_{57} - 576\hat{\sigma}^z_{5}\hat{\sigma}^z_{56} - 864\hat{\sigma}^z_{6}\hat{\sigma}^z_{55} -$$
$$1152\hat{\sigma}^z_{7}\hat{\sigma}^z_{54} - 576\hat{\sigma}^z_{8}\hat{\sigma}^z_{53} - 576\hat{\sigma}^z_{9}\hat{\sigma}^z_{52} - 288\hat{\sigma}^z_{10}\hat{\sigma}^z_{51} - 576\hat{\sigma}^z_{11}\hat{\sigma}^z_{50} - 1728\hat{\sigma}^z_{14}\hat{\sigma}^z_{47} - 576\hat{\sigma}^z_{15}\hat{\sigma}^z_{46} - 864\hat{\sigma}^z_{16}\hat{\sigma}^z_{45} -$$
$$1152\hat{\sigma}^z_{17}\hat{\sigma}^z_{44} - 576\hat{\sigma}^z_{18}\hat{\sigma}^z_{43} - 576\hat{\sigma}^z_{19}\hat{\sigma}^z_{42} + 576\hat{\sigma}^z_{24}\hat{\sigma}^z_{37} + 576\hat{\sigma}^z_{25}\hat{\sigma}^z_{36} + 576\hat{\sigma}^z_{26}\hat{\sigma}^z_{35} + 576\hat{\sigma}^z_{27}\hat{\sigma}^z_{34} - 1152\hat{\sigma}^z_{3}\hat{\sigma}^z_{59} -$$
$$1152\hat{\sigma}^z_{4}\hat{\sigma}^z_{58} - 576\hat{\sigma}^z_{5}\hat{\sigma}^z_{57} - 1152\hat{\sigma}^z_{6}\hat{\sigma}^z_{56} - 864\hat{\sigma}^z_{7}\hat{\sigma}^z_{55} - 864\hat{\sigma}^z_{8}\hat{\sigma}^z_{54} - 576\hat{\sigma}^z_{9}\hat{\sigma}^z_{53} - 288\hat{\sigma}^z_{10}\hat{\sigma}^z_{52} - 288\hat{\sigma}^z_{11}\hat{\sigma}^z_{51} -$$
$$576\hat{\sigma}^z_{12}\hat{\sigma}^z_{50} - 5272704\hat{\sigma}^z_{13}\hat{\sigma}^z_{49} - 5272704\hat{\sigma}^z_{14}\hat{\sigma}^z_{48} - 576\hat{\sigma}^z_{15}\hat{\sigma}^z_{47} - 1152\hat{\sigma}^z_{16}\hat{\sigma}^z_{46} - 864\hat{\sigma}^z_{17}\hat{\sigma}^z_{45} - 864\hat{\sigma}^z_{18}\hat{\sigma}^z_{44} -$$
$$576\hat{\sigma}^z_{19}\hat{\sigma}^z_{43} + 576\hat{\sigma}^z_{21}\hat{\sigma}^z_{41} + 576\hat{\sigma}^z_{24}\hat{\sigma}^z_{38} + 576\hat{\sigma}^z_{25}\hat{\sigma}^z_{37} + 1152\hat{\sigma}^z_{26}\hat{\sigma}^z_{36} + 576\hat{\sigma}^z_{27}\hat{\sigma}^z_{35} + 576\hat{\sigma}^z_{28}\hat{\sigma}^z_{34} - 864\hat{\sigma}^z_{4}\hat{\sigma}^z_{59} -$$
$$576\hat{\sigma}^z_{5}\hat{\sigma}^z_{58} - 1152\hat{\sigma}^z_{6}\hat{\sigma}^z_{57} - 864\hat{\sigma}^z_{7}\hat{\sigma}^z_{56} - 1152\hat{\sigma}^z_{8}\hat{\sigma}^z_{55} - 864\hat{\sigma}^z_{9}\hat{\sigma}^z_{54} - 288\hat{\sigma}^z_{10}\hat{\sigma}^z_{53} - 576\hat{\sigma}^z_{11}\hat{\sigma}^z_{52} - 576\hat{\sigma}^z_{12}\hat{\sigma}^z_{51} -$$
$$576\hat{\sigma}^z_{13}\hat{\sigma}^z_{50} - 5272704\hat{\sigma}^z_{14}\hat{\sigma}^z_{49} - 576\hat{\sigma}^z_{15}\hat{\sigma}^z_{48} - 1152\hat{\sigma}^z_{16}\hat{\sigma}^z_{47} - 864\hat{\sigma}^z_{17}\hat{\sigma}^z_{46} - 1152\hat{\sigma}^z_{18}\hat{\sigma}^z_{45} - 864\hat{\sigma}^z_{19}\hat{\sigma}^z_{44} + 576\hat{\sigma}^z_{24}\hat{\sigma}^z_{39} +$$
$$576\hat{\sigma}^z_{25}\hat{\sigma}^z_{38} + 576\hat{\sigma}^z_{26}\hat{\sigma}^z_{37} + 576\hat{\sigma}^z_{27}\hat{\sigma}^z_{36} + 576\hat{\sigma}^z_{28}\hat{\sigma}^z_{35} + 576\hat{\sigma}^z_{29}\hat{\sigma}^z_{34} - 576\hat{\sigma}^z_{5}\hat{\sigma}^z_{59} - 864\hat{\sigma}^z_{6}\hat{\sigma}^z_{58} - 864\hat{\sigma}^z_{7}\hat{\sigma}^z_{57} - 864\hat{\sigma}^z_{8}\hat{\sigma}^z_{56} -$$
$$1152\hat{\sigma}^z_{9}\hat{\sigma}^z_{55} - 576\hat{\sigma}^z_{10}\hat{\sigma}^z_{54} - 576\hat{\sigma}^z_{11}\hat{\sigma}^z_{53} - 576\hat{\sigma}^z_{12}\hat{\sigma}^z_{52} - 576\hat{\sigma}^z_{13}\hat{\sigma}^z_{51} - 288\hat{\sigma}^z_{14}\hat{\sigma}^z_{50} - 576\hat{\sigma}^z_{15}\hat{\sigma}^z_{49} - 864\hat{\sigma}^z_{16}\hat{\sigma}^z_{48} -$$
$$864\hat{\sigma}^z_{17}\hat{\sigma}^z_{47} - 864\hat{\sigma}^z_{18}\hat{\sigma}^z_{46} - 1152\hat{\sigma}^z_{19}\hat{\sigma}^z_{45} + 576\hat{\sigma}^z_{20}\hat{\sigma}^z_{44} + 576\hat{\sigma}^z_{22}\hat{\sigma}^z_{42} + 576\hat{\sigma}^z_{24}\hat{\sigma}^z_{40} + 576\hat{\sigma}^z_{25}\hat{\sigma}^z_{39} + 576\hat{\sigma}^z_{26}\hat{\sigma}^z_{38} +$$
$$1152\hat{\sigma}^z_{27}\hat{\sigma}^z_{37} + 576\hat{\sigma}^z_{28}\hat{\sigma}^z_{36} + 576\hat{\sigma}^z_{29}\hat{\sigma}^z_{35} - 864\hat{\sigma}^z_{6}\hat{\sigma}^z_{59} - 1152\hat{\sigma}^z_{7}\hat{\sigma}^z_{58} - 1152\hat{\sigma}^z_{8}\hat{\sigma}^z_{57} - 1152\hat{\sigma}^z_{9}\hat{\sigma}^z_{56} - 576\hat{\sigma}^z_{10}\hat{\sigma}^z_{55} -$$
$$1152\hat{\sigma}^z_{11}\hat{\sigma}^z_{54} - 288\hat{\sigma}^z_{12}\hat{\sigma}^z_{53} - 288\hat{\sigma}^z_{13}\hat{\sigma}^z_{52} - 576\hat{\sigma}^z_{14}\hat{\sigma}^z_{51} - 5272704\hat{\sigma}^z_{15}\hat{\sigma}^z_{50} - 864\hat{\sigma}^z_{16}\hat{\sigma}^z_{49} - 1152\hat{\sigma}^z_{17}\hat{\sigma}^z_{48} - 1152\hat{\sigma}^z_{18}\hat{\sigma}^z_{47} -$$
$$1152\hat{\sigma}^z_{19}\hat{\sigma}^z_{46} - 1152\hat{\sigma}^z_{7}\hat{\sigma}^z_{59} - 864\hat{\sigma}^z_{8}\hat{\sigma}^z_{58} - 864\hat{\sigma}^z_{9}\hat{\sigma}^z_{57} - 576\hat{\sigma}^z_{10}\hat{\sigma}^z_{56} - 864\hat{\sigma}^z_{11}\hat{\sigma}^z_{55} - 1152\hat{\sigma}^z_{12}\hat{\sigma}^z_{54} - 576\hat{\sigma}^z_{13}\hat{\sigma}^z_{53} -$$
$$576\hat{\sigma}^z_{14}\hat{\sigma}^z_{52} - 5272704\hat{\sigma}^z_{15}\hat{\sigma}^z_{51} - 5272704\hat{\sigma}^z_{16}\hat{\sigma}^z_{50} - 1152\hat{\sigma}^z_{17}\hat{\sigma}^z_{49} - 864\hat{\sigma}^z_{18}\hat{\sigma}^z_{48} - 864\hat{\sigma}^z_{19}\hat{\sigma}^z_{47} + 576\hat{\sigma}^z_{21}\hat{\sigma}^z_{45} +$$
$$576\hat{\sigma}^z_{22}\hat{\sigma}^z_{44} + 576\hat{\sigma}^z_{23}\hat{\sigma}^z_{43} + 576\hat{\sigma}^z_{24}\hat{\sigma}^z_{42} + 576\hat{\sigma}^z_{25}\hat{\sigma}^z_{41} + 576\hat{\sigma}^z_{27}\hat{\sigma}^z_{39} + 1152\hat{\sigma}^z_{28}\hat{\sigma}^z_{38} + 576\hat{\sigma}^z_{29}\hat{\sigma}^z_{37} - 1152\hat{\sigma}^z_{8}\hat{\sigma}^z_{59} -$$
$$1152\hat{\sigma}^z_{9}\hat{\sigma}^z_{58} - 576\hat{\sigma}^z_{10}\hat{\sigma}^z_{57} - 1152\hat{\sigma}^z_{11}\hat{\sigma}^z_{56} - 864\hat{\sigma}^z_{12}\hat{\sigma}^z_{55} - 864\hat{\sigma}^z_{13}\hat{\sigma}^z_{54} - 576\hat{\sigma}^z_{14}\hat{\sigma}^z_{53} - 5272704\hat{\sigma}^z_{15}\hat{\sigma}^z_{52} - 864\hat{\sigma}^z_{17}\hat{\sigma}^z_{50} -$$
$$1152\hat{\sigma}^z_{18}\hat{\sigma}^z_{49} - 1152\hat{\sigma}^z_{19}\hat{\sigma}^z_{48} + 576\hat{\sigma}^z_{20}\hat{\sigma}^z_{47} + 576\hat{\sigma}^z_{27}\hat{\sigma}^z_{40} + 576\hat{\sigma}^z_{28}\hat{\sigma}^z_{39} + 576\hat{\sigma}^z_{29}\hat{\sigma}^z_{38} + 1728\hat{\sigma}^z_{30}\hat{\sigma}^z_{37} - 864\hat{\sigma}^z_{9}\hat{\sigma}^z_{59} -$$
$$576\hat{\sigma}^z_{10}\hat{\sigma}^z_{58} - 1152\hat{\sigma}^z_{11}\hat{\sigma}^z_{57} - 864\hat{\sigma}^z_{12}\hat{\sigma}^z_{56} - 1152\hat{\sigma}^z_{13}\hat{\sigma}^z_{55} - 864\hat{\sigma}^z_{14}\hat{\sigma}^z_{54} - 5272704\hat{\sigma}^z_{15}\hat{\sigma}^z_{53} - 5272704\hat{\sigma}^z_{17}\hat{\sigma}^z_{51} -$$
$$1728\hat{\sigma}^z_{18}\hat{\sigma}^z_{50} - 864\hat{\sigma}^z_{19}\hat{\sigma}^z_{49} + 576\hat{\sigma}^z_{22}\hat{\sigma}^z_{46} + 576\hat{\sigma}^z_{23}\hat{\sigma}^z_{45} + 1152\hat{\sigma}^z_{24}\hat{\sigma}^z_{44} + 576\hat{\sigma}^z_{25}\hat{\sigma}^z_{43} + 576\hat{\sigma}^z_{26}\hat{\sigma}^z_{42} + 1152\hat{\sigma}^z_{29}\hat{\sigma}^z_{39} -$$
$$576\hat{\sigma}^z_{10}\hat{\sigma}^z_{59} - 864\hat{\sigma}^z_{11}\hat{\sigma}^z_{58} - 864\hat{\sigma}^z_{12}\hat{\sigma}^z_{57} - 864\hat{\sigma}^z_{13}\hat{\sigma}^z_{56} - 1152\hat{\sigma}^z_{14}\hat{\sigma}^z_{55} - 1728\hat{\sigma}^z_{18}\hat{\sigma}^z_{51} - 864\hat{\sigma}^z_{19}\hat{\sigma}^z_{50} + 576\hat{\sigma}^z_{20}\hat{\sigma}^z_{49} +$$
$$576\hat{\sigma}^z_{21}\hat{\sigma}^z_{48} + 576\hat{\sigma}^z_{23}\hat{\sigma}^z_{46} + 576\hat{\sigma}^z_{26}\hat{\sigma}^z_{43} + 576\hat{\sigma}^z_{28}\hat{\sigma}^z_{41} + 576\hat{\sigma}^z_{29}\hat{\sigma}^z_{40} + 1728\hat{\sigma}^z_{30}\hat{\sigma}^z_{39} - 864\hat{\sigma}^z_{11}\hat{\sigma}^z_{59} - 1152\hat{\sigma}^z_{12}\hat{\sigma}^z_{58} -$$
$$1152\hat{\sigma}^z_{13}\hat{\sigma}^z_{57} - 1152\hat{\sigma}^z_{14}\hat{\sigma}^z_{56} - 5272704\hat{\sigma}^z_{16}\hat{\sigma}^z_{54} - 5272704\hat{\sigma}^z_{18}\hat{\sigma}^z_{52} - 864\hat{\sigma}^z_{19}\hat{\sigma}^z_{51} + 576\hat{\sigma}^z_{20}\hat{\sigma}^z_{50} + 576\hat{\sigma}^z_{21}\hat{\sigma}^z_{49} +$$
$$576\hat{\sigma}^z_{22}\hat{\sigma}^z_{48} + 576\hat{\sigma}^z_{23}\hat{\sigma}^z_{47} + 576\hat{\sigma}^z_{24}\hat{\sigma}^z_{46} + 1152\hat{\sigma}^z_{25}\hat{\sigma}^z_{45} + 576\hat{\sigma}^z_{26}\hat{\sigma}^z_{44} + 576\hat{\sigma}^z_{27}\hat{\sigma}^z_{43} + 576\hat{\sigma}^z_{28}\hat{\sigma}^z_{42} + 576\hat{\sigma}^z_{29}\hat{\sigma}^z_{41} +$$
$$576\hat{\sigma}^z_{30}\hat{\sigma}^z_{40} + 1728\hat{\sigma}^z_{31}\hat{\sigma}^z_{39} - 1152\hat{\sigma}^z_{12}\hat{\sigma}^z_{59} - 864\hat{\sigma}^z_{13}\hat{\sigma}^z_{58} - 864\hat{\sigma}^z_{14}\hat{\sigma}^z_{57} - 5272704\hat{\sigma}^z_{16}\hat{\sigma}^z_{55} - 5272704\hat{\sigma}^z_{17}\hat{\sigma}^z_{54} -$$
$$1728\hat{\sigma}^z_{19}\hat{\sigma}^z_{52} + 576\hat{\sigma}^z_{24}\hat{\sigma}^z_{47} + 576\hat{\sigma}^z_{25}\hat{\sigma}^z_{46} + 576\hat{\sigma}^z_{26}\hat{\sigma}^z_{45} + 576\hat{\sigma}^z_{27}\hat{\sigma}^z_{44} - 1152\hat{\sigma}^z_{13}\hat{\sigma}^z_{59} - 1152\hat{\sigma}^z_{14}\hat{\sigma}^z_{58} - 5272704\hat{\sigma}^z_{16}\hat{\sigma}^z_{56} -$$
$$1728\hat{\sigma}^z_{18}\hat{\sigma}^z_{54} - 5272704\hat{\sigma}^z_{19}\hat{\sigma}^z_{53} + 576\hat{\sigma}^z_{21}\hat{\sigma}^z_{51} + 576\hat{\sigma}^z_{24}\hat{\sigma}^z_{48} + 576\hat{\sigma}^z_{25}\hat{\sigma}^z_{47} + 1152\hat{\sigma}^z_{26}\hat{\sigma}^z_{46} + 576\hat{\sigma}^z_{27}\hat{\sigma}^z_{45} + 576\hat{\sigma}^z_{28}\hat{\sigma}^z_{44} +$$

$$576\hat{\sigma}^z_{31}\hat{\sigma}^z_{41} - 864\hat{\sigma}^z_{14}\hat{\sigma}^z_{59} - 5272704\hat{\sigma}^z_{18}\hat{\sigma}^z_{55} - 864\hat{\sigma}^z_{19}\hat{\sigma}^z_{54} + 576\hat{\sigma}^z_{24}\hat{\sigma}^z_{49} + 576\hat{\sigma}^z_{25}\hat{\sigma}^z_{48} + 576\hat{\sigma}^z_{26}\hat{\sigma}^z_{47} + 576\hat{\sigma}^z_{27}\hat{\sigma}^z_{46} +$$
$$576\hat{\sigma}^z_{28}\hat{\sigma}^z_{45} + 576\hat{\sigma}^z_{29}\hat{\sigma}^z_{44} + 1728\hat{\sigma}^z_{34}\hat{\sigma}^z_{39} - 5272704\hat{\sigma}^z_{17}\hat{\sigma}^z_{57} - 1728\hat{\sigma}^z_{19}\hat{\sigma}^z_{55} + 576\hat{\sigma}^z_{20}\hat{\sigma}^z_{54} + 576\hat{\sigma}^z_{22}\hat{\sigma}^z_{52} + 576\hat{\sigma}^z_{24}\hat{\sigma}^z_{50} +$$
$$576\hat{\sigma}^z_{25}\hat{\sigma}^z_{49} + 576\hat{\sigma}^z_{26}\hat{\sigma}^z_{48} + 1152\hat{\sigma}^z_{27}\hat{\sigma}^z_{47} + 576\hat{\sigma}^z_{28}\hat{\sigma}^z_{46} + 576\hat{\sigma}^z_{29}\hat{\sigma}^z_{45} + 576\hat{\sigma}^z_{30}\hat{\sigma}^z_{44} + 576\hat{\sigma}^z_{32}\hat{\sigma}^z_{42} + 576\hat{\sigma}^z_{34}\hat{\sigma}^z_{40} -$$
$$5272704\hat{\sigma}^z_{17}\hat{\sigma}^z_{58} - 5272704\hat{\sigma}^z_{18}\hat{\sigma}^z_{57} - 5272704\hat{\sigma}^z_{19}\hat{\sigma}^z_{56} - 1728\hat{\sigma}^z_{19}\hat{\sigma}^z_{57} + 576\hat{\sigma}^z_{21}\hat{\sigma}^z_{55} + 576\hat{\sigma}^z_{22}\hat{\sigma}^z_{54} + 576\hat{\sigma}^z_{23}\hat{\sigma}^z_{53} +$$
$$576\hat{\sigma}^z_{24}\hat{\sigma}^z_{52} + 576\hat{\sigma}^z_{25}\hat{\sigma}^z_{51} + 576\hat{\sigma}^z_{27}\hat{\sigma}^z_{49} + 1152\hat{\sigma}^z_{28}\hat{\sigma}^z_{48} + 576\hat{\sigma}^z_{29}\hat{\sigma}^z_{47} + 576\hat{\sigma}^z_{31}\hat{\sigma}^z_{45} + 576\hat{\sigma}^z_{32}\hat{\sigma}^z_{44} + 576\hat{\sigma}^z_{33}\hat{\sigma}^z_{43} +$$
$$576\hat{\sigma}^z_{34}\hat{\sigma}^z_{42} + 576\hat{\sigma}^z_{35}\hat{\sigma}^z_{41} - 5272704\hat{\sigma}^z_{18}\hat{\sigma}^z_{59} - 5272704\hat{\sigma}^z_{19}\hat{\sigma}^z_{58} + 576\hat{\sigma}^z_{20}\hat{\sigma}^z_{57} + 576\hat{\sigma}^z_{27}\hat{\sigma}^z_{50} + 576\hat{\sigma}^z_{28}\hat{\sigma}^z_{49} + 576\hat{\sigma}^z_{29}\hat{\sigma}^z_{48} +$$
$$576\hat{\sigma}^z_{30}\hat{\sigma}^z_{47} + 576\hat{\sigma}^z_{37}\hat{\sigma}^z_{40} - 5272704\hat{\sigma}^z_{19}\hat{\sigma}^z_{59} + 576\hat{\sigma}^z_{22}\hat{\sigma}^z_{56} + 576\hat{\sigma}^z_{23}\hat{\sigma}^z_{55} + 1152\hat{\sigma}^z_{24}\hat{\sigma}^z_{54} + 576\hat{\sigma}^z_{25}\hat{\sigma}^z_{53} + 576\hat{\sigma}^z_{26}\hat{\sigma}^z_{52} +$$
$$1152\hat{\sigma}^z_{29}\hat{\sigma}^z_{49} + 576\hat{\sigma}^z_{32}\hat{\sigma}^z_{46} + 576\hat{\sigma}^z_{33}\hat{\sigma}^z_{45} + 1152\hat{\sigma}^z_{34}\hat{\sigma}^z_{44} + 576\hat{\sigma}^z_{35}\hat{\sigma}^z_{43} + 576\hat{\sigma}^z_{36}\hat{\sigma}^z_{42} + 576\hat{\sigma}^z_{20}\hat{\sigma}^z_{59} + 576\hat{\sigma}^z_{21}\hat{\sigma}^z_{58} +$$
$$576\hat{\sigma}^z_{23}\hat{\sigma}^z_{56} + 576\hat{\sigma}^z_{26}\hat{\sigma}^z_{53} + 576\hat{\sigma}^z_{28}\hat{\sigma}^z_{51} + 576\hat{\sigma}^z_{29}\hat{\sigma}^z_{50} + 576\hat{\sigma}^z_{30}\hat{\sigma}^z_{49} + 576\hat{\sigma}^z_{31}\hat{\sigma}^z_{48} + 576\hat{\sigma}^z_{33}\hat{\sigma}^z_{46} + 576\hat{\sigma}^z_{36}\hat{\sigma}^z_{43} +$$
$$576\hat{\sigma}^z_{38}\hat{\sigma}^z_{41} + 576\hat{\sigma}^z_{39}\hat{\sigma}^z_{40} + 576\hat{\sigma}^z_{21}\hat{\sigma}^z_{59} + 576\hat{\sigma}^z_{22}\hat{\sigma}^z_{58} + 576\hat{\sigma}^z_{23}\hat{\sigma}^z_{57} + 576\hat{\sigma}^z_{24}\hat{\sigma}^z_{56} + 1152\hat{\sigma}^z_{25}\hat{\sigma}^z_{55} + 576\hat{\sigma}^z_{26}\hat{\sigma}^z_{54} +$$
$$576\hat{\sigma}^z_{27}\hat{\sigma}^z_{53} + 576\hat{\sigma}^z_{28}\hat{\sigma}^z_{52} + 576\hat{\sigma}^z_{29}\hat{\sigma}^z_{51} + 576\hat{\sigma}^z_{30}\hat{\sigma}^z_{50} + 576\hat{\sigma}^z_{31}\hat{\sigma}^z_{49} + 576\hat{\sigma}^z_{32}\hat{\sigma}^z_{48} + 576\hat{\sigma}^z_{33}\hat{\sigma}^z_{47} + 576\hat{\sigma}^z_{34}\hat{\sigma}^z_{46} +$$
$$1152\hat{\sigma}^z_{35}\hat{\sigma}^z_{45} + 576\hat{\sigma}^z_{36}\hat{\sigma}^z_{44} + 576\hat{\sigma}^z_{37}\hat{\sigma}^z_{43} + 576\hat{\sigma}^z_{38}\hat{\sigma}^z_{42} + 576\hat{\sigma}^z_{39}\hat{\sigma}^z_{41} + 576\hat{\sigma}^z_{24}\hat{\sigma}^z_{57} + 576\hat{\sigma}^z_{25}\hat{\sigma}^z_{56} + 576\hat{\sigma}^z_{26}\hat{\sigma}^z_{55} +$$
$$576\hat{\sigma}^z_{27}\hat{\sigma}^z_{54} + 576\hat{\sigma}^z_{34}\hat{\sigma}^z_{47} + 576\hat{\sigma}^z_{35}\hat{\sigma}^z_{46} + 576\hat{\sigma}^z_{36}\hat{\sigma}^z_{45} + 576\hat{\sigma}^z_{37}\hat{\sigma}^z_{44} + 576\hat{\sigma}^z_{24}\hat{\sigma}^z_{58} + 576\hat{\sigma}^z_{25}\hat{\sigma}^z_{57} + 1152\hat{\sigma}^z_{26}\hat{\sigma}^z_{56} +$$
$$576\hat{\sigma}^z_{27}\hat{\sigma}^z_{55} + 576\hat{\sigma}^z_{28}\hat{\sigma}^z_{54} + 576\hat{\sigma}^z_{31}\hat{\sigma}^z_{51} + 576\hat{\sigma}^z_{34}\hat{\sigma}^z_{48} + 576\hat{\sigma}^z_{35}\hat{\sigma}^z_{47} + 1152\hat{\sigma}^z_{36}\hat{\sigma}^z_{46} + 576\hat{\sigma}^z_{37}\hat{\sigma}^z_{45} + 576\hat{\sigma}^z_{38}\hat{\sigma}^z_{44} +$$
$$576\hat{\sigma}^z_{24}\hat{\sigma}^z_{59} + 576\hat{\sigma}^z_{25}\hat{\sigma}^z_{58} + 576\hat{\sigma}^z_{26}\hat{\sigma}^z_{57} + 576\hat{\sigma}^z_{27}\hat{\sigma}^z_{56} + 576\hat{\sigma}^z_{28}\hat{\sigma}^z_{55} + 576\hat{\sigma}^z_{29}\hat{\sigma}^z_{54} + 576\hat{\sigma}^z_{34}\hat{\sigma}^z_{49} + 576\hat{\sigma}^z_{35}\hat{\sigma}^z_{48} +$$
$$576\hat{\sigma}^z_{36}\hat{\sigma}^z_{47} + 576\hat{\sigma}^z_{37}\hat{\sigma}^z_{46} + 576\hat{\sigma}^z_{38}\hat{\sigma}^z_{45} + 576\hat{\sigma}^z_{39}\hat{\sigma}^z_{44} + 576\hat{\sigma}^z_{25}\hat{\sigma}^z_{59} + 576\hat{\sigma}^z_{26}\hat{\sigma}^z_{58} + 1152\hat{\sigma}^z_{27}\hat{\sigma}^z_{57} + 576\hat{\sigma}^z_{28}\hat{\sigma}^z_{56} +$$
$$576\hat{\sigma}^z_{29}\hat{\sigma}^z_{55} + 576\hat{\sigma}^z_{30}\hat{\sigma}^z_{54} + 576\hat{\sigma}^z_{32}\hat{\sigma}^z_{52} + 576\hat{\sigma}^z_{34}\hat{\sigma}^z_{50} + 576\hat{\sigma}^z_{35}\hat{\sigma}^z_{49} + 576\hat{\sigma}^z_{36}\hat{\sigma}^z_{48} + 1152\hat{\sigma}^z_{37}\hat{\sigma}^z_{47} + 576\hat{\sigma}^z_{38}\hat{\sigma}^z_{46} +$$
$$576\hat{\sigma}^z_{39}\hat{\sigma}^z_{45} + 576\hat{\sigma}^z_{27}\hat{\sigma}^z_{59} + 1152\hat{\sigma}^z_{28}\hat{\sigma}^z_{58} + 576\hat{\sigma}^z_{29}\hat{\sigma}^z_{57} + 576\hat{\sigma}^z_{31}\hat{\sigma}^z_{55} + 576\hat{\sigma}^z_{32}\hat{\sigma}^z_{54} + 576\hat{\sigma}^z_{33}\hat{\sigma}^z_{53} + 576\hat{\sigma}^z_{34}\hat{\sigma}^z_{52} +$$
$$576\hat{\sigma}^z_{35}\hat{\sigma}^z_{51} + 576\hat{\sigma}^z_{37}\hat{\sigma}^z_{49} + 1152\hat{\sigma}^z_{38}\hat{\sigma}^z_{48} + 576\hat{\sigma}^z_{39}\hat{\sigma}^z_{47} + 576\hat{\sigma}^z_{28}\hat{\sigma}^z_{59} + 576\hat{\sigma}^z_{29}\hat{\sigma}^z_{58} + 576\hat{\sigma}^z_{30}\hat{\sigma}^z_{57} + 576\hat{\sigma}^z_{37}\hat{\sigma}^z_{50} +$$
$$576\hat{\sigma}^z_{38}\hat{\sigma}^z_{49} + 576\hat{\sigma}^z_{39}\hat{\sigma}^z_{48} + 1728\hat{\sigma}^z_{40}\hat{\sigma}^z_{47} + 1152\hat{\sigma}^z_{29}\hat{\sigma}^z_{59} + 576\hat{\sigma}^z_{32}\hat{\sigma}^z_{56} + 576\hat{\sigma}^z_{33}\hat{\sigma}^z_{55} + 1152\hat{\sigma}^z_{34}\hat{\sigma}^z_{54} + 576\hat{\sigma}^z_{35}\hat{\sigma}^z_{53} +$$
$$576\hat{\sigma}^z_{36}\hat{\sigma}^z_{52} + 1152\hat{\sigma}^z_{39}\hat{\sigma}^z_{49} + 576\hat{\sigma}^z_{30}\hat{\sigma}^z_{59} + 576\hat{\sigma}^z_{31}\hat{\sigma}^z_{58} + 576\hat{\sigma}^z_{33}\hat{\sigma}^z_{56} + 576\hat{\sigma}^z_{36}\hat{\sigma}^z_{53} + 576\hat{\sigma}^z_{38}\hat{\sigma}^z_{51} + 576\hat{\sigma}^z_{39}\hat{\sigma}^z_{50} +$$
$$1728\hat{\sigma}^z_{40}\hat{\sigma}^z_{49} + 576\hat{\sigma}^z_{31}\hat{\sigma}^z_{59} + 576\hat{\sigma}^z_{32}\hat{\sigma}^z_{58} + 576\hat{\sigma}^z_{33}\hat{\sigma}^z_{57} + 576\hat{\sigma}^z_{34}\hat{\sigma}^z_{56} + 1152\hat{\sigma}^z_{35}\hat{\sigma}^z_{55} + 576\hat{\sigma}^z_{36}\hat{\sigma}^z_{54} + 576\hat{\sigma}^z_{37}\hat{\sigma}^z_{53} +$$
$$576\hat{\sigma}^z_{38}\hat{\sigma}^z_{52} + 576\hat{\sigma}^z_{39}\hat{\sigma}^z_{51} + 576\hat{\sigma}^z_{40}\hat{\sigma}^z_{50} + 1728\hat{\sigma}^z_{41}\hat{\sigma}^z_{49} + 576\hat{\sigma}^z_{34}\hat{\sigma}^z_{57} + 576\hat{\sigma}^z_{35}\hat{\sigma}^z_{56} + 576\hat{\sigma}^z_{36}\hat{\sigma}^z_{55} + 576\hat{\sigma}^z_{37}\hat{\sigma}^z_{54} +$$
$$576\hat{\sigma}^z_{34}\hat{\sigma}^z_{58} + 576\hat{\sigma}^z_{35}\hat{\sigma}^z_{57} + 1152\hat{\sigma}^z_{36}\hat{\sigma}^z_{56} + 576\hat{\sigma}^z_{37}\hat{\sigma}^z_{55} + 576\hat{\sigma}^z_{38}\hat{\sigma}^z_{54} + 576\hat{\sigma}^z_{41}\hat{\sigma}^z_{51} + 576\hat{\sigma}^z_{34}\hat{\sigma}^z_{59} + 576\hat{\sigma}^z_{35}\hat{\sigma}^z_{58} +$$
$$576\hat{\sigma}^z_{36}\hat{\sigma}^z_{57} + 576\hat{\sigma}^z_{37}\hat{\sigma}^z_{56} + 576\hat{\sigma}^z_{38}\hat{\sigma}^z_{55} + 576\hat{\sigma}^z_{39}\hat{\sigma}^z_{54} + 1728\hat{\sigma}^z_{44}\hat{\sigma}^z_{49} + 576\hat{\sigma}^z_{35}\hat{\sigma}^z_{59} + 576\hat{\sigma}^z_{36}\hat{\sigma}^z_{58} + 1152\hat{\sigma}^z_{37}\hat{\sigma}^z_{57} +$$
$$576\hat{\sigma}^z_{38}\hat{\sigma}^z_{56} + 576\hat{\sigma}^z_{39}\hat{\sigma}^z_{55} + 576\hat{\sigma}^z_{40}\hat{\sigma}^z_{54} + 576\hat{\sigma}^z_{42}\hat{\sigma}^z_{52} + 576\hat{\sigma}^z_{44}\hat{\sigma}^z_{50} + 576\hat{\sigma}^z_{37}\hat{\sigma}^z_{59} + 1152\hat{\sigma}^z_{38}\hat{\sigma}^z_{58} + 576\hat{\sigma}^z_{39}\hat{\sigma}^z_{57} +$$
$$576\hat{\sigma}^z_{41}\hat{\sigma}^z_{55} + 576\hat{\sigma}^z_{42}\hat{\sigma}^z_{54} + 576\hat{\sigma}^z_{43}\hat{\sigma}^z_{53} + 576\hat{\sigma}^z_{44}\hat{\sigma}^z_{52} + 576\hat{\sigma}^z_{45}\hat{\sigma}^z_{51} + 576\hat{\sigma}^z_{38}\hat{\sigma}^z_{59} + 576\hat{\sigma}^z_{39}\hat{\sigma}^z_{58} + 576\hat{\sigma}^z_{40}\hat{\sigma}^z_{57} +$$
$$576\hat{\sigma}^z_{47}\hat{\sigma}^z_{50} + 1152\hat{\sigma}^z_{39}\hat{\sigma}^z_{59} + 576\hat{\sigma}^z_{42}\hat{\sigma}^z_{56} + 576\hat{\sigma}^z_{43}\hat{\sigma}^z_{55} + 1152\hat{\sigma}^z_{44}\hat{\sigma}^z_{54} + 576\hat{\sigma}^z_{45}\hat{\sigma}^z_{53} + 576\hat{\sigma}^z_{46}\hat{\sigma}^z_{52} + 576\hat{\sigma}^z_{40}\hat{\sigma}^z_{59} +$$
$$576\hat{\sigma}^z_{41}\hat{\sigma}^z_{58} + 576\hat{\sigma}^z_{43}\hat{\sigma}^z_{56} + 576\hat{\sigma}^z_{46}\hat{\sigma}^z_{53} + 576\hat{\sigma}^z_{48}\hat{\sigma}^z_{51} + 576\hat{\sigma}^z_{49}\hat{\sigma}^z_{50} + 576\hat{\sigma}^z_{41}\hat{\sigma}^z_{59} + 576\hat{\sigma}^z_{42}\hat{\sigma}^z_{58} + 576\hat{\sigma}^z_{43}\hat{\sigma}^z_{57} +$$
$$576\hat{\sigma}^z_{44}\hat{\sigma}^z_{56} + 1152\hat{\sigma}^z_{45}\hat{\sigma}^z_{55} + 576\hat{\sigma}^z_{46}\hat{\sigma}^z_{54} + 576\hat{\sigma}^z_{47}\hat{\sigma}^z_{53} + 576\hat{\sigma}^z_{48}\hat{\sigma}^z_{52} + 576\hat{\sigma}^z_{49}\hat{\sigma}^z_{51} + 576\hat{\sigma}^z_{44}\hat{\sigma}^z_{57} + 576\hat{\sigma}^z_{45}\hat{\sigma}^z_{56} +$$
$$576\hat{\sigma}^z_{46}\hat{\sigma}^z_{55} + 576\hat{\sigma}^z_{47}\hat{\sigma}^z_{54} + 576\hat{\sigma}^z_{44}\hat{\sigma}^z_{58} + 576\hat{\sigma}^z_{45}\hat{\sigma}^z_{57} + 1152\hat{\sigma}^z_{46}\hat{\sigma}^z_{56} + 576\hat{\sigma}^z_{47}\hat{\sigma}^z_{55} + 576\hat{\sigma}^z_{48}\hat{\sigma}^z_{54} + 576\hat{\sigma}^z_{44}\hat{\sigma}^z_{59} +$$
$$576\hat{\sigma}^z_{45}\hat{\sigma}^z_{58} + 576\hat{\sigma}^z_{46}\hat{\sigma}^z_{57} + 576\hat{\sigma}^z_{47}\hat{\sigma}^z_{56} + 576\hat{\sigma}^z_{48}\hat{\sigma}^z_{55} + 576\hat{\sigma}^z_{49}\hat{\sigma}^z_{54} + 576\hat{\sigma}^z_{45}\hat{\sigma}^z_{59} + 576\hat{\sigma}^z_{46}\hat{\sigma}^z_{58} + 1152\hat{\sigma}^z_{47}\hat{\sigma}^z_{57} +$$
$$576\hat{\sigma}^z_{48}\hat{\sigma}^z_{56} + 576\hat{\sigma}^z_{49}\hat{\sigma}^z_{55} + 576\hat{\sigma}^z_{47}\hat{\sigma}^z_{59} + 1152\hat{\sigma}^z_{48}\hat{\sigma}^z_{58} + 576\hat{\sigma}^z_{49}\hat{\sigma}^z_{57} + 576\hat{\sigma}^z_{48}\hat{\sigma}^z_{59} + 576\hat{\sigma}^z_{49}\hat{\sigma}^z_{58} + 1728\hat{\sigma}^z_{50}\hat{\sigma}^z_{57} +$$
$$1152\hat{\sigma}^z_{49}\hat{\sigma}^z_{59} + 1728\hat{\sigma}^z_{50}\hat{\sigma}^z_{59} + 1728\hat{\sigma}^z_{51}\hat{\sigma}^z_{59} + 1728\hat{\sigma}^z_{54}\hat{\sigma}^z_{59} + 316483200$$

Complete Expression in the Turyn Based Method

A complete expression of $E_k(s)$ in 68-order H matrix using Turyn based method.

$$E_k(s) = 2s_0+2s_1-4s_2+4s_3-4s_4+8s_5+8s_6+8s_7+8s_8+8s_{10}+8s_{12}+4s_0s_2+6s_0s_3+4s_1s_2-6s_0s_4-2s_1s_3+4s_0s_5+2s_1s_4-2s_2s_3+4s_0s_6+4s_1s_5+2s_2s_4+4s_0s_7+4s_1s_6-8s_3s_4+4s_0s_8+4s_1s_7+4s_0s_9+4s_1s_8+8s_0s_{10}+4s_1s_9-4s_0s_{11}+8s_2s_9+4s_1s_{11}+8s_5s_7+4s_3s_{10}+16s_5s_8+16s_6s_7-8s_2s_{12}-4s_3s_{11}-4s_4s_{10}+8s_5s_9+8s_6s_8+4s_4s_{11}+8s_6s_{10}+8s_5s_{12}+8s_6s_{11}+8s_7s_{10}+8s_8s_9+8s_7s_{11}+8s_8s_{12}+8s_9s_{11}+8s_{10}s_{12}+4s_0s_1s_2-2s_0s_2s_3+4s_0s_1s_5+2s_0s_2s_4+2s_1s_2s_3+2s_0s_3s_4-2s_1s_2s_4+2s_1s_3s_4+4s_0s_1s_8+8s_2s_3s_4+4s_0s_1s_9+4s_2s_3s_5+4s_0s_5s_6-4s_2s_3s_6-4s_2s_4s_5+8s_0s_5s_7+4s_1s_5s_6-4s_2s_3s_7+4s_2s_4s_6+4s_3s_4s_5-4s_0s_5s_8+4s_0s_6s_7+4s_2s_3s_8+4s_2s_4s_7+8s_2s_5s_6+8s_0s_6s_8+4s_1s_5s_8+4s_1s_6s_7+4s_2s_3s_9-4s_2s_4s_8+4s_0s_7s_8-4s_2s_3s_{10}-4s_2s_4s_9+8s_2s_6s_7+4s_3s_4s_8+4s_3s_5s_7+4s_1s_7s_8+4s_2s_4s_{10}+4s_3s_4s_9-4s_3s_5s_8-4s_4s_5s_7+8s_2s_7s_8+4s_3s_6s_8+4s_4s_5s_8-4s_4s_6s_8+16s_5s_6s_7+4s_0s_9s_{10}+16s_5s_6s_8+8s_0s_9s_{11}+4s_1s_9s_{10}+8s_5s_6s_9+16s_5s_7s_8-4s_0s_9s_{12}+4s_0s_{10}s_{11}+8s_2s_9s_{10}+16s_6s_7s_8+8s_0s_{10}s_{12}+4s_1s_9s_{12}+4s_1s_{10}s_{11}+8s_5s_7s_{10}+8s_6s_7s_9+4s_0s_{11}s_{12}+8s_2s_{10}s_{11}+4s_3s_9s_{11}+4s_1s_{11}s_{12}-4s_3s_9s_{12}-4s_4s_9s_{11}+8s_5s_8s_{11}+8s_5s_9s_{10}+8s_6s_8s_{10}+8s_7s_8s_9+8s_2s_{11}s_{12}+4s_3s_{10}s_{12}+4s_4s_9s_{12}-4s_4s_{10}s_{12}+8s_5s_{10}s_{11}+8s_6s_9s_{11}+8s_6s_9s_{12}+8s_7s_9s_{11}+8s_8s_9s_{10}+8s_5s_{11}s_{12}+8s_6s_{10}s_{12}+8s_7s_9s_{12}+8s_7s_{10}s_{12}+8s_8s_{10}s_{11}+16s_9s_{10}s_{11}+8s_8s_{11}s_{12}+16s_9s_{11}s_{12}+2s_0s_1s_2s_3-2s_0s_1s_2s_4+2s_0s_1s_3s_4+4s_0s_1s_5s_6+4s_0s_1s_6s_7+4s_0s_1s_7s_8+4s_2s_3s_5s_6-4s_2s_3s_5s_7-4s_2s_4s_5s_6+4s_2s_3s_6s_7+4s_2s_4s_5s_7+4s_3s_4s_5s_6-4s_2s_3s_6s_8-4s_2s_4s_6s_7+4s_0s_1s_9s_{10}+4s_2s_3s_7s_8+4s_2s_4s_6s_8+4s_3s_4s_6s_7-4s_2s_4s_7s_8+4s_0s_1s_{10}s_{11}+4s_3s_4s_7s_8+4s_0s_1s_{11}s_{12}+4s_2s_3s_9s_{10}-4s_2s_3s_9s_{11}-4s_2s_4s_9s_{10}+4s_2s_3s_{10}s_{11}+4s_2s_4s_9s_{11}+4s_3s_4s_9s_{10}+16s_5s_6s_7s_8-4s_2s_3s_{10}s_{12}-4s_2s_4s_{10}s_{11}+4s_2s_3s_{11}s_{12}+4s_2s_4s_{10}s_{12}+4s_3s_4s_{10}s_{11}-4s_2s_4s_{11}s_{12}+4s_3s_4s_{11}s_{12}+8s_5s_6s_9s_{10}+8s_5s_6s_{10}s_{11}+8s_5s_7s_9s_{11}+8s_6s_7s_9s_{10}+8s_5s_6s_{11}s_{12}+8s_5s_7s_{10}s_{12}+8s_5s_8s_9s_{12}+8s_6s_7s_{10}s_{11}+8s_6s_8s_9s_{11}+8s_7s_8s_9s_{10}+8s_6s_7s_{11}s_{12}+8s_6s_8s_{10}s_{12}+8s_7s_8s_{10}s_{11}+8s_7s_8s_{11}s_{12}+16s_9s_{10}s_{11}s_{12}+122$$

A complete expression of $E_k(q)$ in 68-order H matrix using Turyn based method.

$$E_k(q) = 168q_0q_1-212q_1-128q_2-132q_3-36q_4-512q_5-576q_6-576q_7-512q_8-496q_9-512q_{10}-496q_{11}-368q_{12}-244q_0+32q_0q_2+40q_0q_3+32q_1q_2-8q_0q_4+24q_1q_3+80q_0q_5+8q_1q_4+64q_2q_3+112q_0q_6+80q_1q_5+112q_0q_7+80q_1q_6+32q_2q_5+168q_3q_4+80q_0q_8+80q_1q_7+64q_2q_6+48q_3q_5+80q_0q_9+80q_1q_8+64q_2q_7+48q_3q_6+16q_4q_5+128q_0q_{10}+80q_1q_9+32q_2q_8+48q_3q_7+16q_4q_6+80q_0q_{11}+64q_1q_{10}+64q_2q_9+48q_3q_8+16q_4q_7+416q_5q_6+48q_0q_{12}+80q_1q_{11}+64q_2q_{10}+48q_3q_9+16q_4q_8+352q_5q_7+48q_1q_{12}+64q_2q_{11}+64q_3q_{10}+16q_4q_9+320q_5q_8+480q_6q_7+48q_3q_{11}+192q_5q_9+352q_6q_8+16q_3q_{12}+16q_4q_{11}+192q_5q_{10}+224q_6q_9+416q_7q_8+16q_4q_{12}+192q_5q_{11}+256q_6q_{10}+224q_7q_9+160q_5q_{12}+224q_6q_{11}+256q_7q_{10}+192q_8q_9+160q_6q_{12}+224q_7q_{11}+192q_8q_{10}+160q_7q_{12}+192q_8q_{11}+384q_9q_{10}+160q_8q_{12}+384q_9q_{11}+224q_9q_{12}+384q_{10}q_{11}+256q_{10}q_{12}+384q_{11}q_{12}-32q_0q_1q_2-32q_0q_1q_3-64q_0q_1q_5-32q_1q_2q_3-64q_0q_1q_6-32q_0q_3q_4+32q_1q_2q_4-64q_0q_1q_7-32q_1q_3q_4-64q_0q_1q_8-64q_2q_3q_4-64q_0q_1q_9-32q_2q_3q_5-64q_0q_1q_{10}-64q_0q_5q_6+32q_2q_4q_5-64q_0q_1q_{11}-64q_0q_5q_7-64q_1q_5q_6-64q_3q_4q_5-32q_0q_1q_{12}+32q_0q_5q_8-64q_0q_6q_7-32q_2q_3q_8-64q_2q_5q_6-64q_3q_4q_6-64q_0q_6q_8-32q_1q_5q_8-64q_1q_6q_7-32q_2q_3q_9+32q_2q_4q_8-64q_3q_4q_7-64q_3q_5q_6-64q_0q_7q_8+32q_2q_4q_9-64q_2q_6q_7-64q_3q_4q_8-64q_1q_7q_8-32q_2q_3q_{11}-64q_3q_4q_9+32q_3q_5q_8-64q_3q_6q_7+32q_2q_4q_{11}-64q_2q_7q_8-64q_3q_4q_{10}-32q_4q_5q_8-64q_3q_4q_{11}-64q_3q_7q_8-256q_5q_6q_7-64q_0q_9q_{10}-32q_3q_4q_{12}-256q_5q_6q_8-64q_0q_9q_{11}-64q_1q_9q_{10}-128q_5q_6q_9-256q_5q_7q_8+32q_0q_9q_{12}-64q_0q_{10}q_{11}-64q_2q_9q_{10}-128q_5q_6q_{10}-64q_5q_7q_9-256q_6q_7q_8-64q_0q_{10}q_{12}-32q_1q_9q_{12}-64q_1q_{10}q_{11}-64q_3q_9q_{10}-128q_5q_6q_{11}-128q_5q_7q_{10}-64q_5q_8q_9-128q_6q_7q_9-64q_0q_{11}q_{12}-64q_2q_{10}q_{11}-64q_5q_6q_{12}-64q_5q_7q_{11}-128q_6q_7q_{10}-64q_6q_8q_9-64q_1q_{11}q_{12}+32q_3q_9q_{12}-64q_3q_{10}q_{11}-64q_5q_7q_{12}-64q_5q_8q_{11}-128q_5q_9q_{10}-128q_6q_7q_{11}-128q_6q_8q_{10}-128q_7q_8q_9-64q_2q_{11}q_{12}-32q_4q_9q_{12}-64q_5q_8q_{12}-64q_5q_9q_{11}-64q_6q_7q_{12}-64q_6q_8q_{11}-128q_6q_9q_{10}-128q_7q_8q_{10}-64q_3q_{11}q_{12}-64q_5q_9q_{12}-128q_5q_{10}q_{11}-64q_6q_8q_{12}-128q_6q_9q_{11}-128q_7q_8q_{11}-128q_7q_9q_{10}-64q_5q_{10}q_{12}-64q_6q_9q_{12}-128q_6q_{10}q_{11}-64q_7q_8q_{12}-128q_7q_9q_{11}-128q_8q_9q_{10}-128q_5q_{11}q_{12}-128q_6q_{10}q_{12}-64q_7q_9q_{12}-128q_7q_{10}q_{11}-64q_8q_9q_{11}-128q_6q_{11}q_{12}-128q_7q_{10}q_{12}-64q_8q_9q_{12}-128q_8q_{10}q_{11}-128q_7q_{11}q_{12}-64q_8q_{10}q_{12}-256q_9q_{10}q_{11}-128q_8q_{11}q_{12}-128q_9q_{10}q_{12}-256q_9q_{11}q_{12}-128q_{10}q_{11}q_{12}+$$

$32q_0q_1q_2q_3 - 32q_0q_1q_2q_4 + 32q_0q_1q_3q_4 + 64q_0q_1q_5q_6 + 64q_0q_1q_6q_7 + 64q_0q_1q_7q_8 + 64q_2q_3q_5q_6 -$
$64q_2q_3q_5q_7 - 64q_2q_4q_5q_6 + 64q_2q_3q_6q_7 + 64q_2q_4q_5q_7 + 64q_3q_4q_5q_6 - 64q_2q_3q_6q_8 - 64q_2q_4q_6q_7 +$
$64q_0q_1q_9q_{10} + 64q_2q_3q_7q_8 + 64q_2q_4q_6q_8 + 64q_3q_4q_6q_7 - 64q_2q_4q_7q_8 + 64q_0q_1q_{10}q_{11} + 64q_3q_4q_7q_8 +$
$64q_0q_1q_{11}q_{12} + 64q_2q_3q_9q_{10} - 64q_2q_3q_9q_{11} - 64q_2q_4q_9q_{10} + 64q_2q_3q_{10}q_{11} + 64q_2q_4q_9q_{11} + 64q_3q_4q_9q_{10} +$
$256q_5q_6q_7q_8 - 64q_2q_3q_{10}q_{12} - 64q_2q_4q_{10}q_{11} + 64q_2q_3q_{11}q_{12} + 64q_2q_4q_{10}q_{12} + 64q_3q_4q_{10}q_{11} - 64q_2q_4q_{11}q_{12} +$
$64q_3q_4q_{11}q_{12} + 128q_5q_6q_9q_{10} + 128q_5q_6q_{10}q_{11} + 128q_5q_7q_9q_{11} + 128q_6q_7q_9q_{10} + 128q_5q_6q_{11}q_{12} + 128q_5q_7q_{10}q_{12} +$
$128q_5q_8q_9q_{12} + 128q_6q_7q_{10}q_{11} + 128q_6q_8q_9q_{11} + 128q_7q_8q_9q_{10} + 128q_6q_7q_{11}q_{12} + 128q_6q_8q_{10}q_{12} +$
$128q_7q_8q_{10}q_{11} + 128q_7q_8q_{11}q_{12} + 256q_9q_{10}q_{11}q_{12} + 948$

A complete expression of $E_2(q)$ in 68-order H matrix using Turyn based method, using $\delta = 61,032$.

$E_2(q) = 183264q_{13} - 212q_1 - 128q_2 - 132q_3 - 36q_4 - 512q_5 - 576q_6 - 576q_7 - 512q_8 - 496q_9 - 512q_{10} -$
$496q_{11} - 368q_{12} - 244q_0 + 183136q_{14} + 183176q_{15} + 183208q_{16} + 183208q_{17} + 183176q_{18} + 183224q_{19} +$
$183176q_{20} + 183128q_{21} + 183120q_{22} + 183176q_{23} + 183176q_{24} + 183176q_{25} + 183176q_{26} + 183160q_{27} +$
$183176q_{28} + 183160q_{29} + 183096q_{30} + 183128q_{31} + 183160q_{32} + 183160q_{33} + 183160q_{34} + 183160q_{35} +$
$183160q_{36} + 183264q_{37} + 183144q_{38} + 183144q_{39} + 183144q_{40} + 183144q_{41} + 183160q_{42} + 183144q_{43} +$
$183112q_{44} + 183112q_{45} + 183512q_{46} + 183448q_{47} + 183416q_{48} + 183288q_{49} + 183288q_{50} + 183288q_{51} +$
$183576q_{52} + 183448q_{53} + 183320q_{54} + 183352q_{55} + 183320q_{56} + 183512q_{57} + 183320q_{58} + 183352q_{59} +$
$183320q_{60} + 183288q_{61} + 183288q_{62} + 183288q_{63} + 183480q_{64} + 183480q_{65} + 183320q_{66} + 183480q_{67} +$
$183352q_{68} + 183480q_{69} + 61032q_0q_1 + 32q_0q_2 + 61032q_0q_3 + 61032q_1q_2 - 8q_0q_4 + 61032q_1q_3 + 61032q_0q_5 +$
$8q_1q_4 + 61032q_2q_3 + 61032q_0q_6 + 61032q_1q_5 + 61032q_2q_4 + 61032q_0q_7 + 61032q_1q_6 + 61032q_2q_5 + 61032q_3q_4 +$
$80q_0q_8 + 61032q_1q_7 + 61032q_2q_6 + 61032q_3q_5 + 61032q_0q_9 + 80q_1q_8 + 61032q_2q_7 + 61032q_3q_6 + 61032q_4q_5 +$
$61032q_0q_{10} + 61032q_1q_9 + 32q_2q_8 + 61032q_3q_7 + 16q_4q_6 + 61032q_0q_{11} + 61032q_1q_{10} + 61032q_2q_9 + 48q_3q_8 +$
$16q_4q_7 + 61032q_5q_6 + 48q_0q_{12} + 61032q_1q_{11} + 61032q_2q_{10} + 61032q_3q_9 + 16q_4q_8 + 61032q_5q_7 - 122064q_0q_{13} +$
$48q_1q_{12} + 61032q_2q_{11} + 61032q_3q_{10} + 61032q_4q_9 + 61032q_5q_8 + 61032q_6q_7 - 122064q_0q_{14} - 122064q_1q_{13} +$
$61032q_3q_{11} + 61032q_5q_9 + 61032q_6q_8 - 122064q_0q_{15} - 32q_2q_{13} + 16q_3q_{12} + 16q_4q_{11} + 61032q_5q_{10} +$
$61032q_6q_9 + 61032q_7q_8 - 122064q_0q_{16} - 32q_3q_{13} + 16q_4q_{12} + 61032q_5q_{11} + 61032q_6q_{10} + 61032q_7q_9 -$
$122064q_0q_{17} - 122064q_3q_{14} + 160q_5q_{12} + 61032q_6q_{11} + 61032q_7q_{10} + 61032q_8q_9 - 122064q_0q_{18} - 32q_4q_{14} -$
$64q_5q_{13} + 160q_6q_{12} + 61032q_7q_{11} + 61032q_8q_{10} - 122064q_0q_{19} - 64q_6q_{13} + 160q_7q_{12} + 61032q_8q_{11} +$
$61032q_9q_{10} - 122064q_0q_{20} - 122064q_5q_{15} - 64q_7q_{13} + 160q_8q_{12} + 61032q_9q_{11} - 64q_6q_{15} - 64q_8q_{13} +$
$61032q_9q_{12} + 61032q_{10}q_{11} - 122064q_1q_{21} - 122064q_6q_{16} - 64q_7q_{15} - 64q_9q_{13} + 61032q_{10}q_{12} - 122064q_1q_{22} -$
$122064q_2q_{21} - 64q_7q_{16} + 32q_8q_{15} - 64q_{10}q_{13} + 61032q_{11}q_{12} - 122064q_1q_{23} - 32q_3q_{21} - 122064q_7q_{17} -$
$64q_8q_{16} - 64q_{11}q_{13} - 122064q_1q_{24} - 122064q_3q_{22} + 32q_4q_{21} - 64q_8q_{17} - 32q_{12}q_{13} - 122064q_1q_{25} -$
$32q_4q_{22} - 122064q_1q_{26} - 122064q_9q_{18} - 122064q_1q_{27} - 122064q_5q_{23} - 64q_{10}q_{18} - 122064q_1q_{28} - 64q_6q_{23} -$
$122064q_{10}q_{19} - 64q_{11}q_{18} - 122064q_6q_{24} - 64q_{11}q_{19} + 32q_{12}q_{18} - 122064q_2q_{29} - 64q_7q_{24} - 32q_8q_{23} -$
$122064q_{11}q_{20} - 64q_{12}q_{19} - 122064q_2q_{30} - 122064q_3q_{29} - 122064q_7q_{25} - 64q_{12}q_{20} - 122064q_2q_{31} -$
$64q_4q_{29} - 64q_8q_{25} - 122064q_2q_{32} - 122064q_4q_{30} - 32q_5q_{29} - 122064q_2q_{33} + 32q_5q_{30} - 122064q_9q_{26} -$
$122064q_2q_{34} - 122064q_5q_{31} - 64q_{10}q_{26} - 122064q_2q_{35} - 64q_6q_{31} - 32q_8q_{29} - 122064q_{10}q_{27} - 122064q_2q_{36} -$
$122064q_6q_{32} + 32q_8q_{30} - 32q_9q_{29} - 64q_{11}q_{27} - 32q_{12}q_{26} - 64q_7q_{32} + 32q_9q_{30} - 122064q_{11}q_{28} - 122064q_3q_{37} -$
$122064q_7q_{33} - 32q_{11}q_{29} - 64q_{12}q_{28} - 122064q_3q_{38} - 122064q_4q_{37} - 64q_8q_{33} + 32q_{11}q_{30} - 122064q_3q_{39} -$
$64q_5q_{37} + 32q_{13}q_{29} - 122064q_3q_{40} - 122064q_5q_{38} - 64q_6q_{37} - 122064q_9q_{34} - 32q_{13}q_{30} - 122064q_3q_{41} -$
$64q_6q_{38} - 64q_7q_{37} - 64q_{10}q_{34} - 122064q_3q_{42} - 122064q_6q_{39} - 64q_8q_{37} - 122064q_{10}q_{35} - 122064q_3q_{43} -$
$64q_7q_{39} + 32q_8q_{38} - 64q_9q_{37} - 64q_{11}q_{35} - 122064q_7q_{40} - 64q_{10}q_{37} - 122064q_{11}q_{36} - 122064q_4q_{44} - 64q_8q_{40} -$
$64q_{11}q_{37} - 64q_{12}q_{36} - 122064q_4q_{45} - 122064q_5q_{44} - 32q_{12}q_{37} - 122064q_9q_{41} + 32q_{13}q_{37} - 122064q_5q_{46} -$
$64q_{10}q_{41} - 122064q_5q_{47} - 122064q_6q_{46} - 32q_8q_{44} - 122064q_{10}q_{42} - 122064q_5q_{48} - 256q_7q_{46} - 64q_{11}q_{42} +$
$32q_{12}q_{41} - 122064q_5q_{49} - 122064q_7q_{47} - 256q_8q_{46} - 122064q_9q_{45} - 122064q_{11}q_{43} - 122064q_5q_{50} -$
$256q_8q_{47} - 128q_9q_{46} - 64q_{12}q_{43} - 122064q_5q_{51} - 122064q_8q_{48} - 64q_9q_{47} - 128q_{10}q_{46} - 64q_9q_{48} -$
$128q_{10}q_{47} - 128q_{11}q_{46} - 32q_{12}q_{45} - 122064q_6q_{52} - 122064q_9q_{49} - 64q_{11}q_{47} - 64q_{12}q_{46} - 122064q_6q_{53} -$

$122064q_7q_{52} - 128q_{10}q_{49} - 64q_{11}q_{48} - 64q_{12}q_{47} + 64q_{13}q_{46} - 122064q_6q_{54} - 256q_8q_{52} - 122064q_{10}q_{50} - 64q_{11}q_{49} - 64q_{12}q_{48} - 122064q_6q_{55} - 122064q_8q_{53} - 128q_9q_{52} - 128q_{11}q_{50} - 64q_{12}q_{49} - 122064q_6q_{56} - 64q_9q_{53} - 128q_{10}q_{52} - 122064q_{11}q_{51} - 64q_{12}q_{50} - 122064q_9q_{54} - 128q_{10}q_{53} - 128q_{11}q_{52} - 128q_{12}q_{51} - 122064q_7q_{57} - 128q_{10}q_{54} - 64q_{11}q_{53} - 64q_{12}q_{52} - 122064q_7q_{58} - 122064q_8q_{57} - 122064q_{10}q_{55} - 128q_{11}q_{54} - 64q_{12}q_{53} + 64q_{13}q_{52} - 122064q_7q_{59} - 128q_9q_{57} - 128q_{11}q_{55} - 64q_{12}q_{54} - 122064q_7q_{60} - 122064q_9q_{58} - 128q_{10}q_{57} - 122064q_{11}q_{56} - 128q_{12}q_{55} - 128q_{10}q_{58} - 128q_{11}q_{57} - 128q_{12}q_{56} - 122064q_8q_{61} - 122064q_{10}q_{59} - 128q_{11}q_{58} - 64q_{12}q_{57} - 122064q_8q_{62} - 122064q_9q_{61} - 128q_{11}q_{59} - 64q_{12}q_{58} + 64q_{13}q_{57} - 122064q_8q_{63} - 128q_{10}q_{61} - 122064q_{11}q_{60} - 128q_{12}q_{59} - 122064q_{10}q_{62} - 64q_{11}q_{61} - 128q_{12}q_{60} - 122064q_9q_{64} - 128q_{11}q_{62} - 64q_{12}q_{61} - 122064q_9q_{65} - 122064q_{10}q_{64} - 122064q_{11}q_{63} - 64q_{12}q_{62} - 122064q_9q_{66} - 256q_{11}q_{64} - 128q_{12}q_{63} + 64q_{29}q_{46} - 122064q_{11}q_{65} - 128q_{12}q_{64} - 64q_{29}q_{47} - 64q_{30}q_{46} - 122064q_{10}q_{67} - 256q_{12}q_{65} + 64q_{13}q_{64} + 64q_{30}q_{47} - 122064q_{10}q_{68} - 122064q_{11}q_{67} - 122064q_{12}q_{66} - 128q_{12}q_{67} - 122064q_{11}q_{69} - 122064q_{12}q_{68} + 64q_{13}q_{67} - 122064q_{12}q_{69} + 64q_{29}q_{52} + 64q_{13}q_{69} - 64q_{29}q_{53} - 64q_{30}q_{52} + 64q_{30}q_{53} + 64q_{37}q_{46} + 64q_{29}q_{57} - 64q_{30}q_{57} + 64q_{37}q_{52} + 64q_{29}q_{64} - 64q_{29}q_{65} - 64q_{30}q_{64} + 64q_{37}q_{57} + 64q_{30}q_{65} + 64q_{29}q_{67} - 64q_{29}q_{68} - 64q_{30}q_{67} + 64q_{29}q_{69} + 64q_{30}q_{68} - 64q_{30}q_{69} + 64q_{37}q_{64} + 256q_{46}q_{57} + 64q_{37}q_{67} + 64q_{37}q_{69} + 128q_{46}q_{64} + 128q_{47}q_{65} + 128q_{46}q_{67} + 128q_{48}q_{66} + 128q_{46}q_{69} + 128q_{47}q_{68} + 128q_{52}q_{64} + 128q_{53}q_{65} + 128q_{52}q_{67} + 128q_{52}q_{69} + 128q_{53}q_{68} + 128q_{57}q_{64} + 128q_{57}q_{67} + 128q_{57}q_{69} + 256q_{64}q_{69} + 948$

A complete expression of $E_2(s)$ in 68-order H matrix using Turyn based method, using $\delta = 61,032$.

$E_2(s) = 122148s_0 + 137394s_1 + 137378s_2 + 152646s_3 + 61054s_4 + 168086s_5 + 152920s_6 + 153000s_7 + 92004s_8 + 183520s_9 + 168478s_{10} + 168626s_{11} + 46374s_{12} - 30568s_{13} - 30528s_{14} - 30532s_{15} - 30540s_{16} - 30556s_{17} - 30532s_{18} - 30548s_{19} - 30540s_{20} - 30532s_{21} - 30520s_{22} - 30532s_{23} - 30540s_{24} - 30540s_{25} - 30532s_{26} - 30532s_{27} - 30540s_{28} - 30540s_{29} - 30508s_{30} - 30516s_{31} - 30532s_{32} - 30532s_{33} - 30532s_{34} - 30532s_{35} - 30532s_{36} - 30584s_{37} - 30532s_{38} - 30524s_{39} - 30524s_{40} - 30532s_{41} - 30532s_{42} - 30524s_{43} - 30516s_{44} - 30516s_{45} - 30676s_{46} - 30612s_{47} - 30660s_{48} - 30548s_{49} - 30564s_{50} - 30580s_{51} - 30708s_{52} - 30676s_{53} - 30548s_{54} - 30580s_{55} - 30596s_{56} - 30804s_{57} - 30548s_{58} - 30580s_{59} - 30596s_{60} - 30548s_{61} - 30564s_{62} - 30580s_{63} - 30804s_{64} - 30708s_{65} - 30660s_{66} - 30804s_{67} - 30708s_{68} - 30900s_{69} + 15258s_0s_1 + 8s_0s_2 + 15258s_0s_3 + 15258s_1s_2 - 2s_0s_4 + 15258s_1s_3 + 15258s_0s_5 + 2s_1s_4 + 15258s_2s_3 + 15258s_0s_6 + 15258s_1s_5 + 15258s_2s_4 + 15258s_0s_7 + 15258s_1s_6 + 15258s_2s_5 + 15258s_3s_4 + 20s_0s_8 + 15258s_1s_7 + 15258s_2s_6 + 15258s_3s_5 + 15258s_0s_9 + 20s_1s_8 + 15258s_2s_7 + 15258s_3s_6 + 15258s_4s_5 + 15258s_0s_{10} + 15258s_1s_9 + 8s_2s_8 + 15258s_3s_7 + 4s_4s_6 + 15258s_0s_{11} + 15258s_1s_{10} + 15258s_2s_9 + 12s_3s_8 + 4s_4s_7 + 15258s_5s_6 + 12s_0s_{12} + 15258s_1s_{11} + 15258s_2s_{10} + 15258s_3s_9 + 4s_4s_8 + 15258s_5s_7 - 30516s_0s_{13} + 12s_1s_{12} + 15258s_2s_{11} + 15258s_3s_{10} + 15258s_4s_9 + 15258s_5s_8 + 15258s_6s_7 - 30516s_0s_{14} - 30516s_1s_{13} + 15258s_3s_{11} + 15258s_5s_9 + 15258s_6s_8 - 30516s_0s_{15} - 8s_2s_{13} + 4s_3s_{12} + 4s_4s_{11} + 15258s_5s_{10} + 15258s_6s_9 + 15258s_7s_8 - 30516s_0s_{16} - 8s_3s_{13} + 4s_4s_{12} + 15258s_5s_{11} + 15258s_6s_{10} + 15258s_7s_9 - 30516s_0s_{17} - 30516s_3s_{14} + 40s_5s_{12} + 15258s_6s_{11} + 15258s_7s_{10} + 15258s_8s_9 - 30516s_0s_{18} - 8s_4s_{14} - 16s_5s_{13} + 40s_6s_{12} + 15258s_7s_{11} + 15258s_8s_{10} - 30516s_0s_{19} - 16s_6s_{13} + 40s_7s_{12} + 15258s_8s_{11} + 15258s_9s_{10} - 30516s_0s_{20} - 30516s_5s_{15} - 16s_7s_{13} + 40s_8s_{12} + 15258s_9s_{11} - 16s_6s_{15} - 16s_8s_{13} + 15258s_9s_{12} + 15258s_{10}s_{11} - 30516s_1s_{21} - 30516s_6s_{16} - 16s_7s_{15} - 16s_9s_{13} + 15258s_{10}s_{12} - 30516s_1s_{22} - 30516s_2s_{21} - 16s_7s_{16} + 8s_8s_{15} - 16s_{10}s_{13} + 15258s_{11}s_{12} - 30516s_1s_{23} - 8s_3s_{21} - 30516s_7s_{17} - 16s_8s_{16} - 16s_{11}s_{13} - 30516s_1s_{24} - 30516s_3s_{22} + 8s_4s_{21} - 16s_8s_{17} - 8s_{12}s_{13} - 30516s_1s_{25} - 8s_4s_{22} - 30516s_1s_{26} - 30516s_9s_{18} - 30516s_1s_{27} - 30516s_5s_{23} - 16s_{10}s_{18} - 30516s_1s_{28} - 16s_6s_{23} - 30516s_{10}s_{19} - 16s_{11}s_{18} - 30516s_6s_{24} - 16s_{11}s_{19} + 8s_{12}s_{18} - 30516s_2s_{29} - 16s_7s_{24} - 8s_8s_{23} - 30516s_{11}s_{20} - 16s_{12}s_{19} - 30516s_2s_{30} - 30516s_3s_{29} - 30516s_7s_{25} - 16s_{12}s_{20} - 30516s_2s_{31} - 16s_4s_{29} - 16s_8s_{25} - 30516s_2s_{32} - 30516s_4s_{30} - 8s_5s_{29} - 30516s_2s_{33} + 8s_5s_{30} - 30516s_9s_{26} - 30516s_2s_{34} - 30516s_5s_{31} - 16s_{10}s_{26} - 30516s_2s_{35} - 16s_6s_{31} - 8s_8s_{29} - 30516s_{10}s_{27} - 30516s_2s_{36} - 30516s_6s_{32} + 8s_8s_{30} - 8s_9s_{29} - 16s_{11}s_{27} - 8s_{12}s_{26} - 16s_7s_{32} + 8s_9s_{30} - 30516s_{11}s_{28} - 30516s_3s_{37} - 30516s_7s_{33} - 8s_{11}s_{29} - 16s_{12}s_{28} - 30516s_3s_{38} - 30516s_4s_{37} - 16s_8s_{33} + 8s_{11}s_{30} - 30516s_3s_{39} - 16s_5s_{37} + 8s_{13}s_{29} - 30516s_3s_{40} - 30516s_5s_{38} - 16s_6s_{37} - 30516s_9s_{34} - 8s_{13}s_{30} - 30516s_3s_{41} - 16s_6s_{38} - 16s_7s_{37} - 16s_{10}s_{34} - 30516s_3s_{42} - 30516s_6s_{39} - 16s_8s_{37} - 30516s_{10}s_{35} - 30516s_3s_{43} - 16s_7s_{39} + 8s_8s_{38} - 16s_9s_{37} - 16s_{11}s_{35} - 30516s_7s_{40} - 16s_{10}s_{37} - 30516s_{11}s_{36} - 30516s_4s_{44} - 16s_8s_{40} - 16s_{11}s_{37} -$

$16s_{12}s_{36} - 30516s_{4}s_{45} - 30516s_{5}s_{44} - 8s_{12}s_{37} - 30516s_{9}s_{41} + 8s_{13}s_{37} - 30516s_{5}s_{46} - 16s_{10}s_{41} - 30516s_{5}s_{47} -$
$30516s_{6}s_{46} - 8s_{8}s_{44} - 30516s_{10}s_{42} - 30516s_{5}s_{48} - 64s_{7}s_{46} - 16s_{11}s_{42} + 8s_{12}s_{41} - 30516s_{5}s_{49} - 30516s_{7}s_{47} -$
$64s_{8}s_{46} - 30516s_{9}s_{45} - 30516s_{11}s_{43} - 30516s_{5}s_{50} - 64s_{8}s_{47} - 32s_{9}s_{46} - 16s_{12}s_{43} - 30516s_{5}s_{51} - 30516s_{8}s_{48} -$
$16s_{9}s_{47} - 32s_{10}s_{46} - 16s_{9}s_{48} - 32s_{10}s_{47} - 32s_{11}s_{46} - 8s_{12}s_{45} - 30516s_{6}s_{52} - 30516s_{9}s_{49} - 16s_{11}s_{47} - 16s_{12}s_{46} -$
$30516s_{6}s_{53} - 30516s_{7}s_{52} - 32s_{10}s_{49} - 16s_{11}s_{48} - 16s_{12}s_{47} + 16s_{13}s_{46} - 30516s_{6}s_{54} - 64s_{8}s_{52} - 30516s_{10}s_{50} -$
$16s_{11}s_{49} - 16s_{12}s_{48} - 30516s_{6}s_{55} - 30516s_{8}s_{53} - 32s_{9}s_{52} - 32s_{11}s_{50} - 16s_{12}s_{49} - 30516s_{6}s_{56} - 16s_{9}s_{53} - 32s_{10}s_{52} -$
$30516s_{11}s_{51} - 16s_{12}s_{50} - 30516s_{9}s_{54} - 32s_{10}s_{53} - 32s_{11}s_{52} - 32s_{12}s_{51} - 30516s_{7}s_{57} - 32s_{10}s_{54} - 16s_{11}s_{53} -$
$16s_{12}s_{52} - 30516s_{7}s_{58} - 30516s_{8}s_{57} - 30516s_{10}s_{55} - 32s_{11}s_{54} - 16s_{12}s_{53} + 16s_{13}s_{52} - 30516s_{7}s_{59} - 32s_{9}s_{57} -$
$32s_{11}s_{55} - 16s_{12}s_{54} - 30516s_{7}s_{60} - 30516s_{9}s_{58} - 32s_{10}s_{57} - 30516s_{11}s_{56} - 32s_{12}s_{55} - 32s_{10}s_{58} - 32s_{11}s_{57} -$
$32s_{12}s_{56} - 30516s_{8}s_{61} - 30516s_{10}s_{59} - 32s_{11}s_{58} - 16s_{12}s_{57} - 30516s_{8}s_{62} - 30516s_{9}s_{61} - 32s_{11}s_{59} - 16s_{12}s_{58} +$
$16s_{13}s_{57} - 30516s_{8}s_{63} - 32s_{10}s_{61} - 30516s_{11}s_{60} - 32s_{12}s_{59} - 30516s_{10}s_{62} - 16s_{11}s_{61} - 32s_{12}s_{60} - 30516s_{9}s_{64} -$
$32s_{11}s_{62} - 16s_{12}s_{61} - 30516s_{9}s_{65} - 30516s_{10}s_{64} - 30516s_{11}s_{63} - 16s_{12}s_{62} - 30516s_{9}s_{66} - 64s_{11}s_{64} - 32s_{12}s_{63} +$
$16s_{29}s_{46} - 30516s_{11}s_{65} - 32s_{12}s_{64} - 16s_{29}s_{47} - 16s_{30}s_{46} - 30516s_{10}s_{67} - 64s_{12}s_{65} + 16s_{13}s_{64} + 16s_{30}s_{47} -$
$30516s_{10}s_{68} - 30516s_{11}s_{67} - 30516s_{12}s_{66} - 32s_{12}s_{67} - 30516s_{11}s_{69} - 30516s_{12}s_{68} + 16s_{13}s_{67} - 30516s_{12}s_{69} +$
$16s_{29}s_{52} + 16s_{13}s_{69} - 16s_{29}s_{53} - 16s_{30}s_{52} + 16s_{30}s_{53} + 16s_{37}s_{46} + 16s_{29}s_{57} - 16s_{30}s_{57} + 16s_{37}s_{52} + 16s_{29}s_{64} -$
$16s_{29}s_{65} - 16s_{30}s_{64} + 16s_{37}s_{57} + 16s_{30}s_{65} + 16s_{29}s_{67} - 16s_{29}s_{68} - 16s_{30}s_{67} + 16s_{29}s_{69} + 16s_{30}s_{68} - 16s_{30}s_{69} +$
$16s_{37}s_{64} + 64s_{46}s_{57} + 16s_{37}s_{67} + 16s_{37}s_{69} + 32s_{46}s_{64} + 32s_{47}s_{65} + 32s_{46}s_{67} + 32s_{48}s_{66} + 32s_{46}s_{69} + 32s_{47}s_{68} +$
$32s_{52}s_{64} + 32s_{53}s_{65} + 32s_{52}s_{67} + 32s_{52}s_{69} + 32s_{53}s_{68} + 32s_{57}s_{64} + 32s_{57}s_{67} + 32s_{57}s_{69} + 64s_{64}s_{69} + 2610926$

A complete expression of $\hat{H}_2\left(\hat{\sigma}^z\right)$ in 68-order H matrix using Turyn based method, using $\delta = 61,032$.

$\hat{H}_2\left(\hat{\sigma}^z\right) = 122148\hat{\sigma}^z_{0} + 137394\hat{\sigma}^z_{1} + 137378\hat{\sigma}^z_{2} + 152646\hat{\sigma}^z_{3} + 61054\hat{\sigma}^z_{4} + 168086\hat{\sigma}^z_{5} + 152920\hat{\sigma}^z_{6} + 153000\hat{\sigma}^z_{7} +$
$92004\hat{\sigma}^z_{8} + 183520\hat{\sigma}^z_{9} + 168478\hat{\sigma}^z_{10} + 168626\hat{\sigma}^z_{11} + 46374\hat{\sigma}^z_{12} - 30568\hat{\sigma}^z_{13} - 30528\hat{\sigma}^z_{14} - 30532\hat{\sigma}^z_{15} - 30540\hat{\sigma}^z_{16} -$
$30556\hat{\sigma}^z_{17} - 30532\hat{\sigma}^z_{18} - 30548\hat{\sigma}^z_{19} - 30540\hat{\sigma}^z_{20} - 30532\hat{\sigma}^z_{21} - 30520\hat{\sigma}^z_{22} - 30532\hat{\sigma}^z_{23} - 30540\hat{\sigma}^z_{24} - 30540\hat{\sigma}^z_{25} -$
$30532\hat{\sigma}^z_{26} - 30532\hat{\sigma}^z_{27} - 30540\hat{\sigma}^z_{28} - 30540\hat{\sigma}^z_{29} - 30508\hat{\sigma}^z_{30} - 30516\hat{\sigma}^z_{31} - 30532\hat{\sigma}^z_{32} - 30532\hat{\sigma}^z_{33} - 30532\hat{\sigma}^z_{34} -$
$30532\hat{\sigma}^z_{35} - 30532\hat{\sigma}^z_{36} - 30584\hat{\sigma}^z_{37} - 30532\hat{\sigma}^z_{38} - 30524\hat{\sigma}^z_{39} - 30524\hat{\sigma}^z_{40} - 30532\hat{\sigma}^z_{41} - 30532\hat{\sigma}^z_{42} - 30524\hat{\sigma}^z_{43} -$
$30516\hat{\sigma}^z_{44} - 30516\hat{\sigma}^z_{45} - 30676\hat{\sigma}^z_{46} - 30612\hat{\sigma}^z_{47} - 30660\hat{\sigma}^z_{48} - 30548\hat{\sigma}^z_{49} - 30564\hat{\sigma}^z_{50} - 30580\hat{\sigma}^z_{51} - 30708\hat{\sigma}^z_{52} -$
$30676\hat{\sigma}^z_{53} - 30548\hat{\sigma}^z_{54} - 30580\hat{\sigma}^z_{55} - 30596\hat{\sigma}^z_{56} - 30804\hat{\sigma}^z_{57} - 30548\hat{\sigma}^z_{58} - 30580\hat{\sigma}^z_{59} - 30596\hat{\sigma}^z_{60} - 30548\hat{\sigma}^z_{61} -$
$30564\hat{\sigma}^z_{62} - 30580\hat{\sigma}^z_{63} - 30804\hat{\sigma}^z_{64} - 30708\hat{\sigma}^z_{65} - 30660\hat{\sigma}^z_{66} - 30804\hat{\sigma}^z_{67} - 30708\hat{\sigma}^z_{68} - 30900\hat{\sigma}^z_{69} + 15258\hat{\sigma}^z_{0}\hat{\sigma}^z_{1} +$
$8\hat{\sigma}^z_{0}\hat{\sigma}^z_{2} + 15258\hat{\sigma}^z_{0}\hat{\sigma}^z_{3} + 15258\hat{\sigma}^z_{1}\hat{\sigma}^z_{2} - 2\hat{\sigma}^z_{0}\hat{\sigma}^z_{4} + 15258\hat{\sigma}^z_{1}\hat{\sigma}^z_{3} + 15258\hat{\sigma}^z_{0}\hat{\sigma}^z_{5} + 2\hat{\sigma}^z_{1}\hat{\sigma}^z_{4} + 15258\hat{\sigma}^z_{2}\hat{\sigma}^z_{3} + 15258\hat{\sigma}^z_{0}\hat{\sigma}^z_{6} +$
$15258\hat{\sigma}^z_{1}\hat{\sigma}^z_{5} + 15258\hat{\sigma}^z_{2}\hat{\sigma}^z_{4} + 15258\hat{\sigma}^z_{0}\hat{\sigma}^z_{7} + 15258\hat{\sigma}^z_{1}\hat{\sigma}^z_{6} + 15258\hat{\sigma}^z_{2}\hat{\sigma}^z_{5} + 15258\hat{\sigma}^z_{3}\hat{\sigma}^z_{4} + 20\hat{\sigma}^z_{0}\hat{\sigma}^z_{8} + 15258\hat{\sigma}^z_{1}\hat{\sigma}^z_{7} +$
$15258\hat{\sigma}^z_{2}\hat{\sigma}^z_{6} + 15258\hat{\sigma}^z_{3}\hat{\sigma}^z_{5} + 15258\hat{\sigma}^z_{0}\hat{\sigma}^z_{9} + 20\hat{\sigma}^z_{1}\hat{\sigma}^z_{8} + 15258\hat{\sigma}^z_{2}\hat{\sigma}^z_{7} + 15258\hat{\sigma}^z_{3}\hat{\sigma}^z_{6} + 15258\hat{\sigma}^z_{4}\hat{\sigma}^z_{5} + 15258\hat{\sigma}^z_{0}\hat{\sigma}^z_{10} +$
$15258\hat{\sigma}^z_{1}\hat{\sigma}^z_{9} + 8\hat{\sigma}^z_{2}\hat{\sigma}^z_{8} + 15258\hat{\sigma}^z_{3}\hat{\sigma}^z_{7} + 4\hat{\sigma}^z_{4}\hat{\sigma}^z_{6} + 15258\hat{\sigma}^z_{0}\hat{\sigma}^z_{11} + 15258\hat{\sigma}^z_{1}\hat{\sigma}^z_{10} + 15258\hat{\sigma}^z_{2}\hat{\sigma}^z_{9} + 12\hat{\sigma}^z_{3}\hat{\sigma}^z_{8} + 4\hat{\sigma}^z_{4}\hat{\sigma}^z_{7} +$
$15258\hat{\sigma}^z_{5}\hat{\sigma}^z_{6} + 12\hat{\sigma}^z_{0}\hat{\sigma}^z_{12} + 15258\hat{\sigma}^z_{1}\hat{\sigma}^z_{11} + 15258\hat{\sigma}^z_{2}\hat{\sigma}^z_{10} + 15258\hat{\sigma}^z_{3}\hat{\sigma}^z_{9} + 4\hat{\sigma}^z_{4}\hat{\sigma}^z_{8} + 15258\hat{\sigma}^z_{5}\hat{\sigma}^z_{7} - 30516\hat{\sigma}^z_{0}\hat{\sigma}^z_{13} +$
$12\hat{\sigma}^z_{1}\hat{\sigma}^z_{12} + 15258\hat{\sigma}^z_{2}\hat{\sigma}^z_{11} + 15258\hat{\sigma}^z_{3}\hat{\sigma}^z_{10} + 15258\hat{\sigma}^z_{4}\hat{\sigma}^z_{9} + 15258\hat{\sigma}^z_{5}\hat{\sigma}^z_{8} + 15258\hat{\sigma}^z_{6}\hat{\sigma}^z_{7} - 30516\hat{\sigma}^z_{0}\hat{\sigma}^z_{14} - 30516\hat{\sigma}^z_{1}\hat{\sigma}^z_{13} +$
$15258\hat{\sigma}^z_{3}\hat{\sigma}^z_{11} + 15258\hat{\sigma}^z_{5}\hat{\sigma}^z_{9} + 15258\hat{\sigma}^z_{6}\hat{\sigma}^z_{8} - 30516\hat{\sigma}^z_{0}\hat{\sigma}^z_{15} - 8\hat{\sigma}^z_{2}\hat{\sigma}^z_{13} + 4\hat{\sigma}^z_{3}\hat{\sigma}^z_{12} + 4\hat{\sigma}^z_{4}\hat{\sigma}^z_{11} + 15258\hat{\sigma}^z_{5}\hat{\sigma}^z_{10} +$
$15258\hat{\sigma}^z_{6}\hat{\sigma}^z_{9} + 15258\hat{\sigma}^z_{7}\hat{\sigma}^z_{8} - 30516\hat{\sigma}^z_{0}\hat{\sigma}^z_{16} - 8\hat{\sigma}^z_{3}\hat{\sigma}^z_{13} + 4\hat{\sigma}^z_{4}\hat{\sigma}^z_{12} + 15258\hat{\sigma}^z_{5}\hat{\sigma}^z_{11} + 15258\hat{\sigma}^z_{6}\hat{\sigma}^z_{10} + 15258\hat{\sigma}^z_{7}\hat{\sigma}^z_{9} -$
$30516\hat{\sigma}^z_{0}\hat{\sigma}^z_{17} - 30516\hat{\sigma}^z_{3}\hat{\sigma}^z_{14} + 40\hat{\sigma}^z_{5}\hat{\sigma}^z_{12} + 15258\hat{\sigma}^z_{6}\hat{\sigma}^z_{11} + 15258\hat{\sigma}^z_{7}\hat{\sigma}^z_{10} + 15258\hat{\sigma}^z_{8}\hat{\sigma}^z_{9} - 30516\hat{\sigma}^z_{0}\hat{\sigma}^z_{18} - 8\hat{\sigma}^z_{4}\hat{\sigma}^z_{14} -$
$16\hat{\sigma}^z_{5}\hat{\sigma}^z_{13} + 40\hat{\sigma}^z_{6}\hat{\sigma}^z_{12} + 15258\hat{\sigma}^z_{7}\hat{\sigma}^z_{11} + 15258\hat{\sigma}^z_{8}\hat{\sigma}^z_{10} - 30516\hat{\sigma}^z_{0}\hat{\sigma}^z_{19} - 16\hat{\sigma}^z_{6}\hat{\sigma}^z_{13} + 40\hat{\sigma}^z_{7}\hat{\sigma}^z_{12} + 15258\hat{\sigma}^z_{8}\hat{\sigma}^z_{11} +$
$15258\hat{\sigma}^z_{9}\hat{\sigma}^z_{10} - 30516\hat{\sigma}^z_{0}\hat{\sigma}^z_{20} - 30516\hat{\sigma}^z_{5}\hat{\sigma}^z_{15} - 16\hat{\sigma}^z_{7}\hat{\sigma}^z_{13} + 40\hat{\sigma}^z_{8}\hat{\sigma}^z_{12} + 15258\hat{\sigma}^z_{9}\hat{\sigma}^z_{11} - 16\hat{\sigma}^z_{6}\hat{\sigma}^z_{15} - 16\hat{\sigma}^z_{8}\hat{\sigma}^z_{13} +$
$15258\hat{\sigma}^z_{9}\hat{\sigma}^z_{12} + 15258\hat{\sigma}^z_{10}\hat{\sigma}^z_{11} - 30516\hat{\sigma}^z_{1}\hat{\sigma}^z_{21} - 30516\hat{\sigma}^z_{6}\hat{\sigma}^z_{16} - 16\hat{\sigma}^z_{7}\hat{\sigma}^z_{15} - 16\hat{\sigma}^z_{9}\hat{\sigma}^z_{13} + 15258\hat{\sigma}^z_{10}\hat{\sigma}^z_{12} - 30516\hat{\sigma}^z_{1}\hat{\sigma}^z_{22} -$
$30516\hat{\sigma}^z_{2}\hat{\sigma}^z_{21} - 16\hat{\sigma}^z_{7}\hat{\sigma}^z_{16} + 8\hat{\sigma}^z_{8}\hat{\sigma}^z_{15} - 16\hat{\sigma}^z_{10}\hat{\sigma}^z_{13} + 15258\hat{\sigma}^z_{11}\hat{\sigma}^z_{12} - 30516\hat{\sigma}^z_{1}\hat{\sigma}^z_{23} - 8\hat{\sigma}^z_{3}\hat{\sigma}^z_{21} - 30516\hat{\sigma}^z_{7}\hat{\sigma}^z_{17} -$
$16\hat{\sigma}^z_{8}\hat{\sigma}^z_{16} - 16\hat{\sigma}^z_{11}\hat{\sigma}^z_{13} - 30516\hat{\sigma}^z_{1}\hat{\sigma}^z_{24} - 30516\hat{\sigma}^z_{3}\hat{\sigma}^z_{22} + 8\hat{\sigma}^z_{4}\hat{\sigma}^z_{21} - 16\hat{\sigma}^z_{8}\hat{\sigma}^z_{17} - 8\hat{\sigma}^z_{12}\hat{\sigma}^z_{13} - 30516\hat{\sigma}^z_{1}\hat{\sigma}^z_{25} - 8\hat{\sigma}^z_{4}\hat{\sigma}^z_{22} -$
$30516\hat{\sigma}^z_{1}\hat{\sigma}^z_{26} - 30516\hat{\sigma}^z_{9}\hat{\sigma}^z_{18} - 30516\hat{\sigma}^z_{1}\hat{\sigma}^z_{27} - 30516\hat{\sigma}^z_{5}\hat{\sigma}^z_{23} - 16\hat{\sigma}^z_{10}\hat{\sigma}^z_{18} - 30516\hat{\sigma}^z_{1}\hat{\sigma}^z_{28} - 16\hat{\sigma}^z_{6}\hat{\sigma}^z_{23} - 30516\hat{\sigma}^z_{10}\hat{\sigma}^z_{19} -$
$16\hat{\sigma}^z_{11}\hat{\sigma}^z_{18} - 30516\hat{\sigma}^z_{6}\hat{\sigma}^z_{24} - 16\hat{\sigma}^z_{11}\hat{\sigma}^z_{19} + 8\hat{\sigma}^z_{12}\hat{\sigma}^z_{18} - 30516\hat{\sigma}^z_{2}\hat{\sigma}^z_{29} - 16\hat{\sigma}^z_{7}\hat{\sigma}^z_{24} - 8\hat{\sigma}^z_{8}\hat{\sigma}^z_{23} - 30516\hat{\sigma}^z_{11}\hat{\sigma}^z_{20} - 16\hat{\sigma}^z_{12}\hat{\sigma}^z_{19} -$
$30516\hat{\sigma}^z_{2}\hat{\sigma}^z_{30} - 30516\hat{\sigma}^z_{3}\hat{\sigma}^z_{29} - 30516\hat{\sigma}^z_{7}\hat{\sigma}^z_{25} - 16\hat{\sigma}^z_{12}\hat{\sigma}^z_{20} - 30516\hat{\sigma}^z_{2}\hat{\sigma}^z_{31} - 16\hat{\sigma}^z_{4}\hat{\sigma}^z_{29} - 16\hat{\sigma}^z_{8}\hat{\sigma}^z_{25} - 30516\hat{\sigma}^z_{2}\hat{\sigma}^z_{32} -$
$30516\hat{\sigma}^z_{4}\hat{\sigma}^z_{30} - 8\hat{\sigma}^z_{5}\hat{\sigma}^z_{29} - 30516\hat{\sigma}^z_{2}\hat{\sigma}^z_{33} + 8\hat{\sigma}^z_{5}\hat{\sigma}^z_{30} - 30516\hat{\sigma}^z_{9}\hat{\sigma}^z_{26} - 30516\hat{\sigma}^z_{2}\hat{\sigma}^z_{34} - 30516\hat{\sigma}^z_{5}\hat{\sigma}^z_{31} - 16\hat{\sigma}^z_{10}\hat{\sigma}^z_{26} -$
$30516\hat{\sigma}^z_{2}\hat{\sigma}^z_{35} - 16\hat{\sigma}^z_{6}\hat{\sigma}^z_{31} - 8\hat{\sigma}^z_{8}\hat{\sigma}^z_{29} - 30516\hat{\sigma}^z_{10}\hat{\sigma}^z_{27} - 30516\hat{\sigma}^z_{2}\hat{\sigma}^z_{36} - 30516\hat{\sigma}^z_{6}\hat{\sigma}^z_{32} + 8\hat{\sigma}^z_{8}\hat{\sigma}^z_{30} - 8\hat{\sigma}^z_{9}\hat{\sigma}^z_{29} - 16\hat{\sigma}^z_{11}\hat{\sigma}^z_{27} -$

$$8\hat{\sigma}^z_{12}\hat{\sigma}^z_{26}-16\hat{\sigma}^z_{7}\hat{\sigma}^z_{32}+8\hat{\sigma}^z_{9}\hat{\sigma}^z_{30}-30516\hat{\sigma}^z_{11}\hat{\sigma}^z_{28}-30516\hat{\sigma}^z_{3}\hat{\sigma}^z_{37}-30516\hat{\sigma}^z_{7}\hat{\sigma}^z_{33}-8\hat{\sigma}^z_{11}\hat{\sigma}^z_{29}-16\hat{\sigma}^z_{12}\hat{\sigma}^z_{28}-30516\hat{\sigma}^z_{3}\hat{\sigma}^z_{38}-$$
$$30516\hat{\sigma}^z_{4}\hat{\sigma}^z_{37}-16\hat{\sigma}^z_{8}\hat{\sigma}^z_{33}+8\hat{\sigma}^z_{11}\hat{\sigma}^z_{30}-30516\hat{\sigma}^z_{3}\hat{\sigma}^z_{39}-16\hat{\sigma}^z_{5}\hat{\sigma}^z_{37}+8\hat{\sigma}^z_{13}\hat{\sigma}^z_{29}-30516\hat{\sigma}^z_{3}\hat{\sigma}^z_{40}-30516\hat{\sigma}^z_{5}\hat{\sigma}^z_{38}-16\hat{\sigma}^z_{6}\hat{\sigma}^z_{37}-$$
$$30516\hat{\sigma}^z_{9}\hat{\sigma}^z_{34}-8\hat{\sigma}^z_{13}\hat{\sigma}^z_{30}-30516\hat{\sigma}^z_{3}\hat{\sigma}^z_{41}-16\hat{\sigma}^z_{6}\hat{\sigma}^z_{38}-16\hat{\sigma}^z_{7}\hat{\sigma}^z_{37}-16\hat{\sigma}^z_{10}\hat{\sigma}^z_{34}-30516\hat{\sigma}^z_{3}\hat{\sigma}^z_{42}-30516\hat{\sigma}^z_{6}\hat{\sigma}^z_{39}-$$
$$16\hat{\sigma}^z_{8}\hat{\sigma}^z_{37}-30516\hat{\sigma}^z_{10}\hat{\sigma}^z_{35}-30516\hat{\sigma}^z_{3}\hat{\sigma}^z_{43}-16\hat{\sigma}^z_{7}\hat{\sigma}^z_{39}+8\hat{\sigma}^z_{8}\hat{\sigma}^z_{38}-16\hat{\sigma}^z_{9}\hat{\sigma}^z_{37}-16\hat{\sigma}^z_{11}\hat{\sigma}^z_{35}-30516\hat{\sigma}^z_{7}\hat{\sigma}^z_{40}-16\hat{\sigma}^z_{10}\hat{\sigma}^z_{37}-$$
$$30516\hat{\sigma}^z_{11}\hat{\sigma}^z_{36}-30516\hat{\sigma}^z_{4}\hat{\sigma}^z_{44}-16\hat{\sigma}^z_{8}\hat{\sigma}^z_{40}-16\hat{\sigma}^z_{11}\hat{\sigma}^z_{37}-16\hat{\sigma}^z_{12}\hat{\sigma}^z_{36}-30516\hat{\sigma}^z_{4}\hat{\sigma}^z_{45}-30516\hat{\sigma}^z_{5}\hat{\sigma}^z_{44}-8\hat{\sigma}^z_{12}\hat{\sigma}^z_{37}-$$
$$30516\hat{\sigma}^z_{9}\hat{\sigma}^z_{41}+8\hat{\sigma}^z_{13}\hat{\sigma}^z_{37}-30516\hat{\sigma}^z_{5}\hat{\sigma}^z_{46}-16\hat{\sigma}^z_{10}\hat{\sigma}^z_{41}-30516\hat{\sigma}^z_{5}\hat{\sigma}^z_{47}-30516\hat{\sigma}^z_{6}\hat{\sigma}^z_{46}-8\hat{\sigma}^z_{8}\hat{\sigma}^z_{44}-30516\hat{\sigma}^z_{10}\hat{\sigma}^z_{42}-$$
$$30516\hat{\sigma}^z_{5}\hat{\sigma}^z_{48}-64\hat{\sigma}^z_{7}\hat{\sigma}^z_{46}-16\hat{\sigma}^z_{11}\hat{\sigma}^z_{42}+8\hat{\sigma}^z_{12}\hat{\sigma}^z_{41}-30516\hat{\sigma}^z_{5}\hat{\sigma}^z_{49}-30516\hat{\sigma}^z_{7}\hat{\sigma}^z_{47}-64\hat{\sigma}^z_{8}\hat{\sigma}^z_{46}-30516\hat{\sigma}^z_{9}\hat{\sigma}^z_{45}-$$
$$30516\hat{\sigma}^z_{11}\hat{\sigma}^z_{43}-30516\hat{\sigma}^z_{5}\hat{\sigma}^z_{50}-64\hat{\sigma}^z_{8}\hat{\sigma}^z_{47}-32\hat{\sigma}^z_{9}\hat{\sigma}^z_{46}-16\hat{\sigma}^z_{12}\hat{\sigma}^z_{43}-30516\hat{\sigma}^z_{5}\hat{\sigma}^z_{51}-30516\hat{\sigma}^z_{8}\hat{\sigma}^z_{48}-16\hat{\sigma}^z_{9}\hat{\sigma}^z_{47}-$$
$$32\hat{\sigma}^z_{10}\hat{\sigma}^z_{46}-16\hat{\sigma}^z_{9}\hat{\sigma}^z_{48}-32\hat{\sigma}^z_{10}\hat{\sigma}^z_{47}-32\hat{\sigma}^z_{11}\hat{\sigma}^z_{46}-8\hat{\sigma}^z_{12}\hat{\sigma}^z_{45}-30516\hat{\sigma}^z_{6}\hat{\sigma}^z_{52}-30516\hat{\sigma}^z_{9}\hat{\sigma}^z_{49}-16\hat{\sigma}^z_{11}\hat{\sigma}^z_{47}-16\hat{\sigma}^z_{12}\hat{\sigma}^z_{46}-$$
$$30516\hat{\sigma}^z_{6}\hat{\sigma}^z_{53}-30516\hat{\sigma}^z_{7}\hat{\sigma}^z_{52}-32\hat{\sigma}^z_{10}\hat{\sigma}^z_{49}-16\hat{\sigma}^z_{11}\hat{\sigma}^z_{48}-16\hat{\sigma}^z_{12}\hat{\sigma}^z_{47}+16\hat{\sigma}^z_{13}\hat{\sigma}^z_{46}-30516\hat{\sigma}^z_{6}\hat{\sigma}^z_{54}-64\hat{\sigma}^z_{8}\hat{\sigma}^z_{52}-$$
$$30516\hat{\sigma}^z_{10}\hat{\sigma}^z_{50}-16\hat{\sigma}^z_{11}\hat{\sigma}^z_{49}-16\hat{\sigma}^z_{12}\hat{\sigma}^z_{48}-30516\hat{\sigma}^z_{6}\hat{\sigma}^z_{55}-30516\hat{\sigma}^z_{8}\hat{\sigma}^z_{53}-32\hat{\sigma}^z_{9}\hat{\sigma}^z_{52}-32\hat{\sigma}^z_{11}\hat{\sigma}^z_{50}-16\hat{\sigma}^z_{12}\hat{\sigma}^z_{49}-$$
$$30516\hat{\sigma}^z_{6}\hat{\sigma}^z_{56}-16\hat{\sigma}^z_{9}\hat{\sigma}^z_{53}-32\hat{\sigma}^z_{10}\hat{\sigma}^z_{52}-30516\hat{\sigma}^z_{11}\hat{\sigma}^z_{51}-16\hat{\sigma}^z_{12}\hat{\sigma}^z_{50}-30516\hat{\sigma}^z_{9}\hat{\sigma}^z_{54}-32\hat{\sigma}^z_{10}\hat{\sigma}^z_{53}-32\hat{\sigma}^z_{11}\hat{\sigma}^z_{52}-$$
$$32\hat{\sigma}^z_{12}\hat{\sigma}^z_{51}-30516\hat{\sigma}^z_{7}\hat{\sigma}^z_{57}-32\hat{\sigma}^z_{10}\hat{\sigma}^z_{54}-16\hat{\sigma}^z_{11}\hat{\sigma}^z_{53}-16\hat{\sigma}^z_{12}\hat{\sigma}^z_{52}-30516\hat{\sigma}^z_{7}\hat{\sigma}^z_{58}-30516\hat{\sigma}^z_{8}\hat{\sigma}^z_{57}-30516\hat{\sigma}^z_{10}\hat{\sigma}^z_{55}-$$
$$32\hat{\sigma}^z_{11}\hat{\sigma}^z_{54}-16\hat{\sigma}^z_{12}\hat{\sigma}^z_{53}+16\hat{\sigma}^z_{13}\hat{\sigma}^z_{52}-30516\hat{\sigma}^z_{7}\hat{\sigma}^z_{59}-32\hat{\sigma}^z_{9}\hat{\sigma}^z_{57}-32\hat{\sigma}^z_{11}\hat{\sigma}^z_{55}-16\hat{\sigma}^z_{12}\hat{\sigma}^z_{54}-30516\hat{\sigma}^z_{7}\hat{\sigma}^z_{60}-30516\hat{\sigma}^z_{9}\hat{\sigma}^z_{58}-$$
$$32\hat{\sigma}^z_{10}\hat{\sigma}^z_{57}-30516\hat{\sigma}^z_{11}\hat{\sigma}^z_{56}-32\hat{\sigma}^z_{12}\hat{\sigma}^z_{55}-32\hat{\sigma}^z_{10}\hat{\sigma}^z_{58}-32\hat{\sigma}^z_{11}\hat{\sigma}^z_{57}-32\hat{\sigma}^z_{12}\hat{\sigma}^z_{56}-30516\hat{\sigma}^z_{8}\hat{\sigma}^z_{61}-30516\hat{\sigma}^z_{10}\hat{\sigma}^z_{59}-$$
$$32\hat{\sigma}^z_{11}\hat{\sigma}^z_{58}-16\hat{\sigma}^z_{12}\hat{\sigma}^z_{57}-30516\hat{\sigma}^z_{8}\hat{\sigma}^z_{62}-30516\hat{\sigma}^z_{9}\hat{\sigma}^z_{61}-32\hat{\sigma}^z_{11}\hat{\sigma}^z_{59}-16\hat{\sigma}^z_{12}\hat{\sigma}^z_{58}+16\hat{\sigma}^z_{13}\hat{\sigma}^z_{57}-30516\hat{\sigma}^z_{8}\hat{\sigma}^z_{63}-$$
$$32\hat{\sigma}^z_{10}\hat{\sigma}^z_{61}-30516\hat{\sigma}^z_{11}\hat{\sigma}^z_{60}-32\hat{\sigma}^z_{12}\hat{\sigma}^z_{59}-30516\hat{\sigma}^z_{10}\hat{\sigma}^z_{62}-16\hat{\sigma}^z_{11}\hat{\sigma}^z_{61}-32\hat{\sigma}^z_{12}\hat{\sigma}^z_{60}-30516\hat{\sigma}^z_{9}\hat{\sigma}^z_{64}-32\hat{\sigma}^z_{11}\hat{\sigma}^z_{62}-$$
$$16\hat{\sigma}^z_{12}\hat{\sigma}^z_{61}-30516\hat{\sigma}^z_{9}\hat{\sigma}^z_{65}-30516\hat{\sigma}^z_{10}\hat{\sigma}^z_{64}-30516\hat{\sigma}^z_{11}\hat{\sigma}^z_{63}-16\hat{\sigma}^z_{12}\hat{\sigma}^z_{62}-30516\hat{\sigma}^z_{9}\hat{\sigma}^z_{66}-64\hat{\sigma}^z_{11}\hat{\sigma}^z_{64}-32\hat{\sigma}^z_{12}\hat{\sigma}^z_{63}+$$
$$16\hat{\sigma}^z_{29}\hat{\sigma}^z_{46}-30516\hat{\sigma}^z_{11}\hat{\sigma}^z_{65}-32\hat{\sigma}^z_{12}\hat{\sigma}^z_{64}-16\hat{\sigma}^z_{29}\hat{\sigma}^z_{47}-16\hat{\sigma}^z_{30}\hat{\sigma}^z_{46}-30516\hat{\sigma}^z_{10}\hat{\sigma}^z_{67}-64\hat{\sigma}^z_{12}\hat{\sigma}^z_{65}+16\hat{\sigma}^z_{13}\hat{\sigma}^z_{64}+$$
$$16\hat{\sigma}^z_{30}\hat{\sigma}^z_{47}-30516\hat{\sigma}^z_{10}\hat{\sigma}^z_{68}-30516\hat{\sigma}^z_{11}\hat{\sigma}^z_{67}-30516\hat{\sigma}^z_{12}\hat{\sigma}^z_{66}-32\hat{\sigma}^z_{12}\hat{\sigma}^z_{67}-30516\hat{\sigma}^z_{11}\hat{\sigma}^z_{69}-30516\hat{\sigma}^z_{12}\hat{\sigma}^z_{68}+$$
$$16\hat{\sigma}^z_{13}\hat{\sigma}^z_{67}-30516\hat{\sigma}^z_{12}\hat{\sigma}^z_{69}+16\hat{\sigma}^z_{29}\hat{\sigma}^z_{52}+16\hat{\sigma}^z_{13}\hat{\sigma}^z_{69}-16\hat{\sigma}^z_{29}\hat{\sigma}^z_{53}-16\hat{\sigma}^z_{30}\hat{\sigma}^z_{52}+16\hat{\sigma}^z_{30}\hat{\sigma}^z_{53}+16\hat{\sigma}^z_{37}\hat{\sigma}^z_{46}+16\hat{\sigma}^z_{29}\hat{\sigma}^z_{57}-$$
$$16\hat{\sigma}^z_{30}\hat{\sigma}^z_{57}+16\hat{\sigma}^z_{37}\hat{\sigma}^z_{52}+16\hat{\sigma}^z_{29}\hat{\sigma}^z_{64}-16\hat{\sigma}^z_{29}\hat{\sigma}^z_{65}-16\hat{\sigma}^z_{30}\hat{\sigma}^z_{64}+16\hat{\sigma}^z_{37}\hat{\sigma}^z_{57}+16\hat{\sigma}^z_{30}\hat{\sigma}^z_{65}+16\hat{\sigma}^z_{29}\hat{\sigma}^z_{67}-16\hat{\sigma}^z_{29}\hat{\sigma}^z_{68}-$$
$$16\hat{\sigma}^z_{30}\hat{\sigma}^z_{67}+16\hat{\sigma}^z_{29}\hat{\sigma}^z_{69}+16\hat{\sigma}^z_{30}\hat{\sigma}^z_{68}-16\hat{\sigma}^z_{30}\hat{\sigma}^z_{69}+16\hat{\sigma}^z_{37}\hat{\sigma}^z_{64}+64\hat{\sigma}^z_{46}\hat{\sigma}^z_{57}+16\hat{\sigma}^z_{37}\hat{\sigma}^z_{67}+16\hat{\sigma}^z_{37}\hat{\sigma}^z_{69}+32\hat{\sigma}^z_{46}\hat{\sigma}^z_{64}+$$
$$32\hat{\sigma}^z_{47}\hat{\sigma}^z_{65}+32\hat{\sigma}^z_{46}\hat{\sigma}^z_{67}+32\hat{\sigma}^z_{48}\hat{\sigma}^z_{66}+32\hat{\sigma}^z_{46}\hat{\sigma}^z_{69}+32\hat{\sigma}^z_{47}\hat{\sigma}^z_{68}+32\hat{\sigma}^z_{52}\hat{\sigma}^z_{64}+32\hat{\sigma}^z_{53}\hat{\sigma}^z_{65}+32\hat{\sigma}^z_{52}\hat{\sigma}^z_{67}+32\hat{\sigma}^z_{52}\hat{\sigma}^z_{69}+$$
$$32\hat{\sigma}^z_{53}\hat{\sigma}^z_{68}+32\hat{\sigma}^z_{57}\hat{\sigma}^z_{64}+32\hat{\sigma}^z_{57}\hat{\sigma}^z_{67}+32\hat{\sigma}^z_{57}\hat{\sigma}^z_{69}+64\hat{\sigma}^z_{64}\hat{\sigma}^z_{69}+2610926$$

Complete expressions for Turyn type H-matrix of order 92

A complete expression of $E_k(s)$ in 92-order H matrix using Turyn based method

$H_k(s) = 2s_0+4s_2+2s_3-4s_4+4s_5-2s_6+2s_7-4s_8+8s_9+8s_{10}+8s_{11}+8s_{12}+8s_{13}+8s_{14}+8s_{16}+8s_{18}+8s_{20}+$
$6s_0s_2+4s_0s_4-2s_1s_3+4s_0s_5-2s_0s_6+2s_1s_5+2s_0s_7+6s_1s_6+2s_2s_5+4s_3s_4-4s_0s_8-6s_1s_7-2s_2s_6-4s_3s_5+4s_0s_9-$
$2s_1s_8+2s_2s_7-2s_3s_6-2s_4s_5-2s_2s_8+2s_3s_7+2s_4s_6+4s_0s_{11}+4s_1s_{10}+4s_3s_8-2s_4s_7+4s_0s_{12}+4s_1s_{11}+4s_2s_{10}+$
$4s_3s_9+2s_4s_8+2s_5s_7+4s_1s_{12}+4s_2s_{11}-8s_5s_8-8s_6s_7+4s_0s_{14}+4s_1s_{13}+4s_2s_{12}+4s_3s_{11}+2s_6s_8+4s_0s_{15}+4s_2s_{13}+$
$4s_3s_{12}+4s_0s_{16}+4s_1s_{16}+4s_3s_{14}+4s_0s_{18}+8s_1s_{17}+4s_2s_{16}+4s_3s_{15}-4s_0s_{19}-4s_1s_{18}-4s_3s_{16}+8s_4s_{15}+4s_2s_{18}+$
$8s_9s_{11}+4s_3s_{18}+4s_5s_{16}+4s_3s_{19}+8s_9s_{13}+8s_{10}s_{12}+4s_6s_{17}+16s_9s_{14}+16s_{10}s_{13}+16s_{11}s_{12}-8s_4s_{20}-4s_5s_{19}-$
$4s_6s_{18}-4s_7s_{17}-4s_8s_{16}+8s_9s_{15}+8s_{10}s_{14}+8s_{11}s_{13}+4s_7s_{18}+8s_{10}s_{16}+8s_{12}s_{14}+4s_8s_{19}+8s_{11}s_{17}+8s_9s_{20}+$
$8s_{10}s_{19}+8s_{11}s_{18}+8s_{12}s_{17}+8s_{13}s_{16}+8s_{14}s_{15}+8s_{12}s_{18}+8s_{13}s_{19}+8s_{15}s_{17}+8s_{14}s_{20}+8s_{15}s_{19}+8s_{16}s_{18}+8s_{16}s_{20}+$
$8s_{17}s_{19}+8s_{18}s_{20}+4s_0s_1s_2+8s_0s_1s_3+4s_0s_1s_4+4s_1s_2s_3+2s_0s_2s_5+4s_1s_2s_4+2s_0s_3s_6+2s_1s_3s_5+4s_2s_3s_4+4s_0s_1s_9-$
$2s_0s_2s_8-2s_0s_3s_7+2s_0s_4s_6+2s_1s_4s_5-2s_0s_4s_7+2s_0s_5s_6-2s_1s_4s_6-2s_2s_4s_5+4s_0s_2s_{10}+2s_0s_5s_7+4s_1s_2s_9-$
$2s_1s_3s_8+2s_1s_4s_7+2s_2s_4s_6+2s_0s_6s_7-2s_1s_4s_8+2s_1s_5s_7-2s_2s_4s_7-2s_3s_4s_6+4s_0s_3s_{11}+2s_0s_6s_8+4s_1s_3s_{10}+$
$4s_1s_5s_8+4s_2s_3s_9+2s_2s_4s_8+2s_2s_5s_7+2s_3s_4s_7+2s_3s_5s_6+4s_0s_1s_{14}+4s_0s_2s_{13}+4s_0s_3s_{12}+2s_0s_7s_8+2s_1s_6s_8-$
$2s_3s_5s_7+8s_4s_5s_6+4s_0s_1s_{15}+2s_2s_6s_8+2s_3s_6s_7-4s_4s_5s_7+4s_1s_2s_{14}+4s_1s_3s_{13}-2s_3s_6s_8+8s_4s_6s_7+4s_0s_2s_{16}+$
$4s_1s_2s_{15}+2s_3s_7s_8+4s_4s_5s_9-4s_4s_6s_8+4s_0s_9s_{10}+4s_2s_3s_{14}+8s_4s_7s_8+4s_5s_6s_8+4s_0s_3s_{17}+4s_0s_9s_{11}+4s_1s_3s_{16}+$
$4s_2s_3s_{15}-4s_4s_5s_{11}+4s_4s_6s_{10}+4s_5s_6s_9-4s_5s_7s_8+4s_0s_{10}s_{11}+4s_1s_9s_{11}-4s_4s_5s_{12}-4s_4s_6s_{11}-4s_4s_7s_{10}-$
$4s_4s_8s_9+4s_0s_9s_{13}+4s_0s_{10}s_{12}+8s_1s_9s_{12}+4s_2s_9s_{11}+4s_3s_9s_{10}-4s_4s_6s_{12}+4s_4s_7s_{11}+4s_5s_7s_{10}+4s_6s_7s_9-$
$4s_0s_9s_{14}+4s_0s_{11}s_{12}-4s_1s_9s_{13}+4s_1s_{10}s_{12}-4s_3s_9s_{11}+4s_4s_5s_{14}+4s_4s_6s_{13}+4s_4s_7s_{12}+4s_4s_8s_{11}+8s_4s_9s_{10}+$
$4s_0s_{10}s_{14}+4s_0s_{11}s_{13}+8s_1s_{10}s_{13}+4s_2s_9s_{13}+4s_2s_{10}s_{12}+4s_3s_{10}s_{11}+4s_4s_5s_{15}-4s_4s_7s_{13}+4s_4s_8s_{12}+4s_5s_8s_{11}+$
$4s_6s_8s_{10}+4s_7s_8s_9+4s_0s_{12}s_{13}-4s_1s_{10}s_{14}+4s_1s_{11}s_{13}+4s_3s_9s_{13}-4s_3s_{10}s_{12}+8s_4s_{10}s_{11}+4s_5s_6s_{14}+4s_5s_7s_{13}+$
$4s_5s_8s_{12}+4s_5s_9s_{11}+4s_0s_{12}s_{14}+8s_1s_{11}s_{14}+4s_2s_{10}s_{14}+4s_2s_{11}s_{13}+4s_3s_9s_{14}+4s_3s_{11}s_{12}+4s_4s_6s_{16}-4s_4s_8s_{14}+$
$4s_5s_6s_{15}+4s_0s_{13}s_{14}+4s_1s_{12}s_{14}+4s_3s_{10}s_{14}-4s_3s_{11}s_{13}-4s_4s_5s_{18}-4s_4s_6s_{17}-4s_4s_7s_{16}-4s_4s_8s_{15}+8s_4s_{11}s_{12}+$
$4s_5s_{10}s_{12}+4s_6s_7s_{14}+4s_6s_8s_{13}+4s_6s_9s_{12}+4s_2s_{12}s_{14}+4s_3s_{12}s_{13}+4s_4s_7s_{17}+4s_5s_7s_{16}-4s_5s_9s_{14}+4s_6s_7s_{15}-$
$4s_6s_9s_{13}-4s_7s_9s_{12}-4s_8s_9s_{11}-4s_3s_{12}s_{14}+8s_4s_{12}s_{13}+4s_5s_{11}s_{13}+4s_6s_{10}s_{13}+4s_7s_8s_{14}+4s_7s_9s_{13}+4s_3s_{13}s_{14}+$
$4s_4s_8s_{18}+4s_5s_8s_{17}+4s_6s_8s_{16}-4s_6s_{10}s_{14}+4s_7s_8s_{15}-4s_7s_{10}s_{13}-4s_8s_{10}s_{12}+16s_9s_{10}s_{11}+4s_0s_{15}s_{16}+8s_4s_{13}s_{14}+$
$4s_5s_{12}s_{14}+4s_6s_{11}s_{14}+4s_7s_{10}s_{14}+4s_8s_9s_{14}+4s_0s_{15}s_{17}-4s_7s_{11}s_{14}-4s_8s_{11}s_{13}+16s_9s_{11}s_{12}+4s_0s_{16}s_{17}+$
$4s_1s_{15}s_{17}+16s_9s_{10}s_{14}+16s_9s_{11}s_{13}+4s_0s_{15}s_{19}+4s_0s_{16}s_{18}+8s_1s_{15}s_{18}+4s_2s_{15}s_{17}+4s_3s_{15}s_{16}-4s_8s_{12}s_{14}+$
$8s_9s_{10}s_{15}+16s_9s_{12}s_{13}+16s_{10}s_{11}s_{13}-4s_0s_{15}s_{20}+4s_0s_{17}s_{18}-4s_1s_{15}s_{19}+4s_1s_{16}s_{18}-4s_3s_{15}s_{17}+8s_4s_{15}s_{16}+$
$16s_{10}s_{11}s_{14}+16s_{10}s_{12}s_{13}+4s_0s_{16}s_{20}+4s_0s_{17}s_{19}+8s_1s_{16}s_{19}+4s_2s_{15}s_{19}+4s_2s_{16}s_{18}+4s_3s_{16}s_{17}+8s_9s_{11}s_{16}+$
$16s_9s_{13}s_{14}+8s_{10}s_{11}s_{15}+16s_{10}s_{12}s_{14}+4s_0s_{18}s_{19}-4s_1s_{16}s_{20}+4s_1s_{17}s_{19}+4s_3s_{15}s_{19}-4s_3s_{16}s_{18}+8s_4s_{16}s_{17}+$
$4s_5s_{15}s_{17}+16s_{11}s_{12}s_{14}+4s_0s_{18}s_{20}+8s_1s_{17}s_{20}+4s_2s_{16}s_{20}+4s_2s_{17}s_{19}+4s_3s_{15}s_{20}+4s_3s_{17}s_{18}+8s_9s_{12}s_{17}+$
$8s_{10}s_{12}s_{16}+8s_{11}s_{12}s_{15}+4s_0s_{19}s_{20}+4s_1s_{18}s_{20}+4s_3s_{16}s_{20}-4s_3s_{17}s_{19}+8s_4s_{17}s_{18}+4s_5s_{16}s_{18}+4s_6s_{15}s_{18}+$
$16s_{12}s_{13}s_{14}+4s_2s_{18}s_{20}+4s_3s_{18}s_{19}-4s_5s_{15}s_{20}-4s_6s_{15}s_{19}-4s_7s_{15}s_{18}-4s_8s_{15}s_{17}+8s_9s_{13}s_{18}+8s_9s_{15}s_{16}+$
$8s_{10}s_{13}s_{17}+8s_{11}s_{13}s_{16}+8s_{12}s_{13}s_{15}-4s_3s_{18}s_{20}+8s_4s_{18}s_{19}+4s_5s_{17}s_{19}+4s_6s_{16}s_{19}+4s_7s_{15}s_{19}+4s_3s_{19}s_{20}-$
$4s_6s_{16}s_{20}-4s_7s_{16}s_{19}-4s_8s_{16}s_{18}+8s_9s_{14}s_{19}+8s_9s_{16}s_{17}+8s_{10}s_{14}s_{18}+8s_{10}s_{15}s_{17}+8s_{11}s_{14}s_{17}+8s_{12}s_{14}s_{16}+$
$8s_{13}s_{14}s_{15}+8s_4s_{19}s_{20}+4s_5s_{18}s_{20}+4s_6s_{17}s_{20}+4s_7s_{16}s_{20}+4s_8s_{15}s_{20}-4s_7s_{17}s_{20}-4s_8s_{17}s_{19}+8s_9s_{17}s_{18}+$
$8s_{10}s_{16}s_{18}+8s_{11}s_{15}s_{18}+8s_{10}s_{15}s_{20}+8s_{11}s_{15}s_{19}+8s_{12}s_{15}s_{18}+8s_{13}s_{15}s_{17}+8s_{14}s_{15}s_{16}-4s_8s_{18}s_{20}+8s_9s_{18}s_{19}+$
$8s_{10}s_{17}s_{19}+8s_{11}s_{16}s_{19}+8s_{12}s_{15}s_{19}+8s_{11}s_{16}s_{20}+8s_{12}s_{16}s_{19}+8s_{13}s_{16}s_{18}+8s_{14}s_{16}s_{17}+8s_9s_{19}s_{20}+8s_{10}s_{18}s_{20}+$
$8s_{11}s_{17}s_{20}+8s_{12}s_{16}s_{20}+8s_{13}s_{15}s_{20}+16s_{15}s_{16}s_{17}+8s_{12}s_{17}s_{20}+8s_{13}s_{17}s_{19}+8s_{14}s_{17}s_{18}+16s_{15}s_{17}s_{18}+8s_{13}s_{18}s_{20}+$
$8s_{14}s_{18}s_{19}+16s_{15}s_{18}s_{19}+16s_{16}s_{17}s_{19}+8s_{14}s_{19}s_{20}+16s_{15}s_{19}s_{20}+16s_{16}s_{18}s_{20}+4s_0s_1s_2s_3+2s_0s_1s_4s_5+2s_0s_1s_5s_6+$
$2s_0s_2s_4s_6+2s_1s_2s_4s_5-2s_0s_1s_4s_8-2s_0s_2s_4s_7-2s_0s_3s_4s_6+2s_0s_1s_6s_7+2s_0s_2s_5s_7+2s_0s_3s_4s_7+2s_1s_2s_5s_6+$
$2s_1s_3s_4s_6+2s_2s_3s_4s_5-2s_1s_2s_4s_8-2s_1s_3s_4s_7+2s_0s_1s_7s_8+2s_0s_2s_6s_8+2s_0s_3s_5s_8+2s_1s_2s_6s_7+2s_1s_3s_5s_7+$
$2s_2s_3s_5s_6-2s_2s_3s_4s_8+2s_1s_2s_7s_8+2s_1s_3s_6s_8+2s_2s_3s_6s_7+4s_0s_1s_9s_{10}+2s_2s_3s_7s_8+4s_0s_1s_{10}s_{11}+4s_0s_2s_9s_{11}+$
$4s_1s_2s_9s_{10}+4s_4s_5s_6s_7-4s_4s_5s_6s_8+4s_0s_1s_{11}s_{12}+4s_0s_2s_{10}s_{12}+4s_0s_3s_9s_{12}+4s_1s_2s_{10}s_{11}+4s_1s_3s_9s_{11}+4s_2s_3s_9s_{10}+$
$4s_4s_5s_7s_8-4s_4s_6s_7s_8+4s_0s_1s_{12}s_{13}+4s_0s_2s_{11}s_{13}+4s_0s_3s_{10}s_{13}+4s_1s_2s_{11}s_{12}+4s_1s_3s_{10}s_{12}+4s_2s_3s_{10}s_{11}+$
$4s_5s_6s_7s_8+4s_0s_1s_{13}s_{14}+4s_0s_2s_{12}s_{14}+4s_0s_3s_{11}s_{14}+4s_1s_2s_{12}s_{13}+4s_1s_3s_{11}s_{13}+4s_2s_3s_{11}s_{12}+4s_4s_5s_9s_{10}+$

$4s_1s_2s_{13}s_{14} + 4s_1s_3s_{12}s_{14} + 4s_2s_3s_{12}s_{13} + 4s_4s_5s_{10}s_{11} + 4s_4s_6s_9s_{11} + 4s_5s_6s_9s_{10} - 4s_4s_5s_9s_{13} - 4s_4s_6s_9s_{12} -$
$4s_4s_7s_9s_{11} - 4s_4s_8s_9s_{10} + 4s_0s_1s_{15}s_{16} + 4s_2s_3s_{13}s_{14} + 4s_4s_5s_{11}s_{12} + 4s_4s_6s_{10}s_{12} + 4s_4s_7s_9s_{12} + 4s_5s_6s_{10}s_{11} +$
$4s_5s_7s_9s_{11} + 4s_6s_7s_9s_{10} - 4s_4s_5s_{10}s_{14} - 4s_4s_6s_{10}s_{13} - 4s_4s_7s_{10}s_{12} - 4s_4s_8s_{10}s_{11} + 4s_0s_1s_{16}s_{17} + 4s_0s_2s_{15}s_{17} +$
$4s_1s_2s_{15}s_{16} + 4s_4s_5s_{12}s_{13} + 4s_4s_6s_{11}s_{13} + 4s_4s_7s_{10}s_{13} + 4s_4s_8s_9s_{13} + 4s_5s_6s_{11}s_{12} + 4s_5s_7s_{10}s_{12} + 4s_5s_8s_9s_{12} +$
$4s_6s_7s_{10}s_{11} + 4s_6s_8s_9s_{11} + 4s_7s_8s_9s_{10} - 4s_4s_6s_{11}s_{14} - 4s_4s_7s_{11}s_{13} - 4s_4s_8s_{11}s_{12} + 4s_0s_1s_{17}s_{18} + 4s_0s_2s_{16}s_{18} +$
$4s_0s_3s_{15}s_{18} + 4s_1s_2s_{16}s_{17} + 4s_1s_3s_{15}s_{17} + 4s_2s_3s_{15}s_{16} + 4s_4s_5s_{13}s_{14} + 4s_4s_6s_{12}s_{14} + 4s_4s_7s_{11}s_{14} + 4s_4s_8s_{10}s_{14} +$
$4s_5s_6s_{12}s_{13} + 4s_5s_7s_{11}s_{13} + 4s_5s_8s_{10}s_{13} + 4s_6s_7s_{11}s_{12} + 4s_6s_8s_{10}s_{12} + 4s_7s_8s_{10}s_{11} - 4s_4s_7s_{12}s_{14} - 4s_4s_8s_{12}s_{13} +$
$4s_0s_1s_{18}s_{19} + 4s_0s_2s_{17}s_{19} + 4s_0s_3s_{16}s_{19} + 4s_1s_2s_{17}s_{18} + 4s_1s_3s_{16}s_{18} + 4s_2s_3s_{16}s_{17} + 4s_5s_6s_{13}s_{14} + 4s_5s_7s_{12}s_{14} +$
$4s_5s_8s_{11}s_{14} + 4s_6s_7s_{12}s_{13} + 4s_6s_8s_{11}s_{13} + 4s_7s_8s_{11}s_{12} - 4s_4s_8s_{13}s_{14} + 4s_0s_1s_{19}s_{20} + 4s_0s_2s_{18}s_{20} + 4s_0s_3s_{17}s_{20} +$
$4s_1s_2s_{18}s_{19} + 4s_1s_3s_{17}s_{19} + 4s_2s_3s_{17}s_{18} + 4s_4s_5s_{15}s_{16} + 4s_6s_7s_{13}s_{14} + 4s_6s_8s_{12}s_{14} + 4s_7s_8s_{12}s_{13} + 4s_1s_2s_{19}s_{20} +$
$4s_1s_3s_{18}s_{20} + 4s_2s_3s_{18}s_{19} + 4s_4s_5s_{16}s_{17} + 4s_4s_6s_{15}s_{17} + 4s_5s_6s_{15}s_{16} + 4s_7s_8s_{13}s_{14} + 16s_9s_{10}s_{11}s_{12} - 4s_4s_5s_{15}s_{19} -$
$4s_4s_6s_{15}s_{18} - 4s_4s_7s_{15}s_{17} - 4s_4s_8s_{15}s_{16} + 4s_2s_3s_{19}s_{20} + 4s_4s_5s_{17}s_{18} + 4s_4s_6s_{16}s_{18} + 4s_4s_7s_{15}s_{18} + 4s_5s_6s_{16}s_{17} +$
$4s_5s_7s_{15}s_{17} + 4s_6s_7s_{15}s_{16} + 16s_9s_{10}s_{12}s_{13} - 4s_4s_5s_{16}s_{20} - 4s_4s_6s_{16}s_{19} - 4s_4s_7s_{16}s_{18} - 4s_4s_8s_{16}s_{17} + 4s_4s_5s_{18}s_{19} +$
$4s_4s_6s_{17}s_{19} + 4s_4s_7s_{16}s_{19} + 4s_4s_8s_{15}s_{19} + 4s_5s_6s_{17}s_{18} + 4s_5s_7s_{16}s_{18} + 4s_5s_8s_{15}s_{18} + 4s_6s_7s_{16}s_{17} + 4s_6s_8s_{15}s_{17} +$
$4s_7s_8s_{15}s_{16} + 16s_9s_{10}s_{13}s_{14} + 16s_9s_{11}s_{12}s_{14} + 16s_{10}s_{11}s_{12}s_{13} - 4s_4s_6s_{17}s_{20} - 4s_4s_7s_{17}s_{19} - 4s_4s_8s_{17}s_{18} +$
$4s_4s_5s_{19}s_{20} + 4s_4s_6s_{18}s_{20} + 4s_4s_7s_{17}s_{20} + 4s_4s_8s_{16}s_{20} + 4s_5s_6s_{18}s_{19} + 4s_5s_7s_{17}s_{19} + 4s_5s_8s_{16}s_{19} + 4s_6s_7s_{17}s_{18} +$
$4s_6s_8s_{16}s_{18} + 4s_7s_8s_{16}s_{17} + 16s_{10}s_{11}s_{13}s_{14} - 4s_4s_7s_{18}s_{20} - 4s_4s_8s_{18}s_{19} + 4s_5s_6s_{19}s_{20} + 4s_5s_7s_{18}s_{20} + 4s_5s_8s_{17}s_{20} +$
$4s_6s_7s_{18}s_{19} + 4s_6s_8s_{17}s_{19} + 4s_7s_8s_{17}s_{18} + 8s_9s_{10}s_{15}s_{16} + 16s_{11}s_{12}s_{13}s_{14} - 4s_4s_8s_{19}s_{20} + 4s_6s_7s_{19}s_{20} + 4s_6s_8s_{18}s_{20} +$
$4s_7s_8s_{18}s_{19} + 8s_9s_{10}s_{16}s_{17} + 8s_9s_{11}s_{15}s_{17} + 8s_{10}s_{11}s_{15}s_{16} + 4s_7s_8s_{19}s_{20} + 8s_9s_{10}s_{17}s_{18} + 8s_9s_{11}s_{16}s_{18} + 8s_9s_{12}s_{15}s_{18} +$
$8s_{10}s_{11}s_{16}s_{17} + 8s_{10}s_{12}s_{15}s_{17} + 8s_{11}s_{12}s_{15}s_{16} + 8s_9s_{10}s_{18}s_{19} + 8s_9s_{11}s_{17}s_{19} + 8s_9s_{12}s_{16}s_{19} + 8s_9s_{13}s_{15}s_{19} +$
$8s_{10}s_{11}s_{17}s_{18} + 8s_{10}s_{12}s_{16}s_{18} + 8s_{10}s_{13}s_{15}s_{18} + 8s_{11}s_{12}s_{16}s_{17} + 8s_{11}s_{13}s_{15}s_{17} + 8s_{12}s_{13}s_{15}s_{16} + 8s_9s_{10}s_{19}s_{20} +$
$8s_9s_{11}s_{18}s_{20} + 8s_9s_{12}s_{17}s_{20} + 8s_9s_{13}s_{16}s_{20} + 8s_9s_{14}s_{15}s_{20} + 8s_{10}s_{11}s_{18}s_{19} + 8s_{10}s_{12}s_{17}s_{19} + 8s_{10}s_{13}s_{16}s_{19} +$
$8s_{10}s_{14}s_{15}s_{19} + 8s_{11}s_{12}s_{17}s_{18} + 8s_{11}s_{13}s_{16}s_{18} + 8s_{11}s_{14}s_{15}s_{18} + 8s_{12}s_{13}s_{16}s_{17} + 8s_{12}s_{14}s_{15}s_{17} + 8s_{13}s_{14}s_{15}s_{16} +$
$8s_{10}s_{11}s_{19}s_{20} + 8s_{10}s_{12}s_{18}s_{20} + 8s_{10}s_{13}s_{17}s_{20} + 8s_{10}s_{14}s_{16}s_{20} + 8s_{11}s_{12}s_{18}s_{19} + 8s_{11}s_{13}s_{17}s_{19} + 8s_{11}s_{14}s_{16}s_{19} +$
$8s_{12}s_{13}s_{17}s_{18} + 8s_{12}s_{14}s_{16}s_{18} + 8s_{13}s_{14}s_{16}s_{17} + 8s_{11}s_{12}s_{19}s_{20} + 8s_{11}s_{13}s_{18}s_{20} + 8s_{11}s_{14}s_{17}s_{20} + 8s_{12}s_{13}s_{18}s_{19} +$
$8s_{12}s_{14}s_{17}s_{19} + 8s_{13}s_{14}s_{17}s_{18} + 8s_{12}s_{13}s_{19}s_{20} + 8s_{12}s_{14}s_{18}s_{20} + 8s_{13}s_{14}s_{18}s_{19} + 8s_{13}s_{14}s_{19}s_{20} + 16s_{15}s_{16}s_{17}s_{18} +$
$16s_{15}s_{16}s_{18}s_{19} + 16s_{15}s_{16}s_{19}s_{20} + 16s_{15}s_{17}s_{18}s_{20} + 16s_{16}s_{17}s_{18}s_{19} + 16s_{16}s_{17}s_{19}s_{20} + 16s_{17}s_{18}s_{19}s_{20} + 244$

A complete expression of $E_k(q)$ in 92-order H matrix using Turyn based method

$E_k(q) = 208q_0q_1 - 336q_1 - 320q_2 - 264q_3 - 144q_4 - 272q_5 - 248q_6 - 200q_7 - 96q_8 - 328q_9 - 384q_{10} -$
$408q_{11} - 408q_{12} - 384q_{13} - 328q_{14} - 328q_{15} - 368q_{16} - 376q_{17} - 368q_{18} - 328q_{19} - 248q_{20} - 344q_0 + 160q_0q_2 +$
$128q_0q_3 + 192q_1q_2 + 32q_0q_4 + 160q_1q_3 + 72q_0q_5 + 32q_1q_4 + 176q_2q_3 + 56q_0q_6 + 88q_1q_5 + 32q_2q_4 + 40q_0q_7 +$
$72q_1q_6 + 56q_2q_5 + 32q_3q_4 + 8q_0q_8 + 24q_1q_7 + 56q_2q_6 + 24q_3q_5 + 56q_0q_9 + 8q_1q_8 + 40q_2q_7 + 24q_3q_6 + 96q_4q_5 +$
$72q_0q_{10} + 56q_1q_9 + 8q_2q_8 + 40q_3q_7 + 64q_4q_6 + 88q_0q_{11} + 72q_1q_{10} + 56q_2q_9 + 24q_3q_8 + 32q_4q_7 + 208q_5q_6 +$
$88q_0q_{12} + 88q_1q_{11} + 72q_2q_{10} + 56q_3q_9 - 64q_4q_8 + 144q_5q_7 + 72q_0q_{13} + 88q_1q_{12} + 72q_2q_{11} + 56q_3q_{10} + 16q_4q_9 +$
$80q_5q_8 + 160q_6q_7 + 56q_0q_{14} + 72q_1q_{13} + 72q_2q_{12} + 56q_3q_{11} + 32q_4q_{10} + 40q_5q_9 + 112q_6q_8 + 56q_0q_{15} + 56q_1q_{14} +$
$72q_2q_{13} + 56q_3q_{12} + 32q_4q_{11} + 56q_5q_{10} + 40q_6q_9 + 176q_7q_8 + 80q_0q_{16} + 56q_1q_{15} + 56q_2q_{14} + 56q_3q_{13} +$
$32q_4q_{12} + 72q_5q_{11} + 56q_6q_{10} + 40q_7q_9 + 80q_0q_{17} + 72q_1q_{16} + 56q_2q_{15} + 56q_3q_{14} + 32q_4q_{13} + 72q_5q_{12} +$
$56q_6q_{11} + 40q_7q_{10} + 24q_8q_9 + 80q_0q_{18} + 96q_1q_{17} + 72q_2q_{16} + 56q_3q_{15} + 16q_4q_{14} + 56q_5q_{13} + 56q_6q_{12} +$
$40q_7q_{11} + 24q_8q_{10} + 56q_0q_{19} + 72q_1q_{18} + 64q_2q_{17} + 48q_3q_{16} + 32q_4q_{15} + 40q_5q_{14} + 56q_6q_{13} + 40q_7q_{12} +$
$24q_8q_{11} + 208q_9q_{10} + 40q_0q_{20} + 56q_1q_{19} + 72q_2q_{18} + 48q_3q_{17} + 32q_4q_{16} + 40q_5q_{15} + 40q_6q_{14} + 40q_7q_{13} +$
$24q_8q_{12} + 176q_9q_{11} + 40q_1q_{20} + 56q_2q_{19} + 48q_3q_{18} + 32q_4q_{17} + 64q_5q_{16} + 40q_6q_{15} + 40q_7q_{14} + 24q_8q_{13} +$
$144q_9q_{12} + 224q_{10}q_{11} + 40q_2q_{20} + 56q_3q_{19} + 32q_4q_{18} + 80q_5q_{17} + 56q_6q_{16} + 40q_7q_{15} + 24q_8q_{14} + 128q_9q_{13} +$
$176q_{10}q_{12} + 40q_3q_{20} + 32q_4q_{19} + 64q_5q_{18} + 64q_6q_{17} + 40q_7q_{16} + 24q_8q_{15} + 96q_9q_{14} + 176q_{10}q_{13} + 240q_{11}q_{12} +$
$40q_5q_{19} + 56q_6q_{18} + 32q_7q_{17} + 16q_8q_{16} + 64q_9q_{15} + 128q_{10}q_{14} + 176q_{11}q_{13} + 24q_5q_{20} + 40q_6q_{19} + 40q_7q_{18} +$
$16q_8q_{17} + 64q_9q_{16} + 72q_{10}q_{15} + 144q_{11}q_{14} + 224q_{12}q_{13} + 24q_6q_{20} + 40q_7q_{19} + 16q_8q_{18} + 64q_9q_{17} + 80q_{10}q_{16} +$
$72q_{11}q_{15} + 176q_{12}q_{14} + 24q_7q_{20} + 24q_8q_{19} + 64q_9q_{18} + 80q_{10}q_{17} + 88q_{11}q_{16} + 72q_{12}q_{15} + 208q_{13}q_{14} + 24q_8q_{20} +$
$64q_9q_{19} + 80q_{10}q_{18} + 96q_{11}q_{17} + 88q_{12}q_{16} + 72q_{13}q_{15} + 56q_9q_{20} + 72q_{10}q_{19} + 88q_{11}q_{18} + 96q_{12}q_{17} + 80q_{13}q_{16} +$

$64q_{14}q_{15} + 56q_{10}q_{20} + 72q_{11}q_{19} + 88q_{12}q_{18} + 80q_{13}q_{17} + 64q_{14}q_{16} + 56q_{11}q_{20} + 72q_{12}q_{19} + 80q_{13}q_{18} +$
$64q_{14}q_{17} + 200q_{15}q_{16} + 56q_{12}q_{20} + 72q_{13}q_{19} + 64q_{14}q_{18} + 168q_{15}q_{17} + 56q_{13}q_{20} + 64q_{14}q_{19} + 152q_{15}q_{18} +$
$216q_{16}q_{17}+56q_{14}q_{20}+120q_{15}q_{19}+168q_{16}q_{18}+72q_{15}q_{20}+152q_{16}q_{19}+216q_{17}q_{18}+104q_{16}q_{20}+168q_{17}q_{19}+$
$120q_{17}q_{20}+200q_{18}q_{19}+152q_{18}q_{20}+200q_{19}q_{20}-64q_0q_1q_2-96q_0q_1q_3-32q_0q_1q_4-32q_0q_2q_3-32q_0q_1q_5-$
$64q_1q_2q_3-32q_0q_1q_6-32q_0q_2q_5-32q_1q_2q_4-32q_0q_1q_7-32q_0q_2q_6-16q_0q_3q_5-32q_1q_2q_5-16q_0q_4q_5-$
$32q_1q_2q_6-32q_1q_3q_5-32q_2q_3q_4-32q_0q_1q_9-16q_0q_4q_6-32q_1q_2q_7-32q_1q_3q_6-48q_1q_4q_5-32q_2q_3q_5-$
$32q_0q_1q_{10}-16q_0q_2q_9-16q_0q_3q_8+16q_0q_4q_7-32q_0q_5q_6-32q_2q_3q_6-16q_2q_4q_5-32q_0q_1q_{11}-32q_0q_2q_{10}-$
$16q_0q_3q_9+16q_0q_4q_8-32q_0q_5q_7-32q_1q_2q_9-32q_1q_5q_6-32q_2q_3q_7-32q_2q_4q_6-16q_3q_4q_5-32q_0q_1q_{12}-$
$32q_0q_2q_{11}-16q_0q_3q_{10}-16q_0q_5q_8-32q_0q_6q_7-32q_1q_2q_{10}-16q_1q_3q_9+48q_1q_4q_8-32q_1q_5q_7+32q_2q_4q_7-$
$32q_2q_5q_6+16q_3q_4q_6-32q_0q_1q_{13}-32q_0q_2q_{12}-32q_0q_3q_{11}-32q_0q_6q_8-32q_1q_2q_{11}-32q_1q_3q_{10}-32q_1q_5q_8-$
$32q_1q_6q_7-32q_2q_3q_9+16q_2q_4q_8-32q_2q_5q_7-16q_3q_4q_7-32q_3q_5q_6-32q_0q_1q_{14}-32q_0q_2q_{13}-32q_0q_3q_{12}-$
$32q_0q_7q_8-32q_1q_2q_{12}-32q_1q_3q_{11}-32q_1q_6q_8-32q_2q_3q_{10}-32q_2q_6q_7+16q_3q_4q_8-64q_4q_5q_6-32q_0q_1q_{15}-$
$16q_0q_2q_{14}-16q_0q_3q_{13}-32q_1q_2q_{13}-32q_1q_3q_{12}-32q_1q_7q_8-32q_2q_3q_{11}-32q_2q_6q_8-16q_3q_5q_8-32q_3q_6q_7-$
$32q_4q_5q_7 - 32q_0q_1q_{16} - 16q_0q_2q_{15} - 16q_0q_3q_{14} - 32q_1q_2q_{14} - 32q_1q_3q_{13} - 32q_2q_3q_{12} - 32q_2q_7q_8 -$
$64q_4q_6q_7 - 32q_0q_1q_{17} - 32q_0q_2q_{16} - 16q_0q_3q_{15} - 32q_1q_2q_{15} - 16q_1q_3q_{14} - 32q_2q_3q_{13} - 32q_3q_7q_8 -$
$16q_4q_5q_9 + 96q_4q_6q_8 - 64q_5q_6q_7 - 32q_0q_1q_{18} - 32q_0q_2q_{17} - 16q_0q_3q_{16} - 32q_0q_9q_{10} - 32q_1q_2q_{16} -$
$16q_1q_3q_{15} - 32q_2q_3q_{14} - 16q_4q_5q_{10} - 64q_4q_7q_8 - 32q_5q_6q_8 - 32q_0q_1q_{19} - 32q_0q_2q_{18} - 32q_0q_3q_{17} -$
$32q_0q_9q_{11} - 32q_1q_2q_{17} - 32q_1q_3q_{16} - 32q_1q_9q_{10} - 32q_2q_3q_{15} - 16q_4q_5q_{11} - 16q_4q_6q_{10} - 32q_5q_6q_9 -$
$32q_5q_7q_8 - 16q_0q_1q_{20} - 16q_0q_2q_{19} - 16q_0q_3q_{18} - 16q_0q_9q_{12} - 32q_0q_{10}q_{11} - 32q_1q_2q_{18} - 32q_1q_3q_{17} -$
$32q_1q_9q_{11} - 32q_2q_3q_{16} - 32q_2q_9q_{10} - 16q_4q_5q_{12} + 16q_4q_7q_{10} + 16q_4q_8q_9 - 32q_5q_6q_{10} - 16q_5q_7q_9 -$
$16q_0q_2q_{20} - 16q_0q_3q_{19} - 16q_0q_9q_{13} - 32q_0q_{10}q_{12} - 32q_1q_2q_{19} - 32q_1q_3q_{18} - 32q_1q_9q_{12} - 32q_1q_{10}q_{11} -$
$32q_2q_3q_{17} - 32q_2q_9q_{11} - 32q_3q_9q_{10} - 16q_4q_5q_{13} + 16q_4q_8q_{10} - 32q_5q_6q_{11} - 32q_5q_7q_{10} - 16q_5q_8q_9 -$
$32q_6q_7q_9 - 16q_0q_3q_{20} + 16q_0q_9q_{14} - 16q_0q_{10}q_{13} - 32q_0q_{11}q_{12} - 16q_1q_2q_{20} - 16q_1q_3q_{19} + 16q_1q_9q_{13} -$
$32q_1q_{10}q_{12} - 32q_2q_3q_{18} - 32q_2q_{10}q_{11} - 16q_4q_5q_{14} - 16q_4q_6q_{13} + 16q_4q_8q_{11} - 32q_4q_9q_{10} - 32q_5q_6q_{12} -$
$32q_5q_7q_{11} - 16q_5q_8q_{10} - 32q_6q_7q_{10} - 16q_6q_8q_9 - 16q_0q_{10}q_{14} - 32q_0q_{11}q_{13} - 16q_1q_3q_{20} - 32q_1q_{10}q_{13} -$
$32q_1q_{11}q_{12} - 32q_2q_3q_{19} - 16q_2q_9q_{13} - 32q_2q_{10}q_{12} - 16q_3q_9q_{12} - 32q_3q_{10}q_{11} - 16q_4q_5q_{15} + 16q_4q_7q_{13} +$
$16q_4q_8q_{12} - 32q_5q_6q_{13} - 32q_5q_7q_{12} - 32q_5q_8q_{11} - 32q_5q_9q_{10} - 32q_6q_7q_{11} - 32q_6q_8q_{10} - 32q_7q_8q_9 -$
$16q_0q_{11}q_{14} - 32q_0q_{12}q_{13} + 16q_1q_{10}q_{14} - 32q_1q_{11}q_{13} - 16q_2q_3q_{20} - 32q_2q_{11}q_{12} - 16q_3q_9q_{13} - 16q_4q_5q_{16} +$
$16q_4q_8q_{13} - 32q_4q_{10}q_{11} - 32q_5q_6q_{14} - 32q_5q_7q_{13} - 32q_5q_8q_{12} - 32q_5q_9q_{11} - 32q_6q_7q_{12} - 32q_6q_8q_{11} -$
$32q_6q_9q_{10} - 32q_7q_8q_{10} - 32q_0q_{12}q_{14} - 32q_1q_{11}q_{14} - 32q_1q_{12}q_{13} - 16q_2q_{10}q_{14} - 32q_2q_{11}q_{13} - 16q_3q_9q_{14} -$
$16q_3q_{10}q_{13} - 32q_3q_{11}q_{12} - 32q_4q_5q_{17} - 16q_4q_6q_{16} + 16q_4q_8q_{14} - 32q_5q_6q_{15} - 16q_5q_7q_{14} - 16q_5q_8q_{13} -$
$16q_5q_9q_{12} - 32q_5q_{10}q_{11} - 32q_6q_7q_{13} - 32q_6q_8q_{12} - 32q_6q_9q_{11} - 32q_7q_8q_{11} - 32q_7q_9q_{10} - 32q_0q_{13}q_{14} -$
$32q_1q_{12}q_{14} - 32q_2q_{12}q_{13} - 16q_3q_{10}q_{14} - 16q_4q_5q_{18} + 16q_4q_7q_{16} + 16q_4q_8q_{15} - 32q_4q_{11}q_{12} - 32q_5q_6q_{16} -$
$16q_5q_7q_{15} - 16q_5q_8q_{14} + 16q_5q_9q_{13} - 32q_5q_{10}q_{12} - 32q_6q_7q_{14} - 32q_6q_8q_{13} - 32q_6q_{10}q_{11} - 32q_7q_8q_{12} -$
$32q_1q_{13}q_{14} - 32q_2q_{12}q_{14} - 16q_3q_{11}q_{14} - 32q_3q_{12}q_{13} - 16q_4q_5q_{19} - 16q_4q_6q_{18} + 16q_4q_8q_{16} - 32q_5q_6q_{17} -$
$32q_5q_7q_{16} - 16q_5q_8q_{15} + 16q_5q_9q_{14} - 16q_5q_{10}q_{13} - 32q_5q_{11}q_{12} - 32q_6q_7q_{15} - 16q_6q_8q_{14} + 16q_6q_9q_{13} -$
$32q_6q_{10}q_{12} - 32q_7q_8q_{13} - 32q_7q_{10}q_{11} - 32q_2q_{13}q_{14} + 16q_4q_7q_{18} + 32q_4q_8q_{17} - 32q_4q_{12}q_{13} - 32q_5q_6q_{18} -$
$32q_5q_7q_{17} - 16q_5q_8q_{16} + 16q_5q_{10}q_{14} - 32q_5q_{11}q_{13} - 32q_6q_7q_{16} - 16q_6q_8q_{15} - 32q_6q_{11}q_{12} - 32q_7q_8q_{14} -$
$16q_7q_9q_{13} - 16q_8q_9q_{12} - 32q_3q_{13}q_{14} + 16q_4q_8q_{18} - 32q_5q_6q_{19} - 32q_5q_7q_{18} - 32q_5q_8q_{17} - 16q_5q_{11}q_{14} -$
$32q_5q_{12}q_{13} - 32q_6q_7q_{17} - 32q_6q_8q_{16} + 16q_6q_{10}q_{14} - 32q_6q_{11}q_{13} - 32q_7q_8q_{15} - 32q_7q_{11}q_{12} - 16q_8q_9q_{13} -$
$64q_9q_{10}q_{11} - 32q_0q_{15}q_{16} + 16q_4q_8q_{19} - 32q_4q_{13}q_{14} - 16q_5q_6q_{20} - 16q_5q_7q_{19} - 16q_5q_8q_{18} - 32q_5q_{12}q_{14} -$
$32q_6q_7q_{18} - 32q_6q_8q_{17} - 32q_6q_{12}q_{13} - 32q_7q_8q_{16} - 16q_7q_{10}q_{14} - 16q_8q_9q_{14} - 16q_8q_{10}q_{13} - 64q_9q_{10}q_{12} -$
$32q_0q_{15}q_{17} - 32q_1q_{15}q_{16} - 16q_5q_7q_{20} - 16q_5q_8q_{19} - 32q_5q_{13}q_{14} - 32q_6q_7q_{19} - 32q_6q_8q_{18} - 32q_6q_{12}q_{14} -$
$32q_7q_8q_{17} - 32q_7q_{12}q_{13} - 16q_8q_{10}q_{14} - 64q_9q_{10}q_{13} - 96q_9q_{11}q_{12} - 16q_0q_{15}q_{18} - 32q_0q_{16}q_{17} - 32q_1q_{15}q_{17} -$
$32q_2q_{15}q_{16} - 16q_5q_8q_{20} - 16q_6q_7q_{20} - 16q_6q_8q_{19} - 32q_6q_{13}q_{14} - 32q_7q_8q_{18} - 16q_8q_{11}q_{14} - 64q_9q_{10}q_{14} -$
$32q_9q_{11}q_{13} - 64q_{10}q_{11}q_{12} - 16q_0q_{15}q_{19} - 32q_0q_{16}q_{18} - 32q_1q_{15}q_{18} - 32q_1q_{16}q_{17} - 32q_2q_{15}q_{17} - 32q_3q_{15}q_{16} -$
$16q_6q_8q_{20} - 32q_7q_8q_{19} - 32q_7q_{13}q_{14} - 32q_9q_{10}q_{15} - 32q_9q_{11}q_{14} - 64q_9q_{12}q_{13} - 96q_{10}q_{11}q_{13} + 16q_0q_{15}q_{20} -$
$16q_0q_{16}q_{19} - 32q_0q_{17}q_{18} + 16q_1q_{15}q_{19} - 32q_1q_{16}q_{18} - 32q_2q_{16}q_{17} - 32q_4q_{15}q_{16} - 16q_7q_8q_{20} - 32q_9q_{10}q_{16} -$

$16q_9q_{11}q_{15} - 32q_9q_{12}q_{14} - 64q_{10}q_{11}q_{14} - 96q_{10}q_{12}q_{13} - 16q_0q_{16}q_{20} - 32q_0q_{17}q_{19} - 32q_1q_{16}q_{19} - 32q_1q_{17}q_{18} -$
$16q_2q_{15}q_{19} - 32q_2q_{16}q_{18} - 16q_3q_{15}q_{18} - 32q_3q_{16}q_{17} - 32q_5q_{15}q_{16} - 32q_9q_{10}q_{17} - 32q_9q_{11}q_{16} - 16q_9q_{12}q_{15} -$
$64q_9q_{13}q_{14} - 32q_{10}q_{11}q_{15} - 32q_{10}q_{12}q_{14} - 64q_{11}q_{12}q_{13} - 16q_0q_{17}q_{20} - 32q_0q_{18}q_{19} + 16q_1q_{16}q_{20} -$
$32q_1q_{17}q_{19} - 32q_2q_{17}q_{18} - 16q_3q_{15}q_{19} - 32q_4q_{16}q_{17} - 32q_5q_{15}q_{17} - 32q_6q_{15}q_{16} - 32q_9q_{10}q_{18} - 32q_9q_{11}q_{17} -$
$16q_9q_{12}q_{16} - 16q_9q_{13}q_{15} - 32q_{10}q_{11}q_{16} - 16q_{10}q_{12}q_{15} - 64q_{10}q_{13}q_{14} - 96q_{11}q_{12}q_{14} - 32q_0q_{18}q_{20} -$
$32q_1q_{17}q_{20} - 32q_1q_{18}q_{19} - 16q_2q_{16}q_{20} - 32q_2q_{17}q_{19} - 16q_3q_{15}q_{20} - 16q_3q_{16}q_{19} - 32q_3q_{17}q_{18} - 16q_5q_{15}q_{18} -$
$32q_5q_{16}q_{17} - 32q_6q_{15}q_{17} - 32q_7q_{15}q_{16} - 32q_9q_{10}q_{19} - 32q_9q_{11}q_{18} - 32q_9q_{12}q_{17} - 16q_9q_{13}q_{16} - 16q_9q_{14}q_{15} -$
$32q_{10}q_{11}q_{17} - 32q_{10}q_{12}q_{16} - 16q_{10}q_{13}q_{15} - 32q_{11}q_{12}q_{15} - 64q_{11}q_{13}q_{14} - 32q_0q_{19}q_{20} - 32q_1q_{18}q_{20} -$
$32q_2q_{18}q_{19} - 16q_3q_{16}q_{20} - 32q_4q_{17}q_{18} + 16q_5q_{15}q_{19} - 32q_5q_{16}q_{18} - 32q_6q_{16}q_{17} - 16q_9q_{10}q_{20} - 16q_9q_{11}q_{19} -$
$16q_9q_{12}q_{18} - 32q_{10}q_{11}q_{18} - 32q_{10}q_{12}q_{17} - 16q_{10}q_{13}q_{16} - 16q_{10}q_{14}q_{15} - 32q_{11}q_{12}q_{16} - 16q_{11}q_{13}q_{15} -$
$64q_{12}q_{13}q_{14} - 32q_1q_{19}q_{20} - 32q_2q_{18}q_{20} - 16q_3q_{17}q_{20} - 32q_3q_{18}q_{19} + 16q_5q_{15}q_{20} - 16q_5q_{16}q_{19} - 32q_5q_{17}q_{18} +$
$16q_6q_{15}q_{19} - 32q_6q_{16}q_{18} - 32q_7q_{16}q_{17} - 16q_9q_{11}q_{20} - 16q_9q_{12}q_{19} - 16q_9q_{13}q_{18} - 32q_9q_{15}q_{16} - 32q_{10}q_{11}q_{19} -$
$32q_{10}q_{12}q_{18} - 32q_{10}q_{13}q_{17} - 16q_{10}q_{14}q_{16} - 32q_{11}q_{12}q_{17} - 32q_{11}q_{13}q_{16} - 16q_{11}q_{14}q_{15} - 32q_{12}q_{13}q_{15} -$
$32q_2q_{19}q_{20} - 32q_4q_{18}q_{19} + 16q_5q_{16}q_{20} - 32q_5q_{17}q_{19} - 32q_6q_{17}q_{18} - 16q_7q_{15}q_{19} - 16q_8q_{15}q_{18} - 16q_9q_{12}q_{20} -$
$16q_9q_{13}q_{19} - 16q_9q_{15}q_{17} - 16q_{10}q_{11}q_{20} - 16q_{10}q_{12}q_{19} - 16q_{10}q_{13}q_{18} - 32q_{10}q_{15}q_{16} - 32q_{11}q_{12}q_{18} -$
$32q_{11}q_{13}q_{17} - 16q_{11}q_{14}q_{16} - 32q_{12}q_{13}q_{16} - 16q_{12}q_{14}q_{15} - 32q_3q_{19}q_{20} - 16q_5q_{17}q_{20} - 32q_5q_{18}q_{19} +$
$16q_6q_{16}q_{20} - 32q_6q_{17}q_{19} - 32q_7q_{17}q_{18} - 16q_8q_{15}q_{19} - 16q_9q_{13}q_{20} - 16q_9q_{14}q_{19} - 16q_9q_{15}q_{18} - 32q_9q_{16}q_{17} -$
$16q_{10}q_{12}q_{20} - 16q_{10}q_{13}q_{19} - 16q_{10}q_{14}q_{18} - 32q_{10}q_{15}q_{17} - 32q_{11}q_{12}q_{19} - 32q_{11}q_{13}q_{18} - 32q_{11}q_{14}q_{17} -$
$32q_{11}q_{15}q_{16} - 32q_{12}q_{13}q_{17} - 32q_{12}q_{14}q_{16} - 32q_{13}q_{14}q_{15} - 32q_4q_{19}q_{20} - 32q_5q_{18}q_{20} - 32q_6q_{18}q_{19} -$
$16q_7q_{16}q_{20} - 16q_8q_{15}q_{20} - 16q_8q_{16}q_{19} - 16q_9q_{14}q_{20} - 16q_9q_{15}q_{19} - 16q_9q_{16}q_{18} - 16q_{10}q_{13}q_{20} - 16q_{10}q_{14}q_{19} -$
$16q_{10}q_{15}q_{18} - 32q_{10}q_{16}q_{17} - 16q_{11}q_{12}q_{20} - 16q_{11}q_{13}q_{19} - 16q_{11}q_{14}q_{18} - 32q_{11}q_{15}q_{17} - 32q_{12}q_{13}q_{18} -$
$32q_{12}q_{14}q_{17} - 32q_{12}q_{15}q_{16} - 32q_{13}q_{14}q_{16} - 32q_5q_{19}q_{20} - 32q_6q_{18}q_{20} - 32q_7q_{18}q_{19} - 16q_8q_{16}q_{20} -$
$16q_9q_{15}q_{20} - 16q_9q_{16}q_{19} - 32q_9q_{17}q_{18} - 16q_{10}q_{14}q_{20} - 16q_{10}q_{15}q_{19} - 32q_{10}q_{16}q_{18} - 16q_{11}q_{13}q_{20} -$
$16q_{11}q_{14}q_{19} - 32q_{11}q_{15}q_{18} - 32q_{11}q_{16}q_{17} - 32q_{12}q_{13}q_{19} - 32q_{12}q_{14}q_{18} - 32q_{12}q_{15}q_{17} - 32q_{13}q_{14}q_{17} -$
$32q_{13}q_{15}q_{16} - 32q_6q_{19}q_{20} - 16q_8q_{17}q_{20} - 16q_9q_{16}q_{20} - 16q_9q_{17}q_{19} - 16q_{10}q_{15}q_{20} - 16q_{10}q_{16}q_{19} -$
$32q_{10}q_{17}q_{18} - 16q_{11}q_{14}q_{20} - 16q_{11}q_{15}q_{19} - 32q_{11}q_{16}q_{18} - 16q_{12}q_{13}q_{20} - 16q_{12}q_{14}q_{19} - 32q_{12}q_{15}q_{18} -$
$32q_{12}q_{16}q_{17} - 32q_{13}q_{14}q_{18} - 32q_{13}q_{15}q_{17} - 32q_{14}q_{15}q_{16} - 32q_7q_{19}q_{20} - 16q_9q_{17}q_{20} - 32q_9q_{18}q_{19} -$
$16q_{10}q_{16}q_{20} - 32q_{10}q_{17}q_{19} - 32q_{11}q_{16}q_{19} - 32q_{11}q_{17}q_{18} - 16q_{12}q_{14}q_{20} - 16q_{12}q_{15}q_{19} - 32q_{12}q_{16}q_{18} -$
$32q_{13}q_{14}q_{19} - 16q_{13}q_{15}q_{18} - 32q_{13}q_{16}q_{17} - 16q_{14}q_{15}q_{17} - 16q_9q_{18}q_{20} - 16q_{10}q_{17}q_{20} - 32q_{10}q_{18}q_{19} -$
$16q_{11}q_{16}q_{20} - 32q_{11}q_{17}q_{19} - 32q_{12}q_{16}q_{19} - 32q_{12}q_{17}q_{18} - 16q_{13}q_{14}q_{20} - 16q_{13}q_{15}q_{19} - 32q_{13}q_{16}q_{18} -$
$16q_{14}q_{15}q_{18} - 32q_{14}q_{16}q_{17} - 32q_9q_{19}q_{20} - 32q_{10}q_{18}q_{20} - 32q_{11}q_{17}q_{20} - 32q_{11}q_{18}q_{19} - 16q_{12}q_{16}q_{20} -$
$32q_{12}q_{17}q_{19} - 16q_{13}q_{15}q_{20} - 16q_{13}q_{16}q_{19} - 32q_{13}q_{17}q_{18} - 16q_{14}q_{15}q_{19} - 16q_{14}q_{16}q_{18} - 64q_{15}q_{16}q_{17} -$
$32q_{10}q_{19}q_{20} - 32q_{11}q_{18}q_{20} - 32q_{12}q_{17}q_{20} - 32q_{12}q_{18}q_{19} - 16q_{13}q_{16}q_{20} - 32q_{13}q_{17}q_{19} - 16q_{14}q_{15}q_{20} -$
$16q_{14}q_{16}q_{19} - 32q_{14}q_{17}q_{18} - 64q_{15}q_{16}q_{18} - 32q_{11}q_{19}q_{20} - 32q_{12}q_{18}q_{20} - 16q_{13}q_{17}q_{20} - 32q_{13}q_{18}q_{19} -$
$16q_{14}q_{16}q_{20} - 16q_{14}q_{17}q_{19} - 64q_{15}q_{16}q_{19} - 96q_{15}q_{17}q_{18} - 32q_{12}q_{19}q_{20} - 32q_{13}q_{18}q_{20} - 16q_{14}q_{17}q_{20} -$
$32q_{14}q_{18}q_{19} - 32q_{15}q_{16}q_{20} - 64q_{16}q_{17}q_{18} - 32q_{13}q_{19}q_{20} - 16q_{14}q_{18}q_{20} - 32q_{15}q_{17}q_{20} - 64q_{15}q_{18}q_{19} -$
$96q_{16}q_{17}q_{19} - 32q_{14}q_{19}q_{20} - 32q_{15}q_{18}q_{20} - 32q_{16}q_{17}q_{20} - 64q_{16}q_{18}q_{19} - 64q_{15}q_{19}q_{20} - 32q_{16}q_{18}q_{20} -$
$64q_{17}q_{18}q_{19} - 64q_{16}q_{19}q_{20} - 64q_{17}q_{18}q_{20} - 64q_{17}q_{19}q_{20} - 32q_{18}q_{19}q_{20} + 64q_0q_1q_2q_3 + 32q_0q_1q_4q_5 +$
$32q_0q_1q_5q_6 + 32q_0q_2q_4q_6 + 32q_1q_2q_4q_5 - 32q_0q_1q_4q_8 - 32q_0q_2q_4q_7 - 32q_0q_3q_4q_6 + 32q_0q_1q_6q_7 +$
$32q_0q_2q_5q_7 + 32q_0q_3q_4q_7 + 32q_1q_2q_5q_6 + 32q_1q_3q_4q_6 + 32q_2q_3q_4q_5 - 32q_1q_2q_4q_8 - 32q_1q_3q_4q_7 +$
$32q_0q_1q_7q_8 + 32q_0q_2q_6q_8 + 32q_0q_3q_5q_8 + 32q_1q_2q_6q_7 + 32q_1q_3q_5q_7 + 32q_2q_3q_5q_6 - 32q_2q_3q_4q_8 +$
$32q_1q_2q_7q_8 + 32q_1q_3q_6q_8 + 32q_2q_3q_6q_7 + 32q_0q_1q_9q_{10} + 32q_2q_3q_7q_8 + 32q_0q_1q_{10}q_{11} + 32q_0q_2q_9q_{11} +$
$32q_1q_2q_9q_{10} + 64q_4q_5q_6q_7 - 64q_4q_5q_6q_8 + 32q_0q_1q_{11}q_{12} + 32q_0q_2q_{10}q_{12} + 32q_0q_3q_9q_{12} + 32q_1q_2q_{10}q_{11} +$
$32q_1q_3q_9q_{11} + 32q_2q_3q_9q_{10} + 64q_4q_5q_7q_8 - 64q_4q_6q_7q_8 + 32q_0q_1q_{12}q_{13} + 32q_0q_2q_{11}q_{13} + 32q_0q_3q_{10}q_{13} +$
$32q_1q_2q_{11}q_{12} + 32q_1q_3q_{10}q_{12} + 32q_2q_3q_{10}q_{11} + 64q_5q_6q_7q_8 + 32q_0q_1q_{13}q_{14} + 32q_0q_2q_{12}q_{14} + 32q_0q_3q_{11}q_{14} +$
$32q_1q_2q_{12}q_{13} + 32q_1q_3q_{11}q_{13} + 32q_2q_3q_{11}q_{12} + 32q_4q_5q_9q_{10} + 32q_1q_2q_{13}q_{14} + 32q_1q_3q_{12}q_{14} + 32q_2q_3q_{12}q_{13} +$
$32q_4q_5q_{10}q_{11} + 32q_4q_6q_9q_{11} + 32q_5q_6q_9q_{10} - 32q_4q_5q_9q_{13} - 32q_4q_6q_9q_{12} - 32q_4q_7q_9q_{11} - 32q_4q_8q_9q_{10} +$
$32q_0q_1q_{15}q_{16} + 32q_2q_3q_{13}q_{14} + 32q_4q_5q_{11}q_{12} + 32q_4q_6q_{10}q_{12} + 32q_4q_7q_9q_{12} + 32q_5q_6q_{10}q_{11} + 32q_5q_7q_9q_{11} +$

$32q_6q_7q_9q_{10}-32q_4q_5q_{10}q_{14}-32q_4q_6q_{10}q_{13}-32q_4q_7q_{10}q_{12}-32q_4q_8q_{10}q_{11}+32q_0q_1q_{16}q_{17}+32q_0q_2q_{15}q_{17}+$
$32q_1q_2q_{15}q_{16}+32q_4q_5q_{12}q_{13}+32q_4q_6q_{11}q_{13}+32q_4q_7q_{10}q_{13}+32q_4q_8q_9q_{13}+32q_5q_6q_{11}q_{12}+32q_5q_7q_{10}q_{12}+$
$32q_5q_8q_9q_{12}+32q_6q_7q_{10}q_{11}+32q_6q_8q_9q_{11}+32q_7q_8q_9q_{10}-32q_4q_6q_{11}q_{14}-32q_4q_7q_{11}q_{13}-32q_4q_8q_{11}q_{12}+$
$32q_0q_1q_{17}q_{18}+32q_0q_2q_{16}q_{18}+32q_0q_3q_{15}q_{18}+32q_1q_2q_{16}q_{17}+32q_1q_3q_{15}q_{17}+32q_2q_3q_{15}q_{16}+32q_4q_5q_{13}q_{14}+$
$32q_4q_6q_{12}q_{14}+32q_4q_7q_{11}q_{14}+32q_4q_8q_{10}q_{14}+32q_5q_6q_{12}q_{13}+32q_5q_7q_{11}q_{13}+32q_5q_8q_{10}q_{13}+32q_6q_7q_{11}q_{12}+$
$32q_6q_8q_{10}q_{12}+32q_7q_8q_{10}q_{11}-32q_4q_7q_{12}q_{14}-32q_4q_8q_{12}q_{13}+32q_0q_1q_{18}q_{19}+32q_0q_2q_{17}q_{19}+32q_0q_3q_{16}q_{19}+$
$32q_1q_2q_{17}q_{18}+32q_1q_3q_{16}q_{18}+32q_2q_3q_{16}q_{17}+32q_5q_6q_{13}q_{14}+32q_5q_7q_{12}q_{14}+32q_5q_8q_{11}q_{14}+32q_6q_7q_{12}q_{13}+$
$32q_6q_8q_{11}q_{13}+32q_7q_8q_{11}q_{12}-32q_4q_8q_{13}q_{14}+32q_0q_1q_{19}q_{20}+32q_0q_2q_{18}q_{20}+32q_0q_3q_{17}q_{20}+32q_1q_2q_{18}q_{19}+$
$32q_1q_3q_{17}q_{19}+32q_2q_3q_{17}q_{18}+32q_4q_5q_{15}q_{16}+32q_6q_7q_{13}q_{14}+32q_6q_8q_{12}q_{14}+32q_7q_8q_{12}q_{13}+32q_1q_2q_{19}q_{20}+$
$32q_1q_3q_{18}q_{20}+32q_2q_3q_{18}q_{19}+32q_4q_5q_{16}q_{17}+32q_4q_6q_{15}q_{17}+32q_5q_6q_{15}q_{16}+32q_7q_8q_{13}q_{14}+64q_9q_{10}q_{11}q_{12}-$
$32q_4q_5q_{15}q_{19}-32q_4q_6q_{15}q_{18}-32q_4q_7q_{15}q_{17}-32q_4q_8q_{15}q_{16}+32q_2q_3q_{19}q_{20}+32q_4q_5q_{17}q_{18}+32q_4q_6q_{16}q_{18}+$
$32q_4q_7q_{15}q_{18}+32q_5q_6q_{16}q_{17}+32q_5q_7q_{15}q_{17}+32q_6q_7q_{15}q_{16}+64q_9q_{10}q_{12}q_{13}-32q_4q_5q_{16}q_{20}-32q_4q_6q_{16}q_{19}-$
$32q_4q_7q_{16}q_{18}-32q_4q_8q_{16}q_{17}+32q_4q_5q_{18}q_{19}+32q_4q_6q_{17}q_{19}+32q_4q_7q_{16}q_{19}+32q_4q_8q_{15}q_{19}+32q_5q_6q_{17}q_{18}+$
$32q_5q_7q_{16}q_{18}+32q_5q_8q_{15}q_{18}+32q_6q_7q_{16}q_{17}+32q_6q_8q_{15}q_{17}+32q_7q_8q_{15}q_{16}+64q_9q_{10}q_{13}q_{14}+64q_9q_{11}q_{12}q_{14}+$
$64q_{10}q_{11}q_{12}q_{13}-32q_4q_6q_{17}q_{20}-32q_4q_7q_{17}q_{19}-32q_4q_8q_{17}q_{18}+32q_4q_5q_{19}q_{20}+32q_4q_6q_{18}q_{20}+32q_4q_7q_{17}q_{20}+$
$32q_4q_8q_{16}q_{20}+32q_5q_6q_{18}q_{19}+32q_5q_7q_{17}q_{19}+32q_5q_8q_{16}q_{19}+32q_6q_7q_{17}q_{18}+32q_6q_8q_{16}q_{18}+32q_7q_8q_{16}q_{17}+$
$64q_{10}q_{11}q_{13}q_{14}-32q_4q_7q_{18}q_{20}-32q_4q_8q_{18}q_{19}+32q_5q_6q_{19}q_{20}+32q_5q_7q_{18}q_{20}+32q_5q_8q_{17}q_{20}+32q_6q_7q_{18}q_{19}+$
$32q_6q_8q_{17}q_{19}+32q_7q_8q_{17}q_{18}+32q_9q_{10}q_{15}q_{16}+64q_{11}q_{12}q_{13}q_{14}-32q_4q_8q_{19}q_{20}+32q_6q_7q_{19}q_{20}+32q_6q_8q_{18}q_{20}+$
$32q_7q_8q_{18}q_{19} + 32q_9q_{10}q_{16}q_{17} + 32q_9q_{11}q_{15}q_{17} + 32q_{10}q_{11}q_{15}q_{16} + 32q_7q_8q_{19}q_{20} + 32q_9q_{10}q_{17}q_{18} +$
$32q_9q_{11}q_{16}q_{18} + 32q_9q_{12}q_{15}q_{18} + 32q_{10}q_{11}q_{16}q_{17} + 32q_{10}q_{12}q_{15}q_{17} + 32q_{11}q_{12}q_{15}q_{16} + 32q_9q_{10}q_{18}q_{19} +$
$32q_9q_{11}q_{17}q_{19} + 32q_9q_{12}q_{16}q_{19} + 32q_9q_{13}q_{15}q_{19} + 32q_{10}q_{11}q_{17}q_{18} + 32q_{10}q_{12}q_{16}q_{18} + 32q_{10}q_{13}q_{15}q_{18} +$
$32q_{11}q_{12}q_{16}q_{17} + 32q_{11}q_{13}q_{15}q_{17} + 32q_{12}q_{13}q_{15}q_{16} + 32q_9q_{10}q_{19}q_{20} + 32q_9q_{11}q_{18}q_{20} + 32q_9q_{12}q_{17}q_{20} +$
$32q_9q_{13}q_{16}q_{20} + 32q_9q_{14}q_{15}q_{20} + 32q_{10}q_{11}q_{18}q_{19} + 32q_{10}q_{12}q_{17}q_{19} + 32q_{10}q_{13}q_{16}q_{19} + 32q_{10}q_{14}q_{15}q_{19} +$
$32q_{11}q_{12}q_{17}q_{18} + 32q_{11}q_{13}q_{16}q_{18} + 32q_{11}q_{14}q_{15}q_{18} + 32q_{12}q_{13}q_{16}q_{17} + 32q_{12}q_{14}q_{15}q_{17} + 32q_{13}q_{14}q_{15}q_{16} +$
$32q_{10}q_{11}q_{19}q_{20} + 32q_{10}q_{12}q_{18}q_{20} + 32q_{10}q_{13}q_{17}q_{20} + 32q_{10}q_{14}q_{16}q_{20} + 32q_{11}q_{12}q_{18}q_{19} + 32q_{11}q_{13}q_{17}q_{19} +$
$32q_{11}q_{14}q_{16}q_{19} + 32q_{12}q_{13}q_{17}q_{18} + 32q_{12}q_{14}q_{16}q_{18} + 32q_{13}q_{14}q_{16}q_{17} + 32q_{11}q_{12}q_{19}q_{20} + 32q_{11}q_{13}q_{18}q_{20} +$
$32q_{11}q_{14}q_{17}q_{20} + 32q_{12}q_{13}q_{18}q_{19} + 32q_{12}q_{14}q_{17}q_{19} + 32q_{13}q_{14}q_{17}q_{18} + 32q_{12}q_{13}q_{19}q_{20} + 32q_{12}q_{14}q_{18}q_{20} +$
$32q_{13}q_{14}q_{18}q_{19} + 32q_{13}q_{14}q_{19}q_{20} + 64q_{15}q_{16}q_{17}q_{18} + 64q_{15}q_{16}q_{18}q_{19} + 64q_{15}q_{16}q_{19}q_{20} + 64q_{15}q_{17}q_{18}q_{20} +$
$64q_{16}q_{17}q_{18}q_{19} + 64q_{16}q_{17}q_{19}q_{20} + 64q_{17}q_{18}q_{19}q_{20} + 2056$

A complete expression of $E_2(q)$ in 92-order H matrix using Turyn based method with $\delta = 103,328$

$E_2(q) = 310192q_{21} - 336q_1 - 320q_2 - 264q_3 - 144q_4 - 272q_5 - 248q_6 - 200q_7 - 96q_8 - 328q_9 -$
$384q_{10} - 408q_{11} - 408q_{12} - 384q_{13} - 328q_{14} - 328q_{15} - 368q_{16} - 376q_{17} - 368q_{18} - 328q_{19} - 248q_{20} -$
$344q_0 + 310144q_{22} + 310112q_{23} + 310016q_{24} + 310056q_{25} + 310040q_{26} + 310024q_{27} + 310040q_{28} +$
$310056q_{29} + 310072q_{30} + 310072q_{31} + 310056q_{32} + 310040q_{33} + 310064q_{34} + 310064q_{35} + 310064q_{36} +$
$310040q_{37} + 310176q_{38} + 310144q_{39} + 310016q_{40} + 310072q_{41} + 310056q_{42} + 310008q_{43} + 310040q_{44} +$
$310056q_{45} + 310072q_{46} + 310072q_{47} + 310056q_{48} + 310040q_{49} + 310056q_{50} + 310080q_{51} + 310056q_{52} +$
$310040q_{53} + 310160q_{54} + 310016q_{55} + 310040q_{56} + 310040q_{57} + 310024q_{58} + 310040q_{59} + 310056q_{60} +$
$310056q_{61} + 310056q_{62} + 310056q_{63} + 310040q_{64} + 310056q_{65} + 310048q_{66} + 310056q_{67} + 310040q_{68} +$
$310016q_{69} + 310008q_{70} + 310008q_{71} + 310024q_{72} + 310040q_{73} + 310040q_{74} + 310040q_{75} + 310040q_{76} +$
$310040q_{77} + 310040q_{78} + 310032q_{79} + 310032q_{80} + 310032q_{81} + 310040q_{82} + 310080q_{83} + 310048q_{84} +$
$310016q_{85} + 309920q_{86} + 310000q_{87} + 310016q_{88} + 310016q_{89} + 310016q_{90} + 310016q_{91} + 310016q_{92} +$
$310016q_{93} + 310016q_{94} + 310016q_{95} + 310016q_{96} + 310192q_{97} + 310128q_{98} + 310064q_{99} + 310024q_{100} +$
$310040q_{101}+310056q_{102}+310056q_{103}+310040q_{104}+310024q_{105}+310048q_{106}+310064q_{107}+310048q_{108}+$
$310024q_{109}+310144q_{110}+310096q_{111}+310024q_{112}+310040q_{113}+310040q_{114}+310040q_{115}+310040q_{116}+$
$310024q_{117}+310040q_{118}+310048q_{119}+310040q_{120}+310024q_{121}+310160q_{122}+310024q_{123}+310024q_{124}+$
$310024q_{125}+310024q_{126}+310024q_{127}+310024q_{128}+310024q_{129}+310016q_{130}+310024q_{131}+310024q_{132}+$

$310008q_{133}+310008q_{134}+310008q_{135}+310008q_{136}+310000q_{137}+310000q_{138}+310192q_{139}+310160q_{140}+$
$310128q_{141}+310112q_{142}+310080q_{143}+310048q_{144}+310048q_{145}+310048q_{146}+310048q_{147}+310048q_{148}+$
$310208q_{149}+310160q_{150}+310160q_{151}+310112q_{152}+310056q_{153}+310064q_{154}+310064q_{155}+310064q_{156}+$
$310056q_{157}+310224q_{158}+310160q_{159}+310128q_{160}+310056q_{161}+310072q_{162}+310080q_{163}+310072q_{164}+$
$310056q_{165}+310208q_{166}+310160q_{167}+310056q_{168}+310072q_{169}+310080q_{170}+310072q_{171}+310056q_{172}+$
$310192q_{173}+310056q_{174}+310064q_{175}+310064q_{176}+310064q_{177}+310056q_{178}+310048q_{179}+310048q_{180}+$
$310048q_{181}+310048q_{182}+310048q_{183}+310184q_{184}+310152q_{185}+310136q_{186}+310104q_{187}+310056q_{188}+$
$310200q_{189}+310152q_{190}+310136q_{191}+310088q_{192}+310200q_{193}+310152q_{194}+310104q_{195}+310184q_{196}+$
$310136q_{197} + 310184q_{198} + 103328q_0q_1 + 103328q_0q_2 + 103328q_0q_3 + 103328q_1q_2 + 103328q_0q_4 +$
$103328q_1q_3 + 103328q_0q_5 + 103328q_1q_4 + 103328q_2q_3 + 103328q_0q_6 + 103328q_1q_5 + 103328q_2q_4 +$
$103328q_0q_7+103328q_1q_6+103328q_2q_5+103328q_3q_4+8q_0q_8+103328q_1q_7+103328q_2q_6+103328q_3q_5+$
$103328q_0q_9 + 8q_1q_8 + 103328q_2q_7 + 103328q_3q_6 + 103328q_4q_5 + 103328q_0q_{10} + 103328q_1q_9 + 8q_2q_8 +$
$103328q_3q_7+103328q_4q_6+103328q_0q_{11}+103328q_1q_{10}+103328q_2q_9+24q_3q_8+103328q_4q_7+103328q_5q_6+$
$103328q_0q_{12} + 103328q_1q_{11} + 103328q_2q_{10} + 103328q_3q_9 + 103328q_4q_8 + 103328q_5q_7 + 103328q_0q_{13} +$
$103328q_1q_{12} + 103328q_2q_{11} + 103328q_3q_{10} + 103328q_4q_9 + 103328q_5q_8 + 103328q_6q_7 + 56q_0q_{14} +$
$103328q_1q_{13} + 103328q_2q_{12} + 103328q_3q_{11} + 103328q_4q_{10} + 103328q_5q_9 + 103328q_6q_8 + 103328q_0q_{15} +$
$56q_1q_{14} + 103328q_2q_{13} + 103328q_3q_{12} + 103328q_4q_{11} + 103328q_5q_{10} + 103328q_6q_9 + 103328q_7q_8 +$
$103328q_0q_{16} + 103328q_1q_{15} + 56q_2q_{14} + 103328q_3q_{13} + 103328q_4q_{12} + 103328q_5q_{11} + 103328q_6q_{10} +$
$103328q_7q_9 + 103328q_0q_{17} + 103328q_1q_{16} + 103328q_2q_{15} + 56q_3q_{14} + 103328q_4q_{13} + 103328q_5q_{12} +$
$103328q_6q_{11} + 103328q_7q_{10} + 103328q_8q_9 + 103328q_0q_{18} + 103328q_1q_{17} + 103328q_2q_{16} + 103328q_3q_{15} +$
$16q_4q_{14} + 103328q_5q_{13} + 103328q_6q_{12} + 103328q_7q_{11} + 103328q_8q_{10} + 103328q_0q_{19} + 103328q_1q_{18} +$
$103328q_2q_{17} + 103328q_3q_{16} + 103328q_4q_{15} + 40q_5q_{14} + 103328q_6q_{13} + 103328q_7q_{12} + 103328q_8q_{11} +$
$103328q_9q_{10} + 40q_0q_{20} + 103328q_1q_{19} + 103328q_2q_{18} + 103328q_3q_{17} + 103328q_4q_{16} + 103328q_5q_{15} +$
$40q_6q_{14} + 103328q_7q_{13} + 24q_8q_{12} + 103328q_9q_{11} - 206656q_0q_{21} + 40q_1q_{20} + 103328q_2q_{19} + 103328q_3q_{18} +$
$103328q_4q_{17} + 103328q_5q_{16} + 103328q_6q_{15} + 40q_7q_{14} + 24q_8q_{13} + 103328q_9q_{12} + 103328q_{10}q_{11} -$
$206656q_0q_{22} - 206656q_1q_{21} + 40q_2q_{20} + 103328q_3q_{19} + 103328q_4q_{18} + 103328q_5q_{17} + 103328q_6q_{16} +$
$103328q_7q_{15} + 24q_8q_{14} + 103328q_9q_{13} + 103328q_{10}q_{12} - 206656q_0q_{23} - 64q_2q_{21} + 40q_3q_{20} + 103328q_4q_{19} +$
$103328q_5q_{18} + 103328q_6q_{17} + 103328q_7q_{16} + 103328q_8q_{15} + 103328q_9q_{14} + 103328q_{10}q_{13} + 103328q_{11}q_{12} -$
$206656q_0q_{24} - 206656q_2q_{22} - 96q_3q_{21} + 103328q_5q_{19} + 103328q_6q_{18} + 103328q_7q_{17} + 103328q_8q_{16} +$
$103328q_9q_{15} + 103328q_{10}q_{14} + 103328q_{11}q_{13} - 206656q_0q_{25} - 32q_3q_{22} - 32q_4q_{21} + 24q_5q_{20} + 103328q_6q_{19} +$
$103328q_7q_{18} + 103328q_8q_{17} + 103328q_9q_{16} + 103328q_{10}q_{15} + 103328q_{11}q_{14} + 103328q_{12}q_{13} - 206656q_0q_{26} -$
$206656q_3q_{23} - 32q_5q_{21} + 24q_6q_{20} + 103328q_7q_{19} + 16q_8q_{18} + 103328q_9q_{17} + 103328q_{10}q_{16} + 103328q_{11}q_{15} +$
$103328q_{12}q_{14} - 206656q_0q_{27} - 32q_5q_{22} - 32q_6q_{21} + 24q_7q_{20} + 24q_8q_{19} + 103328q_9q_{18} + 103328q_{10}q_{17} +$
$103328q_{11}q_{16} + 103328q_{12}q_{15} + 103328q_{13}q_{14} - 206656q_0q_{28} - 206656q_4q_{24} - 16q_5q_{23} - 32q_6q_{22} -$
$32q_7q_{21} + 24q_8q_{20} + 103328q_9q_{19} + 103328q_{10}q_{18} + 103328q_{11}q_{17} + 103328q_{12}q_{16} + 103328q_{13}q_{15} -$
$206656q_0q_{29} - 16q_5q_{24} + 56q_9q_{20} + 103328q_{10}q_{19} + 103328q_{11}q_{18} + 103328q_{12}q_{17} + 103328q_{13}q_{16} +$
$103328q_{14}q_{15} - 206656q_0q_{30} - 206656q_5q_{25} - 16q_6q_{24} - 32q_9q_{21} + 56q_{10}q_{20} + 103328q_{11}q_{19} + 103328q_{12}q_{18} +$
$103328q_{13}q_{17} + 103328q_{14}q_{16} - 206656q_0q_{31} - 32q_6q_{25} + 16q_7q_{24} - 16q_8q_{23} - 16q_9q_{22} - 32q_{10}q_{21} +$
$56q_{11}q_{20} + 103328q_{12}q_{19} + 103328q_{13}q_{18} + 103328q_{14}q_{17} + 103328q_{15}q_{16} - 206656q_0q_{32} - 206656q_6q_{26} -$
$32q_7q_{25} + 16q_8q_{24} - 16q_9q_{23} - 32q_{10}q_{22} - 32q_{11}q_{21} + 56q_{12}q_{20} + 103328q_{13}q_{19} + 103328q_{14}q_{18} +$
$103328q_{15}q_{17} - 206656q_0q_{33} - 32q_7q_{26} - 16q_8q_{25} - 16q_{10}q_{23} - 32q_{11}q_{22} - 32q_{12}q_{21} + 56q_{13}q_{20} +$
$103328q_{14}q_{19} + 103328q_{15}q_{18} + 103328q_{16}q_{17} - 206656q_0q_{34} - 206656q_7q_{27} - 32q_8q_{26} - 32q_{11}q_{23} -$
$32q_{12}q_{22} - 32q_{13}q_{21} + 56q_{14}q_{20} + 103328q_{15}q_{19} + 103328q_{16}q_{18} - 206656q_0q_{35} - 32q_8q_{27} - 32q_{12}q_{23} -$
$32q_{13}q_{22} - 32q_{14}q_{21} + 103328q_{15}q_{20} + 103328q_{16}q_{19} + 103328q_{17}q_{18} - 206656q_0q_{36} - 16q_{13}q_{23} - 16q_{14}q_{22} -$
$32q_{15}q_{21} + 103328q_{16}q_{20} + 103328q_{17}q_{19} - 206656q_0q_{37} - 206656q_9q_{28} - 16q_{14}q_{23} - 16q_{15}q_{22} - 32q_{16}q_{21} +$
$103328q_{17}q_{20} + 103328q_{18}q_{19} - 32q_{10}q_{28} - 16q_{15}q_{23} - 32q_{16}q_{22} - 32q_{17}q_{21} + 103328q_{18}q_{20} - 206656q_1q_{38} -$
$206656q_{10}q_{29} - 32q_{11}q_{28} - 16q_{16}q_{23} - 32q_{17}q_{22} - 32q_{18}q_{21} + 103328q_{19}q_{20} - 206656q_1q_{39} - 206656q_2q_{38} -$
$32q_{11}q_{29} - 16q_{12}q_{28} - 32q_{17}q_{23} - 32q_{18}q_{22} - 32q_{19}q_{21} - 206656q_1q_{40} - 64q_3q_{38} - 206656q_{11}q_{30} - 32q_{12}q_{29} -$

$16q_{13}q_{28} - 16q_{18}q_{23} - 16q_{19}q_{22} - 16q_{20}q_{21} - 206656q_1q_{41} - 206656q_3q_{39} - 32q_4q_{38} - 32q_{12}q_{30} - 16q_{13}q_{29} +$
$16q_{14}q_{28} - 16q_{19}q_{23} - 16q_{20}q_{22} - 206656q_1q_{42} - 32q_5q_{38} - 206656q_{12}q_{31} - 32q_{13}q_{30} - 16q_{14}q_{29} - 16q_{20}q_{23} -$
$206656q_1q_{43} - 206656q_4q_{40} - 32q_5q_{39} - 32q_6q_{38} - 32q_{13}q_{31} - 16q_{14}q_{30} - 206656q_1q_{44} - 48q_5q_{40} -$
$32q_6q_{39} - 32q_7q_{38} - 206656q_{13}q_{32} - 32q_{14}q_{31} - 206656q_1q_{45} - 206656q_5q_{41} - 32q_{14}q_{32} - 206656q_1q_{46} -$
$32q_6q_{41} - 32q_9q_{38} - 206656q_1q_{47} - 206656q_6q_{42} - 32q_7q_{41} + 48q_8q_{40} - 16q_9q_{39} - 32q_{10}q_{38} - 206656q_{15}q_{33} -$
$206656q_1q_{48} - 32q_7q_{42} - 32q_8q_{41} - 32q_{10}q_{39} - 32q_{11}q_{38} - 32q_{16}q_{33} - 206656q_1q_{49} - 206656q_7q_{43} -$
$32q_8q_{42} - 32q_{11}q_{39} - 32q_{12}q_{38} - 206656q_{16}q_{34} - 32q_{17}q_{33} - 206656q_1q_{50} - 32q_8q_{43} - 32q_{12}q_{39} - 32q_{13}q_{38} -$
$32q_{17}q_{34} - 16q_{18}q_{33} - 206656q_1q_{51} - 32q_{13}q_{39} - 32q_{14}q_{38} - 206656q_{17}q_{35} - 32q_{18}q_{34} - 16q_{19}q_{33} -$
$206656q_1q_{52} - 206656q_9q_{44} - 16q_{14}q_{39} - 32q_{15}q_{38} - 32q_{18}q_{35} - 16q_{19}q_{34} + 16q_{20}q_{33} - 206656q_1q_{53} -$
$32q_{10}q_{44} - 16q_{15}q_{39} - 32q_{16}q_{38} - 206656q_{18}q_{36} - 32q_{19}q_{35} - 16q_{20}q_{34} - 206656q_{10}q_{45} - 32q_{11}q_{44} -$
$32q_{16}q_{39} - 32q_{17}q_{38} - 32q_{19}q_{36} - 16q_{20}q_{35} - 206656q_2q_{54} - 32q_{11}q_{45} - 32q_{12}q_{44} - 32q_{17}q_{39} - 32q_{18}q_{38} -$
$206656q_{19}q_{37} - 32q_{20}q_{36} - 206656q_2q_{55} - 206656q_3q_{54} - 206656q_{11}q_{46} - 32q_{12}q_{45} + 16q_{13}q_{44} - 32q_{18}q_{39} -$
$32q_{19}q_{38} - 32q_{20}q_{37} - 206656q_2q_{56} - 32q_4q_{54} - 32q_{12}q_{46} - 32q_{13}q_{45} - 16q_{19}q_{39} - 16q_{20}q_{38} - 206656q_2q_{57} -$
$206656q_4q_{55} - 32q_5q_{54} - 206656q_{12}q_{47} - 32q_{13}q_{46} + 16q_{14}q_{45} - 16q_{20}q_{39} - 206656q_2q_{58} - 16q_5q_{55} -$
$32q_6q_{54} - 32q_{13}q_{47} - 32q_{14}q_{46} - 206656q_2q_{59} - 206656q_5q_{56} - 32q_6q_{55} - 32q_7q_{54} - 206656q_{13}q_{48} -$
$32q_{14}q_{47} - 206656q_2q_{60} - 32q_6q_{56} + 32q_7q_{55} - 32q_{14}q_{48} - 206656q_2q_{61} - 206656q_6q_{57} - 32q_7q_{56} + 16q_8q_{55} -$
$32q_9q_{54} - 206656q_2q_{62} - 32q_7q_{57} - 32q_{10}q_{54} - 206656q_{15}q_{49} - 206656q_2q_{63} - 206656q_7q_{58} - 32q_8q_{57} -$
$32q_{11}q_{54} - 32q_{16}q_{49} - 206656q_2q_{64} - 32q_8q_{58} - 32q_{12}q_{54} - 206656q_{16}q_{50} - 32q_{17}q_{49} - 206656q_2q_{65} -$
$32q_{13}q_{54} - 32q_{17}q_{50} - 32q_{18}q_{49} - 206656q_2q_{66} - 206656q_9q_{59} - 32q_{14}q_{54} - 206656q_{17}q_{51} - 32q_{18}q_{50} +$
$16q_{19}q_{49} - 206656q_2q_{67} - 32q_{10}q_{59} - 32q_{15}q_{54} - 32q_{18}q_{51} - 32q_{19}q_{50} - 206656q_2q_{68} - 206656q_{10}q_{60} -$
$32q_{11}q_{59} - 32q_{16}q_{54} - 206656q_{18}q_{52} - 32q_{19}q_{51} + 16q_{20}q_{50} - 32q_{11}q_{60} - 32q_{17}q_{54} - 32q_{19}q_{52} - 32q_{20}q_{51} -$
$206656q_3q_{69} - 206656q_{11}q_{61} - 32q_{12}q_{60} - 16q_{13}q_{59} - 32q_{18}q_{54} - 206656q_{19}q_{53} - 32q_{20}q_{52} - 206656q_3q_{70} -$
$206656q_4q_{69} - 32q_{12}q_{61} - 32q_{19}q_{54} - 32q_{20}q_{53} - 206656q_3q_{71} - 16q_5q_{69} - 206656q_{12}q_{62} - 32q_{13}q_{61} -$
$16q_{14}q_{60} - 16q_{20}q_{54} - 206656q_3q_{72} - 206656q_5q_{70} + 16q_6q_{69} - 32q_{13}q_{62} + 64q_{21}q_{54} - 206656q_3q_{73} - 32q_6q_{70} -$
$16q_7q_{69} - 206656q_{13}q_{63} - 32q_{14}q_{62} - 206656q_3q_{74} - 206656q_6q_{71} + 16q_8q_{69} - 32q_{14}q_{63} - 206656q_3q_{75} -$
$32q_7q_{71} - 16q_8q_{70} - 206656q_3q_{76} - 206656q_7q_{72} - 206656q_{15}q_{64} - 206656q_3q_{77} - 32q_8q_{72} - 32q_{16}q_{64} -$
$206656q_3q_{78} - 206656q_{16}q_{65} - 32q_{17}q_{64} - 206656q_3q_{79} - 206656q_9q_{73} - 32q_{17}q_{65} - 206656q_3q_{80} -$
$32q_{10}q_{73} - 206656q_{17}q_{66} - 32q_{18}q_{65} - 16q_{19}q_{64} - 206656q_3q_{81} - 206656q_{10}q_{74} - 32q_{18}q_{66} - 206656q_3q_{82} -$
$32q_{11}q_{74} - 16q_{12}q_{73} - 206656q_{18}q_{67} - 32q_{19}q_{66} - 16q_{20}q_{65} - 206656q_{11}q_{75} - 16q_{13}q_{73} - 32q_{19}q_{67} -$
$206656q_4q_{83} - 32q_{12}q_{75} - 16q_{13}q_{74} - 16q_{14}q_{73} - 206656q_{19}q_{68} - 32q_{20}q_{67} - 206656q_4q_{84} - 206656q_5q_{83} -$
$206656q_{12}q_{76} - 16q_{14}q_{74} - 32q_{20}q_{68} - 206656q_4q_{85} - 64q_6q_{83} - 32q_{13}q_{76} - 16q_{14}q_{75} - 206656q_4q_{86} -$
$206656q_6q_{84} - 32q_7q_{83} - 206656q_{13}q_{77} - 206656q_4q_{87} - 64q_7q_{84} - 32q_{14}q_{77} - 206656q_4q_{88} - 206656q_7q_{85} +$
$96q_8q_{84} - 16q_9q_{83} - 206656q_4q_{89} - 64q_8q_{85} - 16q_{10}q_{83} - 206656q_{15}q_{78} - 206656q_4q_{90} - 206656q_8q_{86} -$
$16q_{10}q_{84} - 16q_{11}q_{83} - 32q_{16}q_{78} - 206656q_4q_{91} + 16q_9q_{86} + 16q_{10}q_{85} - 16q_{12}q_{83} - 206656q_{16}q_{79} -$
$206656q_4q_{92} - 206656q_9q_{87} + 16q_{10}q_{86} - 16q_{13}q_{83} - 32q_{17}q_{79} - 16q_{18}q_{78} - 206656q_4q_{93} - 32q_{10}q_{87} +$
$16q_{11}q_{86} - 16q_{13}q_{84} - 16q_{14}q_{83} - 206656q_{17}q_{80} - 16q_{19}q_{78} - 206656q_4q_{94} - 206656q_{10}q_{88} + 16q_{12}q_{86} +$
$16q_{13}q_{85} - 16q_{15}q_{83} - 32q_{18}q_{80} - 16q_{19}q_{79} - 16q_{20}q_{78} - 206656q_4q_{95} - 32q_{11}q_{88} + 16q_{13}q_{86} - 16q_{16}q_{83} -$
$206656q_{18}q_{81} - 16q_{20}q_{79} - 206656q_4q_{96} - 206656q_{11}q_{89} + 16q_{14}q_{86} - 16q_{16}q_{84} - 32q_{17}q_{83} - 32q_{19}q_{81} -$
$16q_{20}q_{80} - 32q_{12}q_{89} + 16q_{15}q_{86} + 16q_{16}q_{85} - 16q_{18}q_{83} - 206656q_{19}q_{82} - 206656q_5q_{97} - 206656q_{12}q_{90} +$
$16q_{16}q_{86} - 16q_{18}q_{84} - 16q_{19}q_{83} - 32q_{20}q_{82} - 206656q_5q_{98} - 206656q_6q_{97} - 32q_{13}q_{90} + 32q_{17}q_{86} + 16q_{18}q_{85} -$
$206656q_5q_{99} - 64q_7q_{97} - 206656q_{13}q_{91} + 16q_{18}q_{86} + 32q_{21}q_{83} - 206656q_5q_{100} - 206656q_7q_{98} - 32q_8q_{97} -$
$32q_{14}q_{91} + 16q_{19}q_{86} - 206656q_5q_{101} - 32q_8q_{98} - 32q_9q_{97} + 32q_{22}q_{84} - 206656q_5q_{102} - 206656q_8q_{99} -$
$16q_9q_{98} - 32q_{10}q_{97} - 206656q_{15}q_{92} - 32q_{21}q_{86} - 32q_{22}q_{85} - 32q_{23}q_{84} - 206656q_5q_{103} - 16q_9q_{99} - 32q_{10}q_{98} -$
$32q_{11}q_{97} - 32q_{16}q_{92} + 32q_{23}q_{85} - 206656q_5q_{104} - 206656q_9q_{100} - 16q_{10}q_{99} - 32q_{11}q_{98} - 32q_{12}q_{97} -$
$206656q_{16}q_{93} - 206656q_5q_{105} - 32q_{10}q_{100} - 32q_{11}q_{99} - 32q_{12}q_{98} - 32q_{13}q_{97} - 32q_{17}q_{93} - 206656q_5q_{106} -$
$206656q_{10}q_{101} - 32q_{11}q_{100} - 32q_{12}q_{99} - 32q_{13}q_{98} - 32q_{14}q_{97} - 206656q_{17}q_{94} - 206656q_5q_{107} - 32q_{11}q_{101} -$
$16q_{12}q_{100} - 16q_{13}q_{99} - 16q_{14}q_{98} - 32q_{15}q_{97} - 32q_{18}q_{94} - 206656q_5q_{108} - 206656q_{11}q_{102} - 32q_{12}q_{101} +$
$16q_{13}q_{100} - 16q_{14}q_{99} - 16q_{15}q_{98} - 32q_{16}q_{97} - 206656q_{18}q_{95} - 206656q_5q_{109} - 32q_{12}q_{102} - 16q_{13}q_{101} +$

$16q_{14}q_{100} - 16q_{15}q_{99} - 32q_{16}q_{98} - 32q_{17}q_{97} - 32q_{19}q_{95} - 206656q_{12}q_{103} - 32q_{13}q_{102} + 16q_{14}q_{101} - 16q_{16}q_{99} -$
$32q_{17}q_{98} - 32q_{18}q_{97} - 206656q_{19}q_{96} - 206656q_{6}q_{110} - 32q_{13}q_{103} - 16q_{14}q_{102} - 32q_{17}q_{99} - 32q_{18}q_{98} -$
$32q_{19}q_{97} - 32q_{20}q_{96} - 206656q_{6}q_{111} - 206656q_{7}q_{110} - 206656q_{13}q_{104} - 32q_{14}q_{103} - 16q_{18}q_{99} - 16q_{19}q_{98} -$
$16q_{20}q_{97} - 206656q_{6}q_{112} - 32q_{14}q_{104} - 16q_{19}q_{99} - 16q_{20}q_{98} + 32q_{21}q_{97} - 206656q_{6}q_{113} - 206656q_{8}q_{111} -$
$32q_{9}q_{110} - 16q_{20}q_{99} - 206656q_{6}q_{114} - 16q_{9}q_{111} - 32q_{10}q_{110} - 206656q_{15}q_{105} + 32q_{22}q_{98} - 206656q_{6}q_{115} -$
$206656q_{9}q_{112} - 32q_{10}q_{111} - 32q_{11}q_{110} - 32q_{16}q_{105} + 32q_{38}q_{83} - 206656q_{6}q_{116} - 32q_{10}q_{112} - 32q_{11}q_{111} -$
$32q_{12}q_{110} - 206656q_{16}q_{106} - 32q_{17}q_{105} + 32q_{23}q_{99} - 206656q_{6}q_{117} - 206656q_{10}q_{113} - 32q_{11}q_{112} - 32q_{12}q_{111} -$
$32q_{13}q_{110} - 32q_{17}q_{106} - 16q_{18}q_{105} + 32q_{39}q_{84} - 206656q_{6}q_{118} - 32q_{11}q_{113} - 32q_{13}q_{111} - 32q_{14}q_{110} -$
$206656q_{17}q_{107} - 32q_{18}q_{106} + 16q_{19}q_{105} - 32q_{38}q_{86} - 32q_{39}q_{85} - 206656q_{6}q_{119} - 206656q_{11}q_{114} - 32q_{12}q_{113} +$
$16q_{13}q_{112} - 16q_{14}q_{111} - 32q_{15}q_{110} - 32q_{18}q_{107} - 16q_{19}q_{106} + 16q_{20}q_{105} - 206656q_{6}q_{120} - 32q_{12}q_{114} -$
$16q_{15}q_{111} - 32q_{16}q_{110} - 206656q_{18}q_{108} - 32q_{19}q_{107} + 16q_{20}q_{106} - 206656q_{6}q_{121} - 206656q_{12}q_{115} -$
$32q_{13}q_{114} + 16q_{14}q_{113} - 32q_{16}q_{111} - 32q_{17}q_{110} - 32q_{19}q_{108} - 16q_{20}q_{107} - 32q_{13}q_{115} - 32q_{17}q_{111} -$
$32q_{18}q_{110} - 206656q_{19}q_{109} - 32q_{20}q_{108} - 206656q_{7}q_{122} - 206656q_{13}q_{116} - 32q_{14}q_{115} - 32q_{18}q_{111} -$
$32q_{19}q_{110} - 32q_{20}q_{109} - 206656q_{7}q_{123} - 206656q_{8}q_{122} - 32q_{14}q_{116} - 16q_{19}q_{111} - 16q_{20}q_{110} - 206656q_{7}q_{124} -$
$32q_{9}q_{122} - 16q_{20}q_{111} + 32q_{21}q_{110} - 206656q_{7}q_{125} - 206656q_{9}q_{123} - 32q_{10}q_{122} - 206656q_{15}q_{117} -$
$206656q_{7}q_{126} - 32q_{10}q_{123} - 32q_{11}q_{122} - 32q_{16}q_{117} + 32q_{22}q_{111} - 206656q_{7}q_{127} - 206656q_{10}q_{124} - 32q_{12}q_{122} -$
$206656q_{16}q_{118} - 32q_{17}q_{117} - 206656q_{7}q_{128} - 32q_{11}q_{124} - 32q_{13}q_{122} - 32q_{17}q_{118} + 32q_{38}q_{97} - 206656q_{7}q_{129} -$
$206656q_{11}q_{125} - 16q_{13}q_{123} - 32q_{14}q_{122} - 206656q_{17}q_{119} - 32q_{18}q_{118} + 16q_{19}q_{117} - 206656q_{7}q_{130} -$
$32q_{12}q_{125} - 32q_{15}q_{122} - 32q_{18}q_{119} + 32q_{39}q_{98} + 32q_{54}q_{83} - 206656q_{7}q_{131} - 206656q_{12}q_{126} - 16q_{14}q_{124} -$
$32q_{16}q_{122} - 206656q_{18}q_{120} - 32q_{19}q_{119} + 16q_{20}q_{118} - 206656q_{7}q_{132} - 32q_{13}q_{126} - 32q_{17}q_{122} - 32q_{19}q_{120} -$
$206656q_{13}q_{127} - 32q_{18}q_{122} - 206656q_{19}q_{121} - 32q_{20}q_{120} - 32q_{54}q_{86} - 206656q_{8}q_{133} - 32q_{14}q_{127} - 32q_{19}q_{122} -$
$32q_{20}q_{121} - 206656q_{8}q_{134} - 206656q_{9}q_{133} - 16q_{20}q_{122} - 206656q_{8}q_{135} - 206656q_{15}q_{128} + 32q_{21}q_{122} -$
$206656q_{8}q_{136} - 206656q_{10}q_{134} - 32q_{16}q_{128} - 206656q_{8}q_{137} - 16q_{12}q_{133} - 206656q_{16}q_{129} - 206656q_{8}q_{138} -$
$206656q_{11}q_{135} - 16q_{13}q_{133} - 32q_{17}q_{129} - 16q_{13}q_{134} - 16q_{14}q_{133} - 206656q_{17}q_{130} - 16q_{19}q_{128} - 206656q_{9}q_{139} -$
$16q_{14}q_{134} - 32q_{18}q_{130} + 32q_{38}q_{110} - 206656q_{9}q_{140} - 206656q_{10}q_{139} - 16q_{14}q_{135} - 206656q_{18}q_{131} -$
$16q_{20}q_{129} - 206656q_{9}q_{141} - 64q_{11}q_{139} - 32q_{19}q_{131} + 32q_{39}q_{111} - 206656q_{9}q_{142} - 206656q_{11}q_{140} -$
$64q_{12}q_{139} - 206656q_{15}q_{136} - 206656q_{19}q_{132} + 32q_{54}q_{97} - 206656q_{9}q_{143} - 96q_{12}q_{140} - 64q_{13}q_{139} -$
$32q_{20}q_{132} - 206656q_{9}q_{144} - 206656q_{12}q_{141} - 32q_{13}q_{140} - 64q_{14}q_{139} - 206656q_{16}q_{137} - 206656q_{9}q_{145} -$
$64q_{13}q_{141} - 32q_{14}q_{140} - 32q_{15}q_{139} - 16q_{18}q_{136} - 206656q_{9}q_{146} - 206656q_{13}q_{142} - 32q_{14}q_{141} - 16q_{15}q_{140} -$
$32q_{16}q_{139} - 206656q_{17}q_{138} - 16q_{19}q_{136} - 206656q_{9}q_{147} - 64q_{14}q_{142} - 16q_{15}q_{141} - 32q_{16}q_{140} - 32q_{17}q_{139} -$
$16q_{19}q_{137} - 16q_{20}q_{136} - 206656q_{9}q_{148} - 206656q_{14}q_{143} - 16q_{15}q_{142} - 16q_{16}q_{141} - 32q_{17}q_{140} - 32q_{18}q_{139} -$
$16q_{20}q_{137} - 16q_{15}q_{143} - 16q_{16}q_{142} - 32q_{17}q_{141} - 32q_{18}q_{140} - 32q_{19}q_{139} - 16q_{20}q_{138} - 206656q_{10}q_{149} -$
$206656q_{15}q_{144} - 16q_{18}q_{141} - 16q_{19}q_{140} - 16q_{20}q_{139} - 206656q_{10}q_{150} - 206656q_{11}q_{149} - 32q_{16}q_{144} -$
$16q_{18}q_{142} - 16q_{19}q_{141} - 16q_{20}q_{140} + 32q_{21}q_{139} + 32q_{38}q_{122} - 206656q_{10}q_{151} - 64q_{12}q_{149} - 206656q_{16}q_{145} -$
$16q_{17}q_{144} - 16q_{19}q_{142} - 16q_{20}q_{141} - 206656q_{10}q_{152} - 206656q_{12}q_{150} - 96q_{13}q_{149} - 32q_{17}q_{145} - 16q_{18}q_{144} -$
$16q_{19}q_{143} - 16q_{20}q_{142} + 32q_{22}q_{140} - 206656q_{10}q_{153} - 96q_{13}q_{150} - 64q_{14}q_{149} - 206656q_{17}q_{146} - 16q_{18}q_{145} -$
$16q_{19}q_{144} - 16q_{20}q_{143} - 206656q_{10}q_{154} - 206656q_{13}q_{151} - 32q_{14}q_{150} - 32q_{15}q_{149} - 32q_{18}q_{146} - 16q_{19}q_{145} -$
$16q_{20}q_{144} + 32q_{23}q_{141} + 32q_{54}q_{110} - 206656q_{10}q_{155} - 64q_{14}q_{151} - 16q_{15}q_{150} - 32q_{16}q_{149} - 206656q_{18}q_{147} -$
$16q_{19}q_{146} - 16q_{20}q_{145} - 206656q_{10}q_{156} - 206656q_{14}q_{152} - 16q_{15}q_{151} - 32q_{16}q_{150} - 32q_{17}q_{149} - 32q_{19}q_{147} -$
$16q_{20}q_{146} - 206656q_{10}q_{157} - 16q_{15}q_{152} - 16q_{16}q_{151} - 32q_{17}q_{150} - 32q_{18}q_{149} - 206656q_{19}q_{148} - 16q_{20}q_{147} -$
$206656q_{15}q_{153} - 16q_{16}q_{152} - 32q_{17}q_{151} - 32q_{18}q_{150} - 32q_{19}q_{149} - 32q_{20}q_{148} - 206656q_{11}q_{158} - 32q_{16}q_{153} -$
$16q_{18}q_{151} - 16q_{19}q_{150} - 16q_{20}q_{149} - 206656q_{11}q_{159} - 206656q_{12}q_{158} - 206656q_{16}q_{154} - 32q_{17}q_{153} -$
$16q_{18}q_{152} - 16q_{19}q_{151} - 16q_{20}q_{150} + 32q_{21}q_{149} - 206656q_{11}q_{160} - 64q_{13}q_{158} - 32q_{17}q_{154} - 16q_{18}q_{153} -$
$16q_{19}q_{152} - 16q_{20}q_{151} - 206656q_{11}q_{161} - 206656q_{13}q_{159} - 96q_{14}q_{158} - 206656q_{17}q_{155} - 32q_{18}q_{154} -$
$16q_{19}q_{153} - 16q_{20}q_{152} + 32q_{22}q_{150} - 206656q_{11}q_{162} - 64q_{14}q_{159} - 32q_{15}q_{158} - 32q_{18}q_{155} - 16q_{19}q_{154} -$
$16q_{20}q_{153} - 206656q_{11}q_{163} - 206656q_{14}q_{160} - 16q_{15}q_{159} - 32q_{16}q_{158} - 206656q_{18}q_{156} - 32q_{19}q_{155} -$
$16q_{20}q_{154} + 32q_{23}q_{151} - 206656q_{11}q_{164} - 16q_{15}q_{160} - 32q_{16}q_{159} - 32q_{17}q_{158} - 32q_{19}q_{156} - 16q_{20}q_{155} -$
$206656q_{11}q_{165} - 206656q_{15}q_{161} - 16q_{16}q_{160} - 32q_{17}q_{159} - 32q_{18}q_{158} - 206656q_{19}q_{157} - 32q_{20}q_{156} +$

$32q_{54}q_{122} - 32q_{16}q_{161} - 32q_{17}q_{160} - 32q_{18}q_{159} - 32q_{19}q_{158} - 32q_{20}q_{157} + 32q_{38}q_{139} - 206656q_{12}q_{166} -$
$206656q_{16}q_{162} - 32q_{17}q_{161} - 16q_{18}q_{160} - 16q_{19}q_{159} - 16q_{20}q_{158} - 206656q_{12}q_{167} - 206656q_{13}q_{166} -$
$32q_{17}q_{162} - 32q_{18}q_{161} - 16q_{19}q_{160} - 16q_{20}q_{159} + 32q_{21}q_{158} + 32q_{39}q_{140} - 206656q_{12}q_{168} - 64q_{14}q_{166} -$
$206656q_{17}q_{163} - 32q_{18}q_{162} - 16q_{19}q_{161} - 16q_{20}q_{160} - 206656q_{12}q_{169} - 206656q_{14}q_{167} - 32q_{15}q_{166} -$
$32q_{18}q_{163} - 32q_{19}q_{162} + 32q_{22}q_{159} - 206656q_{12}q_{170} - 16q_{15}q_{167} - 32q_{16}q_{166} - 206656q_{18}q_{164} - 32q_{19}q_{163} -$
$16q_{20}q_{162} - 206656q_{12}q_{171} - 206656q_{15}q_{168} - 32q_{16}q_{167} - 32q_{17}q_{166} - 32q_{19}q_{164} - 32q_{20}q_{163} + 32q_{23}q_{160} -$
$206656q_{12}q_{172} - 32q_{16}q_{168} - 32q_{17}q_{167} - 32q_{18}q_{166} - 206656q_{19}q_{165} - 32q_{20}q_{164} - 206656q_{16}q_{169} -$
$32q_{17}q_{168} - 32q_{18}q_{167} - 32q_{19}q_{166} - 32q_{20}q_{165} - 206656q_{13}q_{173} - 32q_{17}q_{169} - 32q_{18}q_{168} - 16q_{19}q_{167} -$
$16q_{20}q_{166} - 206656q_{13}q_{174} - 206656q_{14}q_{173} - 206656q_{17}q_{170} - 32q_{18}q_{169} - 16q_{19}q_{168} - 16q_{20}q_{167} +$
$32q_{21}q_{166} + 32q_{38}q_{149} - 206656q_{13}q_{175} - 32q_{15}q_{173} - 32q_{18}q_{170} - 32q_{19}q_{169} - 206656q_{13}q_{176} - 206656q_{15}q_{174} -$
$32q_{16}q_{173} - 206656q_{18}q_{171} - 32q_{19}q_{170} - 16q_{20}q_{169} + 32q_{22}q_{167} + 32q_{39}q_{150} - 206656q_{13}q_{177} - 32q_{16}q_{174} -$
$32q_{17}q_{173} - 32q_{19}q_{171} - 32q_{20}q_{170} - 206656q_{13}q_{178} - 206656q_{16}q_{175} - 32q_{17}q_{174} - 32q_{18}q_{173} - 206656q_{19}q_{172} -$
$32q_{20}q_{171} - 32q_{17}q_{175} - 16q_{18}q_{174} - 32q_{19}q_{173} - 32q_{20}q_{172} - 206656q_{14}q_{179} - 206656q_{17}q_{176} - 32q_{18}q_{175} -$
$16q_{19}q_{174} - 16q_{20}q_{173} + 32q_{54}q_{139} + 64q_{83}q_{110} - 206656q_{14}q_{180} - 206656q_{15}q_{179} - 32q_{18}q_{176} - 16q_{19}q_{175} -$
$16q_{20}q_{174} + 32q_{21}q_{173} - 64q_{83}q_{111} - 206656q_{14}q_{181} - 32q_{16}q_{179} - 206656q_{18}q_{177} - 32q_{19}q_{176} - 16q_{20}q_{175} -$
$206656q_{14}q_{182} - 206656q_{16}q_{180} - 16q_{17}q_{179} - 32q_{19}q_{177} - 16q_{20}q_{176} + 32q_{38}q_{158} - 206656q_{14}q_{183} -$
$32q_{17}q_{180} - 16q_{18}q_{179} - 206656q_{19}q_{178} - 32q_{20}q_{177} - 206656q_{17}q_{181} - 16q_{18}q_{180} - 16q_{19}q_{179} - 32q_{20}q_{178} +$
$32q_{39}q_{159} - 206656q_{15}q_{184} - 32q_{18}q_{181} - 16q_{19}q_{180} - 16q_{20}q_{179} - 206656q_{15}q_{185} - 206656q_{16}q_{184} -$
$206656q_{18}q_{182} - 16q_{19}q_{181} - 16q_{20}q_{180} - 206656q_{15}q_{186} - 64q_{17}q_{184} - 32q_{19}q_{182} - 16q_{20}q_{181} - 206656q_{15}q_{187} -$
$206656q_{17}q_{185} - 64q_{18}q_{184} - 206656q_{19}q_{183} - 16q_{20}q_{182} - 206656q_{15}q_{188} - 96q_{18}q_{185} - 64q_{19}q_{184} -$
$32q_{20}q_{183} + 32q_{54}q_{149} - 206656q_{18}q_{186} - 32q_{20}q_{184} + 32q_{38}q_{166} - 206656q_{16}q_{189} - 64q_{19}q_{186} - 32q_{20}q_{185} +$
$32q_{21}q_{184} + 64q_{83}q_{122} - 206656q_{16}q_{190} - 206656q_{17}q_{189} - 206656q_{19}q_{187} - 32q_{20}q_{186} + 32q_{39}q_{167} -$
$64q_{84}q_{122} - 206656q_{16}q_{191} - 64q_{18}q_{189} - 64q_{20}q_{187} + 32q_{22}q_{185} - 206656q_{16}q_{192} - 206656q_{18}q_{190} -$
$96q_{19}q_{189} - 206656q_{20}q_{188} - 64q_{19}q_{190} - 32q_{20}q_{189} + 32q_{23}q_{186} - 206656q_{17}q_{193} - 206656q_{19}q_{191} -$
$32q_{20}q_{190} + 32q_{21}q_{189} - 206656q_{17}q_{194} - 206656q_{18}q_{193} - 64q_{20}q_{191} + 32q_{38}q_{173} - 206656q_{17}q_{195} -$
$64q_{19}q_{193} - 206656q_{20}q_{192} + 32q_{22}q_{190} + 32q_{54}q_{158} - 206656q_{19}q_{194} - 64q_{20}q_{193} - 206656q_{18}q_{196} -$
$64q_{20}q_{194} + 32q_{21}q_{193} + 32q_{23}q_{191} - 206656q_{18}q_{197} - 206656q_{19}q_{196} - 206656q_{20}q_{195} - 32q_{20}q_{196} +$
$32q_{22}q_{194} - 206656q_{19}q_{198} - 206656q_{20}q_{197} + 32q_{21}q_{196} - 206656q_{20}q_{198} + 32q_{23}q_{195} + 32q_{21}q_{198} +$
$32q_{22}q_{197} + 64q_{97}q_{122} + 32q_{54}q_{166} + 32q_{38}q_{184} + 32q_{83}q_{139} + 32q_{39}q_{185} + 32q_{84}q_{140} - 32q_{83}q_{142} - 32q_{84}q_{141} -$
$32q_{85}q_{140} - 32q_{86}q_{139} + 32q_{85}q_{141} + 32q_{38}q_{189} + 32q_{54}q_{173} + 32q_{86}q_{142} + 32q_{39}q_{190} + 32q_{38}q_{193} + 32q_{83}q_{149} +$
$32q_{39}q_{194} + 32q_{38}q_{196} + 32q_{84}q_{150} - 32q_{83}q_{152} - 32q_{84}q_{151} - 32q_{85}q_{150} - 32q_{86}q_{149} + 32q_{38}q_{198} + 32q_{39}q_{197} +$
$32q_{85}q_{151} + 32q_{97}q_{139} + 32q_{54}q_{184} + 32q_{86}q_{152} + 32q_{98}q_{140} + 32q_{99}q_{141} + 32q_{83}q_{158} + 32q_{54}q_{189} + 32q_{84}q_{159} -$
$32q_{84}q_{160} - 32q_{85}q_{159} - 32q_{86}q_{158} + 32q_{85}q_{160} + 32q_{97}q_{149} + 32q_{54}q_{193} + 32q_{98}q_{150} + 32q_{83}q_{166} + 32q_{110}q_{139} +$
$32q_{54}q_{196} + 32q_{99}q_{151} + 32q_{84}q_{167} + 32q_{111}q_{140} + 32q_{54}q_{198} - 32q_{85}q_{167} - 32q_{86}q_{166} + 32q_{97}q_{158} + 32q_{83}q_{173} +$
$32q_{98}q_{159} - 32q_{86}q_{173} + 32q_{99}q_{160} + 32q_{110}q_{149} + 32q_{111}q_{150} + 32q_{122}q_{139} + 32q_{97}q_{166} + 32q_{98}q_{167} + 32q_{83}q_{184} +$
$32q_{110}q_{158} + 32q_{84}q_{185} - 32q_{83}q_{187} - 32q_{84}q_{186} - 32q_{85}q_{185} - 32q_{86}q_{184} + 32q_{97}q_{173} + 32q_{111}q_{159} + 32q_{85}q_{186} +$
$32q_{122}q_{149} + 32q_{83}q_{189} + 32q_{86}q_{187} + 32q_{84}q_{190} - 32q_{83}q_{192} - 32q_{84}q_{191} - 32q_{85}q_{190} - 32q_{86}q_{189} + 32q_{83}q_{193} +$
$32q_{85}q_{191} + 32q_{110}q_{166} + 32q_{84}q_{194} + 32q_{86}q_{192} + 32q_{111}q_{167} + 32q_{83}q_{196} - 32q_{84}q_{195} - 32q_{85}q_{194} - 32q_{86}q_{193} +$
$32q_{85}q_{195} + 32q_{122}q_{158} + 32q_{83}q_{198} + 32q_{84}q_{197} + 32q_{97}q_{184} - 32q_{85}q_{197} - 32q_{86}q_{196} + 32q_{98}q_{185} + 32q_{110}q_{173} -$
$32q_{86}q_{198} + 32q_{99}q_{186} + 32q_{97}q_{189} + 32q_{98}q_{190} + 32q_{122}q_{166} + 32q_{97}q_{193} + 32q_{99}q_{191} + 32q_{98}q_{194} + 32q_{97}q_{196} +$
$32q_{99}q_{195} + 32q_{110}q_{184} + 32q_{97}q_{198} + 32q_{98}q_{197} + 32q_{122}q_{173} + 32q_{111}q_{185} + 64q_{139}q_{158} + 32q_{110}q_{189} +$
$32q_{111}q_{190} + 32q_{110}q_{193} + 32q_{111}q_{194} + 64q_{139}q_{166} + 32q_{110}q_{196} + 32q_{122}q_{184} + 64q_{140}q_{167} + 32q_{110}q_{198} +$
$32q_{111}q_{197} + 32q_{122}q_{189} + 64q_{139}q_{173} + 32q_{122}q_{193} + 64q_{149}q_{166} + 32q_{122}q_{196} + 32q_{122}q_{198} + 64q_{149}q_{173} +$
$32q_{139}q_{184} + 32q_{140}q_{185} + 32q_{141}q_{186} + 32q_{139}q_{189} + 32q_{142}q_{187} + 32q_{140}q_{190} + 32q_{143}q_{188} + 64q_{158}q_{173} +$
$32q_{139}q_{193} + 32q_{141}q_{191} + 32q_{149}q_{184} + 32q_{140}q_{194} + 32q_{142}q_{192} + 32q_{139}q_{196} + 32q_{150}q_{185} + 32q_{141}q_{195} +$
$32q_{139}q_{198} + 32q_{140}q_{197} + 32q_{151}q_{186} + 32q_{149}q_{189} + 32q_{152}q_{187} + 32q_{150}q_{190} + 32q_{149}q_{193} + 32q_{151}q_{191} +$
$32q_{158}q_{184} + 32q_{150}q_{194} + 32q_{152}q_{192} + 32q_{159}q_{185} + 32q_{149}q_{196} + 32q_{151}q_{195} + 32q_{160}q_{186} + 32q_{149}q_{198} +$
$32q_{150}q_{197} + 32q_{158}q_{189} + 32q_{159}q_{190} + 32q_{166}q_{184} + 32q_{158}q_{193} + 32q_{160}q_{191} + 32q_{167}q_{185} + 32q_{159}q_{194} +$

$32q_{158}q_{196} + 32q_{160}q_{195} + 32q_{166}q_{189} + 32q_{158}q_{198} + 32q_{159}q_{197} + 32q_{167}q_{190} + 32q_{173}q_{184} + 32q_{166}q_{193} +$
$32q_{167}q_{194} + 32q_{166}q_{196} + 32q_{173}q_{189} + 32q_{166}q_{198} + 32q_{167}q_{197} + 32q_{173}q_{193} + 32q_{173}q_{196} + 32q_{173}q_{198} +$
$64q_{184}q_{193} + 64q_{184}q_{196} + 64q_{184}q_{198} + 64q_{185}q_{197} + 64q_{189}q_{196} + 64q_{189}q_{198} + 64q_{193}q_{198} + 2056$

A complete expression of $E_2(s)$ in 92-order H matrix using Turyn based method with $\delta = 103, 328$

$E_2(s) = 439290s_0 + 439286s_1 + 439294s_2 + 439294s_3 + 465068s_4 + 465164s_5 + 465180s_6 + 465172s_7 +$
$258382s_8+491030s_9+491138s_{10}+491214s_{11}+465440s_{12}+465476s_{13}+258726s_{14}+516956s_{15}+517116s_{16}+$
$517228s_{17} +491472s_{18} +491486s_{19} +129652s_{20} -51724s_{21} -51724s_{22} -51716s_{23} -51680s_{24} -51680s_{25} -$
$51676s_{26} - 51676s_{27} - 51672s_{28} - 51676s_{29} - 51688s_{30} - 51692s_{31} - 51692s_{32} - 51672s_{2}4 - 51680s_{34} -$
$51684s_{35} - 51688s_{36} - 51684s_{37} - 51724s_{38} - 51732s_{39} - 51680s_{40} - 51684s_{41} - 51684s_{42} - 51668s_{43} -$
$51672s_{44} - 51680s_{45} - 51684s_{46} - 51692s_{47} - 51692s_{48} - 51672s_{49} - 51680s_{50} - 51688s_{51} - 51684s_{52} -$
$51684s_{53} - 51748s_{54} - 51680s_{55} - 51676s_{56} - 51676s_{57} - 51676s_{58} - 51672s_{59} - 51680s_{60} - 51684s_{61} -$
$51684s_{62} - 51692s_{63} - 51672s_{64} - 51680s_{65} - 51680s_{66} - 51684s_{67} - 51684s_{68} - 51680s_{69} - 51664s_{70} -$
$51668s_{71} - 51676s_{72} - 51672s_{73} - 51676s_{74} - 51680s_{75} - 51684s_{76} - 51684s_{77} - 51672s_{78} - 51672s_{79} -$
$51676s_{80} - 51680s_{81} - 51684s_{82} - 51728s_{83} - 51696s_{84} - 51656s_{85} - 51608s_{86} - 51664s_{87} - 51672s_{88} -$
$51672s_{89} - 51672s_{90} - 51672s_{91} - 51672s_{92} - 51672s_{93} - 51672s_{94} - 51672s_{95} - 51672s_{96} - 51772s_{97} -$
$51732s_{98}-51700s_{99}-51672s_{100}-51676s_{101}-51680s_{102}-51684s_{9}4-51684s_{104}-51672s_{105}-51680s_{106}-$
$51684s_{107}-51680s_{108}-51676s_{109}-51772s_{110}-51708s_{111}-51672s_{112}-51680s_{113}-51676s_{114}-51676s_{115}-$
$51684s_{116}-51672s_{117}-51680s_{118}-51680s_{119}-51676s_{120}-51676s_{121}-51780s_{122}-51672s_{123}-51672s_{124}-$
$51676s_{125} - 51676s_{11}7 - 51676s_{127} - 51672s_{128} - 51672s_{129} - 51672s_{130} - 51676s_{131} - 51676s_{132} -$
$51664s_{133}-51668s_{134}-51672s_{135}-51664s_{136}-51664s_{137}-51668s_{138}-51796s_{139}-51756s_{140}-51724s_{141}-$
$51708s_{142}-51708s_{143}-51672s_{144}-51676s_{145}-51680s_{146}-51684s_{147}-51688s_{148}-51796s_{149}-51748s_{150}-$
$51748s_{151}-51724s_{152}-51672s_{153}-51680s_{154}-51684s_{155}-51688s_{156}-51692s_{157}-51820s_{158}-51764s_{159}-$
$51748s_{160}-51672s_{161}-51680s_{162}-51688s_{163}-51692s_{164}-51692s_{165}-51836s_{166}-51796s_{167}-51672s_{168}-$
$51680s_{169}-51688s_{170}-51692s_{171}-51692s_{172}-51860s_{173}-51672s_{174}-51680s_{175}-51684s_{176}-51688s_{177}-$
$51692s_{178} - 51672s_{179} - 51676s_{180} - 51680s_{181} - 51684s_{182} - 51688s_{183} - 51844s_{184} - 51796s_{17}6 -$
$51756s_{186}-51724s_{187}-51708s_{188}-51844s_{189}-51788s_{190}-51764s_{191}-51732s_{192}-51860s_{193}-51796s_{194}-$
$51764s_{195}-51876s_{196}-51820s_{197}-51900s_{198}+25832s_0s_1+25832s_0s_2+25832s_0s_3+25832s_1s_2+25832s_0s_4+$
$25832s_1s_3+25832s_0s_5+25832s_1s_4+25832s_2s_3+25832s_0s_6+25832s_1s_5+25832s_2s_4+25832s_0s_7+25832s_1s_6+$
$25832s_2s_5 + 25832s_3s_4 + 2s_0s_8 + 25832s_1s_7 + 25832s_2s_6 + 25832s_3s_5 + 25832s_0s_9 + 2s_1s_8 + 25832s_2s_7 +$
$25832s_3s_6+25832s_4s_5+25832s_0s_{10}+25832s_1s_9+2s_2s_8+25832s_3s_7+25832s_4s_6+25832s_0s_{11}+25832s_1s_{10}+$
$25832s_2s_9+6s_3s_8+25832s_4s_7+25832s_5s_6+25832s_0s_{12}+25832s_1s_{11}+25832s_2s_{10}+25832s_3s_9+25832s_4s_8+$
$25832s_5s_7 + 25832s_0s_{13} + 25832s_1s_{12} + 25832s_2s_{11} + 25832s_3s_{10} + 25832s_4s_9 + 25832s_5s_8 + 25832s_6s_7 +$
$14s_0s_{14}+25832s_1s_{13}+25832s_2s_{12}+25832s_3s_{11}+25832s_4s_{10}+25832s_5s_9+25832s_6s_8+25832s_0s_{15}+14s_1s_{14}+$
$25832s_2s_{13} + 25832s_3s_{12} + 25832s_4s_{11} + 25832s_5s_{10} + 25832s_6s_9 + 25832s_7s_8 + 25832s_0s_{16} + 25832s_1s_{15} +$
$14s_2s_{14} + 25832s_3s_{13} + 25832s_4s_{12} + 25832s_5s_{11} + 25832s_6s_{10} + 25832s_7s_9 + 25832s_0s_{17} + 25832s_1s_{16} +$
$25832s_2s_{15} + 14s_3s_{14} + 25832s_4s_{13} + 25832s_5s_{12} + 25832s_6s_{11} + 25832s_7s_{10} + 25832s_8s_9 + 25832s_0s_{18} +$
$25832s_1s_{17} + 25832s_2s_{16} + 25832s_3s_{15} + 4s_4s_{14} + 25832s_5s_{13} + 25832s_6s_{12} + 25832s_7s_{11} + 25832s_8s_{10} +$
$25832s_0s_{19} + 25832s_1s_{18} + 25832s_2s_{17} + 25832s_3s_{16} + 25832s_4s_{15} + 10s_5s_{14} + 25832s_6s_{13} + 25832s_7s_{12} +$
$25832s_8s_{11}+25832s_9s_{10}+10s_0s_{20}+25832s_1s_{19}+25832s_2s_{18}+25832s_3s_{17}+25832s_4s_{16}+25832s_5s_{15}+10s_6s_{14}+$
$25832s_7s_{13}+6s_8s_{12}+25832s_9s_{11}-51664s_0s_{21}+10s_1s_{20}+25832s_2s_{19}+25832s_3s_{18}+25832s_4s_{17}+25832s_5s_{16}+$
$25832s_6s_{15} + 10s_7s_{14} + 6s_8s_{13} + 25832s_9s_{12} + 25832s_{10}s_{11} - 51664s_0s_{22} - 51664s_1s_{21} + 10s_2s_{20} + 25832s_3s_{19} +$
$25832s_4s_{18}+25832s_5s_{17}+25832s_6s_{16}+25832s_7s_{15}+6s_8s_{14}+25832s_9s_{13}+25832s_{10}s_{12}-51664s_0s_{23}-16s_2s_{21}+$
$10s_3s_{20} + 25832s_4s_{19} + 25832s_5s_{18} + 25832s_6s_{17} + 25832s_7s_{16} + 25832s_8s_{15} + 25832s_9s_{14} + 25832s_{10}s_{13} +$
$25832s_{11}s_{12} - 51664s_0s_{24} - 51664s_2s_{22} - 24s_3s_{21} + 25832s_5s_{19} + 25832s_6s_{18} + 25832s_7s_{17} + 25832s_8s_{16} +$
$25832s_9s_{15} + 25832s_{10}s_{14} + 25832s_{11}s_{13} - 51664s_0s_{25} - 8s_3s_{22} - 8s_4s_{21} + 6s_5s_{20} + 25832s_6s_{19} + 25832s_7s_{18} +$
$25832s_8s_{17} + 25832s_9s_{16} + 25832s_{10}s_{15} + 25832s_{11}s_{14} + 25832s_{12}s_{13} - 51664s_0s_{26} - 51664s_3s_{23} - 8s_5s_{21} +$

$6s_{6}s_{20}+25832s_{7}s_{19}+4s_{8}s_{18}+25832s_{9}s_{17}+25832s_{10}s_{16}+25832s_{11}s_{15}+25832s_{12}s_{14}-51664s_{0}s_{27}-8s_{5}s_{22}-$
$8s_{6}s_{21}+6s_{7}s_{20}+6s_{8}s_{19}+25832s_{9}s_{18}+25832s_{10}s_{17}+25832s_{11}s_{16}+25832s_{12}s_{15}+25832s_{13}s_{14}-51664s_{0}s_{28}-$
$51664s_{4}s_{24}-4s_{5}s_{23}-8s_{6}s_{22}-8s_{7}s_{21}+6s_{8}s_{20}+25832s_{9}s_{19}+25832s_{10}s_{18}+25832s_{11}s_{17}+25832s_{12}s_{16}+$
$25832s_{13}s_{15}-51664s_{0}s_{29}-4s_{5}s_{24}+14s_{9}s_{20}+25832s_{10}s_{19}+25832s_{11}s_{18}+25832s_{12}s_{17}+25832s_{13}s_{16}+$
$25832s_{14}s_{15}-51664s_{0}s_{30}-51664s_{5}s_{25}-4s_{6}s_{24}-8s_{9}s_{21}+14s_{10}s_{20}+25832s_{11}s_{19}+25832s_{12}s_{18}+25832s_{13}s_{17}+$
$25832s_{14}s_{16}-51664s_{0}s_{31}-8s_{6}s_{25}+4s_{7}s_{24}-4s_{8}s_{23}-4s_{9}s_{22}-8s_{10}s_{21}+14s_{11}s_{20}+25832s_{12}s_{19}+25832s_{13}s_{18}+$
$25832s_{14}s_{17}+25832s_{15}s_{16}-51664s_{0}s_{32}-51664s_{6}s_{26}-8s_{7}s_{25}+4s_{8}s_{24}-4s_{9}s_{23}-8s_{10}s_{22}-8s_{11}s_{21}+14s_{12}s_{20}+$
$25832s_{13}s_{19}+25832s_{14}s_{18}+25832s_{15}s_{17}-51664s_{0}s_{2}4-8s_{7}s_{26}-4s_{8}s_{25}-4s_{10}s_{23}-8s_{11}s_{22}-8s_{12}s_{21}+14s_{13}s_{20}+$
$25832s_{14}s_{19}+25832s_{15}s_{18}+25832s_{16}s_{17}-51664s_{0}s_{34}-51664s_{7}s_{27}-8s_{8}s_{26}-8s_{11}s_{23}-8s_{12}s_{22}-8s_{13}s_{21}+$
$14s_{14}s_{20}+25832s_{15}s_{19}+25832s_{16}s_{18}-51664s_{0}s_{35}-8s_{8}s_{27}-8s_{12}s_{23}-8s_{13}s_{22}-8s_{14}s_{21}+25832s_{15}s_{20}+$
$25832s_{16}s_{19}+25832s_{17}s_{18}-51664s_{0}s_{36}-4s_{13}s_{23}-4s_{14}s_{22}-8s_{15}s_{21}+25832s_{16}s_{20}+25832s_{17}s_{19}-51664s_{0}s_{37}-$
$51664s_{9}s_{28}-4s_{14}s_{23}-4s_{15}s_{22}-8s_{16}s_{21}+25832s_{17}s_{20}+25832s_{18}s_{19}-8s_{10}s_{28}-4s_{15}s_{23}-8s_{16}s_{22}-8s_{17}s_{21}+$
$25832s_{18}s_{20}-51664s_{1}s_{38}-51664s_{10}s_{29}-8s_{11}s_{28}-4s_{16}s_{23}-8s_{17}s_{22}-8s_{18}s_{21}+25832s_{19}s_{20}-51664s_{1}s_{39}-$
$51664s_{2}s_{38}-8s_{11}s_{29}-4s_{12}s_{28}-8s_{17}s_{23}-8s_{18}s_{22}-8s_{19}s_{21}-51664s_{1}s_{40}-16s_{3}s_{38}-51664s_{11}s_{30}-8s_{12}s_{29}-$
$4s_{13}s_{28}-4s_{18}s_{23}-4s_{19}s_{22}-4s_{20}s_{21}-51664s_{1}s_{41}-51664s_{3}s_{39}-8s_{4}s_{38}-8s_{12}s_{30}-4s_{13}s_{29}+4s_{14}s_{28}-4s_{19}s_{23}-$
$4s_{20}s_{22}-51664s_{1}s_{42}-8s_{5}s_{38}-51664s_{12}s_{31}-8s_{13}s_{30}-4s_{14}s_{29}-4s_{20}s_{23}-51664s_{1}s_{43}-51664s_{4}s_{40}-8s_{5}s_{39}-$
$8s_{6}s_{38}-8s_{13}s_{31}-4s_{14}s_{30}-51664s_{1}s_{44}-12s_{5}s_{40}-8s_{6}s_{39}-8s_{7}s_{38}-51664s_{13}s_{32}-8s_{14}s_{31}-51664s_{1}s_{45}-$
$51664s_{5}s_{41}-8s_{14}s_{32}-51664s_{1}s_{46}-8s_{6}s_{41}-8s_{9}s_{38}-51664s_{1}s_{47}-51664s_{6}s_{42}-8s_{7}s_{41}+12s_{8}s_{40}-4s_{9}s_{39}-$
$8s_{10}s_{38}-51664s_{15}s_{2}4-51664s_{1}s_{48}-8s_{7}s_{42}-8s_{8}s_{41}-8s_{10}s_{39}-8s_{11}s_{38}-8s_{16}s_{2}4-51664s_{1}s_{49}-51664s_{7}s_{43}-$
$8s_{8}s_{42}-8s_{11}s_{39}-8s_{12}s_{38}-51664s_{16}s_{34}-8s_{17}s_{2}4-51664s_{1}s_{50}-8s_{8}s_{43}-8s_{12}s_{39}-8s_{13}s_{38}-8s_{17}s_{34}-4s_{18}s_{2}4-$
$51664s_{1}s_{51}-8s_{13}s_{39}-8s_{14}s_{38}-51664s_{17}s_{35}-8s_{18}s_{34}-4s_{19}s_{2}4-51664s_{1}s_{52}-51664s_{9}s_{44}-4s_{14}s_{39}-8s_{15}s_{38}-$
$8s_{18}s_{35}-4s_{19}s_{34}+4s_{20}s_{2}4-51664s_{1}s_{53}-8s_{10}s_{44}-4s_{15}s_{39}-8s_{16}s_{38}-51664s_{18}s_{36}-8s_{19}s_{35}-4s_{20}s_{34}-$
$51664s_{10}s_{45}-8s_{11}s_{44}-8s_{16}s_{39}-8s_{17}s_{38}-8s_{19}s_{36}-4s_{20}s_{35}-51664s_{2}s_{54}-8s_{11}s_{45}-8s_{12}s_{44}-8s_{17}s_{39}-$
$8s_{18}s_{38}-51664s_{19}s_{37}-8s_{20}s_{36}-51664s_{2}s_{55}-51664s_{3}s_{54}-51664s_{11}s_{46}-8s_{12}s_{45}+4s_{13}s_{44}-8s_{18}s_{39}-8s_{19}s_{38}-$
$8s_{20}s_{37}-51664s_{2}s_{56}-8s_{4}s_{54}-8s_{12}s_{46}-8s_{13}s_{45}-4s_{19}s_{39}-4s_{20}s_{38}-51664s_{2}s_{57}-51664s_{4}s_{55}-8s_{5}s_{54}-$
$51664s_{12}s_{47}-8s_{13}s_{46}+4s_{14}s_{45}-4s_{20}s_{39}-51664s_{2}s_{58}-4s_{5}s_{55}-8s_{6}s_{54}-8s_{13}s_{47}-8s_{14}s_{46}-51664s_{2}s_{59}-$
$51664s_{5}s_{56}-8s_{6}s_{55}-8s_{7}s_{54}-51664s_{13}s_{48}-8s_{14}s_{47}-51664s_{2}s_{60}-8s_{6}s_{56}+8s_{7}s_{55}-8s_{14}s_{48}-51664s_{2}s_{61}-$
$51664s_{6}s_{57}-8s_{7}s_{56}+4s_{8}s_{55}-8s_{9}s_{54}-51664s_{2}s_{62}-8s_{7}s_{57}-8s_{10}s_{54}-51664s_{15}s_{49}-51664s_{2}s_{63}-51664s_{7}s_{58}-$
$8s_{8}s_{57}-8s_{11}s_{54}-8s_{16}s_{49}-51664s_{2}s_{64}-8s_{8}s_{58}-8s_{12}s_{54}-51664s_{16}s_{50}-8s_{17}s_{49}-51664s_{2}s_{65}-8s_{13}s_{54}-$
$8s_{17}s_{50}-8s_{18}s_{49}-51664s_{2}s_{66}-51664s_{9}s_{59}-8s_{14}s_{54}-51664s_{17}s_{51}-8s_{18}s_{50}+4s_{19}s_{49}-51664s_{2}s_{67}-8s_{10}s_{59}-$
$8s_{15}s_{54}-8s_{18}s_{51}-8s_{19}s_{50}-51664s_{2}s_{68}-51664s_{10}s_{60}-8s_{11}s_{59}-8s_{16}s_{54}-51664s_{18}s_{52}-8s_{19}s_{51}+4s_{20}s_{50}-$
$8s_{11}s_{60}-8s_{17}s_{54}-8s_{19}s_{52}-8s_{20}s_{51}-51664s_{3}s_{69}-51664s_{11}s_{61}-8s_{12}s_{60}-4s_{13}s_{59}-8s_{18}s_{54}-51664s_{19}s_{53}-$
$8s_{20}s_{52}-51664s_{3}s_{70}-51664s_{4}s_{69}-8s_{12}s_{61}-8s_{19}s_{54}-8s_{20}s_{53}-51664s_{3}s_{71}-4s_{5}s_{69}-51664s_{12}s_{62}-8s_{13}s_{61}-$
$4s_{14}s_{60}-4s_{20}s_{54}-51664s_{3}s_{72}-51664s_{5}s_{70}+4s_{6}s_{69}-8s_{13}s_{62}+16s_{21}s_{54}-51664s_{3}s_{73}-8s_{6}s_{70}-4s_{7}s_{69}-$
$51664s_{13}s_{63}-8s_{14}s_{62}-51664s_{3}s_{74}-51664s_{6}s_{71}+4s_{8}s_{69}-8s_{14}s_{63}-51664s_{3}s_{75}-8s_{7}s_{71}-4s_{8}s_{70}-51664s_{3}s_{76}-$
$51664s_{7}s_{72}-51664s_{15}s_{64}-51664s_{3}s_{77}-8s_{8}s_{72}-8s_{16}s_{64}-51664s_{3}s_{78}-51664s_{16}s_{65}-8s_{17}s_{64}-51664s_{3}s_{79}-$
$51664s_{9}s_{73}-8s_{17}s_{65}-51664s_{3}s_{80}-8s_{10}s_{73}-51664s_{17}s_{66}-8s_{18}s_{65}-4s_{19}s_{64}-51664s_{3}s_{81}-51664s_{10}s_{74}-$
$8s_{18}s_{66}-51664s_{3}s_{82}-8s_{11}s_{74}-4s_{12}s_{73}-51664s_{18}s_{67}-8s_{19}s_{66}-4s_{20}s_{65}-51664s_{11}s_{75}-4s_{13}s_{73}-8s_{19}s_{67}-$
$51664s_{4}s_{83}-8s_{12}s_{75}-4s_{13}s_{74}-4s_{14}s_{73}-51664s_{19}s_{68}-8s_{20}s_{67}-51664s_{4}s_{84}-51664s_{5}s_{83}-51664s_{12}s_{76}-$
$4s_{14}s_{74}-8s_{20}s_{68}-51664s_{4}s_{85}-16s_{6}s_{83}-8s_{13}s_{76}-4s_{14}s_{75}-51664s_{4}s_{86}-51664s_{6}s_{84}-8s_{7}s_{83}-51664s_{13}s_{77}-$
$51664s_{4}s_{87}-16s_{7}s_{84}-8s_{14}s_{77}-51664s_{4}s_{88}-51664s_{7}s_{85}+24s_{8}s_{84}-4s_{9}s_{83}-51664s_{4}s_{89}-16s_{8}s_{85}-4s_{10}s_{83}-$
$51664s_{15}s_{78}-51664s_{4}s_{90}-51664s_{8}s_{86}-4s_{10}s_{84}-4s_{11}s_{83}-8s_{16}s_{78}-51664s_{4}s_{91}+4s_{9}s_{86}+4s_{10}s_{85}-4s_{12}s_{83}-$
$51664s_{16}s_{79}-51664s_{4}s_{92}-51664s_{9}s_{87}+4s_{10}s_{86}-4s_{13}s_{83}-8s_{17}s_{79}-4s_{18}s_{78}-51664s_{4}s_{93}-8s_{10}s_{87}+4s_{11}s_{86}-$
$4s_{13}s_{84}-4s_{14}s_{83}-51664s_{17}s_{80}-4s_{19}s_{78}-51664s_{4}s_{94}-51664s_{10}s_{88}+4s_{12}s_{86}+4s_{13}s_{85}-4s_{15}s_{83}-8s_{18}s_{80}-$
$4s_{19}s_{79}-4s_{20}s_{78}-51664s_{4}s_{95}-8s_{11}s_{88}+4s_{13}s_{86}-4s_{16}s_{83}-51664s_{18}s_{81}-4s_{20}s_{79}-51664s_{4}s_{96}-51664s_{11}s_{89}+$
$4s_{14}s_{86}-4s_{16}s_{84}-8s_{17}s_{83}-8s_{19}s_{81}-4s_{20}s_{80}-8s_{12}s_{89}+4s_{15}s_{86}+4s_{16}s_{85}-4s_{18}s_{83}-51664s_{19}s_{82}-51664s_{5}s_{97}-$
$51664s_{12}s_{90}+4s_{16}s_{86}-4s_{18}s_{84}-4s_{19}s_{83}-8s_{20}s_{82}-51664s_{5}s_{98}-51664s_{6}s_{97}-8s_{13}s_{90}+8s_{17}s_{86}+4s_{18}s_{85}-$
$51664s_{5}s_{99}-16s_{7}s_{97}-51664s_{13}s_{91}+4s_{18}s_{86}+8s_{21}s_{83}-51664s_{5}s_{100}-51664s_{7}s_{98}-8s_{8}s_{97}-8s_{14}s_{91}+4s_{19}s_{86}-$

$51664s_5s_{101} - 8s_8s_{98} - 8s_9s_{97} + 8s_{22}s_{84} - 51664s_5s_{102} - 51664s_8s_{99} - 4s_9s_{98} - 8s_{10}s_{97} - 51664s_{15}s_{92} - 8s_{21}s_{86} -$
$8s_{22}s_{85} - 8s_{23}s_{84} - 51664s_5s_94 - 4s_9s_{99} - 8s_{10}s_{98} - 8s_{11}s_{97} - 8s_{16}s_{92} + 8s_{23}s_{85} - 51664s_5s_{104} - 51664s_9s_{100} -$
$4s_{10}s_{99} - 8s_{11}s_{98} - 8s_{12}s_{97} - 51664s_{16}s_{93} - 51664s_5s_{105} - 8s_{10}s_{100} - 8s_{11}s_{99} - 8s_{12}s_{98} - 8s_{13}s_{97} - 8s_{17}s_{93} -$
$51664s_5s_{106} - 51664s_{10}s_{101} - 8s_{11}s_{100} - 8s_{12}s_{99} - 8s_{13}s_{98} - 8s_{14}s_{97} - 51664s_{17}s_{94} - 51664s_5s_{107} - 8s_{11}s_{101} -$
$4s_{12}s_{100} - 4s_{13}s_{99} - 4s_{14}s_{98} - 8s_{15}s_{97} - 8s_{18}s_{94} - 51664s_5s_{108} - 51664s_{11}s_{102} - 8s_{12}s_{101} + 4s_{13}s_{100} - 4s_{14}s_{99} -$
$4s_{15}s_{98} - 8s_{16}s_{97} - 51664s_{18}s_{95} - 51664s_5s_{109} - 8s_{12}s_{102} - 4s_{13}s_{101} + 4s_{14}s_{100} - 4s_{15}s_{99} - 8s_{16}s_{98} - 8s_{17}s_{97} -$
$8s_{19}s_{95} - 51664s_{12}s_94 - 8s_{13}s_{102} + 4s_{14}s_{101} - 4s_{16}s_{99} - 8s_{17}s_{98} - 8s_{18}s_{97} - 51664s_{19}s_{96} - 51664s_6s_{110} - 8s_{13}s_94 -$
$4s_{14}s_{102} - 8s_{17}s_{99} - 8s_{18}s_{98} - 8s_{19}s_{97} - 8s_{20}s_{96} - 51664s_6s_{111} - 51664s_7s_{110} - 51664s_{13}s_{104} - 8s_{14}s_94 - 4s_{18}s_{99} -$
$4s_{19}s_{98} - 4s_{20}s_{97} - 51664s_6s_{112} - 8s_{14}s_{104} - 4s_{19}s_{99} - 4s_{20}s_{98} + 8s_{21}s_{97} - 51664s_6s_{113} - 51664s_8s_{111} - 8s_9s_{110} -$
$4s_{20}s_{99} - 51664s_6s_{114} - 4s_9s_{111} - 8s_{10}s_{110} - 51664s_{15}s_{105} + 8s_{22}s_{98} - 51664s_6s_{115} - 51664s_9s_{112} - 8s_{10}s_{111} -$
$8s_{11}s_{110} - 8s_{16}s_{105} + 8s_{38}s_{83} - 51664s_6s_{116} - 8s_{10}s_{112} - 8s_{11}s_{111} - 8s_{12}s_{110} - 51664s_{16}s_{106} - 8s_{17}s_{105} + 8s_{23}s_{99} -$
$51664s_6s_{117} - 51664s_{10}s_{113} - 8s_{11}s_{112} - 8s_{12}s_{111} - 8s_{13}s_{110} - 8s_{17}s_{106} - 4s_{18}s_{105} + 8s_{39}s_{84} - 51664s_6s_{118} -$
$8s_{11}s_{113} - 8s_{13}s_{111} - 8s_{14}s_{110} - 51664s_{17}s_{107} - 8s_{18}s_{106} + 4s_{19}s_{105} - 8s_{38}s_{86} - 8s_{39}s_{85} - 51664s_6s_{119} -$
$51664s_{11}s_{114} - 8s_{12}s_{113} + 4s_{13}s_{112} - 4s_{14}s_{111} - 8s_{15}s_{110} - 8s_{18}s_{107} - 4s_{19}s_{106} + 4s_{20}s_{105} - 51664s_6s_{120} -$
$8s_{12}s_{114} - 4s_{15}s_{111} - 8s_{16}s_{110} - 51664s_{18}s_{108} - 8s_{19}s_{107} + 4s_{20}s_{106} - 51664s_6s_{121} - 51664s_{12}s_{115} - 8s_{13}s_{114} +$
$4s_{14}s_{113} - 8s_{16}s_{111} - 8s_{17}s_{110} - 8s_{19}s_{108} - 4s_{20}s_{107} - 8s_{13}s_{115} - 8s_{17}s_{111} - 8s_{18}s_{110} - 51664s_{19}s_{109} - 8s_{20}s_{108} -$
$51664s_7s_{122} - 51664s_{13}s_{116} - 8s_{14}s_{115} - 8s_{18}s_{111} - 8s_{19}s_{110} - 8s_{20}s_{109} - 51664s_7s_{123} - 51664s_8s_{122} - 8s_{14}s_{116} -$
$4s_{19}s_{111} - 4s_{20}s_{110} - 51664s_7s_{124} - 8s_9s_{122} - 4s_{20}s_{111} + 8s_{21}s_{110} - 51664s_7s_{125} - 51664s_9s_{123} - 8s_{10}s_{122} -$
$51664s_{15}s_{117} - 51664s_7s_{11}7 - 8s_{10}s_{123} - 8s_{11}s_{122} - 8s_{16}s_{117} + 8s_{22}s_{111} - 51664s_7s_{127} - 51664s_{10}s_{124} - 8s_{12}s_{122} -$
$51664s_{16}s_{118} - 8s_{17}s_{117} - 51664s_7s_{128} - 8s_{11}s_{124} - 8s_{13}s_{122} - 8s_{17}s_{118} + 8s_{38}s_{97} - 51664s_7s_{129} - 51664s_{11}s_{125} -$
$4s_{13}s_{123} - 8s_{14}s_{122} - 51664s_{17}s_{119} - 8s_{18}s_{118} + 4s_{19}s_{117} - 51664s_7s_{130} - 8s_{12}s_{125} - 8s_{15}s_{122} - 8s_{18}s_{119} + 8s_{39}s_{98} +$
$8s_{54}s_{83} - 51664s_7s_{131} - 51664s_{12}s_{11}7 - 4s_{14}s_{124} - 8s_{16}s_{122} - 51664s_{18}s_{120} - 8s_{19}s_{119} + 4s_{20}s_{118} - 51664s_7s_{132} -$
$8s_{13}s_{11}7 - 8s_{17}s_{122} - 8s_{19}s_{120} - 51664s_{13}s_{127} - 8s_{18}s_{122} - 51664s_{19}s_{121} - 8s_{20}s_{120} - 8s_{54}s_{86} - 51664s_8s_{133} -$
$8s_{14}s_{127} - 8s_{19}s_{122} - 8s_{20}s_{121} - 51664s_8s_{134} - 51664s_9s_{133} - 4s_{20}s_{122} - 51664s_8s_{135} - 51664s_{15}s_{128} + 8s_{21}s_{122} -$
$51664s_8s_{136} - 51664s_{10}s_{134} - 8s_{16}s_{128} - 51664s_8s_{137} - 4s_{12}s_{133} - 51664s_{16}s_{129} - 51664s_8s_{138} - 51664s_{11}s_{135} -$
$4s_{13}s_{133} - 8s_{17}s_{129} - 4s_{13}s_{134} - 4s_{14}s_{133} - 51664s_{17}s_{130} - 4s_{19}s_{128} - 51664s_9s_{139} - 4s_{14}s_{134} - 8s_{18}s_{130} + 8s_{38}s_{110} -$
$51664s_9s_{140} - 51664s_{10}s_{139} - 4s_{14}s_{135} - 51664s_{18}s_{131} - 4s_{20}s_{129} - 51664s_9s_{141} - 16s_{11}s_{139} - 8s_{19}s_{131} + 8s_{39}s_{111} -$
$51664s_9s_{142} - 51664s_{11}s_{140} - 16s_{12}s_{139} - 51664s_{15}s_{136} - 51664s_{19}s_{132} + 8s_{54}s_{97} - 51664s_9s_{143} - 24s_{12}s_{140} -$
$16s_{13}s_{139} - 8s_{20}s_{132} - 51664s_9s_{144} - 51664s_{12}s_{141} - 8s_{13}s_{140} - 16s_{14}s_{139} - 51664s_{16}s_{137} - 51664s_9s_{145} -$
$16s_{13}s_{141} - 8s_{14}s_{140} - 8s_{15}s_{139} - 4s_{18}s_{136} - 51664s_9s_{146} - 51664s_{13}s_{142} - 8s_{14}s_{141} - 4s_{15}s_{140} - 8s_{16}s_{139} -$
$51664s_{17}s_{138} - 4s_{19}s_{136} - 51664s_9s_{147} - 16s_{14}s_{142} - 4s_{15}s_{141} - 8s_{16}s_{140} - 8s_{17}s_{139} - 4s_{19}s_{137} - 4s_{20}s_{136} -$
$51664s_9s_{148} - 51664s_{14}s_{143} - 4s_{15}s_{142} - 4s_{16}s_{141} - 8s_{17}s_{140} - 8s_{18}s_{139} - 4s_{20}s_{137} - 4s_{15}s_{143} - 4s_{16}s_{142} - 8s_{17}s_{141} -$
$8s_{18}s_{140} - 8s_{19}s_{139} - 4s_{20}s_{138} - 51664s_{10}s_{149} - 51664s_{15}s_{144} - 4s_{18}s_{141} - 4s_{19}s_{140} - 4s_{20}s_{139} - 51664s_{10}s_{150} -$
$51664s_{11}s_{149} - 8s_{16}s_{144} - 4s_{18}s_{142} - 4s_{19}s_{141} - 4s_{20}s_{140} + 8s_{21}s_{139} + 8s_{38}s_{122} - 51664s_{10}s_{151} - 16s_{12}s_{149} -$
$51664s_{16}s_{145} - 4s_{17}s_{144} - 4s_{19}s_{142} - 4s_{20}s_{141} - 51664s_{10}s_{152} - 51664s_{12}s_{150} - 24s_{13}s_{149} - 8s_{17}s_{145} - 4s_{18}s_{144} -$
$4s_{19}s_{143} - 4s_{20}s_{142} + 8s_{22}s_{140} - 51664s_{10}s_{153} - 24s_{13}s_{150} - 16s_{14}s_{149} - 51664s_{17}s_{146} - 4s_{18}s_{145} - 4s_{19}s_{144} -$
$4s_{20}s_{143} - 51664s_{10}s_{154} - 51664s_{13}s_{151} - 8s_{14}s_{150} - 8s_{15}s_{149} - 8s_{18}s_{146} - 4s_{19}s_{145} - 4s_{20}s_{144} + 8s_{23}s_{141} +$
$8s_{54}s_{110} - 51664s_{10}s_{155} - 16s_{14}s_{151} - 4s_{15}s_{150} - 8s_{16}s_{149} - 51664s_{18}s_{147} - 4s_{19}s_{146} - 4s_{20}s_{145} - 51664s_{10}s_{156} -$
$51664s_{14}s_{152} - 4s_{15}s_{151} - 8s_{16}s_{150} - 8s_{17}s_{149} - 8s_{19}s_{147} - 4s_{20}s_{146} - 51664s_{10}s_{157} - 4s_{15}s_{152} - 4s_{16}s_{151} -$
$8s_{17}s_{150} - 8s_{18}s_{149} - 51664s_{19}s_{148} - 4s_{20}s_{147} - 51664s_{15}s_{153} - 4s_{16}s_{152} - 8s_{17}s_{151} - 8s_{18}s_{150} - 8s_{19}s_{149} - 8s_{20}s_{148} -$
$51664s_{11}s_{158} - 8s_{16}s_{153} - 4s_{18}s_{151} - 4s_{19}s_{150} - 4s_{20}s_{149} - 51664s_{11}s_{159} - 51664s_{12}s_{158} - 51664s_{16}s_{154} -$
$8s_{17}s_{153} - 4s_{18}s_{152} - 4s_{19}s_{151} - 4s_{20}s_{150} + 8s_{21}s_{149} - 51664s_{11}s_{160} - 16s_{13}s_{158} - 8s_{17}s_{154} - 4s_{18}s_{153} - 4s_{19}s_{152} -$
$4s_{20}s_{151} - 51664s_{11}s_{161} - 51664s_{13}s_{159} - 24s_{14}s_{158} - 51664s_{17}s_{155} - 8s_{18}s_{154} - 4s_{19}s_{153} - 4s_{20}s_{152} + 8s_{22}s_{150} -$
$51664s_{11}s_{162} - 16s_{14}s_{159} - 8s_{15}s_{158} - 8s_{18}s_{155} - 4s_{19}s_{154} - 4s_{20}s_{153} - 51664s_{11}s_{163} - 51664s_{14}s_{160} - 4s_{15}s_{159} -$
$8s_{16}s_{158} - 51664s_{18}s_{156} - 8s_{19}s_{155} - 4s_{20}s_{154} + 8s_{23}s_{151} - 51664s_{11}s_{164} - 4s_{15}s_{160} - 8s_{16}s_{159} - 8s_{17}s_{158} - 8s_{19}s_{156} -$
$4s_{20}s_{155} - 51664s_{11}s_{165} - 51664s_{15}s_{161} - 4s_{16}s_{160} - 8s_{17}s_{159} - 8s_{18}s_{158} - 51664s_{19}s_{157} - 8s_{20}s_{156} + 8s_{54}s_{122} -$
$8s_{16}s_{161} - 8s_{17}s_{160} - 8s_{18}s_{159} - 8s_{19}s_{158} - 8s_{20}s_{157} + 8s_{38}s_{139} - 51664s_{12}s_{166} - 51664s_{16}s_{162} - 8s_{17}s_{161} -$
$4s_{18}s_{160} - 4s_{19}s_{159} - 4s_{20}s_{158} - 51664s_{12}s_{167} - 51664s_{13}s_{166} - 8s_{17}s_{162} - 8s_{18}s_{161} - 4s_{19}s_{160} - 4s_{20}s_{159} +$

$8s_{21}s_{158}+8s_{39}s_{140}-51664s_{12}s_{168}-16s_{14}s_{166}-51664s_{17}s_{163}-8s_{18}s_{162}-4s_{19}s_{161}-4s_{20}s_{160}-51664s_{12}s_{169}-$
$51664s_{14}s_{167}-8s_{15}s_{166}-8s_{18}s_{163}-8s_{19}s_{162}+8s_{22}s_{159}-51664s_{12}s_{170}-4s_{15}s_{167}-8s_{16}s_{166}-51664s_{18}s_{164}-$
$8s_{19}s_{163}-4s_{20}s_{162}-51664s_{12}s_{171}-51664s_{15}s_{168}-8s_{16}s_{167}-8s_{17}s_{166}-8s_{19}s_{164}-8s_{20}s_{163}+8s_{23}s_{160}-$
$51664s_{12}s_{172}-8s_{16}s_{168}-8s_{17}s_{167}-8s_{18}s_{166}-51664s_{19}s_{165}-8s_{20}s_{164}-51664s_{16}s_{169}-8s_{17}s_{168}-8s_{18}s_{167}-$
$8s_{19}s_{166}-8s_{20}s_{165}-51664s_{13}s_{173}-8s_{17}s_{169}-8s_{18}s_{168}-4s_{19}s_{167}-4s_{20}s_{166}-51664s_{13}s_{174}-51664s_{14}s_{173}-$
$51664s_{17}s_{170}-8s_{18}s_{169}-4s_{19}s_{168}-4s_{20}s_{167}+8s_{21}s_{166}+8s_{38}s_{149}-51664s_{13}s_{175}-8s_{15}s_{173}-8s_{18}s_{170}-8s_{19}s_{169}-$
$51664s_{13}s_{176}-51664s_{15}s_{174}-8s_{16}s_{173}-51664s_{18}s_{171}-8s_{19}s_{170}-4s_{20}s_{169}+8s_{22}s_{167}+8s_{39}s_{150}-51664s_{13}s_{177}-$
$8s_{16}s_{174}-8s_{17}s_{173}-8s_{19}s_{171}-8s_{20}s_{170}-51664s_{13}s_{178}-51664s_{16}s_{175}-8s_{17}s_{174}-8s_{18}s_{173}-51664s_{19}s_{172}-$
$8s_{20}s_{171}-8s_{17}s_{175}-4s_{18}s_{174}-8s_{19}s_{173}-8s_{20}s_{172}-51664s_{14}s_{179}-51664s_{17}s_{176}-8s_{18}s_{175}-4s_{19}s_{174}-4s_{20}s_{173}+$
$8s_{54}s_{139}+16s_{83}s_{110}-51664s_{14}s_{180}-51664s_{15}s_{179}-8s_{18}s_{176}-4s_{19}s_{175}-4s_{20}s_{174}+8s_{21}s_{173}-16s_{83}s_{111}-$
$51664s_{14}s_{181}-8s_{16}s_{179}-51664s_{18}s_{177}-8s_{19}s_{176}-4s_{20}s_{175}-51664s_{14}s_{182}-51664s_{16}s_{180}-4s_{17}s_{179}-$
$8s_{19}s_{177}-4s_{20}s_{176}+8s_{38}s_{158}-51664s_{14}s_{183}-8s_{17}s_{180}-4s_{18}s_{179}-51664s_{19}s_{178}-8s_{20}s_{177}-51664s_{17}s_{181}-$
$4s_{18}s_{180}-4s_{19}s_{179}-8s_{20}s_{178}+8s_{39}s_{159}-51664s_{15}s_{184}-8s_{18}s_{181}-4s_{19}s_{180}-4s_{20}s_{179}-51664s_{15}s_{17}6-$
$51664s_{16}s_{184}-51664s_{18}s_{182}-4s_{19}s_{181}-4s_{20}s_{180}-51664s_{15}s_{186}-16s_{17}s_{184}-8s_{19}s_{182}-4s_{20}s_{181}-$
$51664s_{15}s_{187}-51664s_{17}s_{17}6-16s_{18}s_{184}-51664s_{19}s_{183}-4s_{20}s_{182}-51664s_{15}s_{188}-24s_{18}s_{17}6-16s_{19}s_{184}-$
$8s_{20}s_{183}+8s_{54}s_{149}-51664s_{18}s_{186}-8s_{20}s_{184}+8s_{38}s_{166}-51664s_{16}s_{189}-16s_{19}s_{186}-8s_{20}s_{17}6+8s_{21}s_{184}+$
$16s_{83}s_{122}-51664s_{16}s_{190}-51664s_{17}s_{189}-51664s_{19}s_{187}-8s_{20}s_{186}+8s_{39}s_{167}-16s_{84}s_{122}-51664s_{16}s_{191}-$
$16s_{18}s_{189}-16s_{20}s_{187}+8s_{22}s_{17}6-51664s_{16}s_{192}-51664s_{18}s_{190}-24s_{19}s_{189}-51664s_{20}s_{188}-16s_{19}s_{190}-$
$8s_{20}s_{189}+8s_{23}s_{186}-51664s_{17}s_{193}-51664s_{19}s_{191}-8s_{20}s_{190}+8s_{21}s_{189}-51664s_{17}s_{194}-51664s_{18}s_{193}-$
$16s_{20}s_{191}+8s_{38}s_{173}-51664s_{17}s_{195}-16s_{19}s_{193}-51664s_{20}s_{192}+8s_{22}s_{190}+8s_{54}s_{158}-51664s_{19}s_{194}-16s_{20}s_{193}-$
$51664s_{18}s_{196}-16s_{20}s_{194}+8s_{21}s_{193}+8s_{23}s_{191}-51664s_{18}s_{197}-51664s_{19}s_{196}-51664s_{20}s_{195}-8s_{20}s_{196}+$
$8s_{22}s_{194}-51664s_{19}s_{198}-51664s_{20}s_{197}+8s_{21}s_{196}-51664s_{20}s_{198}+8s_{23}s_{195}+8s_{21}s_{198}+8s_{22}s_{197}+16s_{97}s_{122}+$
$8s_{54}s_{166}+8s_{38}s_{184}+8s_{83}s_{139}+8s_{39}s_{17}6+8s_{84}s_{140}-8s_{83}s_{142}-8s_{84}s_{141}-8s_{85}s_{140}-8s_{86}s_{139}+8s_{85}s_{141}+8s_{38}s_{189}+$
$8s_{54}s_{173}+8s_{86}s_{142}+8s_{39}s_{190}+8s_{38}s_{193}+8s_{83}s_{149}+8s_{39}s_{194}+8s_{38}s_{196}+8s_{84}s_{150}-8s_{83}s_{152}-8s_{84}s_{151}-8s_{85}s_{150}-$
$8s_{86}s_{149}+8s_{38}s_{198}+8s_{39}s_{197}+8s_{85}s_{151}+8s_{97}s_{139}+8s_{54}s_{184}+8s_{86}s_{152}+8s_{98}s_{140}+8s_{99}s_{141}+8s_{83}s_{158}+8s_{54}s_{189}+$
$8s_{84}s_{159}-8s_{84}s_{160}-8s_{85}s_{159}-8s_{86}s_{158}+8s_{85}s_{160}+8s_{97}s_{149}+8s_{54}s_{193}+8s_{98}s_{150}+8s_{83}s_{166}+8s_{110}s_{139}+8s_{54}s_{196}+$
$8s_{99}s_{151}+8s_{84}s_{167}+8s_{111}s_{140}+8s_{54}s_{198}-8s_{85}s_{167}-8s_{86}s_{166}+8s_{97}s_{158}+8s_{83}s_{173}+8s_{98}s_{159}-8s_{86}s_{173}+8s_{99}s_{160}+$
$8s_{110}s_{149}+8s_{111}s_{150}+8s_{122}s_{139}+8s_{97}s_{166}+8s_{98}s_{167}+8s_{83}s_{184}+8s_{110}s_{158}+8s_{84}s_{17}6-8s_{83}s_{187}-8s_{84}s_{186}-$
$8s_{85}s_{17}6-8s_{86}s_{184}+8s_{97}s_{173}+8s_{111}s_{159}+8s_{85}s_{186}+8s_{122}s_{149}+8s_{83}s_{189}+8s_{86}s_{187}+8s_{84}s_{190}-8s_{83}s_{192}-$
$8s_{84}s_{191}-8s_{85}s_{190}-8s_{86}s_{189}+8s_{83}s_{193}+8s_{85}s_{191}+8s_{110}s_{166}+8s_{84}s_{194}+8s_{86}s_{192}+8s_{111}s_{167}+8s_{83}s_{196}-$
$8s_{84}s_{195}-8s_{85}s_{194}-8s_{86}s_{193}+8s_{85}s_{195}+8s_{122}s_{158}+8s_{83}s_{198}+8s_{84}s_{197}+8s_{97}s_{184}-8s_{85}s_{197}-8s_{86}s_{196}+8s_{98}s_{17}6+$
$8s_{110}s_{173}-8s_{86}s_{198}+8s_{99}s_{186}+8s_{97}s_{189}+8s_{98}s_{190}+8s_{122}s_{166}+8s_{97}s_{193}+8s_{99}s_{191}+8s_{98}s_{194}+8s_{97}s_{196}+8s_{99}s_{195}+$
$8s_{110}s_{184}+8s_{97}s_{198}+8s_{98}s_{197}+8s_{122}s_{173}+8s_{111}s_{17}6+16s_{139}s_{158}+8s_{110}s_{189}+8s_{111}s_{190}+8s_{110}s_{193}+8s_{111}s_{194}+$
$16s_{139}s_{166}+8s_{110}s_{196}+8s_{122}s_{184}+16s_{140}s_{167}+8s_{110}s_{198}+8s_{111}s_{197}+8s_{122}s_{189}+16s_{139}s_{173}+8s_{122}s_{193}+$
$16s_{149}s_{166}+8s_{122}s_{196}+8s_{122}s_{198}+16s_{149}s_{173}+8s_{139}s_{184}+8s_{140}s_{17}6+8s_{141}s_{186}+8s_{139}s_{189}+8s_{142}s_{187}+$
$8s_{140}s_{190}+8s_{143}s_{188}+16s_{158}s_{173}+8s_{139}s_{193}+8s_{141}s_{191}+8s_{149}s_{184}+8s_{140}s_{194}+8s_{142}s_{192}+8s_{139}s_{196}+8s_{150}s_{17}6+$
$8s_{141}s_{195}+8s_{139}s_{198}+8s_{140}s_{197}+8s_{151}s_{186}+8s_{149}s_{189}+8s_{152}s_{187}+8s_{150}s_{190}+8s_{149}s_{193}+8s_{151}s_{191}+8s_{158}s_{184}+$
$8s_{150}s_{194}+8s_{152}s_{192}+8s_{159}s_{17}6+8s_{149}s_{196}+8s_{151}s_{195}+8s_{160}s_{186}+8s_{149}s_{198}+8s_{150}s_{197}+8s_{158}s_{189}+8s_{159}s_{190}+$
$8s_{166}s_{184}+8s_{158}s_{193}+8s_{160}s_{191}+8s_{167}s_{17}6+8s_{159}s_{194}+8s_{158}s_{196}+8s_{160}s_{195}+8s_{166}s_{189}+8s_{158}s_{198}+8s_{159}s_{197}+$
$8s_{167}s_{190}+8s_{173}s_{184}+8s_{166}s_{193}+8s_{167}s_{194}+8s_{166}s_{196}+8s_{173}s_{189}+8s_{166}s_{198}+8s_{167}s_{197}+8s_{173}s_{193}+8s_{173}s_{196}+$
$8s_{173}s_{198}+16s_{184}s_{193}+16s_{184}s_{196}+16s_{184}s_{198}+16s_{17}6s_{197}+16s_{189}s_{196}+16s_{189}s_{198}+16s_{193}s_{198}+13798138$

A complete expression of $\hat{H}_2\left(\hat{\sigma}^z\right)$ in 92-order H matrix using Turyn based method with $\delta = 103,328$

$\hat{H}_2\left(\hat{\sigma}^z\right) = 439290\hat{\sigma}^z_0+439286\hat{\sigma}^z_1+439294\hat{\sigma}^z_2+439294\hat{\sigma}^z_3+465068\hat{\sigma}^z_4+465164\hat{\sigma}^z_5+465180\hat{\sigma}^z_6+465172\hat{\sigma}^z_7+$
$258382\hat{\sigma}^z_8+491030\hat{\sigma}^z_9+491138\hat{\sigma}^z_{10}+491214\hat{\sigma}^z_{11}+465440\hat{\sigma}^z_{12}+465476\hat{\sigma}^z_{13}+258726\hat{\sigma}^z_{14}+516956\hat{\sigma}^z_{15}+$
$517116\hat{\sigma}^z_{16}+517228\hat{\sigma}^z_{17}+491472\hat{\sigma}^z_{18}+491486\hat{\sigma}^z_{19}+129652\hat{\sigma}^z_{20}-51724\hat{\sigma}^z_{21}-51724\hat{\sigma}^z_{22}-51716\hat{\sigma}^z_{23}-$
$51680\hat{\sigma}^z_{24}-51680\hat{\sigma}^z_{25}-51676\hat{\sigma}^z_{26}-51676\hat{\sigma}^z_{27}-51672\hat{\sigma}^z_{28}-51676\hat{\sigma}^z_{29}-51688\hat{\sigma}^z_{30}-51692\hat{\sigma}^z_{31}-51692\hat{\sigma}^z_{32}-$

$51672\hat{\sigma}^z_{2}4 - 51680\hat{\sigma}^z_{34} - 51684\hat{\sigma}^z_{35} - 51688\hat{\sigma}^z_{36} - 51684\hat{\sigma}^z_{37} - 51724\hat{\sigma}^z_{38} - 51732\hat{\sigma}^z_{39} - 51680\hat{\sigma}^z_{40} - 51684\hat{\sigma}^z_{41} -$
$51684\hat{\sigma}^z_{42} - 51668\hat{\sigma}^z_{43} - 51672\hat{\sigma}^z_{44} - 51680\hat{\sigma}^z_{45} - 51684\hat{\sigma}^z_{46} - 51692\hat{\sigma}^z_{47} - 51692\hat{\sigma}^z_{48} - 51672\hat{\sigma}^z_{49} - 51680\hat{\sigma}^z_{50} -$
$51688\hat{\sigma}^z_{51} - 51684\hat{\sigma}^z_{52} - 51684\hat{\sigma}^z_{53} - 51748\hat{\sigma}^z_{54} - 51680\hat{\sigma}^z_{55} - 51676\hat{\sigma}^z_{56} - 51676\hat{\sigma}^z_{57} - 51676\hat{\sigma}^z_{58} - 51672\hat{\sigma}^z_{59} -$
$51680\hat{\sigma}^z_{60} - 51684\hat{\sigma}^z_{61} - 51684\hat{\sigma}^z_{62} - 51692\hat{\sigma}^z_{63} - 51672\hat{\sigma}^z_{64} - 51680\hat{\sigma}^z_{65} - 51680\hat{\sigma}^z_{66} - 51684\hat{\sigma}^z_{67} - 51684\hat{\sigma}^z_{68} -$
$51680\hat{\sigma}^z_{69} - 51664\hat{\sigma}^z_{70} - 51668\hat{\sigma}^z_{71} - 51676\hat{\sigma}^z_{72} - 51672\hat{\sigma}^z_{73} - 51676\hat{\sigma}^z_{74} - 51680\hat{\sigma}^z_{75} - 51684\hat{\sigma}^z_{76} - 51684\hat{\sigma}^z_{77} -$
$51672\hat{\sigma}^z_{78} - 51672\hat{\sigma}^z_{79} - 51676\hat{\sigma}^z_{80} - 51680\hat{\sigma}^z_{81} - 51684\hat{\sigma}^z_{82} - 51728\hat{\sigma}^z_{83} - 51696\hat{\sigma}^z_{84} - 51656\hat{\sigma}^z_{85} - 51608\hat{\sigma}^z_{86} -$
$51664\hat{\sigma}^z_{87} - 51672\hat{\sigma}^z_{88} - 51672\hat{\sigma}^z_{89} - 51672\hat{\sigma}^z_{90} - 51672\hat{\sigma}^z_{91} - 51672\hat{\sigma}^z_{92} - 51672\hat{\sigma}^z_{93} - 51672\hat{\sigma}^z_{94} - 51672\hat{\sigma}^z_{95} -$
$51672\hat{\sigma}^z_{96} - 51772\hat{\sigma}^z_{97} - 51732\hat{\sigma}^z_{98} - 51700\hat{\sigma}^z_{99} - 51672\hat{\sigma}^z_{100} - 51676\hat{\sigma}^z_{101} - 51680\hat{\sigma}^z_{102} - 51684\hat{\sigma}^z_{9}4 -$
$51684\hat{\sigma}^z_{104} - 51672\hat{\sigma}^z_{105} - 51680\hat{\sigma}^z_{106} - 51684\hat{\sigma}^z_{107} - 51680\hat{\sigma}^z_{108} - 51676\hat{\sigma}^z_{109} - 51772\hat{\sigma}^z_{110} - 51708\hat{\sigma}^z_{111} -$
$51672\hat{\sigma}^z_{112} - 51680\hat{\sigma}^z_{113} - 51676\hat{\sigma}^z_{114} - 51676\hat{\sigma}^z_{115} - 51684\hat{\sigma}^z_{116} - 51672\hat{\sigma}^z_{117} - 51680\hat{\sigma}^z_{118} - 51680\hat{\sigma}^z_{119} -$
$51676\hat{\sigma}^z_{120} - 51676\hat{\sigma}^z_{121} - 51780\hat{\sigma}^z_{122} - 51672\hat{\sigma}^z_{123} - 51672\hat{\sigma}^z_{124} - 51676\hat{\sigma}^z_{125} - 51676\hat{\sigma}^z_{11}7 - 51676\hat{\sigma}^z_{127} -$
$51672\hat{\sigma}^z_{128} - 51672\hat{\sigma}^z_{129} - 51672\hat{\sigma}^z_{130} - 51676\hat{\sigma}^z_{131} - 51676\hat{\sigma}^z_{132} - 51664\hat{\sigma}^z_{133} - 51668\hat{\sigma}^z_{134} - 51672\hat{\sigma}^z_{135} -$
$51664\hat{\sigma}^z_{136} - 51664\hat{\sigma}^z_{137} - 51668\hat{\sigma}^z_{138} - 51796\hat{\sigma}^z_{139} - 51756\hat{\sigma}^z_{140} - 51724\hat{\sigma}^z_{141} - 51708\hat{\sigma}^z_{142} - 51708\hat{\sigma}^z_{143} -$
$51672\hat{\sigma}^z_{144} - 51676\hat{\sigma}^z_{145} - 51680\hat{\sigma}^z_{146} - 51684\hat{\sigma}^z_{147} - 51688\hat{\sigma}^z_{148} - 51796\hat{\sigma}^z_{149} - 51748\hat{\sigma}^z_{150} - 51748\hat{\sigma}^z_{151} -$
$51724\hat{\sigma}^z_{152} - 51672\hat{\sigma}^z_{153} - 51680\hat{\sigma}^z_{154} - 51684\hat{\sigma}^z_{155} - 51688\hat{\sigma}^z_{156} - 51692\hat{\sigma}^z_{157} - 51820\hat{\sigma}^z_{158} - 51764\hat{\sigma}^z_{159} -$
$51748\hat{\sigma}^z_{160} - 51672\hat{\sigma}^z_{161} - 51680\hat{\sigma}^z_{162} - 51688\hat{\sigma}^z_{163} - 51692\hat{\sigma}^z_{164} - 51692\hat{\sigma}^z_{165} - 51836\hat{\sigma}^z_{166} - 51796\hat{\sigma}^z_{167} -$
$51672\hat{\sigma}^z_{168} - 51680\hat{\sigma}^z_{169} - 51688\hat{\sigma}^z_{170} - 51692\hat{\sigma}^z_{171} - 51692\hat{\sigma}^z_{172} - 51860\hat{\sigma}^z_{173} - 51672\hat{\sigma}^z_{174} - 51680\hat{\sigma}^z_{175} -$
$51684\hat{\sigma}^z_{176} - 51688\hat{\sigma}^z_{177} - 51692\hat{\sigma}^z_{178} - 51672\hat{\sigma}^z_{179} - 51676\hat{\sigma}^z_{180} - 51680\hat{\sigma}^z_{181} - 51684\hat{\sigma}^z_{182} - 51688\hat{\sigma}^z_{183} -$
$51844\hat{\sigma}^z_{184} - 51796\hat{\sigma}^z_{17}6 - 51756\hat{\sigma}^z_{186} - 51724\hat{\sigma}^z_{187} - 51708\hat{\sigma}^z_{188} - 51844\hat{\sigma}^z_{189} - 51788\hat{\sigma}^z_{190} - 51764\hat{\sigma}^z_{191} -$
$51732\hat{\sigma}^z_{192} - 51860\hat{\sigma}^z_{193} - 51796\hat{\sigma}^z_{194} - 51764\hat{\sigma}^z_{195} - 51876\hat{\sigma}^z_{196} - 51820\hat{\sigma}^z_{197} - 51900\hat{\sigma}^z_{198} + 25832\hat{\sigma}^z_{0}\hat{\sigma}^z_{1} +$
$25832\hat{\sigma}^z_{0}\hat{\sigma}^z_{2} + 25832\hat{\sigma}^z_{0}\hat{\sigma}^z_{3} + 25832\hat{\sigma}^z_{1}\hat{\sigma}^z_{2} + 25832\hat{\sigma}^z_{0}\hat{\sigma}^z_{4} + 25832\hat{\sigma}^z_{1}\hat{\sigma}^z_{3} + 25832\hat{\sigma}^z_{0}\hat{\sigma}^z_{5} + 25832\hat{\sigma}^z_{1}\hat{\sigma}^z_{4} + 25832\hat{\sigma}^z_{2}\hat{\sigma}^z_{3} +$
$25832\hat{\sigma}^z_{0}\hat{\sigma}^z_{6} + 25832\hat{\sigma}^z_{1}\hat{\sigma}^z_{5} + 25832\hat{\sigma}^z_{2}\hat{\sigma}^z_{4} + 25832\hat{\sigma}^z_{0}\hat{\sigma}^z_{7} + 25832\hat{\sigma}^z_{1}\hat{\sigma}^z_{6} + 25832\hat{\sigma}^z_{2}\hat{\sigma}^z_{5} + 25832\hat{\sigma}^z_{3}\hat{\sigma}^z_{4} + 2\hat{\sigma}^z_{0}\hat{\sigma}^z_{8} +$
$25832\hat{\sigma}^z_{1}\hat{\sigma}^z_{7} + 25832\hat{\sigma}^z_{2}\hat{\sigma}^z_{6} + 25832\hat{\sigma}^z_{3}\hat{\sigma}^z_{5} + 25832\hat{\sigma}^z_{0}\hat{\sigma}^z_{9} + 2\hat{\sigma}^z_{1}\hat{\sigma}^z_{8} + 25832\hat{\sigma}^z_{2}\hat{\sigma}^z_{7} + 25832\hat{\sigma}^z_{3}\hat{\sigma}^z_{6} + 25832\hat{\sigma}^z_{4}\hat{\sigma}^z_{5} +$
$25832\hat{\sigma}^z_{0}\hat{\sigma}^z_{10} + 25832\hat{\sigma}^z_{1}\hat{\sigma}^z_{9} + 2\hat{\sigma}^z_{2}\hat{\sigma}^z_{8} + 25832\hat{\sigma}^z_{3}\hat{\sigma}^z_{7} + 25832\hat{\sigma}^z_{4}\hat{\sigma}^z_{6} + 25832\hat{\sigma}^z_{0}\hat{\sigma}^z_{11} + 25832\hat{\sigma}^z_{1}\hat{\sigma}^z_{10} + 25832\hat{\sigma}^z_{2}\hat{\sigma}^z_{9} +$
$6\hat{\sigma}^z_{3}\hat{\sigma}^z_{8} + 25832\hat{\sigma}^z_{4}\hat{\sigma}^z_{7} + 25832\hat{\sigma}^z_{5}\hat{\sigma}^z_{6} + 25832\hat{\sigma}^z_{0}\hat{\sigma}^z_{12} + 25832\hat{\sigma}^z_{1}\hat{\sigma}^z_{11} + 25832\hat{\sigma}^z_{2}\hat{\sigma}^z_{10} + 25832\hat{\sigma}^z_{3}\hat{\sigma}^z_{9} + 25832\hat{\sigma}^z_{4}\hat{\sigma}^z_{8} +$
$25832\hat{\sigma}^z_{5}\hat{\sigma}^z_{7} + 25832\hat{\sigma}^z_{0}\hat{\sigma}^z_{13} + 25832\hat{\sigma}^z_{1}\hat{\sigma}^z_{12} + 25832\hat{\sigma}^z_{2}\hat{\sigma}^z_{11} + 25832\hat{\sigma}^z_{3}\hat{\sigma}^z_{10} + 25832\hat{\sigma}^z_{4}\hat{\sigma}^z_{9} + 25832\hat{\sigma}^z_{5}\hat{\sigma}^z_{8} + 25832\hat{\sigma}^z_{6}\hat{\sigma}^z_{7} +$
$14\hat{\sigma}^z_{0}\hat{\sigma}^z_{14} + 25832\hat{\sigma}^z_{1}\hat{\sigma}^z_{13} + 25832\hat{\sigma}^z_{2}\hat{\sigma}^z_{12} + 25832\hat{\sigma}^z_{3}\hat{\sigma}^z_{11} + 25832\hat{\sigma}^z_{4}\hat{\sigma}^z_{10} + 25832\hat{\sigma}^z_{5}\hat{\sigma}^z_{9} + 25832\hat{\sigma}^z_{6}\hat{\sigma}^z_{8} + 25832\hat{\sigma}^z_{0}\hat{\sigma}^z_{15} +$
$14\hat{\sigma}^z_{1}\hat{\sigma}^z_{14} + 25832\hat{\sigma}^z_{2}\hat{\sigma}^z_{13} + 25832\hat{\sigma}^z_{3}\hat{\sigma}^z_{12} + 25832\hat{\sigma}^z_{4}\hat{\sigma}^z_{11} + 25832\hat{\sigma}^z_{5}\hat{\sigma}^z_{10} + 25832\hat{\sigma}^z_{6}\hat{\sigma}^z_{9} + 25832\hat{\sigma}^z_{7}\hat{\sigma}^z_{8} + 25832\hat{\sigma}^z_{0}\hat{\sigma}^z_{16} +$
$25832\hat{\sigma}^z_{1}\hat{\sigma}^z_{15} + 14\hat{\sigma}^z_{2}\hat{\sigma}^z_{14} + 25832\hat{\sigma}^z_{3}\hat{\sigma}^z_{13} + 25832\hat{\sigma}^z_{4}\hat{\sigma}^z_{12} + 25832\hat{\sigma}^z_{5}\hat{\sigma}^z_{11} + 25832\hat{\sigma}^z_{6}\hat{\sigma}^z_{10} + 25832\hat{\sigma}^z_{7}\hat{\sigma}^z_{9} + 25832\hat{\sigma}^z_{0}\hat{\sigma}^z_{17} +$
$25832\hat{\sigma}^z_{1}\hat{\sigma}^z_{16} + 25832\hat{\sigma}^z_{2}\hat{\sigma}^z_{15} + 14\hat{\sigma}^z_{3}\hat{\sigma}^z_{14} + 25832\hat{\sigma}^z_{4}\hat{\sigma}^z_{13} + 25832\hat{\sigma}^z_{5}\hat{\sigma}^z_{12} + 25832\hat{\sigma}^z_{6}\hat{\sigma}^z_{11} + 25832\hat{\sigma}^z_{7}\hat{\sigma}^z_{10} + 25832\hat{\sigma}^z_{8}\hat{\sigma}^z_{9} +$
$25832\hat{\sigma}^z_{0}\hat{\sigma}^z_{18} + 25832\hat{\sigma}^z_{1}\hat{\sigma}^z_{17} + 25832\hat{\sigma}^z_{2}\hat{\sigma}^z_{16} + 25832\hat{\sigma}^z_{3}\hat{\sigma}^z_{15} + 4\hat{\sigma}^z_{4}\hat{\sigma}^z_{14} + 25832\hat{\sigma}^z_{5}\hat{\sigma}^z_{13} + 25832\hat{\sigma}^z_{6}\hat{\sigma}^z_{12} + 25832\hat{\sigma}^z_{7}\hat{\sigma}^z_{11} +$
$25832\hat{\sigma}^z_{8}\hat{\sigma}^z_{10} + 25832\hat{\sigma}^z_{0}\hat{\sigma}^z_{19} + 25832\hat{\sigma}^z_{1}\hat{\sigma}^z_{18} + 25832\hat{\sigma}^z_{2}\hat{\sigma}^z_{17} + 25832\hat{\sigma}^z_{3}\hat{\sigma}^z_{16} + 25832\hat{\sigma}^z_{4}\hat{\sigma}^z_{15} + 10\hat{\sigma}^z_{5}\hat{\sigma}^z_{14} + 25832\hat{\sigma}^z_{6}\hat{\sigma}^z_{13} +$
$25832\hat{\sigma}^z_{7}\hat{\sigma}^z_{12} + 25832\hat{\sigma}^z_{8}\hat{\sigma}^z_{11} + 25832\hat{\sigma}^z_{9}\hat{\sigma}^z_{10} + 10\hat{\sigma}^z_{0}\hat{\sigma}^z_{20} + 25832\hat{\sigma}^z_{1}\hat{\sigma}^z_{19} + 25832\hat{\sigma}^z_{2}\hat{\sigma}^z_{18} + 25832\hat{\sigma}^z_{3}\hat{\sigma}^z_{17} + 25832\hat{\sigma}^z_{4}\hat{\sigma}^z_{16} +$
$25832\hat{\sigma}^z_{5}\hat{\sigma}^z_{15} + 10\hat{\sigma}^z_{6}\hat{\sigma}^z_{14} + 25832\hat{\sigma}^z_{7}\hat{\sigma}^z_{13} + 6\hat{\sigma}^z_{8}\hat{\sigma}^z_{12} + 25832\hat{\sigma}^z_{9}\hat{\sigma}^z_{11} - 51664\hat{\sigma}^z_{0}\hat{\sigma}^z_{21} + 10\hat{\sigma}^z_{1}\hat{\sigma}^z_{20} + 25832\hat{\sigma}^z_{2}\hat{\sigma}^z_{19} +$
$25832\hat{\sigma}^z_{3}\hat{\sigma}^z_{18} + 25832\hat{\sigma}^z_{4}\hat{\sigma}^z_{17} + 25832\hat{\sigma}^z_{5}\hat{\sigma}^z_{16} + 25832\hat{\sigma}^z_{6}\hat{\sigma}^z_{15} + 10\hat{\sigma}^z_{7}\hat{\sigma}^z_{14} + 6\hat{\sigma}^z_{8}\hat{\sigma}^z_{13} + 25832\hat{\sigma}^z_{9}\hat{\sigma}^z_{12} + 25832\hat{\sigma}^z_{10}\hat{\sigma}^z_{11} -$
$51664\hat{\sigma}^z_{0}\hat{\sigma}^z_{22} - 51664\hat{\sigma}^z_{1}\hat{\sigma}^z_{21} + 10\hat{\sigma}^z_{2}\hat{\sigma}^z_{20} + 25832\hat{\sigma}^z_{3}\hat{\sigma}^z_{19} + 25832\hat{\sigma}^z_{4}\hat{\sigma}^z_{18} + 25832\hat{\sigma}^z_{5}\hat{\sigma}^z_{17} + 25832\hat{\sigma}^z_{6}\hat{\sigma}^z_{16} + 25832\hat{\sigma}^z_{7}\hat{\sigma}^z_{15} +$
$6\hat{\sigma}^z_{8}\hat{\sigma}^z_{14} + 25832\hat{\sigma}^z_{9}\hat{\sigma}^z_{13} + 25832\hat{\sigma}^z_{10}\hat{\sigma}^z_{12} - 51664\hat{\sigma}^z_{0}\hat{\sigma}^z_{23} - 16\hat{\sigma}^z_{2}\hat{\sigma}^z_{21} + 10\hat{\sigma}^z_{3}\hat{\sigma}^z_{20} + 25832\hat{\sigma}^z_{4}\hat{\sigma}^z_{19} + 25832\hat{\sigma}^z_{5}\hat{\sigma}^z_{18} +$
$25832\hat{\sigma}^z_{6}\hat{\sigma}^z_{17} + 25832\hat{\sigma}^z_{7}\hat{\sigma}^z_{16} + 25832\hat{\sigma}^z_{8}\hat{\sigma}^z_{15} + 25832\hat{\sigma}^z_{9}\hat{\sigma}^z_{14} + 25832\hat{\sigma}^z_{10}\hat{\sigma}^z_{13} + 25832\hat{\sigma}^z_{11}\hat{\sigma}^z_{12} - 51664\hat{\sigma}^z_{0}\hat{\sigma}^z_{24} -$
$51664\hat{\sigma}^z_{2}\hat{\sigma}^z_{22} - 24\hat{\sigma}^z_{3}\hat{\sigma}^z_{21} + 25832\hat{\sigma}^z_{5}\hat{\sigma}^z_{19} + 25832\hat{\sigma}^z_{6}\hat{\sigma}^z_{18} + 25832\hat{\sigma}^z_{7}\hat{\sigma}^z_{17} + 25832\hat{\sigma}^z_{8}\hat{\sigma}^z_{16} + 25832\hat{\sigma}^z_{9}\hat{\sigma}^z_{15} + 25832\hat{\sigma}^z_{10}\hat{\sigma}^z_{14} +$
$25832\hat{\sigma}^z_{11}\hat{\sigma}^z_{13} - 51664\hat{\sigma}^z_{0}\hat{\sigma}^z_{25} - 8\hat{\sigma}^z_{3}\hat{\sigma}^z_{22} - 8\hat{\sigma}^z_{4}\hat{\sigma}^z_{21} + 6\hat{\sigma}^z_{5}\hat{\sigma}^z_{20} + 25832\hat{\sigma}^z_{6}\hat{\sigma}^z_{19} + 25832\hat{\sigma}^z_{7}\hat{\sigma}^z_{18} + 25832\hat{\sigma}^z_{8}\hat{\sigma}^z_{17} +$
$25832\hat{\sigma}^z_{9}\hat{\sigma}^z_{16} + 25832\hat{\sigma}^z_{10}\hat{\sigma}^z_{15} + 25832\hat{\sigma}^z_{11}\hat{\sigma}^z_{14} + 25832\hat{\sigma}^z_{12}\hat{\sigma}^z_{13} - 51664\hat{\sigma}^z_{0}\hat{\sigma}^z_{26} - 51664\hat{\sigma}^z_{3}\hat{\sigma}^z_{23} - 8\hat{\sigma}^z_{5}\hat{\sigma}^z_{21} + 6\hat{\sigma}^z_{6}\hat{\sigma}^z_{20} +$
$25832\hat{\sigma}^z_{7}\hat{\sigma}^z_{19} + 4\hat{\sigma}^z_{8}\hat{\sigma}^z_{18} + 25832\hat{\sigma}^z_{9}\hat{\sigma}^z_{17} + 25832\hat{\sigma}^z_{10}\hat{\sigma}^z_{16} + 25832\hat{\sigma}^z_{11}\hat{\sigma}^z_{15} + 25832\hat{\sigma}^z_{12}\hat{\sigma}^z_{14} - 51664\hat{\sigma}^z_{0}\hat{\sigma}^z_{27} - 8\hat{\sigma}^z_{5}\hat{\sigma}^z_{22} -$
$8\hat{\sigma}^z_{6}\hat{\sigma}^z_{21} + 6\hat{\sigma}^z_{7}\hat{\sigma}^z_{20} + 6\hat{\sigma}^z_{8}\hat{\sigma}^z_{19} + 25832\hat{\sigma}^z_{9}\hat{\sigma}^z_{18} + 25832\hat{\sigma}^z_{10}\hat{\sigma}^z_{17} + 25832\hat{\sigma}^z_{11}\hat{\sigma}^z_{16} + 25832\hat{\sigma}^z_{12}\hat{\sigma}^z_{15} + 25832\hat{\sigma}^z_{13}\hat{\sigma}^z_{14} -$
$51664\hat{\sigma}^z_{0}\hat{\sigma}^z_{28} - 51664\hat{\sigma}^z_{4}\hat{\sigma}^z_{24} - 4\hat{\sigma}^z_{5}\hat{\sigma}^z_{23} - 8\hat{\sigma}^z_{6}\hat{\sigma}^z_{22} - 8\hat{\sigma}^z_{7}\hat{\sigma}^z_{21} + 6\hat{\sigma}^z_{8}\hat{\sigma}^z_{20} + 25832\hat{\sigma}^z_{9}\hat{\sigma}^z_{19} + 25832\hat{\sigma}^z_{10}\hat{\sigma}^z_{18} + 25832\hat{\sigma}^z_{11}\hat{\sigma}^z_{17} +$
$25832\hat{\sigma}^z_{12}\hat{\sigma}^z_{16} + 25832\hat{\sigma}^z_{13}\hat{\sigma}^z_{15} - 51664\hat{\sigma}^z_{0}\hat{\sigma}^z_{29} - 4\hat{\sigma}^z_{5}\hat{\sigma}^z_{24} + 14\hat{\sigma}^z_{9}\hat{\sigma}^z_{20} + 25832\hat{\sigma}^z_{10}\hat{\sigma}^z_{19} + 25832\hat{\sigma}^z_{11}\hat{\sigma}^z_{18} + 25832\hat{\sigma}^z_{12}\hat{\sigma}^z_{17} +$
$25832\hat{\sigma}^z_{13}\hat{\sigma}^z_{16} + 25832\hat{\sigma}^z_{14}\hat{\sigma}^z_{15} - 51664\hat{\sigma}^z_{0}\hat{\sigma}^z_{30} - 51664\hat{\sigma}^z_{5}\hat{\sigma}^z_{25} - 4\hat{\sigma}^z_{6}\hat{\sigma}^z_{24} - 8\hat{\sigma}^z_{9}\hat{\sigma}^z_{21} + 14\hat{\sigma}^z_{10}\hat{\sigma}^z_{20} + 25832\hat{\sigma}^z_{11}\hat{\sigma}^z_{19} +$
$25832\hat{\sigma}^z_{12}\hat{\sigma}^z_{18} + 25832\hat{\sigma}^z_{13}\hat{\sigma}^z_{17} + 25832\hat{\sigma}^z_{14}\hat{\sigma}^z_{16} - 51664\hat{\sigma}^z_{0}\hat{\sigma}^z_{31} - 8\hat{\sigma}^z_{6}\hat{\sigma}^z_{25} + 4\hat{\sigma}^z_{7}\hat{\sigma}^z_{24} - 4\hat{\sigma}^z_{8}\hat{\sigma}^z_{23} - 4\hat{\sigma}^z_{9}\hat{\sigma}^z_{22} - 8\hat{\sigma}^z_{10}\hat{\sigma}^z_{21} +$
$14\hat{\sigma}^z_{11}\hat{\sigma}^z_{20} + 25832\hat{\sigma}^z_{12}\hat{\sigma}^z_{19} + 25832\hat{\sigma}^z_{13}\hat{\sigma}^z_{18} + 25832\hat{\sigma}^z_{14}\hat{\sigma}^z_{17} + 25832\hat{\sigma}^z_{15}\hat{\sigma}^z_{16} - 51664\hat{\sigma}^z_{0}\hat{\sigma}^z_{32} - 51664\hat{\sigma}^z_{6}\hat{\sigma}^z_{26} -$

$8\hat\sigma^z_{7}\hat\sigma^z_{25}+4\hat\sigma^z_{8}\hat\sigma^z_{24}-4\hat\sigma^z_{9}\hat\sigma^z_{23}-8\hat\sigma^z_{10}\hat\sigma^z_{22}-8\hat\sigma^z_{11}\hat\sigma^z_{21}+14\hat\sigma^z_{12}\hat\sigma^z_{20}+25832\hat\sigma^z_{13}\hat\sigma^z_{19}+25832\hat\sigma^z_{14}\hat\sigma^z_{18}+25832\hat\sigma^z_{15}\hat\sigma^z_{17}-$
$51664\hat\sigma^z_{0}\hat\sigma^z_{2}4-8\hat\sigma^z_{7}\hat\sigma^z_{26}-4\hat\sigma^z_{8}\hat\sigma^z_{25}-4\hat\sigma^z_{10}\hat\sigma^z_{23}-8\hat\sigma^z_{11}\hat\sigma^z_{22}-8\hat\sigma^z_{12}\hat\sigma^z_{21}+14\hat\sigma^z_{13}\hat\sigma^z_{20}+25832\hat\sigma^z_{14}\hat\sigma^z_{19}+25832\hat\sigma^z_{15}\hat\sigma^z_{18}+$
$25832\hat\sigma^z_{16}\hat\sigma^z_{17}-51664\hat\sigma^z_{0}\hat\sigma^z_{34}-51664\hat\sigma^z_{7}\hat\sigma^z_{27}-8\hat\sigma^z_{8}\hat\sigma^z_{26}-8\hat\sigma^z_{11}\hat\sigma^z_{23}-8\hat\sigma^z_{12}\hat\sigma^z_{22}-8\hat\sigma^z_{13}\hat\sigma^z_{21}+14\hat\sigma^z_{14}\hat\sigma^z_{20}+25832\hat\sigma^z_{15}\hat\sigma^z_{19}+$
$25832\hat\sigma^z_{16}\hat\sigma^z_{18}-51664\hat\sigma^z_{0}\hat\sigma^z_{35}-8\hat\sigma^z_{8}\hat\sigma^z_{27}-8\hat\sigma^z_{12}\hat\sigma^z_{23}-8\hat\sigma^z_{13}\hat\sigma^z_{22}-8\hat\sigma^z_{14}\hat\sigma^z_{21}+25832\hat\sigma^z_{15}\hat\sigma^z_{20}+25832\hat\sigma^z_{16}\hat\sigma^z_{19}+$
$25832\hat\sigma^z_{17}\hat\sigma^z_{18}-51664\hat\sigma^z_{0}\hat\sigma^z_{36}-4\hat\sigma^z_{13}\hat\sigma^z_{23}-4\hat\sigma^z_{14}\hat\sigma^z_{22}-8\hat\sigma^z_{15}\hat\sigma^z_{21}+25832\hat\sigma^z_{16}\hat\sigma^z_{20}+25832\hat\sigma^z_{17}\hat\sigma^z_{19}-51664\hat\sigma^z_{0}\hat\sigma^z_{37}-$
$51664\hat\sigma^z_{9}\hat\sigma^z_{28}-4\hat\sigma^z_{14}\hat\sigma^z_{23}-4\hat\sigma^z_{15}\hat\sigma^z_{22}-8\hat\sigma^z_{16}\hat\sigma^z_{21}+25832\hat\sigma^z_{17}\hat\sigma^z_{20}+25832\hat\sigma^z_{18}\hat\sigma^z_{19}-8\hat\sigma^z_{10}\hat\sigma^z_{28}-4\hat\sigma^z_{15}\hat\sigma^z_{23}-8\hat\sigma^z_{16}\hat\sigma^z_{22}-$
$8\hat\sigma^z_{17}\hat\sigma^z_{21}+25832\hat\sigma^z_{18}\hat\sigma^z_{20}-51664\hat\sigma^z_{1}\hat\sigma^z_{38}-51664\hat\sigma^z_{10}\hat\sigma^z_{29}-8\hat\sigma^z_{11}\hat\sigma^z_{28}-4\hat\sigma^z_{16}\hat\sigma^z_{23}-8\hat\sigma^z_{17}\hat\sigma^z_{22}-8\hat\sigma^z_{18}\hat\sigma^z_{21}+25832\hat\sigma^z_{19}\hat\sigma^z_{20}-$
$51664\hat\sigma^z_{1}\hat\sigma^z_{39}-51664\hat\sigma^z_{2}\hat\sigma^z_{38}-8\hat\sigma^z_{11}\hat\sigma^z_{29}-4\hat\sigma^z_{12}\hat\sigma^z_{28}-8\hat\sigma^z_{17}\hat\sigma^z_{23}-8\hat\sigma^z_{18}\hat\sigma^z_{22}-8\hat\sigma^z_{19}\hat\sigma^z_{21}-51664\hat\sigma^z_{1}\hat\sigma^z_{40}-16\hat\sigma^z_{3}\hat\sigma^z_{38}-$
$51664\hat\sigma^z_{11}\hat\sigma^z_{30}-8\hat\sigma^z_{12}\hat\sigma^z_{29}-4\hat\sigma^z_{13}\hat\sigma^z_{28}-4\hat\sigma^z_{18}\hat\sigma^z_{23}-4\hat\sigma^z_{19}\hat\sigma^z_{22}-4\hat\sigma^z_{20}\hat\sigma^z_{21}-51664\hat\sigma^z_{1}\hat\sigma^z_{41}-51664\hat\sigma^z_{3}\hat\sigma^z_{39}-8\hat\sigma^z_{4}\hat\sigma^z_{38}-$
$8\hat\sigma^z_{12}\hat\sigma^z_{30}-4\hat\sigma^z_{13}\hat\sigma^z_{29}+4\hat\sigma^z_{14}\hat\sigma^z_{28}-4\hat\sigma^z_{19}\hat\sigma^z_{23}-4\hat\sigma^z_{20}\hat\sigma^z_{22}-51664\hat\sigma^z_{1}\hat\sigma^z_{42}-8\hat\sigma^z_{5}\hat\sigma^z_{38}-51664\hat\sigma^z_{12}\hat\sigma^z_{31}-8\hat\sigma^z_{13}\hat\sigma^z_{30}-4\hat\sigma^z_{14}\hat\sigma^z_{29}-$
$4\hat\sigma^z_{20}\hat\sigma^z_{23}-51664\hat\sigma^z_{1}\hat\sigma^z_{43}-51664\hat\sigma^z_{4}\hat\sigma^z_{40}-8\hat\sigma^z_{5}\hat\sigma^z_{39}-8\hat\sigma^z_{6}\hat\sigma^z_{38}-8\hat\sigma^z_{13}\hat\sigma^z_{31}-4\hat\sigma^z_{14}\hat\sigma^z_{30}-51664\hat\sigma^z_{1}\hat\sigma^z_{44}-12\hat\sigma^z_{5}\hat\sigma^z_{40}-$
$8\hat\sigma^z_{6}\hat\sigma^z_{39}-8\hat\sigma^z_{7}\hat\sigma^z_{38}-51664\hat\sigma^z_{13}\hat\sigma^z_{32}-8\hat\sigma^z_{14}\hat\sigma^z_{31}-51664\hat\sigma^z_{1}\hat\sigma^z_{45}-51664\hat\sigma^z_{5}\hat\sigma^z_{41}-8\hat\sigma^z_{14}\hat\sigma^z_{32}-51664\hat\sigma^z_{1}\hat\sigma^z_{46}-8\hat\sigma^z_{6}\hat\sigma^z_{41}-$
$8\hat\sigma^z_{9}\hat\sigma^z_{38}-51664\hat\sigma^z_{1}\hat\sigma^z_{47}-51664\hat\sigma^z_{6}\hat\sigma^z_{42}-8\hat\sigma^z_{7}\hat\sigma^z_{41}+12\hat\sigma^z_{8}\hat\sigma^z_{40}-4\hat\sigma^z_{9}\hat\sigma^z_{39}-8\hat\sigma^z_{10}\hat\sigma^z_{38}-51664\hat\sigma^z_{15}\hat\sigma^z_{2}4-51664\hat\sigma^z_{1}\hat\sigma^z_{48}-$
$8\hat\sigma^z_{7}\hat\sigma^z_{42}-8\hat\sigma^z_{8}\hat\sigma^z_{41}-8\hat\sigma^z_{10}\hat\sigma^z_{39}-8\hat\sigma^z_{11}\hat\sigma^z_{38}-8\hat\sigma^z_{16}\hat\sigma^z_{2}4-51664\hat\sigma^z_{1}\hat\sigma^z_{49}-51664\hat\sigma^z_{7}\hat\sigma^z_{43}-8\hat\sigma^z_{8}\hat\sigma^z_{42}-8\hat\sigma^z_{11}\hat\sigma^z_{39}-8\hat\sigma^z_{12}\hat\sigma^z_{38}-$
$51664\hat\sigma^z_{16}\hat\sigma^z_{34}-8\hat\sigma^z_{17}\hat\sigma^z_{2}4-51664\hat\sigma^z_{1}\hat\sigma^z_{50}-8\hat\sigma^z_{8}\hat\sigma^z_{43}-8\hat\sigma^z_{12}\hat\sigma^z_{39}-8\hat\sigma^z_{13}\hat\sigma^z_{38}-8\hat\sigma^z_{17}\hat\sigma^z_{34}-4\hat\sigma^z_{18}\hat\sigma^z_{2}4-51664\hat\sigma^z_{1}\hat\sigma^z_{51}-$
$8\hat\sigma^z_{13}\hat\sigma^z_{39}-8\hat\sigma^z_{14}\hat\sigma^z_{38}-51664\hat\sigma^z_{17}\hat\sigma^z_{35}-8\hat\sigma^z_{18}\hat\sigma^z_{34}-4\hat\sigma^z_{19}\hat\sigma^z_{2}4-51664\hat\sigma^z_{1}\hat\sigma^z_{52}-51664\hat\sigma^z_{9}\hat\sigma^z_{44}-4\hat\sigma^z_{14}\hat\sigma^z_{39}-8\hat\sigma^z_{15}\hat\sigma^z_{38}-$
$8\hat\sigma^z_{18}\hat\sigma^z_{35}-4\hat\sigma^z_{19}\hat\sigma^z_{34}+4\hat\sigma^z_{20}\hat\sigma^z_{2}4-51664\hat\sigma^z_{1}\hat\sigma^z_{53}-8\hat\sigma^z_{10}\hat\sigma^z_{44}-4\hat\sigma^z_{15}\hat\sigma^z_{39}-8\hat\sigma^z_{16}\hat\sigma^z_{38}-51664\hat\sigma^z_{18}\hat\sigma^z_{36}-8\hat\sigma^z_{19}\hat\sigma^z_{35}-$
$4\hat\sigma^z_{20}\hat\sigma^z_{34}-51664\hat\sigma^z_{10}\hat\sigma^z_{45}-8\hat\sigma^z_{11}\hat\sigma^z_{44}-8\hat\sigma^z_{16}\hat\sigma^z_{39}-8\hat\sigma^z_{17}\hat\sigma^z_{38}-8\hat\sigma^z_{19}\hat\sigma^z_{36}-4\hat\sigma^z_{20}\hat\sigma^z_{35}-51664\hat\sigma^z_{2}\hat\sigma^z_{54}-8\hat\sigma^z_{11}\hat\sigma^z_{45}-$
$8\hat\sigma^z_{12}\hat\sigma^z_{44}-8\hat\sigma^z_{17}\hat\sigma^z_{39}-8\hat\sigma^z_{18}\hat\sigma^z_{38}-51664\hat\sigma^z_{19}\hat\sigma^z_{37}-8\hat\sigma^z_{20}\hat\sigma^z_{36}-51664\hat\sigma^z_{2}\hat\sigma^z_{55}-51664\hat\sigma^z_{3}\hat\sigma^z_{54}-51664\hat\sigma^z_{11}\hat\sigma^z_{46}-8\hat\sigma^z_{12}\hat\sigma^z_{45}+$
$4\hat\sigma^z_{13}\hat\sigma^z_{44}-8\hat\sigma^z_{18}\hat\sigma^z_{39}-8\hat\sigma^z_{19}\hat\sigma^z_{38}-8\hat\sigma^z_{20}\hat\sigma^z_{37}-51664\hat\sigma^z_{2}\hat\sigma^z_{56}-8\hat\sigma^z_{4}\hat\sigma^z_{54}-8\hat\sigma^z_{12}\hat\sigma^z_{46}-8\hat\sigma^z_{13}\hat\sigma^z_{45}-4\hat\sigma^z_{19}\hat\sigma^z_{39}-4\hat\sigma^z_{20}\hat\sigma^z_{38}-$
$51664\hat\sigma^z_{2}\hat\sigma^z_{57}-51664\hat\sigma^z_{4}\hat\sigma^z_{55}-8\hat\sigma^z_{5}\hat\sigma^z_{54}-51664\hat\sigma^z_{12}\hat\sigma^z_{47}-8\hat\sigma^z_{13}\hat\sigma^z_{46}+4\hat\sigma^z_{14}\hat\sigma^z_{45}-4\hat\sigma^z_{20}\hat\sigma^z_{39}-51664\hat\sigma^z_{2}\hat\sigma^z_{58}-4\hat\sigma^z_{5}\hat\sigma^z_{55}-$
$8\hat\sigma^z_{6}\hat\sigma^z_{54}-8\hat\sigma^z_{13}\hat\sigma^z_{47}-8\hat\sigma^z_{14}\hat\sigma^z_{46}-51664\hat\sigma^z_{2}\hat\sigma^z_{59}-51664\hat\sigma^z_{5}\hat\sigma^z_{56}-8\hat\sigma^z_{6}\hat\sigma^z_{55}-8\hat\sigma^z_{7}\hat\sigma^z_{54}-51664\hat\sigma^z_{13}\hat\sigma^z_{48}-8\hat\sigma^z_{14}\hat\sigma^z_{47}-$
$51664\hat\sigma^z_{2}\hat\sigma^z_{60}-8\hat\sigma^z_{6}\hat\sigma^z_{56}+8\hat\sigma^z_{7}\hat\sigma^z_{55}-8\hat\sigma^z_{14}\hat\sigma^z_{48}-51664\hat\sigma^z_{2}\hat\sigma^z_{61}-51664\hat\sigma^z_{6}\hat\sigma^z_{57}-8\hat\sigma^z_{7}\hat\sigma^z_{56}+4\hat\sigma^z_{8}\hat\sigma^z_{55}-8\hat\sigma^z_{9}\hat\sigma^z_{54}-$
$51664\hat\sigma^z_{2}\hat\sigma^z_{62}-8\hat\sigma^z_{7}\hat\sigma^z_{57}-8\hat\sigma^z_{10}\hat\sigma^z_{54}-51664\hat\sigma^z_{15}\hat\sigma^z_{49}-51664\hat\sigma^z_{2}\hat\sigma^z_{63}-51664\hat\sigma^z_{7}\hat\sigma^z_{58}-8\hat\sigma^z_{8}\hat\sigma^z_{57}-8\hat\sigma^z_{11}\hat\sigma^z_{54}-8\hat\sigma^z_{16}\hat\sigma^z_{49}-$
$51664\hat\sigma^z_{2}\hat\sigma^z_{64}-8\hat\sigma^z_{8}\hat\sigma^z_{58}-8\hat\sigma^z_{12}\hat\sigma^z_{54}-51664\hat\sigma^z_{16}\hat\sigma^z_{50}-8\hat\sigma^z_{17}\hat\sigma^z_{49}-51664\hat\sigma^z_{2}\hat\sigma^z_{65}-8\hat\sigma^z_{13}\hat\sigma^z_{54}-8\hat\sigma^z_{17}\hat\sigma^z_{50}-8\hat\sigma^z_{18}\hat\sigma^z_{49}-$
$51664\hat\sigma^z_{2}\hat\sigma^z_{66}-51664\hat\sigma^z_{9}\hat\sigma^z_{59}-8\hat\sigma^z_{14}\hat\sigma^z_{54}-51664\hat\sigma^z_{17}\hat\sigma^z_{51}-8\hat\sigma^z_{18}\hat\sigma^z_{50}+4\hat\sigma^z_{19}\hat\sigma^z_{49}-51664\hat\sigma^z_{2}\hat\sigma^z_{67}-8\hat\sigma^z_{10}\hat\sigma^z_{59}-8\hat\sigma^z_{15}\hat\sigma^z_{54}-$
$8\hat\sigma^z_{18}\hat\sigma^z_{51}-8\hat\sigma^z_{19}\hat\sigma^z_{50}-51664\hat\sigma^z_{2}\hat\sigma^z_{68}-51664\hat\sigma^z_{10}\hat\sigma^z_{60}-8\hat\sigma^z_{11}\hat\sigma^z_{59}-8\hat\sigma^z_{16}\hat\sigma^z_{54}-51664\hat\sigma^z_{18}\hat\sigma^z_{52}-8\hat\sigma^z_{19}\hat\sigma^z_{51}+4\hat\sigma^z_{20}\hat\sigma^z_{50}-$
$8\hat\sigma^z_{11}\hat\sigma^z_{60}-8\hat\sigma^z_{17}\hat\sigma^z_{54}-8\hat\sigma^z_{19}\hat\sigma^z_{52}-8\hat\sigma^z_{20}\hat\sigma^z_{51}-51664\hat\sigma^z_{3}\hat\sigma^z_{69}-51664\hat\sigma^z_{11}\hat\sigma^z_{61}-8\hat\sigma^z_{12}\hat\sigma^z_{60}-4\hat\sigma^z_{13}\hat\sigma^z_{59}-8\hat\sigma^z_{18}\hat\sigma^z_{54}-$
$51664\hat\sigma^z_{19}\hat\sigma^z_{53}-8\hat\sigma^z_{20}\hat\sigma^z_{52}-51664\hat\sigma^z_{3}\hat\sigma^z_{70}-51664\hat\sigma^z_{4}\hat\sigma^z_{69}-8\hat\sigma^z_{12}\hat\sigma^z_{61}-8\hat\sigma^z_{19}\hat\sigma^z_{54}-8\hat\sigma^z_{20}\hat\sigma^z_{53}-51664\hat\sigma^z_{3}\hat\sigma^z_{71}-4\hat\sigma^z_{5}\hat\sigma^z_{69}-$
$51664\hat\sigma^z_{12}\hat\sigma^z_{62}-8\hat\sigma^z_{13}\hat\sigma^z_{61}-4\hat\sigma^z_{14}\hat\sigma^z_{60}-4\hat\sigma^z_{20}\hat\sigma^z_{54}-51664\hat\sigma^z_{3}\hat\sigma^z_{72}-51664\hat\sigma^z_{5}\hat\sigma^z_{70}+4\hat\sigma^z_{6}\hat\sigma^z_{69}-8\hat\sigma^z_{13}\hat\sigma^z_{62}+16\hat\sigma^z_{21}\hat\sigma^z_{54}-$
$51664\hat\sigma^z_{3}\hat\sigma^z_{73}-8\hat\sigma^z_{6}\hat\sigma^z_{70}-4\hat\sigma^z_{7}\hat\sigma^z_{69}-51664\hat\sigma^z_{13}\hat\sigma^z_{63}-8\hat\sigma^z_{14}\hat\sigma^z_{62}-51664\hat\sigma^z_{3}\hat\sigma^z_{74}-51664\hat\sigma^z_{6}\hat\sigma^z_{71}+4\hat\sigma^z_{8}\hat\sigma^z_{69}-8\hat\sigma^z_{14}\hat\sigma^z_{63}-$
$51664\hat\sigma^z_{3}\hat\sigma^z_{75}-8\hat\sigma^z_{7}\hat\sigma^z_{71}-4\hat\sigma^z_{8}\hat\sigma^z_{70}-51664\hat\sigma^z_{3}\hat\sigma^z_{76}-51664\hat\sigma^z_{7}\hat\sigma^z_{72}-51664\hat\sigma^z_{15}\hat\sigma^z_{64}-51664\hat\sigma^z_{3}\hat\sigma^z_{77}-8\hat\sigma^z_{8}\hat\sigma^z_{72}-$
$8\hat\sigma^z_{16}\hat\sigma^z_{64}-51664\hat\sigma^z_{3}\hat\sigma^z_{78}-51664\hat\sigma^z_{16}\hat\sigma^z_{65}-8\hat\sigma^z_{17}\hat\sigma^z_{64}-51664\hat\sigma^z_{3}\hat\sigma^z_{79}-51664\hat\sigma^z_{9}\hat\sigma^z_{73}-8\hat\sigma^z_{17}\hat\sigma^z_{65}-51664\hat\sigma^z_{3}\hat\sigma^z_{80}-$
$8\hat\sigma^z_{10}\hat\sigma^z_{73}-51664\hat\sigma^z_{17}\hat\sigma^z_{66}-8\hat\sigma^z_{18}\hat\sigma^z_{65}-4\hat\sigma^z_{19}\hat\sigma^z_{64}-51664\hat\sigma^z_{3}\hat\sigma^z_{81}-51664\hat\sigma^z_{10}\hat\sigma^z_{74}-8\hat\sigma^z_{18}\hat\sigma^z_{66}-51664\hat\sigma^z_{3}\hat\sigma^z_{82}-$
$8\hat\sigma^z_{11}\hat\sigma^z_{74}-4\hat\sigma^z_{12}\hat\sigma^z_{73}-51664\hat\sigma^z_{18}\hat\sigma^z_{67}-8\hat\sigma^z_{19}\hat\sigma^z_{66}-4\hat\sigma^z_{20}\hat\sigma^z_{65}-51664\hat\sigma^z_{11}\hat\sigma^z_{75}-4\hat\sigma^z_{13}\hat\sigma^z_{73}-8\hat\sigma^z_{19}\hat\sigma^z_{67}-51664\hat\sigma^z_{4}\hat\sigma^z_{83}-$
$8\hat\sigma^z_{12}\hat\sigma^z_{75}-4\hat\sigma^z_{13}\hat\sigma^z_{74}-4\hat\sigma^z_{14}\hat\sigma^z_{73}-51664\hat\sigma^z_{19}\hat\sigma^z_{68}-8\hat\sigma^z_{20}\hat\sigma^z_{67}-51664\hat\sigma^z_{4}\hat\sigma^z_{84}-51664\hat\sigma^z_{5}\hat\sigma^z_{83}-51664\hat\sigma^z_{12}\hat\sigma^z_{76}-4\hat\sigma^z_{14}\hat\sigma^z_{74}-$
$8\hat\sigma^z_{20}\hat\sigma^z_{68}-51664\hat\sigma^z_{4}\hat\sigma^z_{85}-16\hat\sigma^z_{6}\hat\sigma^z_{83}-8\hat\sigma^z_{13}\hat\sigma^z_{76}-4\hat\sigma^z_{14}\hat\sigma^z_{75}-51664\hat\sigma^z_{4}\hat\sigma^z_{86}-51664\hat\sigma^z_{6}\hat\sigma^z_{84}-8\hat\sigma^z_{7}\hat\sigma^z_{83}-51664\hat\sigma^z_{13}\hat\sigma^z_{77}-$
$51664\hat\sigma^z_{4}\hat\sigma^z_{87}-16\hat\sigma^z_{7}\hat\sigma^z_{84}-8\hat\sigma^z_{14}\hat\sigma^z_{77}-51664\hat\sigma^z_{4}\hat\sigma^z_{88}-51664\hat\sigma^z_{7}\hat\sigma^z_{85}+24\hat\sigma^z_{8}\hat\sigma^z_{84}-4\hat\sigma^z_{9}\hat\sigma^z_{83}-51664\hat\sigma^z_{4}\hat\sigma^z_{89}-16\hat\sigma^z_{8}\hat\sigma^z_{85}-$
$4\hat\sigma^z_{10}\hat\sigma^z_{83}-51664\hat\sigma^z_{15}\hat\sigma^z_{78}-51664\hat\sigma^z_{4}\hat\sigma^z_{90}-51664\hat\sigma^z_{8}\hat\sigma^z_{86}-4\hat\sigma^z_{10}\hat\sigma^z_{84}-4\hat\sigma^z_{11}\hat\sigma^z_{83}-8\hat\sigma^z_{16}\hat\sigma^z_{78}-51664\hat\sigma^z_{4}\hat\sigma^z_{91}+4\hat\sigma^z_{9}\hat\sigma^z_{86}+$
$4\hat\sigma^z_{10}\hat\sigma^z_{85}-4\hat\sigma^z_{12}\hat\sigma^z_{83}-51664\hat\sigma^z_{16}\hat\sigma^z_{79}-51664\hat\sigma^z_{4}\hat\sigma^z_{92}-51664\hat\sigma^z_{9}\hat\sigma^z_{87}+4\hat\sigma^z_{10}\hat\sigma^z_{86}-4\hat\sigma^z_{13}\hat\sigma^z_{83}-8\hat\sigma^z_{17}\hat\sigma^z_{79}-4\hat\sigma^z_{18}\hat\sigma^z_{78}-$
$51664\hat\sigma^z_{4}\hat\sigma^z_{93}-8\hat\sigma^z_{10}\hat\sigma^z_{87}+4\hat\sigma^z_{11}\hat\sigma^z_{86}-4\hat\sigma^z_{13}\hat\sigma^z_{84}-4\hat\sigma^z_{14}\hat\sigma^z_{83}-51664\hat\sigma^z_{17}\hat\sigma^z_{80}-4\hat\sigma^z_{19}\hat\sigma^z_{78}-51664\hat\sigma^z_{4}\hat\sigma^z_{94}-51664\hat\sigma^z_{10}\hat\sigma^z_{88}+$
$4\hat\sigma^z_{12}\hat\sigma^z_{86}+4\hat\sigma^z_{13}\hat\sigma^z_{85}-4\hat\sigma^z_{15}\hat\sigma^z_{83}-8\hat\sigma^z_{18}\hat\sigma^z_{80}-4\hat\sigma^z_{19}\hat\sigma^z_{79}-4\hat\sigma^z_{20}\hat\sigma^z_{78}-51664\hat\sigma^z_{4}\hat\sigma^z_{95}-8\hat\sigma^z_{11}\hat\sigma^z_{88}+4\hat\sigma^z_{13}\hat\sigma^z_{86}-4\hat\sigma^z_{16}\hat\sigma^z_{83}-$
$51664\hat\sigma^z_{18}\hat\sigma^z_{81}-4\hat\sigma^z_{20}\hat\sigma^z_{79}-51664\hat\sigma^z_{4}\hat\sigma^z_{96}-51664\hat\sigma^z_{11}\hat\sigma^z_{89}+4\hat\sigma^z_{14}\hat\sigma^z_{86}-4\hat\sigma^z_{16}\hat\sigma^z_{84}-8\hat\sigma^z_{17}\hat\sigma^z_{83}-8\hat\sigma^z_{19}\hat\sigma^z_{81}-4\hat\sigma^z_{20}\hat\sigma^z_{80}-$
$8\hat\sigma^z_{12}\hat\sigma^z_{89}+4\hat\sigma^z_{15}\hat\sigma^z_{86}+4\hat\sigma^z_{16}\hat\sigma^z_{85}-4\hat\sigma^z_{18}\hat\sigma^z_{83}-51664\hat\sigma^z_{19}\hat\sigma^z_{82}-51664\hat\sigma^z_{5}\hat\sigma^z_{97}-51664\hat\sigma^z_{12}\hat\sigma^z_{90}+4\hat\sigma^z_{16}\hat\sigma^z_{86}-4\hat\sigma^z_{18}\hat\sigma^z_{84}-$
$4\hat\sigma^z_{19}\hat\sigma^z_{83}-8\hat\sigma^z_{20}\hat\sigma^z_{82}-51664\hat\sigma^z_{5}\hat\sigma^z_{98}-51664\hat\sigma^z_{6}\hat\sigma^z_{97}-8\hat\sigma^z_{13}\hat\sigma^z_{90}+8\hat\sigma^z_{17}\hat\sigma^z_{86}+4\hat\sigma^z_{18}\hat\sigma^z_{85}-51664\hat\sigma^z_{5}\hat\sigma^z_{99}-16\hat\sigma^z_{7}\hat\sigma^z_{97}-$
$51664\hat\sigma^z_{13}\hat\sigma^z_{91}+4\hat\sigma^z_{18}\hat\sigma^z_{86}+8\hat\sigma^z_{21}\hat\sigma^z_{83}-51664\hat\sigma^z_{5}\hat\sigma^z_{100}-51664\hat\sigma^z_{7}\hat\sigma^z_{98}-8\hat\sigma^z_{8}\hat\sigma^z_{97}-8\hat\sigma^z_{14}\hat\sigma^z_{91}+4\hat\sigma^z_{19}\hat\sigma^z_{86}-51664\hat\sigma^z_{5}\hat\sigma^z_{101}-$
$8\hat\sigma^z_{8}\hat\sigma^z_{98}-8\hat\sigma^z_{9}\hat\sigma^z_{97}+8\hat\sigma^z_{22}\hat\sigma^z_{84}-51664\hat\sigma^z_{5}\hat\sigma^z_{102}-51664\hat\sigma^z_{8}\hat\sigma^z_{99}-4\hat\sigma^z_{9}\hat\sigma^z_{98}-8\hat\sigma^z_{10}\hat\sigma^z_{97}-51664\hat\sigma^z_{15}\hat\sigma^z_{92}-8\hat\sigma^z_{21}\hat\sigma^z_{86}-$
$8\hat\sigma^z_{22}\hat\sigma^z_{85}-8\hat\sigma^z_{23}\hat\sigma^z_{84}-51664\hat\sigma^z_{5}\hat\sigma^z_{9}4-4\hat\sigma^z_{9}\hat\sigma^z_{99}-8\hat\sigma^z_{10}\hat\sigma^z_{98}-8\hat\sigma^z_{11}\hat\sigma^z_{97}-8\hat\sigma^z_{16}\hat\sigma^z_{92}+8\hat\sigma^z_{23}\hat\sigma^z_{85}-51664\hat\sigma^z_{5}\hat\sigma^z_{104}-$

$$51664\hat{\sigma}^z_{9}\hat{\sigma}^z_{100} - 4\hat{\sigma}^z_{10}\hat{\sigma}^z_{99} - 8\hat{\sigma}^z_{11}\hat{\sigma}^z_{98} - 8\hat{\sigma}^z_{12}\hat{\sigma}^z_{97} - 51664\hat{\sigma}^z_{16}\hat{\sigma}^z_{93} - 51664\hat{\sigma}^z_{5}\hat{\sigma}^z_{105} - 8\hat{\sigma}^z_{10}\hat{\sigma}^z_{100} - 8\hat{\sigma}^z_{11}\hat{\sigma}^z_{99} -$$
$$8\hat{\sigma}^z_{12}\hat{\sigma}^z_{98} - 8\hat{\sigma}^z_{13}\hat{\sigma}^z_{97} - 8\hat{\sigma}^z_{17}\hat{\sigma}^z_{93} - 51664\hat{\sigma}^z_{5}\hat{\sigma}^z_{106} - 51664\hat{\sigma}^z_{10}\hat{\sigma}^z_{101} - 8\hat{\sigma}^z_{11}\hat{\sigma}^z_{100} - 8\hat{\sigma}^z_{12}\hat{\sigma}^z_{99} - 8\hat{\sigma}^z_{13}\hat{\sigma}^z_{98} - 8\hat{\sigma}^z_{14}\hat{\sigma}^z_{97} -$$
$$51664\hat{\sigma}^z_{17}\hat{\sigma}^z_{94} - 51664\hat{\sigma}^z_{5}\hat{\sigma}^z_{107} - 8\hat{\sigma}^z_{11}\hat{\sigma}^z_{101} - 4\hat{\sigma}^z_{12}\hat{\sigma}^z_{100} - 4\hat{\sigma}^z_{13}\hat{\sigma}^z_{99} - 4\hat{\sigma}^z_{14}\hat{\sigma}^z_{98} - 8\hat{\sigma}^z_{15}\hat{\sigma}^z_{97} - 8\hat{\sigma}^z_{18}\hat{\sigma}^z_{94} - 51664\hat{\sigma}^z_{5}\hat{\sigma}^z_{108} -$$
$$51664\hat{\sigma}^z_{11}\hat{\sigma}^z_{102} - 8\hat{\sigma}^z_{12}\hat{\sigma}^z_{101} + 4\hat{\sigma}^z_{13}\hat{\sigma}^z_{100} - 4\hat{\sigma}^z_{14}\hat{\sigma}^z_{99} - 4\hat{\sigma}^z_{15}\hat{\sigma}^z_{98} - 8\hat{\sigma}^z_{16}\hat{\sigma}^z_{97} - 51664\hat{\sigma}^z_{18}\hat{\sigma}^z_{95} - 51664\hat{\sigma}^z_{5}\hat{\sigma}^z_{109} - 8\hat{\sigma}^z_{12}\hat{\sigma}^z_{102} -$$
$$4\hat{\sigma}^z_{13}\hat{\sigma}^z_{101} + 4\hat{\sigma}^z_{14}\hat{\sigma}^z_{100} - 4\hat{\sigma}^z_{15}\hat{\sigma}^z_{99} - 8\hat{\sigma}^z_{16}\hat{\sigma}^z_{98} - 8\hat{\sigma}^z_{17}\hat{\sigma}^z_{97} - 8\hat{\sigma}^z_{19}\hat{\sigma}^z_{95} - 51664\hat{\sigma}^z_{12}\hat{\sigma}^z_{9}4 - 8\hat{\sigma}^z_{13}\hat{\sigma}^z_{102} + 4\hat{\sigma}^z_{14}\hat{\sigma}^z_{101} -$$
$$4\hat{\sigma}^z_{16}\hat{\sigma}^z_{99} - 8\hat{\sigma}^z_{17}\hat{\sigma}^z_{98} - 8\hat{\sigma}^z_{18}\hat{\sigma}^z_{97} - 51664\hat{\sigma}^z_{19}\hat{\sigma}^z_{96} - 51664\hat{\sigma}^z_{6}\hat{\sigma}^z_{110} - 8\hat{\sigma}^z_{13}\hat{\sigma}^z_{9}4 - 4\hat{\sigma}^z_{14}\hat{\sigma}^z_{102} - 8\hat{\sigma}^z_{17}\hat{\sigma}^z_{99} - 8\hat{\sigma}^z_{18}\hat{\sigma}^z_{98} -$$
$$8\hat{\sigma}^z_{19}\hat{\sigma}^z_{97} - 8\hat{\sigma}^z_{20}\hat{\sigma}^z_{96} - 51664\hat{\sigma}^z_{6}\hat{\sigma}^z_{111} - 51664\hat{\sigma}^z_{7}\hat{\sigma}^z_{110} - 51664\hat{\sigma}^z_{13}\hat{\sigma}^z_{104} - 8\hat{\sigma}^z_{14}\hat{\sigma}^z_{9}4 - 4\hat{\sigma}^z_{18}\hat{\sigma}^z_{99} - 4\hat{\sigma}^z_{19}\hat{\sigma}^z_{98} - 4\hat{\sigma}^z_{20}\hat{\sigma}^z_{97} -$$
$$51664\hat{\sigma}^z_{6}\hat{\sigma}^z_{112} - 8\hat{\sigma}^z_{14}\hat{\sigma}^z_{104} - 4\hat{\sigma}^z_{19}\hat{\sigma}^z_{99} - 4\hat{\sigma}^z_{20}\hat{\sigma}^z_{98} + 8\hat{\sigma}^z_{21}\hat{\sigma}^z_{97} - 51664\hat{\sigma}^z_{6}\hat{\sigma}^z_{113} - 51664\hat{\sigma}^z_{8}\hat{\sigma}^z_{111} - 8\hat{\sigma}^z_{9}\hat{\sigma}^z_{110} - 4\hat{\sigma}^z_{20}\hat{\sigma}^z_{99} -$$
$$51664\hat{\sigma}^z_{6}\hat{\sigma}^z_{114} - 4\hat{\sigma}^z_{9}\hat{\sigma}^z_{111} - 8\hat{\sigma}^z_{10}\hat{\sigma}^z_{110} - 51664\hat{\sigma}^z_{15}\hat{\sigma}^z_{105} + 8\hat{\sigma}^z_{22}\hat{\sigma}^z_{98} - 51664\hat{\sigma}^z_{6}\hat{\sigma}^z_{115} - 51664\hat{\sigma}^z_{9}\hat{\sigma}^z_{112} - 8\hat{\sigma}^z_{10}\hat{\sigma}^z_{111} -$$
$$8\hat{\sigma}^z_{11}\hat{\sigma}^z_{110} - 8\hat{\sigma}^z_{16}\hat{\sigma}^z_{105} + 8\hat{\sigma}^z_{38}\hat{\sigma}^z_{83} - 51664\hat{\sigma}^z_{6}\hat{\sigma}^z_{116} - 8\hat{\sigma}^z_{10}\hat{\sigma}^z_{112} - 8\hat{\sigma}^z_{11}\hat{\sigma}^z_{111} - 8\hat{\sigma}^z_{12}\hat{\sigma}^z_{110} - 51664\hat{\sigma}^z_{16}\hat{\sigma}^z_{106} - 8\hat{\sigma}^z_{17}\hat{\sigma}^z_{105} +$$
$$8\hat{\sigma}^z_{23}\hat{\sigma}^z_{99} - 51664\hat{\sigma}^z_{6}\hat{\sigma}^z_{117} - 51664\hat{\sigma}^z_{10}\hat{\sigma}^z_{113} - 8\hat{\sigma}^z_{11}\hat{\sigma}^z_{112} - 8\hat{\sigma}^z_{12}\hat{\sigma}^z_{111} - 8\hat{\sigma}^z_{13}\hat{\sigma}^z_{110} - 8\hat{\sigma}^z_{17}\hat{\sigma}^z_{106} - 4\hat{\sigma}^z_{18}\hat{\sigma}^z_{105} + 8\hat{\sigma}^z_{39}\hat{\sigma}^z_{84} -$$
$$51664\hat{\sigma}^z_{6}\hat{\sigma}^z_{118} - 8\hat{\sigma}^z_{11}\hat{\sigma}^z_{113} - 8\hat{\sigma}^z_{13}\hat{\sigma}^z_{111} - 8\hat{\sigma}^z_{14}\hat{\sigma}^z_{110} - 51664\hat{\sigma}^z_{17}\hat{\sigma}^z_{107} - 8\hat{\sigma}^z_{18}\hat{\sigma}^z_{106} + 4\hat{\sigma}^z_{19}\hat{\sigma}^z_{105} - 8\hat{\sigma}^z_{38}\hat{\sigma}^z_{86} - 8\hat{\sigma}^z_{39}\hat{\sigma}^z_{85} -$$
$$51664\hat{\sigma}^z_{6}\hat{\sigma}^z_{119} - 51664\hat{\sigma}^z_{11}\hat{\sigma}^z_{114} - 8\hat{\sigma}^z_{12}\hat{\sigma}^z_{113} + 4\hat{\sigma}^z_{13}\hat{\sigma}^z_{112} - 4\hat{\sigma}^z_{14}\hat{\sigma}^z_{111} - 8\hat{\sigma}^z_{15}\hat{\sigma}^z_{110} - 8\hat{\sigma}^z_{18}\hat{\sigma}^z_{107} - 4\hat{\sigma}^z_{19}\hat{\sigma}^z_{106} + 4\hat{\sigma}^z_{20}\hat{\sigma}^z_{105} -$$
$$51664\hat{\sigma}^z_{6}\hat{\sigma}^z_{120} - 8\hat{\sigma}^z_{12}\hat{\sigma}^z_{114} - 4\hat{\sigma}^z_{15}\hat{\sigma}^z_{111} - 8\hat{\sigma}^z_{16}\hat{\sigma}^z_{110} - 51664\hat{\sigma}^z_{18}\hat{\sigma}^z_{108} - 8\hat{\sigma}^z_{19}\hat{\sigma}^z_{107} + 4\hat{\sigma}^z_{20}\hat{\sigma}^z_{106} - 51664\hat{\sigma}^z_{6}\hat{\sigma}^z_{121} -$$
$$51664\hat{\sigma}^z_{12}\hat{\sigma}^z_{115} - 8\hat{\sigma}^z_{13}\hat{\sigma}^z_{114} + 4\hat{\sigma}^z_{14}\hat{\sigma}^z_{113} - 8\hat{\sigma}^z_{16}\hat{\sigma}^z_{111} - 8\hat{\sigma}^z_{17}\hat{\sigma}^z_{110} - 8\hat{\sigma}^z_{19}\hat{\sigma}^z_{108} - 4\hat{\sigma}^z_{20}\hat{\sigma}^z_{107} - 8\hat{\sigma}^z_{13}\hat{\sigma}^z_{115} - 8\hat{\sigma}^z_{17}\hat{\sigma}^z_{111} -$$
$$8\hat{\sigma}^z_{18}\hat{\sigma}^z_{110} - 51664\hat{\sigma}^z_{19}\hat{\sigma}^z_{109} - 8\hat{\sigma}^z_{20}\hat{\sigma}^z_{108} - 51664\hat{\sigma}^z_{7}\hat{\sigma}^z_{122} - 51664\hat{\sigma}^z_{13}\hat{\sigma}^z_{116} - 8\hat{\sigma}^z_{14}\hat{\sigma}^z_{115} - 8\hat{\sigma}^z_{18}\hat{\sigma}^z_{111} - 8\hat{\sigma}^z_{19}\hat{\sigma}^z_{110} -$$
$$8\hat{\sigma}^z_{20}\hat{\sigma}^z_{109} - 51664\hat{\sigma}^z_{7}\hat{\sigma}^z_{123} - 51664\hat{\sigma}^z_{8}\hat{\sigma}^z_{122} - 8\hat{\sigma}^z_{14}\hat{\sigma}^z_{116} - 4\hat{\sigma}^z_{19}\hat{\sigma}^z_{111} - 4\hat{\sigma}^z_{20}\hat{\sigma}^z_{110} - 51664\hat{\sigma}^z_{7}\hat{\sigma}^z_{124} - 8\hat{\sigma}^z_{9}\hat{\sigma}^z_{122} -$$
$$4\hat{\sigma}^z_{20}\hat{\sigma}^z_{111} + 8\hat{\sigma}^z_{21}\hat{\sigma}^z_{110} - 51664\hat{\sigma}^z_{7}\hat{\sigma}^z_{125} - 51664\hat{\sigma}^z_{9}\hat{\sigma}^z_{123} - 8\hat{\sigma}^z_{10}\hat{\sigma}^z_{122} - 51664\hat{\sigma}^z_{15}\hat{\sigma}^z_{117} - 51664\hat{\sigma}^z_{7}\hat{\sigma}^z_{11}7 - 8\hat{\sigma}^z_{10}\hat{\sigma}^z_{123} -$$
$$8\hat{\sigma}^z_{11}\hat{\sigma}^z_{122} - 8\hat{\sigma}^z_{16}\hat{\sigma}^z_{117} + 8\hat{\sigma}^z_{22}\hat{\sigma}^z_{111} - 51664\hat{\sigma}^z_{7}\hat{\sigma}^z_{127} - 51664\hat{\sigma}^z_{10}\hat{\sigma}^z_{124} - 8\hat{\sigma}^z_{12}\hat{\sigma}^z_{122} - 51664\hat{\sigma}^z_{16}\hat{\sigma}^z_{118} - 8\hat{\sigma}^z_{17}\hat{\sigma}^z_{117} -$$
$$51664\hat{\sigma}^z_{7}\hat{\sigma}^z_{128} - 8\hat{\sigma}^z_{11}\hat{\sigma}^z_{124} - 8\hat{\sigma}^z_{13}\hat{\sigma}^z_{122} - 8\hat{\sigma}^z_{17}\hat{\sigma}^z_{118} + 8\hat{\sigma}^z_{38}\hat{\sigma}^z_{97} - 51664\hat{\sigma}^z_{7}\hat{\sigma}^z_{129} - 51664\hat{\sigma}^z_{11}\hat{\sigma}^z_{125} - 4\hat{\sigma}^z_{13}\hat{\sigma}^z_{123} -$$
$$8\hat{\sigma}^z_{14}\hat{\sigma}^z_{122} - 51664\hat{\sigma}^z_{17}\hat{\sigma}^z_{119} - 8\hat{\sigma}^z_{18}\hat{\sigma}^z_{118} + 4\hat{\sigma}^z_{19}\hat{\sigma}^z_{117} - 51664\hat{\sigma}^z_{7}\hat{\sigma}^z_{130} - 8\hat{\sigma}^z_{12}\hat{\sigma}^z_{125} - 8\hat{\sigma}^z_{15}\hat{\sigma}^z_{122} - 8\hat{\sigma}^z_{18}\hat{\sigma}^z_{119} + 8\hat{\sigma}^z_{39}\hat{\sigma}^z_{98} +$$
$$8\hat{\sigma}^z_{54}\hat{\sigma}^z_{83} - 51664\hat{\sigma}^z_{7}\hat{\sigma}^z_{131} - 51664\hat{\sigma}^z_{12}\hat{\sigma}^z_{11}7 - 4\hat{\sigma}^z_{14}\hat{\sigma}^z_{124} - 8\hat{\sigma}^z_{16}\hat{\sigma}^z_{122} - 51664\hat{\sigma}^z_{18}\hat{\sigma}^z_{120} - 8\hat{\sigma}^z_{19}\hat{\sigma}^z_{119} + 4\hat{\sigma}^z_{20}\hat{\sigma}^z_{118} -$$
$$51664\hat{\sigma}^z_{7}\hat{\sigma}^z_{132} - 8\hat{\sigma}^z_{13}\hat{\sigma}^z_{11}7 - 8\hat{\sigma}^z_{17}\hat{\sigma}^z_{122} - 8\hat{\sigma}^z_{19}\hat{\sigma}^z_{120} - 51664\hat{\sigma}^z_{13}\hat{\sigma}^z_{127} - 8\hat{\sigma}^z_{18}\hat{\sigma}^z_{122} - 51664\hat{\sigma}^z_{19}\hat{\sigma}^z_{121} - 8\hat{\sigma}^z_{20}\hat{\sigma}^z_{120} -$$
$$8\hat{\sigma}^z_{54}\hat{\sigma}^z_{86} - 51664\hat{\sigma}^z_{8}\hat{\sigma}^z_{133} - 8\hat{\sigma}^z_{14}\hat{\sigma}^z_{127} - 8\hat{\sigma}^z_{19}\hat{\sigma}^z_{122} - 8\hat{\sigma}^z_{20}\hat{\sigma}^z_{121} - 51664\hat{\sigma}^z_{8}\hat{\sigma}^z_{134} - 51664\hat{\sigma}^z_{9}\hat{\sigma}^z_{133} - 4\hat{\sigma}^z_{20}\hat{\sigma}^z_{122} -$$
$$51664\hat{\sigma}^z_{8}\hat{\sigma}^z_{135} - 51664\hat{\sigma}^z_{15}\hat{\sigma}^z_{128} + 8\hat{\sigma}^z_{21}\hat{\sigma}^z_{122} - 51664\hat{\sigma}^z_{8}\hat{\sigma}^z_{136} - 51664\hat{\sigma}^z_{10}\hat{\sigma}^z_{134} - 8\hat{\sigma}^z_{16}\hat{\sigma}^z_{128} - 51664\hat{\sigma}^z_{8}\hat{\sigma}^z_{137} -$$
$$4\hat{\sigma}^z_{12}\hat{\sigma}^z_{133} - 51664\hat{\sigma}^z_{16}\hat{\sigma}^z_{129} - 51664\hat{\sigma}^z_{8}\hat{\sigma}^z_{138} - 51664\hat{\sigma}^z_{11}\hat{\sigma}^z_{135} - 4\hat{\sigma}^z_{13}\hat{\sigma}^z_{133} - 8\hat{\sigma}^z_{17}\hat{\sigma}^z_{129} - 4\hat{\sigma}^z_{13}\hat{\sigma}^z_{134} - 4\hat{\sigma}^z_{14}\hat{\sigma}^z_{133} -$$
$$51664\hat{\sigma}^z_{17}\hat{\sigma}^z_{130} - 4\hat{\sigma}^z_{19}\hat{\sigma}^z_{128} - 51664\hat{\sigma}^z_{9}\hat{\sigma}^z_{139} - 4\hat{\sigma}^z_{14}\hat{\sigma}^z_{134} - 8\hat{\sigma}^z_{18}\hat{\sigma}^z_{130} + 8\hat{\sigma}^z_{38}\hat{\sigma}^z_{110} - 51664\hat{\sigma}^z_{9}\hat{\sigma}^z_{140} - 51664\hat{\sigma}^z_{10}\hat{\sigma}^z_{139} -$$
$$4\hat{\sigma}^z_{14}\hat{\sigma}^z_{135} - 51664\hat{\sigma}^z_{18}\hat{\sigma}^z_{131} - 4\hat{\sigma}^z_{20}\hat{\sigma}^z_{129} - 51664\hat{\sigma}^z_{9}\hat{\sigma}^z_{141} - 16\hat{\sigma}^z_{11}\hat{\sigma}^z_{139} - 8\hat{\sigma}^z_{19}\hat{\sigma}^z_{131} + 8\hat{\sigma}^z_{39}\hat{\sigma}^z_{111} - 51664\hat{\sigma}^z_{9}\hat{\sigma}^z_{142} -$$
$$51664\hat{\sigma}^z_{11}\hat{\sigma}^z_{140} - 16\hat{\sigma}^z_{12}\hat{\sigma}^z_{139} - 51664\hat{\sigma}^z_{15}\hat{\sigma}^z_{136} - 51664\hat{\sigma}^z_{19}\hat{\sigma}^z_{132} + 8\hat{\sigma}^z_{54}\hat{\sigma}^z_{97} - 51664\hat{\sigma}^z_{9}\hat{\sigma}^z_{143} - 24\hat{\sigma}^z_{12}\hat{\sigma}^z_{140} - 16\hat{\sigma}^z_{13}\hat{\sigma}^z_{139} -$$
$$8\hat{\sigma}^z_{20}\hat{\sigma}^z_{132} - 51664\hat{\sigma}^z_{9}\hat{\sigma}^z_{144} - 51664\hat{\sigma}^z_{12}\hat{\sigma}^z_{141} - 8\hat{\sigma}^z_{13}\hat{\sigma}^z_{140} - 16\hat{\sigma}^z_{14}\hat{\sigma}^z_{139} - 51664\hat{\sigma}^z_{16}\hat{\sigma}^z_{137} - 51664\hat{\sigma}^z_{9}\hat{\sigma}^z_{145} - 16\hat{\sigma}^z_{13}\hat{\sigma}^z_{141} -$$
$$8\hat{\sigma}^z_{14}\hat{\sigma}^z_{140} - 8\hat{\sigma}^z_{15}\hat{\sigma}^z_{139} - 4\hat{\sigma}^z_{18}\hat{\sigma}^z_{136} - 51664\hat{\sigma}^z_{9}\hat{\sigma}^z_{146} - 51664\hat{\sigma}^z_{13}\hat{\sigma}^z_{142} - 8\hat{\sigma}^z_{14}\hat{\sigma}^z_{141} - 4\hat{\sigma}^z_{15}\hat{\sigma}^z_{140} - 8\hat{\sigma}^z_{16}\hat{\sigma}^z_{139} -$$
$$51664\hat{\sigma}^z_{17}\hat{\sigma}^z_{138} - 4\hat{\sigma}^z_{19}\hat{\sigma}^z_{136} - 51664\hat{\sigma}^z_{9}\hat{\sigma}^z_{147} - 16\hat{\sigma}^z_{14}\hat{\sigma}^z_{142} - 4\hat{\sigma}^z_{15}\hat{\sigma}^z_{141} - 8\hat{\sigma}^z_{16}\hat{\sigma}^z_{140} - 8\hat{\sigma}^z_{17}\hat{\sigma}^z_{139} - 4\hat{\sigma}^z_{19}\hat{\sigma}^z_{137} -$$
$$4\hat{\sigma}^z_{20}\hat{\sigma}^z_{136} - 51664\hat{\sigma}^z_{9}\hat{\sigma}^z_{148} - 51664\hat{\sigma}^z_{14}\hat{\sigma}^z_{143} - 4\hat{\sigma}^z_{15}\hat{\sigma}^z_{142} - 4\hat{\sigma}^z_{16}\hat{\sigma}^z_{141} - 8\hat{\sigma}^z_{17}\hat{\sigma}^z_{140} - 8\hat{\sigma}^z_{18}\hat{\sigma}^z_{139} - 4\hat{\sigma}^z_{20}\hat{\sigma}^z_{137} - 4\hat{\sigma}^z_{15}\hat{\sigma}^z_{143} -$$
$$4\hat{\sigma}^z_{16}\hat{\sigma}^z_{142} - 8\hat{\sigma}^z_{17}\hat{\sigma}^z_{141} - 8\hat{\sigma}^z_{18}\hat{\sigma}^z_{140} - 8\hat{\sigma}^z_{19}\hat{\sigma}^z_{139} - 4\hat{\sigma}^z_{20}\hat{\sigma}^z_{138} - 51664\hat{\sigma}^z_{10}\hat{\sigma}^z_{149} - 51664\hat{\sigma}^z_{15}\hat{\sigma}^z_{144} - 4\hat{\sigma}^z_{18}\hat{\sigma}^z_{141} - 4\hat{\sigma}^z_{19}\hat{\sigma}^z_{140} -$$
$$4\hat{\sigma}^z_{20}\hat{\sigma}^z_{139} - 51664\hat{\sigma}^z_{10}\hat{\sigma}^z_{150} - 51664\hat{\sigma}^z_{11}\hat{\sigma}^z_{149} - 8\hat{\sigma}^z_{16}\hat{\sigma}^z_{144} - 4\hat{\sigma}^z_{18}\hat{\sigma}^z_{142} - 4\hat{\sigma}^z_{19}\hat{\sigma}^z_{141} - 4\hat{\sigma}^z_{20}\hat{\sigma}^z_{140} + 8\hat{\sigma}^z_{21}\hat{\sigma}^z_{139} + 8\hat{\sigma}^z_{38}\hat{\sigma}^z_{122} -$$
$$51664\hat{\sigma}^z_{10}\hat{\sigma}^z_{151} - 16\hat{\sigma}^z_{12}\hat{\sigma}^z_{149} - 51664\hat{\sigma}^z_{16}\hat{\sigma}^z_{145} - 4\hat{\sigma}^z_{17}\hat{\sigma}^z_{144} - 4\hat{\sigma}^z_{19}\hat{\sigma}^z_{142} - 4\hat{\sigma}^z_{20}\hat{\sigma}^z_{141} - 51664\hat{\sigma}^z_{10}\hat{\sigma}^z_{152} - 51664\hat{\sigma}^z_{12}\hat{\sigma}^z_{150} -$$
$$24\hat{\sigma}^z_{13}\hat{\sigma}^z_{149} - 8\hat{\sigma}^z_{17}\hat{\sigma}^z_{145} - 4\hat{\sigma}^z_{18}\hat{\sigma}^z_{144} - 4\hat{\sigma}^z_{19}\hat{\sigma}^z_{143} - 4\hat{\sigma}^z_{20}\hat{\sigma}^z_{142} + 8\hat{\sigma}^z_{22}\hat{\sigma}^z_{140} - 51664\hat{\sigma}^z_{10}\hat{\sigma}^z_{153} - 24\hat{\sigma}^z_{13}\hat{\sigma}^z_{150} - 16\hat{\sigma}^z_{14}\hat{\sigma}^z_{149} -$$
$$51664\hat{\sigma}^z_{17}\hat{\sigma}^z_{146} - 4\hat{\sigma}^z_{18}\hat{\sigma}^z_{145} - 4\hat{\sigma}^z_{19}\hat{\sigma}^z_{144} - 4\hat{\sigma}^z_{20}\hat{\sigma}^z_{143} - 51664\hat{\sigma}^z_{10}\hat{\sigma}^z_{154} - 51664\hat{\sigma}^z_{13}\hat{\sigma}^z_{151} - 8\hat{\sigma}^z_{14}\hat{\sigma}^z_{150} - 8\hat{\sigma}^z_{15}\hat{\sigma}^z_{149} -$$
$$8\hat{\sigma}^z_{18}\hat{\sigma}^z_{146} - 4\hat{\sigma}^z_{19}\hat{\sigma}^z_{145} - 4\hat{\sigma}^z_{20}\hat{\sigma}^z_{144} + 8\hat{\sigma}^z_{23}\hat{\sigma}^z_{141} + 8\hat{\sigma}^z_{54}\hat{\sigma}^z_{110} - 51664\hat{\sigma}^z_{10}\hat{\sigma}^z_{155} - 16\hat{\sigma}^z_{14}\hat{\sigma}^z_{151} - 4\hat{\sigma}^z_{15}\hat{\sigma}^z_{150} - 8\hat{\sigma}^z_{16}\hat{\sigma}^z_{149} -$$
$$51664\hat{\sigma}^z_{18}\hat{\sigma}^z_{147} - 4\hat{\sigma}^z_{19}\hat{\sigma}^z_{146} - 4\hat{\sigma}^z_{20}\hat{\sigma}^z_{145} - 51664\hat{\sigma}^z_{10}\hat{\sigma}^z_{156} - 51664\hat{\sigma}^z_{14}\hat{\sigma}^z_{152} - 4\hat{\sigma}^z_{15}\hat{\sigma}^z_{151} - 8\hat{\sigma}^z_{16}\hat{\sigma}^z_{150} - 8\hat{\sigma}^z_{17}\hat{\sigma}^z_{149} -$$
$$8\hat{\sigma}^z_{19}\hat{\sigma}^z_{147} - 4\hat{\sigma}^z_{20}\hat{\sigma}^z_{146} - 51664\hat{\sigma}^z_{10}\hat{\sigma}^z_{157} - 4\hat{\sigma}^z_{15}\hat{\sigma}^z_{152} - 4\hat{\sigma}^z_{16}\hat{\sigma}^z_{151} - 8\hat{\sigma}^z_{17}\hat{\sigma}^z_{150} - 8\hat{\sigma}^z_{18}\hat{\sigma}^z_{149} - 51664\hat{\sigma}^z_{19}\hat{\sigma}^z_{148} - 4\hat{\sigma}^z_{20}\hat{\sigma}^z_{147} -$$
$$51664\hat{\sigma}^z_{15}\hat{\sigma}^z_{153} - 4\hat{\sigma}^z_{16}\hat{\sigma}^z_{152} - 8\hat{\sigma}^z_{17}\hat{\sigma}^z_{151} - 8\hat{\sigma}^z_{18}\hat{\sigma}^z_{150} - 8\hat{\sigma}^z_{19}\hat{\sigma}^z_{149} - 8\hat{\sigma}^z_{20}\hat{\sigma}^z_{148} - 51664\hat{\sigma}^z_{11}\hat{\sigma}^z_{158} - 8\hat{\sigma}^z_{16}\hat{\sigma}^z_{153} - 4\hat{\sigma}^z_{18}\hat{\sigma}^z_{151} -$$
$$4\hat{\sigma}^z_{19}\hat{\sigma}^z_{150} - 4\hat{\sigma}^z_{20}\hat{\sigma}^z_{149} - 51664\hat{\sigma}^z_{11}\hat{\sigma}^z_{159} - 51664\hat{\sigma}^z_{12}\hat{\sigma}^z_{158} - 51664\hat{\sigma}^z_{16}\hat{\sigma}^z_{154} - 8\hat{\sigma}^z_{17}\hat{\sigma}^z_{153} - 4\hat{\sigma}^z_{18}\hat{\sigma}^z_{152} - 4\hat{\sigma}^z_{19}\hat{\sigma}^z_{151} -$$
$$4\hat{\sigma}^z_{20}\hat{\sigma}^z_{150} + 8\hat{\sigma}^z_{21}\hat{\sigma}^z_{149} - 51664\hat{\sigma}^z_{11}\hat{\sigma}^z_{160} - 16\hat{\sigma}^z_{13}\hat{\sigma}^z_{158} - 8\hat{\sigma}^z_{17}\hat{\sigma}^z_{154} - 4\hat{\sigma}^z_{18}\hat{\sigma}^z_{153} - 4\hat{\sigma}^z_{19}\hat{\sigma}^z_{152} - 4\hat{\sigma}^z_{20}\hat{\sigma}^z_{151} - 51664\hat{\sigma}^z_{11}\hat{\sigma}^z_{161} -$$
$$51664\hat{\sigma}^z_{13}\hat{\sigma}^z_{159} - 24\hat{\sigma}^z_{14}\hat{\sigma}^z_{158} - 51664\hat{\sigma}^z_{17}\hat{\sigma}^z_{155} - 8\hat{\sigma}^z_{18}\hat{\sigma}^z_{154} - 4\hat{\sigma}^z_{19}\hat{\sigma}^z_{153} - 4\hat{\sigma}^z_{20}\hat{\sigma}^z_{152} + 8\hat{\sigma}^z_{22}\hat{\sigma}^z_{150} - 51664\hat{\sigma}^z_{11}\hat{\sigma}^z_{162} -$$
$$16\hat{\sigma}^z_{14}\hat{\sigma}^z_{159} - 8\hat{\sigma}^z_{15}\hat{\sigma}^z_{158} - 8\hat{\sigma}^z_{18}\hat{\sigma}^z_{155} - 4\hat{\sigma}^z_{19}\hat{\sigma}^z_{154} - 4\hat{\sigma}^z_{20}\hat{\sigma}^z_{153} - 51664\hat{\sigma}^z_{11}\hat{\sigma}^z_{163} - 51664\hat{\sigma}^z_{14}\hat{\sigma}^z_{160} - 4\hat{\sigma}^z_{15}\hat{\sigma}^z_{159} -$$
$$8\hat{\sigma}^z_{16}\hat{\sigma}^z_{158} - 51664\hat{\sigma}^z_{18}\hat{\sigma}^z_{156} - 8\hat{\sigma}^z_{19}\hat{\sigma}^z_{155} - 4\hat{\sigma}^z_{20}\hat{\sigma}^z_{154} + 8\hat{\sigma}^z_{23}\hat{\sigma}^z_{151} - 51664\hat{\sigma}^z_{11}\hat{\sigma}^z_{164} - 4\hat{\sigma}^z_{15}\hat{\sigma}^z_{160} - 8\hat{\sigma}^z_{16}\hat{\sigma}^z_{159} - 8\hat{\sigma}^z_{17}\hat{\sigma}^z_{158} -$$
$$8\hat{\sigma}^z_{19}\hat{\sigma}^z_{156} - 4\hat{\sigma}^z_{20}\hat{\sigma}^z_{155} - 51664\hat{\sigma}^z_{11}\hat{\sigma}^z_{165} - 51664\hat{\sigma}^z_{15}\hat{\sigma}^z_{161} - 4\hat{\sigma}^z_{16}\hat{\sigma}^z_{160} - 8\hat{\sigma}^z_{17}\hat{\sigma}^z_{159} - 8\hat{\sigma}^z_{18}\hat{\sigma}^z_{158} - 51664\hat{\sigma}^z_{19}\hat{\sigma}^z_{157} -$$

$$8\hat{\sigma}^z_{20}\hat{\sigma}^z_{156} + 8\hat{\sigma}^z_{54}\hat{\sigma}^z_{122} - 8\hat{\sigma}^z_{16}\hat{\sigma}^z_{161} - 8\hat{\sigma}^z_{17}\hat{\sigma}^z_{160} - 8\hat{\sigma}^z_{18}\hat{\sigma}^z_{159} - 8\hat{\sigma}^z_{19}\hat{\sigma}^z_{158} - 8\hat{\sigma}^z_{20}\hat{\sigma}^z_{157} + 8\hat{\sigma}^z_{38}\hat{\sigma}^z_{139} - 51664\hat{\sigma}^z_{12}\hat{\sigma}^z_{166} -$$
$$51664\hat{\sigma}^z_{16}\hat{\sigma}^z_{162} - 8\hat{\sigma}^z_{17}\hat{\sigma}^z_{161} - 4\hat{\sigma}^z_{18}\hat{\sigma}^z_{160} - 4\hat{\sigma}^z_{19}\hat{\sigma}^z_{159} - 4\hat{\sigma}^z_{20}\hat{\sigma}^z_{158} - 51664\hat{\sigma}^z_{12}\hat{\sigma}^z_{167} - 51664\hat{\sigma}^z_{13}\hat{\sigma}^z_{166} - 8\hat{\sigma}^z_{17}\hat{\sigma}^z_{162} -$$
$$8\hat{\sigma}^z_{18}\hat{\sigma}^z_{161} - 4\hat{\sigma}^z_{19}\hat{\sigma}^z_{160} - 4\hat{\sigma}^z_{20}\hat{\sigma}^z_{159} + 8\hat{\sigma}^z_{21}\hat{\sigma}^z_{158} + 8\hat{\sigma}^z_{39}\hat{\sigma}^z_{140} - 51664\hat{\sigma}^z_{12}\hat{\sigma}^z_{168} - 16\hat{\sigma}^z_{14}\hat{\sigma}^z_{166} - 51664\hat{\sigma}^z_{17}\hat{\sigma}^z_{163} -$$
$$8\hat{\sigma}^z_{18}\hat{\sigma}^z_{162} - 4\hat{\sigma}^z_{19}\hat{\sigma}^z_{161} - 4\hat{\sigma}^z_{20}\hat{\sigma}^z_{160} - 51664\hat{\sigma}^z_{12}\hat{\sigma}^z_{169} - 51664\hat{\sigma}^z_{14}\hat{\sigma}^z_{167} - 8\hat{\sigma}^z_{15}\hat{\sigma}^z_{166} - 8\hat{\sigma}^z_{18}\hat{\sigma}^z_{163} - 8\hat{\sigma}^z_{19}\hat{\sigma}^z_{162} + 8\hat{\sigma}^z_{22}\hat{\sigma}^z_{159} -$$
$$51664\hat{\sigma}^z_{12}\hat{\sigma}^z_{170} - 4\hat{\sigma}^z_{15}\hat{\sigma}^z_{167} - 8\hat{\sigma}^z_{16}\hat{\sigma}^z_{166} - 51664\hat{\sigma}^z_{18}\hat{\sigma}^z_{164} - 8\hat{\sigma}^z_{19}\hat{\sigma}^z_{163} - 4\hat{\sigma}^z_{20}\hat{\sigma}^z_{162} - 51664\hat{\sigma}^z_{12}\hat{\sigma}^z_{171} - 51664\hat{\sigma}^z_{15}\hat{\sigma}^z_{168} -$$
$$8\hat{\sigma}^z_{16}\hat{\sigma}^z_{167} - 8\hat{\sigma}^z_{17}\hat{\sigma}^z_{166} - 8\hat{\sigma}^z_{19}\hat{\sigma}^z_{164} - 8\hat{\sigma}^z_{20}\hat{\sigma}^z_{163} + 8\hat{\sigma}^z_{23}\hat{\sigma}^z_{160} - 51664\hat{\sigma}^z_{12}\hat{\sigma}^z_{172} - 8\hat{\sigma}^z_{16}\hat{\sigma}^z_{168} - 8\hat{\sigma}^z_{17}\hat{\sigma}^z_{167} - 8\hat{\sigma}^z_{18}\hat{\sigma}^z_{166} -$$
$$51664\hat{\sigma}^z_{19}\hat{\sigma}^z_{165} - 8\hat{\sigma}^z_{20}\hat{\sigma}^z_{164} - 51664\hat{\sigma}^z_{16}\hat{\sigma}^z_{169} - 8\hat{\sigma}^z_{17}\hat{\sigma}^z_{168} - 8\hat{\sigma}^z_{18}\hat{\sigma}^z_{167} - 8\hat{\sigma}^z_{19}\hat{\sigma}^z_{166} - 8\hat{\sigma}^z_{20}\hat{\sigma}^z_{165} - 51664\hat{\sigma}^z_{13}\hat{\sigma}^z_{173} -$$
$$8\hat{\sigma}^z_{17}\hat{\sigma}^z_{169} - 8\hat{\sigma}^z_{18}\hat{\sigma}^z_{168} - 4\hat{\sigma}^z_{19}\hat{\sigma}^z_{167} - 4\hat{\sigma}^z_{20}\hat{\sigma}^z_{166} - 51664\hat{\sigma}^z_{13}\hat{\sigma}^z_{174} - 51664\hat{\sigma}^z_{14}\hat{\sigma}^z_{173} - 51664\hat{\sigma}^z_{17}\hat{\sigma}^z_{170} - 8\hat{\sigma}^z_{18}\hat{\sigma}^z_{169} -$$
$$4\hat{\sigma}^z_{19}\hat{\sigma}^z_{168} - 4\hat{\sigma}^z_{20}\hat{\sigma}^z_{167} + 8\hat{\sigma}^z_{21}\hat{\sigma}^z_{166} + 8\hat{\sigma}^z_{38}\hat{\sigma}^z_{149} - 51664\hat{\sigma}^z_{13}\hat{\sigma}^z_{175} - 8\hat{\sigma}^z_{15}\hat{\sigma}^z_{173} - 8\hat{\sigma}^z_{18}\hat{\sigma}^z_{170} - 8\hat{\sigma}^z_{19}\hat{\sigma}^z_{169} - 51664\hat{\sigma}^z_{13}\hat{\sigma}^z_{176} -$$
$$51664\hat{\sigma}^z_{15}\hat{\sigma}^z_{174} - 8\hat{\sigma}^z_{16}\hat{\sigma}^z_{173} - 51664\hat{\sigma}^z_{18}\hat{\sigma}^z_{171} - 8\hat{\sigma}^z_{19}\hat{\sigma}^z_{170} - 4\hat{\sigma}^z_{20}\hat{\sigma}^z_{169} + 8\hat{\sigma}^z_{22}\hat{\sigma}^z_{167} + 8\hat{\sigma}^z_{39}\hat{\sigma}^z_{150} - 51664\hat{\sigma}^z_{13}\hat{\sigma}^z_{177} -$$
$$8\hat{\sigma}^z_{16}\hat{\sigma}^z_{174} - 8\hat{\sigma}^z_{17}\hat{\sigma}^z_{173} - 8\hat{\sigma}^z_{19}\hat{\sigma}^z_{171} - 8\hat{\sigma}^z_{20}\hat{\sigma}^z_{170} - 51664\hat{\sigma}^z_{13}\hat{\sigma}^z_{178} - 51664\hat{\sigma}^z_{16}\hat{\sigma}^z_{175} - 8\hat{\sigma}^z_{17}\hat{\sigma}^z_{174} - 8\hat{\sigma}^z_{18}\hat{\sigma}^z_{173} -$$
$$51664\hat{\sigma}^z_{19}\hat{\sigma}^z_{172} - 8\hat{\sigma}^z_{20}\hat{\sigma}^z_{171} - 8\hat{\sigma}^z_{17}\hat{\sigma}^z_{175} - 4\hat{\sigma}^z_{18}\hat{\sigma}^z_{174} - 8\hat{\sigma}^z_{19}\hat{\sigma}^z_{173} - 8\hat{\sigma}^z_{20}\hat{\sigma}^z_{172} - 51664\hat{\sigma}^z_{14}\hat{\sigma}^z_{179} - 51664\hat{\sigma}^z_{17}\hat{\sigma}^z_{176} -$$
$$8\hat{\sigma}^z_{18}\hat{\sigma}^z_{175} - 4\hat{\sigma}^z_{19}\hat{\sigma}^z_{174} - 4\hat{\sigma}^z_{20}\hat{\sigma}^z_{173} + 8\hat{\sigma}^z_{54}\hat{\sigma}^z_{139} + 16\hat{\sigma}^z_{83}\hat{\sigma}^z_{110} - 51664\hat{\sigma}^z_{14}\hat{\sigma}^z_{180} - 51664\hat{\sigma}^z_{15}\hat{\sigma}^z_{179} - 8\hat{\sigma}^z_{18}\hat{\sigma}^z_{176} -$$
$$4\hat{\sigma}^z_{19}\hat{\sigma}^z_{175} - 4\hat{\sigma}^z_{20}\hat{\sigma}^z_{174} + 8\hat{\sigma}^z_{21}\hat{\sigma}^z_{173} - 16\hat{\sigma}^z_{83}\hat{\sigma}^z_{111} - 51664\hat{\sigma}^z_{14}\hat{\sigma}^z_{181} - 8\hat{\sigma}^z_{16}\hat{\sigma}^z_{179} - 51664\hat{\sigma}^z_{18}\hat{\sigma}^z_{177} - 8\hat{\sigma}^z_{19}\hat{\sigma}^z_{176} -$$
$$4\hat{\sigma}^z_{20}\hat{\sigma}^z_{175} - 51664\hat{\sigma}^z_{14}\hat{\sigma}^z_{182} - 51664\hat{\sigma}^z_{16}\hat{\sigma}^z_{180} - 4\hat{\sigma}^z_{17}\hat{\sigma}^z_{179} - 8\hat{\sigma}^z_{19}\hat{\sigma}^z_{177} - 4\hat{\sigma}^z_{20}\hat{\sigma}^z_{176} + 8\hat{\sigma}^z_{38}\hat{\sigma}^z_{158} - 51664\hat{\sigma}^z_{14}\hat{\sigma}^z_{183} -$$
$$8\hat{\sigma}^z_{17}\hat{\sigma}^z_{180} - 4\hat{\sigma}^z_{18}\hat{\sigma}^z_{179} - 51664\hat{\sigma}^z_{19}\hat{\sigma}^z_{178} - 8\hat{\sigma}^z_{20}\hat{\sigma}^z_{177} - 51664\hat{\sigma}^z_{17}\hat{\sigma}^z_{181} - 4\hat{\sigma}^z_{18}\hat{\sigma}^z_{180} - 4\hat{\sigma}^z_{19}\hat{\sigma}^z_{179} - 8\hat{\sigma}^z_{20}\hat{\sigma}^z_{178} + 8\hat{\sigma}^z_{39}\hat{\sigma}^z_{159} -$$
$$51664\hat{\sigma}^z_{15}\hat{\sigma}^z_{184} - 8\hat{\sigma}^z_{18}\hat{\sigma}^z_{181} - 4\hat{\sigma}^z_{19}\hat{\sigma}^z_{180} - 4\hat{\sigma}^z_{20}\hat{\sigma}^z_{179} - 51664\hat{\sigma}^z_{15}\hat{\sigma}^z_{17}6 - 51664\hat{\sigma}^z_{16}\hat{\sigma}^z_{184} - 51664\hat{\sigma}^z_{18}\hat{\sigma}^z_{182} - 4\hat{\sigma}^z_{19}\hat{\sigma}^z_{181} -$$
$$4\hat{\sigma}^z_{20}\hat{\sigma}^z_{180} - 51664\hat{\sigma}^z_{15}\hat{\sigma}^z_{186} - 16\hat{\sigma}^z_{17}\hat{\sigma}^z_{184} - 8\hat{\sigma}^z_{19}\hat{\sigma}^z_{182} - 4\hat{\sigma}^z_{20}\hat{\sigma}^z_{181} - 51664\hat{\sigma}^z_{15}\hat{\sigma}^z_{187} - 51664\hat{\sigma}^z_{17}\hat{\sigma}^z_{17}6 - 16\hat{\sigma}^z_{18}\hat{\sigma}^z_{184} -$$
$$51664\hat{\sigma}^z_{19}\hat{\sigma}^z_{183} - 4\hat{\sigma}^z_{20}\hat{\sigma}^z_{182} - 51664\hat{\sigma}^z_{15}\hat{\sigma}^z_{188} - 24\hat{\sigma}^z_{18}\hat{\sigma}^z_{17}6 - 16\hat{\sigma}^z_{19}\hat{\sigma}^z_{184} - 8\hat{\sigma}^z_{20}\hat{\sigma}^z_{183} + 8\hat{\sigma}^z_{54}\hat{\sigma}^z_{149} - 51664\hat{\sigma}^z_{18}\hat{\sigma}^z_{186} -$$
$$8\hat{\sigma}^z_{20}\hat{\sigma}^z_{184} + 8\hat{\sigma}^z_{38}\hat{\sigma}^z_{166} - 51664\hat{\sigma}^z_{16}\hat{\sigma}^z_{189} - 16\hat{\sigma}^z_{19}\hat{\sigma}^z_{186} - 8\hat{\sigma}^z_{20}\hat{\sigma}^z_{17}6 + 8\hat{\sigma}^z_{21}\hat{\sigma}^z_{184} + 16\hat{\sigma}^z_{83}\hat{\sigma}^z_{122} - 51664\hat{\sigma}^z_{16}\hat{\sigma}^z_{190} -$$
$$51664\hat{\sigma}^z_{17}\hat{\sigma}^z_{189} - 51664\hat{\sigma}^z_{19}\hat{\sigma}^z_{187} - 8\hat{\sigma}^z_{20}\hat{\sigma}^z_{186} + 8\hat{\sigma}^z_{39}\hat{\sigma}^z_{167} - 16\hat{\sigma}^z_{84}\hat{\sigma}^z_{122} - 51664\hat{\sigma}^z_{16}\hat{\sigma}^z_{191} - 16\hat{\sigma}^z_{18}\hat{\sigma}^z_{189} - 16\hat{\sigma}^z_{20}\hat{\sigma}^z_{187} +$$
$$8\hat{\sigma}^z_{22}\hat{\sigma}^z_{17}6 - 51664\hat{\sigma}^z_{16}\hat{\sigma}^z_{192} - 51664\hat{\sigma}^z_{18}\hat{\sigma}^z_{190} - 24\hat{\sigma}^z_{19}\hat{\sigma}^z_{189} - 51664\hat{\sigma}^z_{20}\hat{\sigma}^z_{188} - 16\hat{\sigma}^z_{19}\hat{\sigma}^z_{190} - 8\hat{\sigma}^z_{20}\hat{\sigma}^z_{189} + 8\hat{\sigma}^z_{23}\hat{\sigma}^z_{186} -$$
$$51664\hat{\sigma}^z_{17}\hat{\sigma}^z_{193} - 51664\hat{\sigma}^z_{19}\hat{\sigma}^z_{191} - 8\hat{\sigma}^z_{20}\hat{\sigma}^z_{190} + 8\hat{\sigma}^z_{21}\hat{\sigma}^z_{189} - 51664\hat{\sigma}^z_{17}\hat{\sigma}^z_{194} - 51664\hat{\sigma}^z_{18}\hat{\sigma}^z_{193} - 16\hat{\sigma}^z_{20}\hat{\sigma}^z_{191} + 8\hat{\sigma}^z_{38}\hat{\sigma}^z_{173} -$$
$$51664\hat{\sigma}^z_{17}\hat{\sigma}^z_{195} - 16\hat{\sigma}^z_{19}\hat{\sigma}^z_{193} - 51664\hat{\sigma}^z_{20}\hat{\sigma}^z_{192} + 8\hat{\sigma}^z_{22}\hat{\sigma}^z_{190} + 8\hat{\sigma}^z_{54}\hat{\sigma}^z_{158} - 51664\hat{\sigma}^z_{19}\hat{\sigma}^z_{194} - 16\hat{\sigma}^z_{20}\hat{\sigma}^z_{193} - 51664\hat{\sigma}^z_{18}\hat{\sigma}^z_{196} -$$
$$16\hat{\sigma}^z_{20}\hat{\sigma}^z_{194} + 8\hat{\sigma}^z_{21}\hat{\sigma}^z_{193} + 8\hat{\sigma}^z_{23}\hat{\sigma}^z_{191} - 51664\hat{\sigma}^z_{18}\hat{\sigma}^z_{197} - 51664\hat{\sigma}^z_{19}\hat{\sigma}^z_{196} - 51664\hat{\sigma}^z_{20}\hat{\sigma}^z_{195} - 8\hat{\sigma}^z_{20}\hat{\sigma}^z_{196} + 8\hat{\sigma}^z_{22}\hat{\sigma}^z_{194} -$$
$$51664\hat{\sigma}^z_{19}\hat{\sigma}^z_{198} - 51664\hat{\sigma}^z_{20}\hat{\sigma}^z_{197} + 8\hat{\sigma}^z_{21}\hat{\sigma}^z_{196} - 51664\hat{\sigma}^z_{20}\hat{\sigma}^z_{198} + 8\hat{\sigma}^z_{23}\hat{\sigma}^z_{195} + 8\hat{\sigma}^z_{21}\hat{\sigma}^z_{198} + 8\hat{\sigma}^z_{22}\hat{\sigma}^z_{197} + 16\hat{\sigma}^z_{97}\hat{\sigma}^z_{122} +$$
$$8\hat{\sigma}^z_{54}\hat{\sigma}^z_{166} + 8\hat{\sigma}^z_{38}\hat{\sigma}^z_{184} + 8\hat{\sigma}^z_{83}\hat{\sigma}^z_{139} + 8\hat{\sigma}^z_{39}\hat{\sigma}^z_{17}6 + 8\hat{\sigma}^z_{84}\hat{\sigma}^z_{140} - 8\hat{\sigma}^z_{83}\hat{\sigma}^z_{142} - 8\hat{\sigma}^z_{84}\hat{\sigma}^z_{141} - 8\hat{\sigma}^z_{85}\hat{\sigma}^z_{140} - 8\hat{\sigma}^z_{86}\hat{\sigma}^z_{139} + 8\hat{\sigma}^z_{85}\hat{\sigma}^z_{141} +$$
$$8\hat{\sigma}^z_{38}\hat{\sigma}^z_{189} + 8\hat{\sigma}^z_{54}\hat{\sigma}^z_{173} + 8\hat{\sigma}^z_{86}\hat{\sigma}^z_{142} + 8\hat{\sigma}^z_{39}\hat{\sigma}^z_{190} + 8\hat{\sigma}^z_{38}\hat{\sigma}^z_{193} + 8\hat{\sigma}^z_{83}\hat{\sigma}^z_{149} + 8\hat{\sigma}^z_{39}\hat{\sigma}^z_{194} + 8\hat{\sigma}^z_{38}\hat{\sigma}^z_{196} + 8\hat{\sigma}^z_{84}\hat{\sigma}^z_{150} - 8\hat{\sigma}^z_{83}\hat{\sigma}^z_{152} -$$
$$8\hat{\sigma}^z_{84}\hat{\sigma}^z_{151} - 8\hat{\sigma}^z_{85}\hat{\sigma}^z_{150} - 8\hat{\sigma}^z_{86}\hat{\sigma}^z_{149} + 8\hat{\sigma}^z_{38}\hat{\sigma}^z_{198} + 8\hat{\sigma}^z_{39}\hat{\sigma}^z_{197} + 8\hat{\sigma}^z_{85}\hat{\sigma}^z_{151} + 8\hat{\sigma}^z_{97}\hat{\sigma}^z_{139} + 8\hat{\sigma}^z_{54}\hat{\sigma}^z_{184} + 8\hat{\sigma}^z_{86}\hat{\sigma}^z_{152} + 8\hat{\sigma}^z_{98}\hat{\sigma}^z_{140} +$$
$$8\hat{\sigma}^z_{99}\hat{\sigma}^z_{141} + 8\hat{\sigma}^z_{83}\hat{\sigma}^z_{158} + 8\hat{\sigma}^z_{54}\hat{\sigma}^z_{189} + 8\hat{\sigma}^z_{84}\hat{\sigma}^z_{159} - 8\hat{\sigma}^z_{84}\hat{\sigma}^z_{160} - 8\hat{\sigma}^z_{85}\hat{\sigma}^z_{159} - 8\hat{\sigma}^z_{86}\hat{\sigma}^z_{158} + 8\hat{\sigma}^z_{85}\hat{\sigma}^z_{160} + 8\hat{\sigma}^z_{97}\hat{\sigma}^z_{149} + 8\hat{\sigma}^z_{54}\hat{\sigma}^z_{193} +$$
$$8\hat{\sigma}^z_{98}\hat{\sigma}^z_{150} + 8\hat{\sigma}^z_{83}\hat{\sigma}^z_{166} + 8\hat{\sigma}^z_{110}\hat{\sigma}^z_{139} + 8\hat{\sigma}^z_{54}\hat{\sigma}^z_{196} + 8\hat{\sigma}^z_{99}\hat{\sigma}^z_{151} + 8\hat{\sigma}^z_{84}\hat{\sigma}^z_{167} + 8\hat{\sigma}^z_{111}\hat{\sigma}^z_{140} + 8\hat{\sigma}^z_{54}\hat{\sigma}^z_{198} - 8\hat{\sigma}^z_{85}\hat{\sigma}^z_{167} - 8\hat{\sigma}^z_{86}\hat{\sigma}^z_{166} +$$
$$8\hat{\sigma}^z_{97}\hat{\sigma}^z_{158} + 8\hat{\sigma}^z_{83}\hat{\sigma}^z_{173} + 8\hat{\sigma}^z_{98}\hat{\sigma}^z_{159} - 8\hat{\sigma}^z_{86}\hat{\sigma}^z_{173} + 8\hat{\sigma}^z_{99}\hat{\sigma}^z_{160} + 8\hat{\sigma}^z_{110}\hat{\sigma}^z_{149} + 8\hat{\sigma}^z_{111}\hat{\sigma}^z_{150} + 8\hat{\sigma}^z_{122}\hat{\sigma}^z_{139} + 8\hat{\sigma}^z_{97}\hat{\sigma}^z_{166} +$$
$$8\hat{\sigma}^z_{98}\hat{\sigma}^z_{167} + 8\hat{\sigma}^z_{83}\hat{\sigma}^z_{184} + 8\hat{\sigma}^z_{110}\hat{\sigma}^z_{158} + 8\hat{\sigma}^z_{84}\hat{\sigma}^z_{17}6 - 8\hat{\sigma}^z_{83}\hat{\sigma}^z_{187} - 8\hat{\sigma}^z_{84}\hat{\sigma}^z_{186} - 8\hat{\sigma}^z_{85}\hat{\sigma}^z_{17}6 - 8\hat{\sigma}^z_{86}\hat{\sigma}^z_{184} + 8\hat{\sigma}^z_{97}\hat{\sigma}^z_{173} +$$
$$8\hat{\sigma}^z_{111}\hat{\sigma}^z_{159} + 8\hat{\sigma}^z_{85}\hat{\sigma}^z_{186} + 8\hat{\sigma}^z_{122}\hat{\sigma}^z_{149} + 8\hat{\sigma}^z_{83}\hat{\sigma}^z_{189} + 8\hat{\sigma}^z_{86}\hat{\sigma}^z_{187} + 8\hat{\sigma}^z_{84}\hat{\sigma}^z_{190} - 8\hat{\sigma}^z_{83}\hat{\sigma}^z_{192} - 8\hat{\sigma}^z_{84}\hat{\sigma}^z_{191} - 8\hat{\sigma}^z_{85}\hat{\sigma}^z_{190} -$$
$$8\hat{\sigma}^z_{86}\hat{\sigma}^z_{189} + 8\hat{\sigma}^z_{83}\hat{\sigma}^z_{193} + 8\hat{\sigma}^z_{85}\hat{\sigma}^z_{191} + 8\hat{\sigma}^z_{110}\hat{\sigma}^z_{166} + 8\hat{\sigma}^z_{84}\hat{\sigma}^z_{194} + 8\hat{\sigma}^z_{86}\hat{\sigma}^z_{192} + 8\hat{\sigma}^z_{111}\hat{\sigma}^z_{167} + 8\hat{\sigma}^z_{83}\hat{\sigma}^z_{196} - 8\hat{\sigma}^z_{84}\hat{\sigma}^z_{195} -$$
$$8\hat{\sigma}^z_{85}\hat{\sigma}^z_{194} - 8\hat{\sigma}^z_{86}\hat{\sigma}^z_{193} + 8\hat{\sigma}^z_{85}\hat{\sigma}^z_{195} + 8\hat{\sigma}^z_{122}\hat{\sigma}^z_{158} + 8\hat{\sigma}^z_{83}\hat{\sigma}^z_{198} + 8\hat{\sigma}^z_{84}\hat{\sigma}^z_{197} + 8\hat{\sigma}^z_{97}\hat{\sigma}^z_{184} - 8\hat{\sigma}^z_{85}\hat{\sigma}^z_{197} - 8\hat{\sigma}^z_{86}\hat{\sigma}^z_{196} + 8\hat{\sigma}^z_{98}\hat{\sigma}^z_{17}6 +$$
$$8\hat{\sigma}^z_{110}\hat{\sigma}^z_{173} - 8\hat{\sigma}^z_{86}\hat{\sigma}^z_{198} + 8\hat{\sigma}^z_{99}\hat{\sigma}^z_{186} + 8\hat{\sigma}^z_{97}\hat{\sigma}^z_{189} + 8\hat{\sigma}^z_{98}\hat{\sigma}^z_{190} + 8\hat{\sigma}^z_{122}\hat{\sigma}^z_{166} + 8\hat{\sigma}^z_{97}\hat{\sigma}^z_{193} + 8\hat{\sigma}^z_{99}\hat{\sigma}^z_{191} + 8\hat{\sigma}^z_{98}\hat{\sigma}^z_{194} +$$
$$8\hat{\sigma}^z_{97}\hat{\sigma}^z_{196} + 8\hat{\sigma}^z_{99}\hat{\sigma}^z_{195} + 8\hat{\sigma}^z_{110}\hat{\sigma}^z_{184} + 8\hat{\sigma}^z_{97}\hat{\sigma}^z_{198} + 8\hat{\sigma}^z_{98}\hat{\sigma}^z_{197} + 8\hat{\sigma}^z_{122}\hat{\sigma}^z_{173} + 8\hat{\sigma}^z_{111}\hat{\sigma}^z_{17}6 + 16\hat{\sigma}^z_{139}\hat{\sigma}^z_{158} + 8\hat{\sigma}^z_{110}\hat{\sigma}^z_{189} +$$
$$8\hat{\sigma}^z_{111}\hat{\sigma}^z_{190} + 8\hat{\sigma}^z_{110}\hat{\sigma}^z_{193} + 8\hat{\sigma}^z_{111}\hat{\sigma}^z_{194} + 16\hat{\sigma}^z_{139}\hat{\sigma}^z_{166} + 8\hat{\sigma}^z_{110}\hat{\sigma}^z_{196} + 8\hat{\sigma}^z_{122}\hat{\sigma}^z_{184} + 16\hat{\sigma}^z_{140}\hat{\sigma}^z_{167} + 8\hat{\sigma}^z_{110}\hat{\sigma}^z_{198} + 8\hat{\sigma}^z_{111}\hat{\sigma}^z_{197} +$$
$$8\hat{\sigma}^z_{122}\hat{\sigma}^z_{189} + 16\hat{\sigma}^z_{139}\hat{\sigma}^z_{173} + 8\hat{\sigma}^z_{122}\hat{\sigma}^z_{193} + 16\hat{\sigma}^z_{149}\hat{\sigma}^z_{166} + 8\hat{\sigma}^z_{122}\hat{\sigma}^z_{196} + 8\hat{\sigma}^z_{122}\hat{\sigma}^z_{198} + 16\hat{\sigma}^z_{149}\hat{\sigma}^z_{173} + 8\hat{\sigma}^z_{139}\hat{\sigma}^z_{184} + 8\hat{\sigma}^z_{140}\hat{\sigma}^z_{17}6 +$$
$$8\hat{\sigma}^z_{141}\hat{\sigma}^z_{186} + 8\hat{\sigma}^z_{139}\hat{\sigma}^z_{189} + 8\hat{\sigma}^z_{142}\hat{\sigma}^z_{187} + 8\hat{\sigma}^z_{140}\hat{\sigma}^z_{190} + 8\hat{\sigma}^z_{143}\hat{\sigma}^z_{188} + 16\hat{\sigma}^z_{158}\hat{\sigma}^z_{173} + 8\hat{\sigma}^z_{139}\hat{\sigma}^z_{193} + 8\hat{\sigma}^z_{141}\hat{\sigma}^z_{191} + 8\hat{\sigma}^z_{149}\hat{\sigma}^z_{184} +$$
$$8\hat{\sigma}^z_{140}\hat{\sigma}^z_{194} + 8\hat{\sigma}^z_{142}\hat{\sigma}^z_{192} + 8\hat{\sigma}^z_{139}\hat{\sigma}^z_{196} + 8\hat{\sigma}^z_{150}\hat{\sigma}^z_{17}6 + 8\hat{\sigma}^z_{141}\hat{\sigma}^z_{195} + 8\hat{\sigma}^z_{139}\hat{\sigma}^z_{198} + 8\hat{\sigma}^z_{140}\hat{\sigma}^z_{197} + 8\hat{\sigma}^z_{151}\hat{\sigma}^z_{186} + 8\hat{\sigma}^z_{149}\hat{\sigma}^z_{189} +$$
$$8\hat{\sigma}^z_{152}\hat{\sigma}^z_{187} + 8\hat{\sigma}^z_{150}\hat{\sigma}^z_{190} + 8\hat{\sigma}^z_{149}\hat{\sigma}^z_{193} + 8\hat{\sigma}^z_{151}\hat{\sigma}^z_{191} + 8\hat{\sigma}^z_{158}\hat{\sigma}^z_{184} + 8\hat{\sigma}^z_{150}\hat{\sigma}^z_{194} + 8\hat{\sigma}^z_{152}\hat{\sigma}^z_{192} + 8\hat{\sigma}^z_{159}\hat{\sigma}^z_{17}6 + 8\hat{\sigma}^z_{149}\hat{\sigma}^z_{196} +$$
$$8\hat{\sigma}^z_{151}\hat{\sigma}^z_{195} + 8\hat{\sigma}^z_{160}\hat{\sigma}^z_{186} + 8\hat{\sigma}^z_{149}\hat{\sigma}^z_{198} + 8\hat{\sigma}^z_{150}\hat{\sigma}^z_{197} + 8\hat{\sigma}^z_{158}\hat{\sigma}^z_{189} + 8\hat{\sigma}^z_{159}\hat{\sigma}^z_{190} + 8\hat{\sigma}^z_{166}\hat{\sigma}^z_{184} + 8\hat{\sigma}^z_{158}\hat{\sigma}^z_{193} + 8\hat{\sigma}^z_{160}\hat{\sigma}^z_{191} +$$
$$8\hat{\sigma}^z_{167}\hat{\sigma}^z_{17}6 + 8\hat{\sigma}^z_{159}\hat{\sigma}^z_{194} + 8\hat{\sigma}^z_{158}\hat{\sigma}^z_{196} + 8\hat{\sigma}^z_{160}\hat{\sigma}^z_{195} + 8\hat{\sigma}^z_{166}\hat{\sigma}^z_{189} + 8\hat{\sigma}^z_{158}\hat{\sigma}^z_{198} + 8\hat{\sigma}^z_{159}\hat{\sigma}^z_{197} + 8\hat{\sigma}^z_{167}\hat{\sigma}^z_{190} + 8\hat{\sigma}^z_{173}\hat{\sigma}^z_{184} +$$
$$8\hat{\sigma}^z_{166}\hat{\sigma}^z_{193} + 8\hat{\sigma}^z_{167}\hat{\sigma}^z_{194} + 8\hat{\sigma}^z_{166}\hat{\sigma}^z_{196} + 8\hat{\sigma}^z_{173}\hat{\sigma}^z_{189} + 8\hat{\sigma}^z_{166}\hat{\sigma}^z_{198} + 8\hat{\sigma}^z_{167}\hat{\sigma}^z_{197} + 8\hat{\sigma}^z_{173}\hat{\sigma}^z_{193} + 8\hat{\sigma}^z_{173}\hat{\sigma}^z_{196} + 8\hat{\sigma}^z_{173}\hat{\sigma}^z_{198} +$$
$$16\hat{\sigma}^z_{184}\hat{\sigma}^z_{193} + 16\hat{\sigma}^z_{184}\hat{\sigma}^z_{196} + 16\hat{\sigma}^z_{184}\hat{\sigma}^z_{198} + 16\hat{\sigma}^z_{17}6\hat{\sigma}^z_{197} + 16\hat{\sigma}^z_{189}\hat{\sigma}^z_{196} + 16\hat{\sigma}^z_{189}\hat{\sigma}^z_{198} + 16\hat{\sigma}^z_{193}\hat{\sigma}^z_{198} + 13798138$$

Complete Expression in the Extended Turyn Based Method

A complete expression of Eq.(??)

$$
\begin{aligned}
E_k(s) = {} & 4s_2 - 14s_0 - 14s_3 + 18s_4 - 4s_5 + 4s_6 - 18s_7 + 24s_8 + 24s_{11} + 40s_{12} - 8s_{13} + 16s_{14} - 40s_{15} - 16s_0s_1 + \\
& 6s_0s_2 - 16s_1s_2 + 4s_0s_4 - 2s_1s_3 - 4s_0s_5 - 16s_2s_3 + 4s_0s_6 + 8s_1s_5 + 4s_2s_4 - 4s_0s_7 - 8s_1s_6 - 4s_2s_5 - 4s_3s_4 - 8s_0s_8 + \\
& 4s_2s_6 + 8s_1s_8 - 4s_2s_7 - 20s_4s_5 - 8s_1s_9 + 4s_3s_7 + 6s_4s_6 - 8s_0s_{11} - 8s_1s_{10} - 8s_3s_8 - 8s_4s_7 - 28s_5s_6 + 4s_0s_{12} + 8s_1s_{11} + \\
& 8s_3s_9 + 6s_5s_7 - 4s_0s_{13} + 8s_3s_{10} - 20s_6s_7 + 8s_0s_{14} + 12s_1s_{13} + 8s_2s_{12} - 8s_3s_{11} - 4s_0s_{15} - 8s_1s_{14} - 4s_2s_{13} - 4s_3s_{12} + \\
& 4s_1s_{15} + 8s_2s_{14} + 4s_3s_{13} + 12s_4s_{12} - 4s_2s_{15} - 4s_4s_{13} - 8s_5s_{12} - 48s_8s_9 + 4s_3s_{15} + 8s_4s_{14} + 20s_5s_{13} + 8s_6s_{12} - 8s_8s_{10} - \\
& 12s_4s_{15} - 16s_5s_{14} - 20s_6s_{13} - 12s_7s_{12} + 32s_8s_{11} - 16s_9s_{10} + 4s_5s_{15} + 16s_6s_{14} + 4s_7s_{13} + 8s_8s_{12} - 8s_9s_{11} - 4s_6s_{15} - \\
& 8s_7s_{14} - 8s_8s_{13} - 48s_{10}s_{11} + 12s_7s_{15} + 8s_9s_{13} + 8s_8s_{15} + 8s_{10}s_{13} + 8s_{11}s_{12} - 8s_9s_{15} - 8s_{11}s_{13} - 8s_{10}s_{15} - 40s_{12}s_{13} + \\
& 8s_{11}s_{15} + 24s_{12}s_{14} - 16s_{12}s_{15} - 56s_{13}s_{14} + 8s_{13}s_{15} - 40s_{14}s_{15} + 4s_0s_1s_2 + 8s_0s_1s_3 - 2s_0s_1s_4 + 2s_0s_2s_4 + 4s_1s_2s_3 - \\
& 2s_0s_2s_5 - 2s_1s_2s_4 + 2s_0s_1s_7 + 2s_0s_2s_6 + 4s_0s_3s_5 + 2s_1s_3s_4 - 4s_0s_1s_8 - 2s_0s_2s_7 - 4s_0s_3s_6 + 2s_0s_4s_5 - 2s_1s_3s_5 - \\
& 2s_2s_3s_4 + 4s_0s_2s_8 + 2s_0s_4s_6 + 2s_1s_2s_7 + 2s_1s_3s_6 - 4s_0s_2s_9 + 2s_0s_5s_6 - 4s_1s_2s_8 - 2s_1s_3s_7 + 2s_1s_4s_6 - 4s_0s_1s_{11} - \\
& 4s_0s_2s_{10} + 2s_0s_5s_7 + 4s_1s_3s_8 + 4s_1s_4s_7 + 2s_2s_3s_7 + 2s_2s_4s_6 + 2s_3s_4s_5 - 4s_0s_1s_{12} + 4s_0s_2s_{11} + 2s_0s_6s_7 - 4s_1s_3s_9 + \\
& 2s_1s_5s_7 - 4s_2s_3s_8 - 2s_3s_4s_6 + 4s_0s_2s_{12} - 4s_1s_2s_{11} - 4s_1s_3s_{10} + 2s_2s_5s_7 + 2s_3s_5s_6 - 4s_0s_2s_{13} - 4s_1s_2s_{12} + 4s_1s_3s_{11} - \\
& 2s_3s_5s_7 - 4s_4s_5s_6 + 4s_0s_1s_{15} + 4s_0s_2s_{14} + 8s_0s_3s_{13} + 4s_1s_3s_{12} - 4s_2s_3s_{11} + 2s_3s_6s_7 + 8s_4s_5s_7 - 4s_0s_3s_{14} + 4s_0s_8s_9 - \\
& 4s_1s_3s_{13} - 4s_2s_3s_{12} - 4s_4s_5s_8 - 8s_4s_6s_7 + 4s_0s_8s_{10} + 4s_1s_2s_{15} + 4s_1s_3s_{14} + 4s_4s_6s_8 + 4s_5s_6s_7 + 4s_0s_9s_{10} + 4s_1s_8s_{10} - \\
& 4s_4s_6s_9 - 4s_5s_6s_8 + 4s_0s_9s_{11} + 8s_1s_8s_{11} + 4s_2s_3s_{15} + 4s_2s_8s_{10} + 4s_3s_8s_9 - 4s_4s_5s_{11} - 4s_4s_6s_{10} + 4s_5s_7s_8 + 4s_0s_{10}s_{11} + \\
& 4s_1s_9s_{11} - 4s_3s_8s_{10} - 4s_4s_5s_{12} + 4s_4s_6s_{11} - 4s_4s_8s_9 - 4s_5s_7s_9 - 4s_6s_7s_8 + 4s_2s_9s_{11} + 4s_3s_9s_{10} + 4s_4s_6s_{12} + \\
& 4s_4s_8s_{10} - 4s_5s_6s_{11} - 4s_5s_7s_{10} - 4s_3s_9s_{11} - 4s_4s_6s_{13} - 4s_4s_9s_{10} - 4s_5s_6s_{12} + 4s_5s_7s_{11} - 4s_5s_8s_{10} + 4s_3s_{10}s_{11} + \\
& 4s_4s_5s_{15} + 4s_4s_6s_{14} + 8s_4s_7s_{13} + 4s_4s_9s_{11} + 4s_5s_7s_{12} + 8s_5s_8s_{11} - 4s_6s_7s_{11} + 4s_6s_8s_{10} + 4s_7s_8s_9 + 4s_0s_{12}s_{13} - \\
& 4s_4s_7s_{14} - 4s_4s_{10}s_{11} - 4s_5s_7s_{13} - 4s_5s_9s_{11} - 4s_6s_7s_{12} - 8s_6s_8s_{11} - 4s_7s_8s_{10} + 4s_0s_{12}s_{14} + 4s_5s_6s_{15} + 4s_5s_7s_{14} + \\
& 4s_6s_9s_{11} + 4s_7s_9s_{10} + 4s_0s_{13}s_{14} + 4s_1s_{12}s_{14} - 4s_7s_9s_{11} - 16s_8s_9s_{10} + 4s_0s_{13}s_{15} + 8s_1s_{12}s_{15} + 4s_2s_{12}s_{14} + 4s_3s_{12}s_{13} + \\
& 4s_6s_7s_{15} + 4s_7s_{10}s_{11} + 4s_0s_{14}s_{15} + 4s_1s_{13}s_{15} - 4s_3s_{12}s_{14} - 4s_4s_{12}s_{13} - 8s_8s_9s_{12} + 4s_2s_{13}s_{15} + 4s_3s_{13}s_{14} + 4s_4s_{12}s_{14} + \\
& 8s_8s_{10}s_{12} - 16s_9s_{10}s_{11} - 4s_3s_{13}s_{15} - 4s_4s_{13}s_{14} - 4s_5s_{12}s_{14} - 8s_8s_{10}s_{13} - 8s_9s_{10}s_{12} + 4s_3s_{14}s_{15} + 4s_4s_{13}s_{15} + \\
& 8s_5s_{12}s_{15} + 4s_6s_{12}s_{14} + 4s_7s_{12}s_{13} + 8s_8s_9s_{15} + 8s_8s_{10}s_{14} + 16s_8s_{11}s_{13} + 8s_9s_{11}s_{12} - 4s_4s_{14}s_{15} - 4s_5s_{13}s_{15} - \\
& 8s_6s_{12}s_{15} - 4s_7s_{12}s_{14} - 8s_8s_{11}s_{14} - 8s_8s_{12}s_{13} - 8s_9s_{11}s_{13} - 8s_{10}s_{11}s_{12} + 4s_6s_{13}s_{15} + 4s_7s_{13}s_{14} + 8s_8s_{12}s_{14} + \\
& 8s_9s_{10}s_{15} + 8s_9s_{11}s_{14} - 4s_7s_{13}s_{15} - 8s_8s_{13}s_{14} - 8s_9s_{12}s_{14} + 4s_7s_{14}s_{15} + 8s_8s_{13}s_{15} + 8s_{10}s_{11}s_{15} - 8s_{10}s_{12}s_{14} - \\
& 8s_{11}s_{12}s_{13} - 8s_8s_{14}s_{15} - 8s_9s_{13}s_{15} + 8s_{11}s_{12}s_{14} - 8s_{10}s_{13}s_{15} - 8s_{11}s_{13}s_{14} + 8s_{11}s_{13}s_{15} - 16s_{12}s_{13}s_{14} - 8s_{11}s_{14}s_{15} + \\
& 32s_{12}s_{13}s_{15} - 16s_{12}s_{14}s_{15} + 16s_{13}s_{14}s_{15} + 4s_0s_1s_2s_3 + 2s_0s_1s_4s_5 + 2s_0s_1s_5s_6 + 2s_0s_2s_4s_6 + 2s_1s_2s_4s_5 + 2s_0s_1s_6s_7 + \\
& 2s_0s_2s_5s_7 + 2s_0s_3s_4s_7 + 2s_1s_2s_5s_6 + 2s_1s_3s_4s_6 + 2s_2s_3s_4s_5 + 2s_1s_2s_6s_7 + 2s_1s_3s_5s_7 + 2s_2s_3s_5s_6 + 4s_0s_1s_8s_9 + \\
& 2s_2s_3s_6s_7 + 4s_0s_1s_9s_{10} + 4s_0s_2s_8s_{10} + 4s_1s_2s_8s_9 + 4s_0s_1s_{10}s_{11} + 4s_0s_2s_9s_{11} + 4s_0s_3s_8s_{11} + 4s_1s_2s_9s_{10} + 4s_1s_3s_8s_{10} + \\
& 4s_2s_3s_8s_9 + 4s_4s_5s_6s_7 + 4s_1s_2s_{10}s_{11} + 4s_1s_3s_9s_{11} + 4s_2s_3s_9s_{10} + 4s_0s_1s_{12}s_{13} + 4s_2s_3s_{10}s_{11} + 4s_4s_5s_8s_9 + 4s_0s_1s_{13}s_{14} + \\
& 4s_0s_2s_{12}s_{14} + 4s_1s_2s_{12}s_{13} + 4s_4s_5s_9s_{10} + 4s_4s_6s_8s_{10} + 4s_5s_6s_8s_9 + 4s_0s_1s_{14}s_{15} + 4s_0s_2s_{13}s_{15} + 4s_0s_3s_{12}s_{15} + \\
& 4s_1s_2s_{13}s_{14} + 4s_1s_3s_{12}s_{14} + 4s_2s_3s_{12}s_{13} + 4s_4s_5s_{10}s_{11} + 4s_4s_6s_9s_{11} + 4s_4s_7s_8s_{11} + 4s_5s_6s_9s_{10} + 4s_5s_7s_8s_{10} + \\
& 4s_6s_7s_8s_9 + 4s_1s_2s_{14}s_{15} + 4s_1s_3s_{13}s_{15} + 4s_2s_3s_{13}s_{14} + 4s_5s_6s_{10}s_{11} + 4s_5s_7s_9s_{11} + 4s_6s_7s_9s_{10} + 4s_2s_3s_{14}s_{15} + \\
& 4s_4s_5s_{12}s_{13} + 4s_6s_7s_{10}s_{11} + 4s_4s_5s_{13}s_{14} + 4s_4s_6s_{12}s_{14} + 4s_5s_6s_{12}s_{13} + 4s_4s_5s_{14}s_{15} + 4s_4s_6s_{13}s_{15} + 4s_4s_7s_{12}s_{15} + \\
& 4s_5s_6s_{13}s_{14} + 4s_5s_7s_{12}s_{14} + 4s_6s_7s_{12}s_{13} + 16s_8s_9s_{10}s_{11} + 4s_5s_6s_{14}s_{15} + 4s_5s_7s_{13}s_{15} + 4s_6s_7s_{13}s_{14} + 4s_6s_7s_{14}s_{15} + \\
& 8s_8s_9s_{12}s_{13} + 8s_8s_9s_{13}s_{14} + 8s_8s_{10}s_{12}s_{14} + 8s_9s_{10}s_{12}s_{13} + 8s_8s_9s_{14}s_{15} + 8s_8s_{10}s_{13}s_{15} + 8s_8s_{11}s_{12}s_{15} + 8s_9s_{10}s_{13}s_{14} + \\
& 8s_9s_{11}s_{12}s_{14} + 8s_{10}s_{11}s_{12}s_{13} + 8s_9s_{10}s_{14}s_{15} + 8s_9s_{11}s_{13}s_{15} + 8s_{10}s_{11}s_{13}s_{14} + 8s_{10}s_{11}s_{14}s_{15} + 16s_{12}s_{13}s_{14}s_{15} + 264
\end{aligned}
$$

A complete expression of $E_k(q)$ in 92-order H matrix using extended Turyn based method.

$$
\begin{aligned}
E_k(q) = {} & 88q_0q_1 - 204q_1 - 172q_2 - 116q_3 - 132q_4 - 92q_5 - 76q_6 - 84q_7 - 240q_8 - 80q_9 - 80q_{10} - 240q_{11} - \\
& 272q_{12} - 160q_{13} - 176q_{14} - 224q_{15} - 180q_0 + 152q_0q_2 + 104q_0q_3 + 72q_1q_2 + 56q_0q_4 + 152q_1q_3 + 40q_0q_5 + \\
& 40q_1q_4 + 56q_2q_3 + 56q_0q_6 + 72q_1q_5 + 40q_2q_4 + 24q_0q_7 + 24q_1q_6 + 24q_2q_5 + 8q_3q_4 + 48q_0q_8 + 56q_1q_7 + 72q_2q_6 + \\
& 40q_3q_5 + 80q_0q_9 + 112q_1q_8 + 24q_2q_7 + 24q_3q_6 + 56q_4q_5 + 80q_0q_{10} + 48q_1q_9 + 48q_2q_8 + 40q_3q_7 + 104q_4q_6 + \\
& 48q_0q_{11} + 48q_1q_{10} + 80q_2q_9 + 16q_3q_8 + 56q_4q_7 + 8q_5q_6 + 96q_0q_{12} + 112q_1q_{11} + 80q_2q_{10} + 80q_3q_9 + 48q_4q_8 +
\end{aligned}
$$

$200q_5q_7+96q_0q_{13}+80q_1q_{12}+48q_2q_{11}+80q_3q_{10}+16q_4q_9+48q_5q_8+24q_6q_7+128q_0q_{14}+128q_1q_{13}+80q_2q_{12}+$
$16q_3q_{11}+16q_4q_{10}+48q_5q_9+16q_6q_8+80q_0q_{15}+80q_1q_{14}+64q_2q_{13}+32q_3q_{12}+48q_4q_{11}+48q_5q_{10}+80q_6q_9+$
$48q_7q_8+144q_1q_{15}+144q_2q_{14}+96q_3q_{13}+96q_4q_{12}+48q_5q_{11}+80q_6q_{10}+48q_7q_9+80q_2q_{15}+64q_3q_{14}+$
$32q_4q_{13}+16q_5q_{12}+16q_6q_{11}+48q_7q_{10}+32q_8q_9+80q_3q_{15}+64q_4q_{14}+128q_5q_{13}+48q_6q_{12}+48q_7q_{11}+$
$160q_8q_{10}+16q_4q_{15}+16q_5q_{14}+320q_8q_{11}+96q_9q_{10}+112q_5q_{15}+176q_6q_{14}+96q_7q_{13}+128q_8q_{12}+160q_9q_{11}+$
$48q_6q_{15}+32q_7q_{14}+64q_8q_{13}+32q_9q_{12}+32q_{10}q_{11}+112q_7q_{15}+64q_8q_{14}+128q_9q_{13}+32q_{10}q_{12}+160q_8q_{15}+$
$160q_9q_{14}+128q_{10}q_{13}+128q_{11}q_{12}+96q_9q_{15}+160q_{10}q_{14}+64q_{11}q_{13}+96q_{10}q_{15}+64q_{11}q_{14}+128q_{12}q_{13}+$
$160q_{11}q_{15}+192q_{12}q_{14}+160q_{12}q_{15}+448q_{13}q_{15}+64q_{14}q_{15}-64q_0q_1q_2-96q_0q_1q_3-32q_0q_2q_3-32q_0q_1q_5-$
$32q_0q_2q_4-64q_1q_2q_3-32q_0q_1q_6-16q_0q_3q_4-32q_0q_1q_7-32q_0q_2q_6-32q_0q_3q_5-32q_1q_2q_5-32q_1q_3q_4+$
$32q_0q_3q_6-32q_0q_4q_5-32q_1q_2q_6-64q_0q_1q_9-64q_0q_2q_8-16q_0q_3q_7-32q_0q_4q_6-32q_1q_2q_7-32q_1q_3q_6-$
$32q_1q_4q_5-32q_2q_3q_5-64q_0q_1q_{10}-32q_0q_3q_8-16q_0q_4q_7-32q_0q_5q_6-32q_1q_4q_6-32q_2q_3q_6-32q_2q_4q_5-$
$32q_0q_5q_7-64q_1q_2q_9-64q_1q_3q_8-32q_1q_4q_7-32q_1q_5q_6-32q_2q_3q_7-32q_2q_4q_6-32q_3q_4q_5-64q_0q_2q_{11}-$
$32q_0q_6q_7-64q_1q_2q_{10}-32q_1q_5q_7-32q_2q_5q_6-64q_0q_1q_{13}-64q_0q_2q_{12}-32q_0q_3q_{11}-32q_1q_6q_7-64q_2q_3q_9-$
$32q_2q_5q_7-16q_3q_4q_7-32q_3q_5q_6-64q_0q_1q_{14}-32q_0q_3q_{12}-64q_1q_3q_{11}-64q_2q_3q_{10}-32q_2q_6q_7-64q_0q_1q_{15}-$
$64q_0q_2q_{14}-64q_0q_3q_{13}-64q_1q_2q_{13}-64q_1q_3q_{12}-32q_3q_6q_7-96q_4q_5q_7-32q_0q_2q_{15}+32q_0q_3q_{14}-$
$64q_0q_8q_9-64q_1q_2q_{14}+32q_4q_6q_7-32q_0q_3q_{15}-64q_0q_8q_{10}-64q_1q_2q_{15}-64q_1q_3q_{14}-64q_1q_8q_9-64q_2q_3q_{13}-$
$64q_4q_5q_9-64q_4q_6q_8-64q_5q_6q_7-32q_0q_8q_{11}-64q_0q_9q_{10}-32q_1q_3q_{15}-64q_1q_8q_{10}-64q_2q_3q_{14}-64q_2q_8q_9-$
$64q_4q_5q_{10}-32q_4q_7q_8-64q_0q_9q_{11}-64q_1q_8q_{11}-64q_1q_9q_{10}-64q_2q_3q_{15}-64q_2q_8q_{10}-64q_3q_8q_9-$
$64q_5q_6q_9-64q_5q_7q_8-64q_0q_{10}q_{11}-64q_1q_9q_{11}-64q_2q_9q_{10}-64q_4q_6q_{11}-64q_5q_6q_{10}-64q_1q_{10}q_{11}-$
$64q_2q_9q_{11}-32q_3q_8q_{11}-64q_3q_9q_{10}-64q_4q_5q_{13}-64q_4q_6q_{12}-32q_4q_7q_{11}-64q_4q_8q_{10}-64q_5q_8q_9-$
$64q_6q_7q_9-64q_2q_{10}q_{11}-64q_4q_5q_{14}-32q_4q_7q_{12}-32q_4q_8q_{11}-64q_5q_7q_{11}-64q_6q_7q_{10}-64q_6q_8q_9-$
$64q_3q_{10}q_{11}-64q_4q_5q_{15}-64q_4q_6q_{14}-64q_4q_7q_{13}-64q_4q_9q_{11}-64q_5q_6q_{13}-64q_5q_7q_{12}-64q_5q_8q_{11}-$
$64q_5q_9q_{10}-64q_6q_8q_{10}-64q_7q_8q_9-64q_0q_{12}q_{13}-32q_4q_6q_{15}+32q_4q_7q_{14}-64q_5q_6q_{14}+64q_6q_8q_{11}-$
$64q_6q_9q_{10}-64q_0q_{12}q_{14}-64q_1q_{12}q_{13}-32q_4q_7q_{15}-64q_5q_6q_{15}-64q_5q_7q_{14}-64q_5q_{10}q_{11}-64q_6q_7q_{13}-$
$64q_6q_9q_{11}-32q_7q_8q_{11}-64q_7q_9q_{10}-32q_0q_{12}q_{15}-64q_0q_{13}q_{14}-64q_1q_{12}q_{14}-64q_2q_{12}q_{13}-32q_5q_7q_{15}-$
$64q_6q_7q_{14}-64q_6q_{10}q_{11}-64q_0q_{13}q_{15}-64q_1q_{12}q_{15}-64q_1q_{13}q_{14}-64q_2q_{12}q_{14}-64q_3q_{12}q_{13}-64q_6q_7q_{15}-$
$64q_7q_{10}q_{11}-128q_8q_9q_{11}-64q_0q_{14}q_{15}-64q_1q_{13}q_{15}-64q_2q_{13}q_{14}-128q_8q_{10}q_{11}-64q_1q_{14}q_{15}-64q_2q_{13}q_{15}-$
$32q_3q_{12}q_{15}-64q_3q_{13}q_{14}-64q_4q_{12}q_{14}-64q_5q_{12}q_{13}-128q_8q_9q_{13}-128q_8q_{10}q_{12}-64q_2q_{14}q_{15}-32q_4q_{12}q_{15}-$
$64q_6q_{12}q_{13}-128q_8q_9q_{14}-64q_8q_{11}q_{12}-64q_3q_{14}q_{15}-64q_4q_{13}q_{15}-64q_5q_{12}q_{15}-64q_5q_{13}q_{14}-64q_6q_{12}q_{14}-$
$64q_7q_{12}q_{13}-128q_8q_9q_{15}-128q_8q_{10}q_{14}-128q_8q_{11}q_{13}-128q_9q_{10}q_{13}-128q_9q_{11}q_{12}+64q_6q_{12}q_{15}-$
$64q_6q_{13}q_{14}-64q_8q_{10}q_{15}+64q_8q_{11}q_{14}-128q_9q_{10}q_{14}-64q_5q_{14}q_{15}-64q_6q_{13}q_{15}-32q_7q_{12}q_{15}-64q_7q_{13}q_{14}-$
$64q_8q_{11}q_{15}-128q_8q_{12}q_{14}-128q_9q_{10}q_{15}-128q_9q_{11}q_{14}-128q_9q_{12}q_{13}-128q_{10}q_{11}q_{13}-64q_6q_{14}q_{15}-$
$64q_8q_{12}q_{15}-64q_9q_{11}q_{15}-128q_{10}q_{11}q_{14}-128q_{10}q_{12}q_{13}-64q_7q_{14}q_{15}-128q_8q_{13}q_{15}-128q_9q_{13}q_{14}-$
$128q_{10}q_{11}q_{15}-128q_{10}q_{13}q_{14}-128q_{11}q_{12}q_{14}-128q_9q_{14}q_{15}-64q_{11}q_{12}q_{15}-128q_{10}q_{14}q_{15}-128q_{11}q_{13}q_{15}-$
$384q_{12}q_{13}q_{15}-256q_{13}q_{14}q_{15}+64q_0q_1q_2q_3+32q_0q_1q_4q_5+32q_0q_1q_5q_6+32q_0q_2q_4q_6+32q_1q_2q_4q_5+$
$32q_0q_1q_6q_7+32q_0q_2q_5q_7+32q_0q_3q_4q_7+32q_1q_2q_5q_6+32q_1q_3q_4q_6+32q_2q_3q_4q_5+32q_1q_2q_6q_7+$
$32q_1q_3q_5q_7+32q_2q_3q_5q_6+64q_0q_1q_8q_9+32q_2q_3q_6q_7+64q_0q_1q_9q_{10}+64q_0q_2q_8q_{10}+64q_1q_2q_8q_9+$
$64q_0q_1q_{10}q_{11}+64q_0q_2q_9q_{11}+64q_0q_3q_8q_{11}+64q_1q_2q_9q_{10}+64q_1q_3q_8q_{10}+64q_2q_3q_8q_9+64q_4q_5q_6q_7+$
$64q_1q_2q_{10}q_{11}+64q_1q_3q_9q_{11}+64q_2q_3q_9q_{10}+64q_0q_1q_{12}q_{13}+64q_2q_3q_{10}q_{11}+64q_4q_5q_8q_9+64q_0q_1q_{13}q_{14}+$
$64q_0q_2q_{12}q_{14}+64q_1q_2q_{12}q_{13}+64q_4q_5q_9q_{10}+64q_4q_6q_8q_{10}+64q_5q_6q_8q_9+64q_0q_1q_{14}q_{15}+64q_0q_2q_{13}q_{15}+$
$64q_0q_3q_{12}q_{15}+64q_1q_2q_{13}q_{14}+64q_1q_3q_{12}q_{14}+64q_2q_3q_{12}q_{13}+64q_4q_5q_{10}q_{11}+64q_4q_6q_9q_{11}+64q_4q_7q_8q_{11}+$
$64q_5q_6q_9q_{10}+64q_5q_7q_8q_{10}+64q_6q_7q_8q_9+64q_1q_2q_{14}q_{15}+64q_1q_3q_{13}q_{15}+64q_2q_3q_{13}q_{14}+64q_5q_6q_{10}q_{11}+$
$64q_5q_7q_9q_{11}+64q_6q_7q_9q_{10}+64q_2q_3q_{14}q_{15}+64q_4q_5q_{12}q_{13}+64q_6q_7q_{10}q_{11}+64q_4q_5q_{13}q_{14}+64q_4q_6q_{12}q_{14}+$
$64q_5q_6q_{12}q_{13}+64q_4q_5q_{14}q_{15}+64q_4q_6q_{13}q_{15}+64q_4q_7q_{12}q_{15}+64q_5q_6q_{13}q_{14}+64q_5q_7q_{12}q_{14}+64q_6q_7q_{12}q_{13}+$
$256q_8q_9q_{10}q_{11}+64q_5q_6q_{14}q_{15}+64q_5q_7q_{13}q_{15}+64q_6q_7q_{13}q_{14}+64q_6q_7q_{14}q_{15}+128q_8q_9q_{12}q_{13}+128q_8q_9q_{13}q_{14}+$
$128q_8q_{10}q_{12}q_{14}+128q_9q_{10}q_{12}q_{13}+128q_8q_9q_{14}q_{15}+128q_8q_{10}q_{13}q_{15}+128q_8q_{11}q_{12}q_{15}+128q_9q_{10}q_{13}q_{14}+$
$128q_9q_{11}q_{12}q_{14}+128q_{10}q_{11}q_{12}q_{13}+128q_9q_{10}q_{14}q_{15}+128q_9q_{11}q_{13}q_{15}+128q_{10}q_{11}q_{13}q_{14}+128q_{10}q_{11}q_{14}q_{15}+$
$256q_{12}q_{13}q_{14}q_{15}+472$

A complete expression of $E_2(q)$ in 92-order H matrix using extended Turyn based method, using $\delta = 65,936$

$E_2(q) = 197896q_{16} - 204q_1 - 172q_2 - 116q_3 - 132q_4 - 92q_5 - 76q_6 - 84q_7 - 240q_8 - 80q_9 - 80q_{10} - 240q_{11} -$
$272q_{12} - 160q_{13} - 176q_{14} - 224q_{15} - 180q_0 + 197960q_{17} + 197912q_{18} + 197864q_{19} + 197848q_{20} + 197864q_{21} +$
$197856q_{22} + 197888q_{23} + 197888q_{24} + 197904q_{25} + 197904q_{26} + 197936q_{27} + 197880q_{28} + 197960q_{29} +$
$197848q_{30} + 197880q_{31} + 197832q_{32} + 197920q_{33} + 197856q_{34} + 197856q_{35} + 197888q_{36} + 197936q_{37} +$
$197888q_{38} + 197864q_{39} + 197848q_{40} + 197832q_{41} + 197880q_{42} + 197856q_{43} + 197888q_{44} + 197888q_{45} +$
$197888q_{46} + 197872q_{47} + 197952q_{48} + 197816q_{49} + 197848q_{50} + 197832q_{51} + 197824q_{52} + 197888q_{53} +$
$197888q_{54} + 197840q_{55} + 197904q_{56} + 197872q_{57} + 197864q_{58} + 197912q_{59} + 197864q_{60} + 197856q_{61} +$
$197824q_{62} + 197904q_{63} + 197840q_{64} + 197816q_{65} + 198008q_{66} + 197856q_{67} + 197856q_{68} + 197856q_{69} +$
$197824q_{70} + 197936q_{71} + 197824q_{72} + 197832q_{73} + 197824q_{74} + 197888q_{75} + 197888q_{76} + 197856q_{77} +$
$197808q_{78} + 197984q_{79} + 197856q_{80} + 197856q_{81} + 197856q_{82} + 197808q_{83} + 197904q_{84} + 197840q_{85} +$
$197840q_{86} + 197968q_{87} + 198128q_{88} + 197936q_{89} + 197872q_{90} + 197904q_{91} + 197968q_{8}3 + 197840q_{93} +$
$197936q_{94} + 197968q_{95} + 197840q_{96} + 197840q_{97} + 197936q_{98} + 197968q_{99} + 197936q_{100} + 197872q_{101} +$
$197936q_{102} + 198000q_{103} + 197968q_{104} + 197808q_{105} + 198256q_{106} + 197872q_{107} + 65936q_0q_1 + 65936q_0q_2 +$
$65936q_0q_3 + 65936q_1q_2 + 65936q_0q_4 + 65936q_1q_3 + 65936q_0q_5 + 65936q_1q_4 + 65936q_2q_3 + 65936q_0q_6 +$
$65936q_1q_5 + 65936q_2q_4 + 24q_0q_7 + 65936q_1q_6 + 65936q_2q_5 + 65936q_3q_4 + 65936q_0q_8 + 56q_1q_7 + 65936q_2q_6 +$
$65936q_3q_5 + 65936q_0q_9 + 65936q_1q_8 + 24q_2q_7 + 65936q_3q_6 + 65936q_4q_5 + 65936q_0q_{10} + 65936q_1q_9 +$
$65936q_2q_8 + 40q_3q_7 + 65936q_4q_6 + 48q_0q_{11} + 65936q_1q_{10} + 65936q_2q_9 + 65936q_3q_8 + 65936q_4q_7 + 65936q_5q_6 +$
$65936q_0q_{12} + 112q_1q_{11} + 65936q_2q_{10} + 65936q_3q_9 + 65936q_4q_8 + 65936q_5q_7 + 65936q_0q_{13} + 65936q_1q_{12} +$
$48q_2q_{11} + 65936q_3q_{10} + 65936q_4q_9 + 65936q_5q_8 + 65936q_6q_7 + 65936q_0q_{14} + 65936q_1q_{13} + 65936q_2q_{12} +$
$16q_3q_{11} + 16q_4q_{10} + 65936q_5q_9 + 65936q_6q_8 + 80q_0q_{15} + 65936q_1q_{14} + 65936q_2q_{13} + 65936q_3q_{12} + 48q_4q_{11} +$
$65936q_5q_{10} + 65936q_6q_9 + 65936q_7q_8 - 131872q_0q_{16} + 144q_1q_{15} + 65936q_2q_{14} + 65936q_3q_{13} + 65936q_4q_{12} +$
$48q_5q_{11} + 65936q_6q_{10} + 65936q_7q_9 - 131872q_0q_{17} - 131872q_1q_{16} + 80q_2q_{15} + 65936q_3q_{14} + 65936q_4q_{13} +$
$65936q_5q_{12} + 16q_6q_{11} + 65936q_7q_{10} + 65936q_8q_9 - 131872q_0q_{18} - 64q_2q_{16} + 80q_3q_{15} + 64q_4q_{14} + 65936q_5q_{13} +$
$65936q_6q_{12} + 48q_7q_{11} + 65936q_8q_{10} - 131872q_0q_{19} - 131872q_2q_{17} - 96q_3q_{16} + 16q_4q_{15} + 65936q_5q_{14} +$
$65936q_6q_{13} + 65936q_7q_{12} + 65936q_8q_{11} + 65936q_9q_{10} - 131872q_0q_{20} - 32q_3q_{17} + 112q_5q_{15} + 65936q_6q_{14} +$
$65936q_7q_{13} + 65936q_8q_{12} + 65936q_9q_{11} - 131872q_0q_{21} - 131872q_3q_{18} - 32q_4q_{17} - 32q_5q_{16} + 48q_6q_{15} +$
$65936q_7q_{14} + 65936q_8q_{13} + 65936q_9q_{12} + 65936q_{10}q_{11} - 131872q_0q_{22} - 16q_4q_{18} - 32q_6q_{16} + 112q_7q_{15} +$
$64q_8q_{14} + 65936q_9q_{13} + 65936q_{10}q_{12} - 131872q_0q_{23} - 131872q_4q_{19} - 32q_5q_{18} - 32q_6q_{17} - 32q_7q_{16} +$
$160q_8q_{15} + 65936q_9q_{14} + 65936q_{10}q_{13} + 65936q_{11}q_{12} - 131872q_0q_{24} - 32q_5q_{19} + 32q_6q_{18} + 96q_9q_{15} +$
$65936q_{10}q_{14} + 65936q_{11}q_{13} - 131872q_0q_{25} - 131872q_5q_{20} - 32q_6q_{19} - 16q_7q_{18} - 64q_8q_{17} - 64q_9q_{16} +$
$96q_{10}q_{15} + 64q_{11}q_{14} + 65936q_{12}q_{13} - 131872q_0q_{26} - 32q_6q_{20} - 16q_7q_{19} - 32q_8q_{18} - 64q_{10}q_{16} + 160q_{11}q_{15} +$
$65936q_{12}q_{14} - 131872q_0q_{27} - 131872q_6q_{21} - 32q_7q_{20} + 65936q_{12}q_{15} + 65936q_{13}q_{14} - 32q_7q_{21} - 64q_{11}q_{17} +$
$65936q_{13}q_{15} - 131872q_1q_{28} - 32q_{11}q_{18} - 64q_{12}q_{17} - 64q_{13}q_{16} + 65936q_{14}q_{15} - 131872q_1q_{29} - 131872q_2q_{28} -$
$131872q_8q_{22} - 32q_{12}q_{18} - 64q_{14}q_{16} - 131872q_1q_{30} - 64q_3q_{28} - 64q_9q_{22} - 64q_{13}q_{18} - 64q_{14}q_{17} - 64q_{15}q_{16} -$
$131872q_1q_{31} - 131872q_3q_{29} - 131872q_9q_{23} - 64q_{10}q_{22} + 32q_{14}q_{18} - 32q_{15}q_{17} - 131872q_1q_{32} - 32q_4q_{29} -$
$32q_5q_{28} - 64q_{10}q_{23} - 32q_{11}q_{22} - 32q_{15}q_{18} - 131872q_1q_{33} - 131872q_4q_{30} - 32q_6q_{28} - 131872q_{10}q_{24} -$
$64q_{11}q_{23} - 131872q_1q_{34} - 32q_5q_{30} - 32q_6q_{29} - 32q_7q_{28} - 64q_{11}q_{24} - 131872q_1q_{35} - 131872q_5q_{31} - 32q_6q_{30} -$
$131872q_1q_{36} - 32q_6q_{31} - 32q_7q_{30} - 64q_8q_{29} - 64q_9q_{28} - 131872q_{12}q_{25} - 131872q_1q_{37} - 131872q_6q_{32} -$
$32q_7q_{31} - 64q_{10}q_{28} - 64q_{13}q_{25} - 131872q_1q_{38} - 32q_7q_{32} - 131872q_{13}q_{26} - 64q_{14}q_{25} - 64q_{11}q_{29} - 64q_{14}q_{26} -$
$32q_{15}q_{25} - 131872q_2q_{39} - 131872q_8q_{33} - 64q_{12}q_{29} - 64q_{13}q_{28} - 131872q_{14}q_{27} - 64q_{15}q_{26} - 131872q_2q_{40} -$
$131872q_3q_{39} - 64q_9q_{33} - 64q_{14}q_{28} - 64q_{15}q_{27} - 131872q_2q_{41} - 131872q_9q_{34} - 64q_{10}q_{33} - 64q_{14}q_{29} -$
$64q_{15}q_{28} - 131872q_2q_{42} - 131872q_4q_{40} - 32q_5q_{39} - 64q_{10}q_{34} - 64q_{11}q_{33} - 32q_{15}q_{29} - 131872q_2q_{43} -$
$32q_5q_{40} - 32q_6q_{39} - 131872q_{10}q_{35} - 64q_{11}q_{34} - 131872q_2q_{44} - 131872q_5q_{41} - 32q_6q_{40} - 32q_7q_{39} -$
$64q_{11}q_{35} - 131872q_2q_{45} - 32q_6q_{41} - 131872q_2q_{46} - 131872q_6q_{42} - 32q_7q_{41} - 64q_9q_{39} - 131872q_{12}q_{36} -$
$131872q_2q_{47} - 32q_7q_{42} - 64q_{10}q_{39} - 64q_{13}q_{36} - 131872q_2q_{48} - 131872q_{13}q_{37} - 64q_{14}q_{36} - 131872q_8q_{43} -$

$64q_{14}q_{37} - 64q_{15}q_{36} - 131872q_{3}q_{49} - 64q_{9}q_{43} - 64q_{13}q_{39} - 131872q_{14}q_{38} - 64q_{15}q_{37} - 131872q_{3}q_{50} -$
$131872q_{4}q_{49} - 131872q_{9}q_{44} - 64q_{10}q_{43} - 64q_{14}q_{39} - 64q_{15}q_{38} - 131872q_{3}q_{51} - 32q_{5}q_{49} - 64q_{10}q_{44} -$
$64q_{15}q_{39} - 131872q_{3}q_{52} - 131872q_{5}q_{50} - 131872q_{10}q_{45} - 64q_{11}q_{44} + 64q_{16}q_{39} - 131872q_{3}q_{53} - 32q_{6}q_{50} -$
$16q_{7}q_{49} - 64q_{11}q_{45} - 131872q_{3}q_{54} - 131872q_{6}q_{51} - 131872q_{3}q_{55} - 32q_{7}q_{51} - 131872q_{12}q_{46} - 131872q_{3}q_{56} -$
$64q_{13}q_{46} - 131872q_{3}q_{57} - 131872q_{8}q_{52} - 131872q_{13}q_{47} - 64q_{14}q_{46} - 64q_{9}q_{52} - 64q_{14}q_{47} - 131872q_{4}q_{58} -$
$131872q_{9}q_{53} - 131872q_{14}q_{48} - 64q_{15}q_{47} - 131872q_{4}q_{59} - 131872q_{5}q_{58} - 64q_{10}q_{53} - 32q_{11}q_{52} - 64q_{15}q_{48} -$
$131872q_{4}q_{60} - 131872q_{10}q_{54} - 131872q_{4}q_{61} - 131872q_{6}q_{59} - 96q_{7}q_{58} - 64q_{11}q_{54} - 131872q_{4}q_{62} + 32q_{7}q_{59} -$
$131872q_{4}q_{63} - 131872q_{7}q_{60} - 64q_{8}q_{59} - 64q_{9}q_{58} - 131872q_{12}q_{55} - 131872q_{4}q_{64} - 32q_{8}q_{60} - 64q_{10}q_{58} -$
$64q_{13}q_{55} - 131872q_{8}q_{61} - 131872q_{13}q_{56} - 131872q_{5}q_{65} - 64q_{11}q_{59} - 64q_{14}q_{56} - 32q_{15}q_{55} - 131872q_{5}q_{66} -$
$131872q_{6}q_{65} - 131872q_{9}q_{62} - 64q_{10}q_{61} - 32q_{11}q_{60} - 64q_{12}q_{59} - 64q_{13}q_{58} - 131872q_{14}q_{57} - 131872q_{5}q_{67} -$
$64q_{7}q_{65} - 32q_{11}q_{61} - 32q_{12}q_{60} - 64q_{14}q_{58} - 64q_{15}q_{57} - 131872q_{5}q_{68} - 131872q_{7}q_{66} - 64q_{11}q_{62} - 64q_{13}q_{60} -$
$64q_{14}q_{59} - 64q_{15}q_{58} - 131872q_{5}q_{69} - 64q_{8}q_{66} - 64q_{9}q_{65} + 32q_{14}q_{60} - 32q_{15}q_{59} + 32q_{16}q_{58} - 131872q_{5}q_{70} -$
$131872q_{8}q_{67} - 64q_{10}q_{65} - 131872q_{12}q_{63} - 32q_{15}q_{60} - 131872q_{5}q_{71} - 64q_{9}q_{67} + 32q_{17}q_{59} - 131872q_{5}q_{72} -$
$131872q_{9}q_{68} - 64q_{11}q_{66} - 131872q_{13}q_{64} - 64q_{14}q_{63} - 64q_{10}q_{68} - 64q_{11}q_{67} - 64q_{12}q_{66} - 64q_{13}q_{65} - 32q_{15}q_{63} +$
$32q_{18}q_{60} - 131872q_{6}q_{73} - 131872q_{10}q_{69} - 64q_{14}q_{65} - 64q_{15}q_{64} - 131872q_{6}q_{74} - 131872q_{7}q_{73} - 64q_{11}q_{69} -$
$64q_{14}q_{66} - 64q_{15}q_{65} - 131872q_{6}q_{75} - 32q_{15}q_{66} + 32q_{16}q_{65} - 131872q_{6}q_{76} - 131872q_{8}q_{74} - 64q_{9}q_{73} -$
$131872q_{12}q_{70} - 131872q_{6}q_{77} - 64q_{9}q_{74} - 64q_{10}q_{73} - 64q_{13}q_{70} + 32q_{17}q_{66} - 131872q_{6}q_{78} - 131872q_{9}q_{75} -$
$64q_{10}q_{74} - 131872q_{13}q_{71} - 131872q_{6}q_{79} - 64q_{10}q_{75} + 64q_{11}q_{74} - 64q_{14}q_{71} - 64q_{15}q_{70} - 131872q_{10}q_{76} -$
$64q_{11}q_{75} - 64q_{13}q_{73} - 131872q_{14}q_{72} + 32q_{28}q_{58} - 131872q_{7}q_{80} - 64q_{11}q_{76} - 64q_{14}q_{73} - 64q_{15}q_{72} -$
$131872q_{7}q_{81} - 131872q_{8}q_{80} - 64q_{15}q_{73} + 32q_{29}q_{59} - 131872q_{7}q_{82} - 64q_{9}q_{80} - 131872q_{12}q_{77} + 32q_{16}q_{73} -$
$131872q_{7}q_{83} - 131872q_{9}q_{81} - 64q_{13}q_{77} - 131872q_{7}q_{84} - 64q_{10}q_{81} - 32q_{11}q_{80} - 131872q_{13}q_{78} - 64q_{14}q_{77} -$
$131872q_{7}q_{85} - 131872q_{10}q_{82} - 64q_{14}q_{78} + 64q_{15}q_{77} - 64q_{11}q_{82} - 131872q_{14}q_{79} - 64q_{15}q_{78} + 32q_{28}q_{65} -$
$131872q_{8}q_{86} - 64q_{15}q_{79} - 131872q_{8}q_{87} - 131872q_{9}q_{86} - 131872q_{12}q_{83} + 32q_{29}q_{66} - 131872q_{8}q_{88} -$
$64q_{13}q_{83} - 131872q_{8}q_{89} - 131872q_{10}q_{87} - 128q_{11}q_{86} - 131872q_{13}q_{84} + 32q_{39}q_{58} - 131872q_{8}q_{90} - 128q_{11}q_{87} -$
$64q_{14}q_{84} - 32q_{15}q_{83} - 131872q_{11}q_{88} - 128q_{12}q_{87} - 128q_{13}q_{86} - 131872q_{14}q_{85} - 131872q_{9}q_{91} - 64q_{12}q_{88} -$
$128q_{14}q_{86} - 64q_{15}q_{85} - 131872q_{9}q_{8}3 - 131872q_{10}q_{91} - 131872q_{12}q_{89} - 128q_{13}q_{88} - 128q_{14}q_{87} - 128q_{15}q_{86} +$
$32q_{28}q_{73} - 131872q_{9}q_{93} + 64q_{14}q_{88} - 64q_{15}q_{87} + 64q_{16}q_{86} - 131872q_{9}q_{94} - 131872q_{11}q_{8}3 - 131872q_{13}q_{90} -$
$128q_{14}q_{89} - 64q_{15}q_{88} - 131872q_{9}q_{95} - 128q_{12}q_{8}3 - 128q_{13}q_{91} - 64q_{15}q_{89} + 64q_{17}q_{87} + 32q_{39}q_{65} -$
$131872q_{12}q_{93} - 128q_{14}q_{91} - 128q_{15}q_{90} - 131872q_{10}q_{96} - 128q_{13}q_{93} - 128q_{14}q_{8}3 - 128q_{15}q_{91} + 64q_{18}q_{88} -$
$131872q_{10}q_{97} - 131872q_{11}q_{96} - 131872q_{13}q_{94} - 64q_{15}q_{8}3 + 64q_{16}q_{91} - 131872q_{10}q_{98} - 128q_{14}q_{94} -$
$131872q_{10}q_{99} - 131872q_{12}q_{97} - 128q_{13}q_{96} - 131872q_{14}q_{95} + 64q_{17}q_{8}3 - 128q_{13}q_{97} - 128q_{14}q_{96} - 128q_{15}q_{95} -$
$131872q_{11}q_{100} - 131872q_{13}q_{98} - 128q_{15}q_{96} - 131872q_{11}q_{101} - 131872q_{12}q_{100} - 128q_{14}q_{98} + 64q_{16}q_{96} +$
$32q_{39}q_{73} - 131872q_{14}q_{99} - 131872q_{12}q_{102} - 131872q_{13}q_{101} - 128q_{14}q_{100} - 128q_{15}q_{99} + 64q_{28}q_{86} -$
$131872q_{12}q_{103} - 131872q_{13}q_{102} - 64q_{15}q_{100} - 131872q_{12}q_{104} - 128q_{15}q_{101} + 64q_{29}q_{87} - 131872q_{14}q_{103} -$
$384q_{15}q_{102} - 131872q_{13}q_{105} + 64q_{16}q_{102} - 131872q_{13}q_{106} - 131872q_{14}q_{105} - 131872q_{15}q_{104} + 64q_{28}q_{91} -$
$256q_{15}q_{105} + 64q_{17}q_{103} - 131872q_{14}q_{107} - 131872q_{15}q_{106} + 64q_{16}q_{105} + 64q_{29}q_{8}3 - 131872q_{15}q_{107} +$
$64q_{18}q_{104} + 64q_{16}q_{107} + 64q_{17}q_{106} + 64q_{28}q_{96} + 64q_{39}q_{86} + 64q_{28}q_{102} + 64q_{39}q_{91} + 64q_{58}q_{73} + 64q_{29}q_{103} +$
$64q_{28}q_{105} + 64q_{28}q_{107} + 64q_{29}q_{106} + 64q_{39}q_{96} + 64q_{39}q_{102} + 64q_{39}q_{105} + 64q_{58}q_{86} + 64q_{39}q_{107} + 64q_{59}q_{87} +$
$64q_{60}q_{88} + 64q_{58}q_{91} + 64q_{59}q_{8}3 + 64q_{65}q_{86} + 64q_{66}q_{87} + 64q_{58}q_{96} + 64q_{65}q_{91} + 64q_{66}q_{8}3 + 64q_{73}q_{86} +$
$64q_{58}q_{102} + 64q_{65}q_{96} + 64q_{59}q_{103} + 64q_{58}q_{105} + 64q_{60}q_{104} + 64q_{73}q_{91} + 64q_{58}q_{107} + 64q_{59}q_{106} + 64q_{65}q_{102} +$
$64q_{66}q_{103} + 64q_{73}q_{96} + 64q_{65}q_{105} + 64q_{65}q_{107} + 64q_{66}q_{106} + 64q_{73}q_{102} + 64q_{73}q_{105} + 64q_{73}q_{107} + 256q_{86}q_{96} +$
$128q_{86}q_{102} + 128q_{87}q_{103} + 128q_{86}q_{105} + 128q_{88}q_{104} + 128q_{86}q_{107} + 128q_{87}q_{106} + 128q_{91}q_{102} + 128q_{8}3q_{103} +$
$128q_{91}q_{105} + 128q_{91}q_{107} + 128q_{8}3q_{106} + 128q_{96}q_{102} + 128q_{96}q_{105} + 128q_{96}q_{107} + 256q_{102}q_{107} + 472$

0.1 A complete expression of $E_2(s)$ in 92-order H matrix using extended-Turyn based method

$E_2(s) = 197860s_0 + 197832s_1 + 197872s_2 + 197880s_3 + 181374s_4 + 214362s_5 + 214402s_6 + 148454s_7 +$
$214436s_8 + 231000s_9 + 214592s_{10} + 82772s_{11} + 247556s_{12} + 247772s_{13} + 198456s_{14} + 50116s_{15} - 33004s_{16} -$

$33028s_{17} - 33012s_{18} - 32976s_{19} - 32972s_{20} - 32988s_{21} - 32952s_{22} - 32976s_{23} - 32992s_{24} - 32976s_{25} -$
$32984s_{26} - 33016s_{27} - 33004s_{28} - 33036s_{29} - 32964s_{30} - 32988s_{31} - 32972s_{32} - 32976s_{33} - 32960s_{34} -$
$32976s_{35} - 32960s_{36} - 33000s_{37} - 32992s_{38} - 33028s_{39} - 32972s_{40} - 32964s_{41} - 32996s_{42} - 32960s_{43} -$
$32976s_{44} - 32992s_{45} - 32976s_{46} - 32968s_{47} - 33024s_{48} - 32960s_{49} - 32980s_{50} - 32972s_{51} - 32952s_{52} -$
$32992s_{53} - 32992s_{54} - 32960s_{55} - 33000s_{56} - 32984s_{57} - 33028s_{58} - 33036s_{59} - 32996s_{60} - 32968s_{61} -$
$32960s_{62} - 32992s_{63} - 32968s_{64} - 32996s_{65} - 33076s_{66} - 32960s_{67} - 32976s_{68} - 32976s_{69} - 32944s_{70} -$
$33016s_{71} - 32960s_{72} - 33036s_{73} - 32960s_{74} - 32976s_{75} - 32992s_{76} - 32976s_{77} - 32936s_{78} - 33040s_{79} -$
$32968s_{80} - 32976s_{81} - 32976s_{82} - 32944s_{83} - 33000s_{84} - 32968s_{85} - 33112s_{86} - 33064s_{87} - 33144s_{88} -$
$32984s_{89} - 32968s_{90} - 33112s_{91} - 33096s_{92} - 32952s_{93} - 33000s_{94} - 33016s_{95} - 33144s_{96} - 32952s_{97} -$
$33000s_{98} - 33016s_{99} - 32984s_{100} - 32968s_{101} - 33192s_{102} - 33192s_{103} - 33112s_{104} - 33096s_{105} - 33320s_{106} -$
$33256s_{107} + 16484s_0s_1 + 16484s_0s_2 + 16484s_0s_3 + 16484s_1s_2 + 16484s_0s_4 + 16484s_1s_3 + 16484s_0s_5 + 16484s_1s_4 +$
$16484s_2s_3 + 16484s_0s_6 + 16484s_1s_5 + 16484s_2s_4 + 6s_0s_7 + 16484s_1s_6 + 16484s_2s_5 + 16484s_3s_4 + 16484s_0s_8 +$
$14s_1s_7 + 16484s_2s_6 + 16484s_3s_5 + 16484s_0s_9 + 16484s_1s_8 + 6s_2s_7 + 16484s_3s_6 + 16484s_4s_5 + 16484s_0s_{10} +$
$16484s_1s_9 + 16484s_2s_8 + 10s_3s_7 + 16484s_4s_6 + 12s_0s_{11} + 16484s_1s_{10} + 16484s_2s_9 + 16484s_3s_8 + 16484s_4s_7 +$
$16484s_5s_6 + 16484s_0s_{12} + 28s_1s_{11} + 16484s_2s_{10} + 16484s_3s_9 + 16484s_4s_8 + 16484s_5s_7 + 16484s_0s_{13} + 16484s_1s_{12} +$
$12s_2s_{11} + 16484s_3s_{10} + 16484s_4s_9 + 16484s_5s_8 + 16484s_6s_7 + 16484s_0s_{14} + 16484s_1s_{13} + 16484s_2s_{12} + 4s_3s_{11} +$
$4s_4s_{10} + 16484s_5s_9 + 16484s_6s_8 + 20s_0s_{15} + 16484s_1s_{14} + 16484s_2s_{13} + 16484s_3s_{12} + 12s_4s_{11} + 16484s_5s_{10} +$
$16484s_6s_9 + 16484s_7s_8 - 32968s_0s_{16} + 36s_1s_{15} + 16484s_2s_{14} + 16484s_3s_{13} + 16484s_4s_{12} + 12s_5s_{11} + 16484s_6s_{10} +$
$16484s_7s_9 - 32968s_0s_{17} - 32968s_1s_{16} + 20s_2s_{15} + 16484s_3s_{14} + 16484s_4s_{13} + 16484s_5s_{12} + 4s_6s_{11} + 16484s_7s_{10} +$
$16484s_8s_9 - 32968s_0s_{18} - 16s_2s_{16} + 20s_3s_{15} + 16s_4s_{14} + 16484s_5s_{13} + 16484s_6s_{12} + 12s_7s_{11} + 16484s_8s_{10} -$
$32968s_0s_{19} - 32968s_2s_{17} - 24s_3s_{16} + 4s_4s_{15} + 16484s_5s_{14} + 16484s_6s_{13} + 16484s_7s_{12} + 16484s_8s_{11} + 16484s_9s_{10} -$
$32968s_0s_{20} - 8s_3s_{17} + 28s_5s_{15} + 16484s_6s_{14} + 16484s_7s_{13} + 16484s_8s_{12} + 16484s_9s_{11} - 32968s_0s_{21} - 32968s_3s_{18} -$
$8s_4s_{17} - 8s_5s_{16} + 12s_6s_{15} + 16484s_7s_{14} + 16484s_8s_{13} + 16484s_9s_{12} + 16484s_{10}s_{11} - 32968s_0s_{22} - 4s_4s_{18} -$
$8s_6s_{16} + 28s_7s_{15} + 16s_8s_{14} + 16484s_9s_{13} + 16484s_{10}s_{12} - 32968s_0s_{23} - 32968s_4s_{19} - 8s_5s_{18} - 8s_6s_{17} - 8s_7s_{16} +$
$40s_8s_{15} + 16484s_9s_{14} + 16484s_{10}s_{13} + 16484s_{11}s_{12} - 32968s_0s_{24} - 8s_5s_{19} + 8s_6s_{18} + 24s_9s_{15} + 16484s_{10}s_{14} +$
$16484s_{11}s_{13} - 32968s_0s_{25} - 32968s_5s_{20} - 8s_6s_{19} - 4s_7s_{18} - 16s_8s_{17} - 16s_9s_{16} + 24s_{10}s_{15} + 16s_{11}s_{14} + 16484s_{12}s_{13} -$
$32968s_0s_{26} - 8s_6s_{20} - 4s_7s_{19} - 8s_8s_{18} - 16s_{10}s_{16} + 40s_{11}s_{15} + 16484s_{12}s_{14} - 32968s_0s_{27} - 32968s_6s_{21} - 8s_7s_{20} +$
$16484s_{12}s_{15} + 16484s_{13}s_{14} - 8s_7s_{21} - 16s_{11}s_{17} + 16484s_{13}s_{15} - 32968s_1s_{28} - 8s_{11}s_{18} - 16s_{12}s_{17} - 16s_{13}s_{16} +$
$16484s_{14}s_{15} - 32968s_1s_{29} - 32968s_2s_{28} - 32968s_8s_{22} - 8s_{12}s_{18} - 16s_{14}s_{16} - 32968s_1s_{30} - 16s_3s_{28} - 16s_9s_{22} -$
$16s_{13}s_{18} - 16s_{14}s_{17} - 16s_{15}s_{16} - 32968s_1s_{31} - 32968s_3s_{29} - 32968s_9s_{23} - 16s_{10}s_{22} + 8s_{14}s_{18} - 8s_{15}s_{17} -$
$32968s_1s_{32} - 8s_4s_{29} - 8s_5s_{28} - 16s_{10}s_{23} - 8s_{11}s_{22} - 8s_{15}s_{18} - 32968s_1s_{33} - 32968s_4s_{30} - 8s_6s_{28} - 32968s_{10}s_{24} -$
$16s_{11}s_{23} - 32968s_1s_{34} - 8s_5s_{30} - 8s_6s_{29} - 8s_7s_{28} - 16s_{11}s_{24} - 32968s_1s_{35} - 32968s_5s_{31} - 8s_6s_{30} - 32968s_1s_{36} -$
$8s_6s_{31} - 8s_7s_{30} - 16s_8s_{29} - 16s_9s_{28} - 32968s_{12}s_{25} - 32968s_1s_{37} - 32968s_6s_{32} - 8s_7s_{31} - 16s_{10}s_{28} - 16s_{13}s_{25} -$
$32968s_1s_{38} - 8s_7s_{32} - 32968s_{13}s_{26} - 16s_{14}s_{25} - 16s_{11}s_{29} - 16s_{14}s_{26} - 8s_{15}s_{25} - 32968s_2s_{39} - 32968s_8s_{33} -$
$16s_{12}s_{29} - 16s_{13}s_{28} - 32968s_{14}s_{27} - 16s_{15}s_{26} - 32968s_2s_{40} - 32968s_3s_{39} - 16s_9s_{33} - 16s_{14}s_{28} - 16s_{15}s_{27} -$
$32968s_2s_{41} - 32968s_9s_{34} - 16s_{10}s_{33} - 16s_{14}s_{29} - 16s_{15}s_{28} - 32968s_2s_{42} - 32968s_4s_{40} - 8s_5s_{39} - 16s_{10}s_{34} -$
$16s_{11}s_{33} - 8s_{15}s_{29} - 32968s_2s_{43} - 8s_5s_{40} - 8s_6s_{39} - 32968s_{10}s_{35} - 16s_{11}s_{34} - 32968s_2s_{44} - 32968s_5s_{41} -$
$8s_6s_{40} - 8s_7s_{39} - 16s_{11}s_{35} - 32968s_2s_{45} - 8s_6s_{41} - 32968s_2s_{46} - 32968s_6s_{42} - 8s_7s_{41} - 16s_9s_{39} - 32968s_{12}s_{36} -$
$32968s_2s_{47} - 8s_7s_{42} - 16s_{10}s_{39} - 16s_{13}s_{36} - 32968s_2s_{48} - 32968s_{13}s_{37} - 16s_{14}s_{36} - 32968s_8s_{43} - 16s_{14}s_{37} -$
$16s_{15}s_{36} - 32968s_3s_{49} - 16s_9s_{43} - 16s_{13}s_{39} - 32968s_{14}s_{38} - 16s_{15}s_{37} - 32968s_3s_{50} - 32968s_4s_{49} - 32968s_9s_{44} -$
$16s_{10}s_{43} - 16s_{14}s_{39} - 16s_{15}s_{38} - 32968s_3s_{51} - 8s_5s_{49} - 16s_{10}s_{44} - 16s_{15}s_{39} - 32968s_3s_{52} - 32968s_5s_{50} -$
$32968s_{10}s_{45} - 16s_{11}s_{44} + 16s_{16}s_{39} - 32968s_3s_{53} - 8s_6s_{50} - 4s_7s_{49} - 16s_{11}s_{45} - 32968s_3s_{54} - 32968s_6s_{51} -$
$32968s_3s_{55} - 8s_7s_{51} - 32968s_{12}s_{46} - 32968s_3s_{56} - 16s_{13}s_{46} - 32968s_3s_{57} - 32968s_8s_{52} - 32968s_{13}s_{47} -$
$16s_{14}s_{46} - 16s_9s_{52} - 16s_{14}s_{47} - 32968s_4s_{58} - 32968s_9s_{53} - 32968s_{14}s_{48} - 16s_{15}s_{47} - 32968s_4s_{59} - 32968s_5s_{58} -$
$16s_{10}s_{53} - 8s_{11}s_{52} - 16s_{15}s_{48} - 32968s_4s_{60} - 32968s_{10}s_{54} - 32968s_4s_{61} - 32968s_6s_{59} - 24s_7s_{58} - 16s_{11}s_{54} -$
$32968s_4s_{62} + 8s_7s_{59} - 32968s_4s_{63} - 32968s_7s_{60} - 16s_8s_{59} - 16s_9s_{58} - 32968s_{12}s_{55} - 32968s_4s_{64} - 8s_8s_{60} -$
$16s_{10}s_{58} - 16s_{13}s_{55} - 32968s_8s_{61} - 32968s_{13}s_{56} - 32968s_5s_{65} - 16s_{11}s_{59} - 16s_{14}s_{56} - 8s_{15}s_{55} - 32968s_5s_{66} -$
$32968s_6s_{65} - 32968s_9s_{62} - 16s_{10}s_{61} - 8s_{11}s_{60} - 16s_{12}s_{59} - 16s_{13}s_{58} - 32968s_{14}s_{57} - 32968s_5s_{67} - 16s_7s_{65} -$

$$8s_{11}s_{61} - 8s_{12}s_{60} - 16s_{14}s_{58} - 16s_{15}s_{57} - 32968s_{5}s_{68} - 32968s_{7}s_{66} - 16s_{11}s_{62} - 16s_{13}s_{60} - 16s_{14}s_{59} - 16s_{15}s_{58} - 32968s_{5}s_{69} - 16s_{8}s_{66} - 16s_{9}s_{65} + 8s_{14}s_{60} - 8s_{15}s_{59} + 8s_{16}s_{58} - 32968s_{5}s_{70} - 32968s_{8}s_{67} - 16s_{10}s_{65} - 32968s_{12}s_{63} - 8s_{15}s_{60} - 32968s_{5}s_{71} - 16s_{9}s_{67} + 8s_{17}s_{59} - 32968s_{5}s_{72} - 32968s_{9}s_{68} - 16s_{11}s_{66} - 32968s_{13}s_{64} - 16s_{14}s_{63} - 16s_{10}s_{68} - 16s_{11}s_{67} - 16s_{12}s_{66} - 16s_{13}s_{65} - 8s_{15}s_{63} + 8s_{18}s_{60} - 32968s_{6}s_{73} - 32968s_{10}s_{69} - 16s_{14}s_{65} - 16s_{15}s_{64} - 32968s_{6}s_{74} - 32968s_{7}s_{73} - 16s_{11}s_{69} - 16s_{14}s_{66} - 16s_{15}s_{65} - 32968s_{6}s_{75} - 8s_{15}s_{66} + 8s_{16}s_{65} - 32968s_{6}s_{76} - 32968s_{8}s_{74} - 16s_{9}s_{73} - 32968s_{12}s_{70} - 32968s_{6}s_{77} - 16s_{9}s_{74} - 16s_{10}s_{73} - 16s_{13}s_{70} + 8s_{17}s_{66} - 32968s_{6}s_{78} - 32968s_{9}s_{75} - 16s_{10}s_{74} - 32968s_{13}s_{71} - 32968s_{6}s_{79} - 16s_{10}s_{75} + 16s_{11}s_{74} - 16s_{14}s_{71} - 16s_{15}s_{70} - 32968s_{10}s_{76} - 16s_{11}s_{75} - 16s_{13}s_{73} - 32968s_{14}s_{72} + 8s_{28}s_{58} - 32968s_{7}s_{80} - 16s_{11}s_{76} - 16s_{14}s_{73} - 16s_{15}s_{72} - 32968s_{7}s_{81} - 32968s_{8}s_{80} - 16s_{15}s_{73} + 8s_{29}s_{59} - 32968s_{7}s_{82} - 16s_{9}s_{80} - 32968s_{12}s_{77} + 8s_{16}s_{73} - 32968s_{7}s_{83} - 32968s_{9}s_{81} - 16s_{13}s_{77} - 32968s_{7}s_{84} - 16s_{10}s_{81} - 8s_{11}s_{80} - 32968s_{13}s_{78} - 16s_{14}s_{77} - 32968s_{7}s_{85} - 32968s_{10}s_{82} - 16s_{14}s_{78} + 16s_{15}s_{77} - 16s_{11}s_{82} - 32968s_{14}s_{79} - 16s_{15}s_{78} + 8s_{28}s_{65} - 32968s_{8}s_{86} - 16s_{15}s_{79} - 32968s_{8}s_{87} - 32968s_{9}s_{86} - 32968s_{12}s_{83} + 8s_{29}s_{66} - 32968s_{8}s_{88} - 16s_{13}s_{83} - 32968s_{8}s_{89} - 32968s_{10}s_{87} - 32s_{11}s_{86} - 32968s_{13}s_{84} + 8s_{39}s_{58} - 32968s_{8}s_{90} - 32s_{11}s_{87} - 16s_{14}s_{84} - 8s_{15}s_{83} - 32968s_{11}s_{88} - 32s_{12}s_{87} - 32s_{13}s_{86} - 32968s_{14}s_{85} - 32968s_{9}s_{91} - 16s_{12}s_{88} - 32s_{14}s_{86} - 16s_{15}s_{85} - 32968s_{9}s_{92} - 32968s_{10}s_{91} - 32968s_{12}s_{89} - 32s_{13}s_{88} - 32s_{14}s_{87} - 32s_{15}s_{86} + 8s_{28}s_{73} - 32968s_{9}s_{93} + 16s_{14}s_{88} - 16s_{15}s_{87} + 16s_{16}s_{86} - 32968s_{9}s_{94} - 32968s_{11}s_{92} - 32968s_{13}s_{90} - 32s_{14}s_{89} - 16s_{15}s_{88} - 32968s_{9}s_{95} - 32s_{12}s_{92} - 32s_{13}s_{91} - 16s_{15}s_{89} + 16s_{17}s_{87} + 8s_{39}s_{65} - 32968s_{12}s_{93} - 32s_{14}s_{91} - 32s_{15}s_{90} - 32968s_{10}s_{96} - 32s_{13}s_{93} - 32s_{14}s_{92} - 32s_{15}s_{91} + 16s_{18}s_{88} - 32968s_{10}s_{97} - 32968s_{11}s_{96} - 32968s_{13}s_{94} - 16s_{15}s_{92} + 16s_{16}s_{91} - 32968s_{10}s_{98} - 32s_{14}s_{94} - 32968s_{10}s_{99} - 32968s_{12}s_{97} - 32s_{13}s_{96} - 32968s_{14}s_{95} + 16s_{17}s_{92} - 32s_{13}s_{97} - 32s_{14}s_{96} - 32s_{15}s_{95} - 32968s_{11}s_{100} - 32968s_{13}s_{98} - 32s_{15}s_{96} - 32968s_{11}s_{101} - 32968s_{12}s_{100} - 32s_{14}s_{98} + 16s_{16}s_{96} + 8s_{39}s_{73} - 32968s_{14}s_{99} - 32968s_{12}s_{102} - 32968s_{13}s_{101} - 32s_{14}s_{100} - 32s_{15}s_{99} + 16s_{28}s_{86} - 32968s_{12}s_{103} - 32968s_{13}s_{102} - 16s_{15}s_{100} - 32968s_{12}s_{104} - 32s_{15}s_{101} + 16s_{29}s_{87} - 32968s_{14}s_{103} - 96s_{15}s_{102} - 32968s_{13}s_{105} + 16s_{16}s_{102} - 32968s_{13}s_{106} - 32968s_{14}s_{105} - 32968s_{15}s_{104} + 16s_{28}s_{91} - 64s_{15}s_{105} + 16s_{17}s_{103} - 32968s_{14}s_{107} - 32968s_{15}s_{106} + 16s_{16}s_{105} + 16s_{29}s_{92} - 32968s_{15}s_{107} + 16s_{18}s_{104} + 16s_{16}s_{107} + 16s_{17}s_{106} + 16s_{28}s_{96} + 16s_{39}s_{86} + 16s_{28}s_{102} + 16s_{39}s_{91} + 16s_{58}s_{73} + 16s_{29}s_{103} + 16s_{28}s_{105} + 16s_{28}s_{107} + 16s_{29}s_{106} + 16s_{39}s_{96} + 16s_{39}s_{102} + 16s_{39}s_{105} + 16s_{58}s_{86} + 16s_{39}s_{107} + 16s_{59}s_{87} + 16s_{60}s_{88} + 16s_{58}s_{91} + 16s_{59}s_{92} + 16s_{65}s_{86} + 16s_{66}s_{87} + 16s_{58}s_{96} + 16s_{65}s_{91} + 16s_{66}s_{92} + 16s_{73}s_{86} + 16s_{58}s_{102} + 16s_{65}s_{96} + 16s_{59}s_{103} + 16s_{58}s_{105} + 16s_{60}s_{104} + 16s_{73}s_{91} + 16s_{58}s_{107} + 16s_{59}s_{106} + 16s_{65}s_{102} + 16s_{66}s_{103} + 16s_{73}s_{96} + 16s_{65}s_{105} + 16s_{65}s_{107} + 16s_{66}s_{106} + 16s_{73}s_{102} + 16s_{73}s_{105} + 16s_{73}s_{107} + 64s_{86}s_{96} + 32s_{86}s_{102} + 32s_{87}s_{103} + 32s_{86}s_{105} + 32s_{88}s_{104} + 32s_{86}s_{107} + 32s_{87}s_{106} + 32s_{91}s_{102} + 32s_{92}s_{103} + 32s_{91}s_{105} + 32s_{91}s_{107} + 32s_{92}s_{106} + 32s_{96}s_{102} + 32s_{96}s_{105} + 32s_{96}s_{107} + 64s_{102}s_{107} + 4551232$$

0.2 A complete expression of $\hat{H}_2\left(\hat{\sigma}^z\right)$ in 92-order H matrix using extended-Turyn based method

$$\hat{H}_2\left(\hat{\sigma}^z\right) = 197860\hat{\sigma}^z_{0} + 197832\hat{\sigma}^z_{1} + 197872\hat{\sigma}^z_{2} + 197880\hat{\sigma}^z_{3} + 181374\hat{\sigma}^z_{4} + 214362\hat{\sigma}^z_{5} + 214402\hat{\sigma}^z_{6} + 148454\hat{\sigma}^z_{7} + 214436\hat{\sigma}^z_{8} + 231000\hat{\sigma}^z_{9} + 214592\hat{\sigma}^z_{10} + 82772\hat{\sigma}^z_{11} + 247556\hat{\sigma}^z_{12} + 247772\hat{\sigma}^z_{13} + 198456\hat{\sigma}^z_{14} + 50116\hat{\sigma}^z_{15} - 33004\hat{\sigma}^z_{16} - 33028\hat{\sigma}^z_{17} - 33012\hat{\sigma}^z_{18} - 32976\hat{\sigma}^z_{19} - 32972\hat{\sigma}^z_{20} - 32988\hat{\sigma}^z_{21} - 32952\hat{\sigma}^z_{22} - 32976\hat{\sigma}^z_{23} - 32992\hat{\sigma}^z_{24} - 32976\hat{\sigma}^z_{25} - 32984\hat{\sigma}^z_{26} - 33016\hat{\sigma}^z_{27} - 33004\hat{\sigma}^z_{28} - 33036\hat{\sigma}^z_{29} - 32964\hat{\sigma}^z_{30} - 32988\hat{\sigma}^z_{31} - 32972\hat{\sigma}^z_{32} - 32976\hat{\sigma}^z_{33} - 32960\hat{\sigma}^z_{34} - 32976\hat{\sigma}^z_{35} - 32960\hat{\sigma}^z_{36} - 33000\hat{\sigma}^z_{37} - 32992\hat{\sigma}^z_{38} - 33028\hat{\sigma}^z_{39} - 32972\hat{\sigma}^z_{40} - 32964\hat{\sigma}^z_{41} - 32996\hat{\sigma}^z_{42} - 32960\hat{\sigma}^z_{43} - 32976\hat{\sigma}^z_{44} - 32992\hat{\sigma}^z_{45} - 32976\hat{\sigma}^z_{46} - 32968\hat{\sigma}^z_{47} - 33024\hat{\sigma}^z_{48} - 32960\hat{\sigma}^z_{49} - 32980\hat{\sigma}^z_{50} - 32972\hat{\sigma}^z_{51} - 32952\hat{\sigma}^z_{52} - 32992\hat{\sigma}^z_{53} - 32992\hat{\sigma}^z_{54} - 32960\hat{\sigma}^z_{55} - 33000\hat{\sigma}^z_{56} - 32984\hat{\sigma}^z_{57} - 33028\hat{\sigma}^z_{58} - 33036\hat{\sigma}^z_{59} - 32996\hat{\sigma}^z_{60} - 32968\hat{\sigma}^z_{61} - 32960\hat{\sigma}^z_{62} - 32992\hat{\sigma}^z_{63} - 32968\hat{\sigma}^z_{64} - 32996\hat{\sigma}^z_{65} - 33076\hat{\sigma}^z_{66} - 32960\hat{\sigma}^z_{67} - 32976\hat{\sigma}^z_{68} - 32976\hat{\sigma}^z_{69} - 32944\hat{\sigma}^z_{70} - 33016\hat{\sigma}^z_{71} - 32960\hat{\sigma}^z_{72} - 33036\hat{\sigma}^z_{73} - 32960\hat{\sigma}^z_{74} - 32976\hat{\sigma}^z_{75} - 32992\hat{\sigma}^z_{76} - 32976\hat{\sigma}^z_{77} - 32936\hat{\sigma}^z_{78} - 33040\hat{\sigma}^z_{79} - 32968\hat{\sigma}^z_{80} - 32976\hat{\sigma}^z_{81} - 32976\hat{\sigma}^z_{82} - 32944\hat{\sigma}^z_{83} - 33000\hat{\sigma}^z_{84} - 32968\hat{\sigma}^z_{85} - 33112\hat{\sigma}^z_{86} - 33064\hat{\sigma}^z_{87} - 33144\hat{\sigma}^z_{88} - 32984\hat{\sigma}^z_{89} - 32968\hat{\sigma}^z_{90} - 33112\hat{\sigma}^z_{91} - 33096\hat{\sigma}^z_{92} - 32952\hat{\sigma}^z_{93} - 33000\hat{\sigma}^z_{94} - 33016\hat{\sigma}^z_{95} - 33144\hat{\sigma}^z_{96} - 32952\hat{\sigma}^z_{97} - 33000\hat{\sigma}^z_{98} - 33016\hat{\sigma}^z_{99} - 32984\hat{\sigma}^z_{100} - 32968\hat{\sigma}^z_{101} - 33192\hat{\sigma}^z_{102} - 33192\hat{\sigma}^z_{103} - 33112\hat{\sigma}^z_{104} - 33096\hat{\sigma}^z_{105} - 33320\hat{\sigma}^z_{106} - 33256\hat{\sigma}^z_{107} + 16484\hat{\sigma}^z_{0}\hat{\sigma}^z_{1} + 16484\hat{\sigma}^z_{0}\hat{\sigma}^z_{2} + 16484\hat{\sigma}^z_{0}\hat{\sigma}^z_{3} + 16484\hat{\sigma}^z_{1}\hat{\sigma}^z_{2} + 16484\hat{\sigma}^z_{0}\hat{\sigma}^z_{4} + 16484\hat{\sigma}^z_{1}\hat{\sigma}^z_{3} + 16484\hat{\sigma}^z_{0}\hat{\sigma}^z_{5} + 16484\hat{\sigma}^z_{1}\hat{\sigma}^z_{4} + 16484\hat{\sigma}^z_{2}\hat{\sigma}^z_{3} + 16484\hat{\sigma}^z_{0}\hat{\sigma}^z_{6} + 16484\hat{\sigma}^z_{1}\hat{\sigma}^z_{5} + 16484\hat{\sigma}^z_{2}\hat{\sigma}^z_{4} + 6\hat{\sigma}^z_{0}\hat{\sigma}^z_{7} + 16484\hat{\sigma}^z_{1}\hat{\sigma}^z_{6} + 16484\hat{\sigma}^z_{2}\hat{\sigma}^z_{5} + 16484\hat{\sigma}^z_{3}\hat{\sigma}^z_{4} + 16484\hat{\sigma}^z_{0}\hat{\sigma}^z_{8} + 14\hat{\sigma}^z_{1}\hat{\sigma}^z_{7} + 16484\hat{\sigma}^z_{2}\hat{\sigma}^z_{6} + 16484\hat{\sigma}^z_{3}\hat{\sigma}^z_{5} + 16484\hat{\sigma}^z_{0}\hat{\sigma}^z_{9} + 16484\hat{\sigma}^z_{1}\hat{\sigma}^z_{8} + 6\hat{\sigma}^z_{2}\hat{\sigma}^z_{7} + 16484\hat{\sigma}^z_{3}\hat{\sigma}^z_{6} + 16484\hat{\sigma}^z_{4}\hat{\sigma}^z_{5} + 16484\hat{\sigma}^z_{0}\hat{\sigma}^z_{10} + 16484\hat{\sigma}^z_{1}\hat{\sigma}^z_{9} + 16484\hat{\sigma}^z_{2}\hat{\sigma}^z_{8} + 10\hat{\sigma}^z_{3}\hat{\sigma}^z_{7} +$$

$16484\hat{\sigma}^z_{4}\hat{\sigma}^z_{6} + 12\hat{\sigma}^z_{0}\hat{\sigma}^z_{11} + 16484\hat{\sigma}^z_{1}\hat{\sigma}^z_{10} + 16484\hat{\sigma}^z_{2}\hat{\sigma}^z_{9} + 16484\hat{\sigma}^z_{3}\hat{\sigma}^z_{8} + 16484\hat{\sigma}^z_{4}\hat{\sigma}^z_{7} + 16484\hat{\sigma}^z_{5}\hat{\sigma}^z_{6} + 16484\hat{\sigma}^z_{0}\hat{\sigma}^z_{12} +$
$28\hat{\sigma}^z_{1}\hat{\sigma}^z_{11} + 16484\hat{\sigma}^z_{2}\hat{\sigma}^z_{10} + 16484\hat{\sigma}^z_{3}\hat{\sigma}^z_{9} + 16484\hat{\sigma}^z_{4}\hat{\sigma}^z_{8} + 16484\hat{\sigma}^z_{5}\hat{\sigma}^z_{7} + 16484\hat{\sigma}^z_{0}\hat{\sigma}^z_{13} + 16484\hat{\sigma}^z_{1}\hat{\sigma}^z_{12} + 12\hat{\sigma}^z_{2}\hat{\sigma}^z_{11} +$
$16484\hat{\sigma}^z_{3}\hat{\sigma}^z_{10} + 16484\hat{\sigma}^z_{4}\hat{\sigma}^z_{9} + 16484\hat{\sigma}^z_{5}\hat{\sigma}^z_{8} + 16484\hat{\sigma}^z_{6}\hat{\sigma}^z_{7} + 16484\hat{\sigma}^z_{0}\hat{\sigma}^z_{14} + 16484\hat{\sigma}^z_{1}\hat{\sigma}^z_{13} + 16484\hat{\sigma}^z_{2}\hat{\sigma}^z_{12} + 4\hat{\sigma}^z_{3}\hat{\sigma}^z_{11} +$
$4\hat{\sigma}^z_{4}\hat{\sigma}^z_{10} + 16484\hat{\sigma}^z_{5}\hat{\sigma}^z_{9} + 16484\hat{\sigma}^z_{6}\hat{\sigma}^z_{8} + 20\hat{\sigma}^z_{0}\hat{\sigma}^z_{15} + 16484\hat{\sigma}^z_{1}\hat{\sigma}^z_{14} + 16484\hat{\sigma}^z_{2}\hat{\sigma}^z_{13} + 16484\hat{\sigma}^z_{3}\hat{\sigma}^z_{12} + 12\hat{\sigma}^z_{4}\hat{\sigma}^z_{11} +$
$16484\hat{\sigma}^z_{5}\hat{\sigma}^z_{10} + 16484\hat{\sigma}^z_{6}\hat{\sigma}^z_{9} + 16484\hat{\sigma}^z_{7}\hat{\sigma}^z_{8} - 32968\hat{\sigma}^z_{0}\hat{\sigma}^z_{16} + 36\hat{\sigma}^z_{1}\hat{\sigma}^z_{15} + 16484\hat{\sigma}^z_{2}\hat{\sigma}^z_{14} + 16484\hat{\sigma}^z_{3}\hat{\sigma}^z_{13} + 16484\hat{\sigma}^z_{4}\hat{\sigma}^z_{12} +$
$12\hat{\sigma}^z_{5}\hat{\sigma}^z_{11} + 16484\hat{\sigma}^z_{6}\hat{\sigma}^z_{10} + 16484\hat{\sigma}^z_{7}\hat{\sigma}^z_{9} - 32968\hat{\sigma}^z_{0}\hat{\sigma}^z_{17} - 32968\hat{\sigma}^z_{1}\hat{\sigma}^z_{16} + 20\hat{\sigma}^z_{2}\hat{\sigma}^z_{15} + 16484\hat{\sigma}^z_{3}\hat{\sigma}^z_{14} + 16484\hat{\sigma}^z_{4}\hat{\sigma}^z_{13} +$
$16484\hat{\sigma}^z_{5}\hat{\sigma}^z_{12} + 4\hat{\sigma}^z_{6}\hat{\sigma}^z_{11} + 16484\hat{\sigma}^z_{7}\hat{\sigma}^z_{10} + 16484\hat{\sigma}^z_{8}\hat{\sigma}^z_{9} - 32968\hat{\sigma}^z_{0}\hat{\sigma}^z_{18} - 16\hat{\sigma}^z_{2}\hat{\sigma}^z_{16} + 20\hat{\sigma}^z_{3}\hat{\sigma}^z_{15} + 16\hat{\sigma}^z_{4}\hat{\sigma}^z_{14} + 16484\hat{\sigma}^z_{5}\hat{\sigma}^z_{13} +$
$16484\hat{\sigma}^z_{6}\hat{\sigma}^z_{12} + 12\hat{\sigma}^z_{7}\hat{\sigma}^z_{11} + 16484\hat{\sigma}^z_{8}\hat{\sigma}^z_{10} - 32968\hat{\sigma}^z_{0}\hat{\sigma}^z_{19} - 32968\hat{\sigma}^z_{2}\hat{\sigma}^z_{17} - 24\hat{\sigma}^z_{3}\hat{\sigma}^z_{16} + 4\hat{\sigma}^z_{4}\hat{\sigma}^z_{15} + 16484\hat{\sigma}^z_{5}\hat{\sigma}^z_{14} +$
$16484\hat{\sigma}^z_{6}\hat{\sigma}^z_{13} + 16484\hat{\sigma}^z_{7}\hat{\sigma}^z_{12} + 16484\hat{\sigma}^z_{8}\hat{\sigma}^z_{11} + 16484\hat{\sigma}^z_{9}\hat{\sigma}^z_{10} - 32968\hat{\sigma}^z_{0}\hat{\sigma}^z_{20} - 8\hat{\sigma}^z_{3}\hat{\sigma}^z_{17} + 28\hat{\sigma}^z_{5}\hat{\sigma}^z_{15} + 16484\hat{\sigma}^z_{6}\hat{\sigma}^z_{14} +$
$16484\hat{\sigma}^z_{7}\hat{\sigma}^z_{13} + 16484\hat{\sigma}^z_{8}\hat{\sigma}^z_{12} + 16484\hat{\sigma}^z_{9}\hat{\sigma}^z_{11} - 32968\hat{\sigma}^z_{0}\hat{\sigma}^z_{21} - 32968\hat{\sigma}^z_{3}\hat{\sigma}^z_{18} - 8\hat{\sigma}^z_{4}\hat{\sigma}^z_{17} - 8\hat{\sigma}^z_{5}\hat{\sigma}^z_{16} + 12\hat{\sigma}^z_{6}\hat{\sigma}^z_{15} +$
$16484\hat{\sigma}^z_{7}\hat{\sigma}^z_{14} + 16484\hat{\sigma}^z_{8}\hat{\sigma}^z_{13} + 16484\hat{\sigma}^z_{9}\hat{\sigma}^z_{12} + 16484\hat{\sigma}^z_{10}\hat{\sigma}^z_{11} - 32968\hat{\sigma}^z_{0}\hat{\sigma}^z_{22} - 4\hat{\sigma}^z_{4}\hat{\sigma}^z_{18} - 8\hat{\sigma}^z_{6}\hat{\sigma}^z_{16} + 28\hat{\sigma}^z_{7}\hat{\sigma}^z_{15} +$
$16\hat{\sigma}^z_{8}\hat{\sigma}^z_{14} + 16484\hat{\sigma}^z_{9}\hat{\sigma}^z_{13} + 16484\hat{\sigma}^z_{10}\hat{\sigma}^z_{12} - 32968\hat{\sigma}^z_{0}\hat{\sigma}^z_{23} - 32968\hat{\sigma}^z_{4}\hat{\sigma}^z_{19} - 8\hat{\sigma}^z_{5}\hat{\sigma}^z_{18} - 8\hat{\sigma}^z_{6}\hat{\sigma}^z_{17} - 8\hat{\sigma}^z_{7}\hat{\sigma}^z_{16} + 40\hat{\sigma}^z_{8}\hat{\sigma}^z_{15} +$
$16484\hat{\sigma}^z_{9}\hat{\sigma}^z_{14} + 16484\hat{\sigma}^z_{10}\hat{\sigma}^z_{13} + 16484\hat{\sigma}^z_{11}\hat{\sigma}^z_{12} - 32968\hat{\sigma}^z_{0}\hat{\sigma}^z_{24} - 8\hat{\sigma}^z_{5}\hat{\sigma}^z_{19} + 8\hat{\sigma}^z_{6}\hat{\sigma}^z_{18} + 24\hat{\sigma}^z_{9}\hat{\sigma}^z_{15} + 16484\hat{\sigma}^z_{10}\hat{\sigma}^z_{14} +$
$16484\hat{\sigma}^z_{11}\hat{\sigma}^z_{13} - 32968\hat{\sigma}^z_{0}\hat{\sigma}^z_{25} - 32968\hat{\sigma}^z_{5}\hat{\sigma}^z_{20} - 8\hat{\sigma}^z_{6}\hat{\sigma}^z_{19} - 4\hat{\sigma}^z_{7}\hat{\sigma}^z_{18} - 16\hat{\sigma}^z_{8}\hat{\sigma}^z_{17} - 16\hat{\sigma}^z_{9}\hat{\sigma}^z_{16} + 24\hat{\sigma}^z_{10}\hat{\sigma}^z_{15} + 16\hat{\sigma}^z_{11}\hat{\sigma}^z_{14} +$
$16484\hat{\sigma}^z_{12}\hat{\sigma}^z_{13} - 32968\hat{\sigma}^z_{0}\hat{\sigma}^z_{26} - 8\hat{\sigma}^z_{6}\hat{\sigma}^z_{20} - 4\hat{\sigma}^z_{7}\hat{\sigma}^z_{19} - 8\hat{\sigma}^z_{8}\hat{\sigma}^z_{18} - 16\hat{\sigma}^z_{10}\hat{\sigma}^z_{16} + 40\hat{\sigma}^z_{11}\hat{\sigma}^z_{15} + 16484\hat{\sigma}^z_{12}\hat{\sigma}^z_{14} - 32968\hat{\sigma}^z_{0}\hat{\sigma}^z_{27} -$
$32968\hat{\sigma}^z_{6}\hat{\sigma}^z_{21} - 8\hat{\sigma}^z_{7}\hat{\sigma}^z_{20} + 16484\hat{\sigma}^z_{12}\hat{\sigma}^z_{15} + 16484\hat{\sigma}^z_{13}\hat{\sigma}^z_{14} - 8\hat{\sigma}^z_{7}\hat{\sigma}^z_{21} - 16\hat{\sigma}^z_{11}\hat{\sigma}^z_{17} + 16484\hat{\sigma}^z_{13}\hat{\sigma}^z_{15} - 32968\hat{\sigma}^z_{1}\hat{\sigma}^z_{28} -$
$8\hat{\sigma}^z_{11}\hat{\sigma}^z_{18} - 16\hat{\sigma}^z_{12}\hat{\sigma}^z_{17} - 16\hat{\sigma}^z_{13}\hat{\sigma}^z_{16} + 16484\hat{\sigma}^z_{14}\hat{\sigma}^z_{15} - 32968\hat{\sigma}^z_{1}\hat{\sigma}^z_{29} - 32968\hat{\sigma}^z_{2}\hat{\sigma}^z_{28} - 32968\hat{\sigma}^z_{8}\hat{\sigma}^z_{22} - 8\hat{\sigma}^z_{12}\hat{\sigma}^z_{18} -$
$16\hat{\sigma}^z_{14}\hat{\sigma}^z_{16} - 32968\hat{\sigma}^z_{1}\hat{\sigma}^z_{30} - 16\hat{\sigma}^z_{3}\hat{\sigma}^z_{28} - 16\hat{\sigma}^z_{9}\hat{\sigma}^z_{22} - 16\hat{\sigma}^z_{13}\hat{\sigma}^z_{18} - 16\hat{\sigma}^z_{14}\hat{\sigma}^z_{17} - 16\hat{\sigma}^z_{15}\hat{\sigma}^z_{16} - 32968\hat{\sigma}^z_{1}\hat{\sigma}^z_{31} - 32968\hat{\sigma}^z_{3}\hat{\sigma}^z_{29} -$
$32968\hat{\sigma}^z_{9}\hat{\sigma}^z_{23} - 16\hat{\sigma}^z_{10}\hat{\sigma}^z_{22} + 8\hat{\sigma}^z_{14}\hat{\sigma}^z_{18} - 8\hat{\sigma}^z_{15}\hat{\sigma}^z_{17} - 32968\hat{\sigma}^z_{1}\hat{\sigma}^z_{32} - 8\hat{\sigma}^z_{4}\hat{\sigma}^z_{29} - 8\hat{\sigma}^z_{5}\hat{\sigma}^z_{28} - 16\hat{\sigma}^z_{10}\hat{\sigma}^z_{23} - 8\hat{\sigma}^z_{11}\hat{\sigma}^z_{22} - 8\hat{\sigma}^z_{15}\hat{\sigma}^z_{18} -$
$32968\hat{\sigma}^z_{1}\hat{\sigma}^z_{33} - 32968\hat{\sigma}^z_{4}\hat{\sigma}^z_{30} - 8\hat{\sigma}^z_{6}\hat{\sigma}^z_{28} - 32968\hat{\sigma}^z_{10}\hat{\sigma}^z_{24} - 16\hat{\sigma}^z_{11}\hat{\sigma}^z_{23} - 32968\hat{\sigma}^z_{1}\hat{\sigma}^z_{34} - 8\hat{\sigma}^z_{5}\hat{\sigma}^z_{30} - 8\hat{\sigma}^z_{6}\hat{\sigma}^z_{29} - 8\hat{\sigma}^z_{7}\hat{\sigma}^z_{28} -$
$16\hat{\sigma}^z_{11}\hat{\sigma}^z_{24} - 32968\hat{\sigma}^z_{1}\hat{\sigma}^z_{35} - 32968\hat{\sigma}^z_{5}\hat{\sigma}^z_{31} - 8\hat{\sigma}^z_{6}\hat{\sigma}^z_{30} - 32968\hat{\sigma}^z_{1}\hat{\sigma}^z_{36} - 8\hat{\sigma}^z_{6}\hat{\sigma}^z_{31} - 8\hat{\sigma}^z_{7}\hat{\sigma}^z_{30} - 16\hat{\sigma}^z_{8}\hat{\sigma}^z_{29} - 16\hat{\sigma}^z_{9}\hat{\sigma}^z_{28} -$
$32968\hat{\sigma}^z_{12}\hat{\sigma}^z_{25} - 32968\hat{\sigma}^z_{1}\hat{\sigma}^z_{37} - 32968\hat{\sigma}^z_{6}\hat{\sigma}^z_{32} - 8\hat{\sigma}^z_{7}\hat{\sigma}^z_{31} - 16\hat{\sigma}^z_{10}\hat{\sigma}^z_{28} - 16\hat{\sigma}^z_{13}\hat{\sigma}^z_{25} - 32968\hat{\sigma}^z_{1}\hat{\sigma}^z_{38} - 8\hat{\sigma}^z_{7}\hat{\sigma}^z_{32} -$
$32968\hat{\sigma}^z_{13}\hat{\sigma}^z_{26} - 16\hat{\sigma}^z_{14}\hat{\sigma}^z_{25} - 16\hat{\sigma}^z_{11}\hat{\sigma}^z_{29} - 16\hat{\sigma}^z_{14}\hat{\sigma}^z_{26} - 8\hat{\sigma}^z_{15}\hat{\sigma}^z_{25} - 32968\hat{\sigma}^z_{2}\hat{\sigma}^z_{39} - 32968\hat{\sigma}^z_{8}\hat{\sigma}^z_{33} - 16\hat{\sigma}^z_{12}\hat{\sigma}^z_{29} - 16\hat{\sigma}^z_{13}\hat{\sigma}^z_{28} -$
$32968\hat{\sigma}^z_{14}\hat{\sigma}^z_{27} - 16\hat{\sigma}^z_{15}\hat{\sigma}^z_{26} - 32968\hat{\sigma}^z_{2}\hat{\sigma}^z_{40} - 32968\hat{\sigma}^z_{3}\hat{\sigma}^z_{39} - 16\hat{\sigma}^z_{9}\hat{\sigma}^z_{33} - 16\hat{\sigma}^z_{14}\hat{\sigma}^z_{28} - 16\hat{\sigma}^z_{15}\hat{\sigma}^z_{27} - 32968\hat{\sigma}^z_{2}\hat{\sigma}^z_{41} -$
$32968\hat{\sigma}^z_{9}\hat{\sigma}^z_{34} - 16\hat{\sigma}^z_{10}\hat{\sigma}^z_{33} - 16\hat{\sigma}^z_{14}\hat{\sigma}^z_{29} - 16\hat{\sigma}^z_{15}\hat{\sigma}^z_{28} - 32968\hat{\sigma}^z_{2}\hat{\sigma}^z_{42} - 32968\hat{\sigma}^z_{4}\hat{\sigma}^z_{40} - 8\hat{\sigma}^z_{5}\hat{\sigma}^z_{39} - 16\hat{\sigma}^z_{10}\hat{\sigma}^z_{34} - 16\hat{\sigma}^z_{11}\hat{\sigma}^z_{33} -$
$8\hat{\sigma}^z_{15}\hat{\sigma}^z_{29} - 32968\hat{\sigma}^z_{2}\hat{\sigma}^z_{43} - 8\hat{\sigma}^z_{5}\hat{\sigma}^z_{40} - 8\hat{\sigma}^z_{6}\hat{\sigma}^z_{39} - 32968\hat{\sigma}^z_{10}\hat{\sigma}^z_{35} - 16\hat{\sigma}^z_{11}\hat{\sigma}^z_{34} - 32968\hat{\sigma}^z_{2}\hat{\sigma}^z_{44} - 32968\hat{\sigma}^z_{5}\hat{\sigma}^z_{41} - 8\hat{\sigma}^z_{6}\hat{\sigma}^z_{40} -$
$8\hat{\sigma}^z_{7}\hat{\sigma}^z_{39} - 16\hat{\sigma}^z_{11}\hat{\sigma}^z_{35} - 32968\hat{\sigma}^z_{2}\hat{\sigma}^z_{45} - 8\hat{\sigma}^z_{6}\hat{\sigma}^z_{41} - 32968\hat{\sigma}^z_{2}\hat{\sigma}^z_{46} - 32968\hat{\sigma}^z_{6}\hat{\sigma}^z_{42} - 8\hat{\sigma}^z_{7}\hat{\sigma}^z_{41} - 16\hat{\sigma}^z_{9}\hat{\sigma}^z_{39} - 32968\hat{\sigma}^z_{12}\hat{\sigma}^z_{36} -$
$32968\hat{\sigma}^z_{2}\hat{\sigma}^z_{47} - 8\hat{\sigma}^z_{7}\hat{\sigma}^z_{42} - 16\hat{\sigma}^z_{10}\hat{\sigma}^z_{39} - 16\hat{\sigma}^z_{13}\hat{\sigma}^z_{36} - 32968\hat{\sigma}^z_{2}\hat{\sigma}^z_{48} - 32968\hat{\sigma}^z_{13}\hat{\sigma}^z_{37} - 16\hat{\sigma}^z_{14}\hat{\sigma}^z_{36} - 32968\hat{\sigma}^z_{8}\hat{\sigma}^z_{43} -$
$16\hat{\sigma}^z_{14}\hat{\sigma}^z_{37} - 16\hat{\sigma}^z_{15}\hat{\sigma}^z_{36} - 32968\hat{\sigma}^z_{3}\hat{\sigma}^z_{49} - 16\hat{\sigma}^z_{9}\hat{\sigma}^z_{43} - 16\hat{\sigma}^z_{13}\hat{\sigma}^z_{39} - 32968\hat{\sigma}^z_{14}\hat{\sigma}^z_{38} - 16\hat{\sigma}^z_{15}\hat{\sigma}^z_{37} - 32968\hat{\sigma}^z_{3}\hat{\sigma}^z_{50} -$
$32968\hat{\sigma}^z_{4}\hat{\sigma}^z_{49} - 32968\hat{\sigma}^z_{9}\hat{\sigma}^z_{44} - 16\hat{\sigma}^z_{10}\hat{\sigma}^z_{43} - 16\hat{\sigma}^z_{14}\hat{\sigma}^z_{39} - 16\hat{\sigma}^z_{15}\hat{\sigma}^z_{38} - 32968\hat{\sigma}^z_{3}\hat{\sigma}^z_{51} - 8\hat{\sigma}^z_{5}\hat{\sigma}^z_{49} - 16\hat{\sigma}^z_{10}\hat{\sigma}^z_{44} - 16\hat{\sigma}^z_{15}\hat{\sigma}^z_{39} -$
$32968\hat{\sigma}^z_{3}\hat{\sigma}^z_{52} - 32968\hat{\sigma}^z_{5}\hat{\sigma}^z_{50} - 32968\hat{\sigma}^z_{10}\hat{\sigma}^z_{45} - 16\hat{\sigma}^z_{11}\hat{\sigma}^z_{44} + 16\hat{\sigma}^z_{16}\hat{\sigma}^z_{39} - 32968\hat{\sigma}^z_{3}\hat{\sigma}^z_{53} - 8\hat{\sigma}^z_{6}\hat{\sigma}^z_{50} - 4\hat{\sigma}^z_{7}\hat{\sigma}^z_{49} -$
$16\hat{\sigma}^z_{11}\hat{\sigma}^z_{45} - 32968\hat{\sigma}^z_{3}\hat{\sigma}^z_{54} - 32968\hat{\sigma}^z_{6}\hat{\sigma}^z_{51} - 32968\hat{\sigma}^z_{3}\hat{\sigma}^z_{55} - 8\hat{\sigma}^z_{7}\hat{\sigma}^z_{51} - 32968\hat{\sigma}^z_{12}\hat{\sigma}^z_{46} - 32968\hat{\sigma}^z_{3}\hat{\sigma}^z_{56} - 16\hat{\sigma}^z_{13}\hat{\sigma}^z_{46} -$
$32968\hat{\sigma}^z_{3}\hat{\sigma}^z_{57} - 32968\hat{\sigma}^z_{8}\hat{\sigma}^z_{52} - 32968\hat{\sigma}^z_{13}\hat{\sigma}^z_{47} - 16\hat{\sigma}^z_{14}\hat{\sigma}^z_{46} - 16\hat{\sigma}^z_{9}\hat{\sigma}^z_{52} - 16\hat{\sigma}^z_{14}\hat{\sigma}^z_{47} - 32968\hat{\sigma}^z_{4}\hat{\sigma}^z_{58} - 32968\hat{\sigma}^z_{9}\hat{\sigma}^z_{53} -$
$32968\hat{\sigma}^z_{14}\hat{\sigma}^z_{48} - 16\hat{\sigma}^z_{15}\hat{\sigma}^z_{47} - 32968\hat{\sigma}^z_{4}\hat{\sigma}^z_{59} - 32968\hat{\sigma}^z_{5}\hat{\sigma}^z_{58} - 16\hat{\sigma}^z_{10}\hat{\sigma}^z_{53} - 8\hat{\sigma}^z_{11}\hat{\sigma}^z_{52} - 16\hat{\sigma}^z_{15}\hat{\sigma}^z_{48} - 32968\hat{\sigma}^z_{4}\hat{\sigma}^z_{60} -$
$32968\hat{\sigma}^z_{10}\hat{\sigma}^z_{54} - 32968\hat{\sigma}^z_{4}\hat{\sigma}^z_{61} - 32968\hat{\sigma}^z_{6}\hat{\sigma}^z_{59} - 24\hat{\sigma}^z_{7}\hat{\sigma}^z_{58} - 16\hat{\sigma}^z_{11}\hat{\sigma}^z_{54} - 32968\hat{\sigma}^z_{4}\hat{\sigma}^z_{62} + 8\hat{\sigma}^z_{7}\hat{\sigma}^z_{59} - 32968\hat{\sigma}^z_{4}\hat{\sigma}^z_{63} -$
$32968\hat{\sigma}^z_{7}\hat{\sigma}^z_{60} - 16\hat{\sigma}^z_{8}\hat{\sigma}^z_{59} - 16\hat{\sigma}^z_{9}\hat{\sigma}^z_{58} - 32968\hat{\sigma}^z_{12}\hat{\sigma}^z_{55} - 32968\hat{\sigma}^z_{4}\hat{\sigma}^z_{64} - 8\hat{\sigma}^z_{8}\hat{\sigma}^z_{60} - 16\hat{\sigma}^z_{10}\hat{\sigma}^z_{58} - 16\hat{\sigma}^z_{13}\hat{\sigma}^z_{55} -$
$32968\hat{\sigma}^z_{8}\hat{\sigma}^z_{61} - 32968\hat{\sigma}^z_{13}\hat{\sigma}^z_{56} - 32968\hat{\sigma}^z_{5}\hat{\sigma}^z_{65} - 16\hat{\sigma}^z_{11}\hat{\sigma}^z_{59} - 16\hat{\sigma}^z_{14}\hat{\sigma}^z_{56} - 8\hat{\sigma}^z_{15}\hat{\sigma}^z_{55} - 32968\hat{\sigma}^z_{5}\hat{\sigma}^z_{66} - 32968\hat{\sigma}^z_{6}\hat{\sigma}^z_{65} -$
$32968\hat{\sigma}^z_{9}\hat{\sigma}^z_{62} - 16\hat{\sigma}^z_{10}\hat{\sigma}^z_{61} - 8\hat{\sigma}^z_{11}\hat{\sigma}^z_{60} - 16\hat{\sigma}^z_{12}\hat{\sigma}^z_{59} - 16\hat{\sigma}^z_{13}\hat{\sigma}^z_{58} - 32968\hat{\sigma}^z_{14}\hat{\sigma}^z_{57} - 32968\hat{\sigma}^z_{5}\hat{\sigma}^z_{67} - 16\hat{\sigma}^z_{7}\hat{\sigma}^z_{65} -$
$8\hat{\sigma}^z_{11}\hat{\sigma}^z_{61} - 8\hat{\sigma}^z_{12}\hat{\sigma}^z_{60} - 16\hat{\sigma}^z_{14}\hat{\sigma}^z_{58} - 16\hat{\sigma}^z_{15}\hat{\sigma}^z_{57} - 32968\hat{\sigma}^z_{5}\hat{\sigma}^z_{68} - 32968\hat{\sigma}^z_{7}\hat{\sigma}^z_{66} - 16\hat{\sigma}^z_{11}\hat{\sigma}^z_{62} - 16\hat{\sigma}^z_{13}\hat{\sigma}^z_{60} - 16\hat{\sigma}^z_{14}\hat{\sigma}^z_{59} -$
$16\hat{\sigma}^z_{15}\hat{\sigma}^z_{58} - 32968\hat{\sigma}^z_{5}\hat{\sigma}^z_{69} - 16\hat{\sigma}^z_{8}\hat{\sigma}^z_{66} - 16\hat{\sigma}^z_{9}\hat{\sigma}^z_{65} + 8\hat{\sigma}^z_{14}\hat{\sigma}^z_{60} - 8\hat{\sigma}^z_{15}\hat{\sigma}^z_{59} + 8\hat{\sigma}^z_{16}\hat{\sigma}^z_{58} - 32968\hat{\sigma}^z_{5}\hat{\sigma}^z_{70} - 32968\hat{\sigma}^z_{8}\hat{\sigma}^z_{67} -$
$16\hat{\sigma}^z_{10}\hat{\sigma}^z_{65} - 32968\hat{\sigma}^z_{12}\hat{\sigma}^z_{63} - 8\hat{\sigma}^z_{15}\hat{\sigma}^z_{60} - 32968\hat{\sigma}^z_{5}\hat{\sigma}^z_{71} - 16\hat{\sigma}^z_{9}\hat{\sigma}^z_{67} + 8\hat{\sigma}^z_{17}\hat{\sigma}^z_{59} - 32968\hat{\sigma}^z_{5}\hat{\sigma}^z_{72} - 32968\hat{\sigma}^z_{9}\hat{\sigma}^z_{68} -$
$16\hat{\sigma}^z_{11}\hat{\sigma}^z_{66} - 32968\hat{\sigma}^z_{13}\hat{\sigma}^z_{64} - 16\hat{\sigma}^z_{14}\hat{\sigma}^z_{63} - 16\hat{\sigma}^z_{10}\hat{\sigma}^z_{68} - 16\hat{\sigma}^z_{11}\hat{\sigma}^z_{67} - 16\hat{\sigma}^z_{12}\hat{\sigma}^z_{66} - 16\hat{\sigma}^z_{13}\hat{\sigma}^z_{65} - 8\hat{\sigma}^z_{15}\hat{\sigma}^z_{63} + 8\hat{\sigma}^z_{18}\hat{\sigma}^z_{60} -$
$32968\hat{\sigma}^z_{6}\hat{\sigma}^z_{73} - 32968\hat{\sigma}^z_{10}\hat{\sigma}^z_{69} - 16\hat{\sigma}^z_{14}\hat{\sigma}^z_{65} - 16\hat{\sigma}^z_{15}\hat{\sigma}^z_{64} - 32968\hat{\sigma}^z_{6}\hat{\sigma}^z_{74} - 32968\hat{\sigma}^z_{7}\hat{\sigma}^z_{73} - 16\hat{\sigma}^z_{11}\hat{\sigma}^z_{69} - 16\hat{\sigma}^z_{14}\hat{\sigma}^z_{66} -$
$16\hat{\sigma}^z_{15}\hat{\sigma}^z_{65} - 32968\hat{\sigma}^z_{6}\hat{\sigma}^z_{75} - 8\hat{\sigma}^z_{15}\hat{\sigma}^z_{66} + 8\hat{\sigma}^z_{16}\hat{\sigma}^z_{65} - 32968\hat{\sigma}^z_{6}\hat{\sigma}^z_{76} - 32968\hat{\sigma}^z_{8}\hat{\sigma}^z_{74} - 16\hat{\sigma}^z_{9}\hat{\sigma}^z_{73} - 32968\hat{\sigma}^z_{12}\hat{\sigma}^z_{70} -$
$32968\hat{\sigma}^z_{6}\hat{\sigma}^z_{77} - 16\hat{\sigma}^z_{9}\hat{\sigma}^z_{74} - 16\hat{\sigma}^z_{10}\hat{\sigma}^z_{73} - 16\hat{\sigma}^z_{13}\hat{\sigma}^z_{70} + 8\hat{\sigma}^z_{17}\hat{\sigma}^z_{66} - 32968\hat{\sigma}^z_{6}\hat{\sigma}^z_{78} - 32968\hat{\sigma}^z_{9}\hat{\sigma}^z_{75} - 16\hat{\sigma}^z_{10}\hat{\sigma}^z_{74} -$
$32968\hat{\sigma}^z_{13}\hat{\sigma}^z_{71} - 32968\hat{\sigma}^z_{6}\hat{\sigma}^z_{79} - 16\hat{\sigma}^z_{10}\hat{\sigma}^z_{75} + 16\hat{\sigma}^z_{11}\hat{\sigma}^z_{74} - 16\hat{\sigma}^z_{14}\hat{\sigma}^z_{71} - 16\hat{\sigma}^z_{15}\hat{\sigma}^z_{70} - 32968\hat{\sigma}^z_{10}\hat{\sigma}^z_{76} - 16\hat{\sigma}^z_{11}\hat{\sigma}^z_{75} -$
$16\hat{\sigma}^z_{13}\hat{\sigma}^z_{73} - 32968\hat{\sigma}^z_{14}\hat{\sigma}^z_{72} + 8\hat{\sigma}^z_{28}\hat{\sigma}^z_{58} - 32968\hat{\sigma}^z_{7}\hat{\sigma}^z_{80} - 16\hat{\sigma}^z_{11}\hat{\sigma}^z_{76} - 16\hat{\sigma}^z_{14}\hat{\sigma}^z_{73} - 16\hat{\sigma}^z_{15}\hat{\sigma}^z_{72} - 32968\hat{\sigma}^z_{7}\hat{\sigma}^z_{81} -$
$32968\hat{\sigma}^z_{8}\hat{\sigma}^z_{80} - 16\hat{\sigma}^z_{15}\hat{\sigma}^z_{73} + 8\hat{\sigma}^z_{29}\hat{\sigma}^z_{59} - 32968\hat{\sigma}^z_{7}\hat{\sigma}^z_{82} - 16\hat{\sigma}^z_{9}\hat{\sigma}^z_{80} - 32968\hat{\sigma}^z_{12}\hat{\sigma}^z_{77} + 8\hat{\sigma}^z_{16}\hat{\sigma}^z_{73} - 32968\hat{\sigma}^z_{7}\hat{\sigma}^z_{83} -$

$$
\begin{aligned}
&32968\hat{\sigma}^z_{9}\hat{\sigma}^z_{81} - 16\hat{\sigma}^z_{13}\hat{\sigma}^z_{77} - 32968\hat{\sigma}^z_{7}\hat{\sigma}^z_{84} - 16\hat{\sigma}^z_{10}\hat{\sigma}^z_{81} - 8\hat{\sigma}^z_{11}\hat{\sigma}^z_{80} - 32968\hat{\sigma}^z_{13}\hat{\sigma}^z_{78} - 16\hat{\sigma}^z_{14}\hat{\sigma}^z_{77} - 32968\hat{\sigma}^z_{7}\hat{\sigma}^z_{85} - \\
&32968\hat{\sigma}^z_{10}\hat{\sigma}^z_{82} - 16\hat{\sigma}^z_{14}\hat{\sigma}^z_{78} + 16\hat{\sigma}^z_{15}\hat{\sigma}^z_{77} - 16\hat{\sigma}^z_{11}\hat{\sigma}^z_{82} - 32968\hat{\sigma}^z_{14}\hat{\sigma}^z_{79} - 16\hat{\sigma}^z_{15}\hat{\sigma}^z_{78} + 8\hat{\sigma}^z_{28}\hat{\sigma}^z_{65} - 32968\hat{\sigma}^z_{8}\hat{\sigma}^z_{86} - \\
&16\hat{\sigma}^z_{15}\hat{\sigma}^z_{79} - 32968\hat{\sigma}^z_{8}\hat{\sigma}^z_{87} - 32968\hat{\sigma}^z_{9}\hat{\sigma}^z_{86} - 32968\hat{\sigma}^z_{12}\hat{\sigma}^z_{83} + 8\hat{\sigma}^z_{29}\hat{\sigma}^z_{66} - 32968\hat{\sigma}^z_{8}\hat{\sigma}^z_{88} - 16\hat{\sigma}^z_{13}\hat{\sigma}^z_{83} - 32968\hat{\sigma}^z_{8}\hat{\sigma}^z_{89} - \\
&32968\hat{\sigma}^z_{10}\hat{\sigma}^z_{87} - 32\hat{\sigma}^z_{11}\hat{\sigma}^z_{86} - 32968\hat{\sigma}^z_{13}\hat{\sigma}^z_{84} + 8\hat{\sigma}^z_{39}\hat{\sigma}^z_{58} - 32968\hat{\sigma}^z_{8}\hat{\sigma}^z_{90} - 32\hat{\sigma}^z_{11}\hat{\sigma}^z_{87} - 16\hat{\sigma}^z_{14}\hat{\sigma}^z_{84} - 8\hat{\sigma}^z_{15}\hat{\sigma}^z_{83} - \\
&32968\hat{\sigma}^z_{11}\hat{\sigma}^z_{88} - 32\hat{\sigma}^z_{12}\hat{\sigma}^z_{87} - 32\hat{\sigma}^z_{13}\hat{\sigma}^z_{86} - 32968\hat{\sigma}^z_{14}\hat{\sigma}^z_{85} - 32968\hat{\sigma}^z_{9}\hat{\sigma}^z_{91} - 16\hat{\sigma}^z_{12}\hat{\sigma}^z_{88} - 32\hat{\sigma}^z_{14}\hat{\sigma}^z_{86} - 16\hat{\sigma}^z_{15}\hat{\sigma}^z_{85} - \\
&32968\hat{\sigma}^z_{9}\hat{\sigma}^z_{92} - 32968\hat{\sigma}^z_{10}\hat{\sigma}^z_{91} - 32968\hat{\sigma}^z_{12}\hat{\sigma}^z_{89} - 32\hat{\sigma}^z_{13}\hat{\sigma}^z_{88} - 32\hat{\sigma}^z_{14}\hat{\sigma}^z_{87} - 32\hat{\sigma}^z_{15}\hat{\sigma}^z_{86} + 8\hat{\sigma}^z_{28}\hat{\sigma}^z_{73} - 32968\hat{\sigma}^z_{9}\hat{\sigma}^z_{93} + \\
&16\hat{\sigma}^z_{14}\hat{\sigma}^z_{88} - 16\hat{\sigma}^z_{15}\hat{\sigma}^z_{87} + 16\hat{\sigma}^z_{16}\hat{\sigma}^z_{86} - 32968\hat{\sigma}^z_{9}\hat{\sigma}^z_{94} - 32968\hat{\sigma}^z_{11}\hat{\sigma}^z_{92} - 32968\hat{\sigma}^z_{13}\hat{\sigma}^z_{90} - 32\hat{\sigma}^z_{14}\hat{\sigma}^z_{89} - 16\hat{\sigma}^z_{15}\hat{\sigma}^z_{88} - \\
&32968\hat{\sigma}^z_{9}\hat{\sigma}^z_{95} - 32\hat{\sigma}^z_{12}\hat{\sigma}^z_{92} - 32\hat{\sigma}^z_{13}\hat{\sigma}^z_{91} - 16\hat{\sigma}^z_{15}\hat{\sigma}^z_{89} + 16\hat{\sigma}^z_{17}\hat{\sigma}^z_{87} + 8\hat{\sigma}^z_{39}\hat{\sigma}^z_{65} - 32968\hat{\sigma}^z_{12}\hat{\sigma}^z_{93} - 32\hat{\sigma}^z_{14}\hat{\sigma}^z_{91} - 32\hat{\sigma}^z_{15}\hat{\sigma}^z_{90} - \\
&32968\hat{\sigma}^z_{10}\hat{\sigma}^z_{96} - 32\hat{\sigma}^z_{13}\hat{\sigma}^z_{93} - 32\hat{\sigma}^z_{14}\hat{\sigma}^z_{92} - 32\hat{\sigma}^z_{15}\hat{\sigma}^z_{91} + 16\hat{\sigma}^z_{18}\hat{\sigma}^z_{88} - 32968\hat{\sigma}^z_{10}\hat{\sigma}^z_{97} - 32968\hat{\sigma}^z_{11}\hat{\sigma}^z_{96} - 32968\hat{\sigma}^z_{13}\hat{\sigma}^z_{94} - \\
&16\hat{\sigma}^z_{15}\hat{\sigma}^z_{92} + 16\hat{\sigma}^z_{16}\hat{\sigma}^z_{91} - 32968\hat{\sigma}^z_{10}\hat{\sigma}^z_{98} - 32\hat{\sigma}^z_{14}\hat{\sigma}^z_{94} - 32968\hat{\sigma}^z_{10}\hat{\sigma}^z_{99} - 32968\hat{\sigma}^z_{12}\hat{\sigma}^z_{97} - 32\hat{\sigma}^z_{13}\hat{\sigma}^z_{96} - 32968\hat{\sigma}^z_{14}\hat{\sigma}^z_{95} + \\
&16\hat{\sigma}^z_{17}\hat{\sigma}^z_{92} - 32\hat{\sigma}^z_{13}\hat{\sigma}^z_{97} - 32\hat{\sigma}^z_{14}\hat{\sigma}^z_{96} - 32\hat{\sigma}^z_{15}\hat{\sigma}^z_{95} - 32968\hat{\sigma}^z_{11}\hat{\sigma}^z_{100} - 32968\hat{\sigma}^z_{13}\hat{\sigma}^z_{98} - 32\hat{\sigma}^z_{15}\hat{\sigma}^z_{96} - 32968\hat{\sigma}^z_{11}\hat{\sigma}^z_{101} - \\
&32968\hat{\sigma}^z_{12}\hat{\sigma}^z_{100} - 32\hat{\sigma}^z_{14}\hat{\sigma}^z_{98} + 16\hat{\sigma}^z_{16}\hat{\sigma}^z_{96} + 8\hat{\sigma}^z_{39}\hat{\sigma}^z_{73} - 32968\hat{\sigma}^z_{14}\hat{\sigma}^z_{99} - 32968\hat{\sigma}^z_{12}\hat{\sigma}^z_{102} - 32968\hat{\sigma}^z_{13}\hat{\sigma}^z_{101} - 32\hat{\sigma}^z_{14}\hat{\sigma}^z_{100} - \\
&32\hat{\sigma}^z_{15}\hat{\sigma}^z_{99} + 16\hat{\sigma}^z_{28}\hat{\sigma}^z_{86} - 32968\hat{\sigma}^z_{12}\hat{\sigma}^z_{103} - 32968\hat{\sigma}^z_{13}\hat{\sigma}^z_{102} - 16\hat{\sigma}^z_{15}\hat{\sigma}^z_{100} - 32968\hat{\sigma}^z_{12}\hat{\sigma}^z_{104} - 32\hat{\sigma}^z_{15}\hat{\sigma}^z_{101} + 16\hat{\sigma}^z_{29}\hat{\sigma}^z_{87} - \\
&32968\hat{\sigma}^z_{14}\hat{\sigma}^z_{103} - 96\hat{\sigma}^z_{15}\hat{\sigma}^z_{102} - 32968\hat{\sigma}^z_{13}\hat{\sigma}^z_{105} + 16\hat{\sigma}^z_{16}\hat{\sigma}^z_{102} - 32968\hat{\sigma}^z_{13}\hat{\sigma}^z_{106} - 32968\hat{\sigma}^z_{14}\hat{\sigma}^z_{105} - 32968\hat{\sigma}^z_{15}\hat{\sigma}^z_{104} + \\
&16\hat{\sigma}^z_{28}\hat{\sigma}^z_{91} - 64\hat{\sigma}^z_{15}\hat{\sigma}^z_{105} + 16\hat{\sigma}^z_{17}\hat{\sigma}^z_{103} - 32968\hat{\sigma}^z_{14}\hat{\sigma}^z_{107} - 32968\hat{\sigma}^z_{15}\hat{\sigma}^z_{106} + 16\hat{\sigma}^z_{16}\hat{\sigma}^z_{105} + 16\hat{\sigma}^z_{29}\hat{\sigma}^z_{92} - 32968\hat{\sigma}^z_{15}\hat{\sigma}^z_{107} + \\
&16\hat{\sigma}^z_{18}\hat{\sigma}^z_{104} + 16\hat{\sigma}^z_{16}\hat{\sigma}^z_{107} + 16\hat{\sigma}^z_{17}\hat{\sigma}^z_{106} + 16\hat{\sigma}^z_{28}\hat{\sigma}^z_{96} + 16\hat{\sigma}^z_{39}\hat{\sigma}^z_{86} + 16\hat{\sigma}^z_{28}\hat{\sigma}^z_{102} + 16\hat{\sigma}^z_{39}\hat{\sigma}^z_{91} + 16\hat{\sigma}^z_{58}\hat{\sigma}^z_{73} + 16\hat{\sigma}^z_{29}\hat{\sigma}^z_{103} + \\
&16\hat{\sigma}^z_{28}\hat{\sigma}^z_{105} + 16\hat{\sigma}^z_{28}\hat{\sigma}^z_{107} + 16\hat{\sigma}^z_{29}\hat{\sigma}^z_{106} + 16\hat{\sigma}^z_{39}\hat{\sigma}^z_{96} + 16\hat{\sigma}^z_{39}\hat{\sigma}^z_{102} + 16\hat{\sigma}^z_{39}\hat{\sigma}^z_{105} + 16\hat{\sigma}^z_{58}\hat{\sigma}^z_{86} + 16\hat{\sigma}^z_{39}\hat{\sigma}^z_{107} + 16\hat{\sigma}^z_{59}\hat{\sigma}^z_{87} + \\
&16\hat{\sigma}^z_{60}\hat{\sigma}^z_{88} + 16\hat{\sigma}^z_{58}\hat{\sigma}^z_{91} + 16\hat{\sigma}^z_{59}\hat{\sigma}^z_{92} + 16\hat{\sigma}^z_{65}\hat{\sigma}^z_{86} + 16\hat{\sigma}^z_{66}\hat{\sigma}^z_{87} + 16\hat{\sigma}^z_{58}\hat{\sigma}^z_{96} + 16\hat{\sigma}^z_{65}\hat{\sigma}^z_{91} + 16\hat{\sigma}^z_{66}\hat{\sigma}^z_{92} + 16\hat{\sigma}^z_{73}\hat{\sigma}^z_{86} + \\
&16\hat{\sigma}^z_{58}\hat{\sigma}^z_{102} + 16\hat{\sigma}^z_{65}\hat{\sigma}^z_{96} + 16\hat{\sigma}^z_{59}\hat{\sigma}^z_{103} + 16\hat{\sigma}^z_{58}\hat{\sigma}^z_{105} + 16\hat{\sigma}^z_{60}\hat{\sigma}^z_{104} + 16\hat{\sigma}^z_{73}\hat{\sigma}^z_{91} + 16\hat{\sigma}^z_{58}\hat{\sigma}^z_{107} + 16\hat{\sigma}^z_{59}\hat{\sigma}^z_{106} + 16\hat{\sigma}^z_{65}\hat{\sigma}^z_{102} + \\
&16\hat{\sigma}^z_{66}\hat{\sigma}^z_{103} + 16\hat{\sigma}^z_{73}\hat{\sigma}^z_{96} + 16\hat{\sigma}^z_{65}\hat{\sigma}^z_{105} + 16\hat{\sigma}^z_{65}\hat{\sigma}^z_{107} + 16\hat{\sigma}^z_{66}\hat{\sigma}^z_{106} + 16\hat{\sigma}^z_{73}\hat{\sigma}^z_{102} + 16\hat{\sigma}^z_{73}\hat{\sigma}^z_{105} + 16\hat{\sigma}^z_{73}\hat{\sigma}^z_{107} + 64\hat{\sigma}^z_{86}\hat{\sigma}^z_{96} + \\
&32\hat{\sigma}^z_{86}\hat{\sigma}^z_{102} + 32\hat{\sigma}^z_{87}\hat{\sigma}^z_{103} + 32\hat{\sigma}^z_{86}\hat{\sigma}^z_{105} + 32\hat{\sigma}^z_{88}\hat{\sigma}^z_{104} + 32\hat{\sigma}^z_{86}\hat{\sigma}^z_{107} + 32\hat{\sigma}^z_{87}\hat{\sigma}^z_{106} + 32\hat{\sigma}^z_{91}\hat{\sigma}^z_{102} + 32\hat{\sigma}^z_{92}\hat{\sigma}^z_{103} + 32\hat{\sigma}^z_{91}\hat{\sigma}^z_{105} + \\
&32\hat{\sigma}^z_{91}\hat{\sigma}^z_{107} + 32\hat{\sigma}^z_{92}\hat{\sigma}^z_{106} + 32\hat{\sigma}^z_{96}\hat{\sigma}^z_{102} + 32\hat{\sigma}^z_{96}\hat{\sigma}^z_{105} + 32\hat{\sigma}^z_{96}\hat{\sigma}^z_{107} + 64\hat{\sigma}^z_{102}\hat{\sigma}^z_{107} + 4551232
\end{aligned}
$$
